# Performance-Enhancing Asymmetric Catalysis Driven by Achiral Counterion Design

**DOI:** 10.1021/jacs.5c05263

**Published:** 2025-05-14

**Authors:** Zihang Deng, Jenna L. Payne, Mahesh Vishe, Julius E. L. Jan, Cody M. Funk, Jeffrey N. Johnston

**Affiliations:** Department of Chemistry and Vanderbilt Institute of Chemical Biology, 5718 Vanderbilt University, Nashville, Tennessee 37235, United States

## Abstract

The development of highly enantioselective reactions often requires the adventitious discovery of a promising chiral catalyst and its resource-intensive optimization to high selectivity and generality. We report an approach less dependent on happenstance, whereby the performance of a single chiral ligand is enhanced not by modification of the architecturally complex chiral features but instead by an achiral counteranion. Critical to this strategy and its general application is the tactical development of *N*-aryl trifluoromethyl sulfonamide Brønsted acid donors and their ability to unlock the full enantioselectivity potential of a single chiral Brønsted basic ligand for the enantioselective addition of azide to nitroalkene.

## Graphical abstract

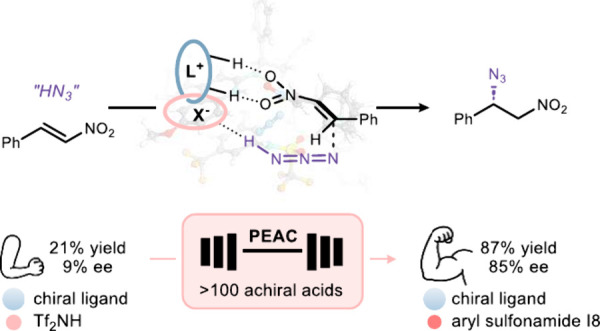

Asymmetric catalysis is critical to the preparation of single-enantiomer therapeutics and novel materials where the desired behavior is maximal with one of the two mirror image forms.^1^ The discovery of new asymmetric catalysts is often dependent on the identification of a promising hit under a set of defined conditions, evaluated with a specific substrate or a representative group of ‘informer’ substrates. The pursuit of an optimal catalyst following the initial ‘hit’ involves iterative modification of the chiral ligand (Scheme 1A, left), requiring extensive synthesis. The effort required to probe a chiral architecture is intense, even when the synthetic process is modular and dependent on readily available chiral building blocks. While this approach can be hypothesis-driven and based on mechanistic insights of catalyst structure–activity relationships, it is often an intensive hunt for subtle changes to the chiral ligand that might lead to large increases in performance, particularly since knowledge of all activity- and selectivity-determining elementary reaction rates cannot be known *a priori*. We questioned whether the achiral counterion of a chiral ion pair catalyst could be designed to improve the discovery and optimization process, based on the hypothesis that even a dissociated counterion might still induce a favorable conformational change to the chiral element, or create a more defined substrate-binding pocket (Scheme 1B).^2^ This approach reduces the dependence on covalent bond modification of the chiral ligand to tune catalyst performance, where the influence of critical remote substituent effects can depend on multistep synthesis. A prominent example of the latter is the chiral phosphoric acid (CPA) design where unique 3,3′-substituents are critical for performance.^3^

**Scheme 1:**
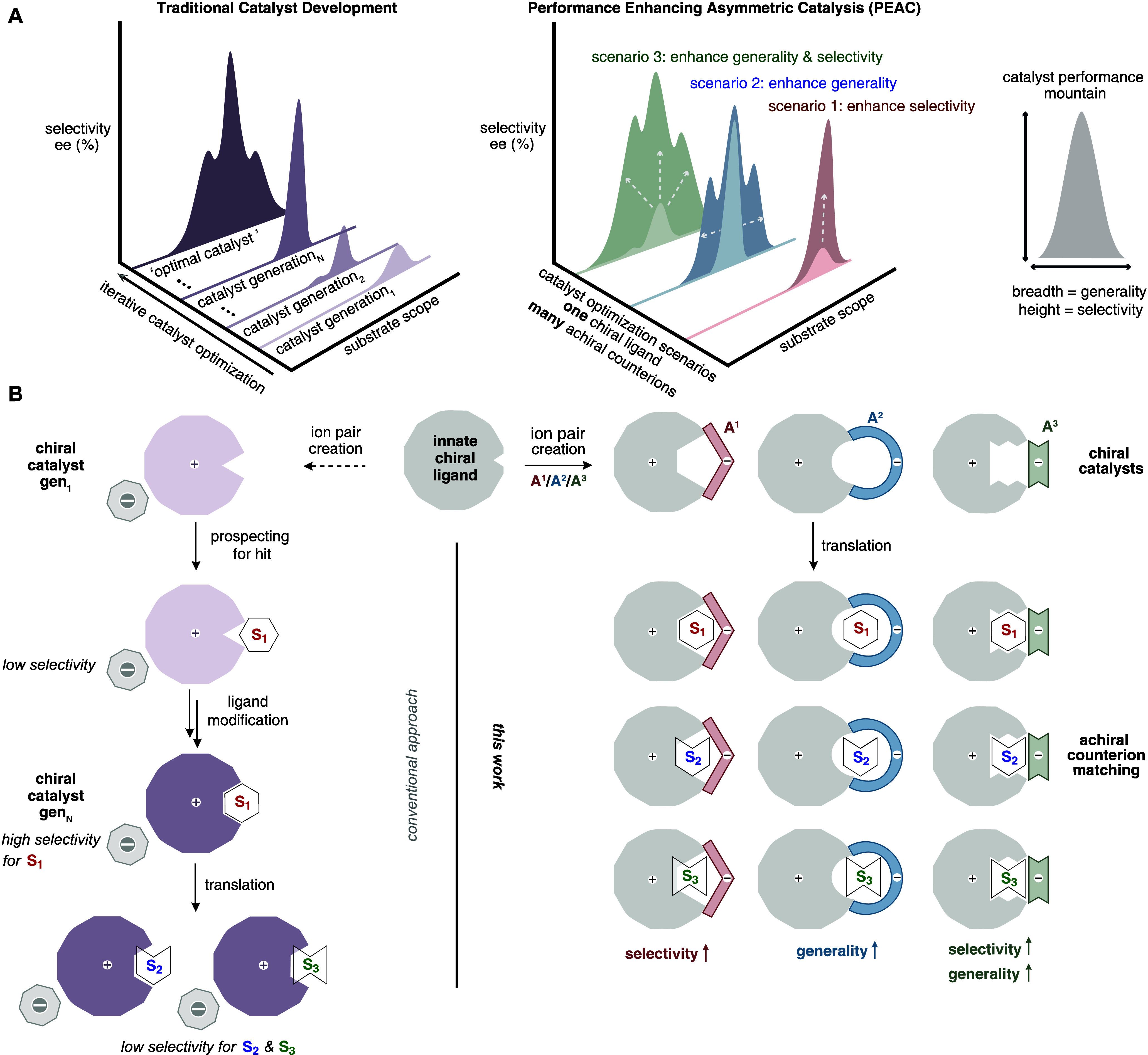
Performance Enhancing Asymmetric Catalysis (PEAC)^a^

a (A) Illustration of catalyst development approaches for enantioselective catalysis, highlighting iterative chiral ligand engineering to improve selectivity and generality (left) and the PEAC approach dependent on a single chiral ligand whose peak performance is developed by screening an achiral co-catalyst (right). (B) Schematic for catalyst development processes. Left: Current paradigm for ion pairing asymmetric catalysis, focusing on chiral ligand modifications (depicted by varying ligand shapes). Right: PEAC approach: achiral donors form tight ion pairs with the catalyst, perturbing ligand topology. This is reflected in changes to the ligand-counterion cavity size and shape, thereby enhancing catalyst performance.

We report herein an ion pair catalyst that uses a hypervariable achiral modifier. Critical to success is the modifier’s balance of acid strength to engage a basic chiral ligand through positive charge generation and the ability to remain associated in order to affect ligand–substrate binding, thereby enhancing either selectivity or generality, or potentially both (Scheme 1A, right). This approach decouples the primacy of the chiral ligand from the activity of a Brønsted acid by using achiral aryl sulfonamides of varying acid strength and structure. The design employs an aryl trifluorosulfonamide (triflamide) to confer an activating hydrogen bond to the chiral ligand while remaining at a close distance to perturb the topology of the chiral pocket. In cases where a chiral ligand is ineffective initially, we show that selectivity can emerge through the use of a specific aryl triflamide. This performance-enhancing asymmetric catalysis (PEAC) approach is an answer to the growing expectation for more general catalysts, single catalyst systems that exhibit broad scope, or the evolution of catalysts to ‘privileged catalyst’ stature.^4^

This initial design is predicated on the use of polar ionic hydrogen bonding (Scheme 2A), a feature that is rare^5^ in asymmetric catalysis when compared to polar covalent hydrogen bonds, but is reminiscent of ion pairing catalysis.^6^ Unlike prevailing approaches that focus on stronger chiral Brønsted acids,^7^ we reasoned that selectivity can be optimized by crafting the electronic and steric nature of an achiral counterion (Scheme 2A) within a narrow p*K*_a_ range. Importantly, this design borrows features from triflimidic acid (Scheme 2B), a Brønsted acid used in many enantioselective reactions to leverage the dissociated nature of its triflimide counteranion.^8^ As an orthogonal element, the aryl triflamide offers generous chemical space in which to reveal activity and selectivity effects (Scheme 2C).

**Scheme 2:**
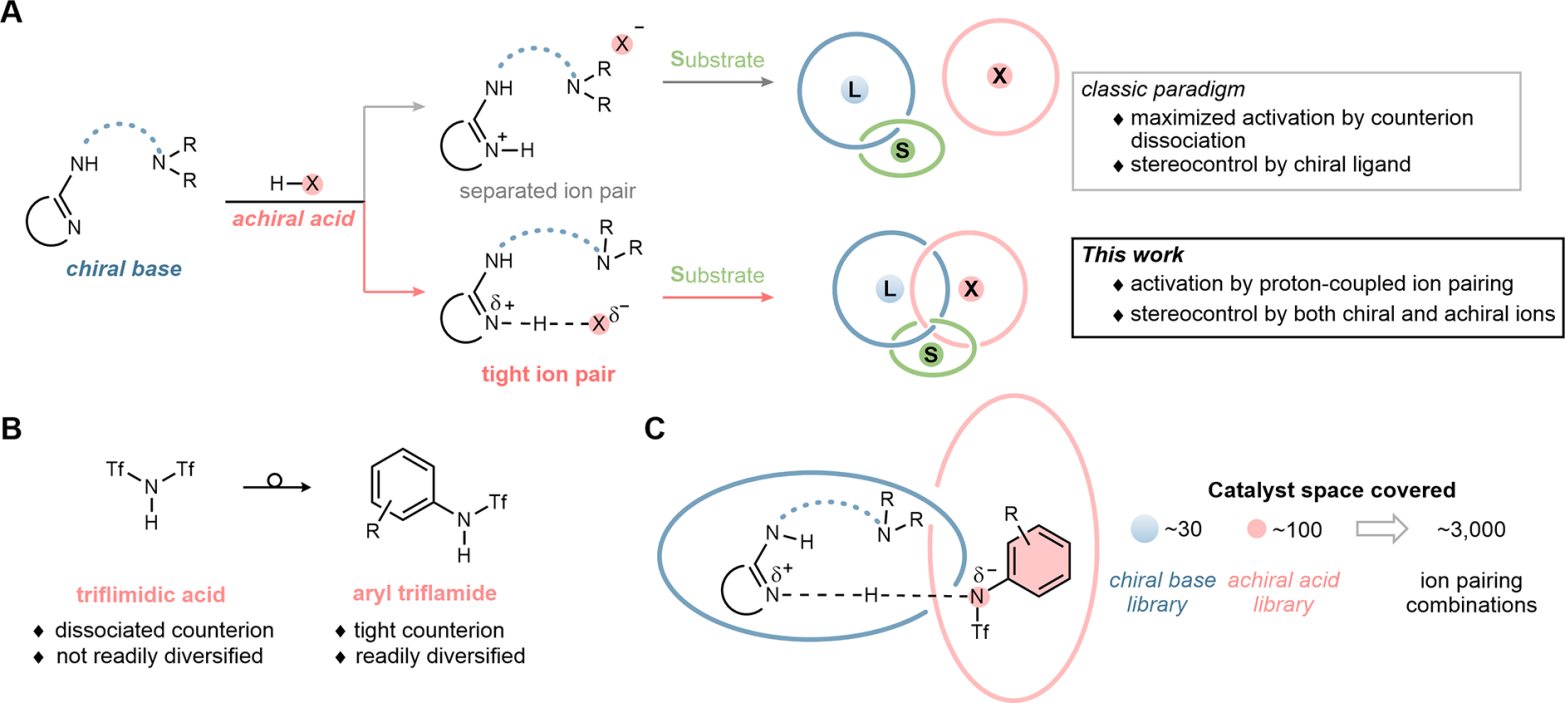
(A) Description of Ion Pair Catalysts, (B) Attributes of Triflamide-Derived Brønsted Acids, and (C) Use of an *N*-Aryl Trifluoromethane Sulfonamide Design for Activation and Topological Control in Catalyst Formation

Enantioselective examples of azide addition to nitroalkenes are exceptionally rare. This highlights the challenge in controlling linear and ambident nucleophiles in enantioselective catalytic reaction development. Enantioselectivity in reactions of an azide nucleophile with aziridines,^9^ enoyl,^10,11^ or enone^12^ electrophiles^13^ has evolved steadily, but nitroalkenes have been notably recalcitrant substrates.^14^ Initial investigation revealed no selectivity for most ligands screened (Figure S10) except lig_2_·HNTf_2_, which exhibited minimal selectivity (15% ee). We prepared a library of 102 unique aryl triflamides (see Figures S1 and S2), but it was more practical in the early stages to use an informer subset in order to explore other contributing variables. Thus, lig_2_ was combined with 23 achiral acids selected from the library with three different solvents: toluene, 1,2-dichloroethane, and heptane.

To quantify the impact of the counterion on enantioselectivity, ee was expressed as apparent ΔΔ*G* ^‡^, and then ΔΔ*G* ^‡^_ArNHTf_ was compared to ΔΔ*G* ^‡^_TfNHTf_ in each case and tabulated as ΔΔΔ*G* ^‡^.^15^ Since triflimide is generally regarded as a weakly coordinating anion, the result from a ligand–triflimidic acid salt catalyst was used to approximate the behavior of the protonated ligand with a dissociated counteranion. Comparison of ΔΔΔ*G* ^‡^ provides a convenient quantification of relative selectivity-based performance. The results are shown in Scheme 3. There was no selectivity in heptane in most cases, presumably due to the insolubility of phthalic acid, which is critical for the generation of active hydrazoic acid (HN_3_). Interestingly, moderate selectivity was achieved in several cases in toluene and DCE. Aryl triflamide I8 delivered the highest selectivity in toluene (79% ee); thus a full evaluation of the aryl triflamide library followed. Most of the aryl triflamides gave low enantioselectivity, but the few exceptions appeared to share a common structure: 1,3-bistriflamides with a substituent in the 5-position.

**Scheme 3:**
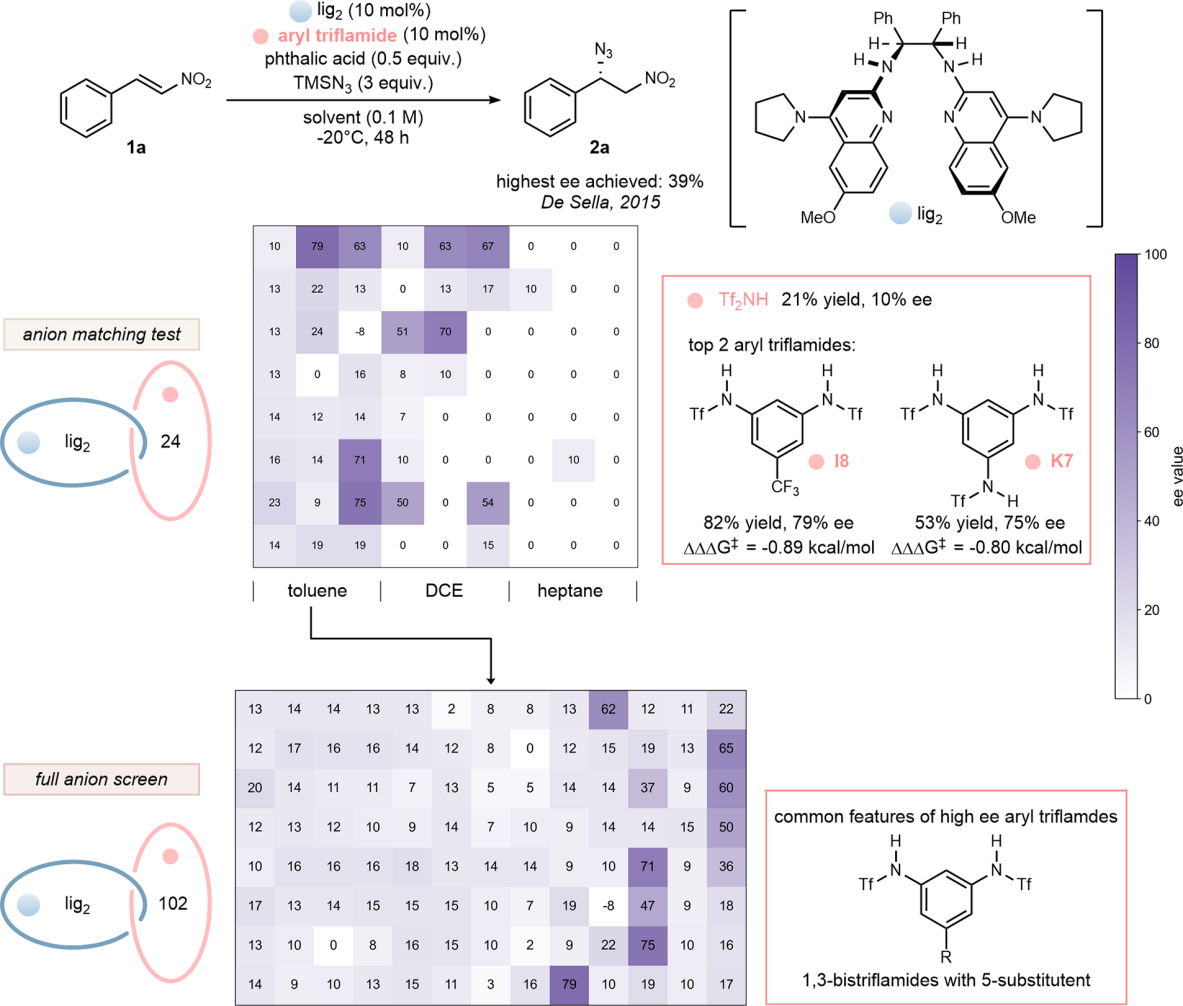
Development of Enantioselective Azide Addition to Nitroalkene with Diverse *N*-Aryl Triflamides as Performance-Enhancing Achiral Catalysts

The effectiveness of the lig_2_·I8 catalyst was then examined with different substrates (Scheme 4). As a control, the Tf_2_NH salt was minimally reactive in most cases and exhibited poor selectivity, even when product formation could be detected. In contrast, lig_2_·I8 yielded satisfying results for most substrates. Addition to *ortho*-substituted nitroalkenes, such as **2b**, **2j**, and **2m**, was generally less selective. Alkyl substrate **2r** was also produced with a lower selectivity. The average ΔΔΔ*G* ^‡^ = −0.86 kcal/mol; aryl triflamide I8 transformed a ligand with unpromising initial behavior to the most effective catalyst for enantioselective azide–nitroalkene addition to date. To further explore the potential of tuning chiral catalysts with achiral counterions, we examined substrates that initially gave low to moderate ee values. Notably, aryl triflamides M3 and M4 delivered higher enantioselectivities than I8 for substrates **2m** and **2o**, respectively. These preliminary results suggest a promising pathway for achieving higher enantioselectivity through strategic modification of aryl triflimides (see Figure S13 for details).

**Scheme 4:**
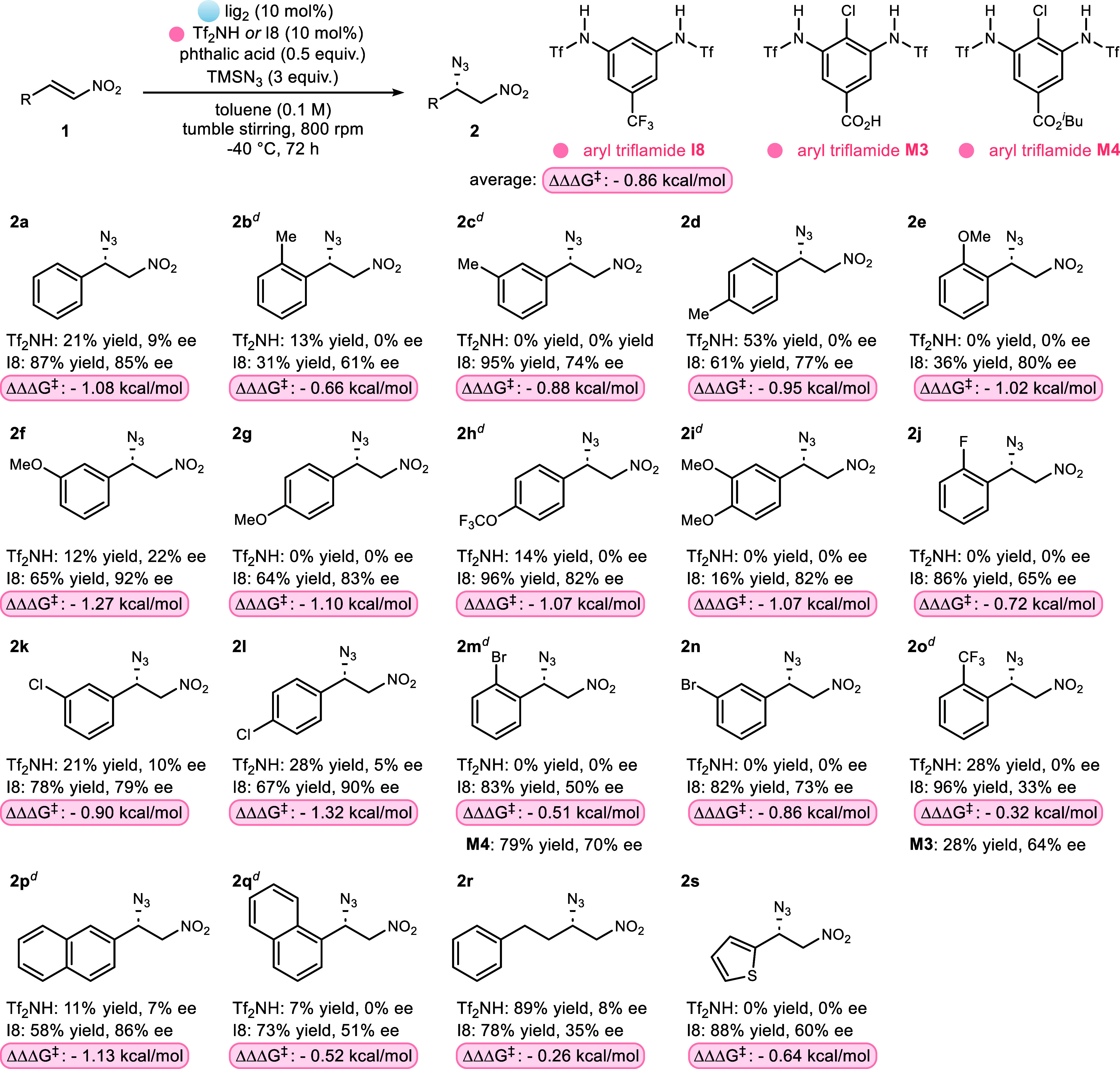
Direct Comparison of lig_2_ with Tf_2_NH or Aryl Triflamide I8 with Different Nitroalkene Substrates in Azide Addition

a General experimental details: the nitroalkene (20 μmol), lig_2_ (10 mol %), and aryl triflamide (10 mol %) were prepared in stock solution and added to a 1 mL glass insert. The solvent was evaporated, and phthalic acid (0.5 equiv) was added. The reaction was then cooled to −78 °C, and TMSN_3_ (3.0 equiv) stock solution in toluene was added and stirred at −40 °C for 72 h prior to workup and analysis; see Supporting Information for complete details.
b Measured by HPLC using a chiral stationary phase.
c Yields measured by ^1^H NMR using internal standard.
d Reaction runs at 100 μmol scale and yields of isolated products are reported here.

A variety of experiments were conducted to probe the mechanistic hypothesis that was originally advanced, centering on the speciation of the active catalyst. A key assumption is the formation of a complex between the ligand and sulfonamide. An interesting feature of the sulfonamide functionality is its potential to function in both single- and two-point binding as a ligand for the amidinium ion. The ^1^H NMR (DMSO-*d*_6_) of the 1:1 ligand:sulfonamide complex of lig_2_·I8 reveals a shift upfield for the sulfonamide aryl proton from 7.38 to 6.78 ppm (Scheme 5A; also see Figure S5). NMR spectroscopy was also used to measure the aryl sulfonamide p*K*_a_ by titration of I8 with DBU, leading to the determination that p*K*_a__1_ ≈ p*K*_a__2_ = 3.1 (DMSO). This acidity pairs well with that for bis­(amidine) ligands (p*K*_a_ = 5.78, DMSO).^16^ The speciation of the complex was also examined by a 2D DOSY experiment, with a single species observed and a corresponding mass = 1198 (estimated using the SEGWE method;^18^ see Figure S6) that identified the 1:1 lig_2_·I8 as the major species (8% difference) in CDCl_3_. Due to the limited solubility of lig_2_·I8 in CDCl_3_,^19^ we employed ligNMe_2_ ^20^ paired with I8 for rotating-frame Overhauser effect spectroscopy (ROESY) NMR experiments, which demonstrated close spatial proximity between I8 and multiple protons on ligNMe_2_ (Scheme 5A; also see Figures S7 and S8). Unfortunately, cocrystallization of lig_2_·I8 led to the consistent formation of a noncrystalline substance.

**Scheme 5:**
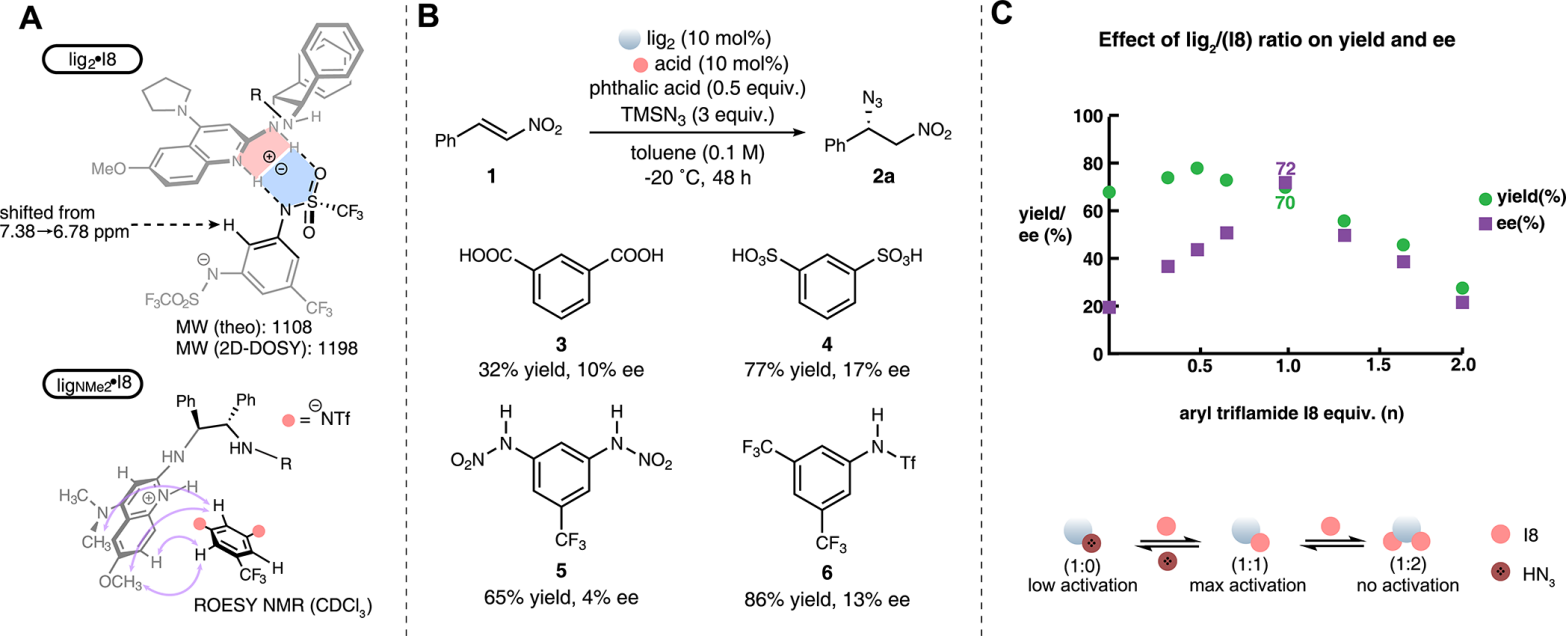
(A) Possible Ligand–Sulfonamide Complementarity and NMR Observations for lig_2_·I8 and a lig_2_ Derivative ligNMe_2_ Forming a Tight Ion Pair with Aryl Triflamide I8; (B) Evaluation of Hydrogen Bond Donors Analogous to the Aryl 1,3-Bis­(triflamide); (C) Aryl Triflamide Equivalence Study

Several organic acids (**3**–**5**) structurally similar to I8 were evaluated as replacements in the azide addition reaction (Scheme 5B). These formed poorly selective (<17% ee) catalysts when combined with lig_2_. Similarly, the *meta*-trifluoromethyl-substituted sulfonamide **6** gave a product with 13% ee, suggesting the importance of a double hydrogen bond donor, which could help to overcome the directionless nature of typical ion pairing catalysis.^21^

While a 1:1 complex of ligand with aryl triflamide is possible by stoichiometry, activity and selectivity can result from the confluence of many species acting proportionately to their concentration. The correlation of lig·I8 stoichiometry with selectivity and yield is summarized for the azide addition reaction in Scheme 5C. Consistent with our hypothesis, both the yield and ee were highest in combinations up to 1:1 and then decreased beyond the midpoint. A possible explanation for these behaviors follows from the simplified equilibrium in Scheme 5C. In the case of each ligand-only reaction, the pronucleophile can function as an acid, forming a poorly selective catalyst. Increasing the amount of sulfonamide increases the concentration of the 1:1 ligand:sulfonamide catalyst. It is for this reason that sulfonamide amounts beyond 1:1 increase the concentration of inactive species (catalyst poisoning).

Finally, DFT calculations were conducted^22^ to understand the structural basis for the enhanced performance of aryl triflamide I8. The calculated energy barriers align well with experimental observations (ΔΔ*G* ^‡^_calc_ = 1.6 kcal/mol vs ΔΔ*G* ^‡^_expt_ = 1.1 kcal/mol for I8), revealing that the reaction barrier is significantly lowered compared to both the uncatalyzed pathway and when using Tf_2_NH. The minimized structures suggest that preorganization is achieved through a hydrogen bonding network where one sulfonamide anchors the acid–base complex while the other activates the hydrazoic acid.

Further inspection of **TS1** for lig_2_/I8 and lig_2_/I4 revealed an unanticipated mechanistic change resulting from the shorter sulfonamide distance. Hydrogen bonding between I4 and hydrazoic acid is made possible by the extended reach of the oxygen atom, and the sizable trifluorosulfonyl group causes the nitroalkene to twist in its hydrogen bonding with the ligand amidinium. The increase in intra-nitrogen distance in I8 allows for improved hydrogen bonding between nitroalkene and amidinum. The aryl triflamide is also involved in proton shuttling from hydrazoic acid to the addition intermediate (**INT1**), which might attenuate the second transition step (**TS1**) energy (proton transfer) to yield the product. Analysis of noncovalent interactions^23^ (see SI, Scheme 6, expanded) revealed extensive π–π interaction evident between the aryl triflamide and quinoline, while the 5-trifluoromethyl group of I8 shows significant F···H interaction with a methoxy hydrogen, perhaps contributing to performance enhancement.

**Scheme 6:**
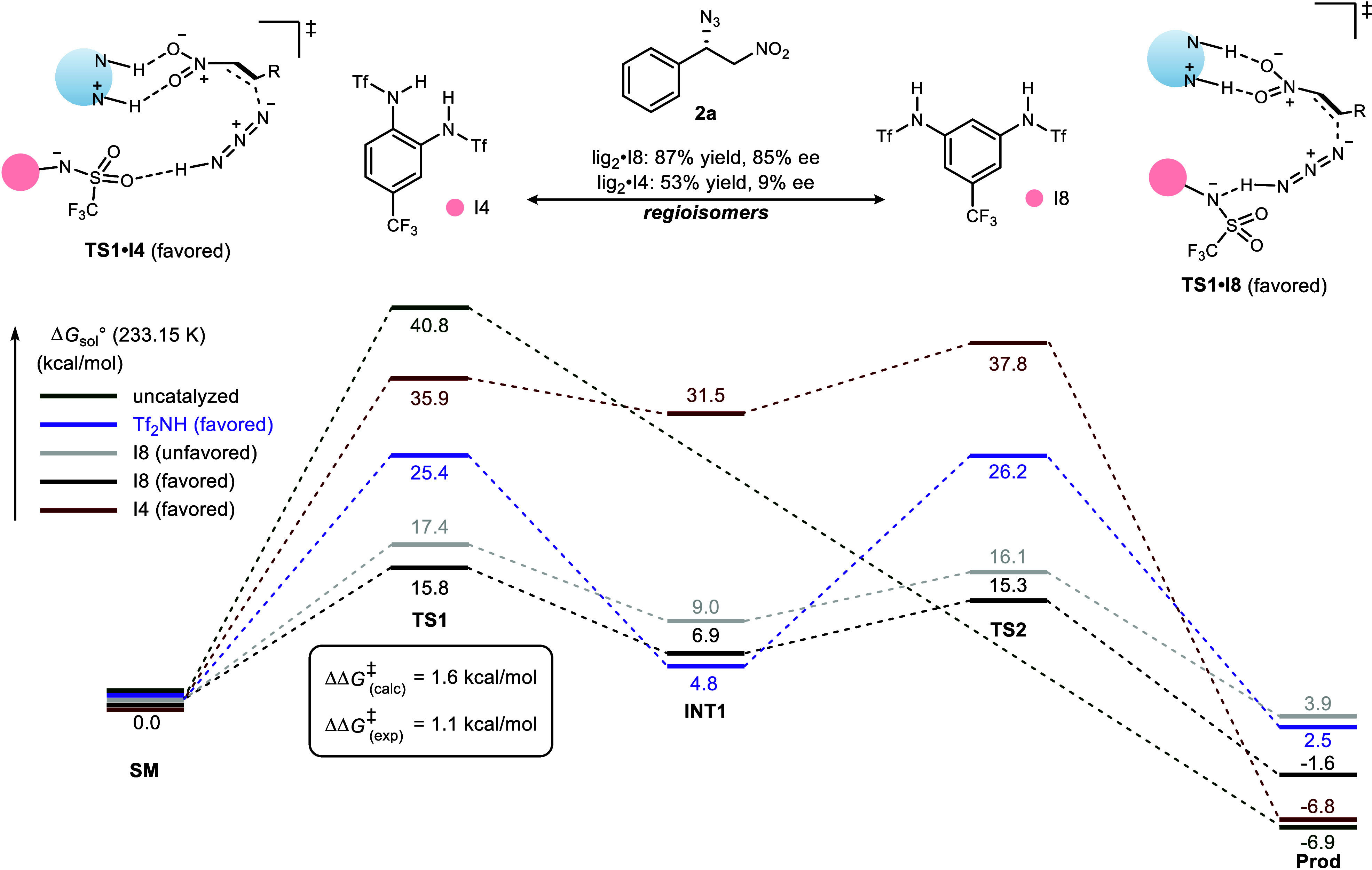
Outlines of Reaction Pathways Using I4, I8, Tf_2_NH, or No Co-catalyst^a^

a All structures were optimized at the r2scan-3c level^17^ method, and all single point energies were calculated using the wB97M-D4/def2-TZVPP level of theory. See SI for noncovalent interaction plot of favored transition state with optimal aryl triflamide I8 illustrating interactions between Iig2 and I8.

Comparison of different ligand–sulfonamide complexes revealed that I8 brings the arms of the chiral ligand into an optimal conformation for substrate binding, while I4 induces a more extreme conformational change that leads to a less favorable transition state (Scheme 7). A strong correlation was found between DFT-derived geometric descriptors (pocket size and geometry) and experimental enantioselectivity (*R* ^2^ = 0.98), supporting our hypothesis that aryl triflamides tune the ligand conformation to enhance catalyst performance (see SI, Scheme 7, expanded).

**Scheme 7:**
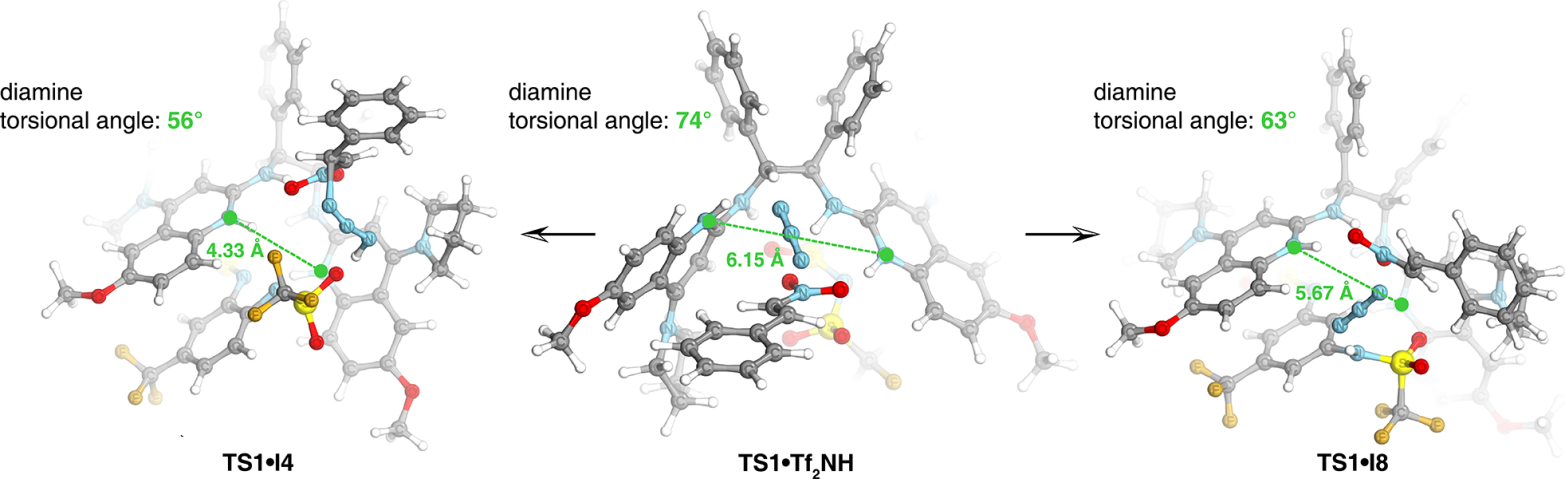
Catalyst Topology Perturbation Analysis When Using Different Aryl Triflamides^a^

a See SI for overlay of lig_2_ geometry when binding with different aryl triflamides and DFT-determined geometric descriptors and their correlation with enantioselectivity.

In conclusion, we have developed a counterion-centric catalyst design predicated on the hypothesis that the performance of a single chiral ligand can be enhanced by a diverse collection of Brønsted acids. Enantioselectivity can be optimized using readily prepared acids, despite their achiral nature and potential to behave as dissociated counteranions. This effect enhances the practical potential of Brønsted acid catalysis by shifting the development workflow away from synthesis-intensive ligand modifications to the more straightforward, modular changes offered by *N*-aryl trifluorosulfonamides. The challenges in *de novo* design and prediction of complex speciation equilibria of this type highlight the advantage of PEAC and its amenability to HTE techniques. This success serves as a proof-of-principle that achiral counterions may be a potential design element to enhance the performance of chiral ligands that underperform in prospecting studies for asymmetric synthesis.

## Supplementary Material





## Notes

Complete experimental details (PDF)
Spectroscopic data (PDF)
