# Supplementary material for: Performance-Enhancing Asymmetric Catalysis Driven by Achiral Counterion Design
Source: J Am Chem Soc. 2025 May 14;147(21):17584–91. doi: 10.1021/jacs.5c05263 (PMC12123621; doi:10.1021/jacs.5c05263)
Supplement: Supplementary file 1 [file ja5c05263_si_001.pdf]

Performance Enhancing Asymmetric Catalysis Driven by Achiral Counterion Design

Zihang Deng, Jenna L. Payne, Mahesh Vishe, Julius E. L. Jan, Cody M. Funk, Jeffrey N. Johnston*
Department of Chemistry and Vanderbilt Institute of Chemical Biology,
Vanderbilt University, Nashville, Tennessee 37235

Table of Contents

Expanded Manuscript Schemes 7
Scheme 6 (expanded) 7
Scheme 7 (expanded) 8
General Experimental Procedures 9
General Experimental Procedures for High-Throughput Experimentation 9
Reproducibility of HTE experiment results 11
Aryl Triflamide Library Index 11
NMR titration for determination of the pKa for aryl triflamide I8 in DMSO 14
NMR titration for determination of the pKa for aryl triflamide I4 in DMSO 15
Acid Equivalence Study for $Lig_2 \cdot n(I8)$ 16
NMR Evidence for the formation of $lig_2 \cdot I8$ Ion Pair 17
2D-DOSY Experiment 18
1D-ROESY Experiment 19
Study of Proton Source on the Reaction Outcome 21
Catalyst Development for Azide Addition 22
Initial evaluation 23
Anion match test 23
Full anion screening 23
Aryl triflamide matching screening for challenging substrates 25
DFT Calculation 26
Methods 26
Noncovalent interactions plotting 29
Cartesian coordinates of optimized geometries of reaction pathways 30
Uncatalyzed hydrozic acid and nitroalkene (SM) 30
Uncatalyzed TS (TS) 31
Uncatalyzed product (Prod) 32
Iig_2/I8 with hydrozic acid and nitroalkene (SM) 33
Iig_2/I8 TS1 (favored enantiomer) 38
Iig_2/I8 TS1 (unfavored enantiomer) 43

Iig$_2$/I8 intermediate (favored enantiomer) 48
Iig$_2$/I8 intermediate (unfavored enantiomer) 53
Iig$_2$/I8 TS2 (favored enantiomer) 57
Iig$_2$/I8 TS2 (unfavored enantiomer) 62
Iig$_2$/I8 product (favored enantiomer) 67
Iig$_2$/I8 product (unfavored enantiomer) 72
Iig$_2$/I4 with hydrazoic acid and nitroalkene 77
Iig$_2$/I4 TS1 (favored enantiomer) 82
Iig$_2$/I4 intermediate (favored enantiomer) 87
Iig$_2$/I4 TS2 (favored enantiomer) 92
Iig$_2$/I4 product (favored enantiomer) 97
Iig$_2$/Tf$_2$NH with hydrozic acid and nitroalkene 102
Iig$_2$/Tf$_2$NH TS1 (favored enantiomer) 107
Iig$_2$/Tf$_2$NH intermediate (favored enantiomer) 111
Iig$_2$/Tf$_2$NH TS2 (favored enantiomer) 116
Iig$_2$/Tf$_2$NH product (favored enantiomer) 120
Cartesian coordinates of optimized geometries of ligand-aryl triflamide pairs 125
Coordinates of optimized geometry of ligNMe$_2$·I8 125
Coordinates of optimized geometry of lig$_2$·I3 129
Coordinates of optimized geometry of lig$_2$·I4 133
Coordinates of optimized geometry of lig$_2$·I8 137
Coordinates of optimized geometry of lig$_2$·J1 141
Coordinates of optimized geometry of lig$_2$·K5 146
Coordinates of optimized geometry of lig$_2$·K7 150
Catalyst Synthesis 155
(1*R*,2*R*)-*N*^{1}-(4-(Pyrrolidin-1-yl)quinolin-2-yl)cyclohexane-1,2-diamine (S1). 155
(1*R*,2*R*)-*N*^{1},*N*^{1}-Didodecyl-*N*^{2}-(4-(pyrrolidin-1-yl)quinolin-2-yl)cyclohexane-1,2-diamine (lig$_7$). 156
(1*R*,2*R*)-*N*1-(7-Methoxy-4-(pyrrolidin-1-yl)quinolin-2-yl)-*N*^{2},*N*^{2}-dioctadecylcyclohexane-1,2-diamine (lig$_8$). 156
General Procedure 1 for Preparation of *N*-Aryl Triflamides 157
1,1,1-Trifluoro-*N*-phenylmethanesulfonamide (A1) 157
N-(2-Ethylphenyl)-1,1,1-trifluoromethanesulfonamide (A2) 157
N-(3-(*tert*-Butyl)phenyl)-1,1,1-trifluoromethanesulfonamide (A3) 157
N-(4-(*tert*-Butyl)phenyl)-1,1,1-trifluoromethanesulfonamide (A4) 157
N-(2,3-Dimethylphenyl)-1,1,1-trifluoromethanesulfonamide (A5) 157

N-(2,6-Dimethylphenyl)-1,1,1-trifluoromethanesulfonamide (A6) 158
N-(3,5-Dimethylphenyl)-1,1,1-trifluoromethanesulfonamide (A7) 158
N-(2,6-Diisopropylphenyl)-1,1,1-trifluoromethanesulfonamide (A8) 158
1,1,1-Trifluoro-*N*-(2-methoxyphenyl)methanesulfonamide (B1) 158
1,1,1-Trifluoro-*N*-(3-methoxyphenyl)methanesulfonamide (B2) 158
1,1,1-Trifluoro-*N*-(4-methoxyphenyl)methanesulfonamide (B3) 158
1,1,1-Trifluoro-*N*-(2,4-dimethoxyphenyl)methanesulfonamide (B4) 158
1,1,1-Trifluoro-*N*-(3-(methylthio)phenyl)methanesulfonamide (B5) 158
1,1,1-Trifluoro-*N*-(2-nitrophenyl)methanesulfonamide (B6) 159
1,1,1-Trifluoro-*N*-(3-nitrophenyl)methanesulfonamide (B7) 159
1,1,1-Trifluoro-*N*-(4-nitrophenyl)methanesulfonamide (B8) 159
N-(2-Acetylphenyl)-1,1,1-trifluoromethanesulfonamide (C1) 159
N-(2-Acetylphenyl)-1,1,1-trifluoromethanesulfonamide (C2) 159
1,1,1-Trifluoro-*N*-(4-(trifluoromethyl)phenyl)methanesulfonamide (C3) 159
1,1,1-Trifluoro-*N*-(4-fluorophenyl)methanesulfonamide (C4) 159
1,1,1-Trifluoro-*N*-(2,4,6-trifluorophenyl)methanesulfonamide (C5) 159
1,1,1-Trifluoro-*N*-(2,3,5,6-tetrafluorophenyl)methanesulfonamide (C6) 160
1,1,1-Trifluoro-*N*-(perfluorophenyl)methanesulfonamide (C7) 160
N-(2-Chlorophenyl)-1,1,1-trifluoromethanesulfonamide (C8) 160
N-(4-Bromophenyl)-1,1,1-trifluoromethanesulfonamide (D1) 160
1,1,1-Trifluoro-*N*-(2-iodophenyl)methanesulfonamide (D2) 160
1,1,1-Trifluoro-*N*-(4-iodophenyl)methanesulfonamide (D3) 160
N-(4-Chloro-3-nitrophenyl)-1,1,1-trifluoromethanesulfonamide (D4) 160
1,1,1-Trifluoro-*N*-(2-methoxy-4-nitrophenyl)methanesulfonamide (D5) 160
1,1,1-Trifluoro-*N*-(4-methoxy-2-nitrophenyl)methanesulfonamide (D6) 161
3-((Trifluoromethyl)sulfonamido)phenyl trifluoromethanesulfonate (D7) 161
N-(2-Chloro-4-(trifluoromethyl)phenyl)-1,1,1-trifluoromethanesulfonamide (D8) 161
N-(2-Cyano-6-fluorophenyl)-1,1,1-trifluoromethanesulfonamide (E1) 161
5-Fluoro-2-((trifluoromethyl)sulfonamido)benzoic acid (E2) 161
N-(3-Chloro-5-(trifluoromethyl)phenyl)-1,1,1-trifluoromethanesulfonamide (E3) 162
N-(3-(Difluoromethyl)phenyl)-1,1,1-trifluoromethanesulfonamide (E4) 162
1,1,1-Trifluoro-*N*-(4-(4,4,5,5-tetramethyl-1,3,2-dioxaborolan-2-yl)phenyl)methanesulfonamide (E5) 162
1,1,1-Trifluoro-*N*-(4-(trifluoromethyl)pyridin-2-yl)methanesulfonamide (E6) 162
Methyl 3-hydroxy-4-((trifluoromethyl)sulfonamido)benzoate (E7) 162
Methyl 4-((trifluoromethyl)sulfonamido)-3-(((trifluoromethyl)sulfonyl)oxy)benzoate (E8) 162

1,1,1-Trifluoro-*N*-(4-(trifluoromethyl)benzyl)methanesulfonamide (F1) 163
N-(2,4-Dimethoxybenzyl)-1,1,1-trifluoromethanesulfonamide (F2) 163
1,1,1-Trifluoro-*N*-(pyridin-2-ylmethyl)methanesulfonamide (F3) 163
N-((1S,3S)-Adamantan-1-yl)-1,1,1-trifluoromethanesulfonamide (F4) 163
N-Cyclohexyl-1,1,1-trifluoromethanesulfonamide (F5) 163
1,1,1-Trifluoro-*N*-(4-methylpyrimidin-2-yl)methanesulfonamide (F6) 163
1,1,1-Trifluoro-*N*-(6-fluoropyridin-2-yl)methanesulfonamide (F7) 163
1,1,1-Trifluoro-*N*-(3-fluoropyridin-2-yl)methanesulfonamide (F8) 163
N,*N*'-(Pyridine-2,6-diyl)bis(1,1,1-trifluoromethanesulfonamide) (G1) 164
N-(4,5-Dichloropyridin-2-yl)-1,1,1-trifluoromethanesulfonamide (G2) 164
1,1,1-Trifluoro-*N*-(6-phenylpyridin-2-yl)methanesulfonamide (G3) 164
N-(3-Cyanopyridin-2-yl)-1,1,1-trifluoromethanesulfonamide (G4) 164
1,1,1-Trifluoro-*N*-(5-nitrothiazol-2-yl)methanesulfonamide (G5) 164
1,1,1-Trifluoro-*N*-(5-methylisoxazol-3-yl)methanesulfonamide (G6) 164
Ethyl 2-((trifluoromethyl)sulfonamido)-5,6,7,8-tetrahydro-4H-cyclohepta[b]thiophene-3-carboxylate (G7) 165
1,1,1-Trifluoro-*N*-(4-phenylthiazol-2-yl)methanesulfonamide (G8) 165
1,1,1-Trifluoro-*N*-(4-methylthiazol-2-yl)methanesulfonamide (H1) 165
1,1,1-Trifluoro-*N*-(1,3,4-thiadiazol-2-yl)methanesulfonamide (H2) 165
1,1,1-Trifluoro-*N*-(3-oxo-3,4-dihydro-2H-benzo[b][1,4]oxazin-7-yl)methanesulfonamide (H3) 165
N-(2,3-Dihydrobenzofuran-7-yl)-1,1,1-trifluoromethanesulfonamide (H4) 165
1,1,1-Trifluoro-*N*-(4-methylpyridin-2-yl)methanesulfonamide (H5) 166
1,1,1-Trifluoro-*N*-(quinolin-2-yl)methanesulfonamide (H6) 166
1,1,1-Trifluoro-*N*-(quinoxalin-6-yl)methanesulfonamide (H7) 166
1,1,1-Trifluoro-*N*-(isoquinolin-3-yl)methanesulfonamide (H8) 166
1,1,1-Trifluoro-*N*-(naphthalen-2-yl)methanesulfonamide (I1) 166
N,*N*'-(1,2-Phenylene)bis(1,1,1-trifluoromethanesulfonamide) (I2) 166
N,*N*'-(1,4-Phenylene)bis(1,1,1-trifluoromethanesulfonamide) (I3) 166
N,*N*'-(4-(Trifluoromethyl)-1,2-phenylene)bis(1,1,1-trifluoromethanesulfonamide) (I4) 167
N,*N*'-(Pyridine-2,3-diyl)bis(1,1,1-trifluoromethanesulfonamide) (I6) 167
N,*N*'-(5-(Trifluoromethyl)-1,3-phenylene)bis(1,1,1-trifluoromethanesulfonamide) (I8) 167
N,*N*'-(5-(Perfluoroethyl)-1,3-phenylene)bis(1,1,1-trifluoromethanesulfonamide) (J1) 167
N,*N*'-(Naphthalene-1,5-diyl)bis(1,1,1-trifluoromethanesulfonamide) (J2) 167
N-([1,1'-Biphenyl]-4-yl)-1,1,1-trifluoromethanesulfonamide (J3) 167
N,*N*'-([1,1'-Biphenyl]-2,2'-diyl)bis(1,1,1-trifluoromethanesulfonamide) (J4) 168

1,1,1-Trifluoro-*N*-(3-(trifluoromethoxy)phenyl)methanesulfonamide (J5) 168
N,*N*'-(Oxybis(3,1-phenylene))bis(1,1,1-trifluoromethanesulfonamide) (J6) 168
N,*N*'-(Oxybis(2,1-phenylene))bis(1,1,1-trifluoromethanesulfonamide) (J7) 168
N,*N*'-(Methylenebis(3,1-phenylene))bis(1,1,1-trifluoromethanesulfonamide) (J8) 168
N,*N*'-(Anthracene-1,8-diyl)bis(1,1,1-trifluoromethanesulfonamide) (K1) 168
N,*N*'-(2,2",4,4"-Tetrafluoro-[1,1':3',1"-terphenyl]-4',6'-diyl)bis(1,1,1-trifluoromethanesulfonamide) (K2) 169
N,*N*'-(4,6-Di(naphthalen-1-yl)-1,3-phenylene)bis(1,1,1-trifluoromethanesulfonamide) (K3) 169
N,*N*'-((9H-fluorene-9,9-diyl)bis(4,1-phenylene))bis(1,1,1-trifluoromethanesulfonamide) (K4) 169
N,*N*'-(3',5'-bis(trifluoromethyl)-[1,1'-biphenyl]-3,5-diyl)bis(1,1,1-trifluoromethanesulfonamide) (K5) 169
N,*N*'-(2',4'-Difluoro-[1,1'-biphenyl]-3,5-diyl)bis(1,1,1-trifluoromethanesulfonamide) (K6) 170
N,*N*',*N*"-(Benzene-1,3,5-triyl)tris(1,1,1-trifluoromethanesulfonamide) (K7) 170
5-(Trifluoromethoxy)-2-(5-(trifluoromethoxy)-2-((trifluoromethyl)sulfonamido)benzamido)benzoic acid (K8) 170
1,1,1-Trifluoro-*N*-(naphthalen-1-yl)methanesulfonamide (L1) 170
1,1,1-Trifluoro-*N*-(3-phenoxyphenyl)methanesulfonamide (L2) 170
N-(Benzo[d]thiazol-6-yl)-1,1,1-trifluoromethanesulfonamide (L3) 170
N-(5-(Benzyloxy)pyridin-2-yl)-1,1,1-trifluoromethanesulfonamide (L4) 171
tert-Butyl (4-((trifluoromethyl)sulfonamido)benzyl)carbamate (L5) 171
N-(2-Benzoylphenyl)-1,1,1-trifluoromethanesulfonamide (L6) 171
N-(1H-Benzo[d]imidazol-6-yl)-1,1,1-trifluoromethanesulfonamide (L7) 171
N,*N*'-(9,10-Dioxo-4a,9,9a,10-tetrahydroanthracene-2,6-diyl)bis(1,1,1-trifluoromethanesulfonamide) (L8) 171
N,*N'*-(2,4,6-Trimethyl-1,3-phenylene)bis(1,1,1-trifluoromethanesulfonamide) (M1) 171
1,1,1-Trifluoro-*N*-((trifluoromethyl)sulfonyl)-*N*-(2,4,6-trimethyl-3-((trifluoromethyl)sulfonamido)phenyl)methanesulfonamide (M2) 172
4-Chloro-3,5-bis((trifluoromethyl)sulfonamido)benzoic acid (M3) 172
Isobutyl 4-chloro-3,5-bis((trifluoromethyl)sulfonamido)benzoate (M4) 172
N,*N'*-(2-Nitro-1,4-phenylene)bis(1,1,1-trifluoromethanesulfonamide) (M5) 172
(Bis((trifluoromethyl)sulfonyl)methyl)benzene (M6) 172
1-(Bis((trifluoromethyl)sulfonyl)methyl)-2-(((trifluoromethyl)sulfonyl)methyl)benzene (M7) 172
1-(Bis((trifluoromethyl)sulfonyl)methyl)-3-(((trifluoromethyl)sulfonyl)methyl)benzene (M8) 173
General Procedure 2 for Enantioselective Azide Addition to Nitroalkenes 173
(*S*)-(1-Azido-2-nitroethyl)benzene (2a). 174
(*S*)-1-(1-Azido-2-nitroethyl)-2-methylbenzene (2b). 174
(*S*)-1-(1-Azido-2-nitroethyl)-3-methylbenzene (2c). 174
(*S*)-1-(1-Azido-2-nitroethyl)-4-methylbenzene (2d). 174

(*S*)-1-(1-Azido-2-nitroethyl)-2-methoxylbenzene (2e). 175
(*S*)-1-(1-Azido-2-nitroethyl)-3-methoxylbenzene (2f). 175
(*S*)-1-(1-Azido-2-nitroethyl)-4-methoxylbenzene (2g). 175
(*S*)-2-Azido-1-nitro-2-(4-(trifluoromethoxy)phenyl)ethan-1-ide (2h). 175
(*S*)-4-(1-Azido-2-nitroethyl)-1,2-dimethoxybenzene (2i). 175
(*S*)-1-(1-Azido-2-nitroethyl)-2-fluorobenzene (2j). 176
(*S*)-1-(1-Azido-2-nitroethyl)-3-chlorobenzene (2k). 176
(*S*)-1-(1-Azido-2-nitroethyl)-4-chlorobenzene (2l). 176
(*S*)-1-(1-Azido-2-nitroethyl)-2-bromobenzene (2m). 176
(*S*)-1-(1-Azido-2-nitroethyl)-3-bromobenzene (2n). 176
(*S*)-1-(1-Azido-2-nitroethyl)-2-(trifluoromethyl)benzene (2o). 177
(*S*)-2-(1-Azido-2-nitroethyl)naphthalene (2p). 177
(*S*)-1-(1-Azido-2-nitroethyl)naphthalene (2q). 177
(*S*)-(3-Azido-4-nitrobutyl)benzene (2r). 177
(*S*)-2-(1-Azido-2-nitroethyl)thiophene (2s). 177

Expanded Manuscript Schemes

Scheme 6 (expanded). A) Outlines of reaction pathways using I4, I8, Tf_2NH, or no co-catalyst. All structures were optimized using the r2scan-3c method, and all single point energies were calculated using wB97M-D4/def2-TZVPP level of theory. B) Noncovalent interaction plot of favored transition state with optimal aryl triflamide I8 illustrating interactions between Iig2 and I8.

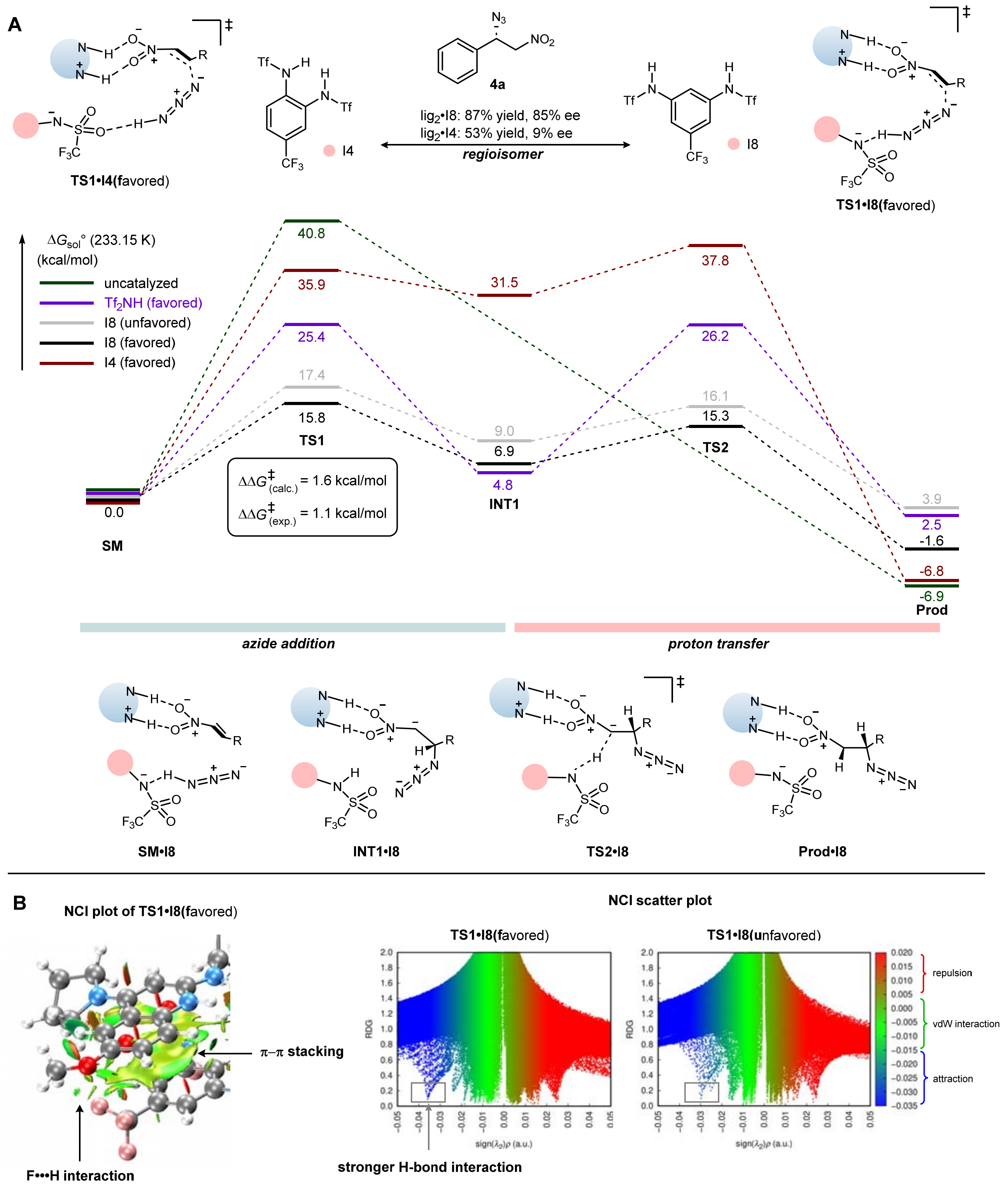

Scheme 7 (expanded). A) Catalyst topology perturbations analysis when using different aryl triflamides B) Overlay of lig_2 geometry when binding with different aryl triflamides (aryl triflamides are hidden so that ligand geometry variation is not obscured). The 'free' lig_2 geometry without aryl triflamide is shown in gold. C) DFT-determined geometric descriptors and their correlation with enantioselectivity.

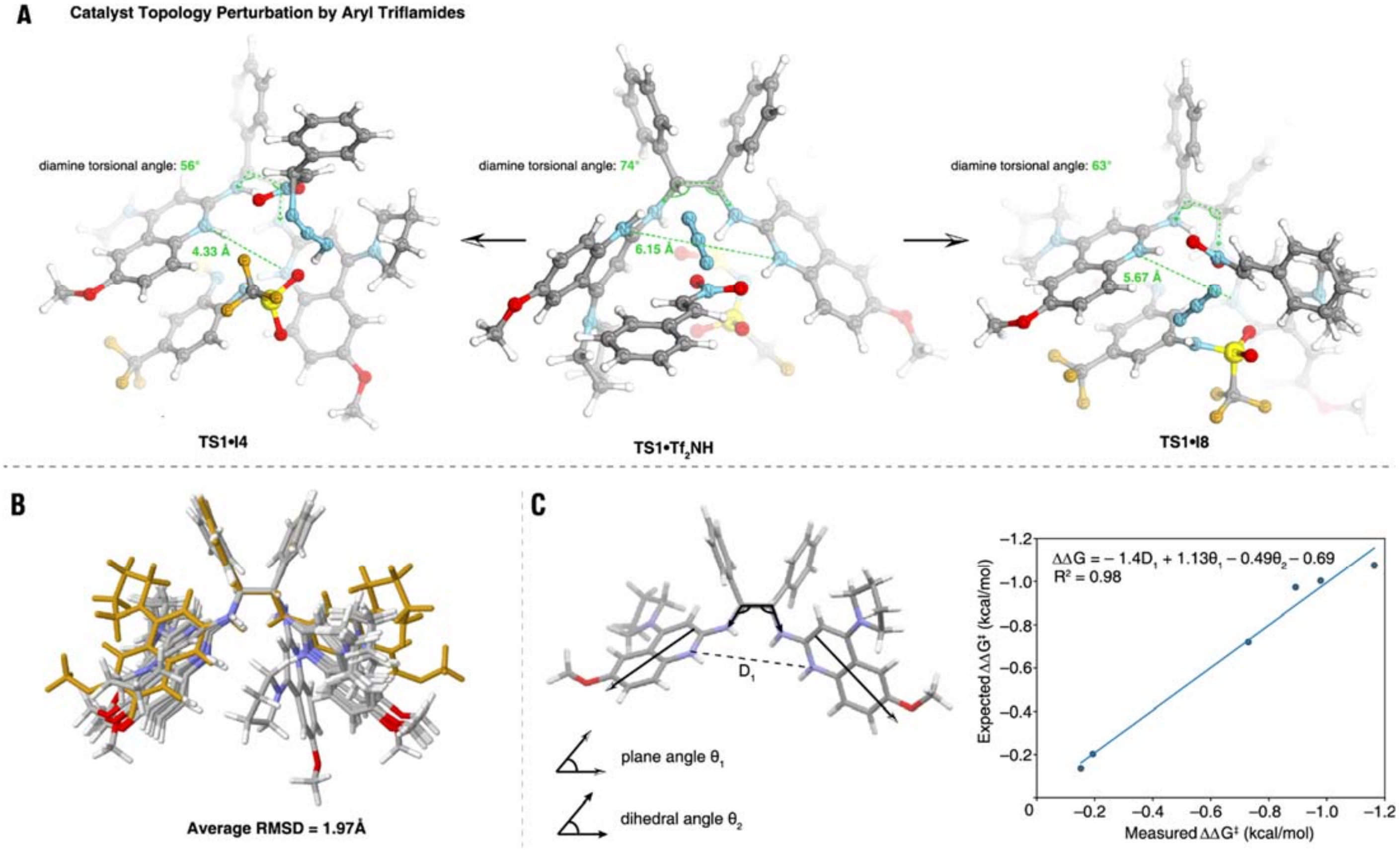

General Experimental Procedures

All reagents and solvents were commercial grade and purified prior to use when necessary. Acetonitrile (MeCN), tetrahydrofuran (THF), dichloromethane (CH_2Cl_2), and toluene (PhMe) were dried by passage through a column of activated alumina as described by Grubbs.[1]

Thin layer chromatography (TLC) was performed using glass-backed silica gel (250 μm) plates, and flash chromatography utilized 230–400 mesh silica gel from Sorbent Technologies. UV light, and/or the use of *para*-anisaldehyde, potassium permanganate, or phosphomolybdic acid solutions were used to visualize products.

Melting points were measured on an SRS Meltemp melting point apparatus and were not corrected. IR spectra were recorded on a Nicolet IR 200 spectrophotometer and are reported in wavenumbers (cm^{-1}) analyzed as neat films on NaCl plates (transmission). Nuclear magnetic resonance spectra (NMR) were obtained on a Bruker DRX-400 (400 MHz) or a Bruker AVIII-600 (600 MHz) spectrometer. Chemical shifts are measured to residual non-deuterated solvent as an internal standard. Mass spectra were recorded on a high resolution Thermo Electron Corporation MAT 95XP-Trap or Thermo Fisher LTQ Orbitrap XL by use of electro-spray ionization (ESI) or atmospheric pressure chemical ionization (APCI) by the Vanderbilt Mass Spectrometry Research Center (MSRC) Cores or the Indiana University Mass Spectrometry Facility. Optical rotations were measured on a Perkin Elmer-341 polarimeter. Chiral HPLC analysis was conducted on an Agilent 1100 series instrument using the designated ChiralPak column, and the racemic material was prepared using an achiral (or racemic) catalyst.

General Experimental Procedures for High-Throughput Experimentation

1) Stock Solution Preparation

   - Reagents are typically added by preparing a stock solution in which they are highly soluble (> 100 μmol/mL) before dispensing into the well plate using either a standard or multichannel pipette.
   - As multiple reagents may require different solvents, it is advisable to select solvents with lower boiling points, such as chloroform, acetone, and methanol, to facilitate easier removal by evaporation.
   - Prepared solutions, especially those containing sensitive chiral bases or aryl triflamides, should be stored in a freezer to extend their shelf life and maintain chemical integrity.

2) Reaction Set-up

   - Reaction blocks made of either polypropylene (PP), or aluminum are used, sourced from Analytical Sales and Services Inc.
   - PP blocks are chosen for their resistance to most common organic solvents and cost-effectiveness, while aluminum blocks are preferred, despite expense, for improved heat conductivity.
   - The choice between PP and aluminum depends on the thermal requirements of the reaction, such as the need for heating or cooling.
   - Glass inserts that fit into the 96-well plate are used to ensure chemical inertness, and are available in sizes ranging from 1 mL to 2 mL. The 1 mL size is sufficient for most scenarios, while 2 mL inserts are primarily used for preparing stock solutions and HPLC samples.
   - Sealing the reaction setup effectively can be challenging, particularly when working with volatile solvents at room temperature. PP blocks are prone to solvent leakage, and commercially available caps often fail to prevent this entirely. Many caps are also made of materials like rubber, which can absorb organic solvents and distort.

- Quantitative tests show that using a typical PP block setup at room temperature overnight can result in solvent losses exceeding 100 μL for low boiling point solvents (e.g., DCM, diethyl ether, THF, acetone), and around 50 μL for higher boiling point solvents like ethyl acetate, DCE, and toluene.

- Aluminum blocks originally designed with a rubber layer can also experience similar solvent losses. However, covering the glass inserts with aluminum foil before adding the rubber layer significantly improves the seal, reducing overnight solvent loss to less than 5 μL with higher boiling point solvents.

- Effective stirring is crucial, especially for heterogeneous reactions, as it promotes turbulence and enhances heat and mass transfer. A tumble stirrer (VP 710C5-7A from V&P Scientific, Inc.) is used, with stir bars for glass inserts sourced from the same company.

- When using an aluminum reaction block, electromagnetic induction from the stirrer can cause the block to heat up to 40-60°C. To mitigate this, the reaction block should be positioned 5-10 inches from the stirrer, and a fan should be used to dissipate heat.

3) Work-up and Purification

- Thermo Scientific™ HyperSep™ Silica Plates from Fisher Scientific are used for fast purification of multiple wells. Each well of the HyperSep Silica Plate is loaded with approximately 25 to 50 mg of silica gel.

- A vacuum manifold system sourced from Analytical Sales and Services Inc. is used to provide the driving force for passing the reaction mixture through the silica.

4) Data Acquisition

- After purification, the reaction mixtures are dissolved in a solvent mixture of hexanes and isopropanol, which is chosen to optimize the dissolution of reaction products while ensuring compatibility with the chiral HPLC columns.

- A pure sample of the product is prepared to obtain a calibration curve on HPLC to obtain both yield and ee values.

- The typical HPLC method is set for a 30-minute run time, which is usually sufficient to achieve good resolution of peaks and accurate quantification of both yield and ee.

- In scenarios where peak separations are substantial (i.e., the retention time between two peaks exceeds five minutes), adopting a more polar HPLC method can significantly reduce the run time to about 15-20 minutes without compromising the separation quality.

- In high-throughput setups, where numerous samples are processed simultaneously, reducing the HPLC run time can lead to substantial increases in overall screening efficiency by allowing more samples to be analyzed within the same timeframe.

5) Data Analysis

- The simplest and most immediate form of analysis involves creating a heatmap to visualize the yield and ee data for each well of the 96-well plate. This visualization technique provides a quick and clear way to identify which conditions produced the best outcomes in terms of yield and ee.

- The heatmap is generated using a Python script, which is available in the code section of the supporting information.

Reproducibility of HTE experiment results

The reproducibility of HTE experiment results was tested by selecting a few experiments carried out under HTE conditions and reproducing them at typical bench conditions on a 100 µmol scale. (Table S1) It was found that error seemed to favor an underestimate of performance: larger scale conditions generally gave better yields, and a few reactions that showed low ee at HTE conditions exhibited higher ee at a larger scale. Based on observations of component solubilities, we hypothesize that the active catalyst in this reaction may exhibit gel-like physicochemical properties leading to lower solubility in toluene. Efficient stirring maximizes the possibility of a homogeneous mixture or solution. In general, HTE results with high ee's (>70%) indicates that the reaction was appropriately activated, and results are highly consistent.

Table S1. Reproducibility of azide addition.

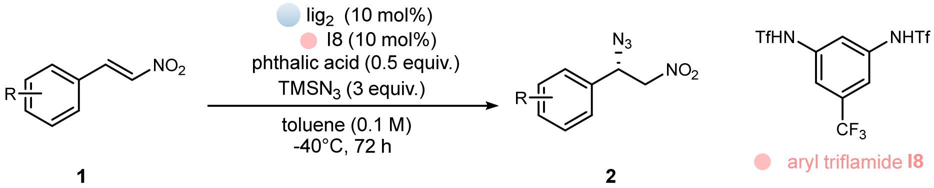

entry	sm/prod	R	5 µmol THE yield (%)[a]	100 µmol Bench yield (%)[b]	yield (%) difference	5 µmol HTE ee (%)	100 µmol Bench ee (%)	ee (%) difference
**1**	a	H	87	95	+8	85	85	0
**2**	c	^{m}Me	69	95	+26	71	74	+3
**3**	h	$^{p}CF_3O$	46	96	+50	63	83	+20
**4**	i	$^{3,4}(MeO)_2$	11	25	+14	82	79	-3
**5**	m	^{o}Br	83	99	+16	50	50	0
**6**	o	$^{o}CF_3$	63	96	+23	20	33	+13
**7**	p	^{2}Naphth	58	81	+23	86	84	-2
**8**	q	^{1}Naphth	73	86	+13	51	46	-5

[a]Yields measured by ^{1}H NMR using internal standard. [b]Reproduced at 100 µmol; Reaction time = 48 h; the separated yields were measured.

Aryl Triflamide Library Index

To manage the naming complexity of aryl triflamides effectively, we have developed a systematic naming convention for this purpose. Each compound is designated by a combination of a capitalized letter followed by an integer, such as 'A1' for the first entry, 'L8' for another, and so forth. This method allows for straightforward identification and reference across various documents and discussions. The complete list of aryl triflamides, along with their corresponding codes, is detailed in Figure S1 and Figure S2. This figure not only serves as a quick reference but also ensures that each aryl triflamide can be easily located and identified within the library.

Figure S1. Aryl triflamide library (1/2).

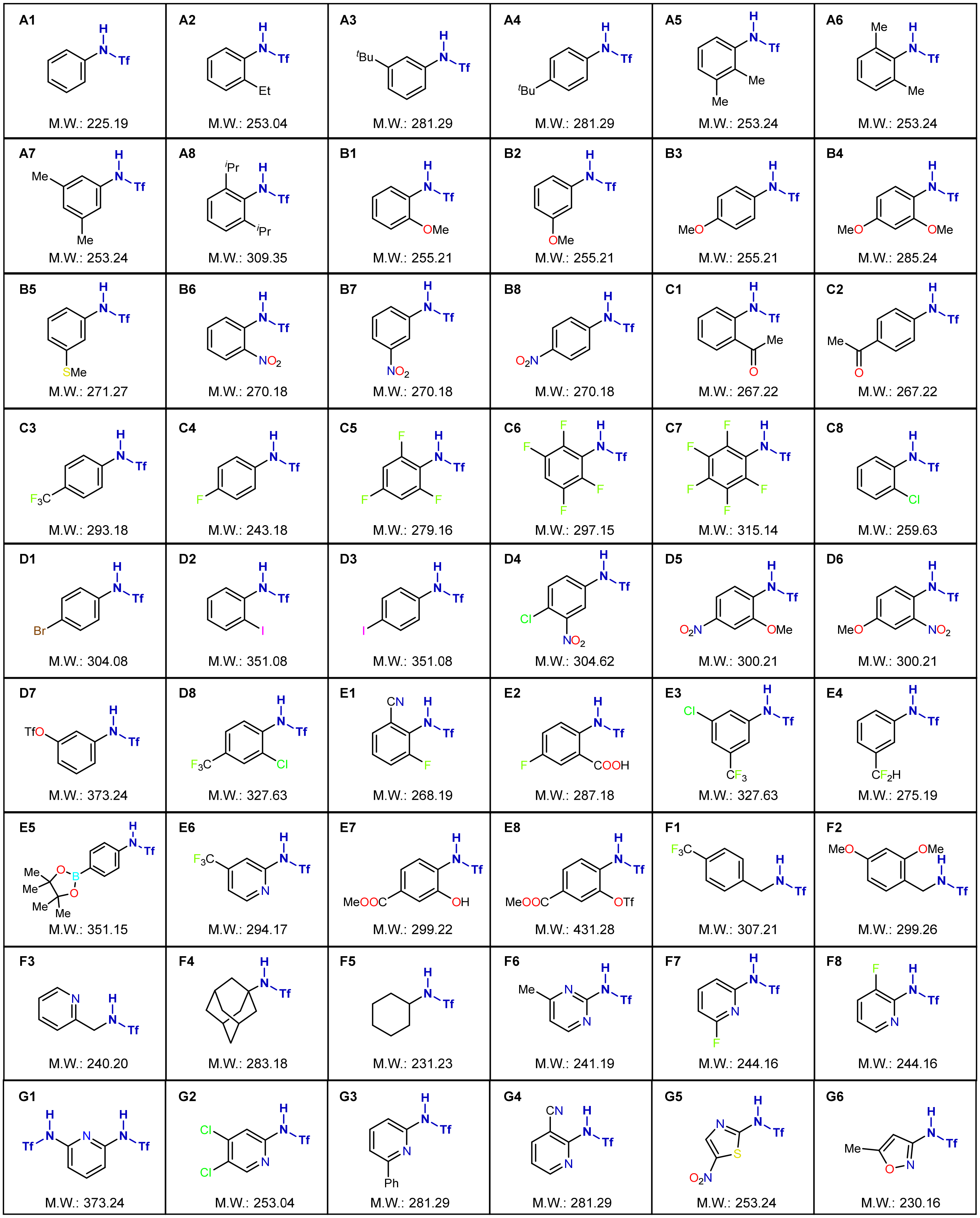

Figure S2. Aryl triflamide library (2/2).

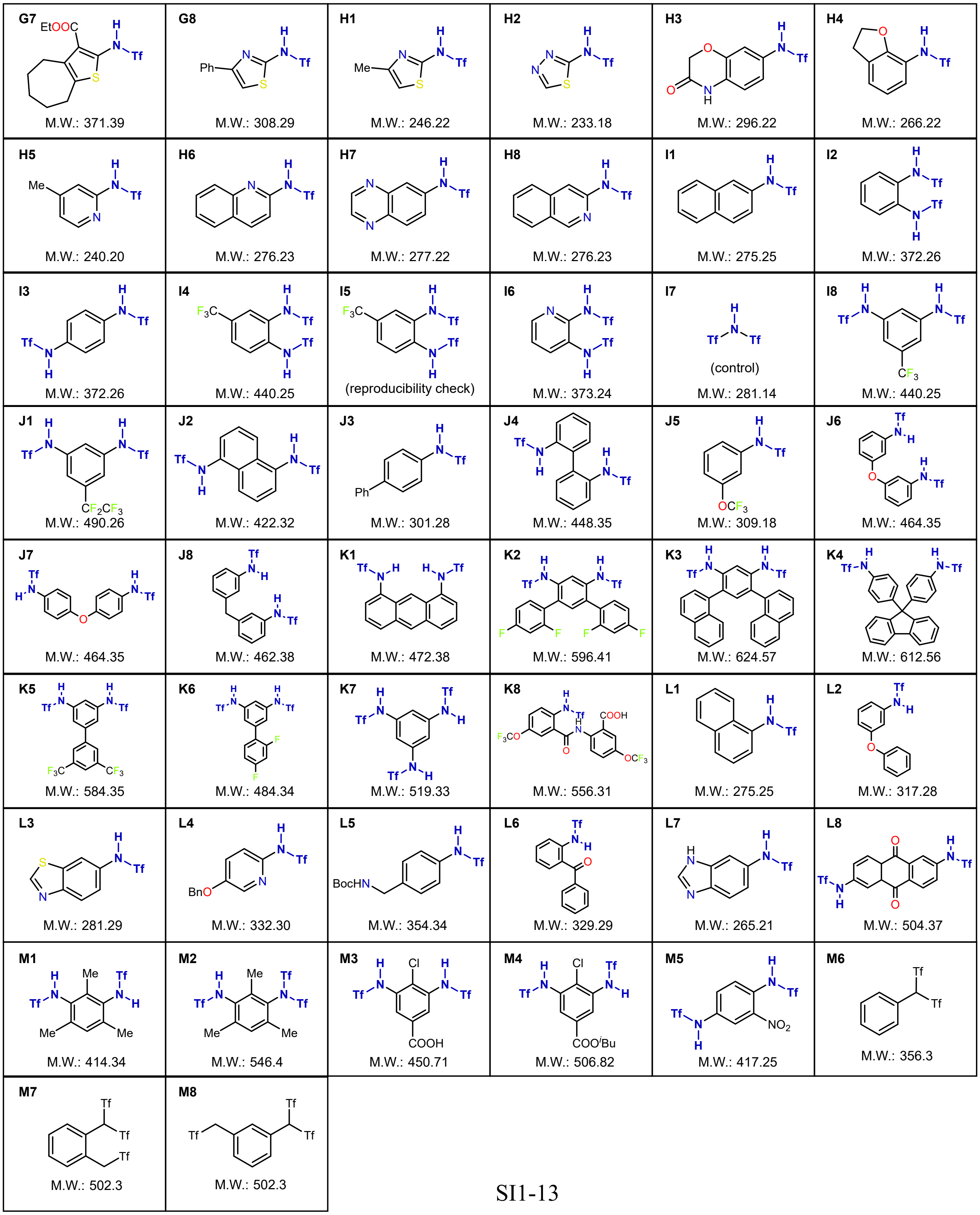

NMR titration for determination of the pKa for aryl triflamide I8 in DMSO

The pKa of aryl triflamide **I8** was determined using the Perrin method.[2,3] In 0.6 mL of DMSO-d_6, dissolve 0.06 mmol of aryl triflamide and bis(trifluoromethanesulfonyl)methane (pKa = 2.1 in DMSO).[4] 5 µL of TMS was added as internal standard. DBU was used as titrant and prepared by dissolving 80 µL of DBU in 400 µL DMSO-d_6. 5 µL of titrant was added each time to record a ^{1}H NMR spectrum. The pKa of **I8** was determined to be 3.1 in DMSO (Figure S3).

A graph was created using following this equation:

$$(\partial A^{\circ}-\partial a)(\partial b-\partial[B\text{-}H]^{-}) \text{ vs. } (\partial B^{\circ}-\partial b)(\partial a-\partial[A\text{-}H]^{-})$$

∂a The individual shifts for the peak in the spectrum of aryl triflamide

∂b Same as ∂a, but for bis(trifluoromethanesulfonyl)methane

∂A° The initial shift for aryl triflamide

∂B° Same as ∂A°, but for bis(trifluoromethanesulfonyl)methane

$\partial[A\text{-}H]^{-}$ The final shift for aryl triflamide

$\partial[B\text{-}H]^{-}$ Same as $[A\text{-}H]^{-}$, for bis(trifluoromethanesulfonyl)methane

Figure S3. NMR titration for determination of the pKa for aryl triflamide **I8** in DMSO-d_6.

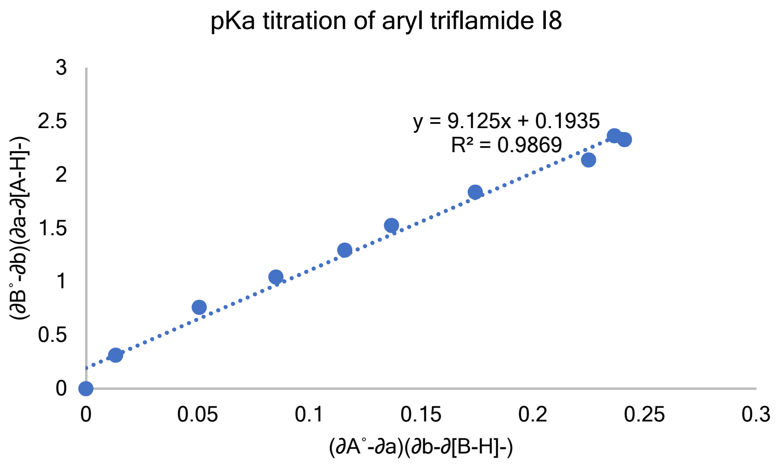

pKa (**I8**) = pKa (CH_2Tf_2) + log(9.125) = 3.1

The slope of this line is the difference in K_a between the two bases.

NMR titration for determination of the pKa for aryl triflamide I4 in DMSO

The method used above for pKa determination of **I8** was used to determine aryl triflamide **I4**. In 0.6 mL of DMSO-d_6 was dissolved 0.06 mmol of aryl triflamide and bis(trifluoromethanesulfonyl)methane. 5 µL of TMS was added as an internal standard. DBU was used as the titrant, prepared by dissolving 80 µL of DBU in 400 µL DMSO-d_6. 5 µL of titrant was added each time and a ^{1}H NMR spectrum was recorded. The pKa of **I8** was determined to be 2.8 in DMSO (Figure S4).

Figure S4. NMR titration for determination of the pKa for aryl triflamide I4 in DMSO.

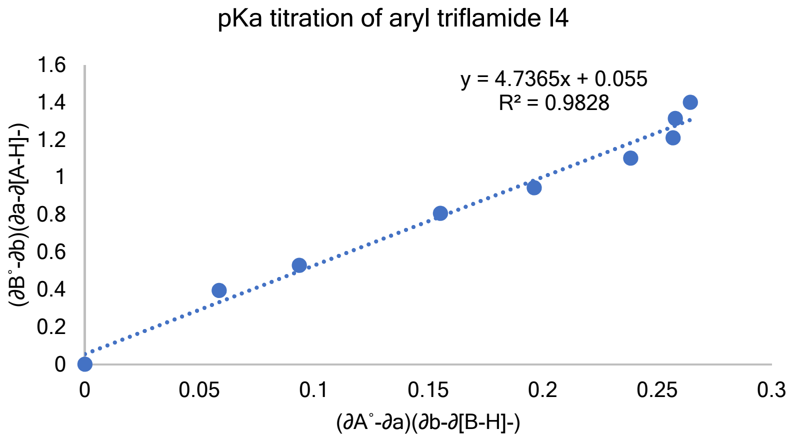

pKa (**I4**) = pKa (CH_2Tf_2) + log(4.7365) = 2.8

Acid Equivalence Study for $Lig_2{\cdot}n(I8)$

The reaction was set up according to the general procedure for HTE and was repeated twice at 5 µmol and 10 µmol and the average results were used to plot the graph. (Table S2)

Table S2. Acid Equivalence Study for $Lig_2{\cdot}(I8)_n$.

n	5 µmol yield (%)	10 µmol yield (%)	average yield (%)	5 µmol ee (%)	10 µmol ee (%)	average ee (%)
**0**	65	71	68	21	19	20
**1/3**	70	78	74	36	38	37
**0.5**	69	87	78	43	44	44
**2/3**	67	79	73	50	51	51
**1.0**	71	69	70	71	72	72
**4/3**	50	62	56	48	52	50
**5/3**	39	53	46	40	38	39
**2.0**	30	26	28	21	23	22

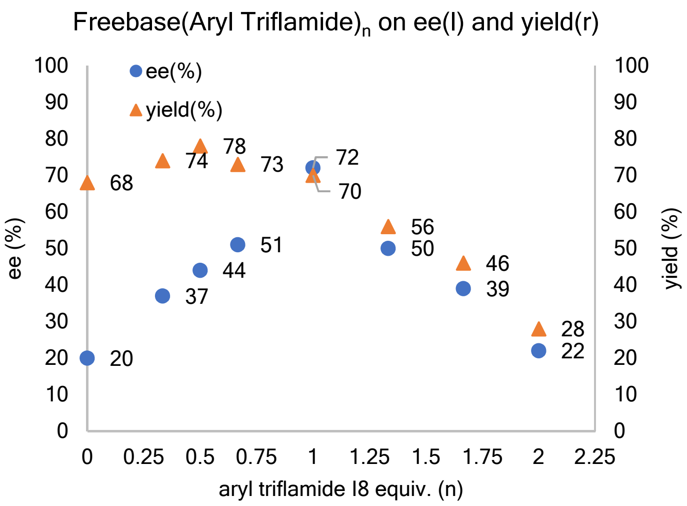

NMR Evidence for the formation of lig$_2$·I8 Ion Pair

A 1:1 molar ratio of lig$_2$·I8 (approximately 2 mg) was dissolved in 600 µL of DMSO-d_6. ^{1}H NMR spectra were recorded before and after mixing the components. Comparison of these spectra revealed a significant upfield shift of the signal corresponding to the 2-position aromatic proton of aryl triflamide I8, from 7.38 ppm to 6.78 ppm. This 0.60 ppm shift is consistent with the double deprotonation of I8 observed in pKa titration, providing strong evidence for the formation of the lig2·I8 ion pair.

Figure S5. NMR comparison lig$_2$, I8, and lig$_2$·I8.

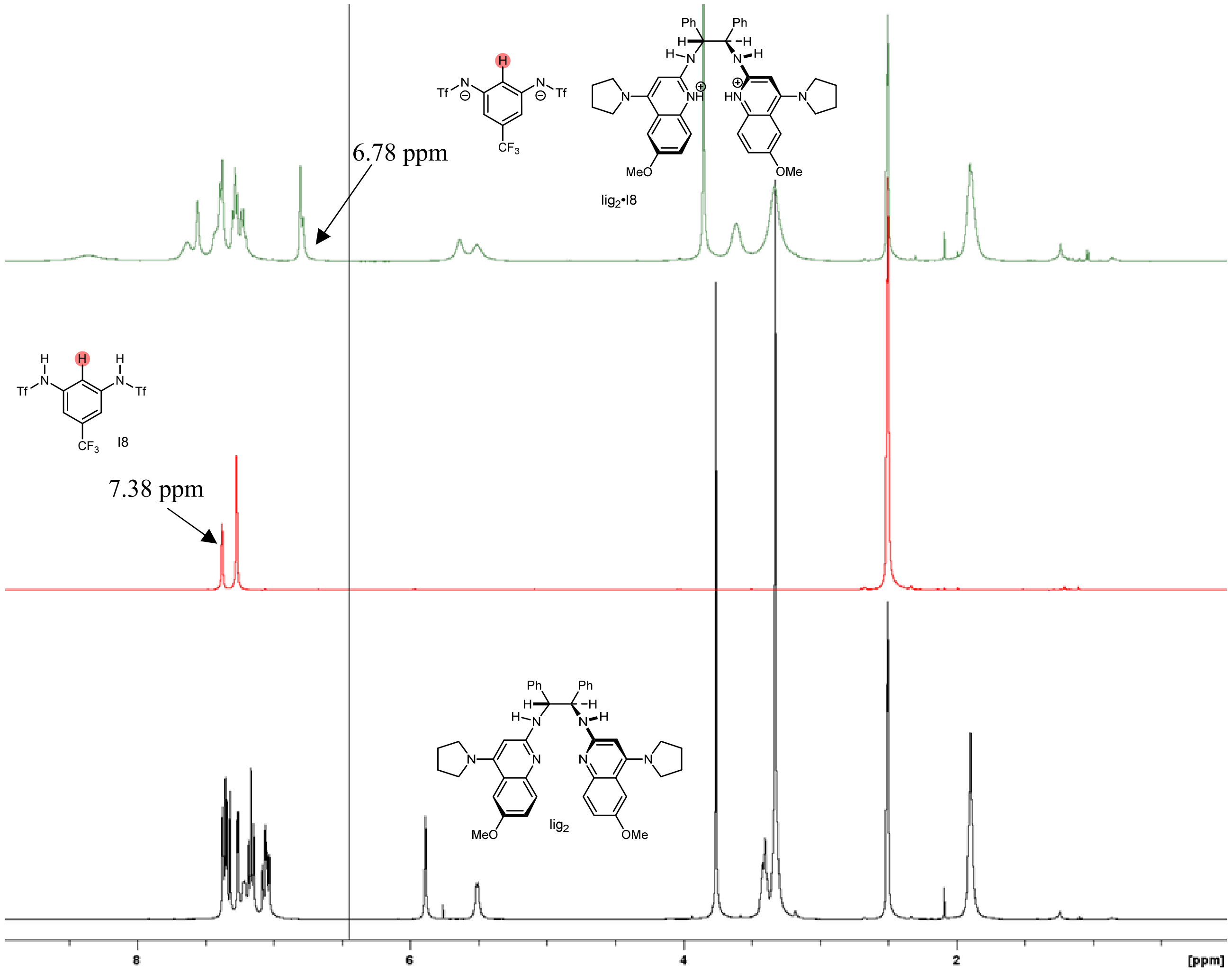

2D-DOSY Experiment

A 1:1 molar ratio of lig$_2$·I8 (approximately 2 mg) was dissolved in 600 µL of $CDCl_3$. 2D-DOSY NMR spectroscopy revealed a single major species containing proton signals from both lig$_2$ and aryl triflamide I8. The diffusion coefficient (D) of this species was measured to be 6.24×10^{-10} m^2/s. The molecular mass of the species was estimated using the Stokes-Einstein-Gierer-Wirtz Estimation (SEGWE) method,[5] yielding a value of 1198 Da. This estimated mass is 8% larger than the expected mass (1108 Da) of the lig$_2$·I8 complex. Notably, this discrepancy falls within the reported 15% root mean square (rms) error typically associated with the SEGWE method.

Figure S6. 2D-DOSY of lig$_2$·I8.

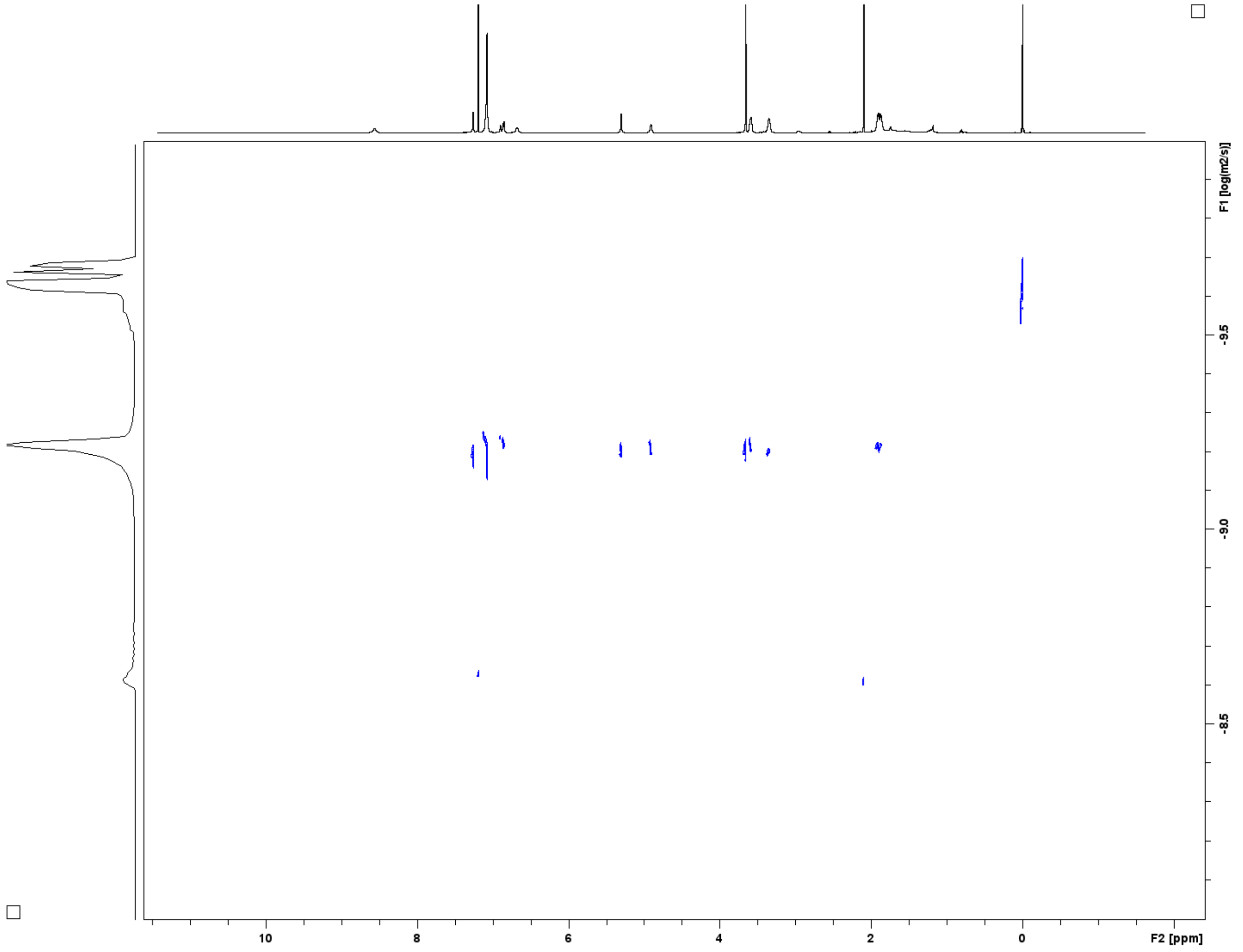

1D-ROESY Experiment

The I8 complex with lig2 tends to form a gel at the concentrations of NMR analysis, so the dimethylamino analog was prepared as a very soluble analog for NMR analysis. A 1:1 molar ratio of lig_{NMe2}·I8 (MW 1053, approximately 18 mg) was dissolved in 600 µL of $CDCl_3$. The solution was degassed using the freeze-pump-thaw method. The ^1H spectrum exhibited a single species, albeit with some broadening, suggesting an averaged complex. 1D ROESY (Rotating frame Overhauser Effect SpectroscopY) NMR experiments were performed, selectively exciting two chemically distinct protons on the lig_{NMe2} (designated as H^5 and H^7). Figure S7 presents

Figure S7. 1D-ROESY of $ligNMe_2$·I8.

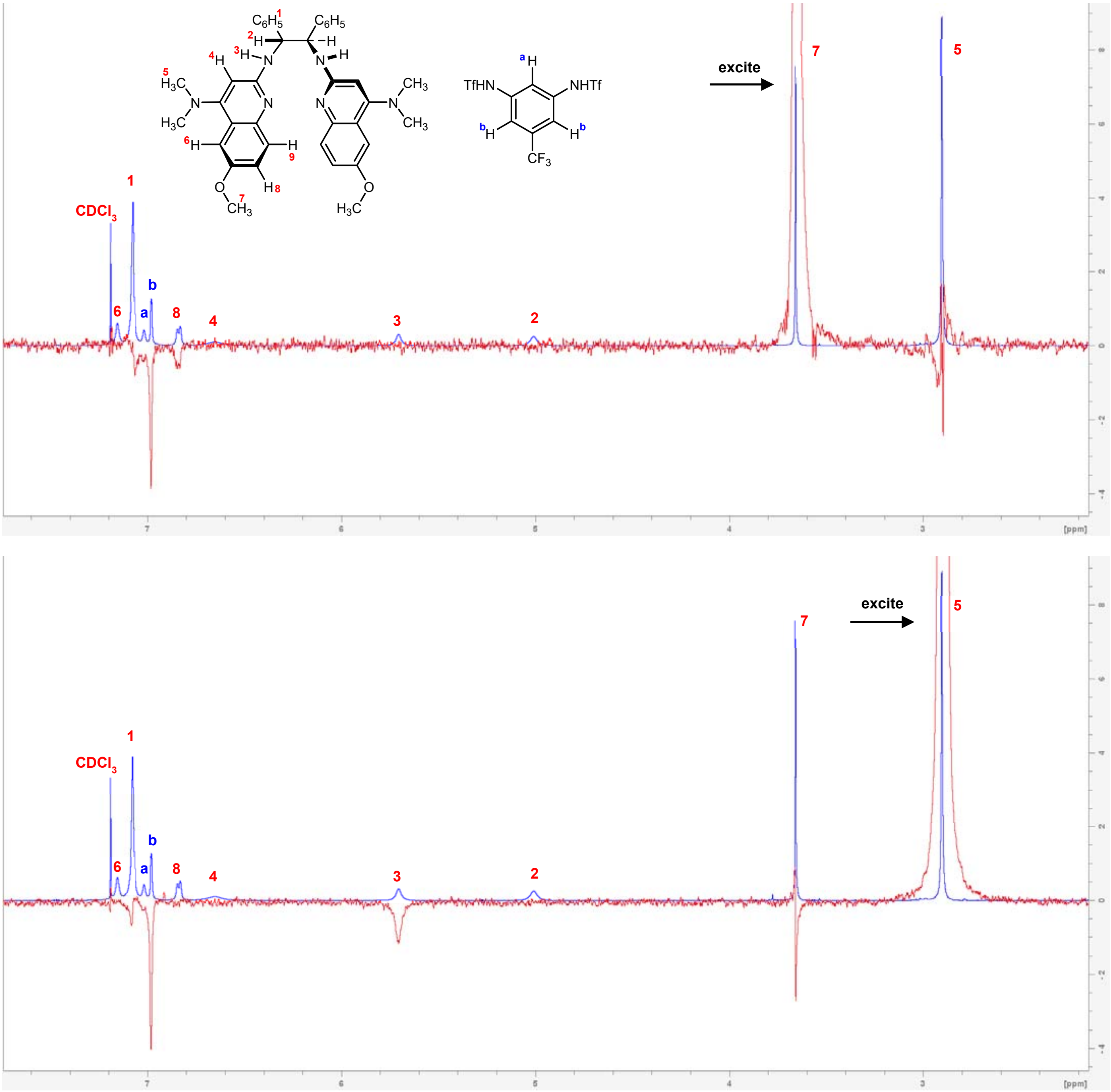

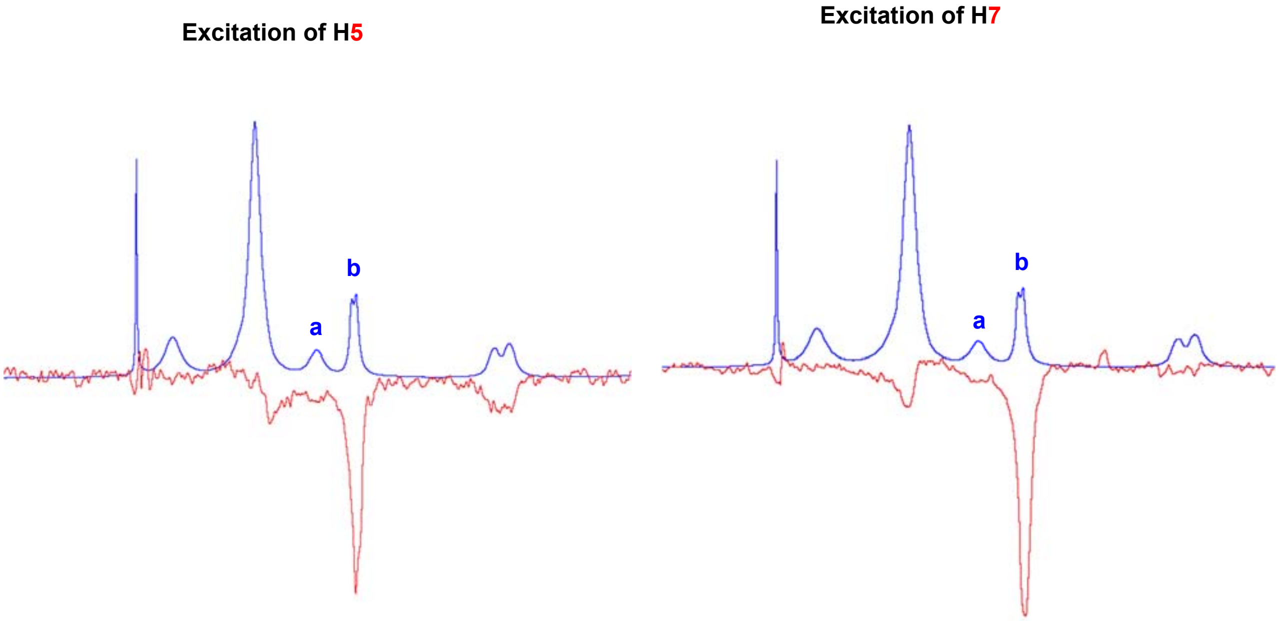

an overlay of the original ^{1}H NMR spectrum of ligNMe$_2$·I8 with the two 1D ROESY spectra resulting from these selective excitations. Close examination shows that both H^{a} and H^{b} of the aryl triflamide have cross peaks with both excitations. ^{1}H NMR of ligNMe$_2$·I8 (600 MHz, CDCl$_3$) δ 9.00 (br s, 2H^{9}), 7.22 (br s, 2H^{6}), 7.19-7.10 (m, 10H^{1}), 7.08 (br s, 1H^{a}), 7.05 (d, J = 1.9 Hz, 2H^{b}), 6.91 (d, J = 8.3 Hz, 2H^{8}), 6.72 (br s, 2H^{4}), 5.77 (br s, 2H^{3}), 5.07 (br s, 2H^{2}), 3.72 (s, 6H^{7}), 2.97 (s, 12H^{5}).

Figure S8 depicts two views of the minimized structure, highlighting the positioning of the aryl sulfonamide in the quinoline cavity after amidinium formation. The proximity of aryl sulfonamide H^{b} to both methoxy and dimethyl amino groups then follows.

Figure S8. DFT-optimized structure of lig$_2$·I8 illustrating pseudo-C2 symmetry, and highlighting proximity of I8-H^{b} to both MeO and pyrrolidine, consistent with ROESY spectroscopy.

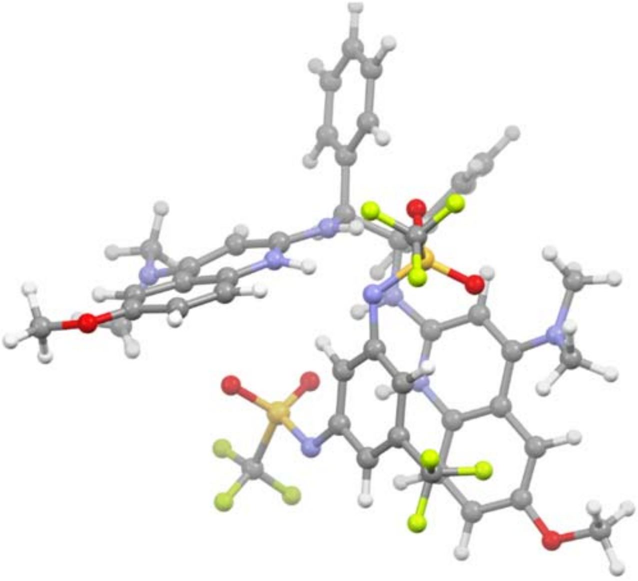

Study of Proton Source on the Reaction Outcome

The effect of different Brønsted acids on the conjugate azidation reaction was investigated using the optimal Lig2/I8 catalyst combination. Five representative acids with pKa values ranging from 2.83 to 4.19 were evaluated (FigureS9).

Benzoic acid (pKa = 4.19) provided good enantioselectivity (65% ee) with moderate yield (58%). 2-(Trifluoromethyl)benzoic acid (pKa = 3.2) showed comparable yield (59%) but slightly lower enantioselectivity (58% ee). 3-Aminobenzoic acid (pKa = 3.09) delivered improved yield (68%) with good enantioselectivity (64% ee). Notably, both isophthalic acid (pKa = 3.46) and malonic acid (pKa = 2.83) gave excellent results with 71% yield and 81% ee.

These results demonstrate that acids with pKa values in the range of 2.8-3.5 generally provide optimal reaction outcomes. This can be rationalized by their ability to efficiently generate HN_3 (pKa = 4.7) while maintaining sufficient catalyst activity. The comparable performance of structurally diverse acids (such as aromatic isophthalic acid versus aliphatic malonic acid) with similar pKa values strongly suggests that the primary role of the acid is to generate the active azide species rather than engaging in specific steric interactions with the chiral catalyst system.

Figure S9. Study of Proton Source on the Reaction Outcome

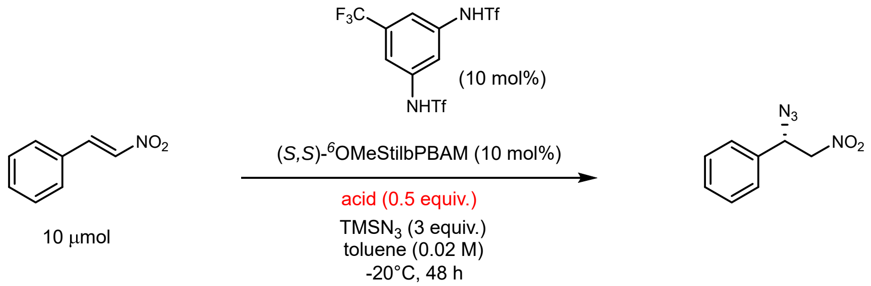

Acid	pKa	yield(%)	ee(%)
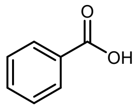	4.19	58	65
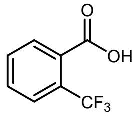	3.2	59	58
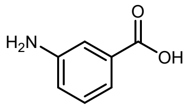	3.09	68	64
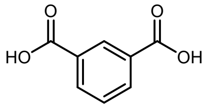	3.46	71	81
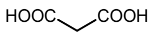	2.83	71	81

Catalyst Development for Azide Addition

Figure S10. A) Initial investigation of different chiral ligands for azide addition, and B) initial investigation of achiral acid additives using ligand lig_2 for azide addition.

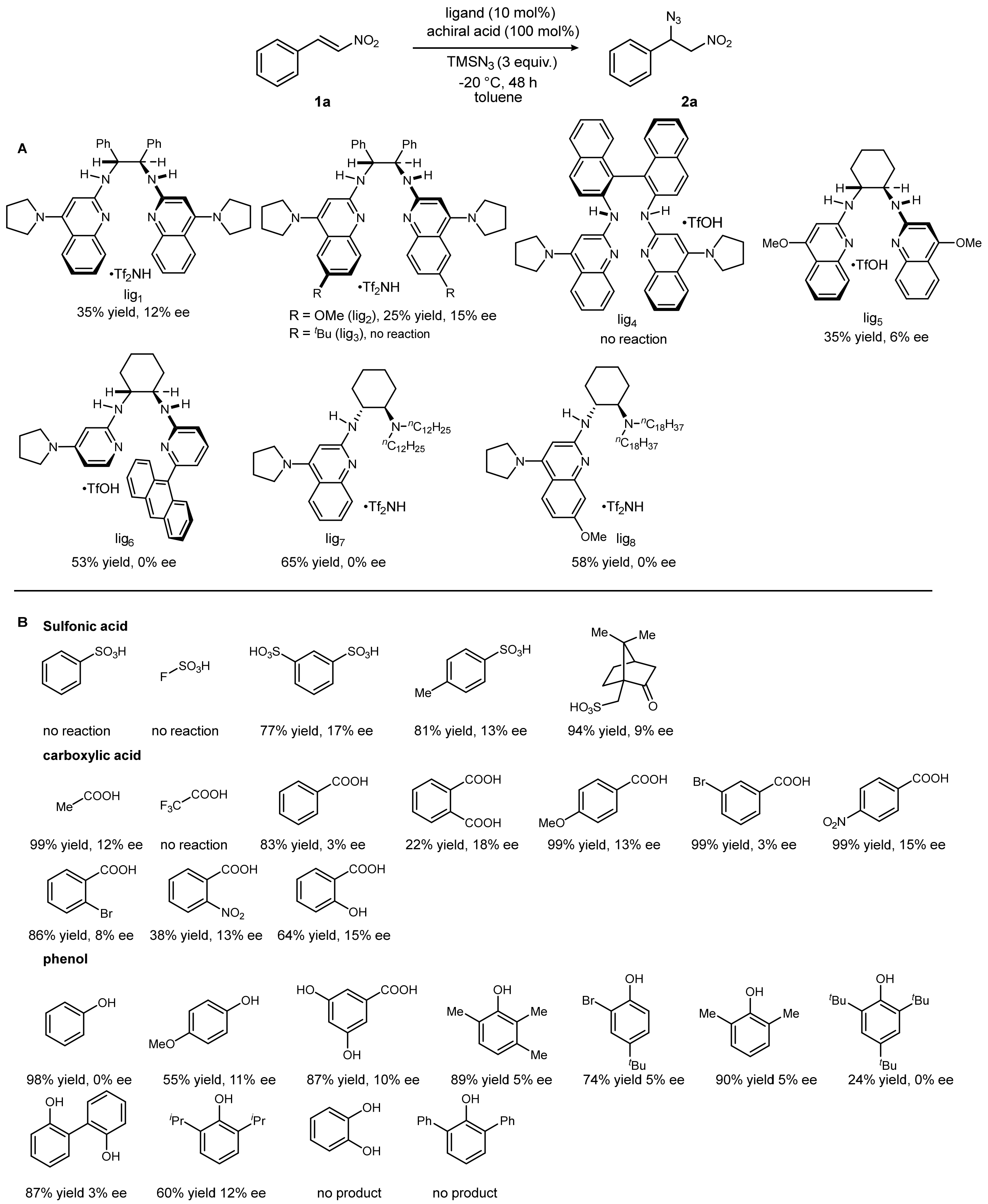

Initial evaluation. The results of screening different catalysts are summarized in Figure S10A, and the results using lig_2 with different achiral acids are summarized in Figure S10B.

Figure S11. Anion match test for lig_2.

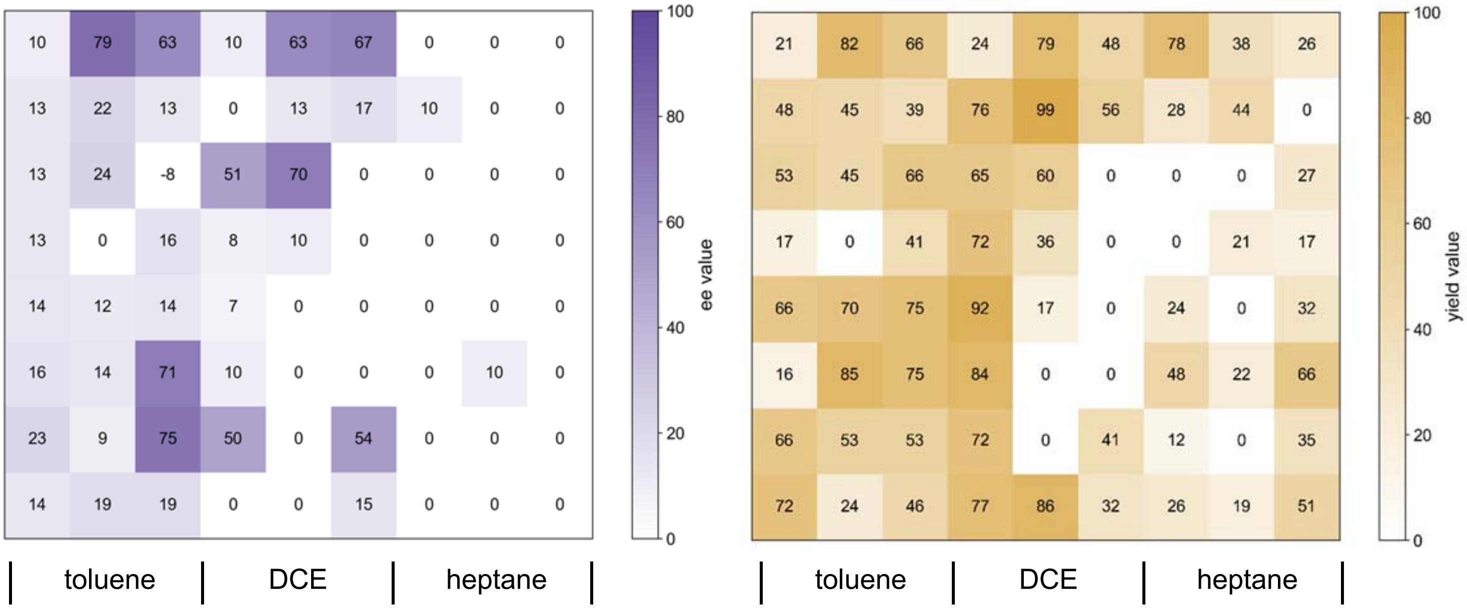

Anion match test. Based on the results of the initial evaluation, lig_2 was selected for the anion match test in three different solvents. The results are summarized in Figure S11.

Full anion screening. Since chiral ligand lig_2 demonstrated promising performance with various achiral aryl triflamides during the anion binding study, a full screening of all aryl triflamides in the library was conducted to potentially identify more effective options. The aryl triflamides used are identified by the x and y axis of the heatmap. The results are shown in Figure S12.

Figure S12. Full anion screening for lig2.

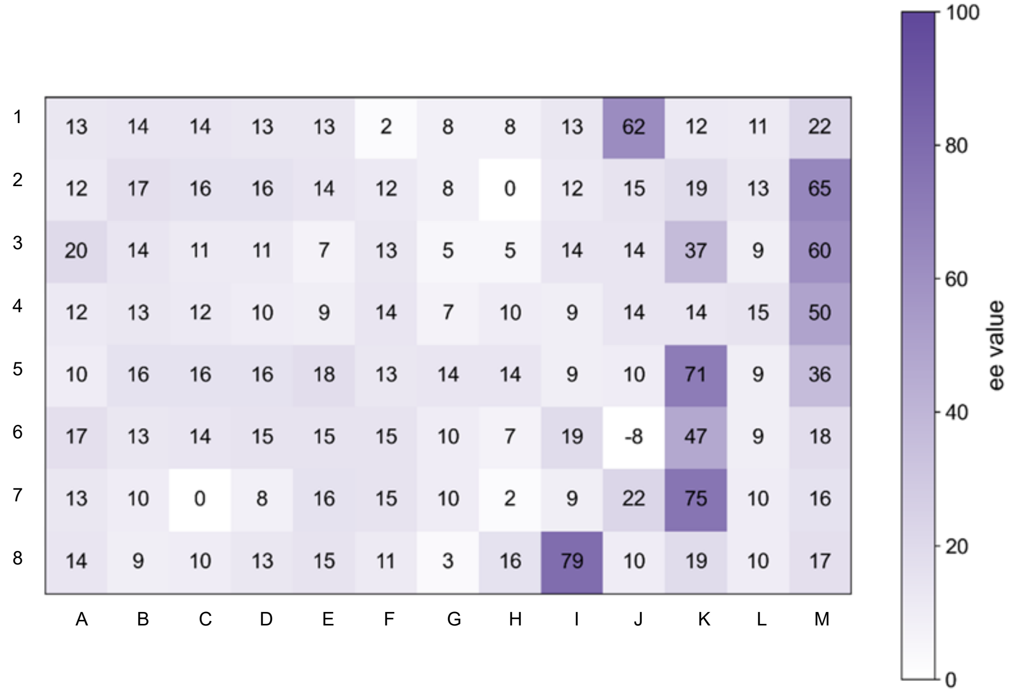

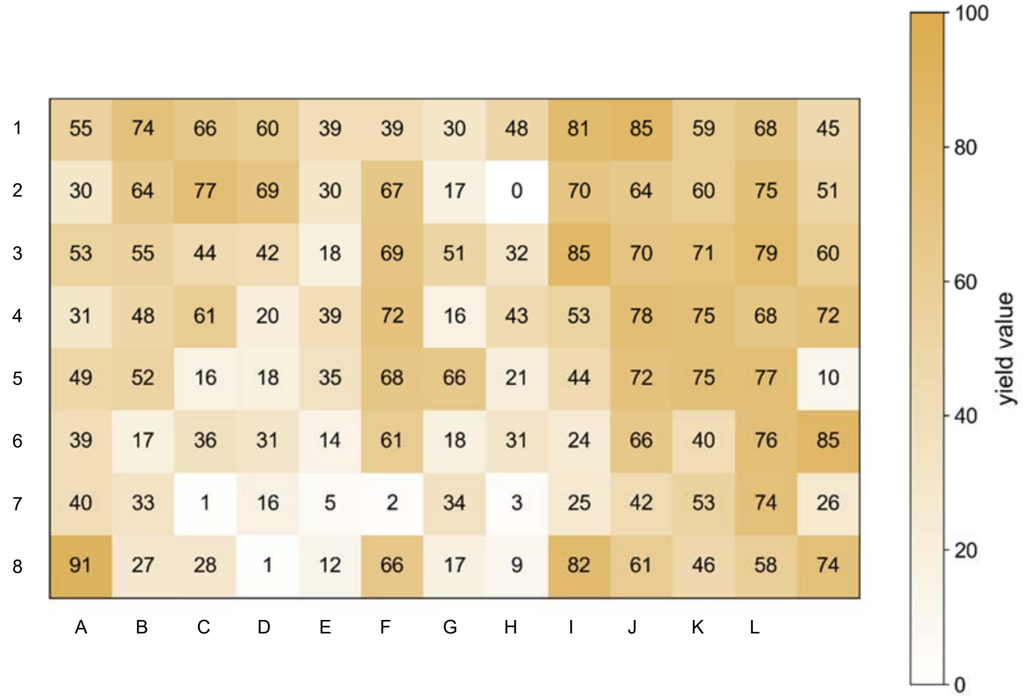

I5 is a reproducibility check that contains the same aryl triflamide as I4; I7 is the control group that used Tf_2NH as the achiral acid.

Aryl triflamide matching screening for challenging substrates

While aryl triflamide I8 demonstrated high enantioselectivity for most substrates, *ortho*-substituted nitroalkenes and alkyl nitroalkenes generally showed low to moderate enantiomeric excess (ee). Therefore, we conducted a focused anion matching screen to identify optimal aryl triflamides for these challenging substrates. Based on our previous findings that bis(triflamide)s consistently delivered superior enantioselectivity, we limited our investigation to this structural class.

The screen was performed on substrates **2b**, **2j**, **2m**, **2o**, **2r**, and **2s**, with results summarized in Figure S13. Notably, aryl triflamide M4 provided enhanced enantioselectivity (70% ee) for substrate **2m** compared to I8 (50% ee). Similarly, aryl triflamide M3 improved the enantioselectivity for substrate **2o** from 33% ee with I8 to 64% ee. These results demonstrate that while I8 serves as the optimal sulfonamide for most substrates, challenging substrates can be further addressed through counterion optimization.

Figure S13. Aryl triflamide matching screening for challenging substrates

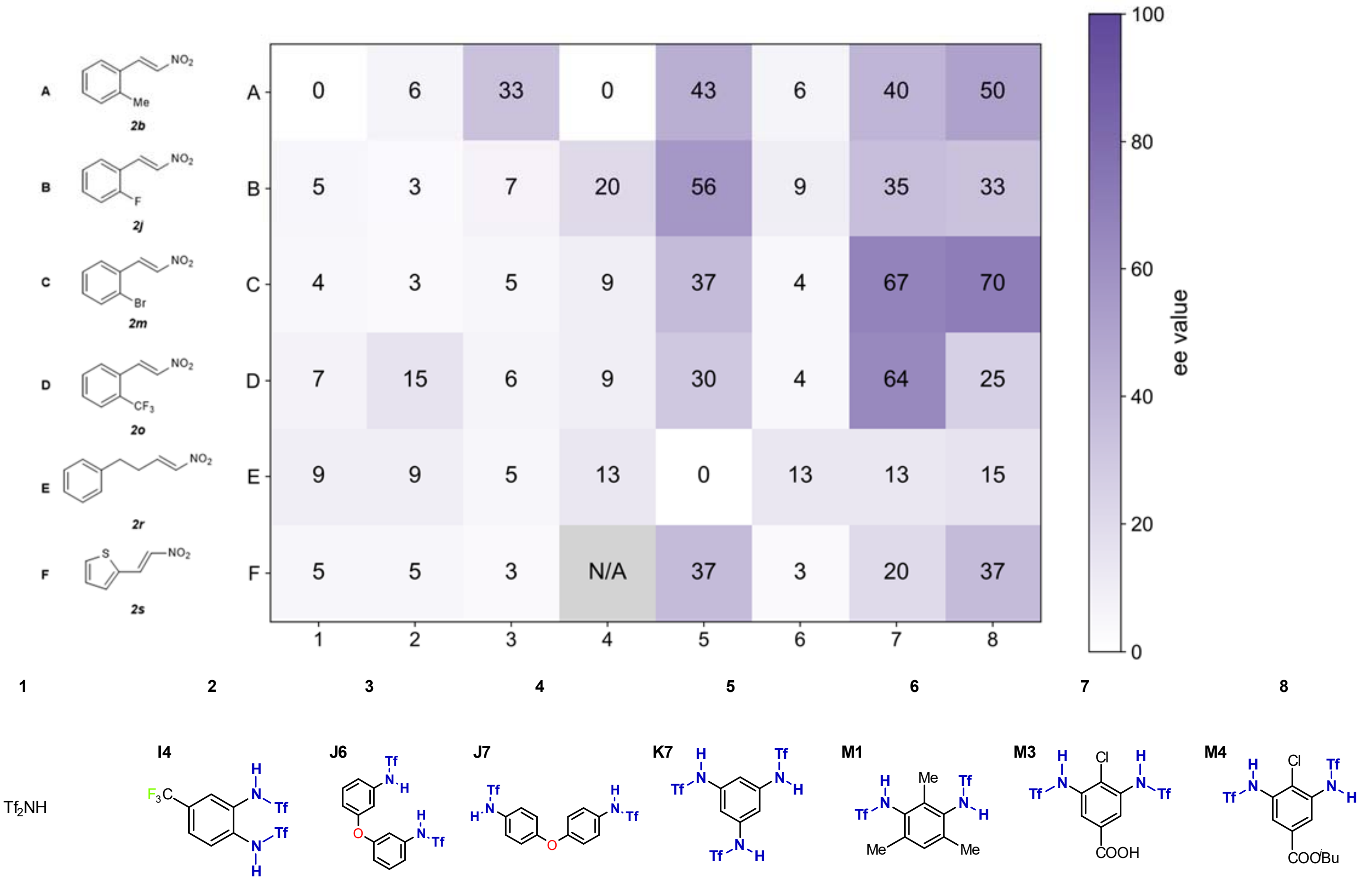

DFT Calculation

Methods

All DFT calculations were performed using the ORCA[6] 5.0.4 or 6.0.1 software package along with the xtb program package for GFN2-xTB[7] level calculations. Conformational searches of the ligand-aryl sulfonamide complex were performed with the Conformer–Rotamer Ensemble Sampling Tool (CREST)/Commandline Energetic SOrting (CENSO) workflow developed by the Grimme group.[8] Initial conformational sampling using CREST were performed at the semiempirical GFN2-xTB level with ALPB(toluene) as the solvent model using the iMTD-GC algorithm which combines iterative metadynamics simulations with Cartesian root-mean-square-deviation (RMSD) between generated structures as collective variables and genetic Z-matrix crossing. Default settings for sampling of non-covalent complexes were used. The unique conformers found were then sorted using CENSO, where screening was performed in three steps. In the first step, single-point calculations at the B97-D3(0)/def2-SV(P) level with solvation treated at the GFN2-xTB/ALPB(toluene) level were used to remove conformers that were more than 4.0 kcal/mol from the lowest lying conformer. Next, single-point calculations were performed at the r^2SCAN-3c/SMD(toluene) level with thermostatistical contributions calculated at the GFN2-xTB/ALPB(toluene) level; conformers above the 3.5 kcal/mol threshold were removed. Finally, the remaining conformers were optimized at the same level as the previous step iteratively using the default program protocol until all conformers were successfully optimized. Six counterions were selected for this process, specifically I3, I4, I8, J1, K5, and K7. The geometry of lig_2/I8 ion pair was later become the foundation for calculation of the transition state. Nudged Elastic Band (NEB) method[9] was used to identify the key transition states as well as the intermediates. All the structures in transition state study were optimized using r2SCAN-3c[10]/SMD(toluene) level of theories and the all the single point energies were calculated at wB97M-D4[11]/def2-TVZPP level of theory with SMD(toluene) as the implicit solvent model. Gibbs free energy corrections were calculated at a temperature and pressure corresponding to standard reaction conditions (233.15 K, 1.0 atm). The located saddle points were confirmed by both single imaginary vibrational frequency as well as the Intrinsic Reaction Coordinates (IRC).[12] I3 and I4 were selected to examine the effect of the 1,4- and 1,2-bis(triflamide) substitution pattern on ligand conformation compared to the 1,3-substitution, while the others were selected for high enantioselectivity in the azide addition to nitrostyrene catalyzed by their Lig_2-counterion salt. The doubly protonated Lig_2 without any counterion was optimized at the r^2SCAN-3c/SMD(toluene) level and compared with the optimized structures of different Lig_2-counterion complexes. In order to facilitate evaluation of topological distortion resulting a coordinating counterion, the counterions removed. The RMSD of optimized Lig_2-counterion complexes with counterion removed from doubly protonated Lig_2 without presence of counterion during optimization was calculated to quantify the degree of topological distortion as a result of the counterion. (Table S3)

Table S3. RMSD of Lig_2-counterion with counterion removed from doubly protonated Lig_2.

	I3	I4	I8	J1	K5	K7	Mean
RMSD (Å)	4.296	1.510	1.427	1.482	1.638	1.485	1.973

In order to extract geometric parameters from the optimized DFT structures, the coordinates of the lowest lying conformers for each Lig_2-counterion complex were parsed using the Morfeus library. Three functions were defined to compute distance, dihedral angle, and plane angle as desired, and the atoms from which these parameters are calculated are defined manually. (Table S4) The visualization of descriptors are shown in Table

S5. Then, multiple linear regression was used to study the relationship between the geometric parameters and $\Delta\Delta G^{\ddagger}$ of the reactions catalyzed by these salts.

Table S4. Geometry parameters calculated from optimized structures of lig_2-counterion complexes

	I3	I4	I8	J1	K5	K7
dihedral angle (°)	70.30235	60.04161	63.7416	63.10757	62.34652	63.15303
plane angle (°)	64.8749	46.55169	69.79975	70.68174	66.11475	68.85133
plane dist 1 (Å)	8.433111	11.14586	11.14167	11.20947	11.12541	11.13912
plane dist 2 (Å)	4.244009	4.664202	4.71976	4.714262	4.719362	4.716266
plane dist 3 (Å)	8.80384	9.341878	10.27162	10.34195	10.12974	10.23616
quinoline dist (Å)	5.194053	5.045341	5.585437	5.564858	5.526157	5.56872
near OH dist mean (Å)	5.694719	3.674901	3.350762	3.34761	3.314253	3.327064
far OH dist mean (Å)	4.685453	4.216526	5.201972	5.17255	5.097213	5.181068
near NH dist mean (Å)	5.525317	2.863432	2.89795	2.899348	2.89992	2.886582
far NH dist mean (Å)	4.491406	3.862397	5.182491	5.163938	5.081545	5.165288

Table S5. Visualization of geometry parameters.

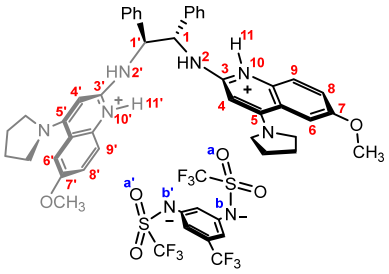

Descriptor name	geometric meaning
dihedral angle	$\angle\theta_1$ = 2-1-1'-2'
plane angle	$\angle\theta_2$ = plane (3-6-9) vs plane (3'-6'-9')
plane dist1	D_1 = 6 to 6'
plane dist1	D_2 = 3 to 3'
plane dist3	D_3 = 9 to 9'
quin dist	D_4 = 10 to 10'
near OH dist (avg)	D_5 = (a to 11 + a' to 11')/2
far OH dist (avg)	D_6 = (a' to 11 + a to 10')/2
near NH dist (avg)	D_7 = (b to 11 + b' to 11')/2
far NH dist (avg)	D_9 = (b to 11' + b' to 11)/2

By sequentially adding parameters that resulted in the greatest increase in R^2, the following ordering of geometric parameters was found starting from the greatest increase: quinoline dist, plane angle, dihedral angle, far OH dist mean, plane dist 1, plane dist 2, plane dist 3, near OH dist mean, near NH dist mean, far NH dist mean. The change in R^2 resulting from additional geometric parameters considered in the aforementioned order is visualize in Figure S14.

Figure S14. Change in R^2 as Additional Geometric Parameters are Considered.

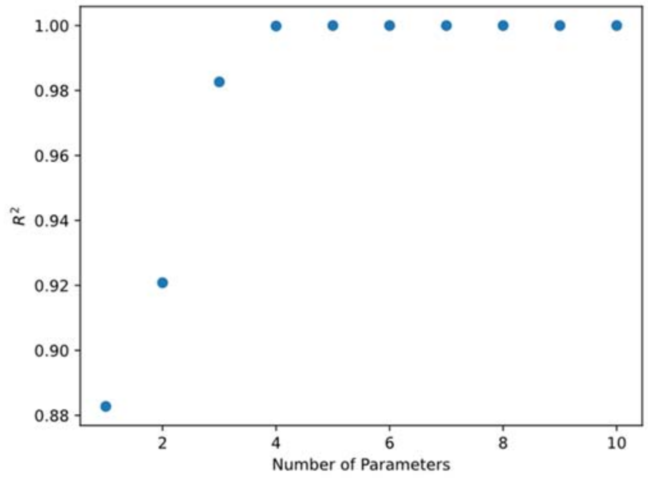

Figure S15. Full NCI plot of lig_2/I8 for TS1 (favored enantiomer).

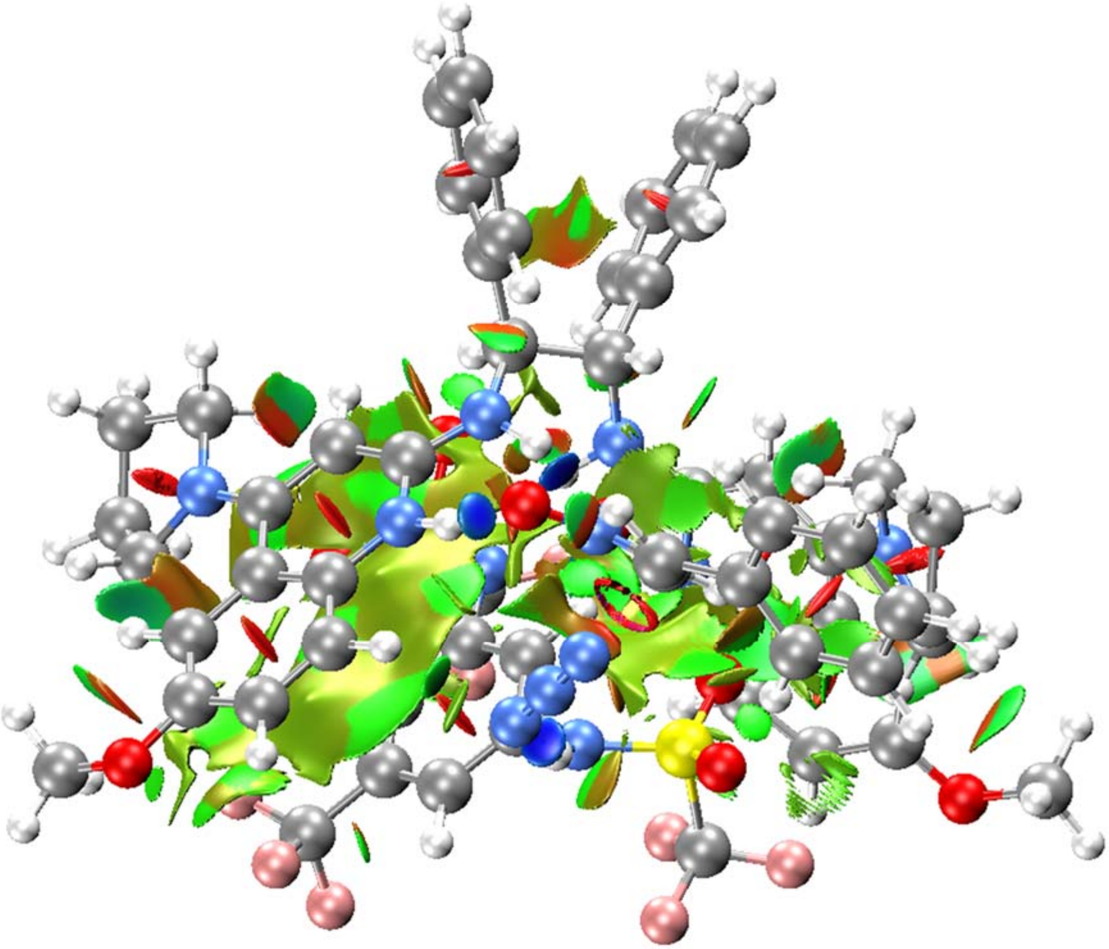

Noncovalent interactions plotting

Noncovalent interactions (NCI) were calculated using Multiwfn[13] with the grid setting of 0.1 Bohr and then visualized by VMD. NCI scatter plot were using the output file from Multiwfn and then plotted using Gunplot program. The detailed NCI plots for both favored and unfavored pathway are shown here.

Figure S16. Full NCI plot of lig_2/I8 for TS1. (unfavored enantiomer)

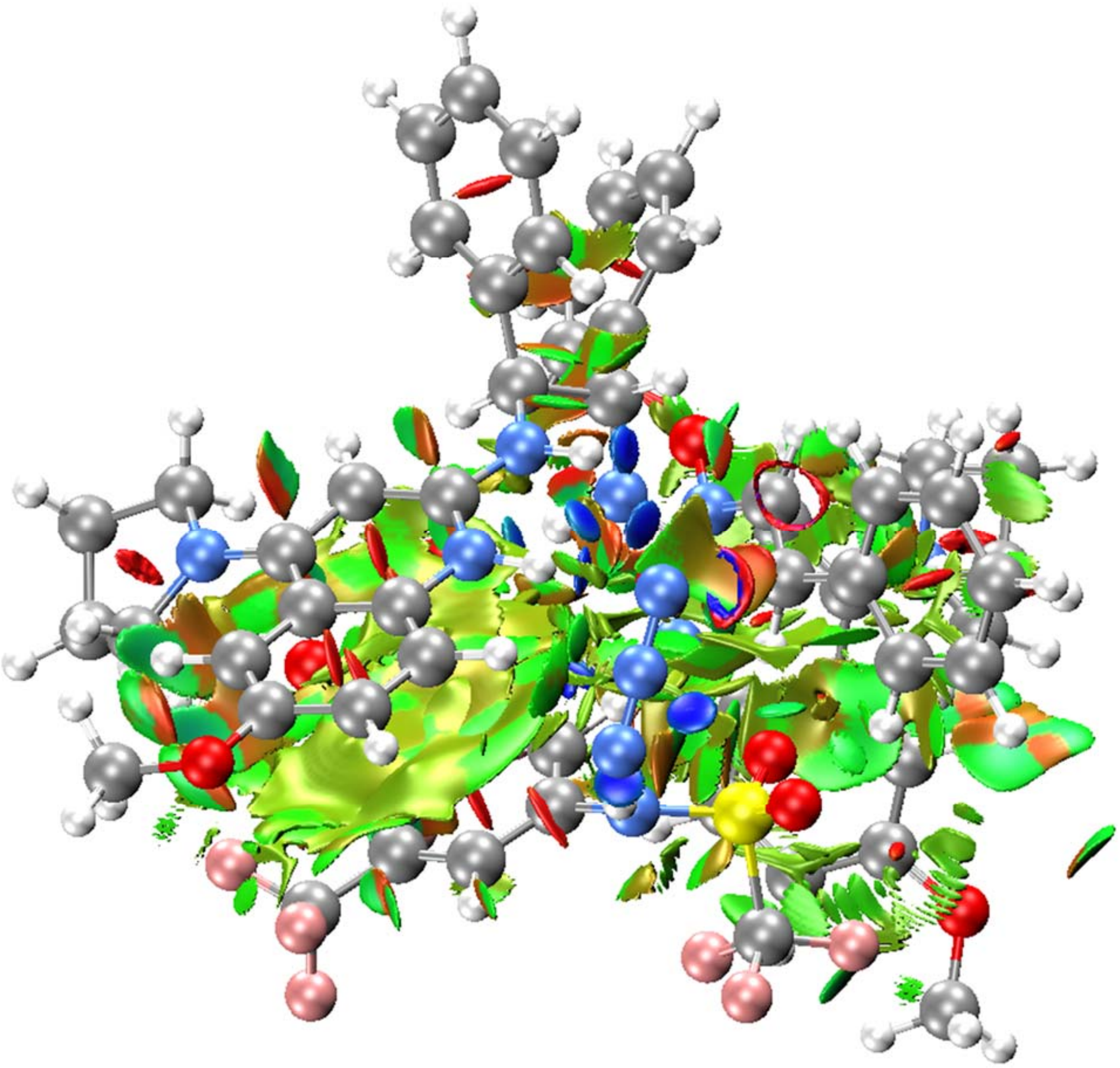

Cartesian coordinates of optimized geometries of reaction pathways

Uncatalyzed hydrozic acid and nitroalkene (SM)

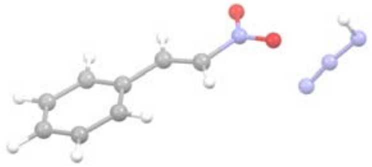

E -679.4165000

G -679.2720349

N	-2.39319289639419	0.47135402509049	-4.02852401014531
N	-3.26525638311181	1.14911743169466	-3.84832952612845
N	-4.24461145080385	1.89519686390107	-3.78789642234899
H	-4.37228974491670	2.17068559431108	-2.81442995142689
O	-2.95266046839354	-0.02978045334142	-0.83193804379818
N	-2.24310545455804	0.95371139863928	-0.75569510949620
C	-0.91940032168207	0.85954189218620	-0.17353023080838
C	-0.47461532136625	-0.30594441685081	0.27827020522671
C	0.83374366191331	-0.55438717504236	0.88277686504717
C	1.76813381550165	0.45865641504595	1.11363736820006
C	2.99149748739472	0.16184562083751	1.68486767160802
C	3.30159652329112	-1.14796540178861	2.03432244582330
C	2.38145716436710	-2.16070479787183	1.81139294499073
C	1.15409802669574	-1.86388737332494	1.24019502869317
O	-2.57269016785813	2.06279085903281	-1.15541556370109
H	-0.41486742908859	1.80872722573104	-0.17550492937631
H	-1.13607052223848	-1.15784377275890	0.18817928813761
H	1.54100260777964	1.48143296120872	0.85010375242988
H	3.70723601123027	0.95213863724921	1.85993878567514
H	4.25932880496082	-1.37519811051674	2.48047401450381
H	2.61811402943969	-3.17943800086245	2.08239768820159
H	0.43255002783759	-2.65004942256995	1.06470972869262

Uncatalyzed TS (TS)

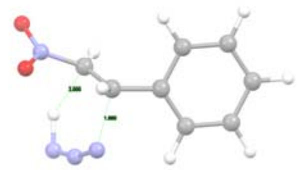

E -679.3517250

G -679.2069604

N	-1.44668629236779	-1.50787964742049	-1.14189411232006
N	-1.62620627591573	-0.99578674070988	-2.14529874560898
N	-1.76496656674266	-0.12591288753547	-2.97347720226514
H	-1.75665241256747	0.76008561419188	-2.37823286397884

O	-3.67153910353676	0.65437637728566	-0.06919968515552
N	-2.82577638976274	1.49813094513003	-0.31560301973502
C	-1.44131638581219	1.08009166451429	-0.42934617156167
C	-1.06543217595363	-0.12305701029602	0.08652420105773
C	0.38269129345114	-0.32778344802251	0.42550000027441
C	0.87348619154320	0.42603757640909	1.49260240888398
C	2.19097385649873	0.29881043865921	1.89959940082919
C	3.04139651939890	-0.57604322579606	1.23846347076870
C	2.56193120449610	-1.32027867488862	0.17295095019689
C	1.23864548671497	-1.20284719605229	-0.23151308062883
O	-3.06027366154024	2.68242428823795	-0.48063212045459
H	-0.77816927956208	1.86897067717609	-0.73894002655428
H	-1.79443616258662	-0.61867254941156	0.71250837980264
H	0.21409598363590	1.11009004116123	2.00795113424604
H	2.55219629497371	0.88662778258673	2.73123420186107
H	4.07103901514166	-0.67409561471865	1.55110644414866
H	3.21766963282089	-1.99978647810521	-0.35231310164746
H	0.88732922767267	-1.79350193239539	-1.06199046215892

Uncatalyzed product (Prod)

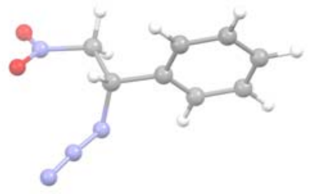

E -679.4332207

G -679.2829867

N	-1.33213032019679	-0.95869801140678	-1.33249447826372
N	-2.35539269693992	-1.61854487906552	-1.22397807336118
N	-3.26209600615517	-2.27861396207070	-1.23901929774306
H	-0.47839922516010	1.55194452885569	-1.58419854841422
O	-3.43959636574899	1.08879378582687	-0.75000425708032
N	-2.50877159400296	1.75366502795173	-1.15920966509144
C	-1.11745853726903	1.38331252991445	-0.72343874687825
C	-1.02207333495408	-0.04639226428628	-0.21569217929124

C	0.37952878682253	-0.29457061273795	0.29783926901118
C	0.66528902193724	-0.06558299731474	1.64071250970480
C	1.95582609549235	-0.23825762591073	2.12344066419058
C	2.97072997810275	-0.64254472761102	1.26556777545884
C	2.68870671932311	-0.87398808457854	-0.07514464626852
C	1.39904490475152	-0.69936665848328	-0.55912922735027
O	-2.60950749437372	2.71226699475129	-1.89307621541476
H	-0.86451833617314	2.09969580376300	0.05499012851791
H	-1.73504407796446	-0.18440711519534	0.59561746301380
H	-0.12591214896546	0.24110174893680	2.31255452482250
H	2.16525318736176	-0.06439494683601	3.16948361201344
H	3.97469587971017	-0.78166466799440	1.64085867374923
H	3.47355057193262	-1.19339394072669	-0.74623349657119
H	1.17827199246980	-0.89036092578182	-1.59944378875415

Iig$_2$/I8 with hydrozic acid and nitroalkene (SM)

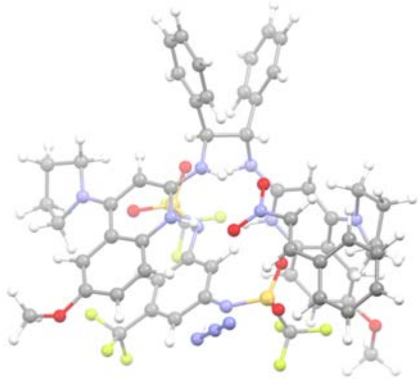

E -5239.486510

G -5238.477131

C	3.20814177347477	1.40140905386557	-4.77092312762910
C	3.61326816255014	1.78840002318329	-3.50004821703430
C	2.68468000304219	2.02225436070802	-2.47139172795443
C	3.03146181205469	2.40913127741745	-1.09610052547377
C	2.01952273788184	2.95927685280993	-0.30163070994839
C	0.66924254452084	2.80656292745452	-0.66535451681521
N	-0.37818879347557	3.10730044034151	0.12408880444488
C	-0.35107167501963	3.47079124087590	1.52574100653705

C	-1.41142588492975	2.58993463344276	2.24308015091973
C	-1.64563064177377	3.02242948038367	3.67776260194262
C	-0.75099788187457	2.65577746142797	4.68752884778681
C	-0.92967968331795	3.11803774358763	5.99048860833448
C	-2.00697809940988	3.95097500808698	6.29633284317147
C	-2.90794273553257	4.31271432012989	5.29493593641852
C	-2.72788467308170	3.84737977607694	3.99285686601970
N	-0.99233354949837	1.20648113910465	2.12450438977900
C	-1.81569701933197	0.15059083805690	1.97721011334506
C	-3.20997492439098	0.24989549376261	1.86190168851113
C	-4.00389765384839	-0.85211492918692	1.52206467133025
N	-5.32617778534820	-0.66520959014218	1.32604835997134
C	-6.21575915813149	-1.48214148145369	0.48223001092895
C	-7.29067788032300	-0.49909117600216	0.02302707242693
C	-7.40323611106238	0.45232596593244	1.21241050680759
C	-5.94522172847909	0.63520100489835	1.61812616476545
C	-3.35185168648308	-2.16718013161927	1.44664401654296
C	-4.02205659726769	-3.39992149476161	1.23940904610000
C	-3.33076600386381	-4.59876798062298	1.21021875049519
C	-1.93618355420102	-4.61398759993260	1.42110794060396
C	-1.26854261393973	-3.43844925770401	1.64827845041801
C	-1.96496183027384	-2.21395950358081	1.66347737629509
N	-1.24186048130843	-1.06082380857351	1.89183125289726
C	-0.60995186555333	4.94778128815844	1.75367117835530
C	-1.60976710299299	5.60602881382825	1.03066703764141
C	-1.90493610406799	6.94187366350027	1.29826997556159
C	-1.20427547546230	7.63128065899941	2.28935955457050
C	-0.20237487591251	6.98014076058277	3.00855948000165
C	0.09291682841494	5.64404075112529	2.73891894855846
N	0.36021646125412	2.26282038693670	-1.85404183393768
C	1.31624523856652	1.93203952716505	-2.79153987345260
C	0.90669656500081	1.52422994714451	-4.06760284759108
C	1.83875176852842	1.24937402328982	-5.05106995483844

N	4.27543421861496	2.26510727153943	-0.60034160551499
C	4.57081517206264	2.67085884064213	0.77884040136395
C	5.96892539720902	2.12117015525705	1.04941684622485
C	6.01666331245766	0.87607195076980	0.16656544145457
C	5.30418543014858	1.33654209308129	-1.10015264777763
H	4.68063226437786	1.91745334307747	-3.35090294391515
H	2.23978451705059	3.37929292294811	0.67295444655687
H	-1.30837473139582	2.92611664357885	-0.25826884180967
H	0.63077441585213	3.21587057220701	1.94952690007561
H	-2.34412764421021	2.72206115607667	1.67740495339321
H	0.09555795491157	2.00350190858803	4.45606846105800
H	-0.22368334531336	2.82630408215875	6.77212829014690
H	-2.14665439990982	4.31388779151606	7.31764337683131
H	-3.75691859776330	4.96002119009446	5.52804658719235
H	-3.42984784449326	4.13726473013915	3.20622674496074
H	0.00324153599253	1.01383902538354	2.23255462274970
H	-3.65375785549857	1.23320085455463	1.95002873288642
H	-6.67859533855601	-2.29306123885259	1.07021289925227
H	-5.65708077478436	-1.92010609062451	-0.35484670620809
H	-8.23333777271301	-1.00928737093931	-0.22425069815995
H	-6.93617923738868	0.04729583404759	-0.86267915457416
H	-7.97761162362304	-0.01508707611238	2.02966847874324
H	-7.88171165762899	1.41046464216531	0.96273154800547
H	-5.81331854253247	0.88790187337382	2.68247112173353
H	-5.47447587342762	1.41983831832818	1.00593082796641
H	-5.09530006080788	-3.41633347604023	1.11205997499078
H	-1.39825944776212	-5.56220463986033	1.37635321981855
H	-0.18641991361187	-3.43918699242198	1.78575628936006
H	-0.22509541973352	-1.14858372135085	1.96699277476075
H	-2.16855350284138	5.06987201794004	0.25853551450149
H	-2.68893606178949	7.44771149791149	0.72921256887357
H	-1.43804100906014	8.67812725243684	2.49886501389808
H	0.35324355971365	7.51429555259810	3.78330266738924

H	0.87202639461659	5.13150245603966	3.31003255291479
H	-0.61296632254563	1.90767695716016	-1.95925775384555
H	-0.16184689178483	1.44526226971502	-4.27815452634562
H	1.49430706795845	0.93460355242889	-6.03587461543451
H	4.50640966605970	3.76577426282489	0.88571848245513
H	3.82704544358281	2.22155666756185	1.45916159397968
H	6.12797603495441	1.91264330406739	2.11798369270489
H	6.73647760086498	2.84573204628590	0.73160949158461
H	5.44235688940204	0.05437515927133	0.61996564640800
H	7.03586803319054	0.51595425686199	-0.03512873873547
H	4.83751486039337	0.50396522248810	-1.63491580721455
H	6.00650742098080	1.85506994740773	-1.77469450892984
O	3.53520515653664	-2.68422377263623	-0.09622911091311
S	2.49280163516945	-1.88207175407509	-0.74568340880034
N	1.14295043677589	-2.66460726916856	-0.69901357146996
C	-0.09601669637988	-2.18622783668207	-1.14619618413336
C	-0.48438698189919	-0.84471464034570	-1.11846923109876
C	-1.77253637229705	-0.43950052395594	-1.50379786954335
N	-2.01664114349988	0.93778644255619	-1.41830799378086
S	-3.40364291762246	1.63921662671094	-1.41003394737104
O	-3.28511935065068	2.92492817653500	-0.69219769881718
C	-3.63748423647785	2.18730707812045	-3.16253116309945
F	-2.62276977050200	2.97218470357155	-3.53787884498315
F	-3.65726671776913	1.12188932328153	-3.96552095903203
F	-4.77321662534008	2.86012482407203	-3.31525496641252
O	-4.59532041804135	0.82565674400825	-1.14985324053192
C	-2.69588291198271	-1.40349847913776	-1.92677383852236
C	-2.31054725995310	-2.74544695432707	-1.93275576046475
C	-3.30334894123023	-3.77824158170068	-2.38757595032416
F	-4.57172257753095	-3.43991883287372	-2.06492699128854
F	-3.28988055065499	-3.94690460812242	-3.72163737992566
F	-3.07703136260459	-4.98492379141683	-1.84416084322248
C	-1.03317260090914	-3.14747662191744	-1.56434707952078

C	2.97831304928362	-1.93900356694605	-2.53669227162044
F	2.07095941436186	-1.30119337532723	-3.27875550029268
F	4.16773615561853	-1.36433337204488	-2.74350376878230
F	3.05175121522690	-3.20042631692595	-2.95144188056516
O	2.47794326695828	-0.43812629689697	-0.44889653450458
H	0.21473198083959	-0.09601538884919	-0.75334108596519
H	-3.70162538274633	-1.11495793223980	-2.22314098110552
H	-0.74979122071261	-4.20002350502402	-1.56583830966846
N	3.24949066567068	-5.14382890755391	2.04709224735474
N	2.40421013661676	-5.09004867925925	1.29373788753380
N	1.46586832193624	-5.14402767961348	0.51855870799033
H	1.36403987243067	-4.23411400595912	-0.02464476053357
O	1.64170476151805	-1.27557521472797	2.31749231065087
N	2.35922201831500	-0.30923803383488	2.51014639455950
C	3.76835069689104	-0.48998170486911	2.70692242279920
C	4.31202037682871	-1.70688823070245	2.56246324403514
C	5.74038257575478	-2.00960346270352	2.65540585924215
C	6.67764361908374	-1.11046155263537	3.19224916597899
C	8.02849580041771	-1.43691274126620	3.21433691375371
C	8.46131319426341	-2.66230132767785	2.69860836692486
C	7.53840023868666	-3.56320872396953	2.16894803786740
C	6.18306231031082	-3.24127533434337	2.15027827755700
O	1.93607545523127	0.83957554522528	2.55572687396677
H	4.28032305483551	0.44547852037496	2.91589695860408
H	3.64802973942989	-2.53265132481827	2.30406272349808
H	6.34996236848282	-0.15479421445336	3.60737117303964
H	8.75085540963549	-0.73494417320596	3.63737879378534
H	9.52396248134468	-2.91653421624344	2.71860171645851
H	7.87443262658560	-4.52215671886423	1.76816696631881
H	5.45158888788909	-3.93809661895337	1.73670643345935
O	4.19717720945026	1.19508834481637	-5.67464169993035
C	3.84921842232130	0.77566730977271	-6.97270967158412
H	3.32509981095446	-0.19725228385024	-6.96317522612121

H	4.79233458848072	0.66248402192957	-7.52613726789054
H	3.22120927569375	1.52215885676684	-7.49241725580002
O	-3.90404996537629	-5.80274014953110	0.98827538861205
C	-5.26044646840155	-5.84235202235032	0.61344135135932
H	-5.48979509904533	-6.89240528871350	0.38274425923683
H	-5.45183381425427	-5.22757827422829	-0.28307121710796
H	-5.92311728130121	-5.50830716282210	1.43336499156408

Iig$_2$/I8 TS1 (favored enantiomer)

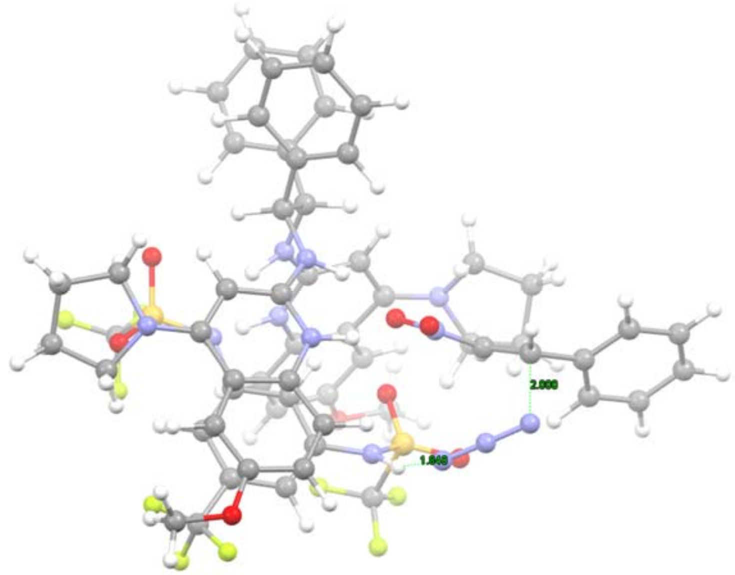

E -5239.473789

G -5238.451936

C	4.54759640701068	0.89989995547204	-5.78099863399021
C	4.73425209617219	1.40397074894783	-4.50897607329361
C	3.67900455785424	1.39870702536662	-3.57229938821462
C	3.80859111201146	1.82581496019151	-2.17694998018298
C	2.64084556370691	2.17052255644515	-1.49445168430555
C	1.40652447908231	1.69081170901095	-1.95043370659709
N	0.26358911782776	1.72249415764367	-1.24977030980754
C	0.04413453071593	2.40614992972368	0.00773082228485
C	-1.10558629415035	1.65915564743087	0.73182722282667
C	-1.53787696325172	2.37762439534973	1.99425417846720
C	-0.84012812094046	2.18839827485056	3.18509574340729

C	-1.19115114515619	2.89968168907073	4.32610993140664
C	-2.24613516917115	3.80571586679337	4.28622051961304
C	-2.95360205092066	3.98915526700303	3.10296882557874
C	-2.60196647352207	3.27511966063637	1.96346820209974
N	-0.63984878364779	0.31241106199251	0.99221134814176
C	-1.37481715549459	-0.80965763078906	0.93372631618525
C	-2.72319350953574	-0.86906452633325	0.56491253342921
C	-3.36134616803310	-2.08629853487909	0.32237393762899
N	-4.63679604061938	-2.07091557506597	-0.11767423998391
C	-5.30998519783129	-3.10343835583319	-0.92468102748703
C	-6.37655173797629	-2.33039441486155	-1.69605233490117
C	-6.74610892601582	-1.20980663311865	-0.72764099296350
C	-5.38805176135708	-0.80866774585809	-0.16815933072306
C	-2.60833101400391	-3.31876517374099	0.57951546957599
C	-3.12032951424738	-4.63072128052494	0.43820592226681
C	-2.33887800682804	-5.73379423724409	0.70315573728559
C	-1.02046772839476	-5.57196475879955	1.16616319418019
C	-0.50934070005866	-4.31650252183109	1.34173384770278
C	-1.29271408094670	-3.18707865366687	1.04491519207149
N	-0.72100179221572	-1.94739260600032	1.20932087158627
C	-0.27950999128012	3.87398765325061	-0.18276984560292
C	-1.17601684566567	4.26794427468597	-1.17490363405181
C	-1.53523829134346	5.60448192856907	-1.30014116538824
C	-1.00055488080766	6.55689871555953	-0.43730198112829
C	-0.10020091116464	6.16789420773096	0.54777852450920
C	0.25895123420031	4.82965487339671	0.67206875594903
N	1.34256614661576	1.04831984039144	-3.13425686844077
C	2.42172777960011	0.95272003895951	-3.98550139592498
C	2.24470613514177	0.41139380645232	-5.26852039762398
C	3.29514586907990	0.37485545748481	-6.14983407093719
N	4.99283283190459	1.83656782878096	-1.54322197090927
C	5.05054535785105	2.14236130673985	-0.10552533866949
C	6.46402986727499	1.75355923096605	0.31332589656400

C	6.78726273493833	0.60134903910896	-0.63305990398308
C	6.17972692879223	1.06143888664873	-1.95382661669508
H	5.68659966019241	1.82977876327557	-4.23305119867745
H	2.68094193444805	2.58705396388832	-0.50093428036021
H	-0.46523573368740	1.08863775013191	-1.59377979898210
H	0.93502275795176	2.31882034799541	0.64110910621944
H	-1.94596754165276	1.63225007979889	0.03094485568919
H	-0.02513533211558	1.47107313610136	3.22301798280820
H	-0.64154318613755	2.74296187211151	5.24929206992221
H	-2.52037343226302	4.36133160645398	5.17753583217873
H	-3.78314901363648	4.68876078602722	3.06691371270907
H	-3.15225037457891	3.42337282522126	1.03864180655710
H	0.37130818277841	0.16628137382439	1.02567266802283
H	-3.22488830041501	0.06019450549146	0.35327002857969
H	-5.78686056988896	-3.85192065637991	-0.28055771531754
H	-4.59717309894776	-3.60107388981137	-1.58554381881771
H	-7.22059815345580	-2.96861651856212	-1.96978664941807
H	-5.93799947747481	-1.90870247832183	-2.60434574934995
H	-7.39474229544241	-1.58857680341721	0.07074509640074
H	-7.25076516213729	-0.36869148807110	-1.20915131817340
H	-5.44010370918234	-0.36325705409639	0.83049680805265
H	-4.89374647891354	-0.10102349880456	-0.84307434604628
H	-4.13517190982597	-4.78219875715117	0.12036741811186
H	-0.42079078843654	-6.45389599438451	1.36449817689799
H	0.51246671549919	-4.17911469890464	1.68209039043273
H	0.22041607964979	-1.87805784554121	1.60832513049950
H	-1.60058438007736	3.52296582441544	-1.84247660094274
H	-2.23710404333073	5.90417531320996	-2.07254729758329
H	-1.28344731266898	7.60081613505828	-0.53560946050581
H	0.32366994790922	6.90617599758229	1.22114846386892
H	0.95563567946981	4.52345172783336	1.44788510455152
H	0.45129920516363	0.63205133096940	-3.39590759455074
H	1.26795410430633	0.03130677850062	-5.55161829453950

H	3.17406085113440	-0.03440372270307	-7.14772952870036
H	4.81933722839479	3.19629326255358	0.07598711088017
H	4.30473035283230	1.53127917076176	0.41957013271653
H	6.51057401015523	1.47155189716838	1.36765855027116
H	7.15639069268127	2.58610260708624	0.15013683620834
H	6.29391417076108	-0.31812900698667	-0.30335566518572
H	7.85712475847300	0.39728932663453	-0.72192299153780
H	5.88109167720582	0.22515120542785	-2.58538469996647
H	6.88526444318247	1.69345017084385	-2.50542176807280
O	5.03532009079798	-2.81311055537125	-0.91230008591945
S	3.89456580441709	-2.39015990338923	-1.67901890535297
N	2.62288346120113	-3.21327361848310	-1.15949543631406
C	1.28744688926254	-3.10767148880213	-1.65514528503706
C	0.67210372903530	-1.87465545384512	-1.79550656920336
C	-0.62475997339886	-1.77380249863551	-2.31848813627880
N	-1.12912638160278	-0.47493078623848	-2.43058933480445
S	-2.55918447882499	-0.07747715925941	-2.89462427495675
O	-2.87307708629934	1.24326502879334	-2.37965915467985
C	-2.35765607390828	0.22320609539256	-4.70902630756215
F	-1.94905272849610	-0.89505296900434	-5.31792180036219
F	-3.49787501654991	0.61804891399127	-5.26782871113001
F	-1.43255767942112	1.16981288319778	-4.92577753343761
O	-3.59723793130726	-1.08901396123316	-2.83612206012450
C	-1.29901921605214	-2.95418111218140	-2.65482741025306
C	-0.68047583291577	-4.18233317304186	-2.45925780229495
C	-1.41374351197134	-5.43713415801366	-2.84683051367796
F	-1.11243610363701	-5.82718633103940	-4.10190836418675
F	-1.11578767301805	-6.47183873812022	-2.04435202561776
F	-2.75264087559608	-5.28629038047018	-2.80739785428139
C	0.61679332142366	-4.28401350326624	-1.97522871326647
C	4.13796942914874	-3.03844013793452	-3.39218812069664
F	3.08963023246208	-2.70917999669263	-4.14699150408555
F	5.23375617485033	-2.49604702930506	-3.91267371120935

F	4.26207542561383	-4.35734951410168	-3.38743130183633
O	3.61967965698135	-1.00062158540975	-1.89688703824349
H	1.19513269643118	-0.98535718159940	-1.47580521182947
H	-2.30426011723635	-2.91191800951811	-3.04881995893663
H	1.10212418451187	-5.24474596978668	-1.84604842315062
N	4.32548072936853	-3.80721928712654	3.15612910689265
N	3.63265057140574	-4.12496720277812	2.24974191425974
N	2.93670703759078	-4.40937102280245	1.37444934686957
H	2.83231083366167	-3.85289685818947	-0.37645436439876
O	1.76223757507982	-1.61589797368286	2.68559665126733
N	2.52689922577549	-1.15693694596551	1.81064548304688
C	3.87763292277415	-1.31380023487634	1.89846099127316
C	4.46006376235435	-1.81378103915368	3.06617783657584
C	5.92636236164092	-1.67592820735223	3.21493329129416
C	6.46329619853095	-1.21979247457236	4.41544081489907
C	7.83686161586844	-1.05411858729727	4.55044651849511
C	8.68221699018847	-1.35758788099164	3.49075669381463
C	8.15055433351667	-1.83018586167727	2.29472045605214
C	6.78062172732861	-1.98863323369700	2.15559740292155
O	2.05815954032242	-0.53286865401646	0.82938758873905
H	4.42399857893040	-0.96853322767301	1.03356682268427
H	3.87115650154986	-1.73252360320694	3.97506407813742
H	5.80180501078324	-0.98791480266092	5.24427992364845
H	8.24647316585227	-0.69267467328618	5.48734232321434
H	9.75467882791472	-1.23654482448265	3.59747878698125
H	8.80907588443396	-2.08408303072140	1.47023322164423
H	6.36585606172963	-2.37857908452940	1.23210705539661
O	5.50484128876357	0.86604555319855	-6.74114278354915
C	6.79398917510162	1.33490341178099	-6.40650349944946
H	7.22839986741534	0.76285118683411	-5.57635489203639
H	6.78252066812630	2.40012420760815	-6.14548568064797
H	7.41080323034632	1.19540628635374	-7.29535243746123
O	-2.74531344647369	-7.01542447034141	0.54092623534129

C	-3.99503502699993	-7.23845248355694	-0.08054692075287
H	-4.80650429532094	-6.92065510394961	0.51257625897285
H	-4.07559332835775	-8.26756052526757	-0.21655790655138
H	-4.06986894922489	-6.77034954689046	-1.02621449765015

Iig$_2$/I8 TS1 (unfavored enantiomer)

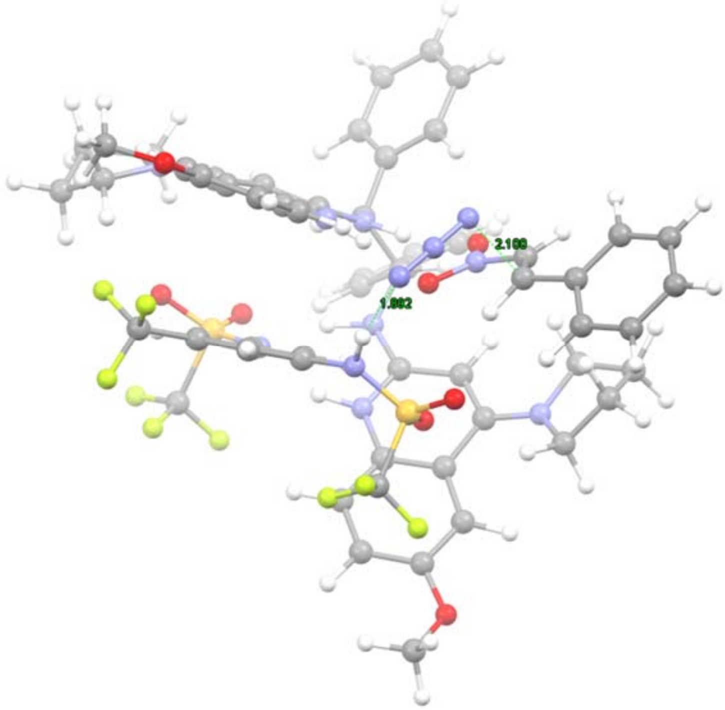

E -5239.470353

G -5238.449416

C	3.20580240772990	1.25318690027615	-5.08877777600353
C	3.54576426605614	1.73680030589264	-3.83915052180254
C	2.58087129513868	1.93179216527583	-2.84092722403709
C	2.87366305664477	2.30113112267937	-1.45478648836788
C	1.82199254878822	2.80186039879414	-0.68427375298141
C	0.49295201800215	2.59687442591556	-1.08615948045455
N	-0.58500235034379	2.88284691156512	-0.33847393708218
C	-0.59086257490981	3.23843903948532	1.06657956087595
C	-1.57986611327639	2.27678310530208	1.77833085042327
C	-1.74922079254066	2.61959447560244	3.24475675607322
C	-0.79808690250009	2.20760583933524	4.17687815146915

C	-0.91524933867044	2.57745683800148	5.51176003133441
C	-1.98645305190434	3.36083622294548	5.92738317633463
C	-2.94228246667658	3.76795136049758	5.00272823493522
C	-2.82313180427071	3.39704556050357	3.66751464663830
N	-1.09784814320651	0.92895545255836	1.56522124679844
C	-1.86534658816541	-0.15422714089290	1.37891015435705
C	-3.26603528153532	-0.14240089124813	1.40133571512000
C	-4.00714568625436	-1.26741071371700	1.04185270050518
N	-5.33932316825017	-1.15235611621451	0.88240687676400
C	-6.18940074863739	-2.01335108187684	0.04320285347511
C	-7.28509603686302	-1.07620561063421	-0.45239399655428
C	-7.44929216685207	-0.10725015395656	0.71507060155364
C	-6.00616586088839	0.12962605907147	1.14085691227078
C	-3.28838370721095	-2.53643537618871	0.88753742241432
C	-3.89847572728930	-3.80359760316343	0.76160920361136
C	-3.13908685483015	-4.95025431045724	0.63786876416472
C	-1.73629349397651	-4.87702759519202	0.66704069645175
C	-1.12570192134105	-3.66432185833234	0.81743434519053
C	-1.89214941182332	-2.49336735886402	0.93601351640542
N	-1.22669002284380	-1.30307276993456	1.11173547945699
C	-0.95776290052168	4.68639265870202	1.31112050645329
C	-1.99884629773745	5.27788441699641	0.59677336315339
C	-2.39703942221554	6.57814030956587	0.88439151786107
C	-1.75832673721152	7.29961295522106	1.88960718836358
C	-0.71475485801722	6.71608830872516	2.60002046835310
C	-0.31688933107290	5.41544363904271	2.30981190910612
N	0.24027710199768	2.00755659498411	-2.26616926275959
C	1.23689314043253	1.72510308826538	-3.17637919910152
C	0.89219433860289	1.21792840437455	-4.43220494579561
C	1.86309742380180	0.96676249754898	-5.37743493376161
N	4.09559882017142	2.15607800663154	-0.92008691338973
C	4.34120223835198	2.52239037293807	0.48047634855053
C	5.75691125094161	2.03011260324502	0.75540728396697

C	5.87058555712569	0.81224482285145	-0.15516760240840
C	5.16140696389621	1.26880954703314	-1.42270552486908
H	4.58743055449030	1.96550735396220	-3.66970410599370
H	2.00190520244317	3.21836417088966	0.29345327211303
H	-1.49417700962182	2.65791518158916	-0.74038653333093
H	0.40039965724072	3.04710079527413	1.48745107193433
H	-2.54297280215589	2.39287580798913	1.27054541856519
H	0.03967044580437	1.59227844499010	3.85905248143258
H	-0.16784795159226	2.25136701427913	6.23058408952458
H	-2.07828082728191	3.64828880119224	6.97183816883293
H	-3.78456703476064	4.37489873426492	5.32188614076579
H	-3.56704764278554	3.72158901422804	2.94342422296884
H	-0.08663807091847	0.77985605854650	1.60247518925635
H	-3.75774569541445	0.79366744411490	1.61153621135950
H	-6.62844775822358	-2.82164867823231	0.63632223866098
H	-5.60818054589203	-2.44601941310465	-0.77139737189355
H	-8.20220040026298	-1.61738763148560	-0.70469868188490
H	-6.92979563036165	-0.53875510341903	-1.33660571428476
H	-8.02021709946134	-0.57400613170495	1.52802064769833
H	-7.95092637456298	0.82477903470843	0.43797280630801
H	-5.89967954270596	0.40896095261559	2.19474493213106
H	-5.55048662847008	0.91024386559588	0.52033473363739
H	-4.97011233080580	-3.88365534512168	0.78540019127743
H	-1.15264034271718	-5.78791069619841	0.56723994411556
H	-0.04581639076007	-3.58646227939673	0.85484612940817
H	-0.21221597462031	-1.28744082203712	0.94837874555255
H	-2.50800556577926	4.71875341953458	-0.18564065616390
H	-3.21160334303538	7.02784222373464	0.32251237199806
H	-2.07179901706749	8.31574855597668	2.11656290485000
H	-0.20963399699762	7.27445063159236	3.38438589814739
H	0.49124511984736	4.95694885516172	2.87541639645459
H	-0.68447831695519	1.55419829192356	-2.37915310793349
H	-0.15511133567015	1.04558812693947	-4.66409390020741

H	1.56063751177169	0.58442875759123	-6.34572800423307
H	4.22661609288505	3.60221345265938	0.61957105531608
H	3.60982277212052	2.01608292584725	1.12768255633752
H	5.90616470636539	1.79827670311224	1.81319496063407
H	6.49166545278676	2.79076049218812	0.46430882862419
H	5.33528467554436	-0.03779925030570	0.28181928614493
H	6.90259696721172	0.50582195591909	-0.35058248150158
H	4.72559243638618	0.44046068723557	-1.98438568443206
H	5.85467530286830	1.82070912869556	-2.07020486898726
O	3.78997572976897	-3.06829443975578	-1.79821786080736
S	2.68158367740275	-2.35874556465166	-2.38841781076963
N	1.33631836001174	-3.10840751582717	-1.93409714623402
C	-0.00656780876930	-2.68432000700028	-2.16629464799911
C	-0.39868105269423	-1.36646321360052	-1.99966459016388
C	-1.73543908864492	-0.98177810685667	-2.18814563048411
N	-2.01154156362487	0.37245148632927	-1.97738081019530
S	-3.41643581989195	1.03169488647937	-1.95920462653365
O	-3.33237916703939	2.30378125516546	-1.23956104988701
C	-3.67256498399563	1.59008048409688	-3.70477573524309
F	-3.63739439327528	0.53957866265879	-4.53206063843767
F	-4.84435788898098	2.20635770691658	-3.84861125869235
F	-2.70105885272575	2.43747488441277	-4.06595278543248
O	-4.57663933438336	0.19515318257048	-1.69872304701939
C	-2.66993840525709	-1.96282279577783	-2.53036886179323
C	-2.26226156996779	-3.28545447945941	-2.65664197361255
C	-3.29126736235039	-4.32089648713649	-3.01002522273808
F	-4.49987103752006	-4.03241603761389	-2.47635771038073
F	-3.47556260827567	-4.41380717488180	-4.34422603099859
F	-2.95606398492088	-5.55033754546351	-2.57208870359349
C	-0.93718334355671	-3.66424378688554	-2.49969347654424
C	2.79459450318239	-2.68474140518978	-4.20443249918975
F	1.69797291135646	-2.25684738727940	-4.82138228493280
F	3.84936290688323	-2.03714533145539	-4.69828510961267

F	2.93722841691419	-3.98282745478795	-4.42367178025815
O	2.62007298802267	-0.92878682691049	-2.29195403987293
H	0.32343801783148	-0.62766835555848	-1.68576982488830
H	-3.71198499648659	-1.70132567309944	-2.66338307751786
H	-0.62716446931473	-4.69815300986080	-2.60158604362678
N	2.63777376630705	-3.74274872978074	2.05411007516637
N	2.36119492196938	-4.36531547793876	1.09136208536786
N	2.08484977143245	-4.96922368853265	0.14378597915389
H	1.50948631626984	-3.93402519325493	-1.33105489432118
O	1.53395804106004	-1.14345457367779	0.60837474773585
N	2.17217041388116	-0.52561070666977	1.50243315910770
C	3.35710816801188	-1.02146830041307	1.96944273946962
C	3.87064263295139	-2.17646698640533	1.39320489010929
C	5.19397175402877	-2.70753661417661	1.75806601273627
C	5.94885548409228	-3.34125144182930	0.76549743816443
C	7.21403118260636	-3.84007076345823	1.05871089338696
C	7.72607341727687	-3.72526122862825	2.34598114478023
C	6.97122799811431	-3.10529262449327	3.34157398977333
C	5.71381992695922	-2.59537525976570	3.05055728888075
O	1.70813283709457	0.52703285201304	1.99327840980748
H	3.76168207862970	-0.50160572661197	2.82811525020376
H	3.54800823958154	-2.39852011620802	0.38167217546664
H	5.53486319931103	-3.43267553465267	-0.23662488404624
H	7.79609687740369	-4.32741996086455	0.27998380362782
H	8.71116463520103	-4.12458773295508	2.58065602145087
H	7.36358788985433	-3.02697496710177	4.35096283431466
H	5.12286817428986	-2.13257101207516	3.83720694658108
O	4.22883436059699	1.13735395475578	-5.97461079045939
C	4.14839115219357	0.24372732180913	-7.07167000591326
H	4.72089052776539	-0.66361439094641	-6.85131898665958
H	4.59038579790501	0.74580402156810	-7.93078959731210
H	3.12815144635608	-0.04350273843514	-7.33669866518994
O	-3.65180156275388	-6.19227335151480	0.48857363768933

C	-5.05093422282458	-6.32011041727783	0.35813464671143
H	-5.42914739250906	-5.74930033842087	-0.50088412766472
H	-5.24817751281792	-7.38103089972873	0.19645819241942
H	-5.57534887766743	-5.99761697740585	1.26735332665536

Iig$_2$/I8 intermediate (favored enantiomer)

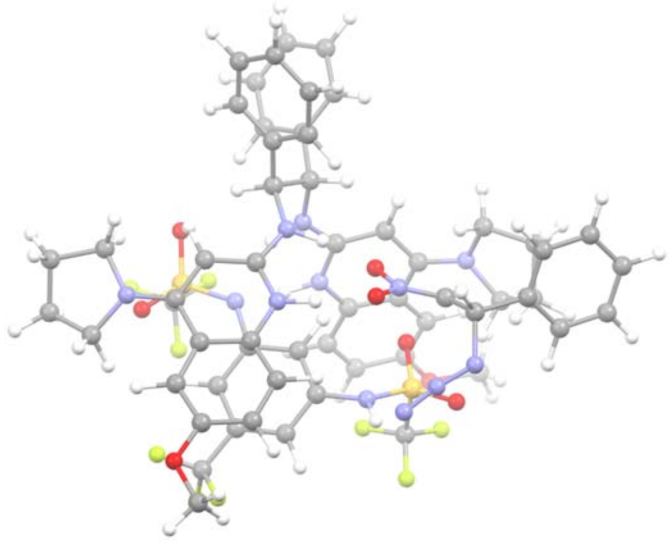

E -5239.484015

G -5238.466081

C	4.62212194175535	0.70632601656690	-5.63715013688302
C	4.81884948921655	1.21257940714265	-4.36267383236630
C	3.75727518578704	1.25576380089083	-3.42920243817469
C	3.89491197460792	1.69708387485383	-2.03524592166495
C	2.73376155231865	2.07674436981205	-1.35732120695625
C	1.48424202859239	1.62684063728657	-1.81499325979522
N	0.33769091883421	1.68800571406367	-1.12093157674142
C	0.12801609165700	2.30597149555499	0.17139152454285
C	-1.04425316333384	1.53805610210773	0.84429137794127
C	-1.48185731656319	2.20256566388076	2.13608722827718
C	-0.78086342477312	1.96692044910332	3.32215373449190
C	-1.13187127382113	2.63620266404719	4.49377144493826
C	-2.19124183983126	3.54498814935079	4.49049328030237
C	-2.90228585217036	3.77433528829131	3.31249196355397
C	-2.54956636350775	3.10329079469666	2.14203093564970

N	-0.60020834922386	0.17429920135992	1.03801373338663
C	-1.32649096634325	-0.93818218632473	0.85826329907283
C	-2.68048792271435	-0.96849854473326	0.47015359972972
C	-3.31583529021712	-2.15760492154398	0.10786875851241
N	-4.59339048983028	-2.10889414753747	-0.33282348634020
C	-5.26886102938405	-3.07920728868625	-1.21026485029270
C	-6.34304514612117	-2.25490722161419	-1.91784046948032
C	-6.70144142163260	-1.19875984650399	-0.87501532240204
C	-5.33774617077878	-0.84403958085351	-0.29518252874637
C	-2.55643537609169	-3.41227742665188	0.25745815880298
C	-3.05356341870786	-4.70569134552226	0.00733272134369
C	-2.27403028436319	-5.84130592169444	0.17906971257571
C	-0.95628941164349	-5.72042699600347	0.65273397581533
C	-0.45177419152605	-4.46619292335116	0.93390980830077
C	-1.23236781680185	-3.31623497699510	0.74123031208969
N	-0.66439571387781	-2.09469572003396	1.02393064922168
C	-0.16639426233909	3.78916578419264	0.06083248702992
C	-1.04748909879833	4.25509189601228	-0.92062360487509
C	-1.38498332583669	5.60612235033077	-0.97525680582478
C	-0.84424546473143	6.50273535131461	-0.05115078750933
C	0.03995420394168	6.04293804625409	0.92421907269878
C	0.37782442657134	4.69034077702420	0.97710369087884
N	1.40405156619455	0.99626631538302	-3.00570470251070
C	2.48148514667825	0.85882716452128	-3.85185431089374
C	2.28736230123490	0.32870762206783	-5.14183898738591
C	3.34539835695120	0.24049919674653	-6.01582889455908
N	5.08718169436128	1.70251903756010	-1.40329131049545
C	5.15807029542406	2.06250762810462	0.02124630401704
C	6.54856318249083	1.61636756190412	0.46968378192525
C	6.84905127676167	0.44452359826772	-0.46279722384044
C	6.26131817694724	0.90192470584201	-1.79393424594694
H	5.79038474228027	1.60543282095938	-4.08276982104335
H	2.77758799163608	2.49481732681264	-0.35832538398448

H	-0.41102260436275	1.08527503971020	-1.48403017941804
H	1.01860181218395	2.15813316911787	0.80323296952450
H	-1.88172686571740	1.56314085610164	0.12965927083026
H	0.04186776256840	1.24687914554129	3.32823439551339
H	-0.57681074424657	2.44514509858274	5.41578347228785
H	-2.46667442616737	4.06973441291379	5.40881717941925
H	-3.73809100551876	4.47844947168671	3.30422843810109
H	-3.10333173532279	3.28817738006861	1.21769490786538
H	0.42690323256094	0.01893497678836	1.09019142762914
H	-3.18900924763491	-0.01897893487626	0.35684632570720
H	-5.73787812391355	-3.88220161585309	-0.61678910577443
H	-4.55695991081166	-3.52768246082893	-1.91582828175378
H	-7.19640053482657	-2.87545115905651	-2.22919327536089
H	-5.90988870793787	-1.77303682499665	-2.80623099808745
H	-7.35188486225922	-1.62863585693912	-0.09501445407673
H	-7.20905812690605	-0.31956395372071	-1.29783217329983
H	-5.38680621854483	-0.46233958976377	0.73716698232756
H	-4.83401987062282	-0.09018054211500	-0.92243590795974
H	-4.06646638222971	-4.88032471324145	-0.33283842118133
H	-0.32129469600483	-6.59386272358369	0.79926355291447
H	0.56942970967464	-4.35003317557410	1.30298009015760
H	0.30713943692632	-2.06899530103377	1.41305315443219
H	-1.47718197970589	3.55098657743976	-1.63902354387464
H	-2.07663276111713	5.96213245832817	-1.74305008352067
H	-1.11084290183095	7.56181342238966	-0.09419339393609
H	0.46959665361757	6.73992762656780	1.64814329137881
H	1.06373911014452	4.32731049188057	1.74765566877742
H	0.49760959261231	0.58719387445526	-3.25321719727181
H	1.28800270803856	0.00200786717539	-5.43676782281440
H	3.21563205236059	-0.16543632193799	-7.02066858159065
H	4.98216398529418	3.14134884809378	0.15978427069328
H	4.37082995743401	1.51729909025926	0.56914044948118
H	6.56606119425183	1.33710838583582	1.53371391629394

H	7.28239658875253	2.42476322413441	0.31741784300104
H	6.32775759387922	-0.46461785553177	-0.12607014287973
H	7.92091051312347	0.21066156904020	-0.54015159073787
H	5.95553834013635	0.05722180646154	-2.42208624458341
H	6.98946977893749	1.51602595863120	-2.35164992908998
O	5.04689502032401	-3.04690884100485	-0.77778493319067
S	3.95223652684760	-2.49182432268010	-1.55110316805075
N	2.61131336701843	-3.32555360597590	-1.15703436432613
C	1.32484931355924	-3.16633268157551	-1.75570606743454
C	0.71728845027743	-1.91967797510552	-1.79681521428418
C	-0.54560268247795	-1.76216817165059	-2.39734408570178
N	-1.03965682089442	-0.45800162776089	-2.42905754530239
S	-2.47353461555531	-0.01713712330616	-2.88042620526490
O	-2.78767000072828	1.28888153878008	-2.29004143773059
C	-2.23383984558564	0.38183866344889	-4.67294680320432
F	-1.82559071323298	-0.70925836437621	-5.32743381983350
F	-3.35501600190267	0.82175533703642	-5.22970702603343
F	-1.29033355589346	1.32096776827740	-4.81148272748633
O	-3.52820329553832	-1.03573935094189	-2.90064127013243
C	-1.20085287055021	-2.90267021553840	-2.88865694393478
C	-0.59288041787388	-4.15188952994537	-2.78472952262407
C	-1.32165206535328	-5.36018721250118	-3.30728592767738
F	-1.07606121191989	-5.57324857905754	-4.61184743288399
F	-0.95902454229252	-6.48315307383742	-2.66085713027712
F	-2.65307353105245	-5.24274440309688	-3.18106604555383
C	0.68203376464595	-4.30202941306979	-2.24973216472087
C	4.28304685743884	-2.98323283806041	-3.30899328766494
F	3.26008710596708	-2.62956266966277	-4.07666330312988
F	5.38136870563252	-2.36768277016868	-3.73417952493561
F	4.45518081845252	-4.29588299962309	-3.38529782496602
O	3.68799824254864	-1.07185929852381	-1.63761112643989
H	1.20868860443031	-1.07567732294678	-1.31389322172085
H	-2.19262774165557	-2.81603499159424	-3.32572508129625

H	1.16123334037985	-5.27959664979616	-2.18990659207826
N	4.73238355956862	-3.53239657592692	2.37902627263071
N	3.84359871112952	-4.15268819999777	1.83426759192878
N	3.11064550440367	-4.85322905103260	1.32472431008499
H	2.81240197242574	-4.20840729852967	-0.68393200968004
O	1.82158975677928	-1.96216447264971	2.20306259799812
N	2.58707573154088	-1.17724275902154	1.53955809551016
C	3.89442445073612	-1.25601756994491	1.65711262965951
C	4.51684009906054	-2.10025510874999	2.71991849438526
C	5.86527871421618	-1.50301830561976	3.07296047037962
C	5.94693817060838	-0.50629567101792	4.04943652679902
C	7.16238433702852	0.12145332512687	4.32115056226758
C	8.30990834676800	-0.24127362491164	3.61427416313450
C	8.23347995409855	-1.23598977908418	2.63874529042568
C	7.01696932912778	-1.86343324486696	2.36859545013270
O	2.05558276823651	-0.30702399724420	0.76802721282491
H	4.46994660115020	-0.57782760793669	1.03702466455337
H	3.85852692770064	-2.07883790966703	3.60393756338807
H	5.04778496516165	-0.21973906239578	4.60213459599948
H	7.21422751746240	0.89498097580585	5.09170221630817
H	9.26375479337681	0.24799355476574	3.82722502767190
H	9.12827788828353	-1.52709746567254	2.08238775278345
H	6.95318812252358	-2.64209538051244	1.60539060388239
O	5.58630191736371	0.61948109369962	-6.58445600193212
C	6.89144555536930	1.02319642600606	-6.24781721328856
H	7.51545925168709	0.83626172695741	-7.13326926913978
H	7.29243274524360	0.43978399716388	-5.39835887198468
H	6.93841490308794	2.09997433875180	-6.00198828085897
O	-2.86631553276775	-7.02099352984908	-0.13063212106565
C	-2.10778151335639	-8.20247707770578	-0.03103176124870
H	-1.79308493075155	-8.40294230385845	1.00964877462566
H	-1.21670095980524	-8.17542934482281	-0.68221186325974
H	-2.76270391556791	-9.02074522240994	-0.36326861854608

Iig$_2$/I8 intermediate (unfavored enantiomer)

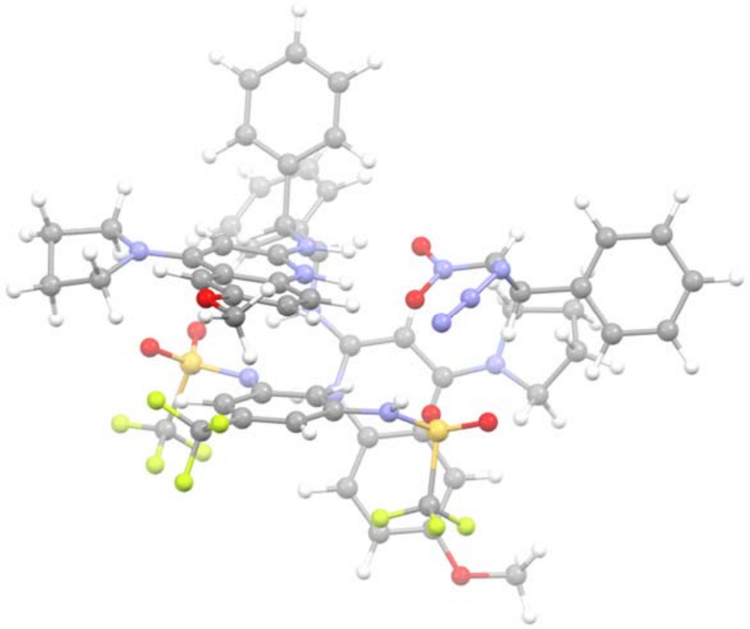

E -5239.481060

G -5238.462749

C	3.35378752321144	0.92876991834252	-5.08777296176196
C	3.71140592333941	1.38219136020832	-3.83199336182626
C	2.74998574697932	1.58398067380381	-2.82819886716207
C	3.04589562992725	1.99147956410999	-1.46561913000780
C	1.99134824038008	2.52588484203069	-0.72196041263241
C	0.66589691305495	2.36481834735091	-1.12137110915333
N	-0.40271501279948	2.79806814943823	-0.43626553342974
C	-0.45645400066280	3.12395880180927	0.97238978808136
C	-1.50987634192184	2.19240292561371	1.62672320576218
C	-1.66004732690074	2.51986929093361	3.09389096517023
C	-0.69027486769479	2.11741899789582	4.00464419620485
C	-0.80921468886934	2.45644018524887	5.34103439743625
C	-1.88990467471036	3.20502169490125	5.77714698887079
C	-2.85624169071927	3.61079690057568	4.87236934557009
C	-2.74286255147203	3.26767690476377	3.53646487837347
N	-1.06952694187859	0.82628092466921	1.39769629912180
C	-1.88857017230695	-0.23223478081334	1.23578623551148
C	-3.29212830728203	-0.14190782128521	1.24099762698002

C	-4.11727594073738	-1.23225157008996	0.97025000943353
N	-5.44609773165872	-1.05279650164632	0.88208003269681
C	-6.38997406814685	-1.90414784599523	0.17196047267505
C	-7.45056125515345	-0.93443118082469	-0.34259769338055
C	-7.52203734594957	0.07070474116005	0.79914345119298
C	-6.04371038470280	0.25818007480587	1.11960686069075
C	-3.47594623959330	-2.53746313814922	0.87915911849586
C	-4.14532392843650	-3.76494168535759	0.84918565549692
C	-3.47497312975418	-4.97813834823767	0.76735620209874
C	-2.07807963488519	-4.99391933029357	0.72858783298254
C	-1.39387578099922	-3.80501862858164	0.81384388209068
C	-2.06424620642134	-2.58528620132450	0.90331264061971
N	-1.31037650159463	-1.44447311700841	1.04840255345251
C	-0.84128766849395	4.56612803488381	1.20817061488214
C	-1.88152993015589	5.14153548540548	0.48746469061851
C	-2.26242363590993	6.44750217179330	0.74269591607857
C	-1.61789788523380	7.18474240266764	1.72247635826664
C	-0.58605107302694	6.61267799184864	2.44672275096793
C	-0.19835230856046	5.30932516728673	2.18890418829885
N	0.40781621753216	1.73931223534583	-2.28677947177419
C	1.39611608822260	1.40927421584150	-3.17209173719028
C	1.04243042811646	0.91805624269836	-4.43414810729873
C	2.00354237456790	0.66771215676718	-5.37509777515922
N	4.27303295441079	1.90180318272362	-0.92371808786469
C	4.51378593784400	2.32784232640698	0.45258581327214
C	5.93389193911297	1.86454291350597	0.75904099343846
C	6.02853530909865	0.58641979744676	-0.06509076483018
C	5.32454957699868	0.99194054820638	-1.35814692872020
H	4.73778058878871	1.62576716055975	-3.63229854132045
H	2.17529523480227	2.99049964769294	0.22773355762008
H	-1.30859032564565	2.57593992702629	-0.84468736298274
H	0.51063297892936	2.91054916305895	1.43590427152699
H	-2.45550506786162	2.36068823969590	1.09984143710752

H	0.15706078978235	1.54017728181668	3.66482786110112
H	-0.05273526129583	2.13859776664667	6.04374419866515
H	-1.97813971961472	3.47181263760593	6.82002011198541
H	-3.70022750089049	4.19566897595393	5.20693577141764
H	-3.49390082256753	3.59052243823898	2.82945998537681
H	-0.04504056392062	0.64537414691848	1.49220247857443
H	-3.72267776328694	0.82725806204926	1.40025701514365
H	-6.85765801902162	-2.61101487514654	0.86815218287847
H	-5.89995892352885	-2.44494846881956	-0.63642074005478
H	-8.39612314221585	-1.42991601812837	-0.55101420372752
H	-7.08718426642401	-0.44407773425659	-1.24588693617897
H	-8.05183491992704	-0.35817847710808	1.65128022281402
H	-7.99994111253758	1.00621873527167	0.51830217400804
H	-5.86557814540520	0.56886648462917	2.15235005801662
H	-5.61179537017973	0.99268449960355	0.43112490144915
H	-5.21368823332424	-3.83021432464881	0.91974944619683
H	-1.52353295686314	-5.91502404631766	0.65692550090808
H	-0.31468707119177	-3.80124682026609	0.82440631994299
H	-0.26460283181864	-1.51859821606398	1.00672703825769
H	-2.39596062216326	4.56629557331558	-0.26848437861603
H	-3.07027314950858	6.88921047664038	0.17771592339562
H	-1.92097340424330	8.20196865922784	1.92227249890942
H	-0.08202847668475	7.18127314755437	3.21417345854795
H	0.60232047180088	4.86136903148728	2.75989210236013
H	-0.54505778265656	1.38026979516069	-2.43240129095177
H	-0.00139829056121	0.75531401075062	-4.65299822171157
H	1.74957752836530	0.29188754293040	-6.35307156030225
H	4.39915482616654	3.41126932949821	0.53570354177238
H	3.80047631896410	1.83840577715099	1.12561626100118
H	6.08972502833413	1.69508986037935	1.82180866431207
H	6.66128006265959	2.59746065174287	0.40766675232597
H	5.47653760518221	-0.21523892622861	0.42532809757383
H	7.05058542171480	0.25573379982868	-0.23516895379465

H	4.89683368014625	0.13725996169403	-1.88516609914739
H	6.03690817443801	1.51833434587932	-2.00438611195598
O	3.62443205338890	-3.35676561485102	-1.58493957857113
S	2.53377575354540	-2.54299536477648	-2.12864429819537
N	1.14671023369768	-3.31239594015477	-1.75173473773136
C	-0.16513366221327	-2.83557028861518	-2.00324732564497
C	-0.45386119989653	-1.49172169988825	-1.92471667691504
C	-1.76215675516753	-1.02509236827654	-2.13659327096341
N	-1.96584311517484	0.31443512536376	-2.01914621876814
S	-3.35306345746089	1.02272937214682	-2.00638535060490
O	-3.18426931098052	2.31097280811590	-1.37479967344314
C	-3.61640678525257	1.50039504329967	-3.82524642286211
F	-3.85992041040249	0.41542607578209	-4.57190088926496
F	-4.63638159276893	2.34616279153289	-3.99202398954552
F	-2.51612832427953	2.08412323553417	-4.32021117276207
O	-4.54548416969545	0.29259913791442	-1.67503052013314
C	-2.76508099694532	-1.95530126548144	-2.43022761695965
C	-2.44964827383713	-3.30204739666447	-2.49802860802254
C	-3.53524681874563	-4.28177570554500	-2.83026453749502
F	-4.72920152571329	-3.94811775160977	-2.31357513268173
F	-3.73655666148642	-4.39995179445221	-4.16184668368478
F	-3.27963788575469	-5.52798835515718	-2.39615363716164
C	-1.15726608896854	-3.76574717798398	-2.30164511729728
C	2.63868032624007	-2.78245561372510	-3.98136882785533
F	1.55896672436672	-2.27339970141846	-4.56958356186414
F	3.72285397849971	-2.15610787073277	-4.43414350097231
F	2.72893211983060	-4.07625966882379	-4.27286248922428
O	2.49988382047861	-1.09807688967555	-1.96536615399008
H	0.32064152614322	-0.79786571387692	-1.65354887418555
H	-3.77897469386734	-1.62239250994250	-2.57877038858072
H	-0.92845473764010	-4.81762189729801	-2.35448498165208
N	3.21140990144510	-3.54403113720993	2.28406938204961
N	2.50021924800421	-4.19144214977273	1.54449683906099

N	1.81315656965440	-4.86392071226778	0.91213241780893
H	1.26729875212163	-4.22644538471175	-1.29849169213196
O	1.51614840260139	-1.52241310831727	0.88369160887372
N	2.18440898996830	-0.71024009491009	1.62074999370495
C	3.40301267120549	-1.06003962999342	2.04455711397971
C	3.95777263913858	-2.36785996222454	1.63276523991036
C	5.41438186472908	-2.52013815693357	2.00466695166277
C	6.35211438300363	-2.80458864423688	1.02080874627455
C	7.69027872680294	-2.93218688093881	1.35265546742223
C	8.09686160662186	-2.78896178973709	2.66873136264685
C	7.16077000807909	-2.52468289134197	3.65534677006567
C	5.82370157734682	-2.39135904878670	3.32576612168464
O	1.69407350573554	0.42645893801499	1.93626272180390
H	3.88249076394287	-0.37486727832081	2.74515659367378
H	3.83746521499010	-2.50602761912561	0.54263586268951
H	6.02662709486631	-2.92817886183800	-0.00331657281542
H	8.41513789599844	-3.15066024662387	0.58209921258318
H	9.14061281000500	-2.89172893092265	2.92682233729389
H	7.47294704869197	-2.42480951139920	4.68465631912957
H	5.08758580541732	-2.19933045876533	4.09230683142661
O	4.21973000508793	0.70084381449162	-6.10874343862008
C	5.60101693677787	0.88722684611370	-5.88887582589834
H	5.83110000980529	1.93154532466071	-5.65530107370017
H	5.96706896352543	0.23629487945608	-5.08883262193692
H	6.08342452467719	0.61437280198964	-6.82600968812969
O	-4.26742388369033	-6.08193538139829	0.74655436826021
C	-3.66035194330724	-7.34771861797153	0.59882650459249
H	-2.99237192633107	-7.57208467961290	1.43631885624024
H	-3.10920816441172	-7.41505582056991	-0.34370305263241
H	-4.48056846834350	-8.06356985544322	0.58954929397355

Iig$_2$/I8 TS2 (favored enantiomer)

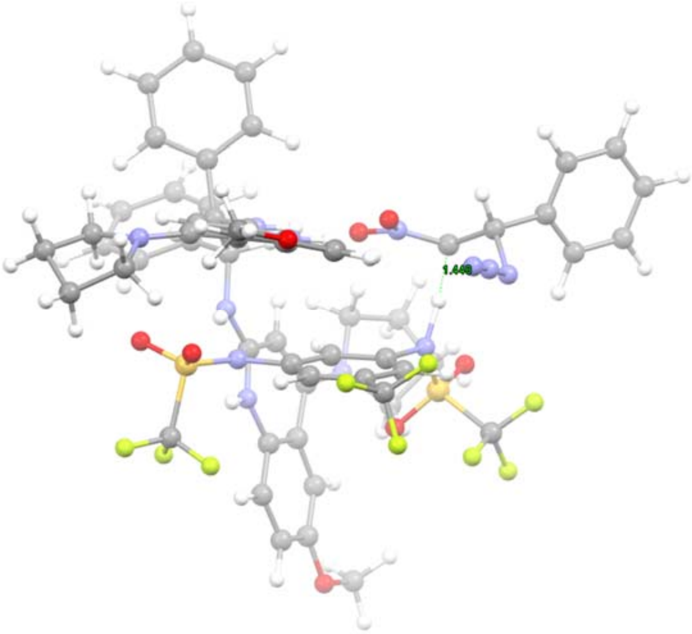

E -5239.476937

G -5238.452794

C	3.18453600115248	1.95530398781129	-5.75483999448686
C	3.37059199836009	2.53926799342531	-4.52347999682110
C	2.41086299730470	2.38716099580392	-3.50419599752654
C	2.56664499481444	2.89670700170439	-2.14507999953617
C	1.42909999369623	2.96313700246291	-1.34275000109706
C	0.27576599526822	2.25828799890816	-1.68944999982669
N	-0.77709600526652	2.08524299951659	-0.87234000046206
C	-0.82119100580115	2.59634400250503	0.48837399824235
C	-1.96266000470700	1.85592700234272	1.22044599940979
C	-2.23156800312276	2.46927000361423	2.57875999914927
C	-1.38421500163612	2.21007400495529	3.65008299829780
C	-1.60101700022827	2.81921000610558	4.87678399797543
C	-2.66837700029542	3.69139000592233	5.04327299850543
C	-3.52187800177316	3.94560300458310	3.97945099937091
C	-3.30331200318000	3.33406500343361	2.75294299969334
N	-1.60366700377286	0.45350300272060	1.29087199793630
C	-2.47549800445664	-0.56469199672874	1.20787099862516
C	-3.84605100458748	-0.40192699540962	1.00630100167720

C	-4.67110000565354	-1.49061499435579	0.74460000124843
N	-5.92447500607833	-1.28382599229396	0.30668300369423
C	-6.71917100859451	-2.23703198985385	-0.49259699669770
C	-7.50134900896493	-1.34431398711307	-1.44492799382574
C	-7.75012300592661	-0.09867498825050	-0.60296099124907
C	-6.41111200452586	0.08809300872684	0.09273300689222
C	-4.12831200620414	-2.82284399514685	0.96894399774653
C	-4.90496800674481	-3.99868499466380	1.03073099662476
C	-4.31064500730840	-5.22378499536955	1.18735799289658
C	-2.91258500697918	-5.31289399651093	1.27974399033395
C	-2.14928300639628	-4.17960499699276	1.29566399134440
C	-2.75429600595065	-2.92630499645374	1.17359499501270
N	-1.96307100525782	-1.80163299712948	1.24984599548855
C	-1.02583800761037	4.09557300235092	0.53606199775565
C	-1.96655800879535	4.72343800052192	-0.27557000230496
C	-2.18603101094184	6.09160000021578	-0.16125700298601
C	-1.46097901197918	6.84238200183855	0.75765899646167
C	-0.51063601081692	6.22028500380695	1.55990799659170
C	-0.29793300855569	4.85176400400415	1.44740799723135
N	0.21790199774826	1.63166999427428	-2.87515899737610
C	1.22751799887986	1.71794099232199	-3.80707899629918
C	1.04789400176580	1.11544898671636	-5.05926199388113
C	2.01647200293242	1.22296498445313	-6.01567099282077
N	3.75172399377173	3.28970800634376	-1.65186800025282
C	3.84616999178789	3.79871101252789	-0.27366500243175
C	5.33966199128117	3.93076401718413	-0.02422800193857
C	5.90595599461229	2.77439501500284	-0.84316699636955
C	5.07619599528374	2.83089100738245	-2.12072899720970
H	4.24894299700335	3.12454599586068	-4.33948099809110
H	1.46974399172194	3.40691300634401	-0.36761600278103
H	-1.37659700387152	1.29174799771672	-1.10695199844172
H	0.11016399463406	2.35402500336639	1.00523399786196
H	-2.85053200595264	1.96658600194508	0.60166800112602

H	-0.55112500157570	1.53098400510437	3.52743099788275
H	-0.93569099907808	2.61279200714564	5.70359799730954
H	-2.83689099919746	4.16574100681929	5.99994699825409
H	-4.35776100183362	4.61952200443063	4.10236699979566
H	-3.96407100433857	3.53871100238699	1.92081800035593
H	-0.63326300334610	0.25136400149109	1.52240799449617
H	-4.22652900407275	0.59428600498475	0.92071600397937
H	-7.40597300825609	-2.79326199010879	0.14593000344423
H	-6.06731601036377	-2.92850898975430	-1.01253599904825
H	-8.41092101016866	-1.82531898529314	-1.79524999319924
H	-6.88284401004144	-1.09872098642365	-2.30708399440163
H	-8.53832000498823	-0.28564698844923	0.12663200971376
H	-8.02356300569941	0.77368501313802	-1.18940998928955
H	-6.48689900224110	0.61627900719353	1.04131200792854
H	-5.71496400464173	0.62263700876588	-0.55576699345194
H	-5.97321100678508	-3.93363299387821	0.98127199852788
H	-2.45412700696956	-6.28649199671616	1.33864198740329
H	-1.07206600624305	-4.24776699780835	1.38713598915402
H	-0.97391700487768	-1.91286299782131	1.46444399289557
H	-2.52826600808398	4.14110499927403	-0.99582000185081
H	-2.92353101186645	6.57356499870137	-0.79239100306205
H	-1.63197001371046	7.90821300160050	0.84355099596898
H	0.06723398825047	6.79740300525432	2.27026399621906
H	0.43758599232727	4.36185400550457	2.07646599736749
H	-0.57065500095349	1.01801599197359	-3.04510799575950
H	0.13836300309432	0.56699698414428	-5.25535899284932
H	1.89536000517368	0.75445698007256	-6.98181499097748
H	3.30321898971823	4.73747201168791	-0.17457900700504
H	3.40803599288333	3.07367401459043	0.41625500043143
H	5.57832799071847	3.86980502246319	1.03543299849186
H	5.70693798922914	4.88434601637971	-0.40474900593507
H	5.73003799661877	1.82491201701615	-0.33932199222917
H	6.96846199452625	2.86708701664619	-1.04943699607425

H	4.99023099787714	1.86411000507812	-2.60796599309497
H	5.50579599413179	3.55173900539408	-2.81843799997330
O	4.76014401446724	-0.45674899167213	-0.64419898231491
S	3.78025301523893	-1.12592699052881	-1.43145298396514
N	2.69338501573322	-1.72914099311660	-0.47512298567886
C	1.41725801508822	-2.13839299379015	-0.99183798735847
C	0.44070100915723	-1.19354099788424	-1.27481498847918
C	-0.82518498868867	-1.55747400462529	-1.73800198893835
N	-1.70417699395559	-0.49158800878720	-1.97448898911881
S	-3.17895499342711	-0.59753401654512	-2.42840598933282
O	-3.85003600026715	0.65161797984039	-2.17295098918620
C	-3.09255899305659	-0.63682401608869	-4.26753498932516
F	-2.42521798725456	-1.71556501252324	-4.68693098926379
F	-4.31024099286854	-0.66311002259828	-4.81252798943137
F	-2.45340499881401	0.44799598732598	-4.73156998927251
O	-3.88488598694415	-1.81624202030186	-2.11573798933874
C	-1.09736998199643	-2.92055900577629	-1.89600498896570
C	-0.11491997766786	-3.85254000080919	-1.60481298856057
C	-0.40432497059849	-5.31502000217411	-1.79107298858751
F	0.09748803211085	-5.78151799991075	-2.95538898832665
F	0.14433903259132	-6.06643499935031	-0.81606198820435
F	-1.72026496927407	-5.59071000847447	-1.80333498906882
C	1.15021302041516	-3.48743899491352	-1.16528098799768
C	4.66220601645978	-2.60348398856636	-2.09034298673178
F	3.82444901718958	-3.36945098771900	-2.77667298856828
F	5.66265301646656	-2.23913898622331	-2.90146098567094
F	5.17502001660769	-3.32968399030895	-1.08400998806466
O	3.25305501509666	-0.49592898837243	-2.60471098314673
H	0.66914000067826	-0.15144599603126	-1.13413598979902
H	-2.06889598028305	-3.24807401053209	-2.22614798928979
H	1.91022802346367	-4.22448499169661	-0.95940198773496
N	3.23992400226629	-3.64706801399809	1.81446097978235
N	2.23475200012238	-4.32667201122724	1.82961398105131

N	1.33607099802492	-4.99617300887739	1.79970198256988
H	2.83967701529055	-1.52572099566874	0.71337001564258
O	0.67640399880604	-1.74709400199018	2.34300898665737
N	1.54694599778894	-0.87341100163349	2.14241398393649
C	2.87475600594465	-1.23057500489031	2.12236197332583
C	3.21504300319024	-2.52446800867317	2.80601197518804
C	4.56320800380310	-2.50343301400407	3.48625597801557
C	4.62874899894876	-2.69465001289365	4.85726397885688
C	5.85278999640800	-2.77640401878606	5.50585998423076
C	7.01983200069576	-2.66287402594814	4.77546398964757
C	6.96027100613099	-2.44581902689209	3.40385398852932
C	5.73761000816298	-2.36942602097997	2.75599098292205
O	1.21959399747698	0.28920999948008	1.83279398838866
H	3.52010200662745	-0.37572000537091	2.21670497301007
H	2.46100300151216	-2.75483600465113	3.54806897473148
H	3.71703999640629	-2.80589900761094	5.42464297580725
H	5.87886999070540	-2.95103801786368	6.57410398452078
H	7.97850199951732	-2.74528203090878	5.27445499494573
H	7.86879900868602	-2.35633903232665	2.82839099171811
H	5.70420801352707	-2.22576502203266	1.68433998261374
O	4.06796300238241	2.02652498505863	-6.78311599362084
C	5.28798700078028	2.69697198842864	-6.54456399489844
H	5.12947999846413	3.75430798906652	-6.32676699968076
H	5.83948000057149	2.23731399303188	-5.72441899217811
H	5.86381800217588	2.60413698557052	-7.46086499373183
O	-4.98697200892234	-6.40139899452343	1.26217899107692
C	-6.39924300902150	-6.34971499083656	1.17019999292966
H	-6.71783500899665	-5.91721598764944	0.22407499437821
H	-6.75318901184952	-7.37625298993674	1.22935299062801
H	-6.82900200659366	-5.77925799174414	1.99222099482878

Iig$_2$/I8 TS2 (unfavored enantiomer)

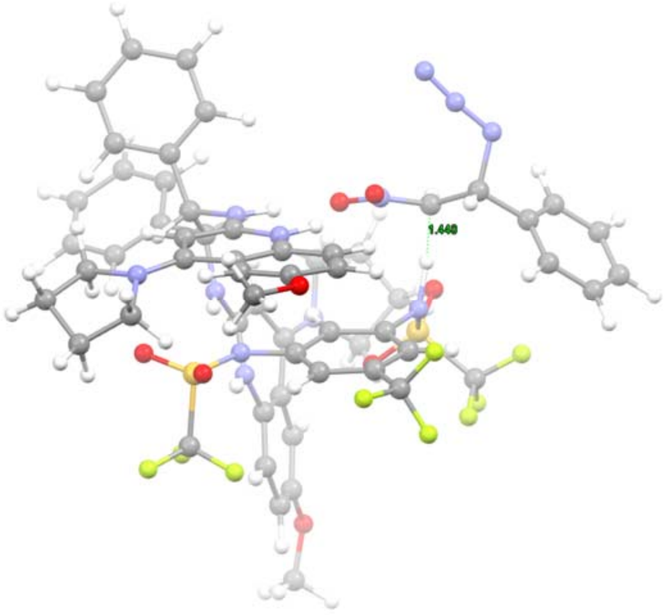

E -5239.471358

G -5238.451490

C	3.25242756686892	1.90021591933132	-5.75559982480253
C	3.38980180416130	2.54144019091400	-4.53977873342995
C	2.51200736234611	2.29512701482699	-3.48149826045050
C	2.64094151062100	2.88148509120525	-2.15074263124266
C	1.50690820580596	2.90155870529250	-1.34363741686043
C	0.41835662133440	2.07946198343118	-1.64072464301859
N	-0.64329948211407	1.92398180086795	-0.83517295036386
C	-0.82029457527389	2.63196255655730	0.41804988968766
C	-2.01288616089495	1.98457386363340	1.16084790894893
C	-2.41410707168032	2.80920659335332	2.36642069375895
C	-1.67301606466475	2.74625007682607	3.53995122146331
C	-1.99356825590206	3.55999667032165	4.61544102367284
C	-3.05910157635705	4.44502231988103	4.52592540449536
C	-3.80928512537143	4.50289557330756	3.36080615082901
C	-3.48789516182263	3.68570621340894	2.28723809441572
N	-1.66526217972744	0.61924415072215	1.50897681188535
C	-2.50763331788787	-0.42480759335529	1.44003220433838
C	-3.85374078459967	-0.32071313965306	1.08218338045276

C	-4.63643276348917	-1.44845434997296	0.85253175999580
N	-5.85225815593663	-1.30485133149405	0.29646345892087
C	-6.57038598965728	-2.33019586772430	-0.48155163336570
C	-7.30137893786176	-1.52801565434024	-1.54974609358889
C	-7.63434909370750	-0.23334279638746	-0.81831726621440
C	-6.34851281858029	0.03496388273620	-0.05229274119755
C	-4.09042211197019	-2.74548410606100	1.23828416871275
C	-4.82719224661084	-3.94772094140827	1.29687967989606
C	-4.21511259376113	-5.13310424758488	1.62890999992639
C	-2.84338816753800	-5.16037772158921	1.92779050422104
C	-2.12903624841818	-3.99903549730368	1.94255814303007
C	-2.75018644530885	-2.78413947141248	1.61879034967235
N	-1.99314122017785	-1.63794727937372	1.68415813811098
C	-1.06990825774172	4.11150332152399	0.20020439378516
C	-1.92023915080896	4.53764516930463	-0.81246261031402
C	-2.20214079518081	5.88602841499457	-0.96815027975196
C	-1.63686936729066	6.81975013655133	-0.11090893138386
C	-0.78256945182971	6.39898602176000	0.89784450480430
C	-0.49820799032936	5.05004886686592	1.04858517417591
N	0.43528652610311	1.32868714381254	-2.75545742608847
C	1.42007376202084	1.45291626884378	-3.71141710359227
C	1.32356564392922	0.75004174973459	-4.90997770273680
C	2.23724865091758	0.95219996060826	-5.91948087209491
N	3.80347136427136	3.36788859923693	-1.69991972500136
C	3.89703840220961	3.93084550968259	-0.34588668694791
C	5.38972527919069	4.13404080807996	-0.12833172363810
C	5.98971435776256	2.98075311434513	-0.92652758035817
C	5.14102820136840	2.98652037740860	-2.19106408118436
H	4.18418064585702	3.26062014402389	-4.44553868259495
H	1.50479011183767	3.42165130663255	-0.40555375985654
H	-1.23305189380545	1.11759760942357	-1.04232472841233
H	0.06621619652897	2.51269249036694	1.04852096003054
H	-2.84227389271028	1.96961217917196	0.45492198050963

H	-0.83967942193103	2.06149031102202	3.61440172300854
H	-1.40914593472010	3.50505786356300	5.52293485166000
H	-3.30647814674564	5.08240238769029	5.36302946930980
H	-4.64251110290774	5.18720728670807	3.28535241973790
H	-4.06568334077980	3.74057691298043	1.37444936674025
H	-0.69991369710065	0.42979387985728	1.77921103487528
H	-4.24185230950280	0.65366773795892	0.86635510958088
H	-7.29181787056150	-2.85274330540353	0.14867290924738
H	-5.86989392065621	-3.04143000027821	-0.90378445462688
H	-8.17401930286207	-2.05479163016623	-1.92770856938375
H	-6.62750749780473	-1.32652518915093	-2.38113372287985
H	-8.46499080384157	-0.38911721433102	-0.13008265930675
H	-7.88633708792941	0.58767660346062	-1.48375656509079
H	-6.49694914549158	0.63104838520608	0.84707518630971
H	-5.62176818563063	0.53382842464499	-0.69571805483810
H	-5.88259369651626	-3.93546567282302	1.11012241471849
H	-2.37203321040564	-6.10578658405445	2.15163665841231
H	-1.07903214775851	-4.00129282295579	2.19691951304706
H	-1.03918612207196	-1.74415949701655	2.03310174496893
H	-2.36357998146100	3.81115428167071	-1.47984907281128
H	-2.86493502034493	6.20829808763329	-1.75892170088858
H	-1.85846129251532	7.87068244469662	-0.23125154689179
H	-0.33997319433787	7.12123433979742	1.56917137725956
H	0.16248043315122	4.72118267955927	1.83968369648071
H	-0.29801958893149	0.63997425312215	-2.87494422571959
H	0.51947951595026	0.04188595914869	-5.04273628390101
H	2.14070309016782	0.37748468778985	-6.82621701922923
H	3.31652853151913	4.84930269579156	-0.27492959088274
H	3.50019159930163	3.21191958539719	0.37513188491211
H	5.64890366182009	4.11201796668038	0.92678004363613
H	5.70887702521022	5.09031498920000	-0.54355117229943
H	5.84450224935685	2.04027310406412	-0.39536827195663
H	7.04646344327134	3.10669539283989	-1.14707502947019

H	5.10199914149842	2.01843777535967	-2.68011699156631
H	5.51619645933919	3.72812941566017	-2.89696669197916
O	4.53816127162896	-0.03057205322279	0.19930863735256
S	3.73671742763992	-0.80579926895863	-0.71121003742628
N	2.79716166619074	-1.77423275979738	0.11079960579403
C	1.54592319368977	-2.23300900676305	-0.40984956346105
C	0.55880510641657	-1.33047491894070	-0.76801045464169
C	-0.67197871091422	-1.74904831100927	-1.27545270920427
N	-1.54997649105458	-0.72742751269981	-1.64688321254132
S	-2.97007514813897	-0.90100071490018	-2.23351895593085
O	-3.67583412905198	0.35392136152577	-2.15392466919157
C	-2.71232635986755	-1.08897376252696	-4.05855426687341
F	-2.01101856722774	-2.19588076814344	-4.33422404406509
F	-3.87772129638417	-1.16121446836708	-4.70739998242773
F	-2.03170400531552	-0.04164394488763	-4.55486168170319
O	-3.69212984151713	-2.10367265680719	-1.89595994512073
C	-0.91967753954671	-3.12356440488768	-1.34525657423301
C	0.05598773019631	-4.01317206755351	-0.93054887469352
C	-0.22059025285779	-5.48915800079029	-0.96732135178650
F	0.51100113341185	-6.11899662909635	-1.91021485017841
F	0.08613979217397	-6.08698256389689	0.20416246981293
F	-1.50521779759165	-5.78049579925174	-1.22118768230001
C	1.30250218800554	-3.59491494761800	-0.48248146298586
C	4.94559263045316	-1.94970839485662	-1.50903980177821
F	4.29570880893251	-2.85229433409957	-2.24926739047419
F	5.76281010560755	-1.25665720554027	-2.30884462446037
F	5.68057260394246	-2.58914597672317	-0.60452347731038
O	3.10668038229678	-0.16664978479348	-1.83193555407528
H	0.75244122878125	-0.28039976211923	-0.64825124057687
H	-1.87180838860074	-3.48794140582143	-1.68952182134607
H	2.06375271804408	-4.30158930610268	-0.18998864048403
N	3.29377347558455	-2.26606566343171	4.94497453318797
N	2.32652591939388	-1.76867863674527	5.50095914001971

N	1.50500484789280	-1.30547998306578	6.10877010380158
H	2.83302484207069	-1.57411967705201	1.29271818512084
O	0.54658140485274	-1.75201404501094	2.97736011717223
N	1.42941944831905	-0.91472556973094	2.67511684476019
C	2.75392026140672	-1.28047705753494	2.70023955051063
C	3.13068924024028	-2.50860645594451	3.47589129677539
C	4.44366863484561	-3.06943115771058	2.98574267807185
C	4.45273733268024	-4.23449901600725	2.23346650334849
C	5.64580585223792	-4.75940531326123	1.76281279908184
C	6.84105970710369	-4.11733326944676	2.04018362596172
C	6.83736062452172	-2.94897462405511	2.78734242577564
C	5.64430984107330	-2.42725367817920	3.25850142996858
O	1.10152420323392	0.18165219392670	2.18251004290190
H	3.41895254730658	-0.42922031252631	2.71747299362872
H	2.35753981964821	-3.26124669070576	3.33885041476093
H	3.52042765088175	-4.73870919558276	2.02214879214220
H	5.63928904681353	-5.66887233187815	1.18076588389986
H	7.77240624241523	-4.52410571273033	1.67517157610770
H	7.76672828891096	-2.44436452305829	3.00629616210804
H	5.64565028933520	-1.52518885354231	3.85239640840378
O	4.17121556596856	2.24298030658466	-6.69797427119477
C	4.01049771555238	1.86941306689280	-8.06016861302403
H	3.94194623651384	2.77092786893747	-8.67151081792606
H	4.88665671814434	1.31108763063131	-8.37417134477527
H	3.13572605002266	1.27029497620545	-8.24499415513765
O	-4.84416502078800	-6.33345072729077	1.70058940306167
C	-6.22212875012274	-6.38298144665437	1.37098055547130
H	-6.40651084364253	-6.01629503611128	0.36621072221092
H	-6.50352428052265	-7.42453175690576	1.42302948559152
H	-6.82874414213912	-5.82136182001747	2.08182080909079

Iig$_2$/I8 product (favored enantiomer)

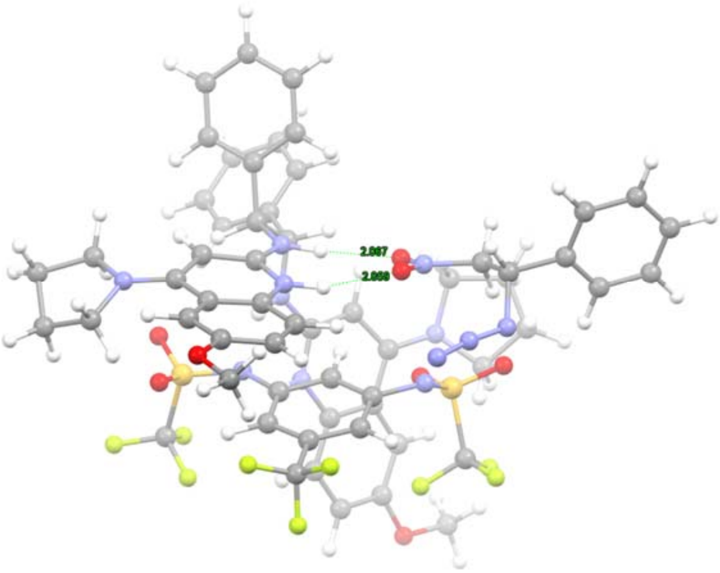

E -5239.504639

G -5238.479780

C	4.24181511299440	1.00918791906029	-6.07518698404197
C	4.43386435126159	1.62689307322869	-4.86216146619105
C	3.45109599183263	1.55225122279674	-3.85547247186010
C	3.59724556661901	2.12968940679294	-2.52406947065146
C	2.44773148898516	2.29861023410858	-1.75732971118186
C	1.28451493190835	1.60649188532439	-2.08926035029278
N	0.20423189329925	1.51646270948771	-1.29433672081279
C	0.11165954715819	2.07159962311169	0.03980149350785
C	-1.05731504230166	1.34122598070505	0.74157730718597
C	-1.41601378080822	1.97831865956933	2.06678078329635
C	-0.66064739595825	1.72531267671679	3.20517518553146
C	-0.95729386619088	2.36400765193857	4.39971985711377
C	-2.01557117390003	3.25991687154506	4.46653816424289
C	-2.77958234339234	3.50765571394150	3.33543305347082
C	-2.48056794114631	2.86683140132540	2.14218703505239
N	-0.70070142313252	-0.06130008812198	0.86298301608050
C	-1.56838146763343	-1.08597704018981	0.78151613505277
C	-2.93927366936760	-0.92307308764954	0.59312579753905
C	-3.75984907832490	-2.00725446338322	0.28939382427320

N	-5.01547901446167	-1.78627149319282	-0.12113492677059
C	-5.83676591774584	-2.69907754423879	-0.94089716448412
C	-6.67527599480834	-1.75869810017877	-1.79561761700500
C	-6.89366083734086	-0.56866112722747	-0.86795129165786
C	-5.52515114356451	-0.41003888219604	-0.22500280078062
C	-3.19984608872258	-3.35034852517189	0.44837738850992
C	-3.94436030190391	-4.53604222415815	0.42022227172339
C	-3.34276860194953	-5.76892568359847	0.56434020606269
C	-1.96102935350105	-5.85273875746988	0.76247118496703
C	-1.21876970095846	-4.69958998901345	0.84272199281053
C	-1.82651409616234	-3.45391509165890	0.70322906451213
N	-1.05009265271085	-2.31936918468858	0.82240978124588
C	-0.10206421397897	3.57112273378961	0.03377342750590
C	-0.97437659880248	4.15465892160820	-0.87647717236215
C	-1.21918558756678	5.51872561225765	-0.83263357772889
C	-0.59147896847333	6.31014755606172	0.11987791271193
C	0.28786111921594	5.73231836243820	1.02422177951495
C	0.53148912619609	4.36754324602971	0.97798497822905
N	1.22550875629627	0.91051430520926	-3.23390685550766
C	2.25132271871170	0.90905517493493	-4.14861562980896
C	2.07158203064588	0.25781072489634	-5.37608531368759
C	3.05743230130886	0.29664320574702	-6.32094931636457
N	4.79465959239290	2.48218359401841	-2.02869628640285
C	4.89172104430852	3.04486476152719	-0.67557197705580
C	6.38504209711708	3.07473621759519	-0.39156800214980
C	6.87524515802026	1.83099146980328	-1.12561606372954
C	6.08395479735383	1.87780393744604	-2.42579371715494
H	5.32906209357065	2.19167784715654	-4.68717652124549
H	2.48341634359680	2.79521864335353	-0.80681116166406
H	-0.45692209589284	0.78603544429552	-1.55343345696703
H	1.02403900447413	1.85816852247193	0.60572277029115
H	-1.91089666923301	1.42524872472832	0.06918676744417
H	0.16306236455753	1.02562824135427	3.16202616039256

H	-0.36276593829390	2.16168477868440	5.27958614011669
H	-2.24704752214286	3.75748467927567	5.39764803058648
H	-3.60919286471209	4.19890207271253	3.38129697704307
H	-3.07133651416327	3.06571013909306	1.25809111975894
H	0.27496941999520	-0.28788721807250	0.99122835900913
H	-3.32242623929518	0.07579554187023	0.53951071183707
H	-6.48237106576461	-3.30591730911774	-0.30601536692011
H	-5.20036641862004	-3.34534256972394	-1.53484104826178
H	-7.59801253856440	-2.22908078269646	-2.12540227115772
H	-6.10344261911290	-1.45306179202266	-2.67013111975015
H	-7.64559007070303	-0.80568423381584	-0.11495372520286
H	-7.20073514202759	0.33483706974492	-1.38782112630510
H	-5.55883227644458	0.05546764917755	0.75883452346444
H	-4.86591248083577	0.17449699678480	-0.86939012094228
H	-5.01151706128302	-4.52568281735469	0.30161388782589
H	-1.46388680466430	-6.80396612765364	0.86072887604926
H	-0.15453667204241	-4.75929220109301	1.01715904195642
H	-0.05685199478967	-2.43940727701685	0.96873574173224
H	-1.45970226408570	3.53950025239893	-1.62179294229794
H	-1.89996800034788	5.96500707327704	-1.54384644924766
H	-0.78242779489289	7.37347693469631	0.15260510962859
H	0.78460660208109	6.34337013843167	1.76462815164027
H	1.21166433451383	3.91441736537077	1.68708342029913
H	0.41209269428781	0.31951061119669	-3.38177130667765
H	1.14647558783658	-0.26742165180917	-5.56514387770859
H	2.93457363644242	-0.19999271267328	-7.27211562533854
H	4.41779605934871	4.02405388210046	-0.63291591609010
H	4.38328735093116	2.38490321271796	0.03258479932785
H	6.59383611539284	3.05968584184863	0.67483466535347
H	6.83614473384190	3.97119215068552	-0.81736400234175
H	6.61709831291934	0.93321924367165	-0.56550568966426
H	7.94693497276296	1.82799355944080	-1.30689403131075
H	5.91903873813376	0.89549567264088	-2.85534394983472

H	6.59707271458002	2.50918931213732	-3.15113258454735
O	5.34672216392601	-1.98067381319917	-0.52037894555021
S	4.19700710488359	-1.96947044006787	-1.40065523660368
N	3.14497706183699	-2.97975019007363	-0.93119187201413
C	1.87058685868203	-3.07144176707804	-1.51940293602020
C	1.02583124197038	-1.98195606597893	-1.70984133726095
C	-0.24550645823336	-2.11788707566098	-2.27599089735165
N	-0.96286059974110	-0.91710672683504	-2.43109718131610
S	-2.42260213801938	-0.76431395068014	-2.90520990489931
O	-2.90680804701572	0.54845073347336	-2.54712902783386
C	-2.31065087842492	-0.64665514561500	-4.74753515186959
F	-1.77676671890591	-1.76082383000650	-5.26495132954447
F	-3.51358885754661	-0.46596795540991	-5.30075627649606
F	-1.53049049431109	0.38467011467750	-5.11021862702619
O	-3.31360752527025	-1.88279961329638	-2.71041000983663
C	-0.69874257851962	-3.39009662364595	-2.61795092616303
C	0.13093988931157	-4.47809645590247	-2.38633283486010
C	-0.35911835446083	-5.84954619525796	-2.75189600826026
F	-0.01438513757521	-6.19802444187962	-4.01238846851488
F	0.14222929904044	-6.80849260333028	-1.94628665366788
F	-1.70141176613085	-5.95646181451192	-2.68589247107226
C	1.40190295282614	-4.34306626081447	-1.85936748089925
C	4.89347944037195	-2.65413763627541	-2.97269555336202
F	3.93384789865596	-2.76717062142846	-3.90352897997992
F	5.85033673022994	-1.85228138922503	-3.47107184263731
F	5.43185034717220	-3.86444802053154	-2.79047038880079
O	3.73830711714547	-0.66804605002916	-1.83888064947867
H	1.36933873580490	-1.00546883286647	-1.41570377482758
H	-1.68393383538007	-3.53551800583434	-3.02532606467396
H	2.03610776594843	-5.20011247290724	-1.69754708513031
N	4.30177973256752	-4.49766521927531	1.70566873375880
N	3.25788619730229	-5.06609746640370	1.41044279006366
N	2.37483091439479	-5.66524500447502	1.06836685139211

H	4.50873691269351	-1.85807906998415	1.42264695504616
O	1.69608713321079	-2.80420782985872	1.98466247146874
N	2.50353281724337	-1.90509964434794	1.86298311102814
C	3.91943270877330	-2.11504307936681	2.29913216922193
C	4.21976602320844	-3.51860309164749	2.80570590516093
C	5.57221487407532	-3.47514613658369	3.48396342538621
C	5.66564717394890	-3.56384564462038	4.86573233949099
C	6.90395312768770	-3.49305548622685	5.48984433412817
C	8.05352791564514	-3.33305191927768	4.73145016950252
C	7.96211103094174	-3.24361024969024	3.34823869535317
C	6.72659397584946	-3.31252338011101	2.72401516595513
O	2.22952746611373	-0.80673950508125	1.42020807173764
H	4.07567152718448	-1.37539599464172	3.08008066656878
H	3.46470605376479	-3.81894129065101	3.53091910164764
H	4.76872604442886	-3.69631666978292	5.45637345990688
H	6.96872366323690	-3.56910612931662	6.56611166826460
H	9.01880417843008	-3.28179088839465	5.21494350907546
H	8.85605919628209	-3.12130946330461	2.75315311710146
H	6.65683275100855	-3.24670692116595	1.64660308092770
O	5.13443405528291	1.03018612118527	-7.09972955426206
C	6.37003257149983	1.68629732936228	-6.87416910323411
H	6.22802103246752	2.75189040263215	-6.68395104857777
H	6.91159539737292	1.23585833013698	-6.04019773452506
H	6.94930868634443	1.56287311551593	-7.78390567480142
O	-4.16792227618248	-6.84719245045875	0.50237783464098
C	-3.57351879382712	-8.13388988164628	0.58463956358971
H	-4.38701291871384	-8.84666028273434	0.49198679946681
H	-2.86173721625601	-8.29629726255048	-0.22595733836665
H	-3.07590525742468	-8.28328305518929	1.54414639009563

Iig$_2$/I8 product (unfavored enantiomer)

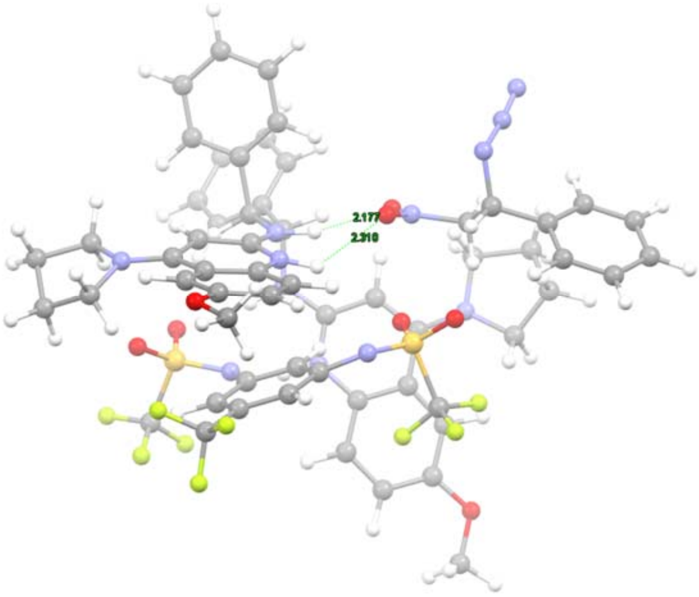

E -5239.498203

G -5238.470871

C	4.96833304628896	0.30633703654626	-5.24658654764457
C	5.05173337363613	0.95029174092564	-4.03133863586179
C	3.92109815052729	1.16506224620958	-3.23432509142550
C	3.92786873740058	1.80638177986008	-1.91972517652077
C	2.70696960217043	2.20296620073915	-1.38756788645187
C	1.50042277557895	1.80510587523784	-1.96871959729180
N	0.28541061760391	2.09308462612049	-1.47058442950098
C	-0.00922597048301	2.49760228443725	-0.10757196688388
C	-1.05628563196869	1.51246753083213	0.46933658084858
C	-1.60308668622006	1.97425783936591	1.80638176927696
C	-0.85319477752021	1.83546228383970	2.96861176734010
C	-1.34187218694644	2.30443224513140	4.17924502202911
C	-2.58723711253416	2.91668410474700	4.23865993414075
C	-3.34231514018763	3.05213274868560	3.08221665410650
C	-2.85090405966420	2.58049763920492	1.87298760039028
N	-0.45396668480852	0.19021425439149	0.54027876776789
C	-1.16103619828957	-0.95022397412286	0.51662495397000
C	-2.54447505635714	-0.98810321045971	0.36306761829740
C	-3.22085747693529	-2.18414797779746	0.14546783902059

N	-4.50729123815480	-2.15255629522997	-0.21932994996950
C	-5.24089142973257	-3.21263118623793	-0.94042407081822
C	-6.22647778639415	-2.42792769430911	-1.79668945301961
C	-6.58143558759484	-1.25280947663903	-0.88731109464936
C	-5.22114680314956	-0.86869301833411	-0.32085666519884
C	-2.48281840998579	-3.42763341522259	0.36640165482828
C	-3.06124262129272	-4.69940095784186	0.44147944685590
C	-2.29910493337669	-5.82882728495613	0.64712464902937
C	-0.91574801288387	-5.71543435606897	0.80527184238767
C	-0.33447181413568	-4.47109700811516	0.78432357115541
C	-1.10398438118116	-3.32957315999579	0.58079465232562
N	-0.48205124910709	-2.09668831366529	0.60494593501012
C	-0.51214391301728	3.92615634494654	-0.03706302211438
C	-1.47687366634786	4.38267842455956	-0.92859105712465
C	-1.97242297611029	5.67366869985620	-0.81923101494042
C	-1.51016469717346	6.51882263976915	0.18151996612976
C	-0.54419379170586	6.06799261823051	1.06992350486601
C	-0.04776625087319	4.77645272033794	0.95744371650275
N	1.51118559746129	1.08097304972754	-3.09312787738294
C	2.67910707401921	0.77794593376496	-3.75682426439942
C	2.60344044905187	0.09614243119208	-4.96940829525883
C	3.73315459129069	-0.15071546978294	-5.71114477914131
N	5.05894461511033	2.05027872447500	-1.23581454535043
C	5.01994752168342	2.78034679948301	0.03894703402234
C	6.45995751341919	2.74734837501022	0.53590593269557
C	6.99726443983601	1.44235664298715	-0.04900020664881
C	6.37242481011673	1.41420775662248	-1.43636182687668
H	6.02527449798681	1.30089250792053	-3.74254885240005
H	2.66334377010464	2.76147111713001	-0.47239582391250
H	-0.50744310854928	1.79481103761753	-2.02888941334142
H	0.88977899803131	2.41365206104042	0.49401783259656
H	-1.86780568172474	1.48250869680025	-0.25140067546945
H	0.11741495683754	1.36044359549619	2.93095024333611

H	-0.75004250144697	2.19149995152197	5.07711874860892
H	-2.96810722581185	3.28215883039569	5.18219319630106
H	-4.31436620467604	3.52413403303672	3.12019145126831
H	-3.43652539991179	2.69308829628780	0.96990907134791
H	0.52037017594153	0.14236790084062	0.77851352570403
H	-3.07083059519179	-0.05760479859087	0.30265404965529
H	-5.78716334912862	-3.84489983283953	-0.24152168588794
H	-4.56549594611957	-3.82417910091687	-1.52707804388826
H	-7.07896310562146	-3.03421377628458	-2.08993158780572
H	-5.72452920097430	-2.06585340010941	-2.69136657722767
H	-7.25010639000019	-1.58063769146368	-0.09176539371036
H	-7.04504761481472	-0.42280427596042	-1.41266956465611
H	-5.27610541883301	-0.38592881977466	0.65266578831248
H	-4.69448920969640	-0.21857108936833	-1.01777829232944
H	-4.12292391207488	-4.84400472310465	0.36383556274700
H	-0.29384269173275	-6.58499976287005	0.94770714790084
H	0.73588698026106	-4.36945645768030	0.89601022177815
H	0.51781420720132	-2.09095467521525	0.60198818449025
H	-1.84810219511975	3.73090254484487	-1.70830959000108
H	-2.72292546438050	6.01955735167020	-1.51664395679252
H	-1.89912107499956	7.52404582425674	0.26585156757270
H	-0.17736615691107	6.71996203931288	1.85069842465804
H	0.69945227017909	4.42231939139117	1.65584363090799
H	0.62308912776351	0.62513017540912	-3.37300333836940
H	1.63371086088309	-0.22202288075177	-5.32682306607342
H	3.64754313742696	-0.68179921931428	-6.64576742739515
H	4.65011983338328	3.79289178485769	-0.11282188831557
H	4.35301308492051	2.27505214084799	0.73633736065300
H	6.51176835301310	2.78714098894603	1.62055258050793
H	7.01768751965460	3.59357540624838	0.13689785105536
H	6.65668263733881	0.59043876807716	0.53582571609045
H	8.08213675770426	1.40768487234445	-0.09673576521910
H	6.26259673689179	0.41020014188087	-1.82433919469014

H	6.98497865480590	1.99762178858579	-2.12330740320749
O	4.21956076117735	-3.06522964718700	0.21460830333125
S	3.33864730050044	-2.56994604506559	-0.81867935139504
N	2.13760252582734	-3.50839732779781	-0.99931329673954
C	1.04172197826402	-3.24481069044485	-1.84947734820716
C	0.61155206139773	-1.98398844632067	-2.24594159737810
C	-0.57076846298207	-1.78591751350659	-2.95685305903257
N	-0.86919523104137	-0.43240865943315	-3.22310944056568
S	-2.27790697291246	0.15930210529241	-3.40855818012162
O	-2.24673162761104	1.56108114896345	-3.02912254111414
C	-2.52587917216367	0.27232842172879	-5.23694944550413
F	-2.71382384024462	-0.94343040943873	-5.76343612951604
F	-3.58800122805542	1.02598277606899	-5.53092478794995
F	-1.45083175305287	0.82207798236662	-5.81700721472857
O	-3.40536906987621	-0.62075223177278	-2.96691932624418
C	-1.33940810903005	-2.89398383477648	-3.31045772932148
C	-0.89543347092773	-4.15610467783911	-2.93615588240631
C	-1.73903951944350	-5.33256242157528	-3.32736331493991
F	-1.65175322581829	-5.60551022454811	-4.64978069447222
F	-1.39845077247202	-6.46313268231095	-2.67828244228890
F	-3.05292298264138	-5.11712834359272	-3.07611081216062
C	0.27392857909698	-4.35117005947062	-2.22599176672708
C	4.36203574792246	-2.74991620083444	-2.35176651067087
F	3.65564532523784	-2.40702698522244	-3.43616181730342
F	5.44520031718642	-1.96376325268749	-2.29949989198732
F	4.77131791216469	-4.01070767607639	-2.50498935995899
O	3.10691715665269	-1.13489190721770	-0.78460515635545
H	1.17270644767919	-1.11954294399557	-1.94475162859789
H	-2.26760744011881	-2.77988676353756	-3.84482221616769
H	0.59602478091282	-5.33431739222897	-1.92369704873729
N	4.16386869609373	-1.42797109226561	4.33728910726308
N	4.57812416812082	-0.44695041779151	4.93718402278824
N	4.86623807010810	0.41199580665489	5.58733302094930

H	4.62704006674562	-0.73794615195010	1.03055528383710
O	2.09379865423224	-1.25748625205091	2.07057983770427
N	2.82718720064337	-0.31529393285242	1.89886682077375
C	4.30597965426391	-0.50167064355026	2.03598585344858
C	4.66727031151858	-1.64454029245389	2.96085312805067
C	6.15904723031262	-1.89906804202570	2.88217395967796
C	7.08760464835066	-0.92502498826940	3.24314090237569
C	8.44795884199693	-1.18323591615157	3.14510805056569
C	8.89148496433775	-2.41310058924358	2.67866807784330
C	7.96969308192689	-3.38239869988862	2.30833943796552
C	6.60972136016028	-3.12618733815766	2.40951576511907
O	2.43681597859915	0.79184588771893	1.58263421353608
H	4.69368315619710	0.45753787564419	2.34435868549961
H	4.15347016320836	-2.52517705642881	2.61001619011611
H	6.76544476325870	0.04194604647938	3.60290703144266
H	9.15965497385913	-0.42079628669703	3.43034064165804
H	9.95110590797165	-2.61345791667657	2.60111342241521
H	8.30805754347856	-4.34010598563305	1.93793347362807
H	5.88905718035184	-3.86952386078478	2.09980661359840
O	6.13702227572007	0.15483084745741	-5.92439612569742
C	6.07865269667429	-0.49726541768010	-7.18323329155230
H	5.43902092235995	0.04055971793852	-7.88395523273561
H	7.09356883130753	-0.50716112313944	-7.56584737509927
H	5.72554187458501	-1.52435750943305	-7.08614959948503
O	-2.97319799038412	-7.00614119918756	0.68491866995938
C	-2.20347761986828	-8.19396528133088	0.79947478182745
H	-2.91097166133101	-9.01548711954526	0.77038188926061
H	-1.50392649520623	-8.29598900897856	-0.03037895074420
H	-1.65743149589492	-8.22791728124038	1.74271884994890

Iig_2/I4 with hydrazoic acid and nitroalkene

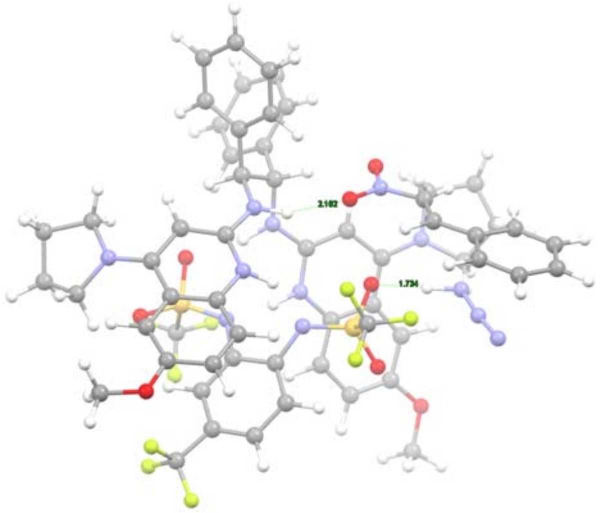

E -5239.472408

G -5238.462898

C	7.35628890707777	2.66625287495848	2.51669351036049
O	7.06964839289542	1.36160951505860	2.05641609706017
C	5.77419789181855	1.02341006409434	1.84596571956692
C	5.55267266001489	-0.27066118086348	1.36909659235540
C	4.25550011714634	-0.75766829547222	1.10300316541319
C	3.93352310724947	-2.08869722612962	0.57405948598229
C	2.58280428912186	-2.48136606561820	0.57829719265917
C	1.55519868193391	-1.58218003168729	0.91257991839745
N	0.23881176703434	-1.87021485765994	0.85861003245719
C	-0.36136580316065	-3.01148961213452	0.20063915619011
C	-1.72429064782312	-2.50858944274515	-0.37750079724758
C	-2.49737124330233	-3.60452269148337	-1.08712279009796
C	-3.51615656722606	-4.29582854869152	-0.41493317874402
C	-4.20280984064001	-5.33929820881775	-1.04467904080087
C	-3.87763927548426	-5.70001945508707	-2.35666135338797
C	-2.86474946467115	-5.01120535542740	-3.03491204488072
C	-2.17805382729621	-3.96914348849128	-2.40432170439367
N	-1.44587784956170	-1.35502859645388	-1.21916459376331
C	-2.14409427874069	-0.19693087991684	-1.19941019839197
C	-3.40329087155144	-0.06122637568758	-0.59569931355820
C	-3.98962016162290	1.20399052242629	-0.39861343186244

N	-5.04154245300647	1.32184374648926	0.44565393941729
C	-5.49223422453143	2.55833546747996	1.11488071160987
C	-6.03055115360683	2.07107355045747	2.45917695702042
C	-6.59799529637686	0.69594881233351	2.11422815491024
C	-5.53451292044271	0.14081254712212	1.17368421897937
C	-3.42530142934833	2.32804295385495	-1.14709822631149
C	-4.04130515704181	3.59827984406405	-1.28616270559322
C	-3.41620721258850	4.63089518033497	-1.98350292644453
C	-2.16999064771345	4.40324157584726	-2.60672879564511
C	-1.57700592309565	3.15927727169364	-2.52999284525873
C	-2.18657935862927	2.11440312575696	-1.80644132947836
N	-1.54771974681262	0.88936785407079	-1.74615737883071
O	-3.92610782002017	5.87417507425472	-2.12636059229133
C	-5.07699141559852	6.21542608801599	-1.38270558333282
C	-0.54374902741715	-4.21251923367078	1.11153132386303
C	-0.35628728253027	-5.51116904399626	0.61583625805246
C	-0.56950805668494	-6.62252811277836	1.43856433030797
C	-0.97065821768411	-6.44435403558018	2.76720701667582
C	-1.15440007925978	-5.15020586963164	3.26948269030887
C	-0.94173679254185	-4.03979980178779	2.44655750349846
N	1.86484765524433	-0.32483459975786	1.28829162284458
C	3.16328988421427	0.10540146628166	1.41542746161409
C	3.39338183318389	1.41317123566192	1.87493527792035
C	4.68209263351703	1.88304345661607	2.08050629222087
N	4.86753950117637	-2.96020412582262	0.11315884342398
C	6.25946706183302	-2.67669626443625	-0.27749814443617
C	6.57834532519670	-3.77697208122852	-1.28711670596024
C	5.78708021973093	-4.96585970820159	-0.74593396444823
C	4.47755971572924	-4.31592813991543	-0.30813099524902
H	6.90245450230204	2.85871224503160	3.50917570777635
H	8.45284738649903	2.72409772389208	2.60690179784931
H	7.01238680888984	3.43669034510896	1.79864749047369
H	6.43934926018741	-0.88601607360873	1.23762028264680

H	2.28951081228558	-3.48110040924144	0.26573049821533
H	-0.40978930357367	-1.19610200647732	1.29419907683818
H	0.26935522684018	-3.30432824298824	-0.65950064746128
H	-2.31458725348630	-2.15868340364124	0.48786115735538
H	-3.76719293492918	-4.01697383676392	0.61644441892736
H	-4.99754006275376	-5.87108853257630	-0.50798528706779
H	-4.41643524557722	-6.51595809280403	-2.85303089202895
H	-2.60788015381372	-5.28671615963238	-4.06484304502785
H	-1.38633589744714	-3.43033065451576	-2.93934279480147
H	-0.58808114177980	-1.38545425544523	-1.77945660251670
H	-3.85540163494148	-0.95346908398555	-0.17018718986706
H	-6.29917146930702	3.04554629637649	0.53206985977667
F	0.43779962197912	0.69906544306537	3.96679702279707
H	-6.77547737637511	2.76680833588858	2.88274253709491
H	-5.19226772963875	1.97002507548401	3.17155597747509
H	-7.56645615473613	0.79516190368636	1.58760107451883
H	-6.74908581947108	0.04593720949174	2.99295223592784
H	-5.92428344842377	-0.61727664885755	0.47044685511791
H	-4.69514657510121	-0.30789977219764	1.74407895144616
H	-5.02800734816124	3.76817089387043	-0.86473633574751
H	-1.69469460184684	5.22441561164622	-3.15290332616485
H	-0.62423765954498	2.97174437666035	-3.03260205842885
H	-0.50693974826147	0.90450963507815	-1.79823944122496
H	-4.92162419085115	6.04280115006751	-0.29956863145850
H	-5.96746590454667	5.65028842216710	-1.72445482064558
H	-5.24847265933617	7.28960653244379	-1.55751165956191
H	-0.04617056002510	-5.65063038882782	-0.42756256242322
H	-0.41845029339371	-7.63291557978529	1.03987618622928
H	-1.13552385382882	-7.31432582097889	3.41410737384021
H	-1.46332637246397	-5.00376096454352	4.31150569553354
H	-1.07931504498921	-3.02635392077702	2.84481955758066
H	1.08745118650346	0.38380782419703	1.29617789495999
H	2.53089564795599	2.05499288740054	2.08588677493998

H	4.82677617431735	2.90668075236357	2.43578012009202
H	6.34032267213831	-1.67208088082490	-0.72056767393532
H	6.93574095649092	-2.75033632744486	0.59746189905872
H	7.66335592841308	-3.96262652483998	-1.36771021149106
H	6.20036950298755	-3.48016783323077	-2.28389535450146
H	6.30708822954604	-5.40720233450220	0.12542108721358
H	5.62341165500375	-5.76657239107142	-1.48750893349007
H	3.98438433824284	-4.85173078416299	0.52388770889654
H	3.75400702792557	-4.25417696737039	-1.14649601252956
O	-1.69494930062081	-0.31755094282256	2.26089700990723
S	-1.49076013534706	1.15550335994728	2.22025853218657
N	-0.38534218090407	1.47162810556698	1.14616913394626
C	-0.09460355394394	2.74542585375115	0.64375401620187
C	-0.63783712301821	3.95311367146887	1.11138717084365
C	-0.24198375233820	5.18274442557328	0.56958989385349
C	-0.96385276318717	6.41776656708515	1.00072801233763
F	-2.17126358634269	6.53919322080447	0.38546115604562
F	-1.22463888770929	6.43271240883024	2.32454686621181
F	-0.28322140762324	7.54431289708724	0.71785720971986
C	0.74974007921711	5.23609101375972	-0.41413744930914
C	1.28612345361284	4.04543125216379	-0.90458776395611
C	0.84331438056771	2.79122183442490	-0.44345824089591
N	1.17547820130489	1.55989259905263	-1.01432222451847
S	2.47379649775648	1.27880710951674	-1.84065013239538
O	2.65491674679739	-0.19918626332963	-1.94755104586340
C	2.07458653654819	1.67527640068729	-3.63226263498014
F	0.96521838134612	1.03262666374409	-4.02752195363282
F	3.08557739132324	1.28812256758925	-4.42232985857711
F	1.87841514329868	2.98495306931218	-3.82503306055412
O	3.68318726013421	2.08228602906890	-1.59131131231619
C	-0.66952110932916	1.44597038611104	3.87771666688384
F	-0.31921207834573	2.72956148725926	4.01130354287069
H	-4.64851861634629	3.25525185214543	1.22845045164054

F	-1.48410278448749	1.11956337900124	4.88079081175125
O	-2.69882646406166	2.00280535111055	2.28263543713506
H	-1.38866110084908	3.93398240340018	1.90448511731279
H	1.08598374620141	6.19635787564164	-0.81782386781920
H	2.04442655303767	4.08468809020495	-1.69016603805187
H	4.26649813423529	-0.58788288306616	-2.45615441331425
N	5.22173502822328	-0.81307461058499	-2.84101191768404
N	5.63433987252476	0.05077849599381	-3.60277271912543
N	6.12769940001628	0.77616842321555	-4.33473774399821
O	1.63140346652236	-3.63314614960295	-2.44507556778198
N	1.57753850186666	-2.72778934471502	-3.28245897412753
O	0.61446465191995	-1.96728123355491	-3.40231235822840
C	2.70677698467071	-2.59666386363212	-4.14666591541201
C	2.71823896990183	-1.67491521233388	-5.13555718976577
H	3.50184265383797	-3.29979779064253	-3.89643320504336
H	1.82199607158343	-1.05187594049010	-5.24055483891896
C	3.81250355238393	-1.41524744038249	-6.05173237670673
C	5.03837825321554	-2.11970748920365	-6.00862247545321
C	6.07080071265130	-1.78842914300099	-6.88105348195579
C	5.90341908151121	-0.75461548962071	-7.81468991044678
C	4.69445572846188	-0.05291868412049	-7.87406668828448
C	3.65863594487588	-0.37912466361758	-6.99958323108222
H	2.71568410058008	0.17864368978393	-7.03011257303478
H	4.56285468565036	0.75789892938296	-8.59939089465849
H	6.72289380206536	-0.49408098037070	-8.49460473860346
H	7.01991667050212	-2.33431623508212	-6.83318184832797
H	5.18847488409687	-2.92103829903403	-5.27770560993269

Iig$_2$/I4 TS1 (favored enantiomer)

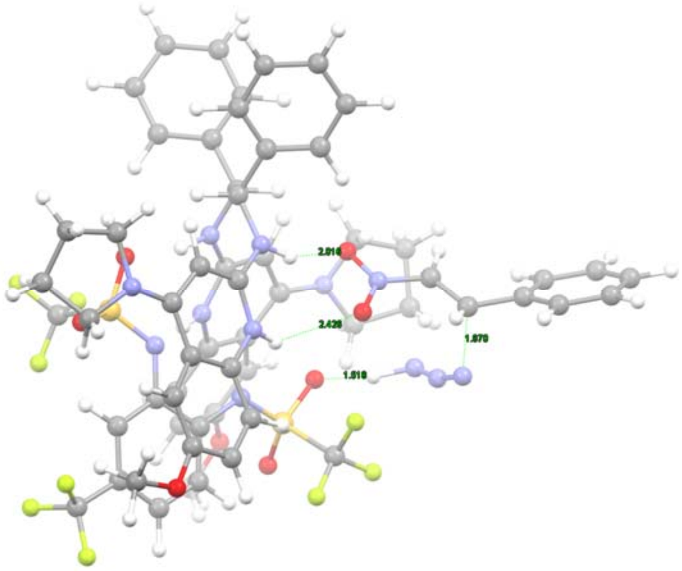

E -5239.422158

G -5238.405753

C	6.58139077986615	3.05731241902685	3.89912892797453
O	6.36670383088361	1.79551477588439	3.30636812148849
C	5.09278005328386	1.43086912355627	2.99019049571513
C	4.96399211330328	0.20365095848038	2.35732901725817
C	3.71989718887039	-0.29921387062675	1.96714304392309
C	3.50717685930115	-1.54518055267794	1.24943317693856
C	2.22044362759318	-2.08347896592499	1.29411794321258
C	1.12676232466181	-1.31550849190949	1.69093989120370
N	-0.14081448624684	-1.75159012929278	1.70102156925356
C	-0.68393636412862	-2.88611126234141	0.98947930745851
C	-1.96347368940562	-2.38842543650028	0.26194867525258
C	-2.59754938854085	-3.50769277494082	-0.52625621034925
C	-3.70964541955625	-4.17016137473621	-0.02278223064228
C	-4.26917415612297	-5.22854693742982	-0.71704148027660
C	-3.72457822856544	-5.63257313013857	-1.92422365309828
C	-2.61570514361010	-4.97642869070066	-2.43209440859937
C	-2.05211658102387	-3.92273638543559	-1.73464324827862
N	-1.57820213710040	-1.25281604268747	-0.57203725794045
C	-2.18663981555962	-0.05127119420947	-0.52555770132269

C	-3.49751379455724	0.11055410019604	-0.04732366058665
C	-4.09104684212273	1.36143184654468	0.13364214893323
N	-5.28549874306063	1.43602603441179	0.74783004771749
C	-5.86106916041035	2.60847111126052	1.39372059551859
C	-6.66651471537251	2.04556339334155	2.56208010514799
C	-7.16659380256713	0.72547444405249	1.99280996661807
C	-5.92910942338147	0.22710551912902	1.25655589853182
C	-3.38781112924319	2.50228917151500	-0.43323588111452
C	-3.91668130366853	3.80088389583004	-0.54364427822020
C	-3.18157196338947	4.84847677220848	-1.06625572336689
C	-1.88638479040888	4.61156344051526	-1.55165753993000
C	-1.37634580922979	3.34389938993312	-1.52005880372762
C	-2.09974373590655	2.28474644023568	-0.96155415257311
N	-1.52436407681554	1.03241083928278	-0.97529807724996
O	-3.62315885563926	6.12631107485661	-1.18177428631162
C	-4.89540315940646	6.46322223421781	-0.67095845722411
C	-1.04510868324959	-4.03067419592494	1.90743140784976
C	-0.80248960550335	-5.34189399212875	1.52081891081392
C	-1.18444223062954	-6.39377349610085	2.33521617782280
C	-1.80856654479957	-6.14196095680366	3.54511724592978
C	-2.05184790597760	-4.83521218628608	3.93586855851984
C	-1.67729033983364	-3.78182268955926	3.12003058690868
N	1.32281175908103	-0.05114537846944	2.10521418368288
C	2.57636958259019	0.44696481586445	2.32800707626683
C	2.71466964275900	1.68818424552191	2.95053800135481
C	3.95303745418655	2.19130694336011	3.26703039682926
N	4.48151153023298	-2.19279360470307	0.58914878113847
C	5.73356304069490	-1.61726263468782	0.12063181920571
C	6.02839789909821	-2.36062618609833	-1.18122967540144
C	5.48159915453959	-3.75145890483130	-0.88210037565905
C	4.19771434209262	-3.43471339143168	-0.12194557325204
H	6.09766365317321	3.12822443241437	4.87868265394309
H	7.66021713486836	3.14060531862212	4.02294395634647

H	6.22483707373154	3.86676472882659	3.25485841729002
H	5.87007052127690	-0.35662047410634	2.21481678112019
H	2.02491037238168	-3.07536540485774	0.93459120343310
H	-0.83721236771089	-1.09357146893565	2.04683753175078
H	0.03336498451121	-3.21998296539339	0.23431546148790
H	-2.64882594021246	-2.03932489981601	1.04096314564890
H	-4.13196312862470	-3.86076129326078	0.92265227638569
H	-5.13205619731376	-5.73821989193863	-0.31470778624183
H	-4.16170968020583	-6.45732520147078	-2.46735562133378
H	-2.18582344289361	-5.28914101028990	-3.37260691601765
H	-1.18255359222783	-3.41971055447437	-2.12767549053105
H	-0.62232178155520	-1.31070629801788	-0.96120268139080
H	-4.01891970435603	-0.78054607638399	0.24571632592185
H	-6.53598611923407	3.12504699772634	0.70011487421524
F	-0.36261045361003	0.56329462360337	4.79796995860676
H	-7.46383212103742	2.71721820703182	2.87308217109190
H	-5.99606831244240	1.86560762078175	3.40260519670714
H	-7.98603631896605	0.89940934234044	1.29353441124525
H	-7.49654900653239	0.02601381832679	2.75733369558616
H	-6.17628537758780	-0.44476977578932	0.43092876002963
H	-5.25556289774531	-0.28002677883091	1.95778623598755
H	-4.92908408380038	3.98683107910248	-0.24718950241376
H	-1.32580952830236	5.43577609164202	-1.96155874600283
H	-0.40323383289641	3.13578847169551	-1.92915973327708
H	-0.50970193127931	0.98364626938578	-1.15753855022576
H	-4.96503896428055	6.24654924262537	0.39889986332219
H	-5.69275775557948	5.94328506650554	-1.21157870784811
H	-4.99471398409845	7.53602768246423	-0.82820092345310
H	-0.32085520584037	-5.53897692436510	0.57371748758568
H	-0.99490260551396	-7.41067485590550	2.02472624438083
H	-2.10645663311432	-6.96177645040984	4.18216032211229
H	-2.54057474885920	-4.63518262349992	4.87837303464250
H	-1.87706381082437	-2.76335774074745	3.41979687694172

H	0.51896180598079	0.59844751659701	2.10384388800831
H	1.82411769278892	2.24998061598496	3.18839482269662
H	4.02356485126808	3.15865279749053	3.73657812743080
H	5.63079016947907	-0.54197457408562	-0.02735766547873
H	6.53372244315818	-1.81785557711150	0.84250528488016
H	7.08949573877328	-2.35922822278553	-1.42025475942531
H	5.47907470481555	-1.90145940877769	-2.00332388153725
H	6.17568207129893	-4.29915216373422	-0.24343569258073
H	5.29391274592188	-4.34035420677489	-1.77726334910814
H	3.93057550361082	-4.22300680719925	0.58601986933032
H	3.36072003078076	-3.27871368396019	-0.81226269088763
O	-2.31319781081829	-0.22565746644732	2.75622338629779
S	-2.12985956231315	1.20657989275081	2.86859746979720
N	-0.94408765008978	1.63112588456043	1.96603318805339
C	-0.62349309585678	2.93911953377381	1.69663711442754
C	-1.33438202311020	4.02013197281487	2.18992609764491
C	-0.92582644053235	5.32632838717825	1.93683904102757
C	-1.76160159780327	6.43540822904227	2.47824746560548
F	-3.08427802126619	6.25734615147640	2.25947456103459
F	-1.65147466589213	6.58774290522415	3.82235755307979
F	-1.47158323491568	7.64357115099369	1.96240893190988
C	0.24226740479679	5.57983446497956	1.23790904932862
C	0.96206774739223	4.52017665974821	0.71857038979449
C	0.52993777585930	3.19377531186659	0.87500259240733
N	1.10822949409411	2.09253338393977	0.24908687382764
S	2.56543291008779	2.19364883349039	-0.34661083184141
O	2.98704942141179	0.79551781564515	-0.68581745386201
C	2.45708213668888	2.91064216094621	-2.11616046666423
F	1.63827343217375	2.17857613299846	-2.87977694334872
F	3.68305194918732	2.84579689549955	-2.66322181747548
F	2.05308492525358	4.17945207350719	-2.14290235481997
O	3.57129668918188	3.07119952304389	0.27741242616342
C	-1.45971020950554	1.32360914278820	4.65686449030147

F	-1.11951504557616	2.57386011580170	5.00307156183682
H	-5.07655294896745	3.27753038896040	1.74489261436480
F	-2.36100706552815	0.89203433269778	5.54893670407097
O	-3.34185059499873	1.98286314207825	2.93219852324070
H	-2.22111603866687	3.84535202347356	2.77673959274123
H	0.57882507546622	6.59338258439585	1.08370489309377
H	1.87438471921076	4.71080987508596	0.17892156963349
H	3.72355383483891	0.29400571273688	-1.91490542390857
N	4.09101942821900	-0.20076420192204	-2.81490532360755
N	3.55046331790908	0.15745435634083	-3.82793946151203
N	2.96528911033271	0.18517536836140	-4.83316231463955
O	1.07154382712428	-2.17437222857897	-1.63007942016707
N	1.13937513709874	-1.51283283786895	-2.68918791408241
O	0.52291416360900	-0.43524980894998	-2.83247366931762
C	1.91300663348247	-2.02119386820343	-3.72731801997073
C	1.95582511898020	-1.38250282635766	-4.97552637232453
H	2.47116465410403	-2.91684246389909	-3.45530751854608
H	1.07419142667611	-0.77191761462974	-5.20973891299406
C	2.61949234382138	-2.04904765403524	-6.11934613901848
C	3.73017215137191	-2.90243519878577	-5.95098220832061
C	4.30893802002486	-3.51039869380889	-7.04681608075837
C	3.81248930213243	-3.28363529528380	-8.32174118167688
C	2.73388078108875	-2.43258300980587	-8.50671922461152
C	2.14412705498682	-1.80985078173066	-7.42463568857135
H	1.30682584563739	-1.14175305590915	-7.56569980409029
H	2.35587569616121	-2.24911789568729	-9.50151338350597
H	4.27318963037486	-3.76330489520843	-9.17231238045317
H	5.15853632303340	-4.16265231548236	-6.90878606778365
H	4.14107739484905	-3.06501701649865	-4.96566529552948

Iig$_2$/I4 intermediate (favored enantiomer)

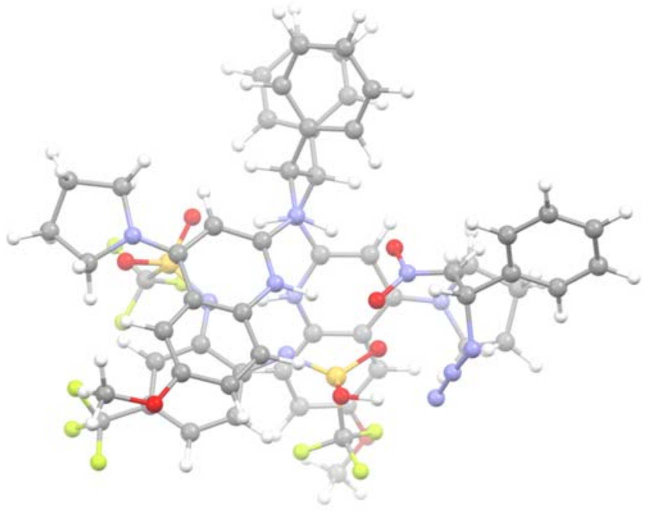

E -5239.430254

G -5238.412651

C	6.69120346281510	2.68147635721268	4.68494020891053
O	6.48796316136246	1.54707797356022	3.87085513071882
C	5.22202576665309	1.24083555167541	3.47793316840431
C	5.09269274019883	0.09172659055399	2.70961017363301
C	3.84847326889123	-0.34438128601083	2.24879560744431
C	3.63499761164751	-1.48930610427247	1.37983992583523
C	2.37136782573770	-2.07827992934770	1.42852339400207
C	1.27442032139331	-1.37870616753631	1.92718958200227
N	0.02059211845492	-1.84892133824649	1.94083695337606
C	-0.49988892686906	-2.94112331858687	1.15169347711426
C	-1.75563886604962	-2.41481646909811	0.40755314427516
C	-2.36542923351144	-3.51382393678835	-0.43100723932071
C	-3.52321930202530	-4.15416649164633	-0.01024916068255
C	-4.05854688338088	-5.19274744930577	-0.75221611526224
C	-3.44328200504580	-5.59775700230995	-1.92440460085969
C	-2.28795370630145	-4.96293864389935	-2.34961475472815
C	-1.74794545811514	-3.92929684979915	-1.60496434338784
N	-1.33458710233264	-1.27914842812924	-0.39717859826839
C	-1.96473115303431	-0.09566421007544	-0.48867989099400
C	-3.18291089132744	0.20263975622776	0.15247291423355
C	-3.78685514663848	1.45779617315194	0.09675958885576

N	-4.96649775723338	1.63514994533878	0.72389242952516
C	-5.53724297568238	2.88904348094944	1.19464553449951
C	-6.40682962552587	2.49077470179380	2.38481680322094
C	-6.91404143715113	1.12215080755121	1.95274611335072
C	-5.64670965753864	0.51357890766993	1.36589309719090
C	-3.13933656940870	2.46686509823814	-0.73312080437478
C	-3.64743945865030	3.75169758663927	-0.99745088036344
C	-2.99097246467804	4.64349911818283	-1.82436155493718
C	-1.78820589574437	4.26636959975442	-2.44116101288702
C	-1.28760591127050	3.01174953806444	-2.23276990426389
C	-1.94322802559311	2.10817826255612	-1.38794308841941
N	-1.39877397060288	0.85234917149331	-1.26085627396798
O	-3.42723998236419	5.89595292541228	-2.12021475118261
C	-4.65509237645971	6.34486170063339	-1.58887092443215
C	-0.87863056504611	-4.13690863614364	1.99465487878728
C	-0.61081239419612	-5.42325677498280	1.54594794410852
C	-1.00990255399310	-6.52122465432236	2.28807177060304
C	-1.67685514229309	-6.34111970889404	3.48782779782733
C	-1.94492490573677	-5.05960760563823	3.94065208374382
C	-1.55334207088254	-3.95985833177697	3.19704872153609
N	1.45244929746300	-0.14671260297745	2.44380653166523
C	2.70157065587830	0.35559009660005	2.68237790445811
C	2.84356682756126	1.54377631333142	3.40033089091352
C	4.08510616644023	1.99698697009975	3.78360275794119
N	4.58552035084289	-1.98218406386079	0.57525708029925
C	5.77210696979609	-1.26367257765043	0.13673551394069
C	5.89181606224867	-1.62159961502827	-1.34251580825922
C	5.43199748545728	-3.07458104902238	-1.35007531534621
C	4.25740097998282	-3.04016792765494	-0.37660959940583
H	6.13837220266104	2.60365985342083	5.62621693830386
H	7.75960569687629	2.70136164778114	4.89421014330773
H	6.40684887726164	3.60348302683164	4.16828396965162
H	5.98858182304520	-0.47278561735064	2.52022173337414

H	2.19803855660879	-3.03662770495856	0.97839739690472
H	-0.68448215402726	-1.22847462517278	2.33524656559321
H	0.23933671198360	-3.22662763279695	0.39792968510202
H	-2.46462753912054	-2.08557184137015	1.17250356421413
H	-4.00125020894187	-3.84397499507559	0.90786921147482
H	-4.95731912178811	-5.68674328873708	-0.41391883357849
H	-3.86112493288250	-6.40736444994168	-2.50442314533186
H	-1.80206833905054	-5.27765397451786	-3.26189034756745
H	-0.84032108164351	-3.44533745372155	-1.93293321128650
H	-0.42113801862784	-1.39566793699050	-0.89006199993959
H	-3.62694505399986	-0.56958265497176	0.74902317840814
H	-6.17528584212253	3.32787688201867	0.41712211609981
F	-0.26481580339831	0.72829023320258	5.21380294632344
H	-7.19789512357232	3.21277862527203	2.57555021785284
H	-5.78063192818473	2.39144502692093	3.27110809652631
H	-7.68464383844332	1.22406990188090	1.18669744353193
H	-7.30748733001538	0.53092990750436	2.77619359027565
H	-5.85006038997359	-0.26947970198345	0.63101741423752
H	-5.02719115534922	0.11298948061093	2.17633179971857
H	-4.57890033677326	4.05494276379383	-0.56625032903343
H	-1.29118521199999	4.97556115246410	-3.08277763316006
H	-0.37426244648142	2.69207978767711	-2.70862213379210
H	-0.52050575236575	0.62796610856726	-1.78484967729973
H	-4.64122888725163	6.36411116028282	-0.49587990421262
H	-5.48867871651759	5.72613130414023	-1.93581224363948
H	-4.77837259245526	7.35946011136960	-1.96425223331282
H	-0.09597446996765	-5.56505758980981	0.60642434117633
H	-0.80022818914815	-7.51792808359385	1.92875176054194
H	-1.98848349732351	-7.19712751889475	4.06819286159692
H	-2.46687780169484	-4.91528837400393	4.87544151832871
H	-1.77286700944155	-2.96169268627636	3.54664779929916
H	0.64011785928348	0.48585771760909	2.48648693125905
H	1.95434176041605	2.09240596342331	3.66930553186683

H	4.15835477943032	2.91706222901364	4.34027303234114
H	5.65747617810626	-0.19227473872775	0.29133208528362
H	6.65129407323413	-1.62767385508072	0.68122320288205
H	6.90188656025226	-1.48521003545523	-1.72253528202895
H	5.20235142215927	-1.00467761392408	-1.92060494705783
H	6.22558927707398	-3.72177875152728	-0.97342286636334
H	5.13440751403030	-3.42385339884470	-2.33642242408065
H	4.12109693904222	-3.99091316432401	0.14503984435889
H	3.32894790124229	-2.77766957192734	-0.89633548066048
O	-2.14904788724950	-0.43847419453873	3.20622767050841
S	-2.07547961204915	1.00557837097658	3.23655224024152
N	-0.89644854543013	1.46652601775811	2.33548373776642
C	-0.77561582731355	2.71848042134921	1.81190247428612
C	-1.58581598201324	3.79671971452087	2.14432146139252
C	-1.40831703222041	5.03729417338123	1.54096753072154
C	-2.32305338875507	6.14368672240168	1.95391338299626
F	-3.63036621516279	5.84279366503134	1.75936983665270
F	-2.22926137120350	6.45235096170042	3.26526206644533
F	-2.12484074354172	7.29688813672241	1.29465026496582
C	-0.41877101600103	5.24440483048517	0.59496031589425
C	0.41604898523809	4.19390720298593	0.25629782252795
C	0.26210638558266	2.93441162570955	0.84492151296728
N	1.07170576008192	1.83562383618380	0.57272540105872
S	2.23042795125743	1.77286418981058	-0.41112272952108
O	1.86897922918720	2.50030956407834	-1.79610173886034
C	3.67868673669203	2.92359302704360	0.12085689634252
F	4.08683151478306	3.68210676245614	-0.89482999384118
F	4.69456927781659	2.16456779872144	0.51597350816470
F	3.29710916050193	3.70211956734854	1.12648982141810
O	2.85860686933934	0.47576031276165	-0.62323250211635
C	-1.45625695798047	1.30759957294207	5.01302672167427
F	-1.30728505069063	2.61638310674295	5.26388656491251
H	-4.75583222119235	3.58858296506829	1.48809186094604

F	-2.30218820163936	0.81710208784886	5.92632680813235
O	-3.33680251739682	1.69867533502104	3.21212335387009
H	-2.36693902386023	3.66340421995648	2.87521781315329
H	-0.29756274434504	6.20924566983654	0.12908299280147
H	1.18141661556636	4.34044943128553	-0.48942239393328
H	2.49808273746767	2.15440732762123	-2.53345089670366
N	3.56574216784555	1.39200896742641	-3.51115562959110
N	3.13526276579649	0.75397329321025	-4.37238525966229
N	2.77356544845240	0.13594968022163	-5.33672155508321
O	1.13408276985296	-1.83604338128570	-1.72914002697336
N	1.24937283805227	-1.19322490770363	-2.81976498610732
O	0.73437132855144	-0.02136273559097	-2.94084180789383
C	1.92002352985662	-1.72403136869517	-3.85468111715703
C	1.86722755407169	-1.06158772495809	-5.18001871666680
H	2.37445624413819	-2.70116264212957	-3.68951353669844
H	0.85650615495872	-0.61923105098243	-5.29881442179741
C	2.13739678497607	-2.04057642168937	-6.29931294308912
C	3.45210706561038	-2.38068785659694	-6.65515660890606
C	3.68286216680134	-3.30517115764468	-7.65658512567087
C	2.62053741247936	-3.90918535459714	-8.30890961834703
C	1.31899845764249	-3.58778267757735	-7.96015545902909
C	1.07001688552590	-2.66014885949967	-6.96550669384408
H	0.05479485454161	-2.40717998943871	-6.69354013877278
H	0.49149706758966	-4.05941250791370	-8.46996534587795
H	2.80705888314800	-4.63045222010564	-9.09078303020135
H	4.69744292191928	-3.55647643545089	-7.92946987595691
H	4.27816920243246	-1.90424740570216	-6.14642759510125

Iig$_2$/I4 TS2 (favored enantiomer)

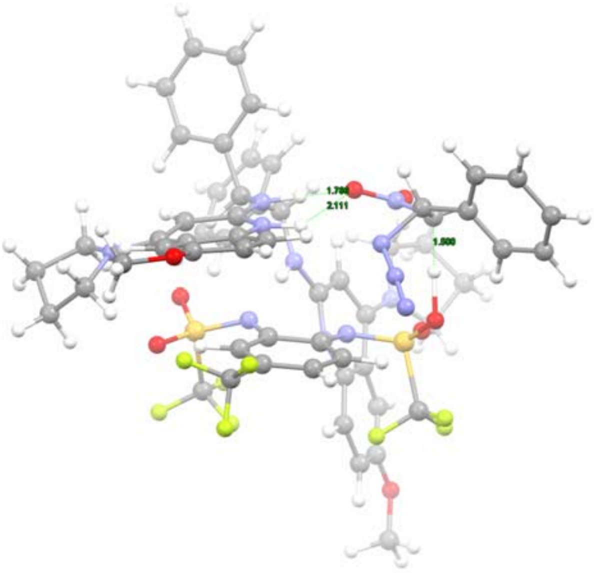

E -5239.429786

G -5238.413497

C	7.32207008182401	2.65288242564852	4.94342476208131
O	6.94984267645006	1.32791083769276	4.63365893008178
C	5.75446270257430	1.10876605339387	4.01983291311869
C	5.44359814914126	-0.22171363037041	3.77821616685338
C	4.25035983669832	-0.60554702095337	3.15843258872030
C	3.87084652179928	-1.97266607256397	2.83448291853188
C	2.53280824234767	-2.20372389219674	2.52720756863550
C	1.62827941737372	-1.15810952851034	2.32205666194178
N	0.34396901296638	-1.31014456335401	1.99660554786941
C	-0.38558959106594	-2.55825152261130	1.91764512541782
C	-1.89090185054310	-2.19819674794793	1.90161413920161
C	-2.73535990178267	-3.45017523606418	1.84048133310133
C	-3.26775447384159	-3.98633537614117	3.00602571316385
C	-3.99789545314982	-5.16109265384556	2.96560152256713
C	-4.20849794256576	-5.80706565916654	1.75872870973686
C	-3.68772333168933	-5.27239036833688	0.59236185194123
C	-2.95135197058667	-4.10109837883594	0.63240125047045
N	-2.12154361511098	-1.31543399358182	0.76835171841357
C	-3.00970484006006	-0.31248793522290	0.74178608634164

C	-3.93363525987357	-0.09043043893675	1.78141295660825
C	-4.78003055327211	1.01566323859417	1.80459689205194
N	-5.48978222975027	1.29866224369692	2.91023499534639
C	-6.01083854631664	2.60927199276424	3.27513960166595
C	-5.83757849072666	2.66692835077411	4.79052830427479
C	-6.13019114406010	1.22493574322200	5.18483523457160
C	-5.37842723020571	0.45954735994784	4.10140160031429
C	-4.90027072818441	1.77042549510174	0.56604221654618
C	-5.93317183996429	2.68034641306640	0.28819096647406
C	-5.98286891075419	3.38065044321342	-0.90340657746097
C	-4.99019556055222	3.16748151741881	-1.87219010125353
C	-4.01446316993935	2.23361149584176	-1.65125211015237
C	-3.96555031708764	1.51406975289779	-0.45365803114510
N	-3.01512395619900	0.52192523036066	-0.31898439105660
O	-6.94473952676606	4.27717962516009	-1.23654363671979
C	-7.92603270561383	4.61358486718116	-0.28028189776844
C	-0.12969911836751	-3.47542311902378	3.09255135989434
C	0.02923726501916	-4.84136595291641	2.89548753475785
C	0.22658709963683	-5.68488859755095	3.97495608414725
C	0.26759105132154	-5.16830892004258	5.25897398154656
C	0.10097434365196	-3.80746481864865	5.46022635980082
C	-0.10280873798531	-2.96173152746239	4.38410190464566
N	2.06365478168450	0.10977051057785	2.42606149110276
C	3.31882869504515	0.41586547782296	2.86568863181004
C	3.64701209890506	1.75395939280236	3.08932170381422
C	4.85466805922705	2.10979809808936	3.64147752491412
N	4.73792140515782	-3.00271250801223	2.84326767220474
C	6.18766406044985	-2.92172075847316	2.73677882077033
C	6.58438107496041	-4.21021840811093	2.01890569509227
C	5.56650807917298	-5.19903502837103	2.57489991135179
C	4.28909690615699	-4.36572487661696	2.57580549769366
H	6.60344175933133	3.12279977291085	5.62219757794977
H	8.28854629002262	2.57485652912084	5.43880137742484

H	7.42588964761847	3.26318284361468	4.04087098615187
H	6.15256162665772	-0.94370617318697	4.13824105676646
H	2.17517731038539	-3.20087520282844	2.36035452372100
H	-0.18534323236815	-0.44406680737950	1.86169374314558
H	-0.12559670507659	-3.07576685500052	0.98074233188326
H	-2.08376739503019	-1.64971074246842	2.83088546526146
H	-3.09966972726982	-3.48228264345073	3.94686547377326
H	-4.40388293460023	-5.57258180357412	3.87783557649659
H	-4.77851496350095	-6.72385531809804	1.72662564274229
H	-3.85193546952745	-5.77106795036518	-0.35178479108765
H	-2.54758819099596	-3.68654793447952	-0.27936996610752
H	-1.52316472613732	-1.44915174108773	-0.06942347012730
H	-3.90837254595099	-0.76714658053271	2.61340794430368
H	-7.07973008314452	2.67016333603302	3.03403435986166
F	0.77095804417712	1.14058726685342	4.85993600968742
H	-6.50218268808609	3.39180731386175	5.25568980221410
H	-4.80226467776513	2.91347676488765	5.02616796575059
H	-7.20179246698605	1.02464025000045	5.12926517058889
H	-5.77345933734330	0.97393748165504	6.18070622808393
H	-5.80435843775909	-0.52742640207809	3.90302858388238
H	-4.32583518067603	0.35534739035241	4.38624998769398
H	-6.72324366864034	2.80898736639490	1.00189813195460
H	-5.03036941851378	3.73585673147412	-2.78708655127077
H	-3.27225145711857	2.02528929957150	-2.40425159248146
H	-2.28822349288707	0.46213682220940	-1.05642707214600
H	-7.47108314593280	5.00557359029309	0.63469059026266
H	-8.56217829030677	3.75542630769460	-0.04084582518785
H	-8.53045830048193	5.39025194374300	-0.74579593824835
H	-0.00424437647472	-5.24471891602877	1.89329857252583
H	0.34773504356826	-6.74558176304110	3.81211789702132
H	0.42413077285986	-5.82530635183235	6.10176542656873
H	0.12390986689656	-3.40160722240288	6.46078971182777
H	-0.25004897283981	-1.90304236924134	4.54029024890553

H	1.48465338561925	0.82852084049195	1.97158636120155
H	2.91479490315752	2.51043017521995	2.85108800062639
H	5.07353411022386	3.15287164760692	3.80264649604203
H	6.48742417737505	-2.02638402207426	2.19139904885296
H	6.64066263256708	-2.91548484954052	3.73475226932591
H	7.61473621835131	-4.49210537609611	2.22325244015382
H	6.45418211852242	-4.09939364668954	0.94185539770371
H	5.83690335405171	-5.48631834823513	3.59145506058230
H	5.46737727293994	-6.09729517555203	1.97003413883529
H	3.57518831537555	-4.68467573002679	3.33907927494541
H	3.80653487696011	-4.41617390788823	1.59287665652219
O	-1.76713290615124	0.06982670912821	4.10621184203275
S	-1.60422603260486	1.40003036886627	3.57008451738824
N	-0.97658566393551	1.33348439203170	2.15005593826075
C	-1.03211010413380	2.36388849593305	1.25381713457376
C	-2.02134549698007	3.33835490081782	1.24419012215921
C	-2.04079346062658	4.33287336972117	0.27450410413144
C	-3.08106617637672	5.40182617316305	0.33270514049694
F	-4.17199631807992	5.07320754008108	1.05017875833077
F	-2.62224420692645	6.54808524034390	0.89753091667466
F	-3.54052757318255	5.77268920900577	-0.87861888355884
C	-1.06852955332304	4.38908117860917	-0.71156401727773
C	-0.05581234084051	3.44664060362156	-0.71539327133868
C	-0.01295253122806	2.42510177601870	0.24260739801049
N	1.01256660247345	1.49833420193147	0.36971190443736
S	2.26920414084491	1.49288187396582	-0.57019393323649
O	1.98353255409953	1.65083863125108	-2.07088556473387
C	3.26078148185729	3.12784545048305	-0.30253526589997
F	3.25632569160769	3.89282524861980	-1.38802040216517
F	4.51899632752644	2.81090905915882	-0.00291796136976
F	2.73256522381366	3.80770008970703	0.71335377788589
O	3.19633805175040	0.41715133163024	-0.22152750250977
C	-0.17235348896490	2.06799491491893	4.64468402217866

F	0.43122823392569	3.11687624881525	4.06338731900670
H	-5.46563102177342	3.40442270007120	2.76648049069259
F	-0.60457081074155	2.47471674272044	5.84640090963686
O	-2.66823931185997	2.32806853010039	3.87164447305016
H	-2.76695263617296	3.32502091571489	2.02038520428248
H	-1.08993541184183	5.16340752159058	-1.46279650296667
H	0.71814028961987	3.50696560642398	-1.46396616944331
H	1.33523057610519	0.63670748784086	-2.56989104211537
N	-0.71342887129514	3.59702758182522	-3.82465064798214
N	-0.89702468389269	2.51918301586742	-3.49676901795002
N	-1.14473183977317	1.38068242594727	-3.10827185614240
O	0.88128021261074	-2.03473126050358	-1.61503221404383
N	0.09861047148283	-1.25437885164570	-2.14839850764703
O	-1.08308576200583	-1.12681041306819	-1.74929129277293
C	0.52625792375048	-0.43595212613042	-3.23694768507329
C	-0.58466778669271	0.25883889022539	-3.99541590032760
H	1.25492119778532	-0.99356558810382	-3.84352449815015
H	-1.44974522066628	-0.42131528882315	-4.09725914599560
C	-0.13855657011590	0.74410993466304	-5.34829266525165
C	1.08768787490849	1.41385411522055	-5.51602873081396
C	1.46322256121095	1.86598575119093	-6.76691186481817
C	0.63866786698276	1.67083514071445	-7.86316121781255
C	-0.57526985839493	1.02306866182694	-7.70957295611914
C	-0.96906241365908	0.56529280598161	-6.46573611373807
H	-1.91888482090977	0.06203677236816	-6.34680662966435
H	-1.22156874832191	0.87542413718234	-8.56211657088251
H	0.94124492191960	2.02822611779112	-8.83604383259703
H	2.40665748436969	2.37821059377096	-6.88459779161109
H	1.72881943202937	1.58019047320384	-4.66206175983689

Iig_2/I4 product (favored enantiomer)

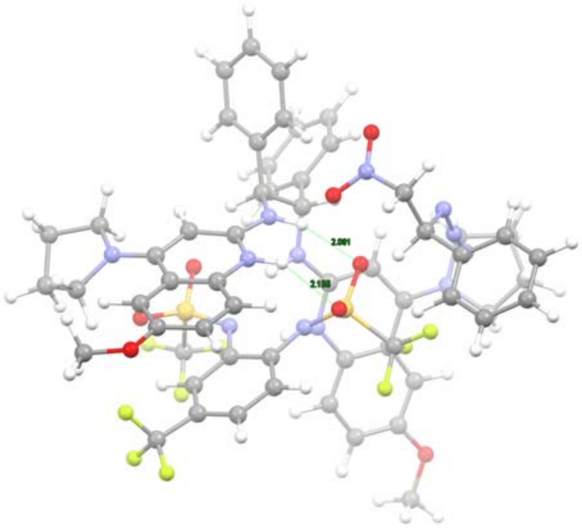

E -5239.486555

G -5238.473757

C	7.16027992074720	2.47015336159397	4.43452090133042
O	6.93132342739093	1.39614527598667	3.54557236765786
C	5.67769825613667	1.21396389880316	3.06351851816918
C	5.50554129679733	0.12558617869390	2.20472716399543
C	4.25415190859503	-0.18310850258601	1.63364582878228
C	3.99361429509538	-1.30815373964969	0.72764117620226
C	2.65703478827942	-1.61817238267985	0.43583146641920
C	1.60357143970849	-0.77823031523665	0.84384618831288
N	0.31385176363673	-0.94639149838792	0.51612531792843
C	-0.22216707548560	-2.03421449549016	-0.27570033317417
C	-1.68463252597474	-1.62615296582857	-0.62488498966427
C	-2.47250542115217	-2.73710324933669	-1.29554484224228
C	-3.27244270990168	-3.59244819978606	-0.52227027047573
C	-3.96312993413689	-4.65166600002458	-1.11962995590592
C	-3.86415388021079	-4.86366001037118	-2.49952489544302
C	-3.07816115247830	-4.00580994742176	-3.27740668349395
C	-2.38748227953011	-2.94629060661054	-2.67993806532137
N	-1.60960918768772	-0.41335482246075	-1.42536554951791
C	-2.46747243482390	0.62411457683936	-1.34967682488601
C	-3.58881651765196	0.65647021116835	-0.50480406216072

C	-4.34748601388353	1.82548889257214	-0.31053816020855
N	-5.32029578810654	1.82290495261577	0.63375114741512
C	-5.87536501200208	2.99242695266928	1.34207116824123
C	-6.32743426254739	2.42647934903481	2.68831544777152
C	-6.75101151959217	1.00414397590522	2.32577896334301
C	-5.65178180061011	0.58327563721878	1.35710616311078
C	-4.04616024277550	2.98077126916619	-1.16269964241702
C	-4.79819344934198	4.18245478097337	-1.20245258989532
C	-4.43302539232688	5.24047734515663	-2.03325011824513
C	-3.31000836611781	5.11558037076700	-2.87962895885503
C	-2.57974020112273	3.94415016481351	-2.88816647291901
C	-2.93935199289007	2.87189303672168	-2.04592763308520
N	-2.18733533331234	1.71717316752984	-2.10106875459703
O	-5.09110358722946	6.41987197326740	-2.09983185088844
C	-6.15815091124247	6.64443523183621	-1.20061203659826
C	-0.16684044329648	-3.36691903564439	0.45116597576460
C	0.18267516672933	-4.53654177018191	-0.23807777057549
C	0.17952877009079	-5.77308281322192	0.41689592361896
C	-0.17076460284817	-5.84760420165551	1.76946621897169
C	-0.51501177421942	-4.68083330677475	2.46343458072420
C	-0.51331366335622	-3.44496684116349	1.80931700366876
N	1.87810797027641	0.31075678231197	1.59583852208847
C	3.14405541275862	0.62685417158273	2.01647281658831
C	3.32598004529311	1.73463758281511	2.86212071887396
C	4.57841039321013	2.03950370393282	3.37827217385450
N	4.98504675598990	-2.06157227390101	0.18117886918604
C	6.37950516407461	-1.64897397483824	-0.06179875110800
C	6.78964391741218	-2.47237310868586	-1.28236714420110
C	6.02315421412697	-3.77688828183423	-1.06670342529585
C	4.66421329703071	-3.26991124950042	-0.59401069683799
H	6.55265978362818	2.37421381160071	5.35619370763303
H	8.22793224548544	2.42266305289703	4.70226105580110
H	6.95223000160230	3.44792025094375	3.95653032505785

H	6.38566013585112	-0.48818846790609	2.02307625835160
H	2.40496466607000	-2.45872027188276	-0.20717031568692
H	-0.29153656436564	-0.13317047475783	0.72002001859106
H	0.33836896755200	-2.12143193885603	-1.23037746932526
H	-2.15923335516037	-1.38463227865631	0.34388139912101
H	-3.34984964750105	-3.42874365715263	0.55993944640552
H	-4.58502521684662	-5.31223377963849	-0.50367657162421
H	-4.40667918412748	-5.69279438155505	-2.96949551235201
H	-3.00226056033832	-4.15650401141658	-4.36104397666213
H	-1.79456579974548	-2.26907260059854	-3.30547067236974
H	-0.76442496283178	-0.29051423280814	-1.99476231275998
H	-3.79144895514970	-0.23007748298359	0.08971794213178
H	-6.74807219668750	3.40219669815672	0.79557456008002
F	0.17449091902937	0.58399876625022	4.33123277779755
H	-7.13514371720111	3.03115433305065	3.13614620576524
H	-5.47098478891033	2.39854799303310	3.38367826171051
H	-7.73505354446568	1.00686963766245	1.81860183788740
H	-6.81327228302148	0.32795296863330	3.19551082953469
H	-5.96519571651129	-0.20124230545824	0.64439853582695
H	-4.76039116688449	0.22886935835678	1.90995679915296
H	-5.68430624121739	4.28396259433117	-0.58471446323885
H	-3.03346586504798	5.96078048454082	-3.51844362640276
H	-1.70094561079221	3.83516777791395	-3.53245585533530
H	-1.35174328830783	1.73440924173110	-2.69816302503517
H	-5.82244032469924	6.55427631804279	-0.14902286207782
H	-7.00035327529584	5.94763242679220	-1.38434875045556
H	-6.50136465673328	7.67504140500049	-1.38453088072904
H	0.45343261482636	-4.47908209957048	-1.30031937804225
H	0.45352876593823	-6.68194293859735	-0.13223597511504
H	-0.17215253387375	-6.81550339337866	2.28507133012430
H	-0.78811595049365	-4.73350753574248	3.52428854908483
H	-0.78585590838826	-2.52813814691785	2.34815445990656
H	1.07831031330207	0.94044585673186	1.79522180252247

H	2.45431488921760	2.34369310916581	3.12770633955095
H	4.68621085262362	2.90486522157817	4.03766643725056
H	6.43774156180600	-0.56087824368148	-0.23158297122539
H	7.02600781912192	-1.90851070290209	0.79934335109098
H	7.88396652297379	-2.60200203349023	-1.34418064351587
H	6.43974886875773	-1.97712144719245	-2.20674505726985
H	6.50675637251786	-4.38333689782974	-0.27737872795568
H	5.93936676103920	-4.39671345005876	-1.97563792804627
H	4.11033735962663	-3.99242915981811	0.03316550833727
H	4.02666792209801	-3.00728041162381	-1.46265288566931
O	-1.97022075097049	-0.38158872485937	2.59923387320501
S	-1.84880525600278	1.08799834545816	2.69924269645392
N	-0.80272013275551	1.60451853216455	1.63309289760475
C	-0.76336690674082	2.91155368508397	1.13005063160519
C	-1.42770685504177	4.02466188209450	1.66868843277254
C	-1.33164473493044	5.28681041443022	1.06286063755796
C	-2.17769744774468	6.39529303902658	1.59472900875260
F	-3.48149913736875	6.27102088998758	1.20937185858756
F	-2.20279112349903	6.43227580296520	2.94298797754513
F	-1.77911043057544	7.60881209976021	1.17177215409293
C	-0.52611980171935	5.47417644475134	-0.06353711328045
C	0.16950231130452	4.38538824335496	-0.59299769960173
C	0.05151907028706	3.09708259705373	-0.03862867015057
N	0.67117890125585	1.94836725578310	-0.51355421535257
S	1.18207753382278	1.71087976939572	-1.95520498964846
O	1.23717795288165	0.25432447407412	-2.25468351094673
C	2.99788494032606	2.15695375474026	-1.97633362446302
F	3.16479455989580	3.44022313064427	-1.64578964830110
F	3.52402462920497	1.96409846912403	-3.19411216200077
F	3.66998572013427	1.40331485100615	-1.10395399346010
O	0.61093395308534	2.55206401067186	-3.05215079968157
C	-0.95826517982986	1.29945326363820	4.32855371141467
F	-0.63619639463953	2.58627047258684	4.51467751239939

H	-5.11287282921679	3.78252768512023	1.44438732803669
F	-1.71423630910317	0.89909222105765	5.35061775373554
O	-3.09479907571439	1.86203750298202	2.88508259474967
H	-2.05315021726154	3.90060622374764	2.55499225593707
H	-0.44122487949618	6.46000598818126	-0.53163350610105
H	0.79809972878846	4.52604431854349	-1.47606468237891
H	0.69576342607665	-0.67763444596698	-7.07589009932944
N	3.95730147958333	-1.04120713871535	-3.30433815993180
N	3.15351837109212	-0.90749529184276	-4.10399285278525
N	2.30208680531227	-0.92789908505193	-4.98205686851485
O	-0.95693445739480	-0.06843204486367	-4.41205512497062
N	-0.48497233212254	-0.71321023503962	-5.33818872871838
O	-0.71895844295213	-1.88547015685845	-5.56946678103341
C	0.48131793708559	0.00099732400571	-6.23845975148367
C	1.73795212064009	0.35399584507662	-5.44612990223129
H	-0.02292964246230	0.91867131125670	-6.57824605192382
H	1.42646351357215	0.95864236257531	-4.57741482461306
C	2.69503274678907	1.14975741936623	-6.31240111331304
C	2.73432197386612	2.54692102619531	-6.19003786300747
C	3.58083235248033	3.29996008643839	-7.01004303635151
C	4.39494775355504	2.66259203995039	-7.95310683314256
C	4.36141900953704	1.26821441889042	-8.07399149564014
C	3.51323662311474	0.51333738059587	-7.25830177724911
H	3.49192840683730	-0.57982532638791	-7.34631861420458
H	5.00164995720187	0.76470045611042	-8.80814551009067
H	5.06213886268377	3.25341341365467	-8.59186102838655
H	3.61095071337652	4.39089432249051	-6.90457903032143
H	2.10581283275713	3.03921754190821	-5.43774825066870

Iig$_2$/Tf$_2$NH with hydrozic acid and nitroalkene

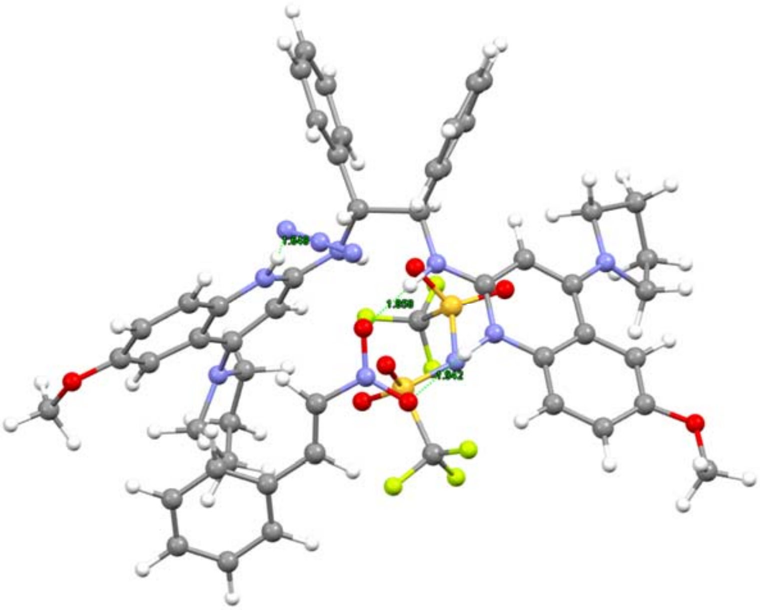

E -4615.598612

G -4614.68157

C	5.97510173363610	5.33902215782727	-4.29900029574500
O	6.18972084734888	4.34942717044098	-3.30894493491700
C	5.16623009740943	3.50908156857374	-3.01996741524196
C	5.42803591814215	2.53641615332213	-2.06282723844939
C	4.45575257533578	1.60339406734765	-1.67554026672946
C	4.67174678876532	0.53238534265516	-0.70944889213816
C	3.72616812987060	-0.49428679290447	-0.65992962211237
C	2.49040310158306	-0.38833722336544	-1.30978662268740
N	1.51245985005481	-1.29728370349053	-1.19159037249766
C	0.19506837007764	-3.02968866296384	-0.10084390021854
C	0.24053615823377	-4.36170402016067	0.62278312679971
C	0.64851111308285	-4.42966138741573	1.95726204647390
C	0.74538673748331	-5.65934297920457	2.60043124458820
C	0.43270051479854	-6.83228120454647	1.91467446113380
C	0.01565699518593	-6.76756498232616	0.58794095294714
C	-0.08194557391403	-5.53654618369719	-0.05635423813286
N	-0.48544908548012	-2.01051517784308	0.67640177022467
C	-1.80356974832960	-1.75188626267522	0.68464170655044
C	-2.33218082736143	-0.90874632457666	1.68189586002056

C	-3.65374794816412	-0.48733474444326	1.65478195526815
N	-4.12862127544346	0.42395437566333	2.54930821550065
C	-3.31937438402884	0.83481943790814	3.71118650298817
C	-4.00723231381355	2.10008953871534	4.21305116147771
C	-4.53884956490314	2.70700832743261	2.91714082285669
C	-5.10037269301745	1.48066217478187	2.20820803526664
C	-4.53173090743922	-1.10344777881842	0.67211292825470
C	-5.93365057514109	-0.95537790307749	0.68291834398403
C	-6.71698654830411	-1.55126518636819	-0.29192575401580
C	-6.11552415822551	-2.33272208700624	-1.29423779587798
C	-4.75606949039369	-2.54348031832293	-1.28696683144298
C	-3.95111072957359	-1.95324861467042	-0.29504330734080
N	-2.60451407007412	-2.23515753855564	-0.28271915130318
O	-8.06600166211298	-1.42556290240862	-0.36988612281427
C	-8.71587090786999	-0.70146593682761	0.65771133616346
C	2.38775483850269	-3.60583335530144	-1.19299633553500
C	2.05882630488793	-3.90346006244425	-2.51730551136477
C	2.72232780877559	-4.92558683621915	-3.18813505488450
C	3.71927961698339	-5.65661507578690	-2.54400252182706
C	4.05288840880224	-5.35953513352438	-1.22523662550177
C	3.38860844016334	-4.33611480072744	-0.55334774760612
N	2.23955703331061	0.70337002276107	-2.06559002687459
C	3.20201093785066	1.64931435820864	-2.33126714855385
C	2.94127117549770	2.63479845183788	-3.28726805110299
C	3.90251513012088	3.56763000053896	-3.62723608854828
N	5.75109592571042	0.48332813408650	0.09767134057626
C	5.93850753817823	-0.65718001000735	1.00330759137643
C	7.16595555571531	-0.28103252130600	1.82040927676931
C	7.01265237932532	1.23213378883199	1.94375528420403
C	6.56001222219079	1.63199691855450	0.54362427348156
H	6.91214452994445	5.89085557503747	-4.38234865166324
H	5.17569157372129	6.03328138446763	-4.00968720906514
H	5.73485408850031	4.89149534814059	-5.27202952095330

H	6.42733632239831	2.51326189397257	-1.65465871005675
H	3.88870719009851	-1.35755261495851	-0.03644440973407
H	2.13340240015230	-2.33012093214558	0.50802542200330
H	-0.33454578622139	-3.18155583853062	-1.04946530179153
H	0.89015876648818	-3.51642089616628	2.49639433865473
H	1.06501822553629	-5.70315829301167	3.63768299719303
H	0.50882336776404	-7.79257755867754	2.41640516146236
H	-0.23829027341370	-7.67720601067044	0.05150499600411
H	-0.41948935935371	-5.47562108798516	-1.08789352974925
H	0.08161465447824	-1.47442144353624	1.32960599215059
H	-1.63301719390066	-0.52036711085251	2.40956929201322
H	-3.28725579902720	0.02499759519168	4.44895052231203
H	-2.29630348211403	1.07611407683015	3.40815595443889
H	-4.83911956911294	1.85560427395371	4.88436840527137
H	-3.31294719703155	2.75138890651051	4.74918556906459
H	-5.29454211783741	3.48308913578041	3.06550174078858
H	-3.70910293727433	3.12417243633785	2.33490331313477
H	-6.09912544127827	1.25075270093904	2.60688817183425
H	-5.17885116192312	1.59547391252525	1.12506938161872
H	-6.39557894889119	-0.38113038141119	1.47053214500527
H	-6.74590958728218	-2.78390513591618	-2.05433771028193
H	-4.27876789502100	-3.17528042790159	-2.03053384604952
H	-8.55186296937385	-1.16552414451175	1.63904853053823
H	-8.37854449276402	0.34360918460960	0.69180617537884
H	-9.78036326437548	-0.72682878539924	0.42139062987436
H	1.27643135896102	-3.34605678477885	-3.03007205516306
H	2.45233315079288	-5.15342155388348	-4.21518466688971
H	4.23443438508543	-6.45569350391811	-3.06905714240780
H	4.82847086844424	-5.92607891436010	-0.71787134230088
H	3.63731569420909	-4.10998770630307	0.48134741651285
H	1.97004325372237	2.65350948480003	-3.77172597674823
H	3.67042832602060	4.31605845811952	-4.37519481020040
H	6.05344421367334	-1.58305070673592	0.42995483981822

H	5.05521900298246	-0.74507353433886	1.65326230778977
H	7.18840393679641	-0.79657873576661	2.78321114092737
H	8.08296579935196	-0.53112534229395	1.27399903289136
H	6.22623833806022	1.47026975313246	2.66603380953568
H	7.93088346831386	1.74686557006852	2.23702768664050
H	5.96397352509962	2.54693652647590	0.52962670915476
H	7.43423812592833	1.76871318787762	-0.10599986623915
O	-0.93117963439898	2.23223754522257	2.13476764542900
S	0.01477652963227	2.00602693558111	1.04634643077542
N	1.55070720555168	1.92899252485350	1.51219915628971
S	2.01440676175412	0.80337368889201	2.53576682136395
O	3.45333716317510	0.89737276697276	2.70055336211894
C	1.33957605949967	1.29275364789559	4.22734886169553
F	0.11912451242023	0.79441672126992	4.42377520328988
F	1.29842314499967	2.61515674015563	4.35702370806822
F	2.14828064767418	0.79262638926383	5.16376712657396
O	1.42368148333544	-0.52067321521487	2.31323083115368
C	0.10146185069333	3.64779969693810	0.17192761183732
F	0.84346413044526	3.57690153989827	-0.92747370715176
F	0.58288850916095	4.59544016088146	0.96615779407114
F	-1.14943714781790	3.97771229481976	-0.18608688338780
O	-0.34505251910273	1.03867268827204	0.02148415149589
N	-0.82742101694195	-3.08680169207907	-4.39166441686639
N	-1.39962547899643	-3.40375254676331	-3.42909362895684
N	-1.98606215234907	-3.74648412210754	-2.45160144677990
O	-0.48715156037100	1.37905806278061	-2.97704578732897
N	-1.27077316352856	0.47233298572065	-2.67984103146894
O	-0.93259867995203	-0.71263153272997	-2.59280666342273
C	-2.63690303505251	0.75758461360997	-2.40630124675851
H	-3.24538888210457	-0.13640233107738	-2.36974272811436
C	-3.01898481798219	2.01223534012200	-2.11115739367507
H	-2.25007678411436	2.78291798748357	-2.09475294136581
C	-4.35830512920526	2.42322595310361	-1.75201342238947

H	-3.69174788115957	4.39673783495718	-1.20135918677444
C	-4.54885893083214	3.73364059177396	-1.27770565102309
C	-5.81005952192813	4.17273196531933	-0.89884478407014
H	-5.94223979845068	5.18456343389467	-0.52852335281699
C	-6.90373817729496	3.31442527747965	-0.99824171718221
H	-7.89202297269451	3.65815555233767	-0.70760267685201
C	-6.73015933238951	2.01339396104105	-1.47458420822367
H	-7.57967197565241	1.34086973159624	-1.55044313259603
C	-5.47060486873005	1.56693822461279	-1.84372427870430
H	-5.34929562335839	0.54963525784778	-2.20249297024666
H	1.30616592028610	0.84048909273352	-2.46292038383577
H	0.64554143399950	-1.12434095468064	-1.70375112901070
H	-2.24644976035212	-2.81084317123287	-1.12033951531445
C	1.62195485277160	-2.53764007956406	-0.44083447830572

Iig$_2$/Tf$_2$NH TS1 (favored enantiomer)

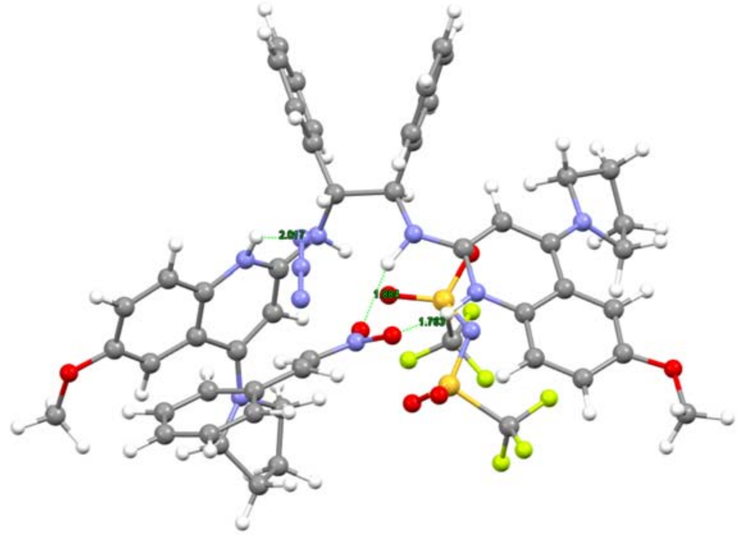

E -4615.559225

G -4614.641073

C	5.64269715177443	6.03993714879705	-2.89724642459680
O	5.92656295526665	4.78175942489724	-2.32502148686736
C	4.90759116656578	3.89462370665277	-2.14845697662683
C	5.26859107005036	2.68837882956190	-1.56469115570661
C	4.33788708991683	1.67649928573372	-1.30772605884154

C	4.61375723192976	0.40875643396277	-0.65514929814009
C	3.64127692708693	-0.58944443139510	-0.73350205696413
C	2.32850743148176	-0.30224739411160	-1.13507510968530
N	1.29114198277160	-1.16039562400314	-1.02789768826253
C	-0.01298785883593	-2.99322082612999	-0.08373827466865
C	0.06680712294405	-4.46790996702651	0.24677038538981
C	0.47373720698482	-4.88736176562095	1.50658453219451
C	0.61536819119296	-6.23690999619097	1.77854612144600
C	0.35787423918307	-7.17655504272149	0.79408580410548
C	-0.05608876366049	-6.76329619185246	-0.46079685383018
C	-0.20446054711733	-5.41463505087181	-0.73235517947680
N	-0.64702929906008	-2.23912021051369	0.99233777851628
C	-1.94002827171749	-1.84338971031590	1.00613943649289
C	-2.33832494651300	-0.91755787599331	1.98674616038353
C	-3.61424975827692	-0.36678090195101	1.99446319198014
N	-3.86893698238034	0.73564183992697	2.72220034485281
C	-2.80977449375542	1.36348318301994	3.51089149773806
C	-3.41068399770603	2.68148873733901	3.98686983500970
C	-4.33320064900998	3.04051559699217	2.82786108786669
C	-4.94885062502090	1.68398529789437	2.49007589743705
C	-4.62461367382013	-1.04927455291892	1.19445649468419
C	-6.01098623948273	-0.87921829251751	1.34696165006929
C	-6.91198630826028	-1.51633672915514	0.51144377752720
C	-6.43794022836231	-2.35129599843888	-0.51386314136749
C	-5.09447835866721	-2.58387130644791	-0.63811061023650
C	-4.18181192349524	-1.96823747640304	0.22533363395343
N	-2.84738689472189	-2.31827255205107	0.12481889211327
O	-8.26002293083992	-1.39530133755866	0.58592075129018
C	-8.82487044588539	-0.59037962201248	1.59866303185113
C	2.21261505162405	-3.41326062019211	-1.16974728159756
C	1.97935499625346	-3.50328394983034	-2.53612677182138
C	2.68015199160871	-4.41546343054413	-3.30487119055228
C	3.61616043093811	-5.24837203875983	-2.71489117341731

C	3.85278685287854	-5.16242503990074	-1.35330810049988
C	3.15522686139881	-4.24780830903936	-0.58384970465704
N	2.06656575980691	0.91603438972375	-1.67080456464962
C	3.01220235249219	1.90601826940673	-1.77233304401429
C	2.64404089466740	3.13669839475465	-2.34586983086724
C	3.57683234648552	4.13010775723340	-2.51400352371798
N	5.76142176397199	0.16525532911672	0.00357280650638
C	6.00292374591455	-1.13283391272797	0.62842050772796
C	7.29161239532278	-0.94156528947487	1.41943973149782
C	7.14708362846387	0.50284202817301	1.88127484300777
C	6.60999021908228	1.17719767476030	0.62035655185452
H	6.59591035488605	6.56583537250295	-2.92801268408940
H	4.93436546947195	6.60820540233673	-2.28645495679632
H	5.25099309956392	5.93893832171274	-3.91420357142137
H	6.31499458452807	2.56347297554304	-1.35509398410008
H	3.83716646067371	-1.56538462904577	-0.33371544911020
H	1.90697829047670	-2.29408441974303	0.64540604068134
H	-0.55969202377722	-2.86476195565703	-1.02727656511833
H	0.67703542164657	-4.15104920032261	2.27024789223769
H	0.93443184887793	-6.55576727519449	2.75993677686047
H	0.47796351393652	-8.22882225800915	1.00509463182892
H	-0.26213695867945	-7.49135188493703	-1.23128379207659
H	-0.52128600386287	-5.09192478897390	-1.71364483254645
H	-0.02017546380088	-1.63434364625823	1.54029533207880
H	-1.55210522714217	-0.53001867531911	2.60780545725284
H	-2.51791971154581	0.70909628725456	4.33523524042953
H	-1.92644839480917	1.56044169720066	2.89200634020495
H	-3.98806719172352	2.53856436632602	4.90175236765182
H	-2.63791273402444	3.42495156576979	4.16386393719751
H	-5.08667621589323	3.78109982145919	3.08735846556443
H	-3.74387067859451	3.40827302484803	1.98691626553165
H	-5.77856342462537	1.48602444919847	3.17965760933502
H	-5.30543339347880	1.63405638010689	1.45963288684960

H	-6.37380952787827	-0.27870430875624	2.16005518126227
H	-7.15276306320160	-2.81746495161738	-1.17212742540276
H	-4.72147380015425	-3.25335813436410	-1.39821428484229
H	-8.56906996045869	-0.96373442686266	2.59474087028556
H	-8.51013381107843	0.45361186071490	1.50207398602580
H	-9.90187958047514	-0.65770195979967	1.45477811188650
H	1.24884986675684	-2.85105703245796	-2.99148896588362
H	2.49255679949820	-4.47741493046099	-4.36680210409175
H	4.16081802148651	-5.96171234792881	-3.31554779291258
H	4.58266715991588	-5.80838523885269	-0.88844859587137
H	3.33896574773971	-4.18213277276439	0.47925293168507
H	1.61546100018088	3.27755055856679	-2.63848725953595
H	3.27258033971688	5.07259032031870	-2.93942460477634
H	6.08404180460242	-1.90848874748371	-0.13670748833400
H	5.17885432393456	-1.36831761421428	1.31118659334720
H	7.37350458292679	-1.64782121603978	2.24200392430770
H	8.16294762258389	-1.04748087463143	0.77138127698490
H	6.40896093394352	0.56491371872073	2.68168570894392
H	8.08161581734495	0.94677732983418	2.21781779796368
H	6.04027079759326	2.08078175537503	0.84035632019624
H	7.44929736992122	1.41745493495614	-0.04282488072017
O	0.14974884678377	2.65422690891623	2.21004891183289
S	0.99316999990977	2.68367470414051	1.04922936619986
N	2.12214483010740	1.57998232386608	1.08529343176055
S	2.00956028164788	0.48346160280430	2.19558120990640
O	3.15917174026717	-0.37170516326646	2.17678428681780
C	2.15161513841103	1.41165116408856	3.85004198646539
F	1.02276705841398	1.39250648944671	4.55205218933660
F	2.51843337497937	2.68378125322263	3.66907128402552
F	3.09456383182892	0.84514021785909	4.61480783157468
O	0.75123990359207	-0.20321245144068	2.30358572202536
C	2.07036587257909	4.25272917643271	1.24485120806269
F	1.94980476184638	5.05419103230895	0.18053384489273

F	3.36976195965585	3.96659262112580	1.36578834790112
F	1.71568769040810	4.97624802855025	2.31200964079379
O	0.37690146584218	2.91495305821298	-0.21976410079151
N	-2.80182689989785	-0.50554190685544	-4.05279628436263
N	-2.30711274649313	-1.31070625477661	-3.33028984862595
N	-1.81715457663128	-2.05686105246981	-2.57068285552710
O	-0.60482035392350	1.17109331786008	-2.42914418232546
N	-1.35764041680364	0.88752533645726	-1.45750539913953
O	-0.87926243340941	0.57556014137961	-0.32585124753792
C	-2.71550099632398	0.88474132885548	-1.63922396586475
H	-3.26290414251803	0.56653598775396	-0.75393905797752
C	-3.25741968463125	1.09085230794804	-2.91963219051857
H	-2.62911432830070	1.67312766779423	-3.59874672462756
C	-4.71373712080303	1.18067185680227	-3.11757624801406
H	-4.49870732396908	2.19043896603262	-5.00407208970379
C	-5.20431471208093	1.79628366150290	-4.28724373135749
C	-6.56214813708309	1.89000493869350	-4.51622451973976
H	-6.92224637957012	2.36712099721950	-5.41605502482283
C	-7.46344766384601	1.36707978316517	-3.60159448998217
H	-8.52479841137752	1.43574746537651	-3.78866278018282
C	-7.00024824123705	0.74763304599752	-2.45154245567546
H	-7.70169430880875	0.32699105131775	-1.74574974994460
C	-5.64508963297695	0.64931925089715	-2.20463281794414
H	-5.30056792754211	0.15056440974010	-1.31380042853207
H	1.07532363851540	1.15163806783952	-1.89688807661544
H	0.36699748591317	-0.70780501202949	-0.91538646217072
H	-2.53610034362114	-2.68122997104995	-0.79257328046094
C	1.41760720707482	-2.43643328494597	-0.33151596484498

Iig$_2$/Tf$_2$NH intermediate (favored enantiomer)

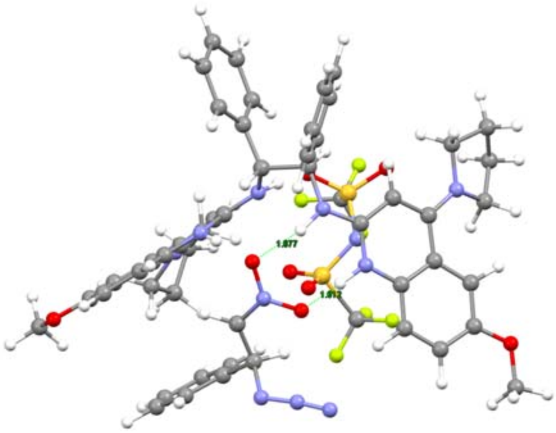

E -4615.589291

G -4614.673848

C	0.84632590542360	-7.85518170845111	-4.45676396753874
O	0.06003764698739	-7.47855206368225	-3.34595440704600
C	-0.40677114173935	-6.20628787601055	-3.29130885719434
C	-1.17879362002335	-5.88752322127369	-2.17243164438659
C	-1.72441846676619	-4.60076825022920	-1.98688500632990
C	-2.56395173799488	-4.19061180629442	-0.85792031682029
C	-3.19820459078746	-2.94027743308652	-0.93426101361765
C	-2.87706325055082	-2.01917089503550	-1.94974076218721
N	-3.29700176428568	-0.73730255658415	-1.97591919211408
C	-3.97419809542357	-0.07541903821411	-0.88423886142662
C	-3.39903877043211	1.36442229782477	-0.78294168477102
C	-4.10086812279192	2.16060787222283	0.30380867936226
C	-3.80220345443156	1.93443465919780	1.65683364783956
C	-4.49845166541435	2.61890335290651	2.65714904209250
C	-5.49908123328077	3.53689662260122	2.31613292832590
C	-5.79604998819312	3.77084115200003	0.96935735564045
C	-5.09833562888679	3.08643942828892	-0.03108410616500
N	-1.96527107853512	1.25390852189068	-0.55442998968931
C	-1.04810717732604	2.18402797734111	-0.90597482295588
C	0.11845842369877	2.37791281597555	-0.15307661058525
C	1.13220210146028	3.25528018863272	-0.57344089795153
N	2.29489245425493	3.29384583014946	0.12253469512006

C	2.45764283368849	2.47751570827549	1.34166769519846
C	3.89053826731491	2.75117803873667	1.78613858107505
C	4.60382003533642	2.97246344925555	0.45404073558432
C	3.59597369940501	3.81908804744461	-0.32103747845138
C	0.83584332351929	4.12061705357855	-1.72017499228655
C	1.64755781277391	5.20035349852915	-2.14988836065212
C	1.32125015185384	5.94766267428779	-3.28030576234698
C	0.14560043767378	5.65359694022197	-4.00393548543796
C	-0.69375037487706	4.64668800514561	-3.56992036312144
C	-0.37091372949009	3.87865949807157	-2.43126698517175
N	-1.25275690236567	2.89291407371167	-2.03880158615610
O	2.06506917528272	6.97232331197582	-3.75606047039978
C	3.27348995318724	7.28492008677134	-3.09591793127170
C	-5.48404531013936	-0.06970952771965	-1.03224482583592
C	-6.06499530041224	0.19328608419608	-2.28169535701550
C	-7.45491041053097	0.26267016613984	-2.41243347504148
C	-8.27669265849722	0.06723480713803	-1.29556445978554
C	-7.70227542528252	-0.20284784803327	-0.04877758750506
C	-6.31121610495199	-0.27177895429086	0.08071180836844
N	-2.05923943438839	-2.39589518634025	-2.94838522775741
C	-1.50154773226250	-3.64459498835219	-3.01873648112989
C	-0.71670399974121	-3.96690654232876	-4.13969284220213
C	-0.16180056289571	-5.22997641553996	-4.27934252584336
N	-2.77206127094820	-4.97662452851811	0.22898520543751
C	-3.75017892822798	-4.58756551897637	1.25552531746811
C	-3.61454725588408	-5.66957882427922	2.32206877049036
C	-2.12219078167562	-5.99343499722770	2.25912210837074
C	-1.85072658429033	-5.99989073401180	0.75500943007275
H	1.11195135058026	-8.91274601095568	-4.29881719481447
H	1.77610996262623	-7.25572580812789	-4.52113601960160
H	0.28350404078616	-7.76249362198269	-5.40683084603225
H	-1.35773446408048	-6.69532881851419	-1.46588563601305
H	-3.87465329329336	-2.61820570065996	-0.14648243476305

H	-3.71252685509539	-0.58080394740952	0.06433630256700
H	-3.57741982549163	1.85711223841018	-1.75323912424924
H	-3.01968754616278	1.21651493485561	1.93476219437055
H	-4.25779324102377	2.43371083163485	3.71078695590460
H	-6.04492015675289	4.07286021304723	3.10164321486333
H	-6.57530191021092	4.49153606592609	0.69447997505158
H	-5.33761753463592	3.26336384171943	-1.08703642605113
H	-1.67510989839699	0.63354937457151	0.21215992007109
H	0.24054512045185	1.74524712739281	0.72489217320203
H	1.70189008111519	2.76399487588229	2.09593503665427
H	2.31350373868947	1.41043603564682	1.09346247490653
H	3.94107560629348	3.66705365656979	2.40523604838104
H	4.30281468067892	1.91418353974652	2.37479753291792
H	5.58272934918711	3.47379127057292	0.54643714277796
H	4.75236956169290	2.00416115860863	-0.06021543578086
H	3.70776684166179	4.88742593173615	-0.04499511205575
H	3.70505640750769	3.71775009271272	-1.41260540700031
H	2.53074673322332	5.47078953804036	-1.58009903248184
H	-0.09325091857171	6.24999173004173	-4.89042420801735
H	-1.62558078259037	4.42996562092349	-4.10415726858610
H	3.96783971092557	6.42091218149270	-3.09255875476968
H	3.72843712669193	8.11205008208206	-3.66357201605123
H	3.09475101905862	7.61829945759329	-2.05424442274589
H	-5.42064448556148	0.33949079630188	-3.15786190874515
H	-7.90014832874958	0.46840057516664	-3.39317620390973
H	-9.36699232549509	0.12033818946040	-1.39916019049661
H	-8.34034396395393	-0.36283890425240	0.82843442683929
H	-5.85858154254479	-0.47566034426577	1.05954559774654
H	-0.55296219718639	-3.20256120049363	-4.90808705578526
H	0.44500955452616	-5.44863919382883	-5.16205542408055
H	-4.76158243944817	-4.52159848321769	0.81360022884545
H	-3.47804649061496	-3.59936374651549	1.67232138812799
H	-3.94699919820927	-5.31508863718338	3.31270258512331

H	-4.21669455854374	-6.55863730797532	2.05310761492180
H	-1.54091359868230	-5.18602956486169	2.73729591694718
H	-1.85593652697129	-6.95601332473491	2.72934283094829
H	-0.80826537242537	-5.74329409691022	0.50244132307847
H	-2.08447765882924	-6.99779281252232	0.33530390580830
O	1.88576339171516	-0.39188861008395	-0.26432440304660
S	1.00666411806036	-1.33280182427522	-0.98140610407261
N	0.05989559338430	-2.22794947135016	-0.02580656997207
S	-0.40868749737249	-1.75938044692750	1.42516071225377
O	-1.22714775743091	-2.82177615783017	2.01043093294409
C	1.13839592050870	-1.72001915289470	2.51507287283390
F	2.06729093283216	-2.53519405337994	2.02721866094278
F	0.79809056252272	-2.13102196151424	3.73486404090268
F	1.63582498339590	-0.49064857190353	2.60656388300109
O	-0.91087116972951	-0.36716368263420	1.51549282639010
C	2.15306859151589	-2.69430888892565	-1.55387852112113
F	1.48218705106952	-3.76788561538110	-1.95078888020700
F	2.97360034266816	-3.03538352198994	-0.56534530202279
F	2.87613580208294	-2.23361506715033	-2.57774391752597
O	0.31896060031642	-0.87652843666739	-2.19436616068231
N	0.97177383194394	0.20690777169275	-6.55559036108093
N	0.59024466999451	-0.91742361112312	-6.83477883064972
N	0.27338974044982	-1.95138220093717	-7.21241645438376
O	-1.47475484075728	-0.55128372839344	-4.78507479662830
N	-1.35758545274295	0.67284948328057	-4.44665014050377
O	-2.37661440412611	1.25231581349888	-3.87666901670904
C	-0.23204477600050	1.32681375086841	-4.68399425723666
H	-0.21520796296863	2.39794422020290	-4.49219111089003
C	0.97772723491269	0.56693254652340	-5.08869821002661
H	1.00063158933539	-0.37474031360234	-4.51094938261784
C	2.21372888897821	1.38313594032899	-4.78638429484802
H	2.70046008939507	0.24509811746320	-3.01235075276047
C	2.98728363350575	1.08234565422079	-3.65659509585825

C	4.11203371545064	1.85390324029307	-3.34683542604420
H	4.72053665994022	1.59535692928665	-2.47190259720897
C	4.46330655453558	2.94276061937800	-4.15282883844945
H	5.34732822990843	3.54567466039796	-3.91130833484907
C	3.68263596132376	3.25714723889785	-5.27235382746135
H	3.94920569834944	4.11127218412648	-5.90659612347843
C	2.56550735228963	2.47852381129415	-5.58978718305102
H	1.96293944777410	2.71262902088069	-6.47563343121380
H	-1.78839449588771	-1.66682670004076	-3.66391050664084
H	-2.96177621688204	-0.12538328212775	-2.74428808482067
H	-1.84689270157181	2.42504856874143	-2.78476515948397

Iig$_2$/Tf$_2$NH TS2 (favored enantiomer)

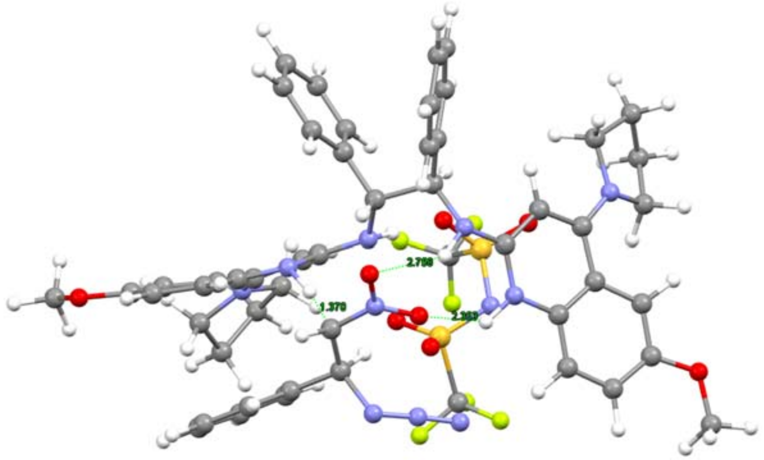

E -4615.557119

G -4614.639889

C	0.43582467880767	-8.89573264602519	-3.54236937525532
O	-0.41715411370738	-8.31208997854920	-2.58065489233612
C	-0.66147849232463	-6.97655808840563	-2.65417975606221
C	-1.53276933859775	-6.47424308229990	-1.69777444302217
C	-1.88964721537500	-5.12254826022537	-1.65158269802336
C	-2.76635172261364	-4.52263307478272	-0.65768014607155
C	-3.26149384283964	-3.25196971443332	-0.95352491405837
C	-2.77601420457027	-2.48050163634393	-2.02352402672452
N	-3.27587905077523	-1.26798692389455	-2.30385343572624

C	-3.61794791386831	-0.30978065442978	-1.25757533794449
C	-2.84868342047857	1.02736562546296	-1.45652324954844
C	-3.34854126645743	2.03158480018174	-0.43680586975119
C	-2.99768826714653	1.90003026398478	0.90191492112656
C	-3.45915224962433	2.81032081960451	1.83608459205803
C	-4.27569318568395	3.85806196646061	1.44326546120908
C	-4.62481017509593	3.99518665258787	0.11082509609241
C	-4.16057603141621	3.08778529839526	-0.82571768586481
N	-1.39931234154374	0.82772920573080	-1.32584126604089
C	-0.59360216221150	1.93879888982274	-1.39120350096684
C	0.45282888730941	2.10549674263825	-0.46882599938761
C	1.33233900588758	3.18170973918887	-0.56816955111306
N	2.44660804339661	3.21283156852742	0.19289879991323
C	2.76590226590519	2.09704713285368	1.07922163461375
C	4.17674351543592	2.38835607513395	1.58235132349250
C	4.79019703808642	3.13391251027415	0.40324079603768
C	3.63056568285951	4.02572371596763	-0.03534776622179
C	0.95141229283943	4.24114567746209	-1.48093392290862
C	1.49139720571634	5.54050349439315	-1.47165456835347
C	1.13007770741258	6.47956723211737	-2.41586816911919
C	0.18743027663982	6.14559679370697	-3.40287806796903
C	-0.41309995510357	4.91947192670975	-3.38206832516983
C	-0.07017916901856	3.96147642980962	-2.41471937872993
N	-0.79438475070428	2.79913637107783	-2.39266661461162
O	1.61301939042549	7.75043330511480	-2.47869000847368
C	2.57748441947342	8.16366334847047	-1.53711161910103
C	-5.09991231610478	-0.00682640137879	-1.22979212891673
C	-5.77130957958245	0.33572354979149	-2.39722469812479
C	-7.11383498562308	0.66981628437588	-2.35523002849114
C	-7.79657517387427	0.66931092668160	-1.15024261601695
C	-7.13334725876025	0.32452829783560	0.01504006812438
C	-5.79223654227188	-0.01429981095816	-0.02593627299503
N	-1.84283815719398	-3.00200721412366	-2.83834837655126

C	-1.39999202876442	-4.29930026250499	-2.68940135541856
C	-0.49733502133195	-4.79789116797387	-3.62758117206054
C	-0.10908173490473	-6.11577792585572	-3.60767901090590
N	-3.16201809844295	-5.14786873825180	0.46174572830481
C	-4.05959858807875	-4.48402871474269	1.40248193704044
C	-4.11969535788719	-5.41884418723253	2.60446016909556
C	-2.70576554002314	-5.98432632759210	2.61633555360177
C	-2.45563316036505	-6.23169494650186	1.13076362604571
H	0.47110125160449	-9.95545862300936	-3.29431306402055
H	1.44532796068154	-8.47666938564204	-3.48992117471382
H	0.03961113264180	-8.77207679088309	-4.55490341587242
H	-1.95375161719429	-7.19302628246446	-1.02007742902254
H	-4.02453999744873	-2.79948535580223	-0.34496880215671
H	-3.30174136647890	-0.72189846584354	-0.29215618992735
H	-3.07630983314308	1.41398269536492	-2.45675716286474
H	-2.35994488591295	1.08296974755894	1.21011557191385
H	-3.18171473058197	2.70037319357086	2.87447764236586
H	-4.63733364732870	4.56632134009583	2.17430931077622
H	-5.25794005719864	4.81227452875728	-0.20227880231982
H	-4.43304068577958	3.19611048835056	-1.86563154509027
H	-1.16004858035703	0.13354205622055	-0.60787726790723
H	0.57894918214783	1.32894644519862	0.26390529659925
H	2.03743877935765	2.03744068346352	1.89144283662907
H	2.73690502355552	1.15262167191468	0.52337073631198
H	4.14466761812957	3.03564429868608	2.46006531930113
H	4.71164264634805	1.47676970638251	1.83772090953573
H	5.67481323088865	3.70858852856163	0.66973259435932
H	5.04861735725693	2.42999042937493	-0.38913582802797
H	3.60358979914639	4.91794259959432	0.60340371910010
H	3.70431107451477	4.32458605802720	-1.08136232936406
H	2.15488627850885	5.82423356177784	-0.67645551289875
H	-0.07209119559061	6.89006576842577	-4.13829835477522
H	-1.18586206735917	4.67362728440797	-4.09317132870112

H	3.47511743120076	7.53865527904430	-1.58208020841915
H	2.83487867530591	9.18501019545615	-1.81334655175832
H	2.17399392207334	8.15605691813687	-0.51951575892521
H	-5.24341292322776	0.34907209504102	-3.33894040377831
H	-7.62591761237197	0.93912672489199	-3.26733714851796
H	-8.84218753019342	0.93834102299928	-1.11932018001526
H	-7.65955239062738	0.32251867503118	0.95833088098429
H	-5.27346575202406	-0.27218001123170	0.88678800726065
H	-0.11358467543833	-4.12962690485385	-4.38314711601490
H	0.59407964186033	-6.46593350797339	-4.34517026372014
H	-5.03799898030321	-4.32380660994956	0.94298684818941
H	-3.62833997917818	-3.52156268305602	1.69996145846051
H	-4.37865726285171	-4.89113031947409	3.51934805803602
H	-4.84707915395629	-6.21456681438725	2.43649025349029
H	-2.00355261389530	-5.23234110599483	2.97605532066231
H	-2.60626033034565	-6.89152630461162	3.20810815390551
H	-1.39414766978699	-6.20025018714284	0.88908587873431
H	-2.89003484932280	-7.19570049504584	0.84145580674529
O	1.63874912373603	-0.72626384353813	-0.66474180890348
S	1.15664714837446	-1.77983131938414	-1.51039217378947
N	0.44244883337316	-2.93044229828432	-0.70304267695798
S	-0.23652390542778	-2.46531986492544	0.63218673839345
O	-0.87049415382282	-3.55213992812312	1.30451133458432
C	1.17368540718871	-1.97297412907942	1.80893598079258
F	2.37128179151352	-2.37272979907580	1.38336341525611
F	0.97150931594817	-2.54771886153917	3.00153998971343
F	1.21697509179928	-0.65932905610839	2.02397773342446
O	-1.00987780817870	-1.25609509506000	0.51586456710420
C	2.69405658265797	-2.68752972248268	-2.10698433931258
F	2.38562627502879	-3.75601300977644	-2.83535891218303
F	3.46809004633499	-3.09195129936957	-1.10011101444343
F	3.42730631780212	-1.86758283981708	-2.86997955701434
O	0.52949588044443	-1.38629177068244	-2.73716566476996

N	0.51860659292717	0.09164237477453	-5.88749032246723
N	0.52246601793384	-1.09652518250872	-5.63051194662275
N	0.54025386143955	-2.23155255992782	-5.45777185234787
O	-1.96686999665329	-0.44497701037391	-4.60607445994655
N	-2.16216361883034	0.77649503299621	-4.82954176698494
O	-3.30276028613859	1.22880397182332	-4.97789588786192
C	-1.06678238579376	1.66657871489865	-4.78795066636462
H	-1.24118673008607	2.47875694332225	-5.51023001206466
C	0.30285953274504	1.04758580138441	-4.71937515854301
H	0.33871051608512	0.41687594160465	-3.80898200708119
C	1.38480838051048	2.10504615590221	-4.65058083578287
H	2.35777257363498	1.15350225705422	-2.98515733077947
C	2.38465204289058	1.98895501801917	-3.67247160976402
C	3.38191632978002	2.94273781585252	-3.58873146314023
H	4.16177113889399	2.83151634537293	-2.84985970443096
C	3.39761917233478	4.02097036580916	-4.45794051906639
H	4.17489929396514	4.76706148220604	-4.38114363459339
C	2.42851782299251	4.13316433637636	-5.44049940123414
H	2.45153590409530	4.96531443906635	-6.12815501149905
C	1.43672162718996	3.17764809329135	-5.55598413214878
H	0.70478843707765	3.25225540836846	-6.34638530732211
H	-1.29646380220165	-2.39267576783755	-3.46858821852152
H	-2.94523899618028	-0.84028077931815	-3.19006378728544
H	-1.12813863753960	2.28329195826374	-3.56614861530893

Iig$_2$/Tf$_2$NH product (favored enantiomer)

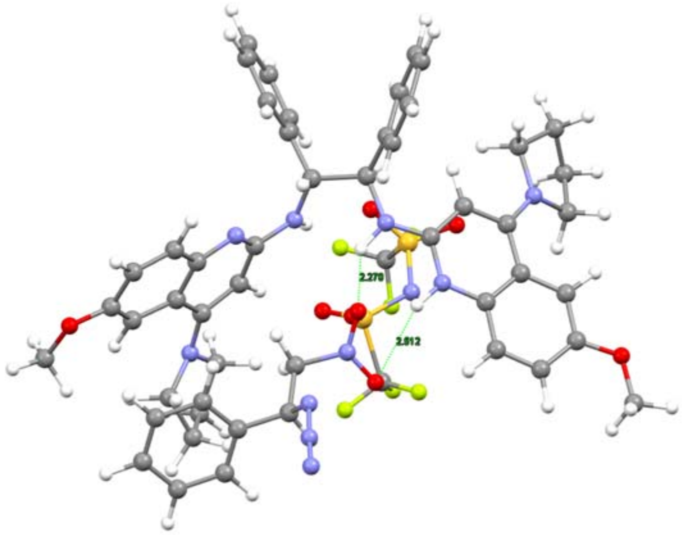

E -4615.592262

G -4614.677638

C	0.26104745525973	-8.66897827475749	-3.70493981753101
O	-0.70654733290696	-8.15847541394601	-2.81098367034990
C	-0.83934630487785	-6.81492025829692	-2.70433317427182
C	-1.81519807224238	-6.36519241758555	-1.81227555689495
C	-2.05740711131086	-4.99272204270877	-1.60744181700929
C	-3.05922169404217	-4.44621648601052	-0.68975980108746
C	-3.35789772213388	-3.07913529188729	-0.79048062749419
C	-2.56650690368130	-2.21002090890662	-1.55637528089215
N	-2.69934302308148	-0.86663755770596	-1.52675832607547
C	-3.60587686060043	-0.18557524200972	-0.61380210114118
C	-3.16991950699222	1.30137025356692	-0.54829348632031
C	-4.01532960198453	2.06841619634083	0.45323959803723
C	-3.88403480121986	1.81622314445857	1.82842873564880
C	-4.67905336848593	2.50039573005397	2.75219467696485
C	-5.61285146985278	3.44657843444640	2.31122252619915
C	-5.74289027467792	3.70739193444485	0.94323103402403
C	-4.94600859018630	3.02153329358877	0.01993064570821
N	-1.75135243846417	1.36536912043331	-0.27418665210411
C	-0.94701449876799	2.37068195772139	-0.76718113678259
C	0.41751095173435	2.38666106418129	-0.37176142936013

C	1.29798860585264	3.32034066128405	-0.91996306891298
N	2.64535680266871	3.23908004356709	-0.65879029338035
C	3.14515461629052	2.19397248768525	0.24249695863131
C	4.66075078225009	2.28191722152479	0.10678589902694
C	4.81784634197673	2.64496919351600	-1.36964982663470
C	3.71457980987487	3.68463090232768	-1.56871121456016
C	0.71883217897270	4.35383933478419	-1.76106519381586
C	1.41870227980081	5.51080472926710	-2.19348537580021
C	0.82276454686236	6.42883490502962	-3.04961690255014
C	-0.50640895579857	6.22330148878927	-3.48991284978839
C	-1.22535840336814	5.14066140537920	-3.02666641132576
C	-0.65966380887566	4.19330636703081	-2.12746370783292
N	-1.46938408414384	3.20987163902703	-1.65047717337898
O	1.43912429249263	7.54360305094837	-3.53151983336482
C	2.73297686280078	7.83702004570242	-3.05545749257700
C	-5.05775932996867	-0.30763010180284	-1.03390536635302
C	-5.42700892483663	-0.14683824170573	-2.37736661100035
C	-6.77234975956390	-0.20490850132697	-2.75198977517631
C	-7.76286558253978	-0.42302208536957	-1.78661831420280
C	-7.40055066939339	-0.58644857940286	-0.44516397871600
C	-6.05351635391603	-0.53087065110996	-0.07246953866611
N	-1.58206978319213	-2.72274011461213	-2.32826714844453
C	-1.31472602062161	-4.07028807573744	-2.39688082980531
C	-0.31931999260689	-4.52406109218374	-3.27672887501117
C	-0.06773134485366	-5.88122446287425	-3.42656208346067
N	-3.69084752679538	-5.19474903472712	0.24395591247368
C	-4.72121877168769	-4.58831853599554	1.10166841013919
C	-5.07404394642271	-5.69244845640442	2.09111567510421
C	-3.72311915095171	-6.37867133498100	2.28836773624907
C	-3.16602143548560	-6.42542533216502	0.86603094654032
H	0.19209775743679	-9.76623256062289	-3.63553008000348
H	1.28473484470975	-8.35361024109124	-3.42166813040878
H	0.05560280970988	-8.35968031496067	-4.74896035306993

H	-2.39826255117049	-7.12995547692446	-1.30197934490117
H	-4.12043820004801	-2.63210914428998	-0.15920640542734
H	-3.48644377641878	-0.61290636313912	0.40093111491363
H	-3.31303846815405	1.75109405082732	-1.54517914028903
H	-3.15313397956707	1.07704109504251	2.18225617998129
H	-4.56879383653573	2.29460811274424	3.82373463229350
H	-6.23581477700877	3.98415442764837	3.03612857950414
H	-6.46694493640524	4.45198723329689	0.59132004194647
H	-5.04480609590079	3.22624159299462	-1.05322370663475
H	-1.39166334665849	0.80460336902685	0.49724566943252
H	0.76925615800813	1.59322258349732	0.28923126687830
H	2.77700072055466	2.35491584788181	1.27280463859366
H	2.78681395412195	1.19440758225610	-0.07747583390657
H	5.06428829336033	3.09048009870062	0.74593807966167
H	5.15802994172096	1.33762844647248	0.38788501549646
H	5.81586688900225	3.03634691453828	-1.63459564355091
H	4.61766740139075	1.75484339267118	-1.99727685219127
H	4.09441234591262	4.68847958710102	-1.28685635994524
H	3.35249714012382	3.73665248253972	-2.60986408932978
H	2.43001035755260	5.68962691797154	-1.83472067095536
H	-0.95055231149783	6.95421072188041	-4.17496178292956
H	-2.26995597693803	4.98921632965027	-3.32042331877053
H	3.45601664275992	7.03264516477664	-3.30060004563542
H	3.04454928622008	8.76727837179681	-3.55791360632464
H	2.73361219171382	7.99982951071510	-1.95858973215062
H	-4.64834522933253	0.01757109697906	-3.13252055234475
H	-7.05050697067070	-0.07916040181332	-3.80526363315505
H	-8.81799808849380	-0.46821292820873	-2.08150967516153
H	-8.17031343718354	-0.75910781241657	0.31639388146080
H	-5.76706499051713	-0.65515120961987	0.97968137776694
H	0.25454451430227	-3.78745292465599	-3.84934304899350
H	0.71441966629306	-6.20327776280513	-4.11899318131750
H	-5.57518981300579	-4.24081290129109	0.49222822954714

H	-4.28768831513958	-3.71705824373414	1.63176537953315
H	-5.50501984770681	-5.28797563628678	3.02269923469196
H	-5.80570383904426	-6.39364692729619	1.64626824963052
H	-3.06930534131903	-5.75146198467832	2.92011482214042
H	-3.79494933701143	-7.38480178845262	2.73632040552050
H	-2.06451709176992	-6.42788143990823	0.83564836608678
H	-3.54327636545194	-7.32328845146285	0.33918259251948
O	1.26905433800587	-0.51609794000956	1.03098378686263
S	0.82570562921071	-1.40108053817330	-0.06132237396486
N	-0.05512734673556	-2.65626544337279	0.42330282602582
S	-0.97969667458262	-2.48188316082319	1.73496584690539
O	-1.70141314733161	-3.73724190832332	1.95178226715818
C	0.22660796218420	-2.39815491653413	3.20122269632524
F	1.42699424823469	-2.84566405473409	2.84383705477988
F	-0.24808717197965	-3.17540149533123	4.17369951077529
F	0.33255152209902	-1.16001117090206	3.65883459706379
O	-1.70977379311232	-1.20118254470389	1.78731164555464
C	2.34927848121692	-2.30298061561654	-0.63188198624003
F	2.04916690858321	-3.16679269075265	-1.59586939784970
F	2.92419046186146	-2.94484088678792	0.37596848633079
F	3.20501610408893	-1.39633019669591	-1.11578095966100
O	0.32845866030272	-0.77288752832958	-1.29907897684255
N	1.20278309525559	0.99636159826273	-6.30662599185722
N	1.77168027642135	0.98710064410813	-7.39141262357793
N	2.18815484715830	0.97187128433681	-8.45361973979530
O	1.05957694336441	-1.25297931538225	-4.06199008382048
N	0.38548800316468	-0.24436888354470	-3.94805081142860
O	-0.83046625827906	-0.23647765265966	-3.82495911936135
C	1.09972332953699	1.07278562661844	-3.92100737247544
H	0.32325569751257	1.85082839342148	-3.93938899509566
C	2.05478590713730	1.15696432793614	-5.10192750611551
H	2.75472090286277	0.30347731234599	-5.04065555872744
C	2.84096947208465	2.45047887105194	-5.12518430944712

H	4.75556725472096	1.45620910316118	-4.93453367914388
C	4.24065890872039	2.41902955444655	-5.04003766207424
C	4.97752694595248	3.60719960650830	-5.09145918133243
H	6.07126354682597	3.57325590857434	-5.02430143906762
C	4.31889109789055	4.83261022253639	-5.23705811384739
H	4.89619559210964	5.76352597219690	-5.28435697803833
C	2.92162823176581	4.86919119413379	-5.32972007387280
H	2.40067470778497	5.82762671940680	-5.43682302594036
C	2.18469646080940	3.68341274613588	-5.27423639350333
H	1.09116755734699	3.71669189580557	-5.35285905868223
H	-0.97043546925829	-2.07251491533598	-2.82941817471391
H	-1.87731834564626	-0.32680551029166	-1.81296296249454
H	1.63849824946236	1.09088511608026	-2.95836111568620

Cartesian coordinates of optimized geometries of ligand-aryl triflamide pairs

Coordinates of optimized geometry of ligNMe$_2$·I8

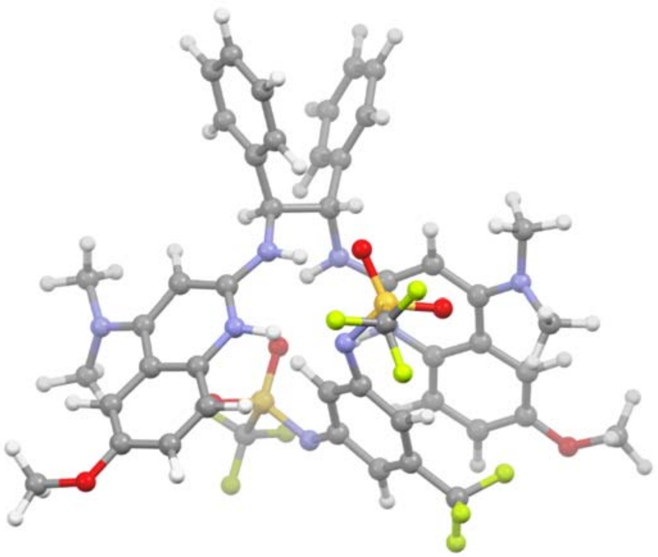

C	-7.9377846265	-0.4452543197	-1.6002199839
O	-6.9958405015	0.2946104639	-2.3789080983
C	-5.6795492493	-0.0030663414	-2.2210034512
C	-5.1961246682	-1.0360227569	-1.4361942859
C	-3.8060322367	-1.2471213977	-1.3108652051
C	-3.2158198569	-2.3001313494	-0.5006232788
C	-1.8552020908	-2.5667121457	-0.6432791588

C	-1.0284367938	-1.7054591613	-1.3725325890
N	0.3217263467	-1.7749254498	-1.3684686047
C	1.0999557749	-2.8342031344	-0.7432654503
C	2.4756849468	-2.2353316256	-0.3254301351
C	3.4202254255	-3.3147790439	0.1682701039
C	4.4271035669	-3.7995556724	-0.6649529769
C	5.2567018771	-4.8334078464	-0.2391325256
C	5.0843933267	-5.3883926760	1.0254570475
C	4.0840235855	-4.9015770238	1.8640170718
C	3.2551921862	-3.8686850380	1.4390354718
N	2.2195218888	-1.2127833451	0.6744061708
C	2.8202939191	-0.0075653607	0.7574022606
C	3.8564914679	0.4211363857	-0.0858111663
C	4.2744540524	1.7478973451	-0.0763608700
N	5.1655283498	2.2133601066	-1.0030519302
C	4.9339113346	3.4974404049	-1.6834041475
C	5.8737705772	1.2572095254	-1.8443759061
C	3.7952571857	2.6198822100	0.9814408080
C	4.3452261401	3.8889092150	1.2591995629
C	3.8206192028	4.6694188462	2.2752465438
C	2.7459440412	4.1947313868	3.0525845060
C	2.2429589955	2.9352216634	2.8390623472
C	2.7781895353	2.1302769267	1.8211021577
N	2.3046212976	0.8496127988	1.6640602298
O	4.2745035124	5.9059701113	2.6117095543
C	5.3171620382	6.4658219244	1.8121544036
C	1.2684791650	-4.0476871390	-1.6362484887
C	1.1372213567	-5.3290087541	-1.1028240767
C	1.3618864569	-6.4492829176	-1.8978524651
C	1.7179232078	-6.2950681824	-3.2346126906
C	1.8391339440	-5.0167119313	-3.7750735094
C	1.6136399378	-3.8978222960	-2.9802363832
N	-1.5825895553	-0.6839120927	-2.0610506453

C	-2.9370888332	-0.4459854130	-2.0751711800
C	-3.4377459918	0.5951998632	-2.8721531923
C	-4.7895457570	0.8236944963	-2.9327214879
N	-3.9806949477	-3.0424502152	0.3466605926
C	-4.9737568152	-2.4148664623	1.2311031850
C	-3.4470357378	-4.2891001170	0.8838261231
H	-7.9448650458	-1.5067315003	-1.8814018853
H	-7.7290372830	-0.3450917805	-0.5266336007
H	-8.9140474319	-0.0096244577	-1.8208169231
H	-5.8813837805	-1.6991476510	-0.9293797813
H	-1.3933098986	-3.3402537563	-0.0487933118
H	0.8213970755	-1.0191289268	-1.8258762340
H	0.6076333288	-3.1486360408	0.1874118886
H	2.9076052351	-1.7710023078	-1.2218376238
H	4.5560054006	-3.3739054738	-1.6577547436
H	6.0377976332	-5.2051573793	-0.8963218942
H	5.7304634006	-6.1952414856	1.3597556402
H	3.9475560821	-5.3276474863	2.8540711776
H	2.4762524805	-3.4966505509	2.1007700128
H	1.4872935791	-1.4174921752	1.3535697359
H	4.2281820596	-0.2639376574	-0.8330508184
H	4.6600007400	3.2998088746	-2.7285404184
H	5.8392811221	4.1171338226	-1.6697004999
H	6.7012003604	1.7792275611	-2.3343234132
H	6.2918339336	0.4480445258	-1.2382838496
H	5.1953487533	4.2330710269	0.6874916712
H	2.3418563770	4.8303036815	3.8344296200
H	1.4349692684	2.5465878296	3.4533440151
H	1.3954877800	0.5874550280	2.1035209881
H	6.2402826181	5.8757973029	1.8863631544
H	5.4972395840	7.4647740212	2.2136565530
H	5.0109841225	6.5470499703	0.7604656493
H	0.8695683957	-5.4494993167	-0.0552864887

H	1.2578401572	-7.4433390040	-1.4721486142
H	1.8948908726	-7.1683438829	-3.8560561663
H	2.1102080365	-4.8911397870	-4.8196895240
H	1.7052380796	-2.9024971720	-3.4094099598
H	-0.9578107373	-0.0196033565	-2.5298361383
H	-2.7445736625	1.2234485527	-3.4243837465
H	-5.1902631606	1.6337027598	-3.5343406133
H	-4.9978743505	-1.3340994806	1.0903845981
H	-5.9758251770	-2.8334895023	1.0706601840
H	-4.2807380673	-4.8667514124	1.2946117417
H	-2.9835832709	-4.8809301819	0.0886220018
O	0.2616403104	-2.2293213677	2.8517798809
S	-0.8388187188	-1.2264320496	2.9182624756
N	-0.3603720710	0.0919337250	2.1917309437
C	-1.2129561746	0.9281940304	1.4474743563
C	-0.6653425354	1.4090673899	0.2522526592
C	-1.3619139653	2.2821239629	-0.5923534289
N	-0.8961952629	2.7425941008	-1.8200039809
S	0.5349231230	2.3393274021	-2.3497440784
O	0.6915663665	0.9039712315	-2.7967489477
C	0.4540220091	3.2807978380	-3.9839621712
F	-0.5444791659	2.8387107973	-4.7632111031
F	0.2944423246	4.5943246669	-3.7837921127
F	1.6144519619	3.0902727119	-4.6381178247
O	1.7357908885	2.8693206175	-1.6604367574
C	-2.6555993781	2.6839480349	-0.2049582516
C	-3.1938687089	2.1973872360	0.9783136755
C	-4.5997823749	2.5548161590	1.3542486442
F	-4.7210076220	2.8648960016	2.6704953855
F	-5.0898908410	3.6040703410	0.6573199805
F	-5.4617213322	1.5145274638	1.1360436081
C	-2.5007884458	1.3244138120	1.8211328006
C	-0.8479618700	-0.7996499052	4.7684144685

F	-1.7373840429	0.1796055154	5.0124482099
F	-1.1824130382	-1.8685607878	5.5100240993
F	0.3645509550	-0.3723823635	5.1636093650
O	-2.2183709880	-1.6925559568	2.6659362094
H	0.3229022720	1.0586680235	-0.0249183599
H	-3.2166657378	3.3533378815	-0.8470747902
H	-2.9520365316	0.9641547576	2.7385045347
H	5.2281653124	0.8285867577	-2.6284953579
H	4.1045633458	4.0380302238	-1.2317965293
H	-4.6766208716	-2.6012723770	2.2692583916
H	-2.7170259133	-4.1115744544	1.6878699345

Coordinates of optimized geometry of lig$_2$·I3

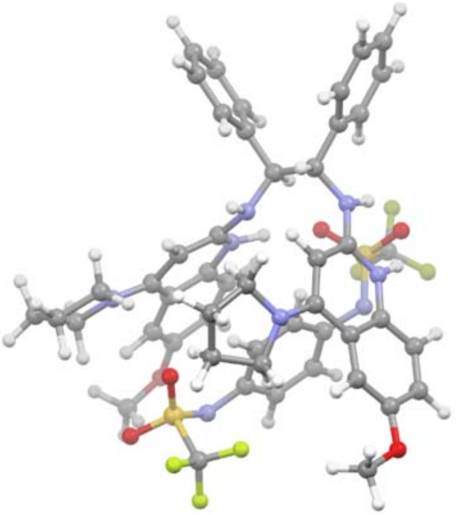

C	-3.4103222572	-6.2273388128	0.1363566924
O	-2.5749999966	-6.2337745248	-1.0228288806
C	-1.5310232539	-5.3652624994	-1.0527345074
C	-1.1615916761	-4.5492824425	0.0035203892
C	-0.0790722096	-3.6512405282	-0.1215829829
C	0.3472107373	-2.7070964599	0.9054558325
C	1.5610354447	-2.0315520721	0.7159618659
C	2.2376328420	-2.0717686771	-0.5059381189
N	3.3029403186	-1.3052478192	-0.8346165506
C	3.8923058000	-0.2096465829	-0.0687886137

C	3.1964943581	1.1512889716	-0.3888065213
C	3.9879678995	2.3447218480	0.1088089856
C	4.2408623967	2.5262880904	1.4711411819
C	4.9659428238	3.6275984653	1.9123137257
C	5.4415609944	4.5641838785	0.9964500608
C	5.1851648649	4.3944343808	-0.3603194280
C	4.4617061172	3.2894730534	-0.8009060523
N	1.8402992251	1.1298119284	0.1795997218
C	0.8030322542	1.9147982878	-0.2593344711
C	-0.1616203997	2.3783122963	0.6281139402
C	-1.3164332170	3.0516974091	0.1893548962
N	-2.1719830152	3.5357867773	1.1035662137
C	-3.2959624555	4.4708392951	0.9021254580
C	-3.5436967842	5.0265203603	2.3015318558
C	-3.2728882402	3.8069261389	3.1816239986
C	-2.0347423523	3.1955446101	2.5322773394
C	-1.5159500754	3.1660357236	-1.2513287230
C	-2.7339091735	3.5226865049	-1.8642305953
C	-2.8621672281	3.5302945433	-3.2434610710
C	-1.7567017969	3.2348879852	-4.0577369044
C	-0.5697304647	2.8479827345	-3.4848504423
C	-0.4533510720	2.7655363525	-2.0906401933
N	0.6898991371	2.1818524820	-1.5720559897
O	-4.0200088246	3.7920333777	-3.8991241461
C	-5.2074176959	3.9393149861	-3.1090942546
C	5.3731603463	-0.1505021883	-0.3915757837
C	6.3218099289	-0.3204666348	0.6146714558
C	7.6812485652	-0.2595025779	0.3200326534
C	8.1015811655	-0.0318522295	-0.9864790098
C	7.1577243004	0.1351148240	-1.9971691643
C	5.8001146545	0.0791336504	-1.7024063951
N	1.7810110733	-2.8953799826	-1.4752307182
C	0.6649477301	-3.6844998247	-1.3186629105

C	0.2806372175	-4.5122347934	-2.3839558459
C	-0.8126348849	-5.3318120470	-2.2610787540
N	-0.3827983760	-2.4524106721	2.0179894017
C	0.1194359835	-1.5187642278	3.0443896196
C	-1.0268747053	-1.4102281458	4.0427176504
C	-2.2515470589	-1.5165841595	3.1355510072
C	-1.8513975704	-2.6235804116	2.1636461617
H	-2.8636113422	-6.5648989706	1.0270095008
H	-3.8355944160	-5.2301937558	0.3138821769
H	-4.2176445560	-6.9309437591	-0.0749363596
H	-1.6983148246	-4.6194193196	0.9343213404
H	1.9240178783	-1.3494119396	1.4664644265
H	3.5866983077	-1.3478033232	-1.8107495796
H	3.7848148207	-0.4211880015	1.0019024948
H	3.1209644566	1.2094205724	-1.4786152239
H	3.8792526679	1.8020022438	2.1992278628
H	5.1633525668	3.7552713809	2.9730236226
H	6.0086916103	5.4239498216	1.3414735659
H	5.5491351897	5.1226440410	-1.0795836901
H	4.2658265048	3.1540466992	-1.8621041002
H	1.8408529165	1.0467362082	1.1880108617
H	0.0228434575	2.2248563653	1.6826774391
H	-4.1900597053	3.9279762633	0.5628976816
H	-3.0326417139	5.2394290270	0.1701136738
H	-4.5584152187	5.4199962286	2.4036323519
H	-2.8316897893	5.8301574702	2.5262734139
H	-4.1119345640	3.1050402896	3.1176580704
H	-3.1020756900	4.0557722138	4.2323142094
H	-1.9863164033	2.1068875043	2.6517316867
H	-1.1145739105	3.6426162302	2.9398199407
H	-3.6070674541	3.7052328619	-1.2636459686
H	-1.8710737923	3.2613529177	-5.1370183587
H	0.2687268949	2.5486383987	-4.1064797792

H	1.2550278936	1.6507532693	-2.2450474847
H	-5.1574863307	4.8419539534	-2.4841389025
H	-5.3772895050	3.0543282343	-2.4836240639
H	-6.0248511903	4.0467084146	-3.8247361277
H	5.9971637057	-0.4993320115	1.6370160935
H	8.4117275927	-0.3922354146	1.1131129581
H	9.1618371227	0.0143085820	-1.2180753061
H	7.4787056885	0.3124631445	-3.0195366937
H	5.0759413629	0.2214609016	-2.5031990733
H	2.1901133466	-2.8286868169	-2.4087326973
H	0.8476343417	-4.4805065559	-3.3096791399
H	-1.1308005885	-5.9644350390	-3.0837086909
H	1.0521985119	-1.8925365973	3.4835865645
H	0.3173283069	-0.5333328098	2.5898917407
H	-0.9836653869	-0.4762183999	4.6094358724
H	-0.9964938254	-2.2468280169	4.7511744043
H	-2.4145570310	-0.5826825384	2.5857476674
H	-3.1727276351	-1.7642725179	3.6696195863
H	-2.3479091810	-2.5316413644	1.1949410153
H	-2.0835512300	-3.6052665061	2.5991231706
N	-4.1826848270	0.6158020620	-1.7490014158
S	-4.5774123104	0.7577625970	-0.2349078425
O	-5.6904834863	1.7244813427	-0.0919139497
C	-5.4184805259	-0.8969112400	0.2121970199
F	-5.7665529109	-0.9466091398	1.5166449847
F	-4.5729682350	-1.9310776497	-0.0203364584
F	-6.5254497358	-1.1027030801	-0.5187861160
O	-3.5092278469	0.8273247713	0.8037016364
C	-2.9764253185	0.0772194410	-2.2112712939
C	-1.8134804573	-0.1647364960	-1.4571350369
C	-0.6539412437	-0.6208448290	-2.0654301746
C	-0.5857134066	-0.8653647086	-3.4398367394
N	0.5677808714	-1.4516164556	-4.0219403581

S	1.9232405162	-0.7024607746	-4.1312678915
O	2.0712586355	0.6580874116	-3.5126993391
C	2.1914544021	-0.2910966713	-5.9674490841
F	1.2321316813	0.5576079249	-6.3821810214
F	3.3862388032	0.2916148455	-6.1780002631
F	2.1258041120	-1.4013262033	-6.7177812755
O	3.0767239656	-1.6248692307	-3.8758589299
C	-1.7440474386	-0.6477775589	-4.1949082304
C	-2.9079636193	-0.1854635462	-3.5943889398
H	-1.8127979798	0.0260449998	-0.3907842973
H	0.2277124350	-0.7819162823	-1.4541368778
H	-1.7248995346	-0.8440255593	-5.2640671536
H	-3.7950209910	-0.0060068660	-4.1965163906

Coordinates of optimized geometry of lig$_2$·I4

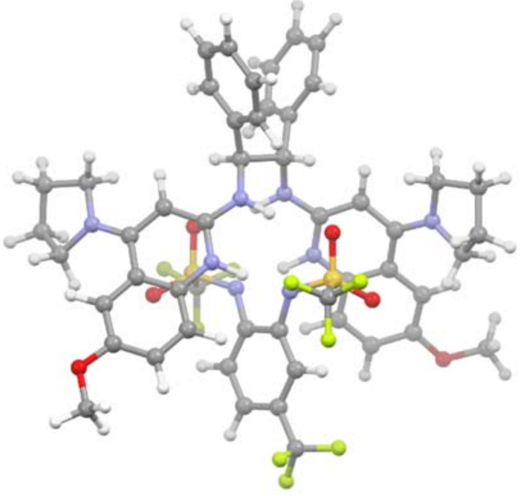

C	-5.2567411215	5.7443976942	-2.5100165522
O	-5.6374265585	4.5051145948	-1.9091557022
C	-4.6757453326	3.5497642217	-1.7692314792
C	-5.0855662428	2.3684099365	-1.1655169140
C	-4.1888558486	1.3101588974	-0.9599741466
C	-4.5131104921	0.0596308391	-0.2812750094
C	-3.6468523946	-1.0243134641	-0.4596268717

C	-2.3557933142	-0.8215045231	-0.9541652061
N	-1.3865482388	-1.7634462622	-0.9413870361
C	-1.4667145001	-3.0149288892	-0.2042210864
C	-0.0292668716	-3.3527321578	0.2919395146
C	0.0143301377	-4.7012224119	0.9833317162
C	0.4498453325	-5.8282778776	0.2867920436
C	0.4330572122	-7.0816375185	0.8917376581
C	-0.0181638903	-7.2165644739	2.2017198726
C	-0.4450504751	-6.0921664176	2.9049038708
C	-0.4273208543	-4.8397871722	2.2996773621
N	0.4047562915	-2.2576065135	1.1467556920
C	1.6726144902	-1.7878917107	1.1889181617
C	2.7688693835	-2.4595978696	0.6438418357
C	3.9953003262	-1.8015924907	0.4990652567
N	4.9382434854	-2.2997136888	-0.3333233901
C	5.9664107572	-1.4804571354	-1.0238842372
C	6.0485406144	-2.1082859218	-2.4128839115
C	5.8004145892	-3.5883749257	-2.1232538423
C	4.6639033703	-3.5275008674	-1.1086226894
C	4.2084172086	-0.5838129901	1.2683764550
C	5.4712790521	-0.0027116889	1.5104889577
C	5.5722649776	1.1952244467	2.1977817137
C	4.4150957901	1.8453551769	2.6650113504
C	3.1843512991	1.2598866835	2.4986019962
C	3.0726224696	0.0301573818	1.8304898581
N	1.8381281068	-0.5625134943	1.7270347186
O	6.7443955578	1.8292943137	2.4757935949
C	7.9480381808	1.2314799340	1.9963413946
C	-2.0484248194	-4.1496940608	-1.0232911479
C	-2.9418273127	-5.0472179570	-0.4397707501
C	-3.4301671862	-6.1290511079	-1.1670791925
C	-3.0284349829	-6.3194272685	-2.4861649558
C	-2.1432495057	-5.4196021381	-3.0762669922

C	-1.6568090039	-4.3381314754	-2.3492511066
N	-1.9962056159	0.3945044117	-1.4094390498
C	-2.8799118798	1.4450918323	-1.4760043484
C	-2.4664555845	2.6460328010	-2.0586525445
C	-3.3458949475	3.7025755145	-2.1917818705
N	-5.6090449059	-0.0746720976	0.4975343613
C	-6.2927548755	1.0332878019	1.2083288593
C	-6.7176778200	0.4007404792	2.5315785760
C	-7.0285180701	-1.0405750306	2.1282705331
C	-5.8857123085	-1.3598047097	1.1706198490
H	-4.9118605581	5.6016624290	-3.5425520012
H	-6.1581775876	6.3602181344	-2.5152115383
H	-4.4780367013	6.2513655164	-1.9253653098
H	-6.1255582055	2.2889562951	-0.8805800668
H	-3.8936298364	-2.0008171538	-0.0721195784
H	-0.4661928439	-1.4905798283	-1.2820463072
H	-2.0877318906	-2.8681784609	0.6920253088
H	0.6151140069	-3.3914103218	-0.5989789626
H	0.7923797166	-5.7234121318	-0.7405283809
H	0.7734247057	-7.9530771462	0.3394468982
H	-0.0317739170	-8.1937953646	2.6761238726
H	-0.7913508016	-6.1906064035	3.9300792806
H	-0.7533157527	-3.9642533645	2.8559653213
H	-0.3268471091	-1.6615490351	1.5314253453
H	2.5998114454	-3.4256855307	0.1934545325
H	6.9373671433	-1.5665075396	-0.5159095895
F	0.1052241346	0.6072117334	-3.9825657717
H	7.0111023827	-1.9100646390	-2.8922598436
H	5.2495271583	-1.7082492404	-3.0481757111
H	6.6916940716	-4.0470950387	-1.6782041738
H	5.5207808453	-4.1649558637	-3.0088479549
H	4.6178585076	-4.4016554538	-0.4480573596
H	3.6971622461	-3.4278117359	-1.6261367806

H	6.3629310649	-0.5120211392	1.1782585396
H	4.5150306196	2.7969927089	3.1776391529
H	2.2879931118	1.7365167176	2.8863005653
H	0.9874733167	0.0296979647	1.8129516411
H	7.9397199210	1.1385782181	0.9016681231
H	8.1150812517	0.2457906651	2.4512424649
H	8.7546336573	1.9043833867	2.2935994610
H	-3.2472288097	-4.9034969598	0.5946175464
H	-4.1252967720	-6.8230968278	-0.7028966984
H	-3.4082047278	-7.1630399671	-3.0556638745
H	-1.8321429862	-5.5598491145	-4.1078443819
H	-0.9715132486	-3.6338809321	-2.8151923369
H	-0.9773552681	0.5862676807	-1.4717611133
H	-1.4443101075	2.7378216789	-2.4165484678
H	-2.9980114341	4.6235604999	-2.6444578086
H	-5.6125081485	1.8781100421	1.3391121525
H	-7.1784725862	1.3645674740	0.6493638551
H	-7.5685303943	0.9241673320	2.9760023021
H	-5.8823138280	0.4237320843	3.2412163174
H	-7.9931311994	-1.0942426347	1.6094892449
H	-7.0514852974	-1.7330034090	2.9737537137
H	-6.1334464128	-2.1359381056	0.4364615368
H	-4.9943805337	-1.6821846494	1.7311170572
O	1.4812681416	-1.2578705726	-2.1273330183
S	1.8185634266	0.1814535900	-1.9574176613
N	0.7522327445	0.7887869664	-0.9669098533
C	0.7804491574	2.0482851096	-0.3567630141
C	1.4449463424	3.1738191273	-0.8419682882
C	1.2655667072	4.4209529169	-0.2428381565
C	1.9418562636	5.6086258794	-0.8442128213
F	3.2037812400	5.3335223187	-1.2613805423
F	1.2801785237	6.0772573693	-1.9458541094
F	2.0283020803	6.6532759295	0.0144951856

C	0.4034204517	4.5690560705	0.8383982998
C	-0.2284404946	3.4493805698	1.3642240996
C	-0.0282622541	2.1768308408	0.8194160779
N	-0.4851505220	0.9732455882	1.3619923236
S	-1.7307561777	0.7680758538	2.3105827914
O	-1.9827640909	-0.6965953201	2.3855275492
C	-1.1537769732	1.0804919379	4.1048086036
F	-0.0508017781	0.3493016627	4.3637562951
F	-2.1094154311	0.7249383077	4.9792617402
F	-0.8501149921	2.3737717256	4.3211000095
O	-2.8853811191	1.6758455811	2.1508014194
C	1.4121116032	0.8110824127	-3.7160379324
F	1.6593081602	2.1266750950	-3.8545721790
H	5.6608870615	-0.4320941266	-1.0471361286
F	2.1358057784	0.1523127269	-4.6364696309
O	3.2361274263	0.5581927805	-1.7749025359
H	2.0983118058	3.0828196470	-1.7020503534
H	0.2379003035	5.5426903947	1.2873122597
H	-0.8791865352	3.5659046678	2.2230283499

Coordinates of optimized geometry of lig$_2$·I8

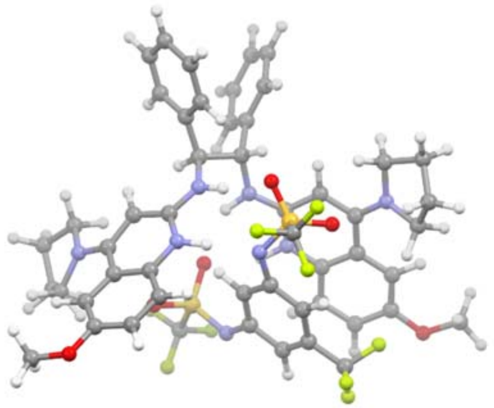

C	-7.1876727292	4.6155838568	-1.5454903604
O	-5.9868550778	4.5926820960	-2.3168071504

C	-5.1365342612	3.5471217149	-2.1269731800
C	-5.3805469708	2.4825702437	-1.2754419330
C	-4.4375813808	1.4414536537	-1.1394408893
C	-4.5843593926	0.3063897506	-0.2373963026
C	-3.7689103332	-0.8116511922	-0.4577621660
C	-2.6394512749	-0.7185381489	-1.2749568431
N	-1.6778330033	-1.6643653632	-1.3589274484
C	-1.6292087119	-2.9014195211	-0.6015526384
C	-0.1493629613	-3.1364167484	-0.1752027821
C	0.0366376452	-4.5040715273	0.4531200309
C	-0.3679369732	-4.7410595551	1.7675538018
C	-0.2630667455	-6.0154723630	2.3148486086
C	0.2505117764	-7.0637817718	1.5543162690
C	0.6664710233	-6.8295165263	0.2467258665
C	0.5604305209	-5.5540457234	-0.3002267357
N	0.2326185561	-2.0499524975	0.7124827714
C	1.4886967890	-1.5678781181	0.8494602734
C	2.5793172530	-2.0083462227	0.0995613023
C	3.7963220221	-1.3146669681	0.1116493071
N	4.7492606086	-1.6393855339	-0.7898719248
C	5.7875646059	-0.7241597922	-1.3235060915
C	6.0018103269	-1.2142711592	-2.7547344677
C	5.7677946995	-2.7195215440	-2.6287155906
C	4.5500184238	-2.7742157952	-1.7143746145
C	3.9846422220	-0.2821657187	1.1253490690
C	5.2168917265	0.3428300399	1.4176224205
C	5.3022858933	1.3195144542	2.3954401251
C	4.1647301983	1.6850187489	3.1360178151
C	2.9672139699	1.0568405634	2.9034428572
C	2.8683663927	0.0676070804	1.9131830778
N	1.6566352257	-0.5546869305	1.7268885920
O	6.4435071223	1.9847039911	2.7191300671
C	7.6067894569	1.7226046349	1.9339139930

C	-2.1784588762	-4.0854717168	-1.3741506597
C	-1.8121473346	-4.2888291873	-2.7059367004
C	-2.2672936997	-5.4087295108	-3.3934805133
C	-3.0939640923	-6.3332796874	-2.7587505283
C	-3.4685926544	-6.1296386796	-1.4338667318
C	-3.0119008597	-5.0091309595	-0.7453142977
N	-2.4126954821	0.4152181040	-1.9740495725
C	-3.2966902059	1.4675019979	-1.9657925877
C	-3.0442623013	2.5670084529	-2.8010496650
C	-3.9439404022	3.6018380127	-2.8707713594
N	-5.4744013332	0.2986509095	0.7785669544
C	-5.5623540673	-0.8717577232	1.6748865341
C	-6.4828859127	-0.4120648155	2.7985959691
C	-6.1172363697	1.0660140073	2.9252230588
C	-5.9936561980	1.5065446497	1.4686721686
H	-7.6992334041	5.5408907451	-1.8168621056
H	-7.8358593812	3.7622566165	-1.7858237036
H	-6.9697066303	4.6247118366	-0.4688688046
H	-6.3119193892	2.4322863873	-0.7338738250
H	-3.9203970166	-1.7195634435	0.1044134548
H	-0.9125556358	-1.4990232763	-2.0110517915
H	-2.2088035726	-2.7854490499	0.3240884529
H	0.4500099700	-3.0881266519	-1.0958125696
H	-0.7633352305	-3.9251399343	2.3684196110
H	-0.5821231808	-6.1914086405	3.3384444607
H	0.3318855943	-8.0588639702	1.9823613381
H	1.0752246667	-7.6404919766	-0.3494785116
H	0.8754300447	-5.3726453287	-1.3254961742
H	-0.4991037149	-1.5748903820	1.2306408013
H	2.4168154708	-2.8055145949	-0.6077192588
H	6.7240746481	-0.8261759990	-0.7576112503
H	5.4470421847	0.3139715764	-1.2722059184
H	6.9983559804	-0.9558137867	-3.1231965688

H	5.2503526938	-0.7715952615	-3.4162775253
H	6.6292806200	-3.2056288832	-2.1547341188
H	5.5757073691	-3.2100819649	-3.5863728588
H	4.4623853904	-3.7115506547	-1.1518948507
H	3.6327518984	-2.6206614859	-2.3012710667
H	6.1077364128	0.0445759243	0.8906386045
H	4.2504483010	2.4626098938	3.8887063274
H	2.0810000232	1.3298026204	3.4697198497
H	0.8354744100	-0.2028300612	2.2274362666
H	7.4256369454	1.9367946843	0.8719453006
H	7.9460665070	0.6846944558	2.0520205314
H	8.3789340934	2.3958468098	2.3107696640
H	-1.1669334326	-3.5729266108	-3.2104090689
H	-1.9754614997	-5.5596242983	-4.4290657983
H	-3.4489952878	-7.2070060276	-3.2979572546
H	-4.1178103593	-6.8433567277	-0.9344812875
H	-3.2964720243	-4.8565408668	0.2935549339
H	-1.4494275650	0.5650747775	-2.3444301419
H	-2.1349845048	2.5814492213	-3.3959600509
H	-3.7616196794	4.4571640843	-3.5139359413
H	-5.9373561030	-1.7482665950	1.1329718824
H	-4.5620010045	-1.1061361478	2.0728246040
H	-6.3162502476	-0.9818359724	3.7164271911
H	-7.5337262597	-0.5253206157	2.5057027758
H	-5.1491188803	1.1757049270	3.4263295798
H	-6.8627421988	1.6563653456	3.4649723908
H	-5.3049342234	2.3438249167	1.3370797069
H	-6.9828298339	1.7809219448	1.0759970320
O	-1.0582523115	0.0033051118	2.5360452064
S	-1.4373833889	1.4645606770	2.4870166065
N	-0.2650690150	2.4721579859	2.1758782954
C	0.4060005700	2.4502338117	0.9566299704
C	0.0792430885	1.6239492963	-0.1247547234

C	0.8438050222	1.5919392823	-1.2986550444
N	0.3637421791	0.7200299870	-2.2919479955
S	1.2898158834	-0.1761508713	-3.2040316521
O	0.5863881668	-1.4612039512	-3.4755577101
C	1.2376214355	0.6474442337	-4.9150169595
F	-0.0311688208	0.7547418749	-5.3491551081
F	1.7642149805	1.8838089409	-4.8509384483
F	1.9353441139	-0.0609752254	-5.8190639753
O	2.7333793134	-0.2290148208	-2.8895171035
C	1.9690411861	2.4142976746	-1.4058762024
C	2.2906689921	3.2412922843	-0.3261344011
C	3.5324572959	4.0716661366	-0.4452043090
F	4.6438301299	3.2969136412	-0.6197154961
F	3.4939069439	4.9076437244	-1.5174988020
F	3.7700648889	4.8406662989	0.6406756335
C	1.5359035443	3.2836924103	0.8365991910
C	-1.6463559086	1.9094142134	4.3097343029
F	-2.0180604320	3.1862862416	4.4622002076
F	-2.6084764884	1.1245361753	4.8312427457
F	-0.5171316027	1.7021484479	5.0027516847
O	-2.7728547523	1.7010177635	1.8867116582
H	-0.7690506358	0.9520460795	-0.0546829188
H	2.5844361723	2.4059308909	-2.2983218607
H	1.8138058668	3.9213726069	1.6676168508

Coordinates of optimized geometry of lig_2·J1

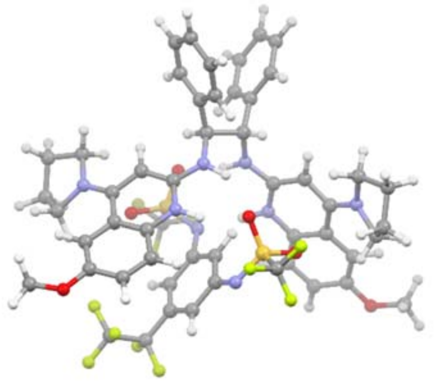

C	-2.8944326574	4.0573053495	2.5676587499
H	-2.1238630649	3.4919364311	3.0873656886
C	-3.5595586820	5.0821688008	3.2321389566
H	-3.3045638950	5.3084710606	4.2636905884
C	-4.5473105170	5.8162276008	2.5791989368
H	-5.0657540008	6.6162982004	3.1000555779
C	-4.8715305558	5.5161601375	1.2593775128
H	-5.6446675058	6.0805904042	0.7456752481
C	-4.2049755539	4.4909623199	0.5940054412
H	-4.4513356311	4.2660496573	-0.4414573548
C	-3.2107035279	3.7588929116	1.2411082759
C	-2.4364281533	2.6912681993	0.4915414948
H	-2.9785769899	2.4443784887	-0.4307077876
N	-2.2404499632	1.4844275186	1.2748074960
H	-1.4626322116	1.4903496476	1.9329290825
C	-2.9866523312	0.3591620579	1.2029497821
N	-2.5474911494	-0.6861122679	1.9369718100
H	-1.5836876538	-0.6228279748	2.3305257868
C	-3.1985365995	-1.8963413873	1.9485743385
C	-2.7449792586	-2.8984729056	2.8208158216
H	-1.8657009997	-2.7095723697	3.4308098788
C	-3.4155881665	-4.0935233327	2.9078331559
H	-3.0760444862	-4.8763931115	3.5788853425
C	-4.5777193312	-4.3041460370	2.1437691058

O	-5.1978303244	-5.4983789069	2.3470533865
C	-6.3493348283	-5.7877992557	1.5545916901
H	-7.1685272115	-5.0872624589	1.7655182118
H	-6.6593963216	-6.7960654237	1.8355342518
H	-6.1109031511	-5.7684538030	0.4823212195
C	-5.0180554969	-3.3331265772	1.2601936012
H	-5.9288399717	-3.4888532234	0.7041607439
C	-4.3056480313	-2.1245041753	1.1075171969
C	-4.6643085281	-1.0665391311	0.1718831379
N	-5.5207999748	-1.2658358665	-0.8532125980
C	-5.7751342420	-2.5708328286	-1.5133393170
H	-4.9353605366	-3.2496230386	-1.3488344136
H	-6.6966172869	-3.0272311486	-1.1248360079
C	-5.9578375335	-2.2025347060	-2.9835372394
H	-4.9779100966	-2.1253006614	-3.4678774003
H	-6.5571630038	-2.9462261177	-3.5158606036
C	-6.6189333582	-0.8270816278	-2.9042702090
H	-6.5541045783	-0.2587579453	-3.8357438327
H	-7.6762065746	-0.9231473667	-2.6289064030
C	-5.8318777140	-0.1607861670	-1.7822829302
H	-4.8943397176	0.2642583288	-2.1748159703
H	-6.3882261504	0.6327664243	-1.2691056074
C	-4.0959241717	0.1990614353	0.3684220251
H	-4.4182807542	1.0415094303	-0.2226561406
C	-1.5623082275	4.6823038842	-1.9200124390
H	-1.7915465732	3.7867566494	-2.4932837368
C	-1.7064833292	5.9350182204	-2.5067665816
H	-2.0525621175	6.0133654991	-3.5337349145
C	-1.4064447054	7.0862285120	-1.7815925998
H	-1.5181135661	8.0641634935	-2.2413406892
C	-0.9554184123	6.9781346891	-0.4692984955
H	-0.7133653379	7.8715073965	0.0994374130
C	-0.8125150207	5.7239359694	0.1174792865

H	-0.4697577653	5.6401543503	1.1465596716
C	-1.1200709430	4.5695877389	-0.6011432861
C	-1.0321979450	3.2107733076	0.0661294073
H	-0.4497795817	3.3082898909	0.9937992222
N	-0.4207871417	2.2030674969	-0.7857107532
H	-1.0281742690	1.5643933257	-1.2894723407
C	0.9107737820	1.9895719872	-0.8889637810
N	1.3024955960	0.9939334494	-1.7130549737
H	0.5809324943	0.4625698596	-2.2100847352
C	2.6234942323	0.6541629258	-1.8826774212
C	2.9498647146	-0.3146372525	-2.8450095193
H	2.1503583656	-0.8153666742	-3.3848472945
C	4.2627376750	-0.6274856310	-3.0909924827
H	4.5282549539	-1.3816329659	-3.8252922095
C	5.2850038271	0.0342501561	-2.3879398596
O	6.5511379197	-0.3314206371	-2.7242220863
C	7.6224121340	0.2202507079	-1.9586554542
H	7.7053855351	1.3055646843	-2.1060707565
H	7.5015467954	-0.0012987039	-0.8894286914
H	8.5308956982	-0.2608549174	-2.3258752893
C	4.9737689625	0.9795705066	-1.4261050141
H	5.7683518625	1.5083359219	-0.9262273141
C	3.6287534468	1.2843615448	-1.1214145937
C	3.2071237832	2.2687721165	-0.1315455876
N	4.0556290169	2.8055973409	0.7719652817
C	5.2741421216	2.1521411877	1.3086699930
H	5.1890741286	1.0647832988	1.2346381845
H	6.1665883413	2.4825884211	0.7584213793
C	5.3487208058	2.6525529741	2.7498490057
H	4.7089772358	2.0371223336	3.3902776495
H	6.3724012832	2.6240506418	3.1333010092
C	4.7766235690	4.0656395308	2.6391749332
H	4.4616223050	4.4824202802	3.5992713355

H	5.5113943739	4.7445346452	2.1890694832
C	3.5934045604	3.8575918776	1.7008987821
H	2.7223418780	3.4948415563	2.2660414163
H	3.3101849525	4.7605120003	1.1463918561
C	1.8683285306	2.6809104529	-0.1472387830
H	1.5306351818	3.4526019441	0.5257066513
S	-1.2802300372	-1.6288702733	-2.4884668825
O	-2.5568018156	-2.1005184941	-1.8998400917
O	-1.1931773927	-0.1242276417	-2.5890787226
N	0.0573573437	-2.3777180936	-2.1188596748
S	0.9794981496	0.6584266360	3.1672537859
O	2.3878673183	0.9387232940	2.8168375068
O	0.0642403820	1.8131260623	3.3877006482
N	0.2165680710	-0.4266495370	2.3123422137
C	-1.3535672710	-2.1659148454	-4.2962018837
F	-0.2760280428	-1.7553171985	-4.9804982537
F	-1.4550320472	-3.4959290427	-4.4082467738
F	-2.4443813984	-1.6107265748	-4.8583202971
C	1.0961777800	-0.0537572238	4.9248747156
F	-0.1247157956	-0.3747570844	5.3873765204
F	1.6492100462	0.8339797372	5.7691916079
F	1.8519418853	-1.1669726902	4.9319138032
C	0.1805377005	-1.3988365309	0.1530165598
H	-0.7030899031	-0.7899237545	-0.0062242781
C	0.6407021059	-2.2496085789	-0.8619523800
C	1.8067773981	-2.9989338396	-0.6156883381
H	2.1773203582	-3.6702694467	-1.3829844722
C	2.4613471222	-2.8669602957	0.6047036207
C	3.7125322815	-3.6616149449	0.8469265679
F	3.9000206891	-3.9073956465	2.1806627065
F	3.6715153200	-4.8754457288	0.2141924375
C	5.0160608963	-2.9721489880	0.3560419244
F	4.9903720420	-2.8133370448	-0.9768347643

F	6.0999639342	-3.7035528195	0.6691180924
F	5.1514406088	-1.7609015920	0.9279249419
C	2.0101713802	-2.0086154881	1.6092092271
H	2.5381720620	-1.9291245149	2.5513789120
C	0.8455524556	-1.2699261547	1.3765784293

Coordinates of optimized geometry of lig$_2$·K5

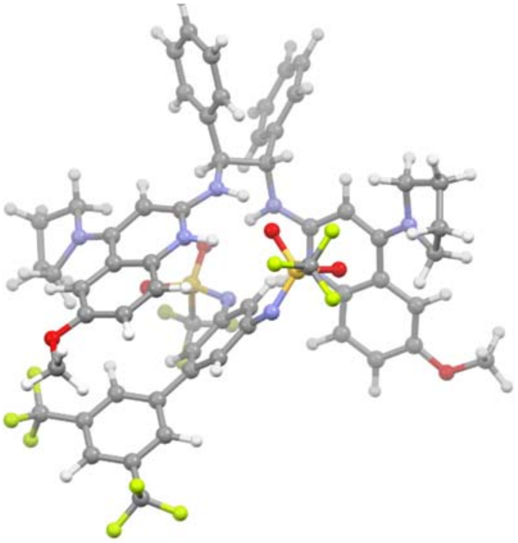

C	-6.5472907176	0.9348343114	2.5926644502
O	-5.7769420736	1.8727879262	1.8386200796
C	-4.4237195083	1.7205759225	1.8352202928
C	-3.7244776806	2.6006634352	1.0205174496
C	-2.3291450578	2.5234340378	0.8901098757
C	-1.5171507018	3.3930867707	0.0421656467
C	-0.1271332810	3.3499401546	0.2027690040
C	0.4848570139	2.3290692774	0.9326423527
N	1.8157567595	2.0902327483	0.9179772109
C	2.7764368309	2.8309798401	0.1152341008
C	3.9077462399	1.8493218575	-0.3081097689
C	5.0343830946	2.5785009957	-1.0154331309
C	4.8842987707	2.9874463196	-2.3418521993
C	5.8926444626	3.7109189694	-2.9695363795

C	7.0601924830	4.0293767417	-2.2793919383
C	7.2176645245	3.6155487046	-0.9598072637
C	6.2077017103	2.8923391043	-0.3312802784
N	3.3070076449	0.8114046430	-1.1251799509
C	3.6578136135	-0.4949189441	-1.1240095820
C	4.6445743999	-1.0451457751	-0.3025212549
C	4.7667400628	-2.4347750153	-0.1665114530
N	5.4917362174	-2.9474671096	0.8506246935
C	5.2961665149	-4.2928347961	1.4462577929
C	5.5709419008	-4.0755930014	2.9325272664
C	6.6375043449	-2.9814902172	2.9213515012
C	6.1221131888	-2.0455069440	1.8350704532
C	4.0975523571	-3.2739599150	-1.1521194030
C	4.3775051303	-4.6413190831	-1.3640760027
C	3.6543749034	-5.3728830563	-2.2912434863
C	2.6322170092	-4.7596304099	-3.0382824055
C	2.3892449895	-3.4162442262	-2.8934991256
C	3.1371788527	-2.6578928423	-1.9787411525
N	2.9151725759	-1.3031065037	-1.9113055647
O	3.8529988160	-6.6930660453	-2.5555789525
C	4.8548700124	-7.3748147831	-1.8016702543
C	3.3312547527	4.0458344006	0.8339828531
C	3.5178662931	5.2425692180	0.1429533799
C	4.0899655214	6.3410782398	0.7779375887
C	4.4782311296	6.2510777457	2.1116616840
C	4.2842557049	5.0608441168	2.8091949912
C	3.7117997251	3.9636783786	2.1739284389
N	-0.2780282174	1.4726559623	1.6435536502
C	-1.6513785568	1.5652854715	1.6816297411
C	-2.3599844748	0.6952047103	2.5133298116
C	-3.7372399828	0.7549404372	2.5858745510
N	-2.0528165411	4.2343311118	-0.8673127596
C	-1.1878906311	5.1449236668	-1.6436063832

C	-2.1409380310	5.7998448620	-2.6362599211
C	-3.1116608199	4.6603789793	-2.9436568653
C	-3.3472656764	4.0435229035	-1.5664565082
H	-7.5921586058	1.1923847186	2.4103811241
H	-6.3653352251	-0.0953644152	2.2599897466
H	-6.3384401316	1.0178646909	3.6676005000
H	-4.3022209209	3.3510117300	0.5022944196
H	0.5046611929	4.0160524671	-0.3626503256
H	2.1344392425	1.2496303030	1.3901548146
H	2.2940781414	3.1652547252	-0.8145571926
H	4.3019964053	1.4015883189	0.6143036314
H	3.9768836861	2.7428805153	-2.8896414021
H	5.7660813357	4.0267494278	-4.0013305751
H	7.8469643818	4.5941820022	-2.7714966864
H	8.1280949638	3.8557279427	-0.4179071079
H	6.3264623358	2.5785316009	0.7035437562
H	2.6181848354	1.1092384058	-1.8153159869
H	5.2110439111	-0.3811772522	0.3309212918
H	6.0211923251	-5.0077019838	1.0323963984
H	4.2820298898	-4.6500645023	1.2527593647
H	5.8959020304	-4.9976168204	3.4224288552
H	4.6619806927	-3.7138183732	3.4257030278
H	7.6130918862	-3.3962980038	2.6402107131
H	6.7430121217	-2.4679720352	3.8805323023
H	6.9107964494	-1.4474894837	1.3630119237
H	5.3610324235	-1.3638970526	2.2470840195
H	5.1808214227	-5.1116285817	-0.8192934885
H	2.0623355167	-5.3571379710	-3.7431661472
H	1.6264541278	-2.9223684520	-3.4896209289
H	2.0079732512	-0.9258413171	-2.2721988565
H	5.8537428478	-6.9619059456	-1.9968301948
H	4.8250713690	-8.4134595014	-2.1363845975
H	4.6408748468	-7.3359491974	-0.7247990771

H	3.2251211317	5.3105199506	-0.9026324451
H	4.2313367965	7.2684562575	0.2300291015
H	4.9242651529	7.1077981616	2.6089458535
H	4.5780459465	4.9875036987	3.8526496903
H	3.5551262198	3.0398822781	2.7262072140
H	0.1830295741	0.6958017709	2.1263158400
H	-1.8128383726	-0.0431428593	3.0939008258
H	-4.2632270827	0.0557086236	3.2252406605
H	-0.6888946640	5.8590899369	-0.9775461664
H	-0.4221647328	4.5637994018	-2.1799368638
H	-1.6133741348	6.1713969794	-3.5185169011
H	-2.6685661737	6.6390500898	-2.1668713159
H	-2.6360632428	3.9266042107	-3.6027513657
H	-4.0478718831	4.9940246920	-3.3994003744
H	-3.5990629884	2.9813457610	-1.6140804192
H	-4.1523476833	4.5839572915	-1.0506428603
N	0.3527621413	-0.2388418348	-2.2577368848
S	0.0605317708	1.0605075857	-3.1040909119
O	1.3496856750	1.7729518840	-3.3348568760
C	-0.3327665947	0.4374628084	-4.8551528067
F	-0.4861598876	1.4610462489	-5.7120748647
F	0.6578509120	-0.3500486943	-5.3093300503
F	-1.4707634817	-0.2812092667	-4.8499859684
O	-1.1247106448	1.8706559540	-2.7495513968
C	-0.5252803301	-0.7791559901	-1.2988875397
C	0.0966222319	-1.2795707687	-0.1486064868
C	-0.6343472401	-1.8762373938	0.8865611008
N	-0.0926571878	-2.3016058083	2.0983146795
S	1.3797917344	-1.9144502640	2.5048498380
O	2.5121549759	-2.7063888363	1.9642137313
C	1.2725489453	-2.4309570842	4.3172858423
F	0.3271172631	-1.7430852756	4.9741078668
F	1.0167028597	-3.7398618367	4.4404024500

F	2.4586662304	-2.1758865680	4.9024791599
O	1.6766607967	-0.4364533960	2.5874569235
C	-2.0263350131	-1.9938885647	0.7312275998
C	-2.6568251339	-1.4731471746	-0.3985786564
C	-4.1335301827	-1.4707053731	-0.4598302456
C	-4.8859143301	-2.5544689043	0.0022023847
C	-6.2769461265	-2.5000209355	-0.0046177220
C	-7.0606193511	-3.6555624146	0.5489533643
F	-8.2953002284	-3.7476170832	0.0043904474
F	-7.2386611963	-3.5363805482	1.8940993191
F	-6.4429669350	-4.8399340869	0.3450531528
C	-6.9511022182	-1.3712003180	-0.4562805161
C	-6.1993884808	-0.2939068441	-0.9194881393
C	-6.9213290563	0.9248933406	-1.4182533097
F	-6.1225159513	2.0128714776	-1.4845792332
F	-7.9736636256	1.2481733893	-0.6274404045
F	-7.4258212838	0.7341353165	-2.6667407344
C	-4.8119248092	-0.3391821603	-0.9305423362
C	-1.9149755741	-0.8708318794	-1.4239993257
H	1.1705393518	-1.1661304491	-0.0563635810
H	-2.6062327706	-2.4125934737	1.5483465831
H	-4.3798226935	-3.4477828966	0.3550652928
H	-8.0352337358	-1.3278097111	-0.4473934192
H	-4.2428563530	0.5199685884	-1.2689702765
H	-2.4113152589	-0.4900808127	-2.3096768773

Coordinates of optimized geometry of $lig_2 \cdot K7$

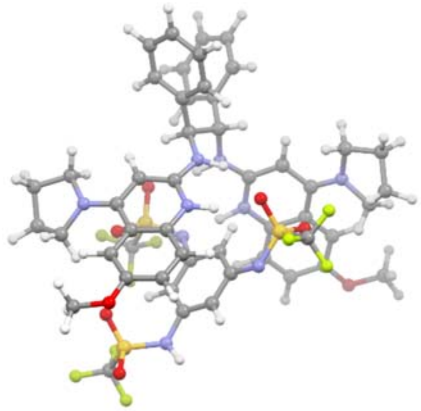

C	5.8453902796	6.4053618265	1.6125843436
O	4.7134260705	5.9691269148	2.3645472315
C	4.2317132298	4.7214826541	2.1110304805
C	4.7827199712	3.8385515936	1.1975272234
C	4.2124308166	2.5629925428	0.9991056990
C	4.6897595190	1.5865889360	0.0280918259
C	4.2814007571	0.2554074875	0.1876969069
C	3.1999475585	-0.0605860792	1.0144459892
N	2.5902534145	-1.2671532732	1.0500699057
C	2.9539489587	-2.4305662451	0.2630005671
C	1.6362558224	-3.1361121131	-0.1739725863
C	1.9168054846	-4.4660813530	-0.8467602299
C	2.3765459909	-4.5092378806	-2.1638208204
C	2.7041352632	-5.7258516136	-2.7529794467
C	2.5708998767	-6.9109453010	-2.0326032647
C	2.1004262622	-6.8740105276	-0.7231823511
C	1.7742856045	-5.6556774574	-0.1339400864
N	0.8961678962	-2.2187389286	-1.0262086798
C	-0.4538758266	-2.1617436119	-1.1016531677
C	-1.3096443241	-2.9740491908	-0.3586096460
C	-2.6844317783	-2.7091565513	-0.2896022529
N	-3.4373836256	-3.3696975267	0.6159668867
C	-4.7056238888	-2.8851762641	1.2143789173
C	-4.6856196996	-3.4658497613	2.6267772052

C	-3.9577838382	-4.7951929240	2.4307433180
C	-2.8329877481	-4.4046394033	1.4791465783
C	-3.2394563717	-1.7358585323	-1.2248586712
C	-4.6176413011	-1.5239155926	-1.4400938915
C	-5.0571635352	-0.5635004789	-2.3350402470
C	-4.1324724653	0.1899844486	-3.0779080136
C	-2.7868681301	-0.0358783002	-2.9260819054
C	-2.3311155598	-0.9996824673	-2.0142936853
N	-0.9762766827	-1.2078083439	-1.9019896151
O	-6.3649112167	-0.2717536891	-2.5668773409
C	-7.3390894318	-0.8909468757	-1.7247440573
C	3.8683688983	-3.3827071199	1.0111133639
C	4.9648761552	-3.9538263989	0.3674675373
C	5.7721315956	-4.8735864352	1.0313828781
C	5.4876463741	-5.2278417833	2.3468807981
C	4.3975367596	-4.6534462321	2.9968785623
C	3.5916477894	-3.7340899794	2.3337947973
N	2.6349648007	0.9023726283	1.7745189136
C	3.1369178312	2.1799302888	1.8246067234
C	2.5652869116	3.0953580932	2.7225790724
C	3.0949796980	4.3553405783	2.8544408936
N	5.5024742509	1.9222166322	-0.9965814647
C	5.5925227988	3.2720174952	-1.6074851579
C	5.7982637546	2.9857222078	-3.0930598298
C	6.6082433360	1.6899954091	-3.0773636472
C	5.9201630946	0.8963620823	-1.9731428666
H	6.7276602761	5.7848633823	1.8198001655
H	5.6345553936	6.4021858283	0.5343906515
H	6.0430917278	7.4287146732	1.9370519449
H	5.6723895760	4.1146192767	0.6534255549
H	4.6997030308	-0.5245656488	-0.4286432825
H	1.8390167677	-1.3906875548	1.7275057538
H	3.4599745564	-2.1041654706	-0.6548542394

H	1.0646247899	-3.3191720760	0.7472154399
H	2.4761901660	-3.5881812899	-2.7337212866
H	3.0636507012	-5.7498103803	-3.7780535306
H	2.8269975701	-7.8606755337	-2.4937250101
H	1.9862395382	-7.7949476099	-0.1583767733
H	1.4165188980	-5.6259600881	0.8930228685
H	1.4114705001	-1.4902271248	-1.5097396731
H	-0.8685829553	-3.7208589335	0.2815409807
H	-5.5695365350	-3.2856056829	0.6646358932
H	-5.6959383171	-3.5757135550	3.0303950583
H	-4.1067891810	-2.8124585721	3.2875256373
H	-4.6207974316	-5.5328892492	1.9623252058
H	-3.5695270111	-5.2187177873	3.3606927183
H	-2.4618726112	-5.2397568283	0.8726153062
H	-1.9937571809	-3.9725345698	2.0445175641
H	-5.3422079517	-2.1185338826	-0.9100743330
H	-4.4967869828	0.9438783027	-3.7690947989
H	-2.0613183831	0.5404203767	-3.4938369670
H	-0.3342580855	-0.5833582823	-2.3978569910
H	-7.3926681721	-1.9737534101	-1.9012855039
H	-8.2952976604	-0.4372329115	-1.9922579799
H	-7.1276296386	-0.6951721947	-0.6653927164
H	5.1826725980	-3.6851893730	-0.6639129670
H	6.6244412814	-5.3127605013	0.5205783329
H	6.1160939594	-5.9451646793	2.8671769635
H	4.1727279192	-4.9218168502	4.0254208442
H	2.7431891499	-3.2930848674	2.8527168306
H	1.6733228042	0.7187187259	2.1429259298
H	1.7098810189	2.7881247718	3.3181300395
H	2.6645279855	5.0716309448	3.5474398735
H	4.6803755619	3.8393465222	-1.4093489515
H	6.4574226758	3.8198889729	-1.2076105562
H	6.3060862967	3.8122752183	-3.5976932844

H	4.8276574288	2.8197781846	-3.5730031220
H	7.6532164808	1.8913779235	-2.8120560698
H	6.5900459272	1.1549815718	-4.0304092890
H	6.5725751299	0.1523852808	-1.5003464177
H	5.0282880873	0.3839529125	-2.3677196884
H	-4.7429390542	-1.7935614527	1.2019379971
N	-0.0719299928	0.3396740477	2.1142185308
S	-0.6679089935	-0.8364726979	2.9808805479
O	0.4146990134	-1.8309272991	3.2273751223
C	-0.9048902624	-0.1145359356	4.7227581491
F	-1.3122137922	-1.0574041955	5.5887739743
F	0.2473917938	0.4089607334	5.1778887984
F	-1.8285632824	0.8652196539	4.7027725441
O	-2.0122069069	-1.3381585580	2.6302572481
C	-0.8039436972	1.0695876771	1.1598302750
C	-0.1223583384	1.3261326999	-0.0350556590
C	-0.6981048993	2.0834645375	-1.0634362870
N	-0.1136025826	2.3230880688	-2.3039633326
S	1.2980157492	1.7244584272	-2.6744769836
O	2.5165402211	2.3336825240	-2.0879052256
C	1.3129685808	2.2707841522	-4.4817258721
F	1.2512173523	3.6039388430	-4.5923643710
F	2.4661033915	1.8551761780	-5.0406124443
F	0.2968096905	1.7329648595	-5.1712917810
O	1.3745878529	0.2202519496	-2.7761648705
C	-1.9961416638	2.5891184889	-0.8658402189
C	-2.6598925598	2.3326447945	0.3286021483
N	-3.9521674665	2.9287036933	0.4916099325
S	-5.3305467250	2.1074212155	0.9494928718
O	-6.4566947859	2.6584374260	0.1926641208
C	-5.5845430166	2.7671456416	2.7111030154
F	-4.5417982417	2.4625575977	3.4881626736
F	-5.7373238704	4.0946674893	2.6821112139

F	-6.6883275065	2.2082437321	3.2193760724
O	-5.0900027564	0.6751589358	1.1050176593
C	-2.0896619251	1.5857566221	1.3570146775
H	0.8631697573	0.8922975598	-0.1624466817
H	-2.4756535400	3.1534330935	-1.6605604776
H	-4.1860567934	3.6463813521	-0.1890430321
H	-2.6200892430	1.4086886543	2.2831291623

Catalyst Synthesis

Ligands lig_1, lig_2, lig_3, lig_4, lig_5, and lig_8 were prepared from the previously published procedure.[14] Ligands lig_7, lig_8 were prepared in one step from the precursor S1.

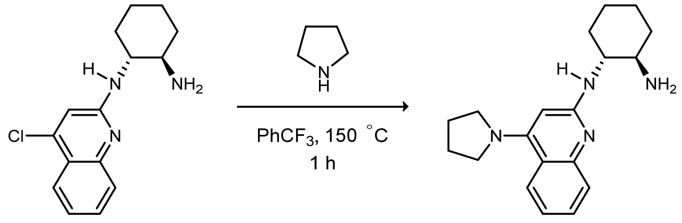

(1*R*,2*R*)-*N*¹-(4-(Pyrrolidin-1-yl)quinolin-2-yl)cyclohexane-1,2-diamine (S1). A 2-5 mL microwave vial equipped with a stir bar was charged with (1*R*,2*R*)-*N*¹-(4-chloroquinolin-2-yl)cyclohexane-1,2-diamine[15] (275 mg, 1 mmol), pyrrolidine (330 µL, 4 mmol), and trifluoromethylbenzene (3 mL). The vial was sealed, and the suspension was heated with stirring at 150 °C in the microwave for 60 min at constant temperature. The reaction mixture was diluted with dichloromethane and transferred to a round-bottomed flask for evaporation. The resulting crude was dissolved in dichloromethane and stirred with 4 M NaOH (~20 mL) overnight. The contents of the flask were then transferred to a separatory funnel where the aqueous layer was extracted with DCM. The resulting organic layer was dried and concentrated. The crude was purified by silica gel chromatography (SiO_2, 2-30% MeOH in DCM with 1% acetic acid). The resulting ammonium salts were dissolved in a minimal amount of MeOH and triturated with diethyl ether. The obtained white solid was filtered and washed with diethyl ether. The free base was liberated by addition of 4 M NaOH, and then extracted with DCM. The resulting organic layer was dried and concentrated to afford a white solid (132 mg, 43%). Mp 101-103 °C; $[\alpha]_D^{20}$ +20.1 (*c* 1.00, $CHCl_3$); R_f = 0.1 (10% MeOH/DCM with 1% AcOH); IR (film) 3278, 2929, 2855, 1588, 1534, 1430 cm^{-1}; ^1H NMR (400 MHz, $CDCl_3$) δ 7.97 (d, *J* = 8.4 Hz, 1H), 7.55 (d, *J* = 8.3 Hz, 1H), 7.40 (dd, *J* = 7.2, 7.2 Hz, 1H), 7.03 (dd, *J* = 7.3, 7.3 Hz, 1H), 5.79 (s, 1H), 4.43 (br s, 1H), 3.55 (s, 5H), 2.55-2.41 (m, 1H), 2.05-1.89 (m, 2H), 1.80-1.65 (m, 5H), 1.75-1.37 (m, 2H), 1.47-1.06 (m, 5H); ^{13}C NMR (150 MHz, $CDCl_3$) ppm 157.8, 154.4, 149.0, 129.2, 125.7, 125.1, 119.9, 118.2, 89.9, 58.1, 56.2, 52.2 (2C), 34.9, 33.0, 25.9 (2C), 25.4, 25.1; HRMS (ESI): Exact mass calcd for $C_{19}H_{27}N_4$ $[M+H]^+$ 311.2230, found 311.2229.

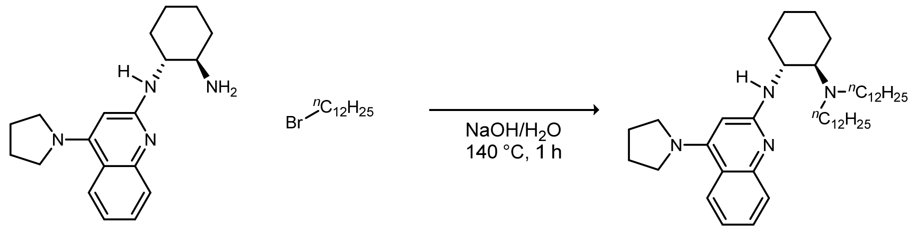

(1*R*,2*R*)-*N*¹,*N*¹-Didodecyl-*N*²-(4-(pyrrolidin-1-yl)quinolin-2-yl)cyclohexane-1,2-diamine (lig_7). A 0.5-2 mL microwave vial equipped with a stir bar was charged with (1*R*,2*R*)-*N*¹-(4-(pyrrolidin-1-yl)quinolin-2-yl)cyclohexane-1,2-diamine (50 mg, 161 μmol), 1-bromododecane (160 μL, 644 μmol), 4 N NaOH (200 μL), and water (300 μL). The vial was sealed, and the suspension was heated with stirring at 140 °C in the microwave for 1 h at constant temperature. The reaction mixture was diluted with DCM/water and transferred to a separatory funnel. The aqueous layer was extracted with DCM and the combined organic layers were dried and concentrated. The crude was purified by silica gel chromatography (SiO_2, 20-80% acetone in hexanes) to afford a light yellow oil (41 mg, 39%). $[\alpha]^{20}_{D}$ -1.8 (*c* 1.00, $CHCl_3$); R_f = 0.2 (40% acetone/hexanes); IR (film) 3353, 2924, 2852, 1591, 1519, 1465 cm^{-1}; ^1H NMR (400 MHz, $CDCl_3$) δ 7.97 (d, *J* = 8.0 Hz, 1H), 7.65 (d, *J* = 8.2 Hz, 1H), 7.38 (dd, *J* = 7.0, 7.0 Hz, 1H), 7.04 (dd, *J* = 7.3, 7.3 Hz, 1H), 5.75 (s, 1H), 5.59 (s, 1H), 3.60-3.47 (m, 4H), 3.38-3.24 (m, 1H), 3.03 (d, *J* = 11.4 Hz, 1H), 2.52-2.43 (m, 2H), 2.34-2.24 (m, 2H), 2.03-1.93 (m, 4H), 1.92-1.79 (m, 1H), 1.75-1.64 (m, 1H), 1.38-1.08 (m, 46H), 0.88 (t, *J* = 6.6 Hz, 6H); ^{13}C NMR (150 MHz, $CDCl_3$) ppm 158.9, 153.7, 150.4, 128.4, 127.4, 124.8, 119.6, 119.0, 93.3, 77.4, 63.9, 52.8, 52.0, 49.6, 33.8, 32.1, 30.0, 29.9, 29.8, 29.5, 29.3, 27.7, 26.3, 25.8, 24.8, 23.4, 22.8, 14.3; HRMS (ESI): Exact mass calcd for $C_{43}H_{75}N_4$ $[M+H]^+$ 647.5986, found 647.5986.

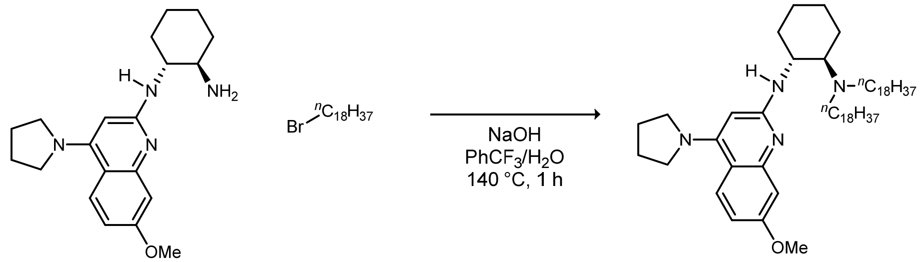

(1*R*,2*R*)-*N*1-(7-Methoxy-4-(pyrrolidin-1-yl)quinolin-2-yl)-*N*²,*N*²-dioctadecylcyclohexane-1,2-diamine (lig_8). A 2-5 mL microwave vial equipped with a stir bar was charged with (1*R*,2*R*)-*N*¹-(6-methoxy-4-(pyrrolidin-1-yl)quinolin-2-yl)cyclohexane-1,2-diamine (285 mg, 840 μmol), 4 N aq NaOH (750 μL), and trifluoromethylbenzene (1 mL). The vial was sealed, and the suspension was heated with stirring at 140 °C in the microwave for 1 h at constant temperature. The reaction mixture was diluted with DCM/water and transferred to a separation funnel. The aqueous layer was extracted with DCM, and the combined organic layers were dried and concentrated. The crude residue was purified by silica gel chromatography (SiO_2, 10-30% acetone in hexanes) to afford a light yellow oil (456 mg, 64%). $[\alpha]^{20}_{D}$ +4.0 (*c* 1.00, $CHCl_3$); R_f = 0.2 (30% acetone/hexanes); IR (film) 3349, 2923, 2852, 1591, 1521, 1489, 1466, 1427 m^{-1}; ^1H NMR (400 MHz, $CDCl_3$) δ 7.83 (d, *J* = 9.2 Hz, 1H), 7.03 (d, *J* = 2.5 Hz, 1H), 6.69 (dd, *J* = 9.2, 2.6 Hz, 1H), 5.65 (s, 1H), 5.55 (br s, 1H), 3.86 (s, 3H), 3.58-3.48 (m, 4H), 3.49-3.42 (m, 1H), 2.97 (d, *J* = 10.9 Hz, 1H), 2.53-2.41 (m, 3H), 2.34-2.23 (m, 2H), 2.02-1.91 (m, 4H), 1.92-1.78 (m, 2H), 1.76-1.64 (m, 1H), 1.41-1.01 (m, 68H), 0.87 (t, *J* = 6.5 Hz, 6H); ^{13}C NMR (150 MHz, $CDCl_3$) ppm 157.9, 152.9, 149.2, 148.9, 126.3, 122.0, 118.5, 111.2, 101.1, 64.2, 55.4, 53.0, 52.1, 49.8, 33.7, 32.1, 30.0, 29.9, 29.8, 29.6, 29.5, 29.3, 27.8, 26.2, 26.0, 25.9, 24.8, 23.7, 22.9, 22.8, 14.3; HRMS (ESI): Exact mass calcd for $C_{56}H_{101}N_4O$ $[M+H]^+$ 845.7970, found 845.7977.

General Procedure 1 for Preparation of *N*-Aryl Triflamides

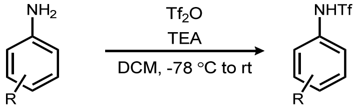

Aniline (1 equiv) was dissolved in DCM (0.2 M) in a round-bottom flask. Triethylamine (1.5 equiv) was then added and the mixture was cooled to -78 °C. Trifluoromethanesulfonic anhydride (1.1 equiv) was added dropwise, the mixture was slowly warmed to room temperature over 20 minutes, and then stirred overnight. The reaction was monitored by mass spectrometry. After completion of the reaction, aq 4 N NaOH was added, the aqueous layer was separated, and then 2 N aq HCl was added to adjust the pH to 1-2. This mixture was extracted with ethyl acetate, and the organic layers were dried and concentrated under reduced pressure. The crude material was purified by flash silica gel chromatography using acetone/hexanes as the eluent. The product was then further purified by distillation under reduced pressure.

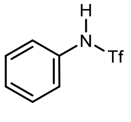

1,1,1-Trifluoro-*N*-phenylmethanesulfonamide (A1). Corresponding aniline (336 μL, 3.6 mmol) was used according to the General Procedure 1, delivering the product as a white solid (700 mg, 69%). ^{1}H NMR (400 MHz, $CDCl_3$) δ 7.39 (m, 2H), 7.31 (m, 3H), 6.82 (br s, 1H). The characterization data (^{1}H NMR) was in agreement with the literature.[16]

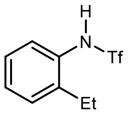

***N*-(2-Ethylphenyl)-1,1,1-trifluoromethanesulfonamide (A2)**. Corresponding aniline (447 μL, 3.6 mmol) was used according to the General Procedure 1, delivering the product as a light red oil (780 mg, 85%). R_f = 0.2 (5% acetone/hexanes); IR (film) 3286, 3252, 2975, 1489, 1427 cm^{-1}; H NMR (400 MHz, $CDCl_3$) δ 7.41 (d, *J* = 7.8 Hz, 1H), 7.31 (m, 2H), 7.23 (m, 1H), 6.45 (s, 1H), 2.73 (q, *J* = 7.6 Hz, 2H), 1.27 (t, *J* = 7.5 Hz, 3H); ^{13}C NMR (150 MHz, $CDCl_3$) ppm 139.6, 131.4, 129.4, 128.8, 127.2, 126.5, 119.9 (q, $^{1}J_{CF}$ = 330.2 Hz), 24.0, 14.3; ^{19}F NMR (282 MHz, $CDCl_3$) δ -75.8; Exact mass calcd for $C_9H_9F_3NO_2S$ [M-H]$^-$ 252.0312, found 252.0312.

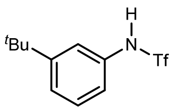

***N*-(3-(*tert*-Butyl)phenyl)-1,1,1-trifluoromethanesulfonamide (A3)**. Corresponding aniline (539 mg, 3.6 mmol) was used according to the General Procedure 1, delivering the product as a light brown liquid (430 g, 42%). Mp 145-148 °C; R_f = 0.2 (5% acetone/hexanes); IR (film) 3649, 3568, 3292, 2966,1608, 1588, 1462, 1414 cm^{-1}; ^{1}H NMR (400 MHz, $CDCl_3$) δ 7.33 (m, 3H), 7.19-7.09 (br s, 1H), 7.10 (d, *J* = 7.8 Hz, 1H), 1.34 (s, 9H); ^{13}C NMR (150 MHz, $CDCl_3$) ppm 153.4, 133.5, 129.4, 124.8, 121.1, 120.9, 120.1 (q, $^{1}J_{CF}$ = 321.2 Hz), 34.9, 31.2; ^{19}F NMR (282 MHz, $CDCl_3$) δ -75.3; HRMS (ESI): Exact mass calcd for $C_{11}H_{13}F_3NO_2S$ [M-H]$^-$ 280.0625, found 280.0625

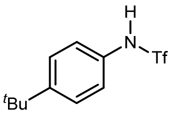

***N*-(4-(*tert*-Butyl)phenyl)-1,1,1-trifluoromethanesulfonamide (A4)**. Corresponding (230 μL, 1.5 mmol) was used according to the General Procedure 1, delivering the product as a white solid (118 mg, 29%). ^{1}H NMR (400 MHz, $CDCl_3$) δ 7.51 (d, *J* = 8.7 Hz, 2H), 7.32 (d, $^{1}J_{CF}$ = 8.6 Hz, 2H), 1.33 (s, 9H) [N*H* not observed]. The characterization data (^{1}H NMR) was in agreement with the literature.[16]

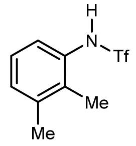

***N*-(2,3-Dimethylphenyl)-1,1,1-trifluoromethanesulfonamide (A5)**. Corresponding aniline (440 μL, 3.6 mmol) was used according to the General Procedure 1, delivering the product as a white solid (800 mg, 87%). Mp 122-123 °C; R_f = 0.2 (5% acetone/hexanes); IR (film) 3247, 2948, 1579, 1467 cm^{-1}; ^{1}H NMR (400 MHz, $CDCl_3$) δ 7.23 (d, *J* = 7.9 Hz, 1H), 7.15 (m, 2H), 6.51 (s, 1H), 2.33 (s, 3H), 2.28 (s, 3H); ^{13}C NMR (150 MHz, $CDCl_3$) ppm 138.7, 133.7, 131.8, 130.4, 126.4, 125.0, 120.0, 20.7, 14.3; ^{19}F NMR (282 MHz, $CDCl_3$) δ -75.8; HRMS (ESI): Exact mass calcd for $C_9H_9F_3NO_2S$ [M-H]$^-$ 252.0312, found 252.0313.

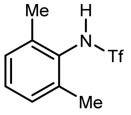

***N*-(2,6-Dimethylphenyl)-1,1,1-trifluoromethanesulfonamide (A6)**. Corresponding aniline (540 µL, 4.4 mmol) was used according to the General Procedure 1, delivering the product as a white solid (486 mg, 44%). Mp = 83-85 °C; R_f = 0.53 (20% EtOAc/hexanes); IR (film) 3285, 1419, 1371, 1135 cm^{-1}; ^1H NMR (400 MHz, $CDCl_3$) δ 7.23 (t, *J* = 7.5 Hz, 1H), 7.16 (d, *J* = 7.5 Hz, 2H), 6.74 (br s, 1H), 2.43 (s, 6H); ^{13}C NMR (100 MHz, $CDCl_3$) ppm 138.1, 130.6, 129.3, 129.1, 119.6 (q, $^1J_{CF}$ = 322 Hz), 18.7; ^{19}F NMR (376 MHz, $CDCl_3$) ppm -75.4; HRMS (CI): Exact mass calcd for $C_9H_{11}F_3NO_2S$ $[M+H]^+$ 254.0457, found 254.0456

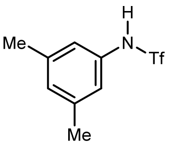

***N*-(3,5-Dimethylphenyl)-1,1,1-trifluoromethanesulfonamide (A7)**. Corresponding aniline (550 µL, 4.4 mmol) was used according to the General Procedure 1, delivering the product as a white solid (500 mg, 45%). ^1H NMR (400 MHz, $CDCl_3$) δ 7.57 (s, 1H), 6.89 (s, 1H), 6.87 (s, 2H), 2.27 (s, 6H); The characterization data (^1H NMR) was in agreement with the literature.[17]

***N*-(2,6-Diisopropylphenyl)-1,1,1-trifluoromethanesulfonamide (A8)**. Corresponding aniline (213 µL, 1.1 mmol) was used according to the General Procedure 1, delivering the product as a colorless liquid (132 mg, 38%). Mp = 65-67 °C; R_f = 0.73 (25% EtOAc/hexanes); IR (film) 3275, 2963, 1420, 1373, 1227, 1194, 1134, 955 cm^{-1}; ^1H NMR (400 MHz, $CDCl_3$) δ 7.28 (t, *J* = 7.7 Hz, 1H), 7.13 (d, *J* = 8.1 Hz, 2H), 6.56 (br s, 1H), 3.28 (septet, *J* = 6.8 Hz, 2H), 1.14 (d, *J* = 6.8 Hz, 12H); ^{13}C NMR (100 MHz, $CDCl_3$) ppm 148.7, 130.2, 127.1, 124.6, 119.8 (q, $^1J_{CF}$ = 322.2 Hz), 28.9, 24.1; ^{19}F NMR (376 MHz, $CDCl_3$) ppm -75.9; HRMS (CI): Exact mass calcd for $C_{13}H_{19}F_3NO_2S$ $[M+H]^+$ 310.1083, found 310.1078.

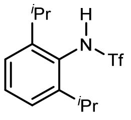

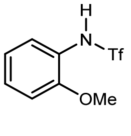

1,1,1-Trifluoro-*N*-(2-methoxyphenyl)methanesulfonamide (B1). Corresponding aniline (405 µL, 3.6 mmol) was used according to the General Procedure 1, delivering the product as a light brown solid (650 mg, 87%). ^1H NMR (400 MHz, $CDCl_3$) δ 7.50 (dd, *J* = 8.0, 1.5 Hz, 1H), 7.21 (m, 1H), 7.09 (s, 1H), 6.98 (m, 1H), 6.92 (dd, *J* = 8.2, 8.2 Hz, 1H), 3.87 (s, 3H). The characterization data (^1H NMR) was in agreement with the literature.[18]

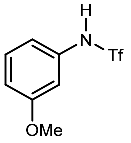

1,1,1-Trifluoro-*N*-(3-methoxyphenyl)methanesulfonamide (B2). Corresponding aniline (445 mg, 3.6 mmol)was used according to the General Procedure 1, delivering the product as a light brown solid (720 g, 96%). ^1H NMR (400 MHz, $CDCl_3$) δ 7.74 (s, 1H), 7.20 (d, *J* = 8.4 Hz, 1H), 6.80 (m, 3H), 3.72 (s, 3H). The characterization data (^1H NMR) was in agreement with the literature.[18]

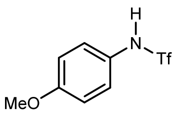

1,1,1-Trifluoro-*N*-(4-methoxyphenyl)methanesulfonamide (B3). Corresponding aniline (445 mg, 3.6 mmol) was used according to the General Procedure 1, delivering the product as a yellow oil (480 mg, 63%). ^1H NMR (400 MHz, $CDCl_3$) δ 7.20 (d, *J* = 9.0 Hz, 2H), 7.01 (s, 1H), 6.79 (d, *J* = 9.0 Hz, 2H), 3.80 (s, 3H). The characterization data (^1H NMR) was in agreement with the literature.[19]

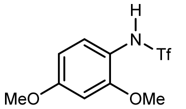

1,1,1-Trifluoro-*N*-(2,4-dimethoxyphenyl)methanesulfonamide (B4). Corresponding aniline (554 mg, 3.6 mmol) was used according to the General Procedure 1, delivering the product as a white solid (420 mg, 41%). Mp 58-62 °C; R_f = 0.25 (10% acetone/hexanes); IR (film) 3275, 2945, 1614, 1512, 1441cm^{-1};^1H NMR (400 MHz, $CDCl_3$) δ 7.36 (d, *J* = 8.4 Hz, 1H), 6.77 (br, s, 1H), 6.53-6.42 (m, 2H), 3.87 (s, 3H), 3.77 (s, 3H); ^{13}C NMR (150 MHz, $CDCl_3$) ppm 159.8, 152.9, 125.4, 119.8 (q, $^1J_{CF}$ = 323.2 Hz), 116.1, 104.5, 99.1, 56.0, 55.7; ^{19}F NMR (282 MHz, $CDCl_3$) δ -76.5; HRMS (ESI): Exact mass calcd for $C_9H_9F_3NO_4S$ $[M-H]^-$ 284.0210, found 284.0211.

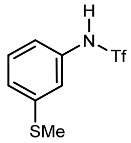

1,1,1-Trifluoro-*N*-(3-(methylthio)phenyl)methanesulfonamide (B5). Corresponding aniline (503 mg, 3.6 mmol) was used according to the General Procedure 1, delivering the product as a yellow liquid (760 mg, 78%). R_f = 0.3 (10% acetone/hexanes); IR (film) 3286, 2926, 1593, 1478, 1414 cm^{-1};^1H NMR (400 MHz, $CDCl_3$) δ 7.76 (s, 1H), 7.29 (dd, *J* = 8.2, 8.2 Hz, 1H), 7.17 (m, 2H), 7.05 (dd, *J* = 7.6, 1.8 Hz, 1H), 2.51 (s, 3H); ^{13}C NMR (150 MHz, $CDCl_3$) ppm 141.0, 134.3, 129.8, 125.2,

120.7, 119.8 (q, ${}^{1}J_{CF}$ = 324.1 Hz), 119.6, 15.5; ^{19}F NMR (282 MHz, $CDCl_3$) δ -75.1; HRMS (ESI): Exact mass calcd for $C_8H_7F_3NO_2S_2$ $[M-H]^-$ 269.9876, found 269.9877.

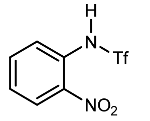

1,1,1-Trifluoro-*N*-(2-nitrophenyl)methanesulfonamide (B6). Corresponding aniline (500 mg, 3.6 mmol) was used according to the General Procedure 1, delivering the product as a yellow solid (465 mg, 47%). Mp 64-68 °C; R_f = 0.25 (10% acetone/hexanes); IR (film) 3239, 1610, 1586, 1536, 1489 cm^{-1}; ^{1}H NMR (400 MHz, $CDCl_3$) δ 10.04 (s, 1H), 8.31 (dd, *J* = 8.4, 1.5 Hz, 1H), 7.89 (dd, *J* = 8.4, 1.2 Hz, 1H), 7.71 (ddd, *J* = 8.9, 7.6, 1.7 Hz, 1H), 7.40 (ddd, *J* = 8.6, 7.4, 1.3 Hz, 1H); ^{13}C NMR (150 MHz, $CDCl_3$) ppm 138.2, 136.4, 131.5, 126.5, 126.1, 121.9, 119.6 (q, ${}^{1}J_{CF}$ = 323.1 Hz); ^{19}F NMR (282 MHz, $CDCl_3$) δ -75.8; HRMS (ESI): Exact mass calcd for $C_8H_7F_3NO_4S_2$ $[M-H]^-$ 269.9849, found 268.9850.

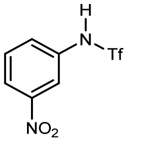

1,1,1-Trifluoro-*N*-(3-nitrophenyl)methanesulfonamide (B7). Corresponding aniline (500 mg, 3.6 mmol) was used according to the General Procedure 1, delivering the product as a yellow oil (740 g, 76%). ^{1}H NMR (400 MHz, $CDCl_3$) δ 8.19 (ddd, *J* = 7.9, 1.8, 1.5 Hz, 1H), 8.15 (t, *J* = 2.0 Hz, 1H), 7.67 (ddd, *J* = 8.1, 1.6, 1.5 Hz, 1H), 7.61 (dd, *J* = 7.9, 7.9 Hz, 1H), 6.94 (s, 1H). The characterization data (^{1}H NMR) was in agreement with the literature.[18]

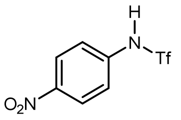

1,1,1-Trifluoro-*N*-(4-nitrophenyl)methanesulfonamide (B8). Corresponding aniline (500 mg, 3.6 mmol) was used according to the General Procedure 1, delivering the product as a yellow oil (1.2 g, 87%). ^{1}H NMR (400 MHz, $CDCl_3$) δ 8.27 (d, *J* = 9.1 Hz, 2H), 7.44 (d, *J* = 9.1 Hz, 2H). All other spectroscopic data were in agreement with the literature.[19]

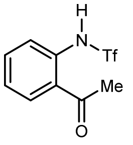

***N*-(2-Acetylphenyl)-1,1,1-trifluoromethanesulfonamide (C1)**. Corresponding aniline (488 mg, 3.6 mmol) was used according to the General Procedure 1, delivering the product as a light yellow solid (900 mg, 93%). ^{1}H NMR (400 MHz, $CDCl_3$) δ 7.96 (dd, *J* = 8.0, 1.4 Hz, 1H), 7.79 (d, *J* = 8.0 Hz, 1H), 7.57 (ddd, *J* = 8.7, 7.6, 1.6 Hz, 1H), 7.29 (d, *J* = 8.1 Hz, 1H), 2.70 (s, 3H). The characterization data (^{1}H NMR) was in agreement with the literature.[20]

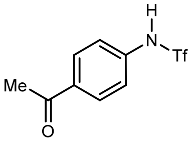

***N*-(2-Acetylphenyl)-1,1,1-trifluoromethanesulfonamide (C2)**. Corresponding aniline (488 mg, 3.6 mmol) was used according to the General Procedure 1, delivering the product as a white solid (210 mg, 21%). ^{1}H NMR (400 MHz, $CDCl_3$) δ 8.05 (d, *J* = 8.6 Hz, 2H), 7.52 (d, *J* = 8.5 Hz, 2H), 2.63 (s, 3H). The characterization data (^{1}H NMR) was in agreement with the literature.[21]

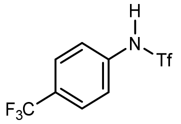

1,1,1-Trifluoro-*N*-(4-(trifluoromethyl)phenyl)methanesulfonamide (C3). Corresponding aniline (455 μL, 3.6 mmol) was used according to the General Procedure 1, delivering the product as a white solid (520 mg, 49%). ^{1}H NMR (400 MHz, $CDCl_3$) δ 7.64 (d, *J* = 8.6 Hz, 2H), 7.39 (d, *J* = 8.5 Hz, 2H), 6.79 (s, 1H). The characterization data (^{1}H NMR) was in agreement with the literature.[22]

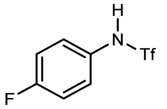

1,1,1-Trifluoro-*N*-(4-fluorophenyl)methanesulfonamide (C4). Corresponding aniline (350 μL, 3.6 mmol) was used according to the General Procedure 1, delivering the product as a light yellow solid (560 mg, 64%). ^{1}H NMR (400 MHz, $CDCl_3$) δ 7.25 (dd, *J* = 9.0, 4.6 Hz, 2H), 7.21 (br s, 1H), 7.07 (dd, *J* = 8.3, 8.3 Hz, 2H). The characterization data (^{1}H NMR) was in agreement with the literature.[18]

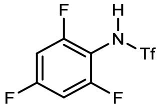

1,1,1-Trifluoro-*N*-(2,4,6-trifluorophenyl)methanesulfonamide (C5). Corresponding aniline (532 mg, 3.6 mmol) was used according to the General Procedure 1, delivering the product as a colorless liquid (285 mg, 28%). R_f = 0.3 (10% acetone/hexanes); IR (film) 3117, 3093, 1642, 1611, 1504, 1454, 1430 cm^{-1}; ^{1}H NMR (400 MHz, $CDCl_3$) δ 6.89 (dd, *J* = 8.4, 8.4 Hz, 2H) [N*H* not observed]; ^{13}C NMR (150 MHz, $CDCl_3$) ppm 165.2 (d, ${}^{1}J_{CF}$ = 270.8 Hz), 160.7 (d, ${}^{1}J_{CF}$ = 261.1 Hz, 2C), 119.3

(q, $^1J_{CF}$ = 327.4 Hz), 102.1 (d, $^2J_{CF}$ = 17.0 Hz, 2C); ^{19}F NMR (282 MHz, $CDCl_3$) δ -71.6, -96.4, -108.4; HRMS (ESI): Exact mass calcd for $C_7H_2F_6NO_2S$ $[M-H]^-$ 277.9716, found 277.9718.

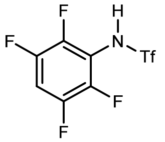

1,1,1-Trifluoro-*N*-(2,3,5,6-tetrafluorophenyl)methanesulfonamide (C6). Corresponding aniline (393 μL, 3.6 mmol) was used according to the General Procedure 1, delivering the product as a colorless liquid (110 mg, 12%). R_f = 0.25 (10% acetone/hexanes); IR (film) 3406, 1529, 1484, 1443 cm^{-1}; ^{1}H NMR (400 MHz, $CDCl_3$) δ 6.98 (tt, *J* = 17.0, 7.2 Hz, 1H), 6.43 (br s, 1H); ^{13}C NMR (150 MHz, $CDCl_3$) ppm 147.2 (ddd, $^1J_{CF}$ = 246.5 Hz, $^2J_{CF}$ = 12.0 Hz, $^3J_{CF}$ = 4.5 Hz, 2C), 143.0 (dd, $^1J_{CF}$ = 247.0 Hz, $^2J_{CF}$ = 15.3 Hz, 2C), 124.7 (q, $^1J_{CF}$ = 335.8 Hz), 119.2 (t, $^2J_{CF}$ = 14.6 Hz); ^{19}F NMR (282 MHz, $CDCl_3$) δ -78.4, -137.2, -150.5; HRMS (ESI): Exact mass calcd for $C_7HF_7NO_2S$ $[M-H]^-$ 295.9622, found 295.9619.

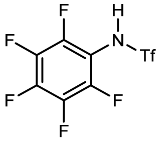

1,1,1-Trifluoro-*N*-(perfluorophenyl)methanesulfonamide (C7). Corresponding aniline (200 mg, 1.1 mmol) was used according to the General Procedure 1, delivering the product as a light brwon solid (115 mg, 33%). ^{1}H NMR (400 MHz, DMSO-d^6) δ 12.31 (s, 1H). The characterization data (^{1}H NMR) was in agreement with the literature.[23]

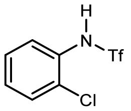

***N*-(2-Chlorophenyl)-1,1,1-trifluoromethanesulfonamide (C8)**. Corresponding aniline (380 μL, 3.6 mmol) was used according to the General Procedure 1, delivering the product as a white solid (920 mg, 98%). ^{1}H NMR (400 MHz, $CDCl_3$) δ 7.83 (dd, *J* = 8.0, 1.4 Hz, 1H), 7.58 (dd, *J* = 8.2, 1.4 Hz, 1H), 7.37 (ddd, *J* = 7.5, 7.5, 1.4 Hz, 1H), 6.98 (ddd, *J* = 7.5, 7.5, 1.4 Hz, 1H). The characterization data (^{1}H NMR) was in agreement with the literature.[18]

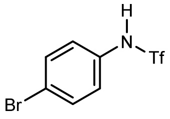

***N*-(4-Bromophenyl)-1,1,1-trifluoromethanesulfonamide (D1)**. Corresponding aniline (623 mg, 3.6 mmol) was used according to the General Procedure 1, delivering the product as a brown oil (340 mg, 31%). ^{1}H NMR (400 MHz, $CDCl_3$) δ 7.53 (d, *J* = 8.8 Hz, 2H), 7.15 (d, *J* = 8.8 Hz, 2H) [N*H* not observed]. The characterization data (^{1}H NMR) was in agreement with the literature.[18]

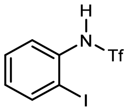

1,1,1-Trifluoro-*N*-(2-iodophenyl)methanesulfonamide (D2). Corresponding aniline (793 mg, 3.6 mmol) was used according to the General Procedure 1, delivering the product as a brown oil (802 mg, 63%). ^{1}H NMR (400 MHz, $CDCl_3$) δ 8.01 (dd, *J* = 8.0, 1.3 Hz, 1H), 7.45 (m, 2H), 7.21 (ddd, *J* = 7.8, 7.3, 1.9 Hz, 1H). The characterization data (^{1}H NMR) was in agreement with the literature.[24]

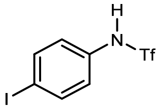

1,1,1-Trifluoro-*N*-(4-iodophenyl)methanesulfonamide (D3). Corresponding aniline (793 mg, 3.6 mmol) was used according to the General Procedure 1, delivering the product as a brown solid (209 mg, 39%). ^{1}H NMR (400 MHz, $CDCl_3$) δ 7.72 (d, *J* = 8.6 Hz, 2H), 7.01 (d, *J* = 8.6 Hz, 2H), 6.72 (s, 1H). The characterization data (^{1}H NMR) was in agreement with the literature.[25]

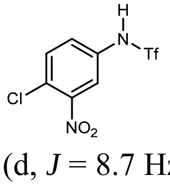

***N*-(4-Chloro-3-nitrophenyl)-1,1,1-trifluoromethanesulfonamide (D4)**. Corresponding aniline (300 mg, 1.7 mmol) was used according to the General Procedure 1, delivering the product as a yellow solid (131 mg 25%). Mp 91-92 °C; R_f = 0.2 (10% acetone/hexanes); IR (film) 3105, 2923, 1600, 1574, 1474, 1450, 1427 cm^{-1}; ^{1}H NMR (400 MHz, $CDCl_3$) δ 7.97 (d, *J* = 2.6 Hz, 1H), 7.74 (d, *J* = 8.7 Hz, 1H), 7.57 (dd, *J* = 8.7, 2.6 Hz, 1H) [N*H* not observed]; ^{13}C NMR (150 MHz, $CDCl_3$) ppm 148.2, 135.4, 133.7, 132.1, 131.0, 128.4, 119.7 (q, $^1J_{CF}$ = 328.5 Hz); ^{19}F NMR (282 MHz, $CDCl_3$) δ -70.3; HRMS (ESI): Exact mass calcd for $C_7H_3ClF_3N_2O_4S$ $[M-H]^-$ 302.9460, found 302.9462.

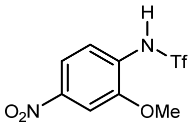

1,1,1-Trifluoro-*N*-(2-methoxy-4-nitrophenyl)methanesulfonamide (D5). Corresponding aniline (608 mg, 3.6 mmol) was used according to the General Procedure 1, delivering the product as a brown oil (820 mg, 76%). R_f = 0.15 (10% acetone/hexanes); IR (film) 3104, 1589, 1543, 1445, 1422 cm^{-1}; ^{1}H NMR (400 MHz, $CDCl_3$) δ 7.94 (m, 2H), 7.55 (d, *J* = 9.3 Hz, 1H), 4.06 (s, 3H) [N*H* not observed]; ^{13}C NMR (150 MHz, $CDCl_3$) ppm 157.9, 151.1, 133.2, 126.1, 119.7 (q, $^1J_{CF}$ =

327.4 Hz), 116.1, 108.0, 57.1; ^{19}F NMR (282 MHz, $CDCl_3$) δ -71.2; HRMS (ESI): Exact mass calcd for $C_8H_6F_3N_2O_5S$ $[M-H]^-$ 298.9955, found 298.9956.

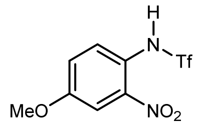

1,1,1-Trifluoro-*N*-(4-methoxy-2-nitrophenyl)methanesulfonamide (D6). Corresponding aniline (608 mg, 3.6 mmol) was used according to the General Procedure 1, delivering the product as a brown oil (452 mg, 42%). R_f = 0.2 (10% acetone/hexanes); IR (film) 3278, 3104, 3018, 2978, 2947, 1539, 1577, 1504, 1462, 1444, 1420 cm^{-1}; ^{1}H NMR (400 MHz, $CDCl_3$) δ 7.75 (d, J = 9.2 Hz, 1H), 7.69 (d, J = 3.0 Hz, 1H), 7.25 (dd, J = 9.2, 3.0 Hz, 1H), 3.88 (s, 3H) [N*H* not observed]; ^{13}C NMR (150 MHz, $CDCl_3$) ppm 157.8, 140.3, 125.2, 123.3, 122.7, 119.8 (q, $^1J_{CF}$ = 325.3 Hz), 110.0, 56.2; ^{19}F NMR (282 MHz, $CDCl_3$) δ -75.8; HRMS (ESI): Exact mass calcd for $C_8H_6F_3N_2O_5S$ $[M-H]^-$ 298.9955, found 298.9954.

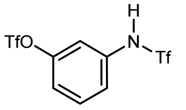

3-((Trifluoromethyl)sulfonamido)phenyl trifluoromethanesulfonate (D7). Corresponding aniline (395 mg, 3.6 mmol) was used according to the General Procedure 1, delivering the product as a brown oil (171 mg, 12%). R_f = 0.2 (5% acetone/hexanes); IR (film) 3303, 2924, 1613, 1596, 1489, 1426 cm^{-1}; ^{1}H NMR (400 MHz, $CDCl_3$) δ 7.51 (dd, J = 8.2, 8.2 Hz, 1H), 7.31 (d, J = 8.2 Hz, 1H), 7.25 (m, 2H) [N*H* not observed]; ^{13}C NMR (150 MHz, $CDCl_3$) ppm 149.9, 136.0, 131.4, 122.7, 120.2, 119.6 (q, $^1J_{CF}$ = 323.1 Hz), 118.7 (q, $^1J_{CF}$ = 320.1 Hz), 116.2; ^{19}F NMR (282 MHz, $CDCl_3$) δ -72.6, -75.4; HRMS (ESI): Exact mass calcd for $C_8H_4F_6NO_5S_2$ $[M-H]^-$ 371.9441, found 371.944.

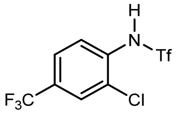

***N*-(2-Chloro-4-(trifluoromethyl)phenyl)-1,1,1-trifluoromethanesulfonamide (D8)**. Corresponding aniline (200 mg, 1.0 mmol) was used according to the General Procedure 1, delivering the product as a pink solid (170 mg, 52%). Mp 96-98 °C; R_f = 0.2 (10% acetone/hexanes); IR (film) 3289, 1597, 1487, 1420 cm^{-1}; ^{1}H NMR (400 MHz, $CDCl_3$) δ 7.97 (d, J = 2.6 Hz, 1H), 7.74 (d, J = 8.7 Hz, 1H), 7.57 (dd, J = 8.7, 2.6 Hz, 1H) [N*H* not observed]; ^{13}C NMR (150 MHz, $CDCl_3$) ppm 134.8, 130.0 (q, $^2J_{CF}$ = 33.6 Hz), 127.3 (q, $^4J_{CF}$ = 3.4 Hz), 126.3, 125.5 (q, $^4J_{CF}$ = 3.4 Hz), 123.0, 122.9 (q, $^1J_{CF}$ = 273.0 Hz), 119.7 (q, $^1J_{CF}$ = 327.1 Hz); ^{19}F NMR (282 MHz, $CDCl_3$) δ -63.0, -76.5; HRMS (ESI): Exact mass calcd for $C_8H_3ClF_6NO_2S$ $[M-H]^-$ 325.9483, found 325.9485.

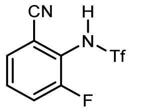

***N*-(2-Cyano-6-fluorophenyl)-1,1,1-trifluoromethanesulfonamide (E1)**. Corresponding aniline (324 mg, 3.6 mmol) was used according to the General Procedure 1, delivering the product as a brown oil (140 mg, 22%). R_f = (10% acetone/hexanes); IR (film) 3107, 1588, 1474, 1434 cm^{-1}; ^{1}H NMR (400 MHz, $CDCl_3$) δ 7.59 (m, 3H), 6.08 (br s, 1H); ^{13}C NMR (150 MHz, $CDCl_3$) ppm 159.0 (d, $^1J_{CF}$ = 254.5 Hz), 131.7 (d, $^3J_{CF}$ = 9.4 Hz), 129.8 (d, $^4J_{CF}$ = 5.2 Hz), 123.7 (d, $^2J_{CF}$ = 16.4 Hz), 122.0 (d, $^2J_{CF}$ = 20.9 Hz), 119.6 (q, $^1J_{CF}$ = 323.1 Hz), 115.6, 114.8 (d, $^4J_{CF}$ = 3.2 Hz); ^{19}F NMR (282 MHz, $CDCl_3$) δ -76.0, -114.5; HRMS (ESI): Exact mass calcd for $C_8H_3F_4N_2O_2S$ $[M-H]^-$ 266.9857, found 266.9858.

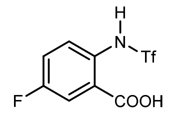

5-Fluoro-2-((trifluoromethyl)sulfonamido)benzoic acid (E2). Corresponding aniline (369 mg, 3.6 mmol) was used according to the General Procedure 1, delivering the product as a white solid (180 mg, 26%). Mp 179-181 °C; R_f = (10% acetone/hexanes); IR (film) 3170, 1678, 1617, 1594, 1498, 1426, 1408 cm^{-1}; ^{1}H NMR (400 MHz, acetone-d_6) δ 11.06 (br s, 1H), 7.88 (dd, J = 8.8, 3.1 Hz, 1H), 7.74 (dd, J = 9.2, 4.7 Hz, 1H), 7.54 (ddd, J = 10.9, 7.4, 3.2 Hz, 1H) [N*H* not observed]; ^{13}C NMR (150 MHz, acetone-d_6) ppm 169.1 (d, $^4J_{CF}$ = 2.0 Hz), 160.3 (d, $^1J_{CF}$ = 247.6 Hz), 134.8 (d, $^4J_{CF}$ = 2.3 Hz), 124.0 (d, $^3J_{CF}$ = 8.2 Hz), 122.8 (d, $^2J_{CF}$ = 22.7 Hz), 121.5 (d, $^3J_{CF}$ = 7.2 Hz), 120.8 (q, $^1J_{CF}$ = 324.1 Hz), 118.8 (d, $^2J_{CF}$ = 24.7 Hz); ^{19}F NMR (282 MHz, $CDCl_3$) δ -77.2, -116.6; HRMS (ESI): Exact mass calcd for $C_8H_4F_4NO_4S$ $[M-H]^-$ 285.9803, found 285.9804.

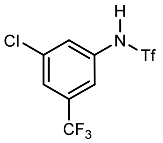

***N*-(3-Chloro-5-(trifluoromethyl)phenyl)-1,1,1-trifluoromethanesulfonamide (E3)**. Corresponding aniline (464 mg, 3.6 mmol) was used according to the General Procedure 1, delivering the product as a yellow liquid (610 mg, 79%). R$_f$ = 0.2 (10% acetone/hexanes); IR (film) 3575, 3290, 3102, 1703, 1612, 1597, 1493, 1432 cm^{-1}; ^{1}H NMR (400 MHz, $CDCl_3$) δ 7.56 (s, 1H), 7.51 (s, 1H), 7.42 (s, 1H) [N*H* not observed]; ^{13}C NMR (150 MHz, $CDCl_3$) ppm 136.5, 133.6 (q, $^2J_{CF}$ = 36.3 Hz), 125.8, 124.2, 123.7, 122.7 (q, $^1J_{CF}$ = 271.3 Hz), 119.8 (q, $^1J_{CF}$ = 323.7 Hz), 117.6; ^{19}F NMR (282 MHz, $CDCl_3$) δ -63.2, -75.8; HRMS (ESI): Exact mass calcd for $C_8H_3ClF_6NO_2S$ [M-H]$^-$ 325.9483, found 325.9484.

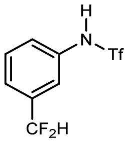

***N*-(3-(Difluoromethyl)phenyl)-1,1,1-trifluoromethanesulfonamide (E4)**. Corresponding aniline (120 mg, 840 µmol) was used according to the General Procedure 1, delivering the product as a light yellow oil (180 mg, 78%). R$_f$ = 0.2 (5% acetone/hexanes); IR (film) 3290, 3091, 1617, 1599, 1477, 1426 cm^{-1}; ^{1}H NMR (400 MHz, acetone-d_6) δ 10.22 (br s, 1H), 7.57 (m, 4H), 6.96 (t, $^2J_{CF}$ = 55.9 Hz, 1H); ^{13}C NMR (150 MHz, $CDCl_3$) ppm 136.2 (t, $^2J_{CF}$ = 22.8 Hz), 134.6, 130.4, 125.5, 124.6 (t, $^3J_{CF}$ = 6.3 Hz), 120.4 (t, $^3J_{CF}$ = 6.3 Hz), 119.9 (q, $^1J_{CF}$ = 322.9 Hz), 113.9 (t, $^1J_{CF}$ = 238.1 Hz); ^{19}F NMR (282 MHz, acetone-d_6) δ -76.7, -121.0; HRMS (ESI): Exact mass calcd for $C_8H_5F_5NO_2S$ [M-H]$^-$ 273.9967, found 273.9967.

1,1,1-Trifluoro-*N*-(4-(4,4,5,5-tetramethyl-1,3,2-dioxaborolan-2-yl)phenyl)methanesulfonamide (E5).

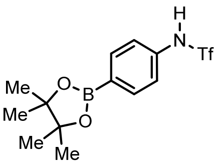

Corresponding aniline (521 mg, 2.4 mmol) was used according to the General Procedure 1, delivering the product as a light brown solid (340 mg, 41%). Mp 119-121 °C; R$_f$ = 0.2 (10% acetone/hexanes); IR (film) 3284, 3138, 2981, 1609, 1489, 1400 cm^{-1}; ^{1}H NMR (400 MHz, $CDCl_3$) δ 7.84 (d, *J* = 8.4 Hz, 2H), 7.24 (d, *J* = 8.3 Hz, 2H), 1.33 (s, 12H) [N*H* not observed]; ^{13}C NMR (150 MHz, $CDCl_3$) ppm 136.6, 136.3, 121.7, 119.8 (q, $^1J_{CF}$ = 323.1 Hz), 84.3, 31.1, 24.9; ^{19}F NMR (282 MHz, $CDCl_3$) δ -75.0; HRMS (ESI): Exact mass calcd for $C_{13}H_{16}BF_3NO_4S$ [M-H]$^-$ 350.0851, found 350.0852.

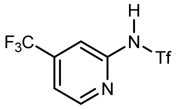

1,1,1-Trifluoro-*N*-(4-(trifluoromethyl)pyridin-2-yl)methanesulfonamide (E6). Corresponding aniline (386 mg, 2.4 mmol) was used according to the General Procedure 1, delivering the product as a light yellow solid (35 mg, 8%). Mp 113-115 °C; R$_f$ = 0.2 (20% acetone/hexanes); IR (film) 3147, 3105, 3083, 1647, 1612, 1525 cm^{-1}; ^{1}H NMR (400 MHz, $CDCl_3$) δ 8.50 (d, *J* = 6.6 Hz, 1H), 7.96 (s, 1H), 7.39 (dd, *J* = 6.5, 1.5 Hz, 1H) [N*H* not observed]; ^{13}C NMR (150 MHz, acetone-d_6) ppm 156.9, 144.8 (q, $^2J_{CF}$ = 33.8 Hz), 141.4, 122.9 (d, $^1J_{CF}$ = 275.1 Hz), 121.4 (q, $^1J_{CF}$ = 323.2 Hz), 115.8, 110.9; ^{19}F NMR (282 MHz, $CDCl_3$) δ -66.8, -79.2; HRMS (ESI): Exact mass calcd for $C_7H_3F_6N_2O_2S$ [M-H]$^-$ 292.9825, found 292.9827.

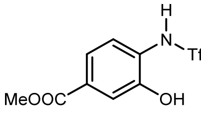

Methyl 3-hydroxy-4-((trifluoromethyl)sulfonamido)benzoate (E7). Corresponding aniline (398 mg, 2.4 mmol) was used according to the General Procedure 1, delivering the product as a brown oil (127 mg, 24%). R$_f$ = 0.1 (20% acetone/hexanes); IR (film) 3214, 2959, 1731, 1612, 1502, 1431 cm^{-1}; ^{1}H NMR (400 MHz, $CDCl_3$) δ 8.10 (d, *J* = 1.9 Hz, 1H), 7.97 (dd, *J* = 8.6, 2.0 Hz, 1H), 7.47 (d, *J* = 8.7 Hz, 1H), 6.65 (s, 1H), 3.96 (s, 3H) [O*H*, N*H* not observed]; ^{13}C NMR (150 MHz, $CDCl_3$) ppm 164.8, 157.7, 143.4, 131.5, 127.9, 123.5 (q, $^1J_{CF}$ = 328.7 Hz), 123.2, 122.9, 53.0; ^{19}F NMR (282 MHz, $CDCl_3$) δ -77.1; HRMS (ESI): Exact mass calcd for $C_9H_7F_3NO_5S$ [M-H]$^-$ 298.0003, found 298.0005.

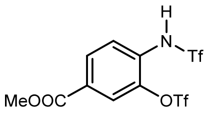

Methyl 4-((trifluoromethyl)sulfonamido)-3-(((trifluoromethyl)sulfonyl)oxy)benzoate (E8). Corresponding aniline (398 mg, 2.4 mmol) was used according to the General Procedure 1, delivering the product as a colorless oil (130 mg, 13%). R$_f$ = 0.2 (10% acetone/hexanes); IR (film) 3339, 2961, 1736, 1605, 1498, 1450 cm^{-1}; ^{1}H NMR (400 MHz, $CDCl_3$) δ 8.35 (dd, *J* = 8.8, 2.1 Hz, 1H), 8.19 (d, *J* = 1.9 Hz, 1H), 7.71 (d, *J* = 8.8 Hz, 1H), 3.98 (s, 3H) [N*H* not observed]; ^{13}C NMR (150 MHz, $CDCl_3$) ppm 163.7, 150.2, 135.5, 134.7, 130.6, 123.7, 120.7, 119.3 (q, $^1J_{CF}$ = 329.3 Hz), 118.4 (q, $^1J_{CF}$ = 322.6 Hz), 53.3; ^{19}F NMR (282 MHz, $CDCl_3$) δ -70.9, -73.7; HRMS (ESI): Exact mass calcd for $C_{10}H_6F_6NO_7S$ [M-H]$^-$ 429.9495, found 429.9501.

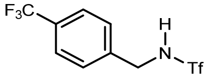

1,1,1-Trifluoro-*N*-(4-(trifluoromethyl)benzyl)methanesulfonamide (F1). Corresponding aniline (455 μL, 3.6 mmol) was used according to the General Procedure 1, delivering the product as a white solid (320 mg, 29%). ^{1}H NMR (400 MHz, $CDCl_3$) δ 7.67 (d, *J* = 8.1 Hz, 2H), 7.44 (d, *J* = 8.0 Hz, 2H), 5.30 (s, 1H), 4.57 (d, *J* = 5.4 Hz, 2H). The characterization data (^{1}H NMR) was in agreement with the literature.[26]

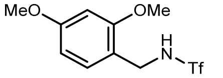

***N*-(2,4-Dimethoxybenzyl)-1,1,1-trifluoromethanesulfonamide (F2)**. Corresponding aniline (605 mg, 3.6 mmol) was used according to the General Procedure 1, delivering the product as a white solid (370 mg, 34%). Mp 75-77 °C; R_f = 0.2 (10% acetone/hexanes); IR (film) 3313, 2944, 1616, 1591, 1510, 1425 cm^{-1}; ^{1}H NMR (400 MHz, $CDCl_3$) δ 7.13 (d, *J* = 8.0 Hz, 1H), 6.39 (m, 2H), 5.53 (s, 1H), 4.36 (d, *J* = 5.7 Hz, 2H), 3.84 (s, 3H), 3.80 (s, 3H); ^{13}C NMR (150 MHz, $CDCl_3$) ppm 161.6, 158.6, 130.6, 119.7 (q, $^{1}J_{CF}$ = 324.6 Hz), 116.4, 104.3, 98.8, 55.5 (2C), 44.7; ^{19}F NMR (282 MHz, $CDCl_3$) δ -77.5; HRMS (ESI): Exact mass calcd for $C_{10}H_{11}F_3NO_4S$ [M-H]$^-$ 298.0366, found 298.0368

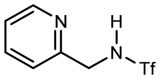

1,1,1-Trifluoro-*N*-(pyridin-2-ylmethyl)methanesulfonamide (F3). Corresponding aniline (339 mg, 3.6 mmol) was used according to the General Procedure 1, delivering the product as a white solid (670 mg, 77%). ^{1}H NMR (400 MHz, $CDCl_3$) δ 8.55 (dd, *J* = 5.4, 1.7 Hz, 1H), 7.74 (ddd, *J* = 7.6, 7.6, 1.5 Hz, 2H), 7.30 (dd, *J* = 7.4, 3.7 Hz, 2H), 4.56 (s, 2H). The characterization data (^{1}H NMR) was in agreement with the literature.[27]

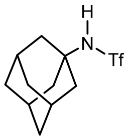

***N*-((1S,3S)-Adamantan-1-yl)-1,1,1-trifluoromethanesulfonamide (F4)**. Corresponding aniline (547 mg, 3.6 mmol) was used according to the General Procedure 1, delivering the product as a white solid (215 mg, 23%). ^{1}H NMR (400 MHz, $CDCl_3$) δ 4.67 (br s, 1H), 2.18-2.06 (m, 3H), 2.00-1.90 (m, 6H), 1.72-1.58 (m, 6H). The characterization data (^{1}H NMR) was in agreement with the literature.[28]

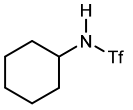

***N*-Cyclohexyl-1,1,1-trifluoromethanesulfonamide (F5)**. Corresponding aniline (413 μg, 3.6 mmol)was used according to the General Procedure 1, delivering the product as a colorless liquid (30 mg, 4%). ^{1}H NMR (400 MHz, $CDCl_3$) δ 4.82 (s, 1H), 3.47 (m, 1H), 2.04 (m, 2H), 1.78 (m, 2H), 1.59 (m, 1H), 1.46-1.27 (m, 4H), 1.25-1.14 (m, 1H). The characterization data (^{1}H NMR) was in agreement with the literature.[29]

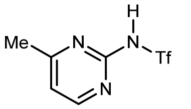

1,1,1-Trifluoro-*N*-(4-methylpyrimidin-2-yl)methanesulfonamide (F6). Corresponding aniline (395 mg, 3.6 mmol)was used according to the General Procedure 1, delivering the product as a light yellow solid (170 mg, 15%). Mp 110-112 °C; R_f = 0.2 (20% acetone/hexanes); IR (film) 3321, 3031, 2959, 1595, 1563, 1476, 1406 cm^{-1}; ^{1}H NMR (400 MHz, acetone-d_6) δ 10.11 (s, 1H), 8.40 (d, *J* = 5.1 Hz, 1H), 7.04 (d, *J* = 5.1 Hz, 1H), 2.40 (s, 3H); ^{13}C NMR (150 MHz, acetone-d_6) ppm 170.5, 160.0, 158.8, 125.6 (q, $^{1}J_{CF}$ = 333.9 Hz), 116.5, 23.8; ^{19}F NMR (282 MHz, acetone-d_6) δ -77.5; HRMS (ESI): Exact mass calcd for $C_6H_5F_3N_3O_2S$ [M-H]$^-$ 240.0060, found 240.0059.

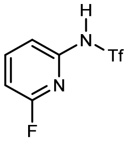

1,1,1-Trifluoro-*N*-(6-fluoropyridin-2-yl)methanesulfonamide (F7). Corresponding aniline (267 mg, 2.4 mmol) was used according to the General Procedure 1, delivering the product as a pale while solid (150 mg, 24%). Mp 99-101 °C; R_f = 0.15 (10% acetone/hexanes); IR (film) 3256, 3063, 2977, 2933, 1619, 1590, 1452, 1428 cm^{-1}; ^{1}H NMR (400 MHz, $CDCl_3$) δ 8.89 (br s, 1H), 7.88 (dd, *J* = 16.0, 8.0 Hz, 1H), 7.41 (dd, *J* = 8.0, 1.5 Hz, 1H), 6.83 (dd, *J* = 8.1, 2.0 Hz, 1H); ^{13}C NMR (150 MHz, acetone-d_6) ppm 163.0 (d, $^{1}J_{CF}$ = 243 Hz), 148.5 (d, $^{3}J_{CF}$ = 15.5 Hz), 145.3 (d, $^{3}J_{CF}$ = 8.0 Hz), 120.7 (q, $^{1}J_{CF}$ = 324.2 Hz), 111.9 (d, $^{4}J_{CF}$ = 4.4 Hz), 107.2 (d, $^{2}J_{CF}$ = 35.8 Hz); ^{19}F NMR (282 MHz, $CDCl_3$) δ -69.2, -77.4; HRMS (ESI): Exact mass calcd for $C_6H_3F_4N_2O_2S$ [M-H]$^-$ 242.9857, found 242.9859.

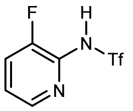

1,1,1-Trifluoro-*N*-(3-fluoropyridin-2-yl)methanesulfonamide (F8). Corresponding aniline (267 mg, 2.4 mmol) was used according to the General Procedure 1, delivering the product as a pale pink solid (160 mg, 25%). Mp 105-108 °C; R_f = 0.15 (10% acetone/hexanes); IR (film) 3242, 1645, 1598,

1560, 1451 cm^{-1}; ^{1}H NMR (400 MHz, $CDCl_3$) δ 11.4-10.3 (br s, 1H), 7.75 (d, J = 11.0 Hz, 1H), 7.69 (ddd, J = 8.7, 8.7, 1.7 Hz, 1H), 6.99 (ddd, J = 10.6, 8.4, 1.7 Hz, 1H); ^{13}C NMR (150 MHz, acetone-d_6) ppm 152.0 (d, $^{1}J_{CF}$ = 255.4 Hz), 148.0 (d, $^{2}J_{CF}$ = 20.2 Hz), 133.8, 129.0 (d, $^{2}J_{CF}$ = 19.2 Hz), 121.1 (q, $^{1}J_{CF}$ = 321.7 Hz), 115.6 (d, $^{3}J_{CF}$ = 4.9 Hz); ^{19}F NMR (282 MHz, $CDCl_3$) δ -78.8, -123.2; HRMS (ESI): Exact mass calcd for $C_6H_3F_4N_2O_2S$ [M-H]$^-$ 242.9857, found 242.9859.

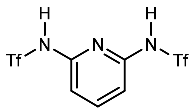

***N*,*N*'-(Pyridine-2,6-diyl)bis(1,1,1-trifluoromethanesulfonamide) (G1)**. Corresponding aniline (394 mg, 3.6 mmol) was used according to the General Procedure 1, delivering the product as a brown solid (121 mg, 9%). Mp 203-205 °C; R_f = 0.2 (40% acetone/hexanes); IR (film) 3252, 3109, 1603, 1539 cm^{-1}; ^{1}H NMR (400 MHz, acetone-d_6) δ 7.98 (t, J = 7.6 Hz, 1H), 7.2 (d, J = 8.3 Hz, 2H) ; ^{13}C NMR (150 MHz, acetone-d_6) ppm 154.7, 145.7, 122.3 (q, $^{1}J_{CF}$ = 324.1 Hz), 105.9; ^{19}F NMR (282 MHz, acetone-d_6) [N*H* not observed]; δ -78.9; HRMS (ESI): Exact mass calcd for $C_7H_4F_6N_3O_4S_2$ [M-H]$^-$ 371.9553, found 371.9553.

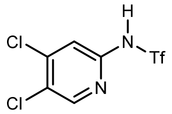

***N*-(4,5-Dichloropyridin-2-yl)-1,1,1-trifluoromethanesulfonamide (G2)**. Corresponding aniline (388 mg, 2.4 mmol) was used according to the General Procedure 1, delivering the product as a white solid (40 mg, 6%). Mp 189-191 °C; R_f = 0.2 (20% acetone/hexanes); IR (film) 3120, 3037, 2926, 1635, 1603, 1497, 1444 cm^{-1}; ^{1}H NMR (400 MHz, acetone-d_6) δ 8.52 (s, 1H), 7.89 (s, 1H) [N*H* not observed]; ^{13}C NMR (150 MHz, acetone-d_6) ppm 155.1, 150.2, 139.6, 122.4, 121.2 (q, $^{1}J_{CF}$ = 322.3 Hz), 118.9; ^{19}F NMR (282 MHz, $CDCl_3$) δ -77.5; HRMS (ESI): Exact mass calcd for $C_6H_2Cl_2F_3N_2O_2S$ [M-H]$^-$ 292.9172, found 292.9177.

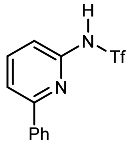

1,1,1-Trifluoro-*N*-(6-phenylpyridin-2-yl)methanesulfonamide (G3). Corresponding aniline (405 mg, 2.4 mmol) was used according to the General Procedure 1, delivering the product as a white solid (380 mg, 53%). Mp 125-128 °C; R_f = 0.2 (30% acetone/hexanes); IR (film) 3249, 3174, 1610, 1578, 1532, 1497, 1415 cm^{-1}; ^{1}H NMR (400 MHz, $CDCl_3$) δ 7.91 (dd, J = 8.7, 7.6 Hz, 1H), 7.67 (m, 2H), 7.56 (m, 3H), 7.17 (t, J = 7.0 Hz, 2H); ^{13}C NMR (150 MHz, acetone-d_6) ppm 156.7, 148.9, 144.8, 132.2, 131.2, 129.3, 127.6, 120.9 (q, $^{1}J_{CF}$ = 323.3 Hz), 116.1, 113.4; ^{19}F NMR (282 MHz, $CDCl_3$) δ -78.6; HRMS (ESI): Exact mass calcd for $C_{12}H_8F_3N_2O_2S$ [M-H]$^-$ 301.0264, found 301.0266.

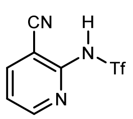

***N*-(3-Cyanopyridin-2-yl)-1,1,1-trifluoromethanesulfonamide (G4)**. Corresponding aniline (283 mg, 2.4 mmol) was used according to the General Procedure 1, delivering the product as an orange solid (120 mg, 20%). Mp 102-104 °C; R_f = 0.3 (20% acetone/hexanes); IR (film) 3240, 3176, 3115, 2240, 1619, 1596, 1451, 1451 cm^{-1}; ^{1}H NMR (400 MHz, acetone-d_6) δ 8.67 (d, J = 5.0 Hz, 1H), 8.49 (d, J = 4.2 Hz, 1H), 7.36 (dd, J = 4.7, 4.7 Hz, 1H) [N*H* not observed]; ^{13}C NMR (150 MHz, acetone-d_6) ppm 155.7, 151.7, 142.4, 121.2 (q, $^{1}J_{CF}$ = 322.8 Hz), 115.6, 114.5, 107.5; ^{19}F NMR (282 MHz, acetone-d_6) δ -79.9; HRMS (ESI): Exact mass calcd for $C_7H_3F_3N_3O_2S$ [M-H]$^-$ 249.9904, found 249.9905.

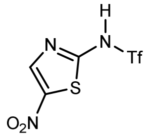

1,1,1-Trifluoro-*N*-(5-nitrothiazol-2-yl)methanesulfonamide (G5). Corresponding aniline (525 mg, 3.6 mmol) was used according to the General Procedure 1, delivering the product as a yellow solid (170 mg, 17%). Mp 85-87 °C; R_f = 0.2 (20% acetone/hexanes); IR (film) 3102, 1591, 1502, 1460, 1446 cm^{-1}; ^{1}H NMR (400 MHz, $CDCl_3$) δ 8.93 (s, 1H) [N*H* not observed]; ^{13}C NMR (150 MHz, acetone-d_6) ppm 169.4, 136.8, 134.4, 120.7 (q, $^{1}J_{CF}$ = 317.4 Hz); ^{19}F NMR (282 MHz, $CDCl_3$) δ -77.9; HRMS (ESI): Exact mass calcd for $C_4HF_3N_3O_4S_2$ [M-H]$^-$ 275.9366, found 275.9368.

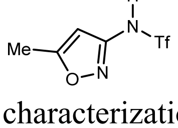

1,1,1-Trifluoro-*N*-(5-methylisoxazol-3-yl)methanesulfonamide (G6). Corresponding aniline (234 mg, 2.4 mmol) was used according to the General Procedure 1, delivering the product as a white solid (66 mg, 13%). ^{1}H NMR (400 MHz, $CDCl_3$) δ 6.32 (s, 1H) [N*H* not observed]. The characterization data (^{1}H NMR) was in agreement with the literature.[30]

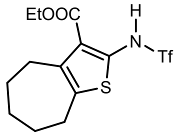

Ethyl 2-((trifluoromethyl)sulfonamido)-5,6,7,8-tetrahydro-4H-cyclohepta[b]thiophene-3-carboxylate (G7). Corresponding aniline (597 mg, 2.4 mmol) was used according to the General Procedure 1, delivering the product as a red oil (360 mg, 41%). R_f = 0.2 (10% acetone/hexanes); IR (film) 3434, 2926, 2925, 1721, 1661, 1553, 1491, 1416 cm^{-1}; ^{1}H NMR (400 MHz, $CDCl_3$) δ 10.77 (s, 1H), 4.36 (q, J = 7.1 Hz, 2H), 3.00 (t, J = 5.5 Hz, 2H), 2.74 (t, J = 5.6 Hz, 2H), 1.84 (m, 2H), 1.69 (m, 2H), 1.62 (m, 2H), 1.38 (t, J = 7.1 Hz, 3H); ^{13}C NMR (150 MHz, $CDCl_3$) ppm 166.0, 141.7, 137.7, 132.7, 119.3 (q, $^1J_{CF}$ = 320.9 Hz), 117.5, 61.5, 31.8, 28.8, 28.3, 27.4, 26.6, 13.9; ^{19}F NMR (282 MHz, $CDCl_3$) δ -75.3; HRMS (ESI): Exact mass calcd for $C_{13}H_{15}F_3NO_4S_2$ [M-H]$^-$ 370.0400, found 370.0402.

1,1,1-Trifluoro-*N*-(4-phenylthiazol-2-yl)methanesulfonamide (G8). Corresponding aniline (419 mg, 2.4 mmol) was used according to the General Procedure 1, delivering the product as a white solid (50 mg, 7%). Mp

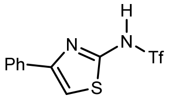

172-174 °C; R_f = 0.2 (30% acetone/hexanes); IR (film) 3236, 1685, 1595, 1570, 1532 cm^{-1}; ^{1}H NMR (400 MHz, $CDCl_3$) δ 7.51 (m, 5H), 6.76 (s, 1H) [N*H* not observed]; ^{13}C NMR (150 MHz, acetone-*d*6) ppm 173.6, 138.4, 130.6, 130.1, 129.3, 126.9, 121.3 (q, $^1J_{CF}$ = 321.6 Hz), 106.4; ^{19}F NMR (282 MHz, $CDCl_3$) δ -78.0; HRMS (ESI): Exact mass calcd for $C_{10}H_6F_3N_2O_2S_2$ [M-H]$^-$ 306.9828, found 306.9829.

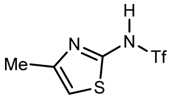

1,1,1-Trifluoro-*N*-(4-methylthiazol-2-yl)methanesulfonamide (H1). Corresponding aniline (272 mg, 2.4 mmol) was used according to the General Procedure 1, delivering the product as a light yellow solid (210 mg, 36%). Mp 178-180 °C; R_f = 0.2 (30% acetone/hexanes); IR (film) 3169, 3106, 3007, 2908, 1614, 1578, 1471, 1449 cm^{-1}; ^{1}H NMR (400 MHz, $CDCl_3$) δ 6.29 (d, J = 1.1 Hz, 1H), 2.37 (d, J = 1.0 Hz, 3H) [N*H* not observed]; ^{13}C NMR (150 MHz, acetone-*d*6) ppm 173.1, 135.5, 121.5 (q, $^1J_{CF}$ = 322.6 Hz), 105.9, 13.5; ^{19}F NMR (282 MHz, $CDCl_3$) δ -77.9; HRMS (ESI): Exact mass calcd for $C_5H_4F_3N_2O_2S_2$ [M-H]$^-$ 244.9672, found 244.9673.

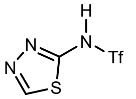

1,1,1-Trifluoro-*N*-(1,3,4-thiadiazol-2-yl)methanesulfonamide (H2). Corresponding aniline (259 mg, 2.4 mmol) was used according to the General Procedure 1, delivering the product as a light yellow solid (40 mg, 7%). Mp 131-133 °C; R_f = 0.2 (30% acetone/hexanes); IR (film) 3122, 3021, 2875, 1543, 1507, cm^{-1}; ^{1}H NMR (400 MHz, acetone-*d*6) δ 9.01 (s, 1H) [N*H* not observed]; ^{13}C NMR (150 MHz, acetone-*d*6) ppm 173.0, 147.8, 121.1 (q, $^1J_{CF}$ = 324.1 Hz); ^{19}F NMR (282 MHz, acetone-*d*6) δ -79.6; HRMS (ESI): Exact mass calcd for $C_3HF_3N_3O_2S_2$ [M-H]$^-$ 231.9468, found 231.9467.

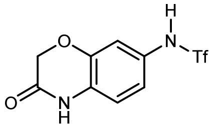

1,1,1-Trifluoro-*N*-(3-oxo-3,4-dihydro-2H-benzo[b][1,4]oxazin-7-yl)methanesulfonamide (H3). Corresponding aniline (390 mg, 2.4 mmol) was used according to the General Procedure 1, delivering the product as a brown solid (125 mg, 15%). Mp 162-164 °C; R_f = 0.2 (30% acetone/hexanes); IR (film) 3328, 3089, 1691, 1516, 1438, 1415 cm^{-1}; ^{1}H NMR (400 MHz, acetone-*d*6) δ 10.14 (s, 1H), 9.80 (s, 1H), 7.03 (d, J = 5.4 Hz, 1H), 6.99 (m, 2H), 4.63 (s, 2H); ^{13}C NMR (150 MHz, $CDCl_3$) ppm 165.0, 144.9, 130.5, 127.7, 121.2 (q, $^1J_{CF}$ = 321.0 Hz), 119.1, 117.1, 113.3, 67.9; ^{19}F NMR (282 MHz, $CDCl_3$) δ -76.5; HRMS (ESI): Exact mass calcd for $C_9H_6F_3N_2O_4S$ [M-H]$^-$ 295.0006, found 295.0005.

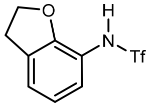

***N*-(2,3-Dihydrobenzofuran-7-yl)-1,1,1-trifluoromethanesulfonamide (H4)**. Corresponding aniline (321 mg, 2.4 mmol) was used according to the General Procedure 1, delivering the product as a brown oil (530 mg, 84%). R_f = 0.2 (10% acetone/hexanes); IR (film) 3276, 2982, 1601, 1484, 1470, 1427 cm^{-1}; ^{1}H NMR (400 MHz, $CDCl_3$) δ 7.22 (d, J = 8.1 Hz, 1H), 7.07 (dd, J = 7.4, 0.9 Hz, 1H), 6.80 (dd, J = 7.7. 7.7 Hz, 1H), 4.62 (t, J = 8.7 Hz, 2H), 3.25 (t, J = 8.7 Hz, 2H) [N*H* not observed]; ^{13}C NMR (150 MHz, $CDCl_3$) ppm 152.5, 128.4, 123.8, 122.4, 121.3, 119.9 (q, $^1J_{CF}$ = 324.2 Hz), 117.4, 72.5, 30.2; ^{19}F NMR (282 MHz, $CDCl_3$) δ -76.0; HRMS (ESI): Exact mass calcd for $C_9H_7F_3NO_3S$ [M-H]$^-$ 266.0104, found 266.0105.

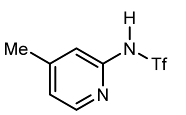

1,1,1-Trifluoro-*N*-(4-methylpyridin-2-yl)methanesulfonamide (H5). Corresponding aniline (391 mg, 3.6 mmol) was used according to the General Procedure 1, delivering the product as a white solid (150 mg, 17%). Mp 112-115 °C; R_f = 0.2 (20% acetone/hexanes); IR (film) 3468, 3228, 3133, 1638, 1606, 1518, 1444, 1421 cm^{-1}; ^1H NMR (400 MHz, $CDCl_3$) δ 8.46 (d, *J* = 5.0 Hz, 1H), 7.38 (d, *J* = 4.4 Hz, 1H), 7.25 (s, 1H), 2.46 (s, 3H) [N*H* not observed]; ^{13}C NMR (150 MHz, acetone-d_6) ppm 154.1, 150.8, 146.7, 129.3, 127.2, 120.1 (q, $^1J_{CF}$ = 331.7 Hz), 20.6; ^{19}F NMR (282 MHz, $CDCl_3$) δ -70.6; HRMS (ESI): Exact mass calcd for $C_7H_6F_3N_2O_2S$ $[M-H]^-$ 239.0108, found 239.0107.

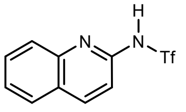

1,1,1-Trifluoro-*N*-(quinolin-2-yl)methanesulfonamide (H6). Corresponding aniline (521 mg, 3.6 mmol) was used according to the General Procedure 1, delivering the product as a red wax (430 mg, 43%). R_f = (10% acetone/hexanes); IR (film) 3270, 3060, 1693, 1632, 1602, 1510, 1467, 1449, 1426 cm^{-1}; ^1H NMR (400 MHz, $CDCl_3$) δ 7.86 (m, 3H), 7.77 (d, *J* = 2.0 Hz, 1H), 7.54 (m, 2H), 7.37 (dd, *J* = 8.8, 2.2 Hz, 1H); ^{13}C NMR (150 MHz, acetone-d_6) ppm 134.4, 133.5, 132.8, 130.4, 128.6, 127.9, 127.2, 122.9, 121.8, 121.1 (q, $^1J_{CF}$ = 323.1 Hz); ^{19}F NMR (282 MHz, $CDCl_3$) δ -76.5; HRMS (ESI): Exact mass calcd for $C_{10}H_{11}F_3N_3O_2S$ $[M+NH_4]^-$ 294.0518, found 294.0523.

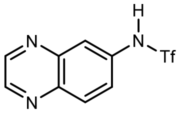

1,1,1-Trifluoro-*N*-(quinoxalin-6-yl)methanesulfonamide (H7). Corresponding aniline (345 mg, 2.4 mmol) was used according to the General Procedure 1, delivering the product as a brown solid (45 mg, 7%). Mp 181-184 °C; R_f = 0.3 (30% acetone/hexanes); IR (film) 3350, 3063, 1695, 1622, 1580, 1500, 1436 cm^{-1}; ^1H NMR (400 MHz, acetone-d_6) δ 8.95 (d, *J* = 1.8 Hz, 1H), 8.92 (d, *J* = 1.8 Hz, 1H), 8.18 (d, *J* = 9.0 Hz, 1H), 8.06 (d, *J* = 2.5 Hz, 1H), 7.90 (dd, *J* = 9.0, 2.5 Hz, 1H) [N*H* not observed]; ^{13}C NMR (150 MHz, acetone-d_6) ppm 147.5, 146.6, 143.9, 142.0, 137.1, 132.0, 125.6, 121.1, 120.9 (q, $^1J_{CF}$ = 321.1 Hz); ^{19}F NMR (282 MHz, acetone-d_6) δ -75.2; HRMS (ESI): Exact mass calcd for $C_9H_5F_3N_3O_2S$ $[M-H]^-$ 276.0060, found 276.0061.

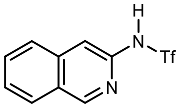

1,1,1-Trifluoro-*N*-(isoquinolin-3-yl)methanesulfonamide (H8). Corresponding aniline (343 mg, 2.4 mmol) was used according to the General Procedure 1, delivering the product as a yellow solid (138 mg, 16%). Mp 168-170 °C; R_f = 0.2 (20% acetone/hexanes); IR (film) 3421, 2923, 1645, 1617 cm^{-1}; ^1H NMR (400 MHz, acetone-d_6) δ 9.32 (s, 1H), 8.22 (d, *J* = 8.4 Hz, 1H), 8.00 (d, *J* = 8.6 Hz, 1H), 7.97 (s, 1H), 7.88 (ddd, *J* = 8.1, 6.8, 1.1 Hz, 1H), 7.59 (ddd, *J* = 8.3, 6.9, 0.9 Hz, 1H) [N*H* not observed]; ^{13}C NMR (150 MHz, acetone-d_6) ppm 150.1, 145.2, 142.6, 135.7, 130.1, 127.6, 127.1, 123.7, 122.3 (q, $^1J_{CF}$ = 323.2 Hz), 112.0; ^{19}F NMR (282 MHz, acetone-d_6) δ -78.2; HRMS (ESI): Exact mass calcd for $C_{10}H_6F_3N_2O_2S$ $[M-H]^-$ 275.0108, found 275.0109.

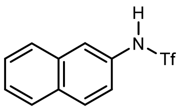

1,1,1-Trifluoro-*N*-(naphthalen-2-yl)methanesulfonamide (I1). Corresponding aniline (518 mg, 3.6 mmol) was used according to the General Procedure 1, delivering the product as a white solid (146 mg, 13%). ^1H NMR (400 MHz, $CDCl_3$) δ 7.70–7.61 (m, 3H), 7.58 (d, *J* = 2.0 Hz, 1H), 7.40-7.36 (m, 2H), 7.20 (dd, *J* = 8.8, 2.4 Hz, 1H), 6.88 (s, 1H). The characterization data (^1H NMR) was in agreement with the literature.[16]

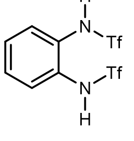

***N*,*N'*-(1,2-Phenylene)bis(1,1,1-trifluoromethanesulfonamide) (I2)**. Corresponding aniline (270 mg, 2.5 mmol) was used according to the General Procedure 1, delivering the product as a red solid (110 mg, 12%). ^1H NMR (400 MHz, $CDCl_3$) δ 7.20 (br s, 2H), 7.43–7.53 (m, 4H). The characterization data (^1H NMR) was in agreement with the literature.[31]

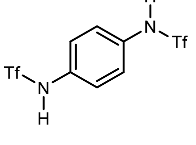

***N*,*N'*-(1,4-Phenylene)bis(1,1,1-trifluoromethanesulfonamide) (I3)**. Corresponding aniline (288 mg, 2.7 mmol) was used according to the General Procedure 1, delivering the product as a brown solid (308 mg, 31%). Mp 194-196 °C; IR (film) 3248, 2900, 1696, 1509, 1474, 1414 cm^{-1}; R_f = 0.6 (30 % EtOAc/hexanes); ^1H NMR (400 MHz, acetone-d_6) δ 10.20 (br s, 1H), 7.55-7.43 (s, 2H); ^{13}C NMR (150 MHz, acetone-d_6) ppm 134.5, 125.1, 120.9 (q, $^1J_{CF}$ = 324.8 Hz); ^{19}F

NMR (282 MHz, CDCl3) δ -76.7; HRMS (ESI): Exact mass calcd for $C_8H_6F_9N_2O_4S_2$ $[M]^+$ 371.9668, found 371.9667.

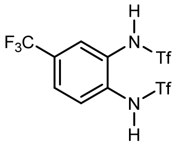

***N,N'*-(4-(Trifluoromethyl)-1,2-phenylene)bis(1,1,1-trifluoromethanesulfonamide) (I4)**. Corresponding aniline (300 mg, 1.7 mmol) was used according to the General Procedure 1, delivering the product as a pink wax (425 mg, 57%). IR (film) 3288, 1621, 1521, 1456 cm^{-1}; R*f* = 0.2 (30 % EtOAc/hexanes); ^{1}H NMR (400 MHz, acetone-*d*6) δ 10.33 (br s, 2H), 7.93 (dd, *J* = 8.7, 1.6 Hz, 1H), 7.88-7.84 (m, 2H); ^{13}C NMR (150 MHz, acetone-*d*6) ppm 135.7, 131.4, 130.9 (q, $^2J_{CF}$ = 33.5 Hz), 128.8, 127.4, 126.1, 124.2 (q, $^1J_{CF}$ = 271.5 Hz), 120.8 (q, $^1J_{CF}$ = 321.8), 120.7 (q, $^1J_{CF}$ = 321.8); ^{19}F NMR (282 MHz, acetone-*d*6) ppm -63.5, -76.9, -77.1; HRMS (ESI): Exact mass calcd for $C_9H_4F_9N_2O_4S_2$ $[M-H]^-$ 438.9474, found 438.9474.

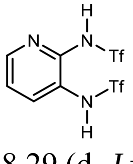

***N,N'*-(Pyridine-2,3-diyl)bis(1,1,1-trifluoromethanesulfonamide) (I6)**. Corresponding aniline (260 mg, 2.4 mmol) was used according to the General Procedure 1, delivering the product as a light brown solid (112 mg, 36%). Mp 210-214 °C; R*f* = 0.2 (40% acetone/hexanes); IR (film) 3505, 3241, 3185, 3129, 1701, 1632, 1604, 1551, 1423, 1402 cm^{-1}; ^{1}H NMR (400 MHz, acetone-*d*6) δ 10.30 (br s, 2H), 8.29 (d, *J* = 7.9 Hz, 1H), 8.22 (d, *J* = 6.2 Hz, 1H), 7.28 (t, *J* = 7.1 Hz, 1H); ^{13}C NMR (150 MHz, acetone-*d*6) ppm 149.9, 137.6, 135.0, 126.5, 120.3 (q, $^1J_{CF}$ = 324.1 Hz), 119.1 (q, $^1J_{CF}$ = 323.1 Hz), 115.6; ^{19}F NMR (282 MHz, $CDCl_3$) δ -77.6, -80.1; HRMS (ESI): Exact mass calcd for $C_7H_4F_6N_3O_4S_2$ $[M-H]^-$ 371.9553, found 371.9554.

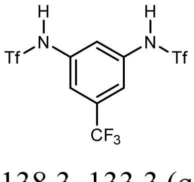

***N,N'*-(5-(Trifluoromethyl)-1,3-phenylene)bis(1,1,1-trifluoromethanesulfonamide) (I8)**. Corresponding aniline (1.0 g, 5.7 mmol) was used according to the General Procedure 1, delivering the product as a yellow solid (1.3 g, 53%). Mp 133-135 °C; IR (film) 3274, 3057, 1619, 1426 cm^{-1}; R*f* = 0.25 (30 % EtOAc/hexanes); ^{1}H NMR (400 MHz, acetone-*d*6) δ 10.75 (br s, 2H), 7.76 (t, *J* = 1.8 Hz, 1H), 7.63 (d, *J* = 1.3 Hz, 2H); ^{13}C NMR (100 MHz, acetone-*d*6) ppm 138.3, 133.3 (q, $^2J_{CF}$ = 33.0 Hz), 122.6, 120.8 (q, $^1J_{CF}$ = 320.1 Hz), 119.3, 117.1 (q, $^3J_{CF}$ = 3.7 Hz); ^{19}F NMR (282 MHz, acetone-*d*6) ppm -63.8, -77.0; HRMS (ESI): Exact mass calcd for $C_9H_5F_9N_2O_4S_2$ $[M]^+$ 439.9542, found 439.9548.

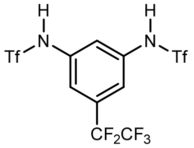

***N,N'*-(5-(Perfluoroethyl)-1,3-phenylene)bis(1,1,1-trifluoromethanesulfonamide) (J1)**. Corresponding aniline (64 mg, 284 μmol) was used according to the General Procedure 1, delivering the product as a yellow solid (85 mg, 61%). Mp 141-143 °C; IR (film) 3585, 3284, 2927, 1697, 1614, 1488, 1425 cm^{-1}; R*f* = 0.25 (30 % EtOAc/hexanes); ^{1}H NMR (600 MHz, acetone-*d*6) δ 10.75 (br s, 2H), 7.76 (t, *J* = 1.8 Hz, 1H), 7.63 (d, *J* = 1.3 Hz, 2H); ^{13}C NMR (150 MHz, acetone-*d*6) ppm 138.6, 131.4 (t, *J* = 25.1 Hz), 120.7 (q, $^1J_{CF}$ = 320.8 Hz), 119.6, 119.4 (qt, $^1J_{CF}$ = 282.1, $^2J_{CF}$ = 43.6 Hz), 118.1, 113.6 (tq, $^1J_{CF}$ = 254.5, $^2J_{CF}$ = 35.8 Hz); ^{19}F NMR (282 MHz, acetone-*d*6) ppm -76.9; HRMS (ESI): Exact mass calcd for $C_{10}H_4F_{11}N_2O_4S_2$ $[M-H]^-$ 488.9442, found 488.9441.

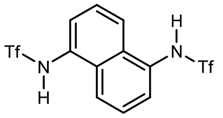

***N,N'*-(Naphthalene-1,5-diyl)bis(1,1,1-trifluoromethanesulfonamide) (J2)**. Corresponding aniline (300 mg, 1.9 mmol) was used according to the General Procedure 1, delivering the product as a light red solid (80 mg, 10%). Mp > 250 °C; IR (film) 3283, 1602 cm^{-1}; R*f* = 0.4 (30% EtOAc/hexanes); ^{1}H NMR (600 MHz, acetone-*d*6) δ 10.52 (br s, 2H), 8.34 (dd, *J* = 3.1, 3.1 Hz, 2H), 7.80 (d, *J* = 3.1 Hz, 4H); ^{13}C NMR (150 MHz, acetone-*d*6) ppm 132.3, 131.3, 127.8, 127.6, 124.3, 121.1 (q, $^1J_{CF}$ = 319.4 Hz); ^{19}F NMR (282 MHz, acetone-*d*6) ppm -76.9; HRMS (ESI): Exact mass calcd for $C_{12}H_8F_6N_2O_4S_2$ $[M]^+$ 421.9824, found 421.9811.

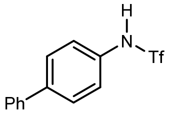

***N*-([1,1'-Biphenyl]-4-yl)-1,1,1-trifluoromethanesulfonamide (J3)**. Corresponding aniline (200 mg, 1.2 mmol) was used according to the General Procedure 1, delivering the product as a colorless liquid (112 mg 31%). ^{1}H NMR (400 MHz, $CDCl_3$) δ 7.61 (d, *J* = 8.6 Hz, 2H), 7.55 (d, *J* = 7.1 Hz,

2H), 7.45 (dd, J = 8.6, 7.2 Hz, 2H), 7.36 (m, 3H), 6.75 (br s, 1H). A The characterization data (^{1}H NMR) was in agreement with the literature.[16]

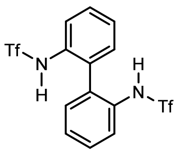

***N,N'*-([1,1'-Biphenyl]-2,2'-diyl)bis(1,1,1-trifluoromethanesulfonamide) (J4)**. Corresponding aniline (110 mg, 595 μmol) was used according to the General Procedure 1, delivering the product as a colorless liquid (170 mg, 68%). Mp 128-130 °C; R$_f$ = 0.2 (20% acetone/hexanes); IR (film) 3434, 1641 cm^{-1}; ^{1}H NMR (400 MHz, $CDCl_3$) δ 7.65 (d, J = 8.0 Hz, 1H), 7.53 (ddd, J = 7.6, 7.6, 1.5 Hz, 1H), 7.45 (ddd, J = 7.6, 7.6, 1.3 Hz, 1H), 7.30 (dd, J = 7.6, 1.5 Hz, 1H), 6.45 (s, 1H); ^{13}C NMR (150 MHz, acetone-d_6) ppm 137.0, 134.7, 133.7, 133.3, 132.4, 131.8, 121.1 (q, $^{1}J_{CF}$ = 323.4 Hz); ^{19}F NMR (282 MHz, $CDCl_3$) ppm -76.0; HRMS (ESI): Exact mass calcd for $C_{14}H_9F_6N_2O_4S_2$ [M-H]$^-$ 446.9913, found 446.9904.

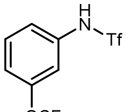

1,1,1-Trifluoro-*N*-(3-(trifluoromethoxy)phenyl)methanesulfonamide (J5). Corresponding aniline (209 μL, 1.5 mmol) was used according to the General Procedure 1, delivering the product as a colorless liquid (145 mg, 31%). R$_f$ = 0.3 (10% acetone/hexanes); IR (film) 3584, 3278, 2961, 1679, 1592, 1460, 1431 cm^{-1}; ^{1}H NMR (600 MHz, acetone-d_6) δ 9.88 (s, 1H), 7.65 (d, J = 5.4 Hz, 1H), 7.42 (dd, J = 5.4, 5.4 Hz, 1H), 7.27 (dd, J = 5.3, 5.3 Hz, 1H), 7.02 (d, J = 5.5 Hz, 1H); ^{13}C NMR (150 MHz, acetone-d_6) ppm 150.7, 129.7, 127.7, 126.5, 125.5, 120.6 (2C, q, $^{1}J_{CF}$ = 327.0 Hz), 119.4; ^{19}F NMR (282 MHz, acetone-d_6) δ -58.9, -77.3; HRMS (ESI): Exact mass calcd for $C_8H_4F_6NO_3S$ [M-H]$^-$ 307.9822, found 307.9824.

***N,N'*-(Oxybis(3,1-phenylene))bis(1,1,1-trifluoromethanesulfonamide) (J6)**. Corresponding aniline (125 mg, 622 μmol) was used according to the General Procedure 1, delivering the product as a brown solid (70 mg, 24%). Mp 136-138 °C; R$_f$ = 0.2 (10% acetone/hexanes); IR (film) 3289, 1597, 1487, 1420 cm^{-1}; ^{1}H NMR (600 MHz, acetone-d_6) δ 10.29 (br s, 1H), 7.49 (dd, J = 5.5, 5.5 Hz, 1H), 7.21 (dd, J = 5.0, 5.0 Hz, 1H), 7.08 (d, J = 1.1 Hz, 1H), 6.99 (d, J = 5.5 Hz, 1H); ^{13}C NMR (150 MHz, acetone-d_6) ppm 158.3, 137.5, 131.9, 120.8 (q, $^{1}J_{CF}$ = 323.0 Hz), 118.6, 117.8, 113.9; ^{19}F NMR (282 MHz, acetone-d_6) δ -77.3; HRMS (ESI): Exact mass calcd for $C_{14}H_9F_6N_2O_5S_2$ [M-H]$^-$ 462.9863, found 462.9863.

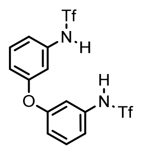

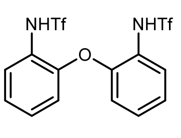

***N,N'*-(Oxybis(2,1-phenylene))bis(1,1,1-trifluoromethanesulfonamide) (J7)**. Corresponding aniline (300 mg, 1.5 mmol) was used according to the General Procedure 1, delivering the product as an off-white solid (553 mg, 80 %). Mp 118-120 °C; IR (film) 3281, 1596, 1498 cm^{-1}; R$_f$ = 0.3 (30 % EtOAc/hexanes); ^{1}H NMR (400 MHz, $CDCl_3$) δ 7.62 (dd, J = 7.9, 1.7 Hz, 1H), 7.50 (br s, 1H), 7.25 (ddd, J = 7.8, 7.8, 1.8 Hz, 1H), 7.21 (ddd, J = 7.7, 7.7, 1.6 Hz, 1H), 6.90 (dd, J = 8.1, 1.5 Hz, 1H); ^{13}C NMR (100 MHz, $CDCl_3$) ppm 148.0, 128.6, 125.5, 125.4, 125.1, 121.4, 118.2; ^{19}F NMR (282 MHz, $CDCl_3$) ppm -75.8; HRMS (ESI): Exact mass calcd for $C_{14}H_{10}F_6N_2O_5S_2$ [M]$^+$ 463.9930, found 463.9916.

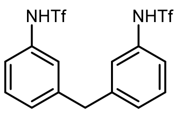

***N,N'*-(Methylenebis(3,1-phenylene))bis(1,1,1-trifluoromethanesulfonamide) (J8)**. Corresponding aniline (130 mg, 650 μmol) was used according to the General Procedure 1, delivering the product as a white solid (121 mg, 40%). Mp 144-146 °C; IR (film) 3266, 1596, 1417 cm^{-1}; R$_f$ = 0.6 (30 % EtOAc/hexanes); ^{1}H NMR (400 MHz, acetone-d_6) δ 10.25 (br s, 2H), 7.37 (m, 2H), 7.26 (br s, 2H), 7.25-7.23 (m, 2H), 7.21 (br d, J = 7.5 Hz, 2H), 4.10 (s, 2H); ^{13}C NMR (100 MHz, acetone-d_6) ppm 143.3, 136.0, 130.5, 128.3, 124.4, 121.8, 120.9 (q, $^{1}J_{CF}$ = 323.5 Hz), 41.6; ^{19}F NMR (282 MHz, acetone-d_6) ppm -76.7; HRMS (ESI): Exact mass calcd for $C_{15}H_{12}F_6N_2O_4S_2$ [M]$^+$ 462.0137, found 462.0128.

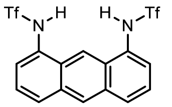

***N,N'*-(Anthracene-1,8-diyl)bis(1,1,1-trifluoromethanesulfonamide) (K1)**. Corresponding aniline (100 mg, 480 μmol) was used according to the General Procedure 1, delivering the product as a white solid (55 mg, 40%). Mp 171-173 °C; IR (film) 3266, 2923, 1692, 1492, 1421cm^{-1}; R$_f$ = 0.1 (40 % EtOAc/hexanes); ^{1}H NMR (600 MHz, acetone-d_6) δ 9.29 (s, 1H), 8.78 (s, 1H), 8.19 (d, J = 5.7 Hz, 2H), 7.73 (d, J = 4.7 Hz, 2H), 7.61 (dd, J = 5.3, 5.3 Hz, 2H) [N*H* not observed]; ^{13}C NMR (150 MHz, acetone-d_6) ppm 133.5, 131.8, 130.4, 129.9, 128.9, 126.7, 126.4, 121.2 (q, $^{1}J_{CF}$ = 323.8 Hz), 118.2; ^{19}F NMR (282

MHz, acetone-d_6) ppm -76.8; HRMS (ESI): Exact mass calcd for $C_{16}H_9F_6N_2O_4S_2$ $[M-H]^-$ 470.9913, found 470.9905.

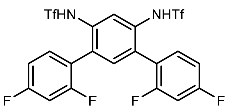

***N*,*N*'-(2,2'',4,4''-Tetrafluoro-[1,1':3',1''-terphenyl]-4',6'-diyl)bis(1,1,1-trifluoromethanesulfonamide) (K2)**. Corresponding aniline (85 mg, 330 μmol) was used according to the General Procedure 1, delivering the product as a pink wax (71 mg, 36%). IR (film) 3282, 3092, 1692, 1515, 1491 cm^{-1}; R_f = 0.2 (30 % EtOAc/hex); ^{1}H NMR (400 MHz, acetone-d_6) δ 9.85 (br s, 2H), 7.76 (s, 1H), 7.58-7.54 (m, 2H). 7.54 (s, 1H), 7.20-7.13 (m, 4H); ^{13}C NMR (150 MHz, acetone-d_6) ppm 164.4 (dd, $^1J_{CF}$ = 248.2, $^3J_{CF}$ = 11.6 Hz), 161.3 (dd, $^1J_{CF}$ = 250.4, $^3J_{CF}$ = 13.8 Hz), 136.3, 134.9, 134.8 (q, $^3J_{CF}$ = 4.8 Hz), 134.0, 126.8, 122.3, 122.2 (d, $^4J_{CF}$ = 3.3 Hz), 113.1 ($^2J_{CF}$ = 21.6, $^4J_{CF}$ = 3.9 Hz), 105.5 (dd, $^2J_{CF}$ = 26.0, 26.0 Hz); ^{19}F NMR (282 MHz, acetone-d_6) ppm -77.7, -109.5, -110.1; HRMS (ESI): Exact mass calcd for $C_{20}H_9F_{10}N_2O_4S_2$ $[M-H]^-$ 594.9850, found 594.9839.

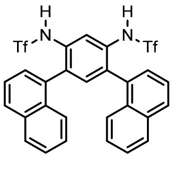

***N*,*N*'-(4,6-Di(naphthalen-1-yl)-1,3-phenylene)bis(1,1,1-trifluoromethanesulfonamide) (K3)**. Corresponding aniline (39 mg, 110 μmol) was used according to the General Procedure 1, delivering the product as a brown solid (49 mg, 67%). Mp 191-193 °C; IR (film) 3273, 3061, 1696, 1502, 1427 cm^{-1}; R_f = 0.15 (40 % EtOAc/hexanes); ^{1}H NMR (600 MHz, acetone-d_6) δ 9.84 (br s, 2H), 8.05 (s, 1H), 8.00-7.95 (m, 4H), 7.74-7.69 (m, 1H), 7.65-7.56 (m, 5H), 7.56-7.49 (m, 4H), 7.44 (d, J = 10.8 Hz, 1H); ^{13}C NMR (150 MHz, acetone-d_6) ppm 136.5, 136.2, 134.8, 134.7, 134.5, 132.8, 129.8, 129.3, 129.2, 127.5, 127.0, 126.3, 126.2, 123.1, 120.7 (q, $^1J_{CF}$ = 322.3 Hz); ^{19}F NMR (282 MHz, acetone-d_6) ppm -77.7; HRMS (ESI): Exact mass calcd for $C_{28}H_{17}F_6N_2O_4S_2$ $[M-H]^-$ 623.0539, found 623.0534.

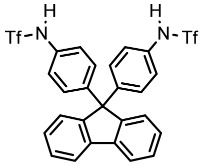

***N*,*N*'-((9H-fluorene-9,9-diyl)bis(4,1-phenylene))bis(1,1,1-trifluoromethanesulfonamide) (K4)**. Corresponding aniline (300 mg, 860 μmol) was used according to the General Procedure 1, delivering the product as a white solid (63 mg, 12%). Mp 151-152 °C; IR (film) 3284, 3063, 2923, 1692, 1508, 1444, 1413 cm^{-1}; R_f = 0.30 (30 % EtOAc/hexanes); ^{1}H NMR (400 MHz, $CDCl_3$) δ 7.78 (d, J = 7.6 Hz, 1H), 7.40 (ddd, J = 7.6, 7.6, 1.6 Hz, 1H), 7.33 (d, J = 6.7 Hz, 1H), 7.30 (dd, J = 6.8, 1.0 Hz, 1H), 7.19 (dd, J = 6.6, 2.1 Hz, 2H), 7.13 (dd, J = 6.8, 6.8 Hz, 2H), 6.68 (br s, 1H); ^{13}C NMR (150 MHz, acetone-d_6) ppm 151.3, 145.3, 141.0, 134.6, 132.0, 130.5, 129.9, 128.9, 127.0, 123.9, 121.4, 120.9 (q, $^1J_{CF}$ = 323.5 Hz); ^{19}F NMR (282 MHz, $CDCl_3$) ppm -75.6; HRMS (ESI): Exact mass calcd for $C_{27}H_{17}F_6N_2O_4S_2$ $[M-H]^-$ 611.0539, found 611.0536.

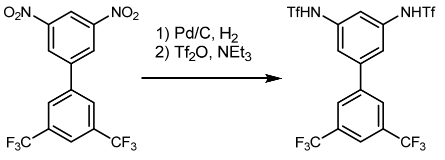

***N*,*N*'-(3',5'-bis(trifluoromethyl)-[1,1'-biphenyl]-3,5-diyl)bis(1,1,1-trifluoromethanesulfonamide) (K5)**. A 15 mL round-bottom flask was charged with nitroarene (115 mg, 0.30 mmol), Pd/C (58 mg, 50% by weight), and methanol (5 mL). The reaction was purged and refilled three times with argon, and then hydrogen gas. The reaction was monitored by TLC, and full conversion was observed after 6 h. The flask was purged with argon, and then passed through Celite with ethyl acetate. The crude material was obtained after concentration and used without further purification.

The triflation reaction was carried out according to the General Procedure 1, using the crude diamine, triethylamine (86 μL, 62 μmol), and trifluoromethane sulfonic anhydride (104 μL, 62 μmol). The crude material was separated by flash chromatography (SiO_2, 5-25% ethyl acetate in hexanes) to afford a white wax (97 mg, 57%). IR (film) 3281, 3265, 3014, 2974, 1603, 1515, 1484 cm^{-1}; R_f = 0.3 (50 % EtOAc/hexanes); ^{1}H NMR (400 MHz, acetone-d_6) δ 10.57 (br s, 2H), 8.28 (br s, 2H), 8.17 (br s, 1H), 7.26 (d, J = 2.2 Hz, 2H), 7.62 (t, J = 1.8 Hz, 1H); ^{13}C NMR (100 MHz, acetone-d_6) ppm 142.4, 141.6, 138.03, 137.97, 133.0 (q, $^2J_{CF}$ = 33.1 Hz), 128.7 (q, $^3J_{CF}$ = 4.1 Hz), 123.0 (q, $^2J_{CF}$ = 4.1 Hz), 121.0 ($^1J_{CF}$ = 322.1 Hz), 120.7, 117.1; ^{19}F NMR (282 MHz, acetone-d_6) ppm -63.4, -76.9; HRMS (ESI): Exact mass calcd for $C_{16}H_8F_{12}N_2O_4S_2$ $[M]^+$ 583.9734, found 583.9738.

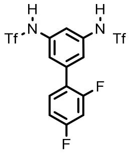

***N*,*N*'-(2',4'-Difluoro-[1,1'-biphenyl]-3,5-diyl)bis(1,1,1-trifluoromethanesulfonamide) (K6)**. The corresponding aniline (42 mg, 190 μmol) was used according to the General Procedure 1, delivering the product as a brown solid (41 mg, 45%). Mp 158-160 °C; IR (film) 3285, 3062, 1607, 1497, 1419 cm^{-1}; R_f = 0.30 (30 % EtOAc/hexanes); ^{1}H NMR (600 MHz, acetone-d_6) δ 10.61 (br s, 2H), 7.61 (dd, J = 10.5, 5.7 Hz, 1H), 7.54-7.49 (m, 3H), 7.23-7.15 (m, 2H); ^{13}C NMR (150 MHz, acetone-d_6) ppm 163.9 (dd, $^1J_{CF}$ = 249.4 Hz, $^3J_{CF}$ = 12.7 Hz), 160.6 (dd, $^1J_{CF}$ = 250.2 Hz, $^3J_{CF}$ = 12.1 Hz), 138.4, 137.4, 132.9 (d, $^3J_{CF}$ = 10.6 Hz), 124.1, 121.8, 120.9 (q, $^1J_{CF}$ = 321.6 Hz), 116.4, 113.2 (dd, $^2J_{CF}$ = 21.7 Hz, $^4J_{CF}$ = 3.4 Hz), 105.4 (dd, $^2J_{CF}$ = 24.7, 24.7 Hz); ^{19}F NMR (282 MHz, acetone-d_6) ppm -76.8, -110.9, -114.4; HRMS (ESI): Exact mass calcd for $C_{14}H_7F_8N_2O_4S_2$ [M-H]$^-$ 482.9725, found 482.9725.

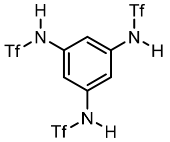

***N*,*N*',*N*''-(Benzene-1,3,5-triyl)tris(1,1,1-trifluoromethanesulfonamide) (K7)**. Corresponding aniline (100 mg, 810 μmol) was used according to the General Procedure 1, delivering the product as a pale pink solid (40 mg, 20%). Mp 156-158 °C; IR (film) 3272, 3082, 1685, 1614, 1513 cm^{-1}; R_f = 0.4 (30 % EtOAc/hexanes); ^{1}H NMR (400 MHz, acetone-d_6) δ 10.63 (br s, 3H), 7.38 (s, 3H); ^{13}C NMR (100 MHz, acetone-d_6) ppm 138.2, 120.8 (q, $^1J_{CF}$ = 322.4 Hz), 113.4; ^{19}F NMR (282 MHz, acetone-d_6) ppm -76.9; HRMS (ESI): Exact mass calcd for $C_9H_5F_9N_3O_6S_3$ [M-H]$^-$ 517.9202, found 517.9193.

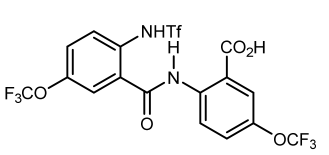

5-(Trifluoromethoxy)-2-(5-(trifluoromethoxy)-2-((trifluoromethyl)sulfonamido)benzamido)benzoic acid (K8). Corresponding aniline (526 mg, 2.4 mmol) was used according to the General Procedure 1, delivering the product as a yellow solid (262 mg, 40%). Mp 180-183 °C; R_f = 0.2 (20% acetone/hexanes); IR (film) 3205, 3094, 1662, 1599, 1540, 1504, 1483, 1404 cm^{-1}; ^{1}H NMR (400 MHz, acetone-d_6) δ 11.3 (br s, 1H), 8.85 (d, *J* = 9.2 Hz, 1H), 8.08 (d, *J* = 2.6 Hz, 1H), 7.98 (d, *J* = 2.5 Hz, 1H), 7.88 (d, *J* = 9.1 Hz, 1H), 7.73 (m, 2H) [N*H*Tf not observed] [N*H*CO not observed]; ^{13}C NMR (150 MHz, acetone-d_6) ppm 168.6, 165.3, 146.3, 144.1, 139.7, 134.9, 127.5, 126.3, 126.1, 124.7, 123.8, 122.4, 121.3, 121.1 (d, $^2J_{CF}$ = 12.4 Hz), 119.9 (q, $^1J_{CF}$ = 325.2 Hz), 119.7 (d, $^2J_{CF}$ = 12.4 Hz), 118.0; ^{19}F NMR (282 MHz, acetone-d_6) δ -58.9, -59.0, -77.1; HRMS (ESI): Exact mass calcd for $C_{17}H_8F_9N_2O_7S$ [M-H]$^-$ 554.9914, found 554.9919.

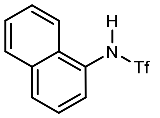

1,1,1-Trifluoro-*N*-(naphthalen-1-yl)methanesulfonamide (L1). Corresponding aniline (300 mg, 2.1 mmol) was used according to the General Procedure 1, delivering the product as a white solid (81 mg, 14%). ^{1}H NMR (400 MHz, $CDCl_3$) δ 8.04 (d, *J* = 8.4 Hz, 1H), 7.91 (t, *J* = 9.0 Hz, 2H), 7.62 (m, 3H), 7.49 (t, *J* = 8.0 Hz, 1H), 7.02 (s, 1H). The characterization data (^{1}H NMR) was in agreement with the literature.[18]

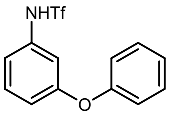

1,1,1-Trifluoro-*N*-(3-phenoxyphenyl)methanesulfonamide (L2). Corresponding aniline (280 mg, 1.5 mmol) was used according to the General Procedure 1, delivering the product as a white solid (223 mg, 48%). Mp 121-123 °C; IR (film) 3287, 1592, 1485 cm^{-1}; R_f = 0.6 (30% EtOAc/hexanes); ^{1}H NMR (400 MHz, $CDCl_3$) δ 7.40-7.35 (m, 2H), 7.32 (ddd, *J* = 8.4, 8.4, 0.9 Hz, 1H), 7.17 (tt, *J* = 7.4, 1.4 Hz, 1H), 7.05-6.97 (m, 3H), 6.94-6.90 (m, 2H), 6.66 (br s, 1H); ^{13}C NMR (100 MHz, $CDCl_3$) ppm 158.6, 156.1, 135.0, 130.8, 130.1, 124.3, 119.7 (q, $^1J_{CF}$ = 320.7 Hz), 119.5, 117.6, 117.3, 113.4; ^{19}F NMR (282 MHz, $CDCl_3$) ppm -76.8; HRMS (ESI): Exact mass calcd for $C_{13}H_{10}F_3NO_3S$ [M]$^+$ 317.0328, found 317.0319.

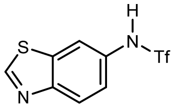

***N*-(Benzo[d]thiazol-6-yl)-1,1,1-trifluoromethanesulfonamide (L3)**. Corresponding aniline (300 mg, 2.0 mmol) was used according to the General Procedure 1, delivering the product as a colorless oil (16 mg, 3%). R_f = 0.3 (30% acetone/hexanes); IR (film) 3280, 3085, 2920, 1607, 1567, 1472, 1441, 1408 cm^{-1}; ^{1}H NMR (400 MHz, $CDCl_3$) δ 9.06 (s, 1H), 8.37 (s, 1H), 8.11 (d, *J* = 5.8 Hz, 1H), 7.97 (s, 1H), 7.42 (d, *J* = 5.8 Hz, 1H); ^{13}C NMR (150 MHz, $CDCl_3$) ppm 155.9, 152.0, 135.0, 131.8, 124.4, 122.9, 120.0 (q, $^1J_{CF}$ = 322.7 Hz), 117.5; ^{19}F NMR (282 MHz, $CDCl_3$) δ -75.7; HRMS (ESI): Exact mass calcd for $C_8H_4F_3N_2O_3S_2$ [M-H]$^-$ 280.9672, found 280.9676.

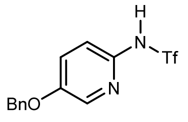

***N*-(5-(Benzyloxy)pyridin-2-yl)-1,1,1-trifluoromethanesulfonamide (L4)**. Corresponding aniline (240 mg, 1.2 mmol) was used according to the General Procedure 1, delivering the product as a light yellow solid (50 mg, 13%). Mp 141-143°C; R_f = 0.2 (20% acetone/hexanes); IR (film) 3137, 3022, 1703, 1651, 1613, 1546, 1518 cm^{-1}; ^1H NMR (400 MHz, acetone-d_6) δ 7.95 (dd, J = 9.7, 3.1 Hz, 1H), 7.90 (d, J = 2.7 Hz, 1H), 7.66 (d, J = 9.7 Hz, 1H), 7.50 (d, J = 8.1 Hz, 2H), 7.42 (m, 3H), 5.21 (s, 2H) [N*H* not observed]; ^{13}C NMR (150 MHz, acetone-d_6) ppm 152.4, 150.0, 136.8, 129.5, 129.2 (2C), 128.8, 122.5, 121.9 (q, $^1J_{CF}$ = 323.4 Hz), 120.0, 72.1; ^{19}F NMR (282 MHz, acetone-d_6) δ -79.2; HRMS (ESI): Exact mass calcd for $C_{13}H_{10}F_3N_2O_3S$ $[M\text{-}H]^-$ 331.0370, found 331.0373.

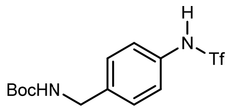

***tert*-Butyl (4-((trifluoromethyl)sulfonamido)benzyl)carbamate (L5)**. Corresponding aniline (528 mg, 2.4 mmol) was used according to the General Procedure 1, delivering the product as a white solid (330 mg, 39%). ^1H NMR (400 MHz, $CDCl_3$) δ 7.11 (m, 4H), 5.09 (s, 1H), 4.28 (d, J = 4.6 Hz, 2H), 1.48 (s, 9H) [N*H* not observed]. The characterization data (^1H NMR) was in agreement with the literature.[32]

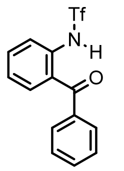

***N*-(2-Benzoylphenyl)-1,1,1-trifluoromethanesulfonamide (L6)**. Corresponding aniline (300 mg, 1.5 mmol) was used according to the General Procedure 1, delivering the product as a yellow solid (207 mg, 41%). ^1H NMR (400 MHz, $CDCl_3$) δ 10.52 (br s, 1H), 7.82 (d, J = 8.2 Hz, 1H), 7.72 (d, J = 7.3 Hz, 2H), 7.63 (m, 3H), 7.53 (dd, J = 7.6, 7.6 Hz, 2H), 7.28 (dd, J = 7.6, 7.6 Hz, 1H). The characterization data (^1H NMR) was in agreement with the literature.[33]

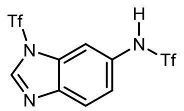

***N*-(1H-Benzo[d]imidazol-6-yl)-1,1,1-trifluoromethanesulfonamide (L7)**. Corresponding aniline (270 mg, 2.0 mmol) was used according to the General Procedure 1, delivering the product as a white solid (38 mg, 35%). Mp 174-176 °C; R_f = 0.2 (20% acetone/hexanes); IR (film) 3421, 3135, 3071, 2829, 1607, 1515, 1482, 1432, 1412 cm^{-1}; ^1H NMR (400 MHz, $CDCl_3$) δ 8.24 (s, 1H), 7.83 (m, 2H), 7.44 (dd, J = 8.8, 2.0 Hz, 1H) [N*H* not observed]; ^{13}C NMR (150 MHz, $CDCl_3$) ppm 145.4, 144.3, 134.2, 130.6, 123.6, 120.9 (q, $^1J_{CF}$ = 327.2 Hz), 120.1 (q, $^1J_{CF}$ = 324.0 Hz), 117.3, 114.5; ^{19}F NMR (282 MHz, $CDCl_3$) δ -75.1, -75.5; HRMS (ESI): Exact mass calcd for $C_9H_4F_6N_3O_4S_2$ $[M\text{-}H]^-$ 395.9553, found 395.9555.

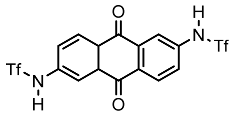

***N,N'*-(9,10-Dioxo-4a,9,9a,10-tetrahydroanthracene-2,6-diyl)bis(1,1,1-trifluoromethanesulfonamide) (L8)**. Corresponding aniline (360 mg, 1.5 mmol) was used according to the General Procedure 1, delivering the product as an orange solid (69 mg, 9%). Mp >400 °C; R_f = 0.2 (60% acetone/hexanes); IR (film) 3510, 3196, 2967, 1708, 1665, 1581, 1488, 1426 cm^{-1}; ^1H NMR (400 MHz, CD_3OD) δ 8.18 (d, J = 8.5 Hz, 2H), 7.99 (d, J = 2.3 Hz, 2H), 7.57 (dd, J = 8.5, 2.3 Hz, 2H) [N*H* not observed]; ^{13}C NMR (150 MHz, acetone-d_6) ppm 182.7, 151.3, 135.7, 129.0, 128.2, 127.3, 122.5, 120.0 (q, $^1J_{CF}$ = 322.2 Hz); ^{19}F NMR (282 MHz, CD_3OD) δ -78.2; HRMS (ESI): Exact mass calcd for $C_{16}H_7F_6N_2O_6S_2$ $[M\text{-}H]^-$ 500.9655, found 500.9659.

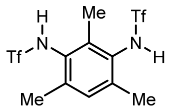

***N,N'*-(2,4,6-Trimethyl-1,3-phenylene)bis(1,1,1-trifluoromethanesulfonamide) (M1)**. Corresponding aniline (300 mg, 2.0 mmol) was used according to the General Procedure 1, delivering the product as a light yellow solid (121 mg, 21%). Mp 136-138 °C; R_f = 0.3 (30% acetone/hexanes); IR (film) 3277, 1693, 1515, 1421 cm^{-1}; ^1H NMR (400 MHz, acetone-d_6) δ 9.58 (br s, 2H), 7.24 (s, 1H), 2.47 (s, 3H), 2.40 (s, 6H) ; ^{13}C NMR (150 MHz, acetone-d) ppm 139.9, 139.4, 131.8, 131.3, 120.7 (q, $^1J_{CF}$ = 317.8 Hz), 18.9, 15.7; ^{19}F NMR (282 MHz, acetone-d_6) δ -77.8; HRMS (ESI): Exact mass calcd for $C_{11}H_{11}F_6N_2O_4S_2$ $[M\text{-}H]^-$ 413.0070, found 413.0074.

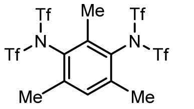

1,1,1-Trifluoro-*N*-((trifluoromethyl)sulfonyl)-*N*-(2,4,6-trimethyl-3-((trifluoromethyl)sulfonamido)phenyl)methanesulfonamide (M2). Corresponding aniline (300 mg, 2.0 mmol) was used according to the General Procedure 1, delivering the product as a white solid (68 mg, 10%). Mp 145-148 °C; R_f = 0.3 (10% acetone/hexanes); IR (film) 3454, 3021, 1441, 1422 cm^{-1}; ^{1}H NMR (400 MHz, $CDCl_3$) δ 7.29 (s, 1H), 2.54 (s, 3H), 2.48 (s, 6H); ^{13}C NMR (150 MHz, acetone-*d*) ppm 145.8, 143.6, 135.0, 131.2, 120.1 (q, $^1J_{CF}$ = 322.9 Hz), 19.9, 17.7; ^{19}F NMR (282 MHz, $CDCl_3$) δ -69.4; HRMS (ESI): Exact mass calcd for $C_{12}H_{10}F_9N_2O_6S_3$ [M-H]$^-$ 544.9563, found 544.9574.

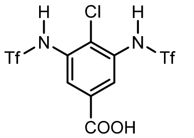

4-Chloro-3,5-bis((trifluoromethyl)sulfonamido)benzoic acid (M3). Corresponding aniline (36 mg, 192 μmol) was used according to the General Procedure 1, delivering the product as a white solid (60 mg, 71%). Mp 191-193 °C; R_f = 0.2 (40% acetone/hexanes); IR (film) 3268, 2968, 1713, 1580, 1432 cm^{-1}; ^{1}H NMR (400 MHz, acetone-d_6) δ 10.65 (br s, 2H), 8.23 (s, 2H), 6.64 (br s, 1H); ^{13}C NMR (150 MHz, acetone-*d*) ppm 165.0, 134.2, 131.5, 129.9, 125.8, 120.9 (q, $^1J_{CF}$ = 319.6 Hz); ^{19}F NMR (282 MHz, acetone-d_6) δ -77.6; HRMS (ESI): Exact mass calcd for $C_9H_4ClF_6N_2O_6S_2$ [M-H]$^-$ 448.9109, found 448.9113.

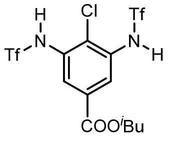

Isobutyl 4-chloro-3,5-bis((trifluoromethyl)sulfonamido)benzoate (M4). Corresponding aniline (485 mg, 2.0 mmol) was used according to the General Procedure 1, delivering the product as a light orange solid (201 mg, 25%). Mp 211-213 °C; R_f = (10% acetone/hexanes); IR (film) 3267, 2968, 1708, 1583, 1430 cm^{-1}; ^{1}H NMR (400 MHz, acetone-d_6) δ 8.23 (s, 2H), 4.19 (d, *J* = 6.5 Hz, 2H), 2.11 (m, 1H), 1.04 (d, *J* = 6.7 Hz, 6H) [N*H* not observed]; ^{13}C NMR (150 MHz, acetone-*d*) ppm 164.2, 134.2, 131.4, 129.5, 125.8, 120.7 (q, $^1J_{CF}$ = 320.7 Hz), 72.4, 28.6, 19.2; ^{19}F NMR (282 MHz, acetone-d_6) δ -77.6; HRMS (ESI): Exact mass calcd for $C_{13}H_{12}ClF_6N_2O_6S_2$ [M-H]$^-$ 504.9735, found 504.9741.

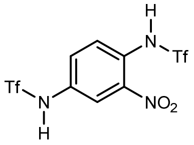

***N,N'*-(2-Nitro-1,4-phenylene)bis(1,1,1-trifluoromethanesulfonamide) (M5)**. Corresponding aniline (306 mg, 2.0 mmol) was used according to the General Procedure 1, delivering the product as a red oil (69 mg, 9%). R_f = 0.2 (30% acetone/hexanes); IR (film) 3492, 3381, 3097, 1637, 1596, 1570, 1521 cm^{-1}; ^{1}H NMR (400 MHz, acetone-d_6) δ 8.03 (d, *J* = 2.6 Hz, 1H), 7.45 (dd, *J* = 9.0, 2.6 Hz, 1H), 7.24 (br s, 2H), 7.15 (d, *J* = 9.0 Hz, 1H); ^{13}C NMR (150 MHz, acetone-d_6) ppm 145.3, 133.2, 124.9, 122.6, 122.0, 121.6 (q, $^1J_{CF}$ = 325.8 Hz), 120.2, 120.1 (q, $^1J_{CF}$ = 326.8 Hz); ^{19}F NMR (282 MHz, acetone-d_6) δ -75.4, -75.8; HRMS (ESI): Exact mass calcd for $C_8H_4F_6N_3O_6S_2$ [M-H]$^-$ 415.9451, found 415.9453.

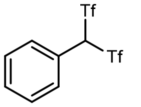

(Bis((trifluoromethyl)sulfonyl)methyl)benzene (M6). A flame-dried round-bottom flask was charged with (((trifluoromethyl)sulfonyl)methyl)benzene (180 mg, 800 μmol) dissolved in anhydrous diethyl ether (5 mL). The solution was cooled to -78 °C in a dry ice/acetone bath. Under an argon atmosphere, *n*-butyllithium (2.5 M in hexanes, 176 μL) was added dropwise, and the resulting mixture was stirred for 15 minutes. Trifluoromethanesulfonic anhydride (75 μL, 440 μmol) was then added dropwise, after which the reaction mixture was allowed to warm slowly to room temperature and stirred for 1 hour. The addition sequence of *n*-butyllithium and trifluoromethanesulfonic anhydride was repeated following the same procedure. The reaction was quenched by the careful addition of 1 N HCl in an ice bath. The aqueous layer was extracted with diethyl ether, and the combined organic extracts were dried over anhydrous Na_2SO_4, filtered, and concentrated under reduced pressure. The crude product was purified by distillation to afford the desired compound as a white solid (68 mg, 23%). ^{1}H NMR (400 MHz, $CDCl_3$) δ 8.18 (d, *J* = 8.5 Hz, 2H), 7.99 (d, *J* = 2.3 Hz, 2H), 7.57 (dd, *J* = 8.5, 2.3 Hz, 2H). The characterization data (^{1}H NMR) was in agreement with the literature. [34]

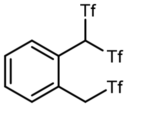

1-(Bis((trifluoromethyl)sulfonyl)methyl)-2-(((trifluoromethyl)sulfonyl)methyl)benzene (M7). A flame-dried round-bottom flask was charged with 1,2-bis(((trifluoromethyl)sulfonyl)methyl)benzene (310 mg, 840 μmol) dissolved in anhydrous diethyl ether (10 mL). The solution was cooled to -78 °C

in a dry ice/acetone bath. Under an argon atmosphere, *n*-butyllithium (2.5 M in hexanes, 740 µL) was added dropwise, and the resulting mixture was stirred for 15 minutes. Trifluoromethanesulfonic anhydride (221 µL, 1.3 mmol) was then added dropwise, after which the reaction mixture was allowed to warm slowly to room temperature and stirred for 1 hour. The addition sequence of *n*-butyllithium and trifluoromethanesulfonic anhydride was repeated following the same procedure. The reaction was quenched by the careful addition of 1 N HCl in an ice bath. The aqueous layer was extracted with diethyl ether, and the combined organic extracts were dried over anhydrous Na_2SO_4, filtered, and concentrated under reduced pressure. The crude product was purified by distillation to afford the desired compound as a light yellow oil (97 mg, 22%). R_f = 0.2 (40% acetone/hexanes); IR (film) 3466, 2971, 1693 cm^{-1}; ^1H NMR (400 MHz, $CDCl_3$) δ 8.28-8.21 (m, 1H), 7.76-7.67 (m, 2H), 7.61-7.55 (m, 1H) 6.68 (s, 1H), 4.56 (s, 2H); ^{13}C NMR (150 MHz, acetone-*d*) ppm 154.6, 139.7, 130.3, 129.1, 129.0, 128.8, 122.6 (q, $^1J_{CF}$ = 322.9 Hz), 120.5 (q, $^1J_{CF}$ = 327.9 Hz), 69.6, 53.5; ^{19}F NMR (282 MHz, $CDCl_3$) δ -71.5, -76.8; HRMS (ESI): Exact mass calcd for $C_{11}H_6F_9O_6S_3$ $[M-H]^-$ 500.9188, found 500.9196.

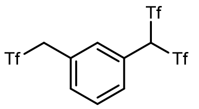

1-(Bis((trifluoromethyl)sulfonyl)methyl)-3-(((trifluoromethyl)sulfonyl)methyl)benzene (M8). A flame-dried round-bottom flask was charged with 1,2-bis(((trifluoromethyl)sulfonyl)methyl)benzene (220 mg, 596 µmol) dissolved in anhydrous diethyl ether (10 mL). The solution was cooled to -78 °C in a dry ice/acetone bath. Under an argon atmosphere, *n*-butyllithium (2.5 M in hexanes, 524 µL) was added dropwise, and the resulting mixture was stirred for 15 minutes. Trifluoromethanesulfonic anhydride (150 µL, 894 µmol) was then added dropwise, after which the reaction mixture was allowed to warm slowly to room temperature and stirred for 1 hour. The addition sequence of *n*-butyllithium and trifluoromethanesulfonic anhydride was repeated following the same procedure. The reaction was quenched by the careful addition of 1 N HCl in an ice bath. The aqueous layer was extracted with diethyl ether, and the combined organic extracts were dried over anhydrous Na_2SO_4, filtered, and concentrated under reduced pressure. The crude product was purified by distillation to afford the desired compound as a brown oil (98 mg, 33%). R_f = 0.2 (40% acetone/hexanes); IR (film) 3435, 2950, 1692 cm^{-1}; ^1H NMR (400 MHz, $CDCl_3$) δ 7.78-7.62 (m, 4H), 5.93 (s, 1H), 4.55 (s, 2H); ^{13}C NMR (150 MHz, acetone-d_6) ppm 154.3, 136.8, 131.4, 128.1, 125.8, 122.0, 120.7 (q, $^1J_{CF}$ = 328.0 Hz), 120.2 (q, $^1J_{CF}$ = 328.0 Hz), 69.3, 55.7; ^{19}F NMR (282 MHz, $CDCl_3$) δ -71.4, -73.6; HRMS (ESI): Exact mass calcd for $C_{11}H_6F_9O_6S_3$ $[M-H]^-$ 500.9188, found 500.9190.

General Procedure 2 for Enantioselective Azide Addition to Nitroalkenes

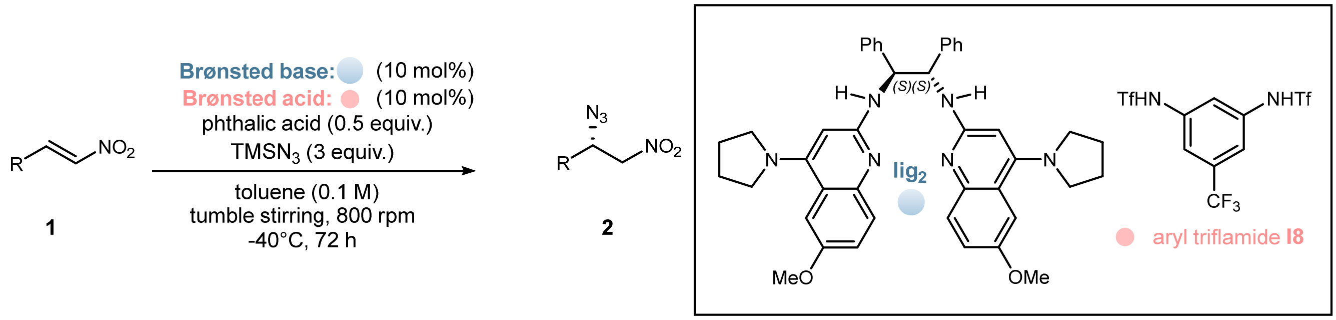

A 1 mL flat-bottom clear glass insert was charged with nitroalkene (1 equiv.), chiral ligand **Lig2** (10 mol%), and aryl triflamide **I8** (10 mol%) in a stock solution (chloroform or acetone). The solvent was then evaporated using an EqualVAP@ 96-well blowdown evaporator. Phthalic acid (0.5 equiv.) was subsequently added as a stock solution (acetone), and the solvent was evaporated. The insert was placed in a 96-well plate, cooled to -40 °C, and azidotrimethylsilane (3.0 equiv.) was added in a stock solution (toluene). The insert was sealed using a round well cap mat with a molded silicone/PTFE liner and stirred using a VP@ 710C5-7A vertical tumble stirrer for 72

hours. The reaction mixture was worked up by filtering through Thermo Scientific™ HyperSep™ silica plates using a vacuum manifold system, and the filtrate was collected in a 2 mL flat-bottom clear glass insert. Each silica plate cell was eluted with 20% acetone in hexanes (300 μL), and the filtrate was evaporated. The reaction mixture was then dissolved in 50% isopropanol in hexane (HPLC grade) and transferred into a 2 mL HPLC vial using an INTEGRA@ 8-channel VOYAGER adjustable tip spacing pipette. The enantiomeric excess was determined by chiral HPLC. The assay was obtained using a racemic catalyst **lig₂** following the same procedure. The absolute configuration of **2r** was assigned as (*S*) based on the literature reference,[35] and the rest of the compounds were assigned by analogy. All the reactions were performed at 20 μmol scale and the yields were obtained by using dibromomethane as internal standard. The examples **2b**, **2c**, **2h**, **2i**, **2m**, **2o**, **2p**, **2q** were reproduced at 100 μmol scale to determine accuracy and reproducibility and the crude reactions were purified by silica gel chromatography (SiO_2, 1-3% diethyl ether in hexanes) to afford the pure products..

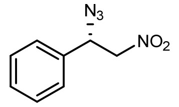

(*S*)-(1-Azido-2-nitroethyl)benzene (2a). The reaction was carried out according to General Procedure 2 to obtain a light yellow oil (87% yield, NMR) that was determined to be 85% ee by chiral HPLC (Chiralcel OD-H, 30% IPA/hexanes, 1.0 mL/min: t_R(minor) = 11.4 min, t_R(major) = 25.5 min). The reaction was also carried out at 100 μmol scale to obtain the product (18 mg, 95%) with 85% ee. The characterization data (^{1}H NMR) was in agreement with the literature.[35] ^{1}H NMR (400 MHz, $CDCl_3$) δ 7.49-7.41 (m, 3H), 7.40-7.33 (m, 2H), 5.32 (dd, *J* = 9.9, 4.4 Hz, 1H), 4.59 (dd, *J* = 13.4, 9.9 Hz, 1H), 4.49 (dd, *J* = 13.4, 4.4 Hz, 1H).

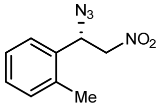

(*S*)-1-(1-Azido-2-nitroethyl)-2-methylbenzene (2b). The reaction was carried out according to the General Procedure 2 to obtain a light yellow oil (31% yield, NMR) that was determined to be 61% ee by chiral HPLC (Chiralcel OJ-H, 30% IPA/hexanes, 1.0 mL/min: t_R(minor) = 10.1 min, t_R(major) = 22.2 min). $[\alpha]_D^{20}$ +77.4 (*c* 0.81, $CHCl_3$); R_f = 0.2 (5% EtOAc/hexanes); IR (film) 3108, 3062, 2965, 2924, 2109, 1631, 1601, 1557, 1514, 1486, 1462, 1420 cm^{-1}; ^{1}H NMR (400 MHz, $CDCl_3$) δ 7.40-7.22 (m, 4H), 5.59 (d, *J* = 6.7 Hz, 1H), 4.60 (dd, *J* = 8.1, 8.1 Hz, 1H), 4.41 (d, *J* = 9.1 Hz, 1H), 2.45 (s, 3H); ^{13}C NMR (150 MHz, $CDCl_3$) ppm 135.7, 132.4, 131.5, 129.5, 127.2, 126.6, 77.9, 59.4, 19.3; HRMS (APCI): Exact mass calcd for $C_9H_{11}N_2O_2$ $[M-N_2+H]^-$ 179.0185, found 179.0814.

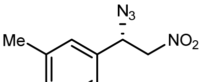

(*S*)-1-(1-Azido-2-nitroethyl)-3-methylbenzene (2c). The reaction was carried out according to the General Procedure 2 to obtain a light yellow oil (69% yield, NMR) that was determined to be 71% ee by chiral HPLC (Chiralcel IB, 5% IPA/hexanes, 1.0 mL/min: t_R(minor) = 12.6 min, t_R(major) = 24.5 min). The reaction was also carried out at 100 μmol scale to obtain the product (20 mg, 95%) with 74% ee. $[\alpha]_D^{20}$ +86.5 (*c* 1.00, $CHCl_3$, 74% ee); R_f = 0.3 (10% EtOAc/hexanes); IR (film) 3110, 3027, 2923, 2123, 1634, 1601, 1557, 1519, 1490, 1456, 1421 cm^{-1}; ^{1}H NMR (400 MHz, $CDCl_3$) δ 7.33 (dd, *J* = 4.3, 4.3 Hz, 1H), 7.22 (d, *J* = 5.0 Hz, 1H), 7.15 (m, 2H), 5.27 (d, *J* = 6.5 Hz, 1H), 4.58 (dd, *J* = 8.2, 8.2 Hz, 1H), 4.43 (d, *J* = 9.0 Hz, 1H), 2.39 (s, 3H); ^{13}C NMR (150 MHz, $CDCl_3$) ppm 139.6, 134.2, 130.6, 129.5, 127.8, 124.2, 78.8, 62.8, 21.6; HRMS (APCI): Exact mass calcd for $C_9H_{11}N_2O_2$ $[M-N_2+H]^+$ 179.0185, found 179.0813.

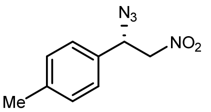

(*S*)-1-(1-Azido-2-nitroethyl)-4-methylbenzene (2d). The reaction was carried out according to the General Procedure 2 to obtain a light yellow oil (61% yield, NMR) that was determined to be 77% ee by chiral HPLC (Chiralcel IB, 30% IPA/hexanes, 1.0 mL/min: t_R(minor) = 7.7 min, t_R(major) = 12.6 min). $[\alpha]_D^{20}$ +79.1 (*c* 0.53, $CHCl_3$); R_f = 0.3 (10% EtOAc/hexanes); IR (film) 3110, 3030, 2922, 2116, 1632, 1610, 1558, 1516, 1504, 1417 cm^{-1}; ^{1}H NMR (400 MHz, $CDCl_3$) δ 7.49 (d, *J* = 5.2 Hz, 2H), 7.28 (d, *J* = 5.2 Hz, 2H), 5.28 (dd, *J* = 7.0, 2.5 Hz, 1H), 4.60 (dd, *J* = 8.0, 8.0 Hz, 1H), 4.46 (dd, *J* = 8.9, 2.5 Hz, 1H), 2.38 (s, 3H); ^{13}C NMR (150 MHz, $CDCl_3$) ppm 140.0, 131.2, 130.3, 127.1, 78.5, 62.5, 21.3; HRMS (APCI): Exact mass calcd for $C_9H_{11}N_2O_2$ $[M-N_2+H]^+$ 179.0185, found 179.0812.

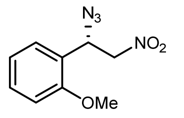

(*S*)-1-(1-Azido-2-nitroethyl)-2-methoxylbenzene (2e). The reaction was carried out according to the General Procedure 2 to obtain a light yellow oil (36% yield, NMR) that was determined to be 80% ee by chiral HPLC (Chiralcel IB, 5% IPA/hexanes, 1.0 mL/min: t_R(minor) = 9.8 min, t_R(major) = 17.1 min). $[\alpha]_D^{20}$ +71.3 (*c* 0.82, $CHCl_3$); R_f = 0.25 (10% EtOAc/hexanes); IR (film) 3150, 3107, 3010, 2940, 2114, 1627, 1599, 1574, 1556, 1508, 1491, 1464, 1437 cm^{-1}; ^1H NMR (400 MHz, $CDCl_3$) δ 7.43-7.35 (m, 2H), 7.05 (dd, *J* = 7.5, 7.5 Hz, 1H), 6.96 (d, *J* = 8.8 Hz, 1H), 5.72 (dd, *J* = 9.7, 3.8 Hz, 1H), 4.61 (dd, *J* = 13.6, 3.9 Hz, 1H), 4.53 (dd, *J* = 13.5, 9.7 Hz, 1H), 3.90 (s, 3H); ^{13}C NMR (150 MHz, $CDCl_3$) ppm 159.6, 130.7, 127.8, 122.6, 121.4, 111.1, 77.6, 58.0, 55.7; HRMS (APCI): Exact mass calcd for $C_9H_{11}N_2O_3$ $[M-N_2+H]^+$ 195.0764, found 195.0763.

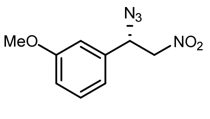

(*S*)-1-(1-Azido-2-nitroethyl)-3-methoxylbenzene (2f). The reaction was carried out according to the General Procedure 2 to obtain a yellow oil (65% yield, NMR) that was determined to be 92% ee by chiral HPLC (Chiralcel IB, 30% IPA/hexanes, 1.0 mL/min: t_R(minor) = 10.2 min, t_R(major) = 24.5 min). $[\alpha]_D^{20}$ +87.5 (*c* 0.73, $CHCl_3$); R_f = 0.3 (10% EtOAc/hexanes); IR (film) 3111, 2970, 2840, 2122, 1638, 1604, 1557, 1515, 1495, 1432 cm^{-1}; ^1H NMR (400 MHz, $CDCl_3$) δ 7.36 (dd, *J* = 7.9, 7.9 Hz, 1H), 6.97-6.91 (m, 2H), 6.87 (d, *J* = 2.0, 2.0 Hz, 1H), 5.31 (dd, *J* = 9.9, 4.3 Hz, 1H), 4.58 (dd, *J* = 13.4, 9.9 Hz, 1H), 4.46 (dd, *J* = 13.4, 4.3 Hz, 1H), 3.84 (s, 3H); ^{13}C NMR (150 MHz, $CDCl_3$) ppm 160.5, 135.8, 130.7, 119.2, 115.1, 112.8, 78.6, 62.6, 55.6; HRMS (APCI): Exact mass calcd for $C_9H_{11}N_2O_3$ $[M-N_2+H]^+$ 195.0764, found 195.0764.

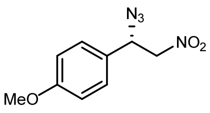

(*S*)-1-(1-Azido-2-nitroethyl)-4-methoxylbenzene (2g). The reaction was carried out according to the General Procedure 2 to obtain a yellow oil (18 mg, 64%) that was determined to be 83% ee by chiral HPLC (Chiralcel IB, 20% IPA/hexanes, 10.6 mL/min: t_R(minor) = 17.2 min, t_R(major) = 17.1 min). $[\alpha]_D^{20}$ +90.5 (*c* 1.00, $CHCl_3$); R_f = 0.2 (10% EtOAc/hexanes); IR (film) 3107, 2962, 2936, 2112, 1625, 1604, 1557, 1515, 1497, 1425 cm^{-1}; ^1H NMR (400 MHz, $CDCl_3$) δ 7.29 (d, *J* = 8.7 Hz, 2H), 6.94 (d, *J* = 8.7 Hz, 2H), 5.25 (dd, *J* = 9.8, 4.6 Hz, 1H), 4.58 (dd, *J* = 13.2, 9.8 Hz, 1H), 4.44 (dd, *J* = 13.2, 4.6 Hz, 1H), 3.82 (s, 3H); ^{13}C NMR (150 MHz, $CDCl_3$) ppm 160.7, 128.5, 126.1, 114.9, 78.7, 62.2, 55.5; HRMS (APCI): Exact mass calcd for $C_9H_{11}N_2O_3$ $[M-N_2+H]^+$ 195.0764, found 195.0764.

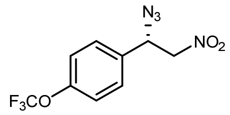

(*S*)-2-Azido-1-nitro-2-(4-(trifluoromethoxy)phenyl)ethan-1-ide (2h). The reaction was carried out according to the General Procedure 2 to obtain a light yellow oil (46% yield, NMR) that was determined to be 63% ee by chiral HPLC (Chiralcel IC, 2% IPA/hexanes, 1.0 mL/min: t_R(minor) = 9.2 min, t_R(major) = 10.6 min). The reaction was also carried out at 100 μmol scale to obtain the product (26 mg, 95%) with 83% ee. $[\alpha]_D^{20}$ +56.3 (*c* 0.60, $CHCl_3$, 83% ee); R_f = 0.3 (10% EtOAc/hexanes); IR (film) 3112, 2923, 2113, 1636, 1509, 1587, 1561, 1525, 1509 cm^{-1}; ^1H NMR (400 MHz, $CDCl_3$) δ 7.43 (d, *J* = 8.6 Hz, 2H), 7.32 (d, *J* = 8.3 Hz, 2H), 5.35 (dd, *J* = 9.5, 4.4 Hz, 1H), 4.58 (dd, *J* = 13.5, 9.7 Hz, 1H), 4.45 (dd, *J* = 13.5, 4.6 Hz, 1H); ^{13}C NMR (150 MHz, $CDCl_3$) ppm 137.8, 133.0, 128.7, 122.0, 120.6 ($^1J_{CF}$ = 258.5 Hz), 78.4, 61.9; ^{19}F NMR (282 MHz, $CDCl_3$) δ -57.5; HRMS (APCI): Exact mass calcd for $C_9H_8F_3N_2O_3$ $[M-N_2+H]^+$ 249.0482, found 249.0480.

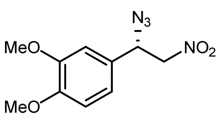

(*S*)-4-(1-Azido-2-nitroethyl)-1,2-dimethoxybenzene (2i). The reaction was carried out according to the General Procedure to obtain a yellow solid (16% yield, NMR) that was determined to be 82% ee by chiral HPLC (Chiralcel IB, 20% IPA/hexanes, 1.0 mL/min: t_R(minor) = 18.9 min, t_R(major) = 20.7 min). The reaction was also carried out at 100 μmol scale to obtain the product (6 mg, 25%) with 79% ee. Mp 82-85 °C; $[\alpha]_D^{20}$ +66.1 (*c* 0.60, $CHCl_3$, 79% ee); R_f = 0.2 (30% EtOAc/hexanes); IR (film) 3108, 2963, 2839, 2113, 1605, 1556, 1515, 1496, 1425 cm^{-1}; ^1H NMR (400 MHz, $CDCl_3$) δ 6.94-6.89 (m, 2H), 6.82 (d, *J* = 1.6 Hz, 1H), 5.29 (dd, *J* = 9.8, 4.5 Hz, 1H), 4.58 (dd, *J* = 13.2, 9.8 Hz, 1H), 4.46 (dd, *J* = 13.2, 4.5 Hz, 1H), 3.92 (s, 3H), 3.90 (s, 3H); ^{13}C NMR (150 MHz, $CDCl_3$) ppm 150.2,

149.9, 126.5, 119.8, 111.6, 109.7, 78.8, 62.6, 56.2, 56.1; HRMS (APCI): Exact mass calcd for $C_{10}H_{13}N_2O_4$ [M-N_2+H]$^+$ 225.0870, found 225.0874.

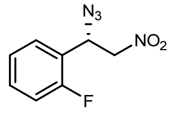

(*S*)-1-(1-Azido-2-nitroethyl)-2-fluorobenzene (2j). The reaction was carried out according to the General Procedure 2 to obtain a colorless oil (18 mg, 86%) that was determined to be 65% ee by chiral HPLC (Chiralcel IB, 5% IPA/hexanes, 1.0 mL/min: t_R(minor) = 9.8 min, t_R(major) = 14.3 min). $[\alpha]_D^{20}$ +73.6 (*c* 1.00, $CHCl_3$); R_f = 0.3 (10% EtOAc/hexanes); IR (film) 3112, 3061, 2921, 2106, 1633, 1557, 1520, 1470, 1440 cm^{-1}; ^{1}H NMR (400 MHz, $CDCl_3$) δ 7.48-7.38 (m, 2H), 7.29-7.23 (m, 1H), 7.16 (dd, *J* = 9.5, 8.4 Hz, 1H), 5.64 (dd, *J* = 9.3, 4.4 Hz, 1H), 4.64 (dd, *J* = 13.7, 9.5 Hz, 1H), 4.56 (dd, *J* = 13.8, 4.6 Hz, 1H); ^{13}C NMR (150 MHz, $CDCl_3$) ppm 160.1 ($^1J_{CF}$ = 274.8 Hz), 131.6 ($^3J_{CF}$ = 8.0 Hz), 128.6 ($^4J_{CF}$ = 3.2 Hz), 125.4, 121.7 ($^2J_{CF}$ = 14.3 Hz), 116.5 ($^2J_{CF}$ = 20.8 Hz), 77.1, 57.0 ; ^{19}F NMR (282 MHz, $CDCl_3$) δ -117.0; HRMS (APCI): Exact mass calcd for $C_8H_4FN_2O_2$ [M-N_2+H]$^+$ 183.0564, found 183.0563.

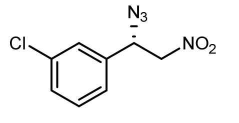

(*S*)-1-(1-Azido-2-nitroethyl)-3-chlorobenzene (2k). The reaction was carried out according to the General Procedure 2 to obtain a light yellow oil (78% yield, NMR) that was determined to be 79% ee by chiral HPLC (Chiralcel IB, 30% IPA/hexanes, 1.0 mL/min: t_R(minor) = 7.6 min, t_R(major) = 13.6 min). $[\alpha]_D^{20}$ +64.3 (*c* 1.00, $CHCl_3$); R_f = 0.3 (10% EtOAc/hexanes); IR (film) 3110, 2922, 2121, 1638, 1558, 1520 cm^{-1}; ^{1}H NMR (400 MHz, $CDCl_3$) δ 7.44-7.35 (m, 3H), 7.28-7.22 (m, 1H), 5.33 (dd, *J* = 9.7, 4.4 Hz, 1H), 4.59 (dd, *J* = 13.5, 9.7 Hz, 1H), 4.44 (dd, *J* = 13.6, 4.5 Hz, 1H); ^{13}C NMR (150 MHz, $CDCl_3$) ppm 138.1, 136.3, 131.8, 130.8, 130.0, 125.1, 78.3, 61.9; HRMS (APCI): Exact mass calcd for $C_8H_8ClN_2O_2$ [M-N_2+H]$^+$ 199.0269, found 199.0266.

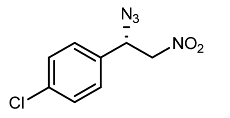

(*S*)-1-(1-Azido-2-nitroethyl)-4-chlorobenzene (2l). The reaction was carried out according to the General Procedure 2 to obtain a light yellow oil (67% yield, NMR) that was determined to be 90% ee by chiral HPLC (Chiralcel IB, 20% IPA/hexanes, 1.0 mL/min: t_R(minor) = 8.8 min, t_R(major) = 14.0 min). $[\alpha]_D^{20}$ +74.5 (*c* 0.53, $CHCl_3$); R_f = 0.2 (10% EtOAc/hexanes); IR (film) 3112, 3061, 2969, 2922, 2128, 1634, 1591, 1558, 1523, 1471 cm^{-1}; ^{1}H NMR (400 MHz, $CDCl_3$) δ 7.43 (d, *J* = 5.4 Hz, 2H), 7.31 (d, *J* = 5.5 Hz, 2H), 5.30 (dd, *J* = 6.5, 3.0 Hz, 1H), 4.59 (dd, *J* = 9.0, 6.5 Hz, 1H), 4.44 (dd, *J* = 9.0, 3.0 Hz, 1H); ^{13}C NMR (150 MHz, $CDCl_3$) ppm 135.9, 132.8, 129.9, 128.5, 78.4, 62.0; HRMS (APCI): Exact mass calcd for $C_8H_8ClN_2O_2$ [M-N_2+H]$^+$ 199.0269, found 199.0265.

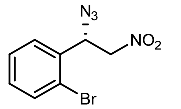

(*S*)-1-(1-Azido-2-nitroethyl)-2-bromobenzene (2m). The reaction was carried out according to the General Procedure 2 to obtain a light yellow oil (83% yield, NMR) that was determined to be 50% ee by chiral HPLC (Chiralcel IB, 10% IPA/hexanes, 1.0 mL/min: t_R(minor) = 9.3 min, t_R(major) = 17.3 min). The reaction was also carried out at 100 μmol scale to obtain the product (27 mg, 99%) with 50% ee. $[\alpha]_D^{20}$ +35.6 (*c* 1.00, $CHCl_3$); R_f = 0.3 (10% EtOAc/hexanes); IR (film) 3112, 3059, 2923, 2852, 2129, 1634, 1586, 1557, 1525, 1467, 1419 cm^{-1}; ^{1}H NMR (400 MHz, $CDCl_3$) δ 7.64 (dd, *J* = 8.0, 0.9 Hz, 1H), 7.55-7.48 (m, 1H), 7.43 (ddd, *J* = 7.5, 7.5, 0.6 Hz, 1H), 7.29 (ddd, *J* = 7.9, 7.9, 1.6 Hz, 1H), 5.83 (dd, *J* = 10.2, 3.2 Hz, 1H), 4.60 (dd, *J* = 13.8, 3.2 Hz, 1H), 4.42 (dd, *J* = 13.8, 10.2 Hz, 1H); ^{13}C NMR (150 MHz, $CDCl_3$) ppm 134.0, 133.7, 131.0, 128.7, 128.5, 122.8, 77.2, 61.9; HRMS (APCI): Exact mass calcd for $C_8H_8BrN_2O_2$ [M-N_2+H]$^+$ 242.9764, found 242.9760. Applying aryl triflamide M4 with the same procedure gave 79% yield with 70% ee.

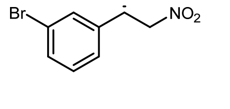

(*S*)-1-(1-Azido-2-nitroethyl)-3-bromobenzene (2n). The reaction was carried out according to the General Procedure 2 to obtain a light yellow oil (82% yield, NMR) that was determined to be 73% ee by chiral HPLC (Chiralcel IB, 30% IPA/hexanes, 1.0 mL/min: t_R(minor) = 9.0 min, t_R(major) = 15.7 min). $[\alpha]_D^{20}$ +43.2 (*c* 0.56, $CHCl_3$); R_f = 0.3 (10% EtOAc/hexanes); IR (film) 3110, 3061, 2921, 2117, 1637, 1558, 1519, 1474, 1425 cm^{-1}; ^{1}H NMR (400 MHz, $CDCl_3$) δ 7.60-7.50 (m, 2H), 7.36-7.28 (m, 2H), 5.31 (dd, *J* = 9.7, 4.4 Hz, 1H), 4.56 (dd, *J* = 13.6, 9.7 Hz, 1H), 4.45 (dd, *J* = 13.6, 4.5 Hz,

1H); ^{13}C NMR (150 MHz, $CDCl_3$) ppm 136.6, 133.0, 131.0, 130.2, 125.7, 123.6, 78.4, 62.0; HRMS (APCI): Exact mass calcd for $C_8H_8BrN_2O_2$ $[M\text{-}N_2\text{+}H]^+$ 242.9764, found 242.9762.

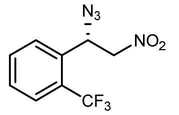

(*S*)-1-(1-Azido-2-nitroethyl)-2-(trifluoromethyl)benzene (2o). The reaction was carried out according to the General Procedure 2 to obtain a colorless oil (63% yield, NMR) that was determined to be 20% ee by chiral HPLC (Chiralcel IB, 2% IPA/hexanes, 1.0 mL/min: t_R(minor) = 8.2 min, t_R(major) = 12.5 min). The reaction was also carried out at 100 μmol scale to obtain the product (25 mg, 96%) with 33% ee. $[\alpha]_D^{20}$ +13.5 (*c* 1.00, $CHCl_3$, 33% ee); R_f = 0.3 (10% EtOAc/hexanes); IR (film) 3112, 3076, 2986, 2924, 2129, 1642, 1604, 1562, 1530, 1490, 1455, 1422 cm^{-1}; ^{1}H NMR (400 MHz, $CDCl_3$) δ 7.76 (d, *J* = 7.9 Hz, 1H), 7.73-7.63 (m, 2H), 7.55 (ddd, *J* = 7.5, 7.5, 1.5 Hz, 1H), 5.78 (dd, *J* = 9.7, 3.8 Hz, 1H), 4.51 (dd, *J* = 13.8, 9.7 Hz, 1H), 4.44 (dd, *J* = 13.8, 3.9 Hz, 1H); ^{13}C NMR (150 MHz, $CDCl_3$) ppm 133.3, 133.2, 129.8, 128.8, 128.3 (d, $^2J_{CF}$ = 31.2 Hz), 126.7 (q, $^3J_{CF}$ = 5.6 Hz), 123.9 (d, $^1J_{CF}$ = 274.2 Hz), 78.2, 58.4; ^{19}F NMR (282 MHz, $CDCl_3$) δ -58.3; HRMS (APCI): Exact mass calcd for $C_9H_8F_3N_2O_3$ $[M\text{-}N_2\text{+}H]^+$ 233.0523, found 233.0530. Applying aryl triflamide M3 with the same procedure gave 28% yield with 64% ee.

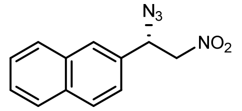

(*S*)-2-(1-Azido-2-nitroethyl)naphthalene (2p). The reaction was carried out according to the General Procedure 2 to obtain a light yellow solid (58% yield, NMR) that was determined to be 86% ee by chiral HPLC (Chiralcel IB, 30% IPA/hexanes, 1.0 mL/min: t_R(minor) = 18.2 min, t_R(major) = 53.6 min). The reaction was also carried out at 100 μmol scale to obtain the product (20 mg, 81%) with 84% ee. Mp 91-93 °C; $[\alpha]_D^{20}$ +91.3 (*c* 1.00, $CHCl_3$, 84% ee); R_f = 0.3 (10% EtOAc/hexanes); IR (film) 3108, 3059, 2964, 2920, 2120, 1632, 1557, 1508, 1420 cm^{-1}; ^{1}H NMR (400 MHz, $CDCl_3$) δ 7.94 (d, *J* = 5.7 Hz, 1H), 7.91-7.83 (m, 3H), 7.60-7.54 (m, 2H), 7.42 (dd, *J* = 5.7, 1.1 Hz, 1H), 5.53 (dd, *J* = 6.6, 2.9 Hz, 1H), 4.73 (dd, *J* = 9.0, 6.6 Hz, 1H), 4.55 (dd, *J* = 9.0, 2.9 Hz, 1H); ^{13}C NMR (150 MHz, $CDCl_3$) ppm 133.7, 133.2, 131.4, 129.7, 128.1, 127.9, 127.2, 127.1, 126.9, 123.6, 78.5, 62.8; HRMS (ESI): Exact mass calcd for $C_{12}H_9N_4O_2$ $[M\text{-}H]^-$ 241.0731, found 241.0720.

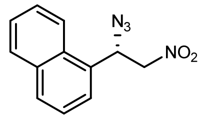

(*S*)-1-(1-Azido-2-nitroethyl)naphthalene (2q). The reaction was carried out according to the General Procedure 2 to obtain a light yellow oil (73% yield, NMR) that was determined to be 51% ee by chiral HPLC (Chiralcel IB, 30% IPA/hexanes, 1.0 mL/min: t_R(minor) = 11.4 min, t_R(major) = 25.8 min). The reaction was also carried out at 100 μmol scale to obtain the product (21 mg, 86%) with 46% ee. $[\alpha]_D^{20}$ +44.1 (*c* 1.00, $CHCl_3$); R_f = 0.3 (10% EtOAc/hexanes); IR (film) 3107, 3059, 2920, 2119, 1632, 1556, 1508 cm^{-1}; ^{1}H NMR (400 MHz, $CDCl_3$) δ 8.13 (d, *J* = 8.5 Hz, 1H), 7.97 (d, *J* = 8.9 Hz, 1H), 7.91 (d, *J* = 9.4 Hz, 1H), 7.71-7.63 (m, 2H), 7.63-7.50 (m, 2H), 6.12 (dd, *J* = 9.5, 4.1 Hz, 1H), 4.70 (dd, *J* = 13.6, 9.5 Hz, 1H), 4.64 (dd, *J* = 13.6, 4.1 Hz, 1H); ^{13}C NMR (150 MHz, $CDCl_3$) ppm 134.2, 130.4, 130.1, 129.9, 129.7, 127.7, 126.6, 125.6, 125.4, 122.0, 78.4, 60.1; HRMS (ESI): Exact mass calcd for $C_{12}H_9N_4O_2$ $[M\text{-}H]^-$ 241.0731, found 241.0718.

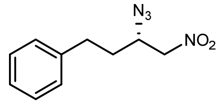

(*S*)-(3-Azido-4-nitrobutyl)benzene (2r). The reaction was carried out according to the General Procedure 2 to obtain a colorless oil (78% yield, NMR) that was determined to be 35% ee by chiral HPLC (Chiralcel IB, 20% IPA/hexanes, 1.0 mL/min: t_R(major) = 14.4 min, t_R(minor) = 16.8 min). $[\alpha]_D^{20}$ +23.3 (*c* 1.00, $CHCl_3$); R_f = 0.3 (10% EtOAc/hexanes); IR (film) 3063, 3028, 2926, 2130, 1556, 1454 cm^{-1}; ^{1}H NMR (400 MHz, $CDCl_3$) δ 7.36-7.28 (m, 2H), 7.23-7.17 (m, 3H), 4.37 (d, *J* = 6.3 Hz, 2H), 4.15-4.05 (m, 1H), 2.92-2.81 (m, 1H), 2.80-2.71 (m, 1H), 1.90 (ddd, *J* = 14.2, 6.5, 6.5 Hz, 2H); ^{13}C NMR (150 MHz, $CDCl_3$) ppm 139.7, 128.9, 128.5, 126.8, 77.8, 58.7, 33.6, 31.8; HRMS (APCI): Exact mass calcd for $C_{10}H_{13}N_2O_2$ $[M\text{-}N_2\text{+}H]^+$ 193.0972, found 193.0969.

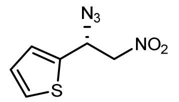

(*S*)-2-(1-Azido-2-nitroethyl)thiophene (2s). The reaction was carried out according to the General Procedure 2 to obtain a light green oil (17 mg, 88%) that was determined to be 60% ee by chiral HPLC (Chiralcel IB, 30% IPA/hexanes, 1.0 mL/min: t_R(minor) = 7.8 min, t_R(major) = 8.9 min). $[\alpha]_D^{20}$ +80.4 (*c* 1.00, $CHCl_3$); R_f = 0.3 (10% EtOAc/hexanes); IR (film) 3107, 3092, 2121,

1623, 1557, 1524, 1492 cm^{-1}; ^{1}H NMR (400 MHz, $CDCl_3$) δ 7.41 (dd, *J* = 5.0, 1.1 Hz, 1H), 7.16 (d, *J* = 3.8 Hz, 1H), 7.07 (dd, *J* = 5.1, 3.6 Hz, 1H), 5.59 (dd, *J* = 9.5, 4.7 Hz, 1H), 4.67 (dd, *J* = 13.4, 9.6 Hz, 1H), 4.56 (dd, *J* = 13.4, 4.7 Hz, 1H); ^{13}C NMR (150 MHz, $CDCl_3$) ppm 136.2, 135.5, 127.5, 127.2, 78.3, 57.9; HRMS (APCI): Exact mass calcd for $C_6H_7N_2O_2S$ $[M-N_2+H]^+$ 171.0223, found 171.0221.

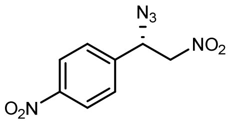

(*S*)-2-(1-Azido-2-nitroethyl)-4-nitrobenzene (2t). The reaction was carried out according to the General Procedure 5 to obtain a light-yellow solid (57% yield, NMR) that was determined to be 64% ee by chiral HPLC (Chiralcel IC, 10% IPA/hexanes, 1.0 mL/min: t_R(minor) = 22.6 min, t_R(major) = 27.7 min). The reaction was also carried out at 100 µmol scale to obtain the product (86% yield, NMR) with 64% ee. R_f = 0.2 (20% Et_2O/hexanes); ^{1}H NMR (400 MHz, $CDCl_3$) δ 7.94 (d, *J* = 5.7 Hz, 1H), 7.91-7.83 (m, 3H), 7.60-7.54 (m, 2H), 7.42 (dd, *J* = 5.7, 1.1 Hz, 1H), 5.53 (dd, *J* = 6.6, 2.9 Hz, 1H), 4.73 (dd, *J* = 9.0, 6.6 Hz, 1H), 4.55 (dd, *J* = 9.0, 2.9 Hz, 1H); Purification of the product by silica gel flash chromatography eluting 20% Et_2O/hexanes led to decomposition of the title compound. HRMS (ESI): Exact mass calcd for $C_8H_8N_3O_4$ $[M-H]^-$ 210.0509, found 210.0507.

---

[1]Pangborn, A. B., et al. *Organometallics* **1996**, *15*, 1518.

[2] Perrin, C. L., et al. *Anal. Chem.* **1996**, *68*, 2127.

[3] Hess, A. S., et al. *Synlett* **2006**, 147.

[4] Bordwell, F. G. *Acc. Chem. Res.* **1988**, *21*, 456. .

[5] Evans, R., et al. *Anal. Chem.* **2018**, *90*, 3987.

[6] Neese, F. *WIREs Computational Molecular Science* **2022**, *12*, e1606.

[7] Bannwarth, C., et al. *J. Chem. Theory Comput.* **2019**, *15*, 1652.

[8] Bursch, M., et al. *Angew. Chem. Int. Ed.* **2022**, *61*, e202205735.

[9] Ásgeirsson, V., et al. *J. Chem. Theory Comput.* **2021**, *17*, 4929.

[10] Gasevic, T., et al. *J. Phys. Chem. A* **2022**, *126*, 3826.

[11] Najibi, A., et al. *J. Comput. Chem.* **2020**, *41*, 2562.

[12] Maeda, S., et al. *Int. J. Quantum Chem.* **2015**, *115*, 258.

[13] a) Lu, T., et al. *J. Comput. Chem.* **2012**, *33*, 580. b) Lu, T. *J. Chem. Phys.* **2024**, *161*, 082503.

[14] Prepared from the published procedure: lig_1 to lig_5 were prepared using similar procedure :Payne, J. L., et al. *Chem. Sci.* **2022**, *13*, 7318.; lig_6: Vara, B. A., et al. *J. Org. Chem.* **2014**, *79*, 6913.

[15] Prepared from the published procedure: Vara, B. A., et al. *J. Org. Chem.* **2014**, *79*, 6913.

[16] Zhang, Z.-B., et al. *Organic Letters* **2020**, *22*, 9609.

[17] Xue, D., et al. Patent CN116554066, 2023.

[18] Qiu, D., et al. *Organic Letters* **2016**, *18*, 3130.

[19] Wang, Z., et al. *The Journal of Organic Chemistry* **2016**, *81*, 11195.

[20] Liang, B., et al. *European Journal of Organic Chemistry* **2021**, *2021*, 1466.

[21] Torti, E., et al. *Chemistry – A European Journal* **2016**, *22*, 16998.

[22] Thiam, A., et al. *Journal of Power Sources* **2017**, *364*, 138.

[23] Kögel, J. F., et al. *Chemistry – A European Journal* **2015**, *21*, 5769.

[24] Kofink, C. C., et al. *Chemical Communications* **2007**, 1954.

[25] Astakhova, V. V., et al. *ChemistrySelect* **2018**, *3*, 5960.

[26] Wang, X., et al. *Journal of the American Chemical Society* **2009**, *131*, 7520.

[27] Congreve, A., et al. *New Journal of Chemistry* **2003**, *27*, 98.

[28] Bosnidou, A. E., et al. *Angewandte Chemie International Edition* **2019**, *58*, 7485.

[29] Ochiai, M., et al. *Science* **2011**, *332*, 448.

[30] Li, B.-Y., et al. *Chemical Science* **2022**, *13*, 2270.

[31] Hulley, M. E., et al. *ChemBioChem* **2010**, *11*, 2433.

[32] Lee, J., et al. *Journal of Medicinal Chemistry* **2003**, *46*, 3116.

[33] Wang, Y., et al. *Chemical Science* **2017**, *8*, 3852.

[34] Hasegawa, A., et al. *Bull. Chem. Soc. Jpn.* **2005**, *78*, 1401.

[35] Bellavista, T., et al. *Adv. Synth. Catal.* **2015**, *357*, 3365.
