# Supplementary material for: Performance-Enhancing Asymmetric Catalysis Driven by Achiral Counterion Design
Source: J Am Chem Soc. 2025 May 14;147(21):17584–91. doi: 10.1021/jacs.5c05263 (PMC12123621; doi:10.1021/jacs.5c05263)
Supplement: Supplementary file 2 [file ja5c05263_si_002.pdf]

Performance Enhancing Asymmetric Catalysis Driven by Achiral Counterion Design

Zihang Deng, Jenna L. Payne, Mahesh Vishe, Julius E. L. Jan, Cody M. Funk, Jeffrey N. Johnston*

Department of Chemistry and Vanderbilt Institute of Chemical Biology,

Vanderbilt University, Nashville, Tennessee 37235

Table of Contents SI2-X

Figure 1. ^{1}H NMR (400 MHz, $CDCl_3$) of S1 10
Figure 2. ^{13}C NMR (150 MHz, $CDCl_3$) of S1 11
Figure 3. ^{1}H NMR (400 MHz, $CDCl_3$) of Lig_7 12
Figure 4. ^{13}C NMR (150 MHz, $CDCl_3$) of Lig_7 13
Figure 5. ^{1}H NMR (400 MHz, $CDCl_3$) of Lig_8 14
Figure 6. ^{13}C NMR (150 MHz, $CDCl_3$) of Lig_8 15
Figure 7. ^{1}H NMR (400 MHz, $CDCl_3$) of A2 16
Figure 8. ^{13}C NMR (150 MHz, $CDCl_3$) of A2 17
Figure 9. ^{19}F NMR (282 MHz, $CDCl_3$) of A2 18
Figure 10. ^{1}H NMR (400 MHz, $CDCl_3$) of A3 19
Figure 11. ^{13}C NMR (150 MHz, $CDCl_3$) of A3 20
Figure 12. ^{19}F NMR (282 MHz, $CDCl_3$) of A3 21
Figure 13. ^{1}H NMR (400 MHz, $CDCl_3$) of A5 22
Figure 14. ^{13}C NMR (150 MHz, $CDCl_3$) of A5 23
Figure 15. ^{19}F NMR (282 MHz, $CDCl_3$) of A5 24
Figure 16. ^{1}H NMR (400 MHz, $CDCl_3$) of A6 25
Figure 17. ^{13}C NMR (150 MHz, $CDCl_3$) of A6 26
Figure 18. ^{19}F NMR (282 MHz, $CDCl_3$) of A6 27
Figure 19. ^{1}H NMR (400 MHz, $CDCl_3$) of A8 28
Figure 20. ^{13}C NMR (150 MHz, $CDCl_3$) of A8 29
Figure 21. ^{19}F NMR (282 MHz, $CDCl_3$) of A8 30
Figure 22. ^{1}H NMR (400 MHz, $CDCl_3$) of B4 31
Figure 23. ^{13}C NMR (150 MHz, $CDCl_3$) of B4 32
Figure 24. ^{19}F NMR (282 MHz, $CDCl_3$) of B4 33
Figure 25. ^{1}H NMR (400 MHz, $CDCl_3$) of B5 34
Figure 26. ^{13}C NMR (150 MHz, $CDCl_3$) of B5 35
Figure 27. ^{19}F NMR (282 MHz, $CDCl_3$) of B5 36
Figure 28. ^{1}H NMR (400 MHz, $CDCl_3$) of B6 37

Figure 29. ^{13}C NMR (150 MHz, $CDCl_3$) of B6 38
Figure 30. ^{19}F NMR (282 MHz, $CDCl_3$) of B6 39
Figure 31. ^{1}H NMR (400 MHz, $CDCl_3$) of C5 40
Figure 32. ^{13}C NMR (150 MHz, $CDCl_3$) of C5 41
Figure 33. ^{19}F NMR (282 MHz, $CDCl_3$) of C5 42
Figure 34. ^{1}H NMR (400 MHz, $CDCl_3$) of C6 43
Figure 35. ^{13}C NMR (150 MHz, acetone-d_6) of C6 44
Figure 36. ^{19}F NMR (282 MHz, $CDCl_3$) of C6 45
Figure 37. ^{1}H NMR (400 MHz, $CDCl_3$) of D4 46
Figure 38. ^{13}C NMR (150 MHz, $CDCl_3$) of D4 47
Figure 39. ^{19}F NMR (282 MHz, $CDCl_3$) of D4 48
Figure 40. ^{1}H NMR (400 MHz, $CDCl_3$) of D5 49
Figure 41. ^{13}C NMR (150 MHz, $CDCl_3$) of D5 50
Figure 42. ^{19}F NMR (282 MHz, $CDCl_3$) of D5 51
Figure 43. ^{1}H NMR (400 MHz, $CDCl_3$) of D6 52
Figure 44. ^{13}C NMR (150 MHz, $CDCl_3$) of D6 53
Figure 45. ^{19}F NMR (282 MHz, $CDCl_3$) of D6 54
Figure 46. ^{1}H NMR (400 MHz, $CDCl_3$) of D7 55
Figure 47. ^{13}C NMR (150 MHz, $CDCl_3$) of D7 56
Figure 48. ^{19}F NMR (282 MHz, $CDCl_3$) of D7 57
Figure 49. ^{1}H NMR (400 MHz, $CDCl_3$) of D8 58
Figure 50. ^{13}C NMR (150 MHz, $CDCl_3$) of D8 59
Figure 51. ^{19}F NMR (282 MHz, $CDCl_3$) of D8 60
Figure 52. ^{1}H NMR (400 MHz, $CDCl_3$) of E1 61
Figure 53. ^{13}C NMR (150 MHz, $CDCl_3$) of E1 62
Figure 54. ^{19}F NMR (282 MHz, $CDCl_3$) of E1 63
Figure 55. ^{1}H NMR (400 MHz, acetone-d_6) of E2 64
Figure 56. ^{13}C NMR (150 MHz, acetone-d_6) of E2 65
Figure 57. ^{19}F NMR (282 MHz, acetone-d_6) of E2 66
Figure 58. ^{1}H NMR (400 MHz, $CDCl_3$) of E3 67
Figure 59. ^{13}C NMR (150 MHz, $CDCl_3$) of E3 68
Figure 60. ^{19}F NMR (282 MHz, $CDCl_3$) of E3 69
Figure 61. ^{1}H NMR (400 MHz, acetone-d_6) of E4 70
Figure 62. ^{13}C NMR (150 MHz, $CDCl_3$) of E4 71

Figure 63. ^{19}F NMR (282 MHz, acetone-d_6) of E4 72
Figure 64. ^{1}H NMR (400 MHz, $CDCl_3$) of E5 73
Figure 65. ^{13}C NMR (150 MHz, $CDCl_3$) of E5 74
Figure 66. ^{19}F NMR (282 MHz, $CDCl_3$) of E5 75
Figure 67. ^{1}H NMR (400 MHz, acetone-d_6) of E6 76
Figure 68. ^{13}C NMR (150 MHz, acetone-d_6) of E6 77
Figure 69. ^{19}F NMR (282 MHz, acetone-d_6) of E6 78
Figure 70. ^{1}H NMR (400 MHz, $CDCl_3$) of E7 79
Figure 71. ^{13}C NMR (150 MHz, $CDCl_3$) of E7 80
Figure 72. ^{19}F NMR (282 MHz, $CDCl_3$) of E7 81
Figure 73. ^{1}H NMR (400 MHz, $CDCl_3$) of E8 82
Figure 74. ^{13}C NMR (150 MHz, $CDCl_3$) of E8 83
Figure 75. ^{19}F NMR (282 MHz, $CDCl_3$) of E8 84
Figure 76. ^{1}H NMR (400 MHz, $CDCl_3$) of F2 85
Figure 77. ^{13}C NMR (150 MHz, $CDCl_3$) of F2 86
Figure 78. ^{19}F NMR (282 MHz, $CDCl_3$) of F2 87
Figure 79. ^{1}H NMR (400 MHz, acetone-d_6) of F6 88
Figure 80. ^{13}C NMR (150 MHz, acetone-d_6) of F6 89
Figure 81. ^{19}F NMR (282 MHz, acetone-d_6) of F6 90
Figure 82. ^{1}H NMR (400 MHz, $CDCl_3$) of F7 91
Figure 83. ^{13}C NMR (150 MHz, acetone-d_6) of F7 92
Figure 84. ^{19}F NMR (282 MHz, $CDCl_3$) of F7 93
Figure 85. ^{1}H NMR (400 MHz, $CDCl_3$) of F8 94
Figure 86. ^{13}C NMR (150 MHz, acetone-d_6) of F8 95
Figure 87. ^{19}F NMR (282 MHz, $CDCl_3$) of F8 96
Figure 88. ^{1}H NMR (400 MHz, acetone-d_6) of G1 97
Figure 89. ^{13}C NMR (150 MHz, acetone-d_6) of G1 98
Figure 90. ^{19}F NMR (282 MHz, acetone-d_6) of G1 99
Figure 91. ^{1}H NMR (400 MHz, acetone-d_6) of G2 100
Figure 92. ^{13}C NMR (150 MHz, acetone-d_6) of G2 101
Figure 93. ^{19}F NMR (282 MHz, acetone-d_6) of G2 102
Figure 94. ^{1}H NMR (400 MHz, $CDCl_3$) of G3 103
Figure 95. ^{13}C NMR (150 MHz, acetone-d_6) of G3 104
Figure 96. ^{19}F NMR (282 MHz, $CDCl_3$) of G3 105

Figure 97. ^{1}H NMR (400 MHz, acetone-d_6) of G4 106
Figure 98. ^{13}C NMR (150 MHz, acetone-d_6) of G4 107
Figure 99. ^{19}F NMR (282 MHz, acetone-d_6) of G4 108
Figure 100. ^{1}H NMR (400 MHz, acetone-d_6) of G5 109
Figure 101. ^{13}C NMR (150 MHz, acetone-d_6) of G5 110
Figure 102. ^{19}F NMR (282 MHz, acetone-d_6) of G5 111
Figure 103. ^{1}H NMR (400 MHz, $CDCl_3$) of G7 112
Figure 104. ^{13}C NMR (150 MHz, $CDCl_3$) of G7 113
Figure 105. ^{19}F NMR (282 MHz, $CDCl_3$) of G7 114
Figure 106. ^{1}H NMR (400 MHz, $CDCl_3$) of G8 115
Figure 107. ^{13}C NMR (150 MHz, acetone-d_6) of G8 116
Figure 108. ^{19}F NMR (282 MHz, $CDCl_3$) of G8 117
Figure 109. ^{1}H NMR (400 MHz, $CDCl_3$) of H1 118
Figure 110. ^{13}C NMR (150 MHz, acetone-d_6) of H1 119
Figure 111. ^{19}F NMR (282 MHz, $CDCl_3$) of H1 120
Figure 112. ^{1}H NMR (400 MHz, acetone-d_6) of H2 121
Figure 113. ^{13}C NMR (150 MHz, acetone-d_6) of H2 122
Figure 114. ^{19}F NMR (282 MHz, acetone-d_6) of H2 123
Figure 115. ^{1}H NMR (400 MHz, acetone-d_6) of H3 124
Figure 116. ^{13}C NMR (150 MHz, acetone-d_6) of H3 125
Figure 117. ^{19}F NMR (282 MHz, acetone-d_6) of H3 126
Figure 118. ^{1}H NMR (400 MHz, $CDCl_3$) of H4 127
Figure 119. ^{13}C NMR (150 MHz, $CDCl_3$) of H4 128
Figure 120. ^{19}F NMR (282 MHz, $CDCl_3$) of H4 129
Figure 121. ^{1}H NMR (400 MHz, $CDCl_3$) of H5 130
Figure 122. ^{13}C NMR (150 MHz, acetone-d_6) of H5 131
Figure 123. ^{19}F NMR (282 MHz, acetone-d_6) of H5 132
Figure 124. ^{1}H NMR (400 MHz, $CDCl_3$) of H6 133
Figure 125. ^{13}C NMR (150 MHz, acetone-d_6) of H6 134
Figure 126. ^{19}F NMR (282 MHz, acetone-d_6) of H6 135
Figure 127. ^{1}H NMR (400 MHz, acetone-d_6) of H7 136
Figure 128. ^{13}C NMR (150 MHz, acetone-d_6) of H7 137
Figure 129. ^{19}F NMR (282 MHz, acetone-d_6) of H7 138
Figure 130. ^{1}H NMR (400 MHz, acetone-d_6) of H8 139

Figure 131. ^{13}C NMR (150 MHz, acetone-d_6) of H8 140
Figure 132. ^{19}F NMR (282 MHz, acetone-d_6) of H8 141
Figure 133. ^{1}H NMR (400 MHz, acetone-d_6) of I3 142
Figure 134. ^{13}C NMR (150 MHz, acetone-d_6) of I3 143
Figure 135. ^{19}F NMR (282 MHz, acetone-d_6) of I3 144
Figure 136. ^{1}H NMR (400 MHz, acetone-d_6) of I4 145
Figure 137. ^{13}C NMR (150 MHz, acetone-d_6) of I4 146
Figure 138. ^{19}F NMR (282 MHz, acetone-d_6) of I4 147
Figure 139. ^{1}H NMR (400 MHz, acetone-d_6) of I6 148
Figure 140. ^{13}C NMR (150 MHz, acetone-d_6) of I6 149
Figure 141. ^{19}F NMR (282 MHz, acetone-d_6) of I6 150
Figure 142. ^{1}H NMR (400 MHz, acetone-d_6) of I8 151
Figure 143. ^{13}C NMR (150 MHz, acetone-d_6) of I8 152
Figure 144. ^{19}F NMR (282 MHz, acetone-d_6) of I8 153
Figure 145. ^{1}H NMR (400 MHz, acetone-d_6) of J1 154
Figure 146. ^{13}C NMR (150 MHz, acetone-d_6) of J1 155
Figure 147. ^{19}F NMR (282 MHz, acetone-d_6) of J1 156
Figure 148. ^{1}H NMR (400 MHz, acetone-d_6) of J2 157
Figure 149. ^{13}C NMR (150 MHz, acetone-d_6) of J2 158
Figure 150. ^{19}F NMR (282 MHz, acetone-d_6) of J2 159
Figure 151. ^{1}H NMR (400 MHz, $CDCl_3$) of J4 160
Figure 152. ^{13}C NMR (150 MHz, $CDCl_3$) of J4 161
Figure 153. ^{19}F NMR (282 MHz, $CDCl_3$) of J4 162
Figure 154. ^{1}H NMR (400 MHz, acetone-d_6) of J5 163
Figure 155. ^{13}C NMR (150 MHz, acetone-d_6) of J5 164
Figure 156. ^{19}F NMR (282 MHz, acetone-d_6) of J5 165
Figure 157. ^{1}H NMR (400 MHz, $CDCl_3$) of J6 166
Figure 158. ^{13}C NMR (150 MHz, $CDCl_3$) of J6 167
Figure 159. ^{19}F NMR (282 MHz, $CDCl_3$) of J6 168
Figure 160. ^{1}H NMR (400 MHz, $CDCl_3$) of J7 169
Figure 161. ^{13}C NMR (150 MHz, $CDCl_3$) of J7 170
Figure 162. ^{19}F NMR (282 MHz, $CDCl_3$) of J7 171
Figure 163. ^{1}H NMR (400 MHz, acetone-d_6) of J8 172
Figure 164. ^{13}C NMR (150 MHz, acetone-d_6) of J8 173

Figure 165. ^{19}F NMR (282 MHz, acetone-d_6) of J8 174
Figure 166. ^{1}H NMR (400 MHz, acetone-d_6) of K1 175
Figure 167. ^{13}C NMR (150 MHz, acetone-d_6) of K1 176
Figure 168. ^{19}F NMR (282 MHz, acetone-d_6) of K1 177
Figure 169. ^{1}H NMR (400 MHz, acetone-d_6) of K2 178
Figure 170. ^{13}C NMR (150 MHz, acetone-d_6) of K2 179
Figure 171. ^{19}F NMR (282 MHz, acetone-d_6) of K2 180
Figure 172. ^{1}H NMR (400 MHz, acetone-d_6) of K3 181
Figure 173. ^{13}C NMR (150 MHz, acetone-d_6) of K3 182
Figure 174. ^{19}F NMR (282 MHz, acetone-d_6) of K3 183
Figure 175. ^{1}H NMR (400 MHz, $CDCl_3$) of K4 184
Figure 176. ^{13}C NMR (150 MHz, acetone-d_6) of K4 185
Figure 177. ^{19}F NMR (282 MHz, $CDCl_3$) of K4 186
Figure 178. ^{1}H NMR (400 MHz, acetone-d_6) of K5 187
Figure 179. ^{13}C NMR (150 MHz, acetone-d_6) of K5 188
Figure 180. ^{19}F NMR (282 MHz, acetone-d_6) of K5 189
Figure 181. ^{1}H NMR (400 MHz, acetone-d_6) of K6 190
Figure 182. ^{13}C NMR (150 MHz, acetone-d_6) of K6 191
Figure 183. ^{19}F NMR (282 MHz, acetone-d_6) of K6 192
Figure 184. ^{1}H NMR (400 MHz, acetone-d_6) of K7 193
Figure 185. ^{13}C NMR (150 MHz, acetone-d_6) of K7 194
Figure 186. ^{19}F NMR (282 MHz, acetone-d_6) of K7 195
Figure 187. ^{1}H NMR (400 MHz, acetone-d_6) of K8 196
Figure 188. ^{13}C NMR (150 MHz, acetone-d_6) of K8 197
Figure 189. ^{19}F NMR (282 MHz, acetone-d_6) of K8 198
Figure 190. ^{1}H NMR (400 MHz, $CDCl_3$) of L2 199
Figure 191. ^{13}C NMR (150 MHz, $CDCl_3$) of L2 200
Figure 192. ^{19}F NMR (282 MHz, $CDCl_3$) of L2 201
Figure 193. ^{1}H NMR (400 MHz, $CDCl_3$) of L3 202
Figure 194. ^{13}C NMR (150 MHz, $CDCl_3$) of L3 203
Figure 195. ^{19}F NMR (282 MHz, $CDCl_3$) of L3 204
Figure 196. ^{1}H NMR (400 MHz, acetone-d_6) of L4 205
Figure 197. ^{13}C NMR (150 MHz, acetone-d_6) of L4 206
Figure 198. ^{19}F NMR (282 MHz, acetone-d_6) of L4 207

Figure 199. ^{1}H NMR (400 MHz, $CDCl_3$) of L7 208
Figure 200. ^{13}C NMR (150 MHz, acetone-d_6) of L7 209
Figure 201. ^{19}F NMR (282 MHz, $CDCl_3$) of L7 210
Figure 202. ^{1}H NMR (400 MHz, CD_3OD) of L8 211
Figure 203. ^{13}C NMR (150 MHz, acetone-d_6) of L8 212
Figure 204. ^{19}F NMR (282 MHz, CD_3OD) of L8 213
Figure 205. ^{1}H NMR (400 MHz, acetone-d_6) of M1 214
Figure 206. ^{13}C NMR (150 MHz, acetone-d_6) of M1 215
Figure 207. ^{19}F NMR (282 MHz, acetone-d_6) of M1 216
Figure 208. ^{1}H NMR (400 MHz, acetone-d_6) of M2 217
Figure 209. ^{13}C NMR (150 MHz, acetone-d_6) of M2 218
Figure 210. ^{19}F NMR (282 MHz, acetone-d_6) of M2 219
Figure 211. ^{1}H NMR (400 MHz, acetone-d_6) of M3 220
Figure 212. ^{13}C NMR (150 MHz, acetone-d_6) of M3 221
Figure 213. ^{19}F NMR (282 MHz, acetone-d_6) of M3 222
Figure 214. ^{1}H NMR (400 MHz, acetone-d_6) of M4 223
Figure 215. ^{13}C NMR (150 MHz, acetone-d_6) of M4 224
Figure 216. ^{19}F NMR (282 MHz, acetone-d_6) of M4 225
Figure 217. ^{1}H NMR (400 MHz, acetone-d_6) of M5 226
Figure 218. ^{13}C NMR (150 MHz, acetone-d_6) of M5 227
Figure 219. ^{19}F NMR (282 MHz, acetone-d_6) of M5 228
Figure 220. ^{1}H NMR (400 MHz, $CDCl_3$) of M7 229
Figure 221. ^{13}C NMR (150 MHz, acetone-d_6) of M7 230
Figure 222. ^{19}F NMR (282 MHz, $CDCl_3$) of M7 231
Figure 223. ^{1}H NMR (400 MHz, $CDCl_3$) of M8 232
Figure 224. ^{13}C NMR (150 MHz, acetone-d_6) of M8 233
Figure 225. ^{19}F NMR (282 MHz, $CDCl_3$) of M8 234
Figure 226. ^{1}H NMR (400 MHz, $CDCl_3$) of 2b 235
Figure 227. ^{13}C NMR (150 MHz, $CDCl_3$) of 2b 236
Figure 228. ^{1}H NMR (400 MHz, $CDCl_3$) of 2c 237
Figure 229. ^{13}C NMR (150 MHz, $CDCl_3$) of 2c 238
Figure 230. ^{1}H NMR (400 MHz, $CDCl_3$) of 2d 239
Figure 231. ^{13}C NMR (150 MHz, $CDCl_3$) of 2d 240
Figure 232. ^{1}H NMR (400 MHz, $CDCl_3$) of 2e 241

Figure 233. ^{13}C NMR (150 MHz, $CDCl_3$) of 2e 242
Figure 234. ^{1}H NMR (400 MHz, $CDCl_3$) of 2f........ 243
Figure 235. ^{13}C NMR (150 MHz, $CDCl_3$) of 2f 244
Figure 236. ^{1}H NMR (400 MHz, $CDCl_3$) of 2g........ 245
Figure 237. ^{13}C NMR (150 MHz, $CDCl_3$) of 2g........ 246
Figure 238. ^{1}H NMR (400 MHz, $CDCl_3$) of 2h........ 247
Figure 239. ^{13}C NMR (150 MHz, $CDCl_3$) of 2h........ 248
Figure 241. ^{1}H NMR (400 MHz, $CDCl_3$) of 2i........ 250
Figure 242. ^{13}C NMR (150 MHz, $CDCl_3$) of 2i........ 251
Figure 243. ^{1}H NMR (400 MHz, $CDCl_3$) of 2j........ 252
Figure 244. ^{13}C NMR (150 MHz, $CDCl_3$) of 2j........ 253
Figure 246. ^{1}H NMR (400 MHz, $CDCl_3$) of 2k........ 255
Figure 247. ^{13}C NMR (150 MHz, $CDCl_3$) of 2k........ 256
Figure 248. ^{1}H NMR (400 MHz, $CDCl_3$) of 2l........ 257
Figure 249. ^{13}C NMR (150 MHz, $CDCl_3$) of 2l........ 258
Figure 250. ^{1}H NMR (400 MHz, $CDCl_3$) of 2m........ 259
Figure 251. ^{13}C NMR (150 MHz, $CDCl_3$) of 2m........ 260
Figure 252. ^{1}H NMR (400 MHz, $CDCl_3$) of 2n........ 261
Figure 253. ^{13}C NMR (150 MHz, $CDCl_3$) of 2n........ 262
Figure 254. ^{1}H NMR (400 MHz, $CDCl_3$) of 2o........ 263
Figure 255. ^{13}C NMR (150 MHz, $CDCl_3$) of 2o........ 264
Figure 257. ^{1}H NMR (400 MHz, $CDCl_3$) of 2p........ 266
Figure 258. ^{13}C NMR (150 MHz, $CDCl_3$) of 2p........ 267
Figure 259. ^{1}H NMR (400 MHz, $CDCl_3$) of 2q........ 268
Figure 260. ^{13}C NMR (150 MHz, $CDCl_3$) of 2q........ 269
Figure 261. ^{1}H NMR (400 MHz, $CDCl_3$) of 2r........ 270
Figure 262. ^{13}C NMR (150 MHz, $CDCl_3$) of 2r 271
Figure 263. ^{1}H NMR (400 MHz, $CDCl_3$) of 2t........ 272
Figure 264. ^{13}C NMR (150 MHz, $CDCl_3$) of 2t........ 273
Figure 265. HPLC trace of 2a 274
Figure 266. HPLC trace of 2b........ 275
Figure 267. HPLC trace of 2c 276
Figure 268. HPLC trace of 2d........ 277
Figure 269. HPLC trace of 2e 278

Figure 270. HPLC trace of 2f 279
Figure 271. HPLC trace of 2g 280
Figure 272. HPLC trace of 2h 281
Figure 273. HPLC trace of 2i 282
Figure 274. HPLC trace of 2j 283
Figure 275. HPLC trace of 2k 284
Figure 276. HPLC trace of 2l 285
Figure 277. HPLC trace of 2m 286
Figure 278. HPLC trace of 2n 287
Figure 279. HPLC trace of 2o 288
Figure 280. HPLC trace of 2p 289
Figure 281. HPLC trace of 2q 290
Figure 282. HPLC trace of 2r 291
Figure 283. HPLC trace of 2s 292
Figure 284. HPLC trace of 2t 293

Figure 1. ^{1}H NMR (400 MHz, $CDCl_3$) of **S1**

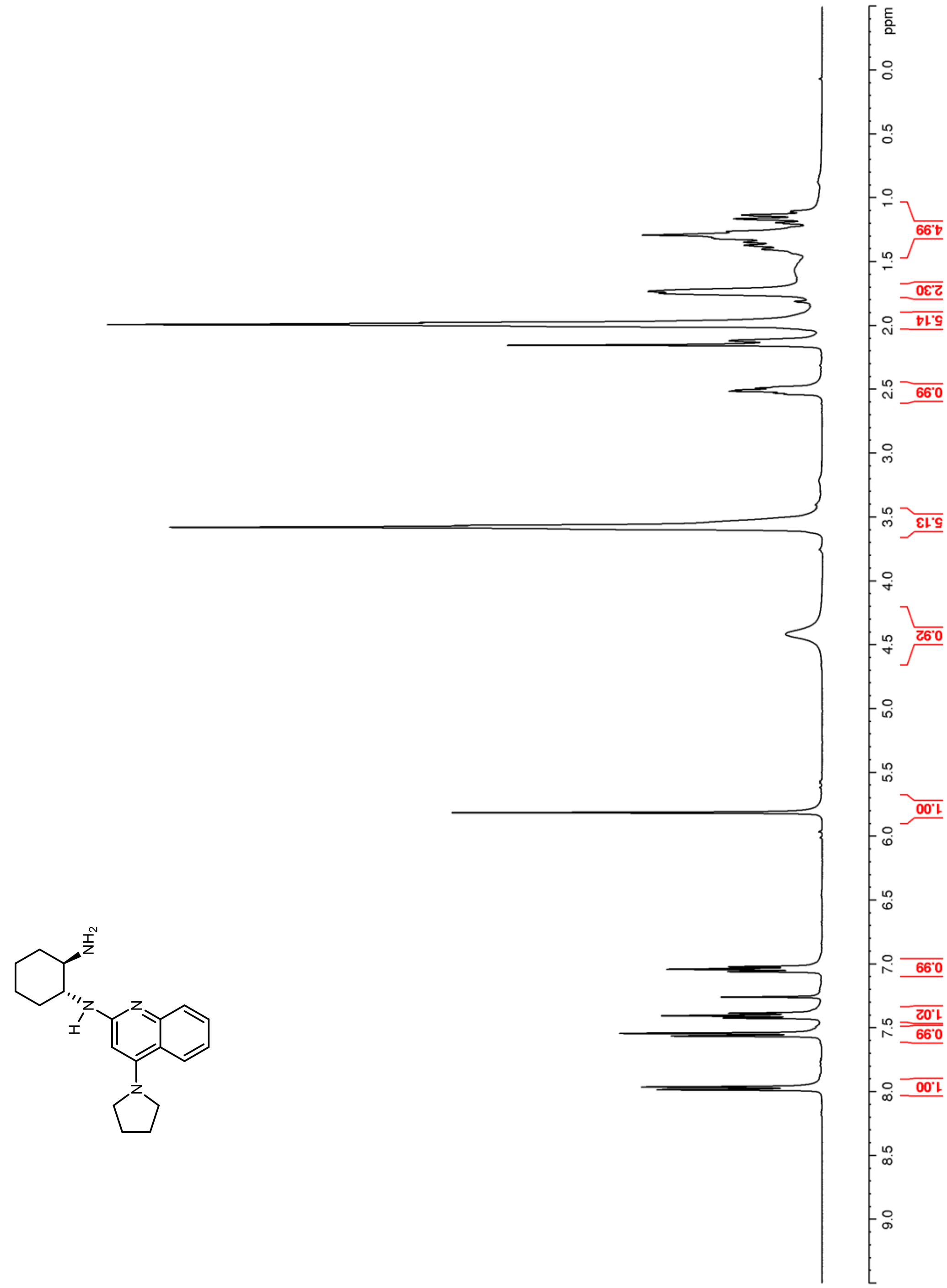

Figure 2. ^{13}C NMR (150 MHz, $CDCl_3$) of **S1**

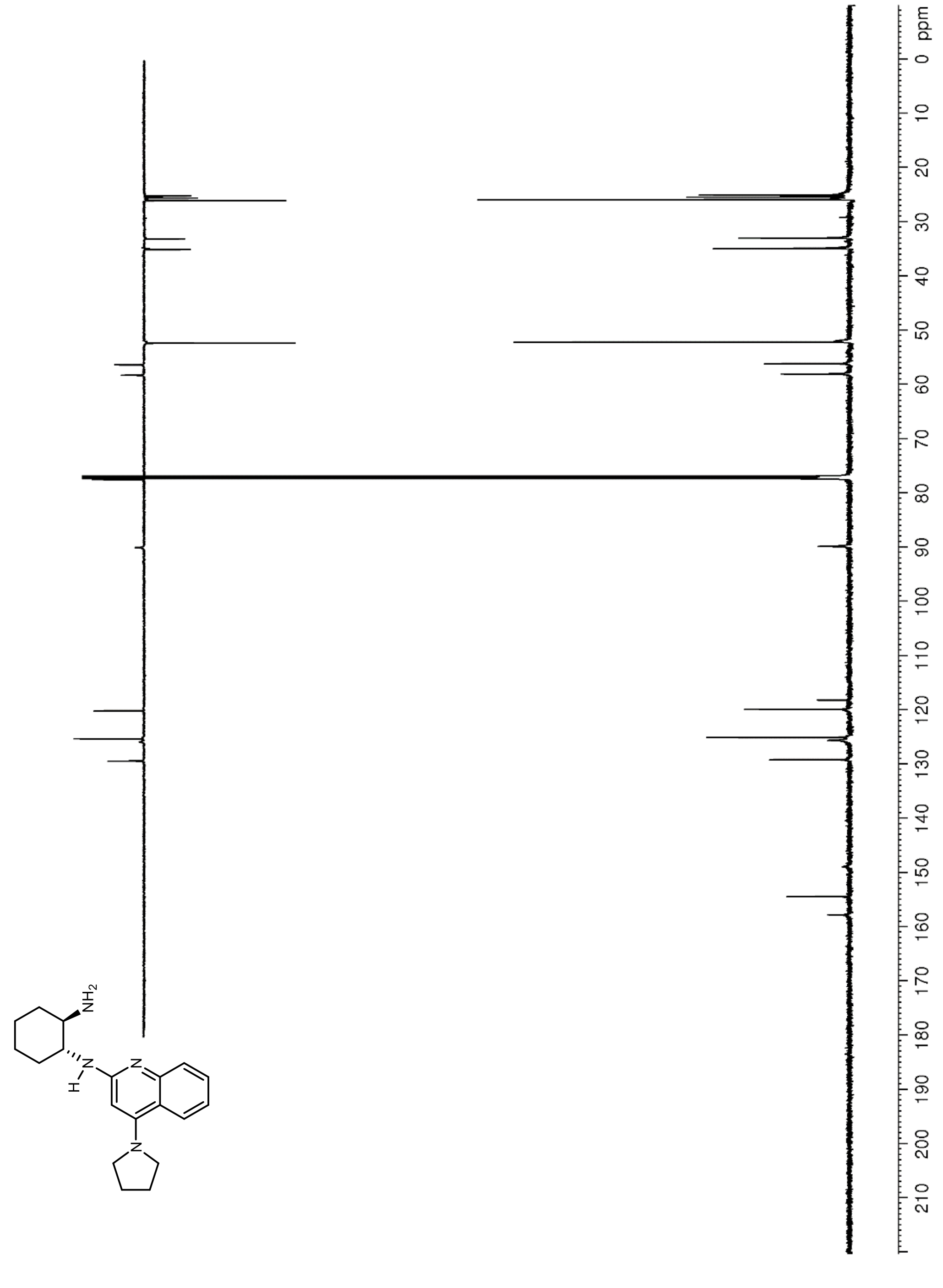

Figure 3. ^{1}H NMR (400 MHz, $CDCl_3$) of **Lig7**

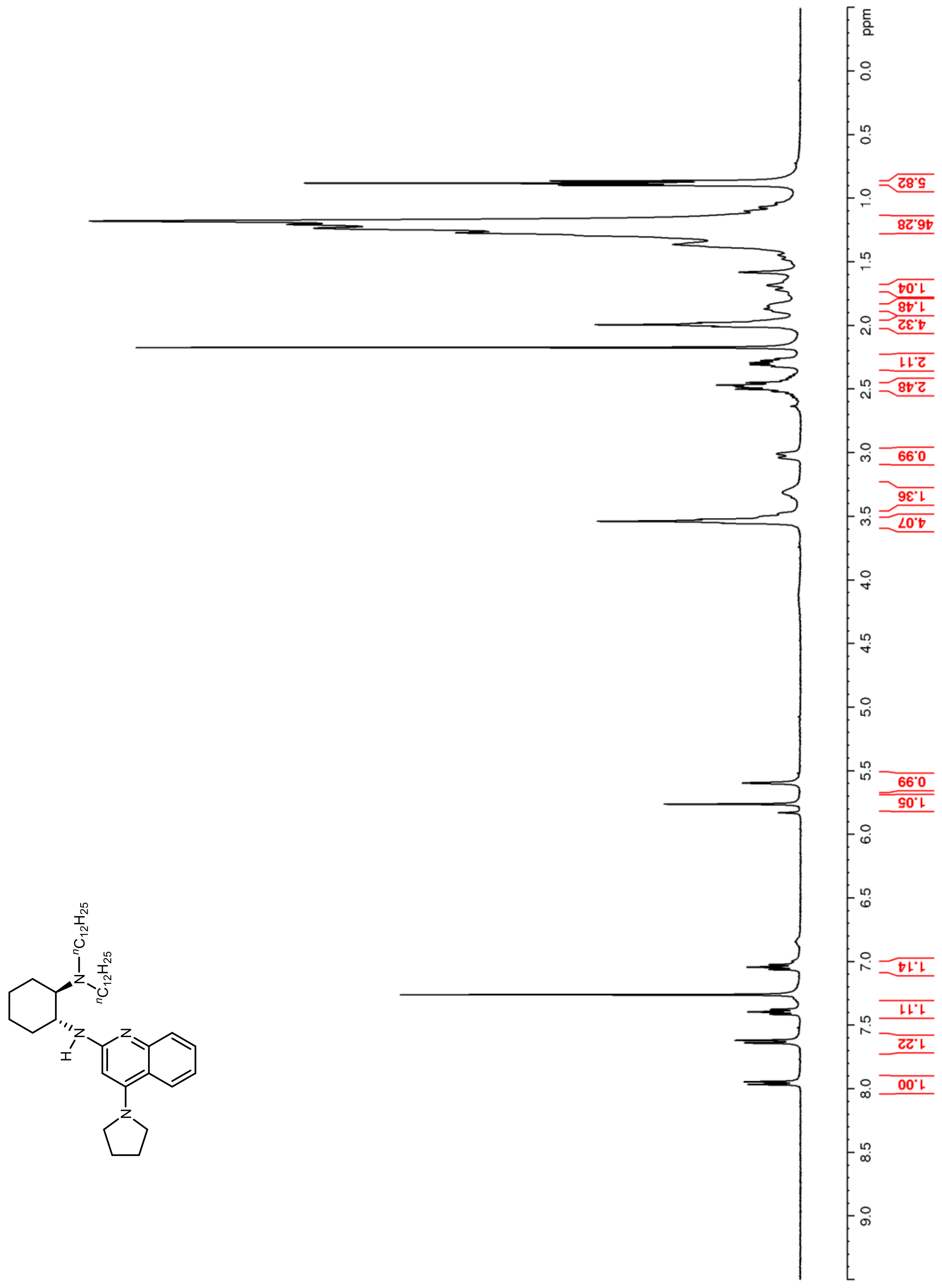

Figure 4. ^{13}C NMR (150 MHz, $CDCl_3$) of **Lig7**

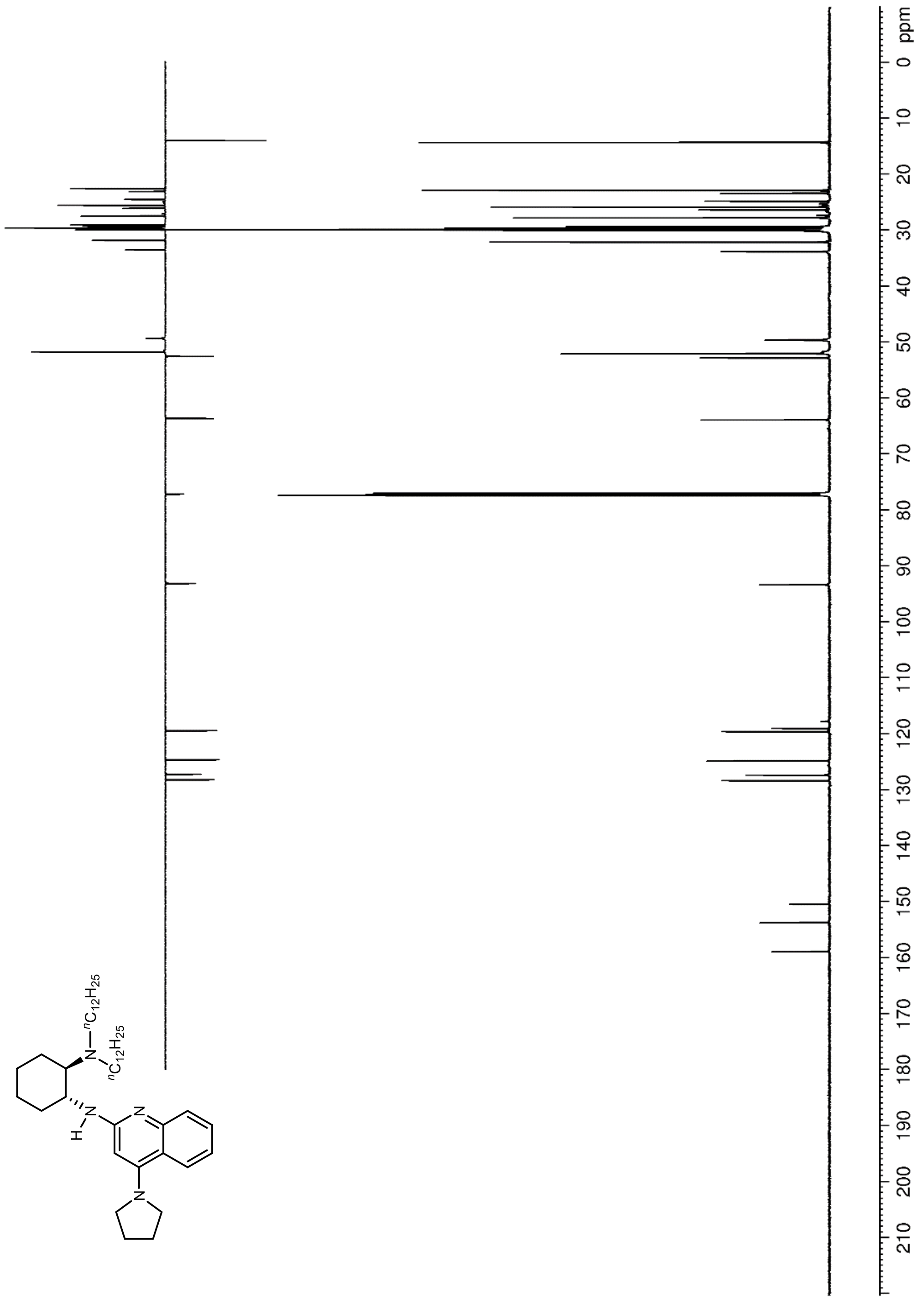

Figure 5. ^{1}H NMR (400 MHz, $CDCl_3$) of **Lig8**

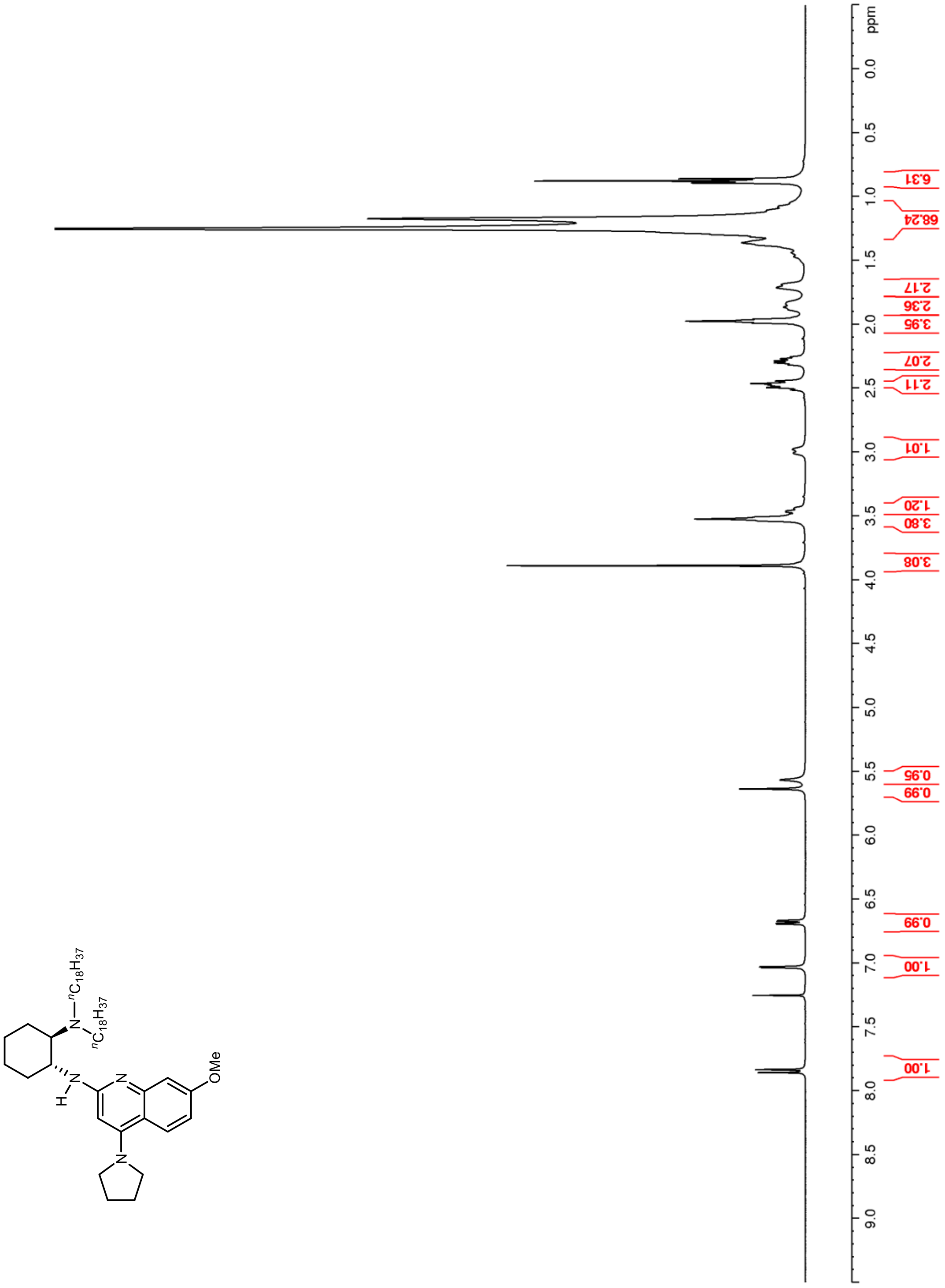

Figure 6. ^{13}C NMR (150 MHz, $CDCl_3$) of **Lig8**

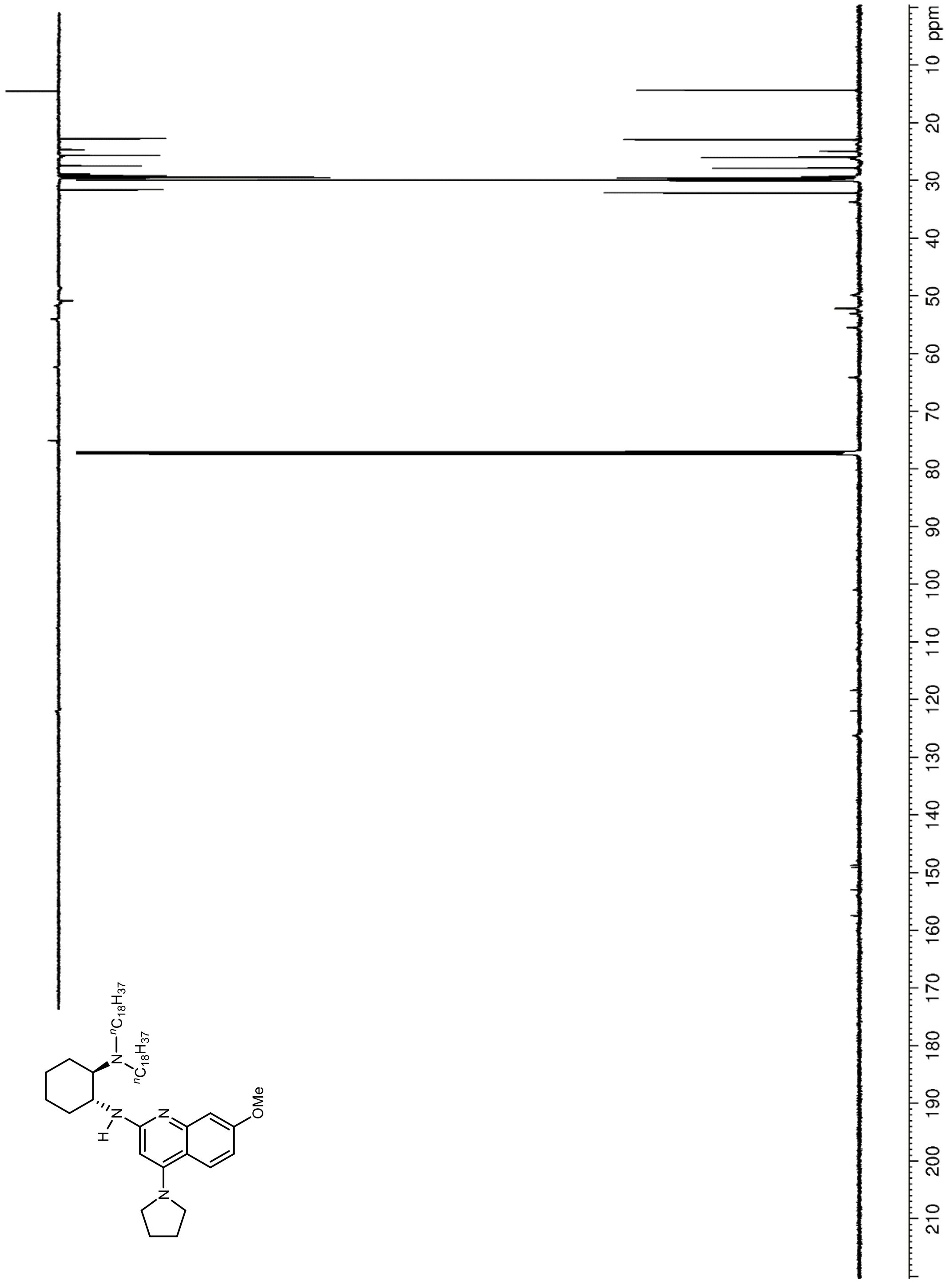

Figure 7. ^{1}H NMR (400 MHz, $CDCl_3$) of **A2**

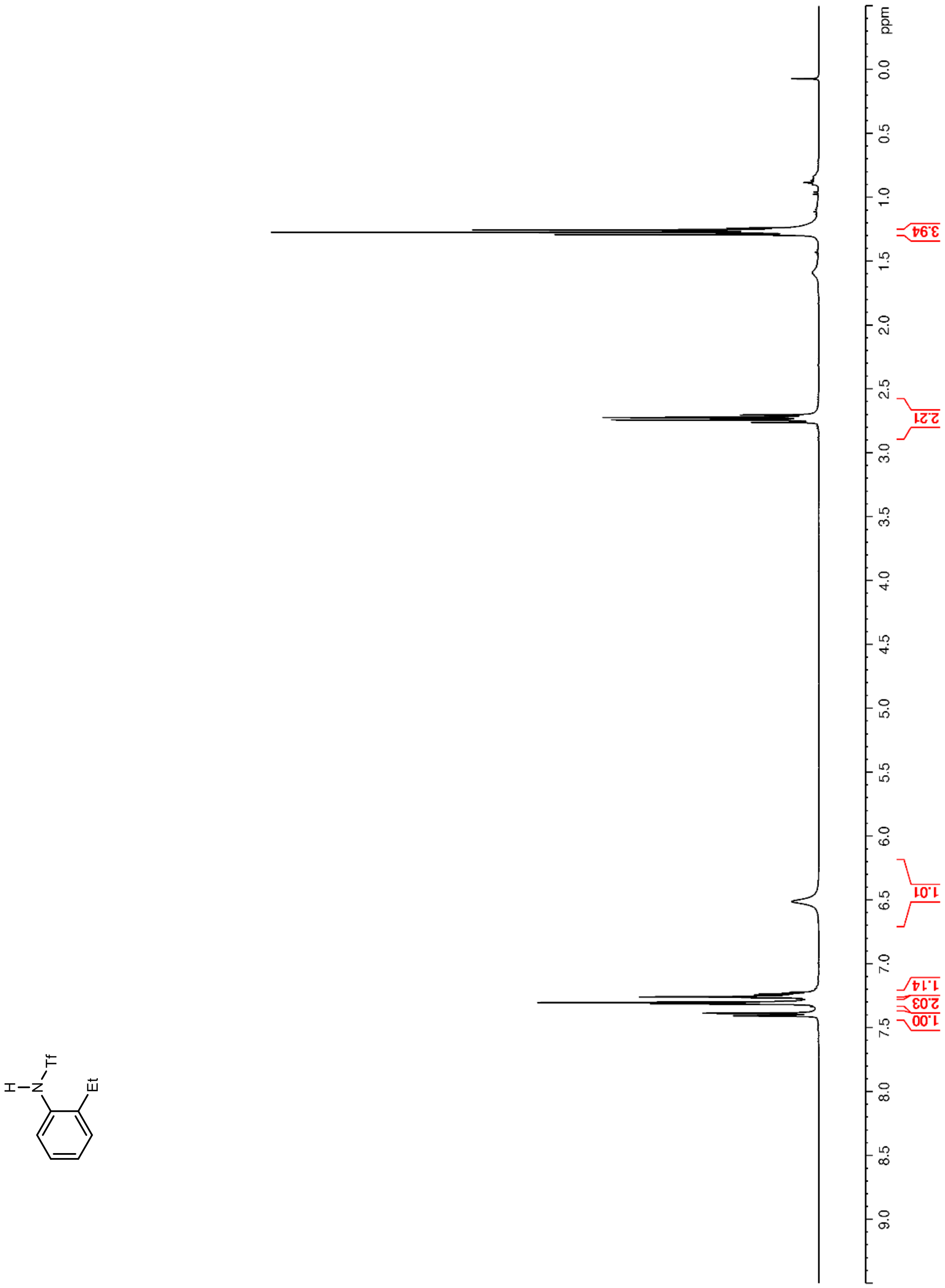

Figure 8. ^{13}C NMR (150 MHz, $CDCl_3$) of **A2**

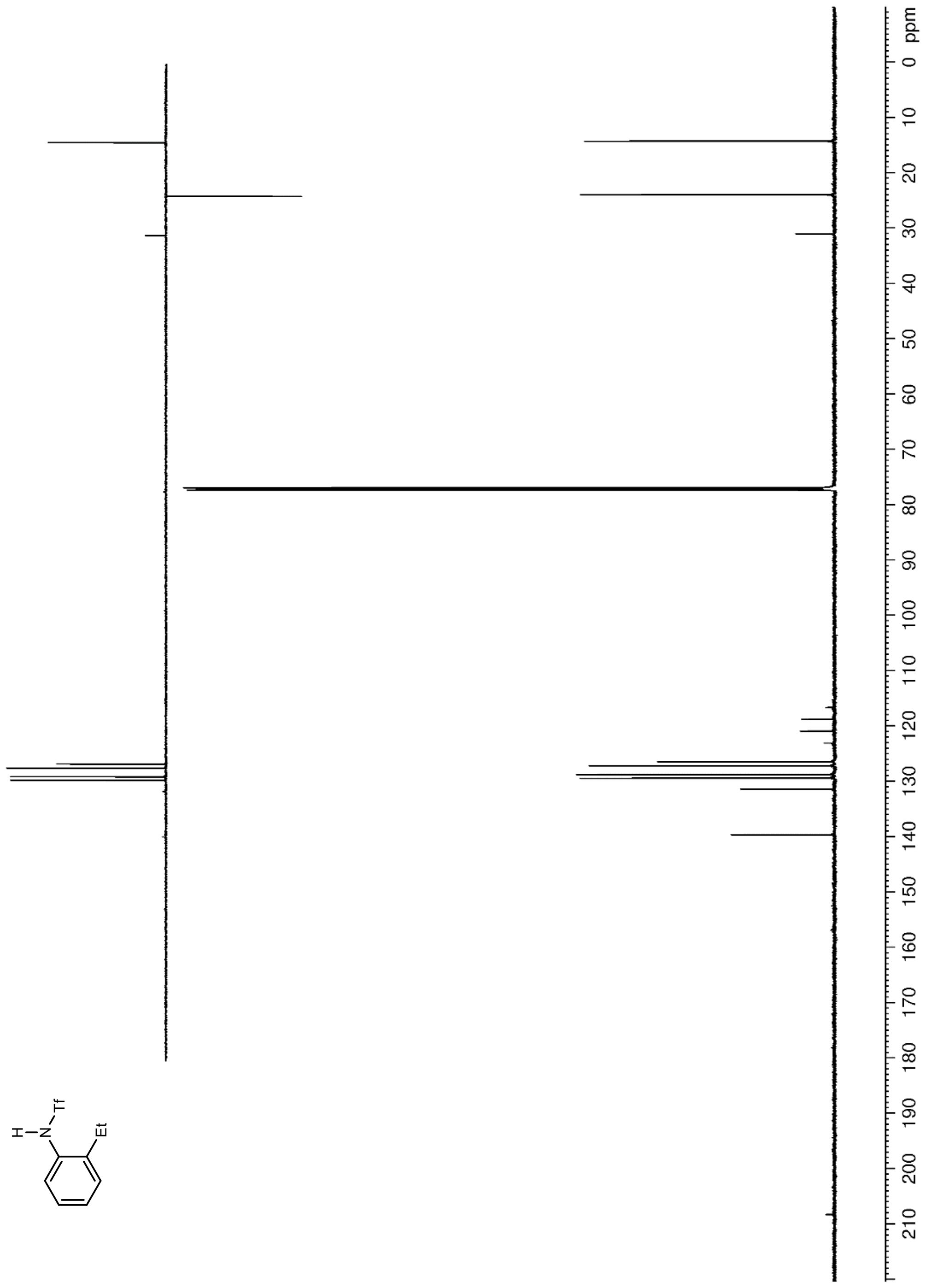

Figure 9. ^{19}F NMR (282 MHz, $CDCl_3$) of **A2**

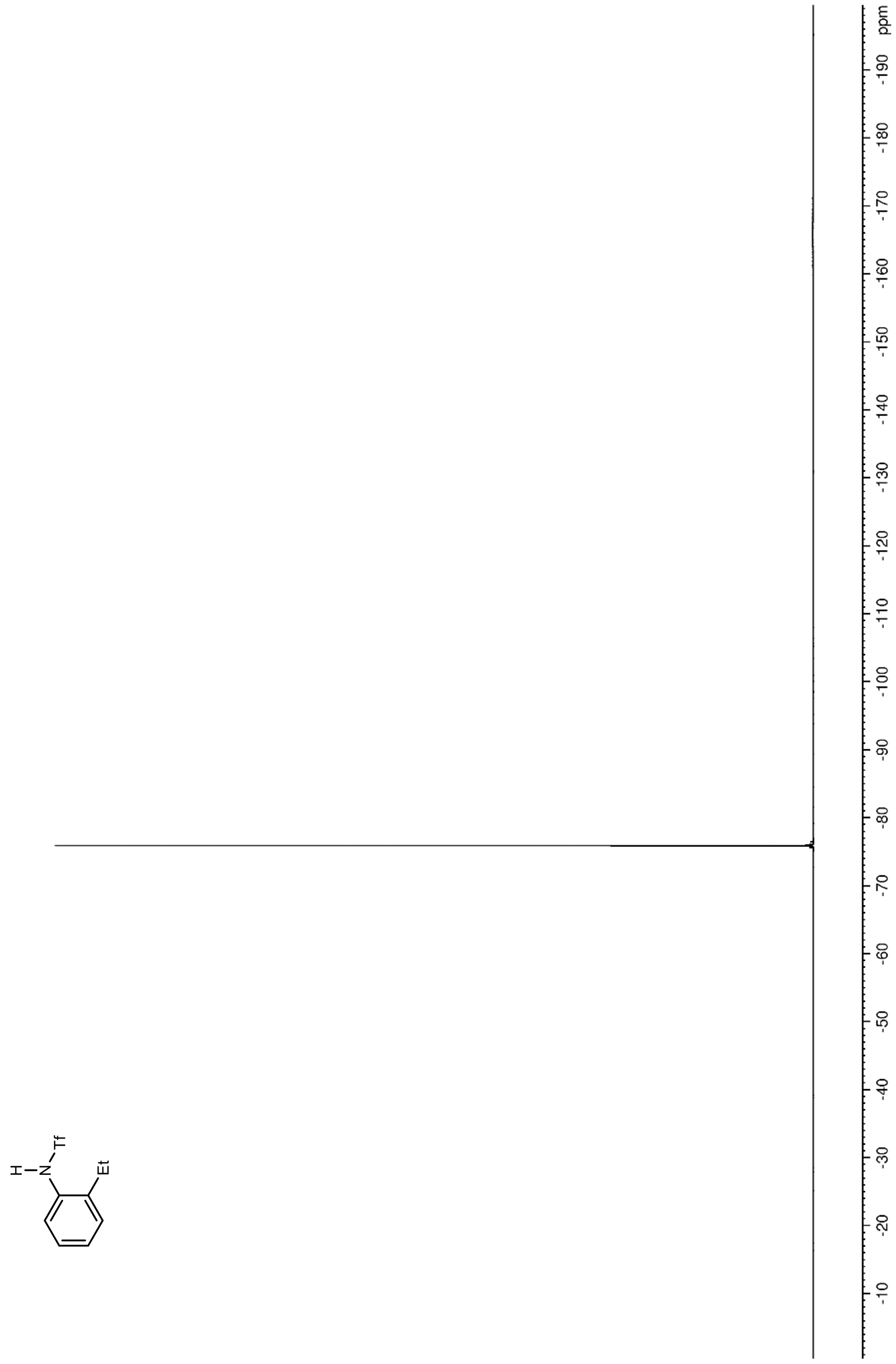

Figure 10. ^{1}H NMR (400 MHz, $CDCl_3$) of **A3**

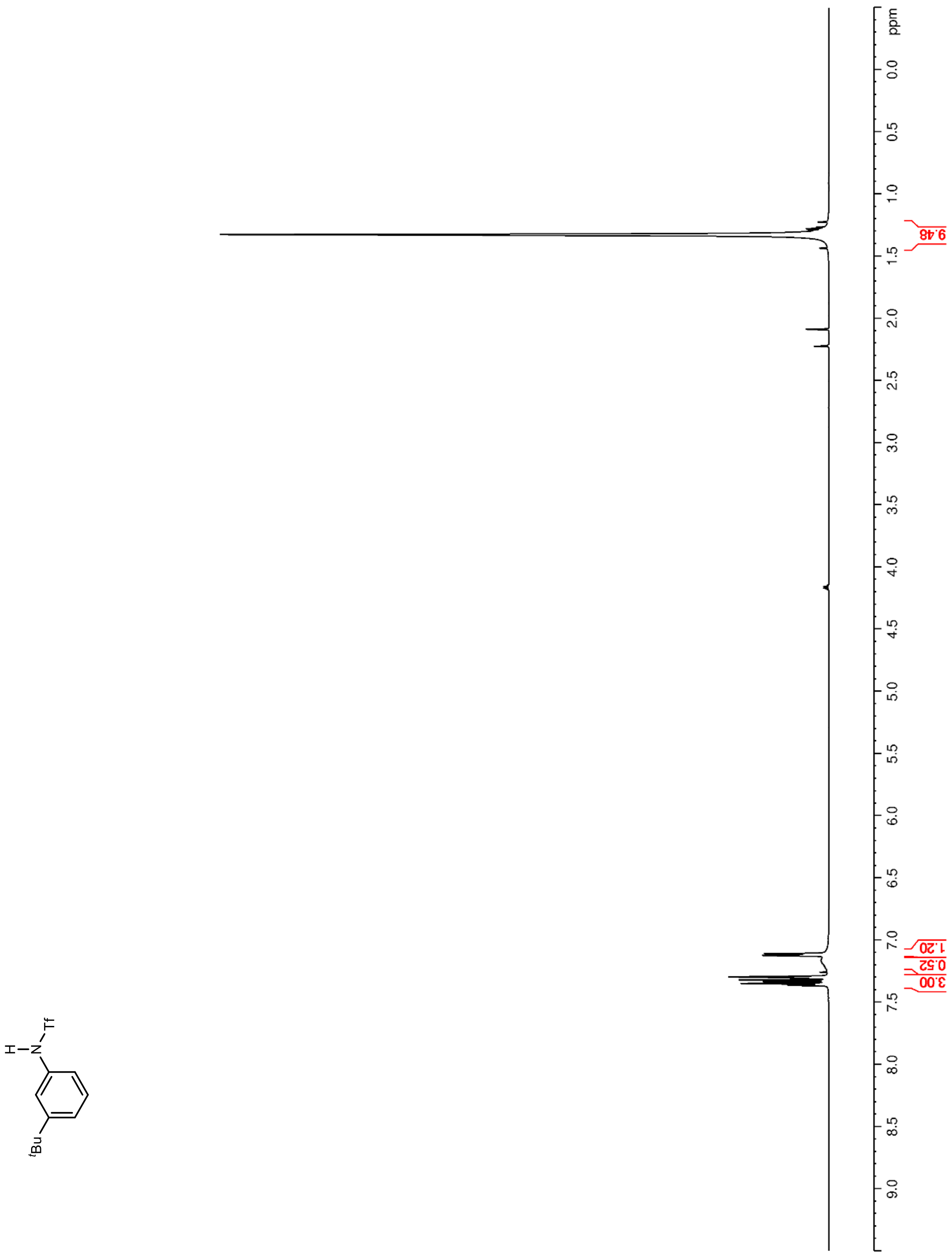

Figure 11. ^{13}C NMR (150 MHz, $CDCl_3$) of **A3**

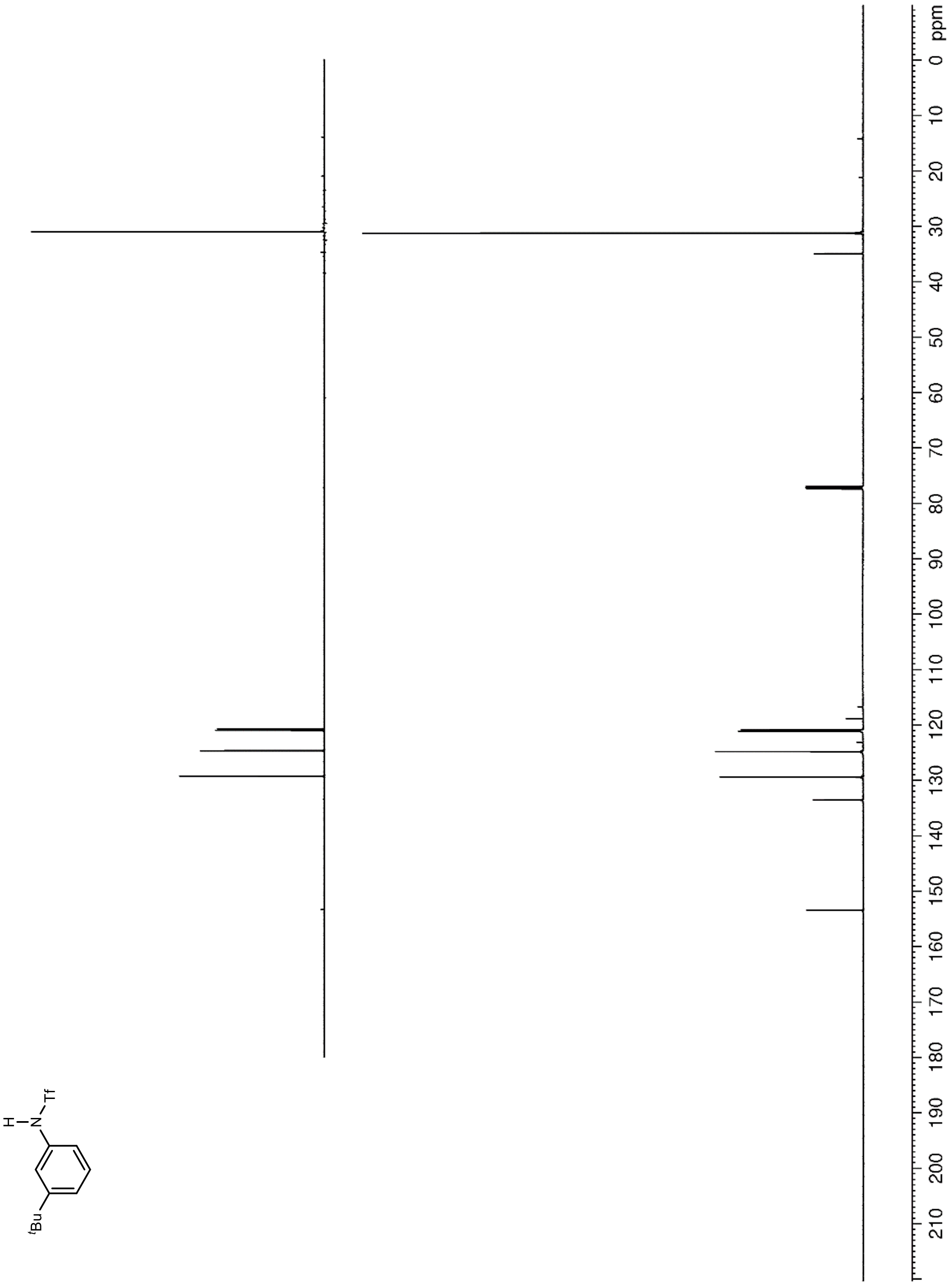

Figure 12. ^{19}F NMR (282 MHz, $CDCl_3$) of **A3**

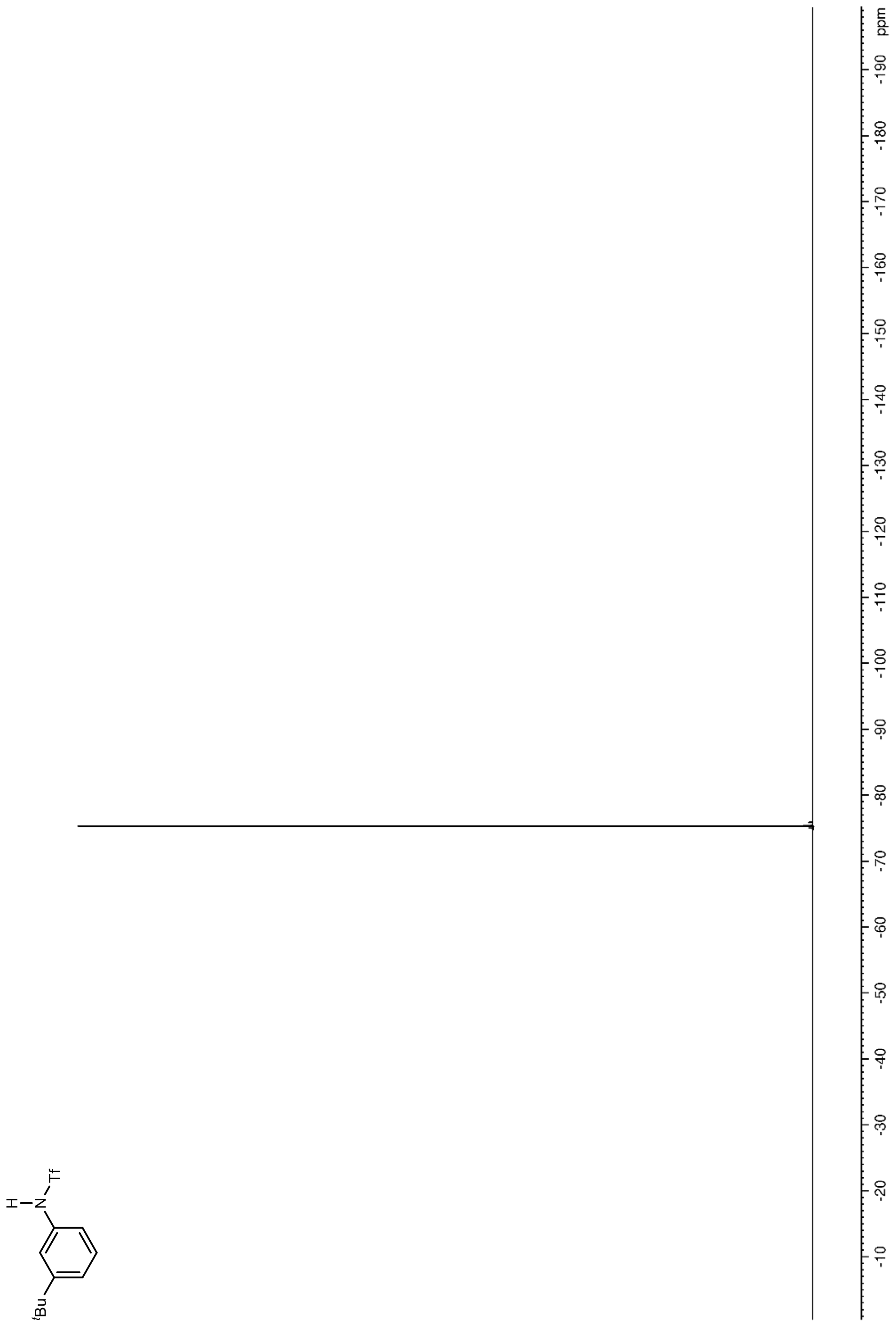

Figure 13. ^{1}H NMR (400 MHz, $CDCl_3$) of **A5**

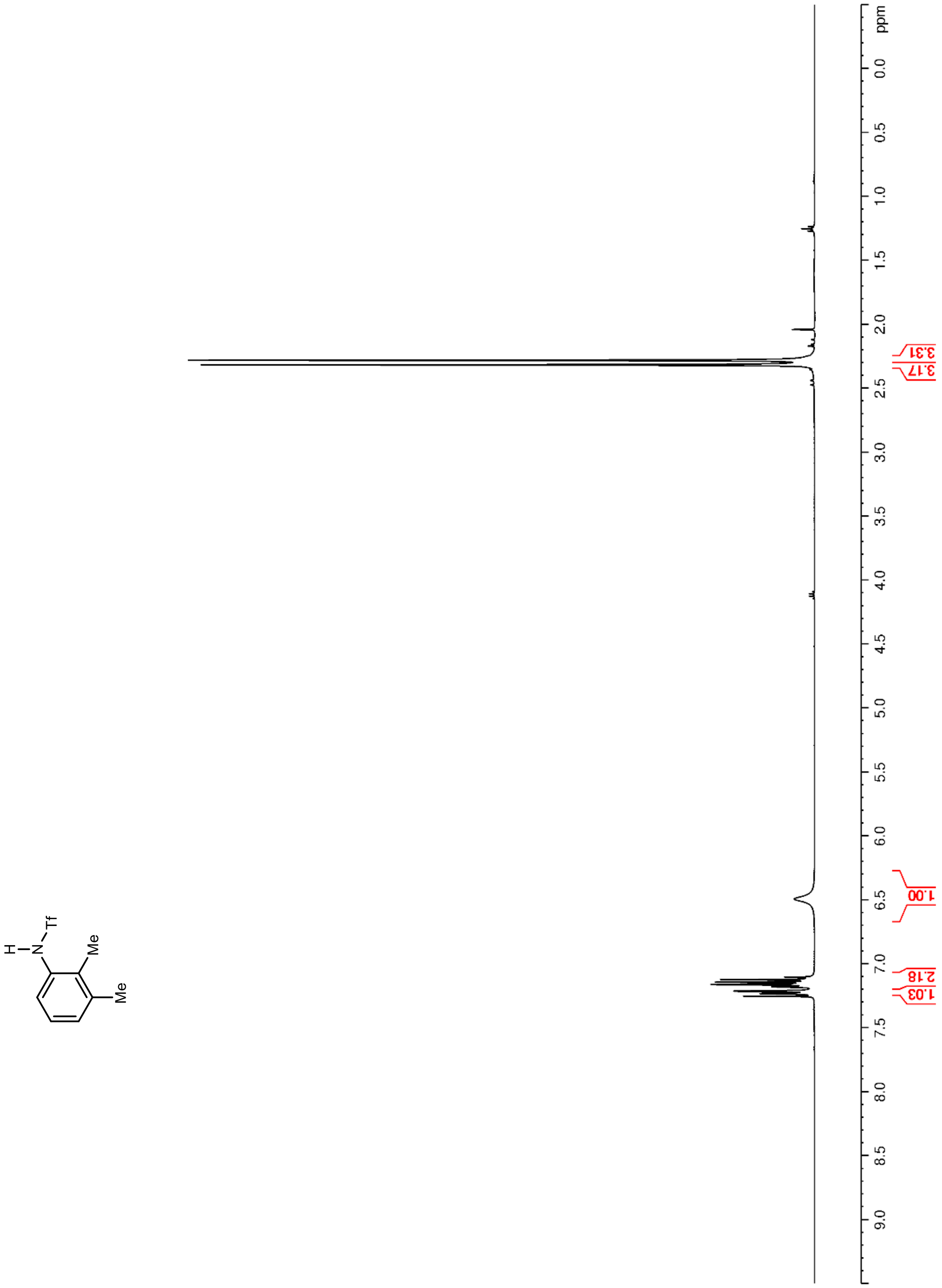

Figure 14. ^{13}C NMR (150 MHz, $CDCl_3$) of **A5**

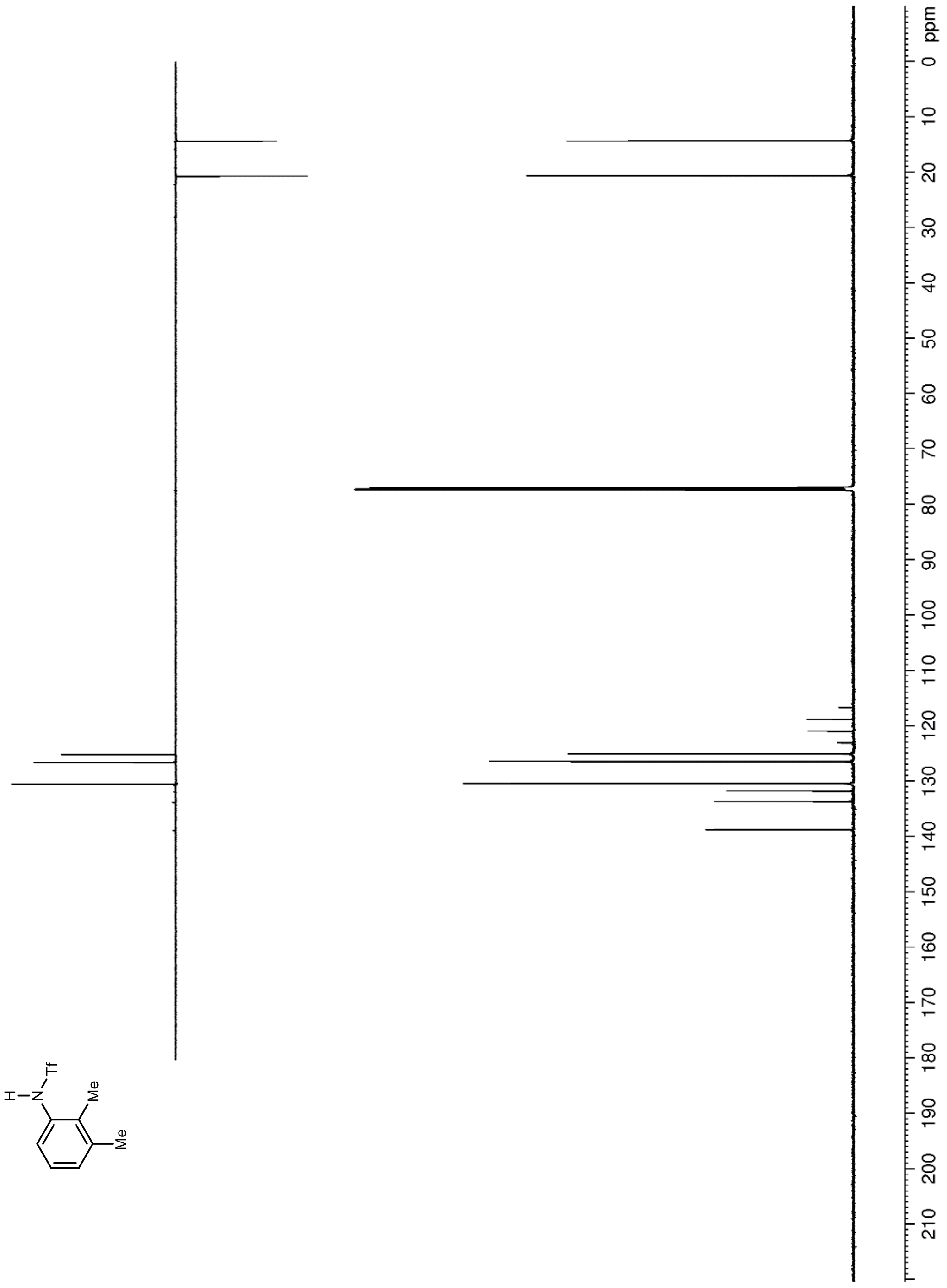

Figure 15. ^{19}F NMR (282 MHz, $CDCl_3$) of **A5**

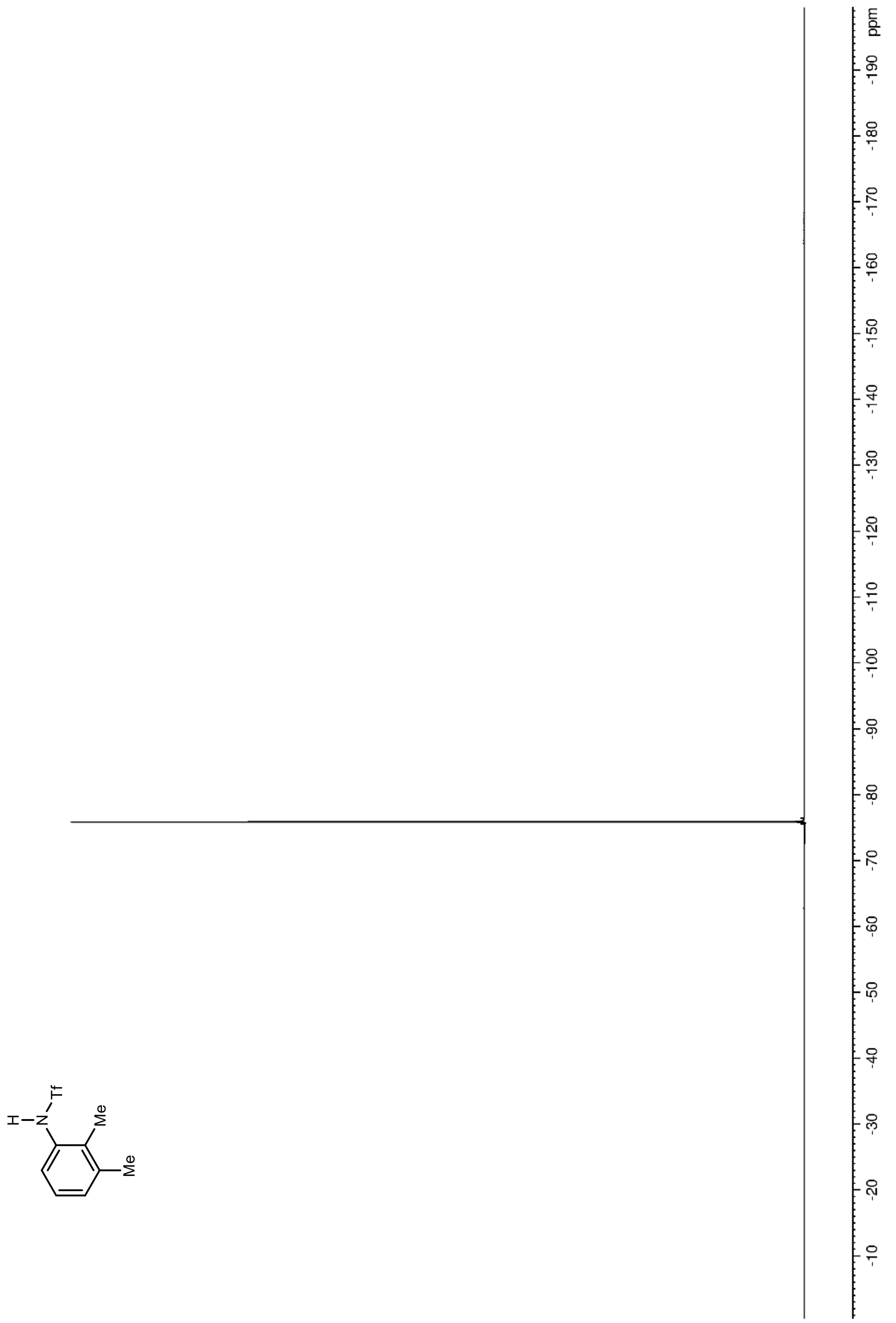

Figure 16. ^{1}H NMR (400 MHz, $CDCl_3$) of **A6**

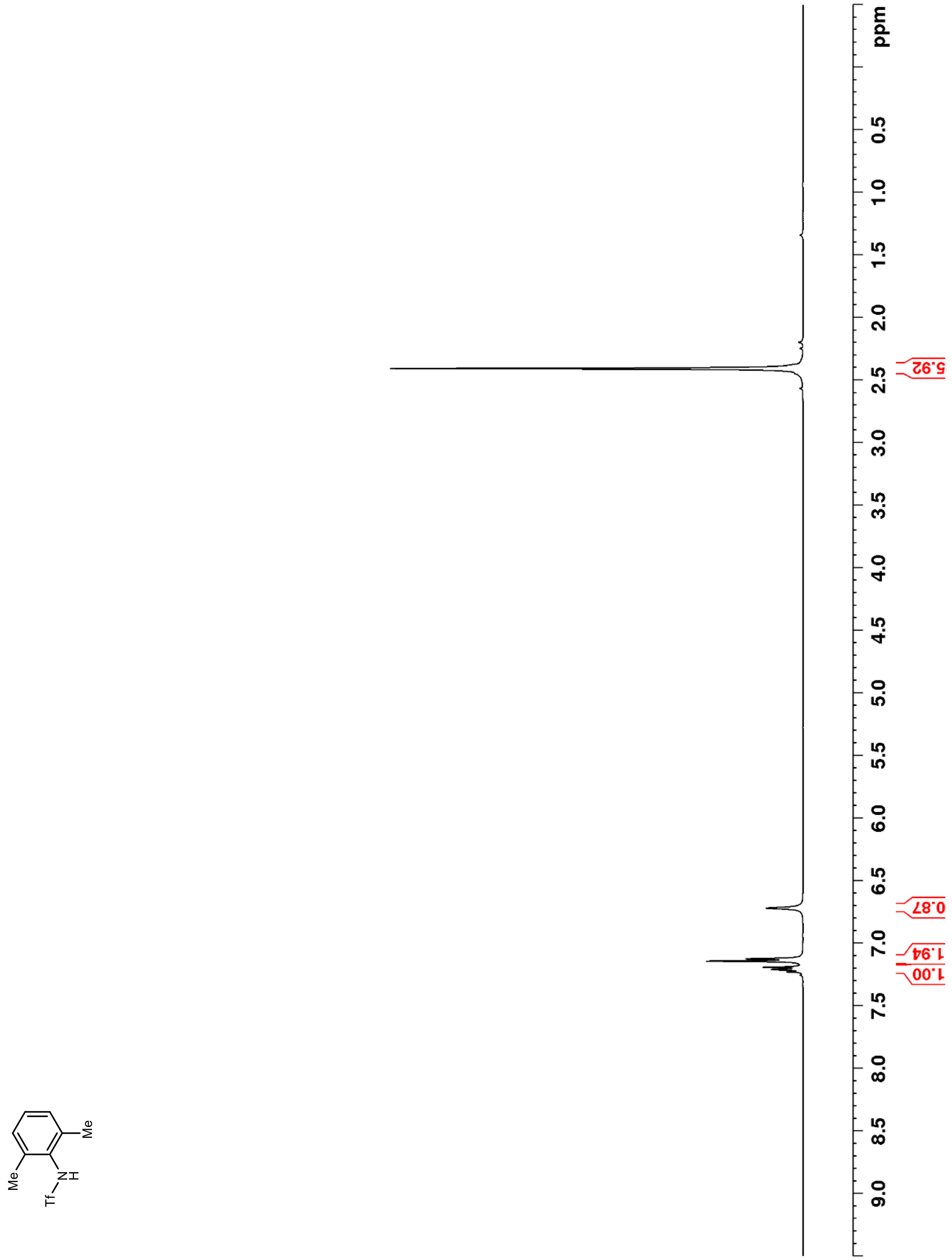

Figure 17. ^{13}C NMR (150 MHz, $CDCl_3$) of **A6**

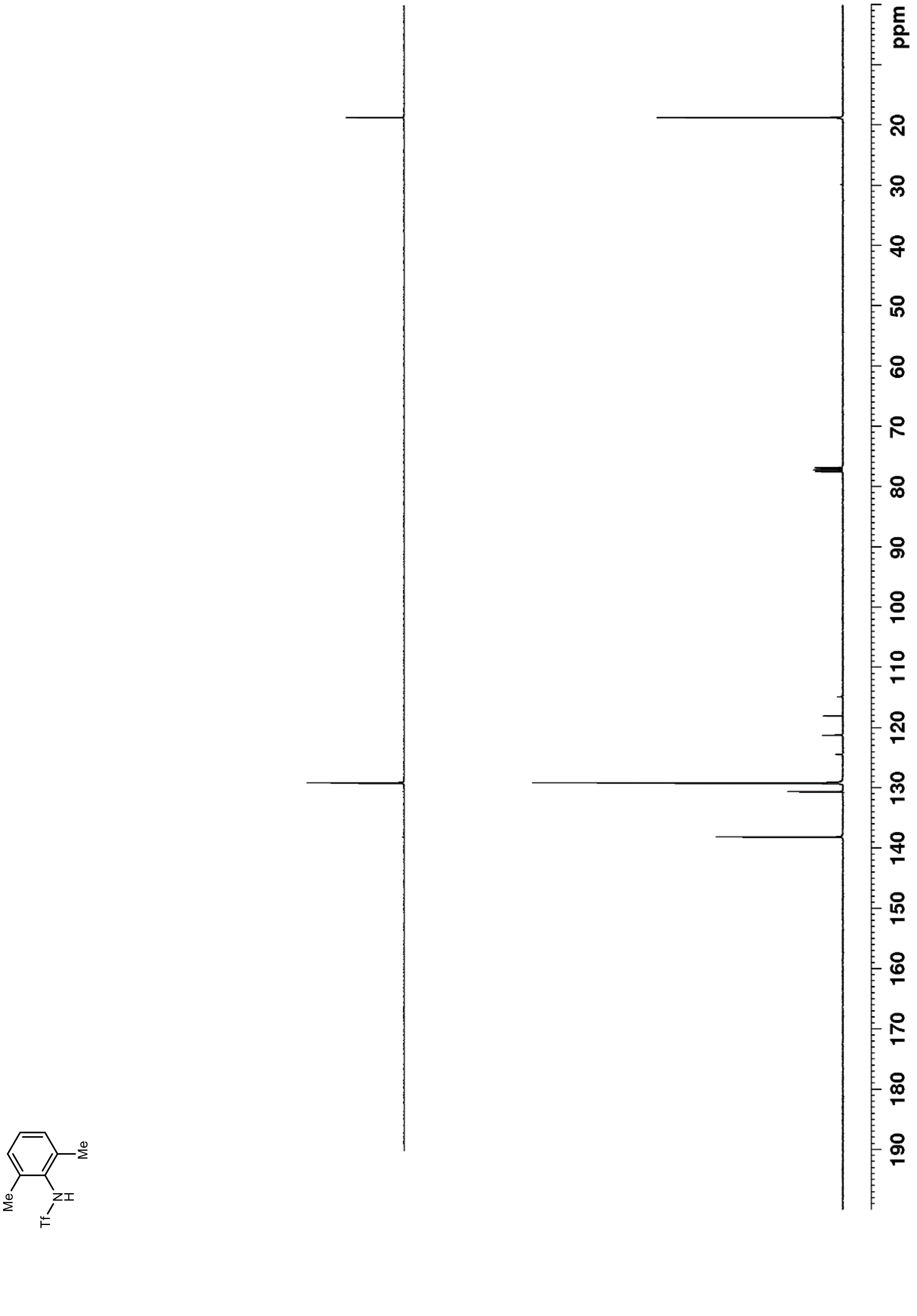

Figure 18. ^{19}F NMR (282 MHz, $CDCl_3$) of **A6**

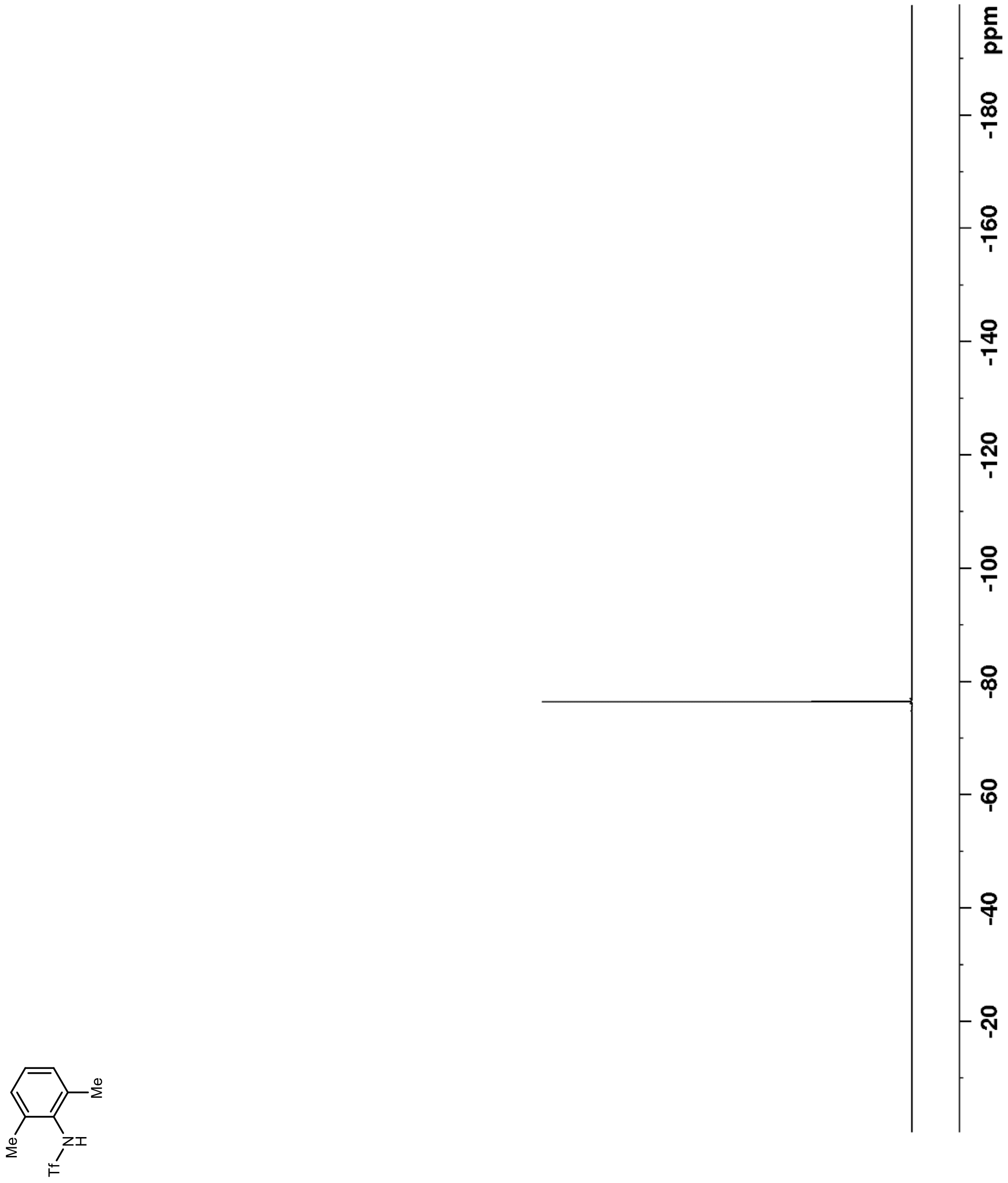

Figure 19. ^{1}H NMR (400 MHz, $CDCl_3$) of **A8**

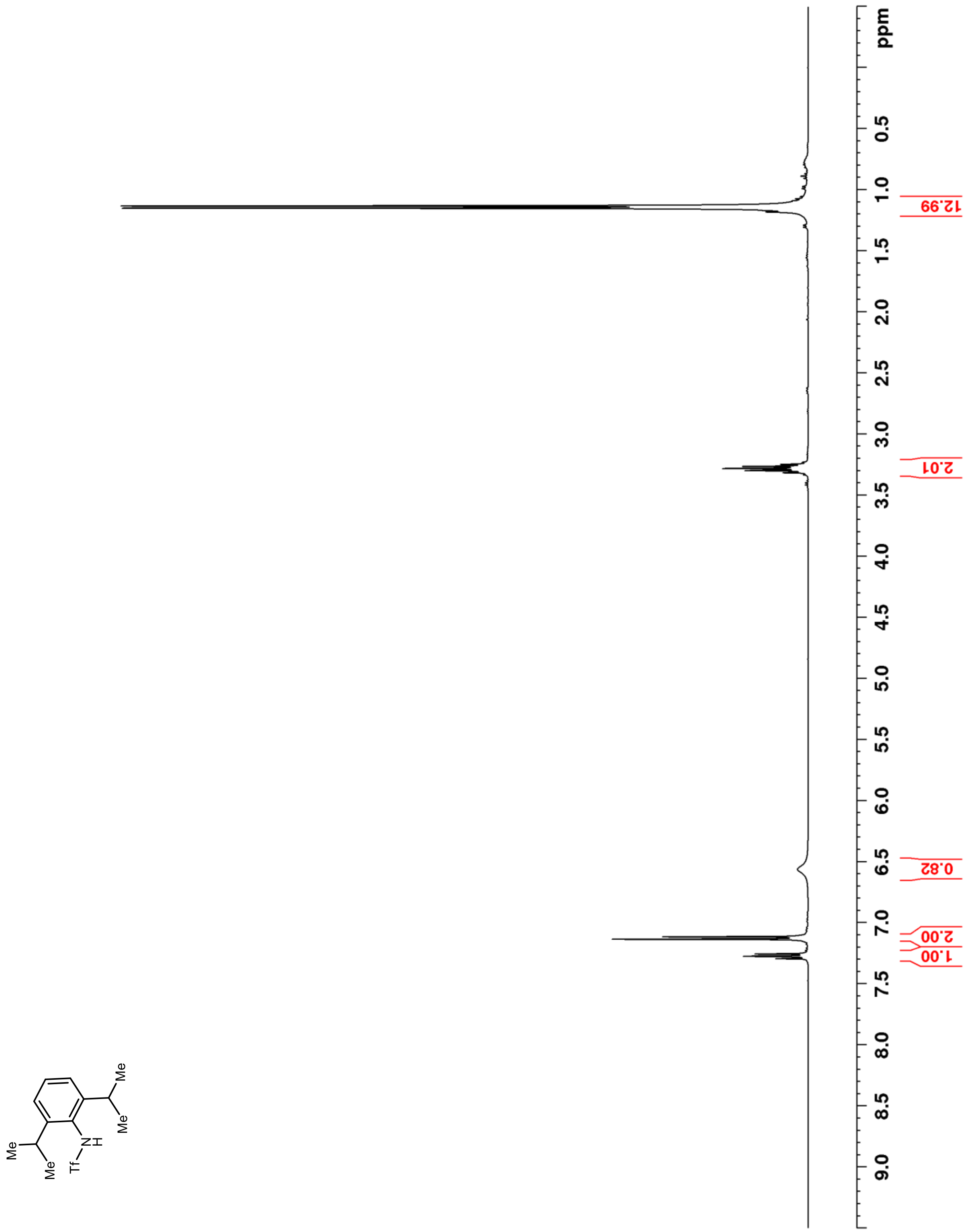

Figure 20. ^{13}C NMR (150 MHz, $CDCl_3$) of **A8**

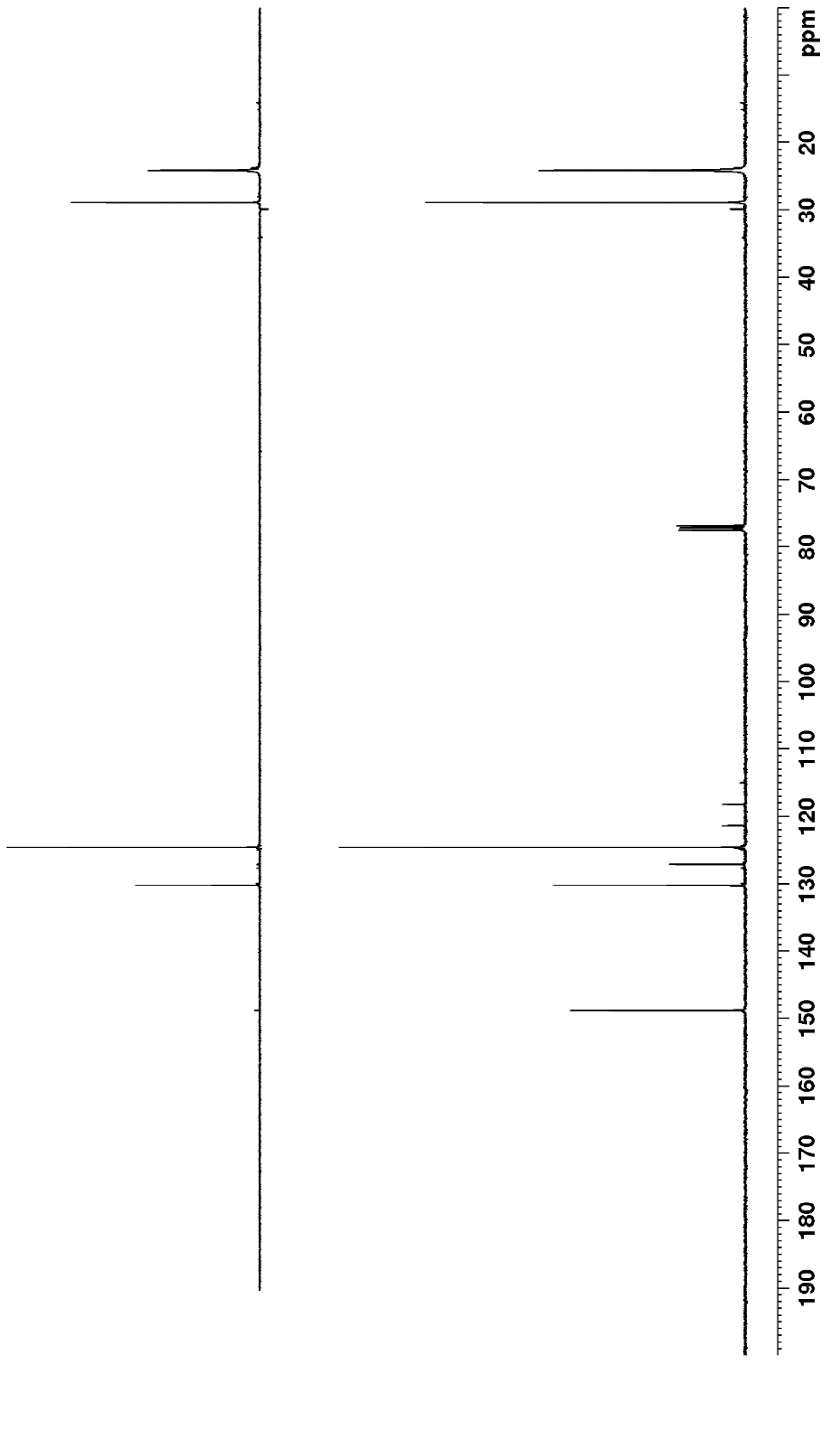

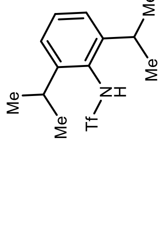

Figure 21. ^{19}F NMR (282 MHz, $CDCl_3$) of **A8**

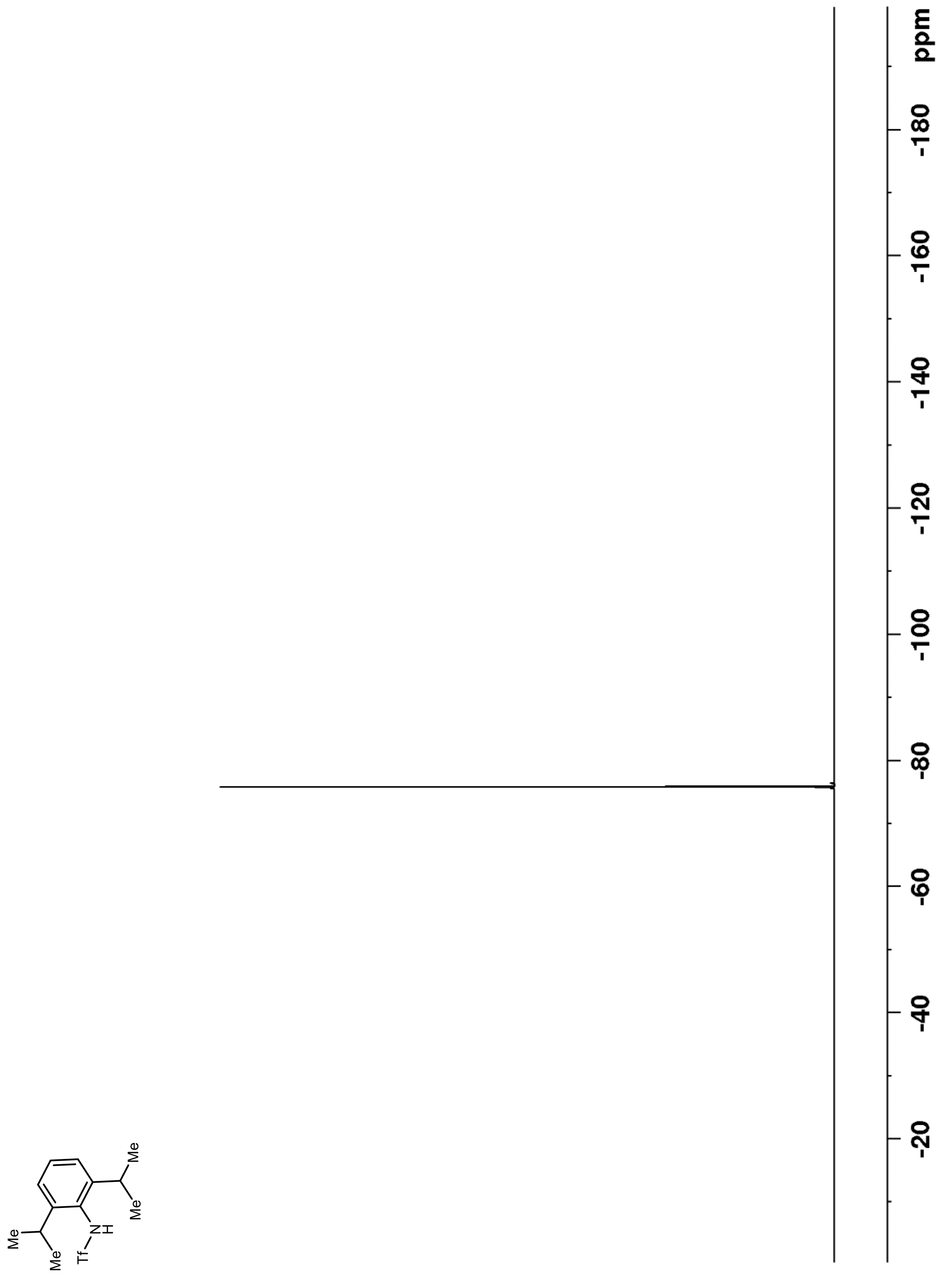

Figure 22. ^{1}H NMR (400 MHz, $CDCl_3$) of **B4**

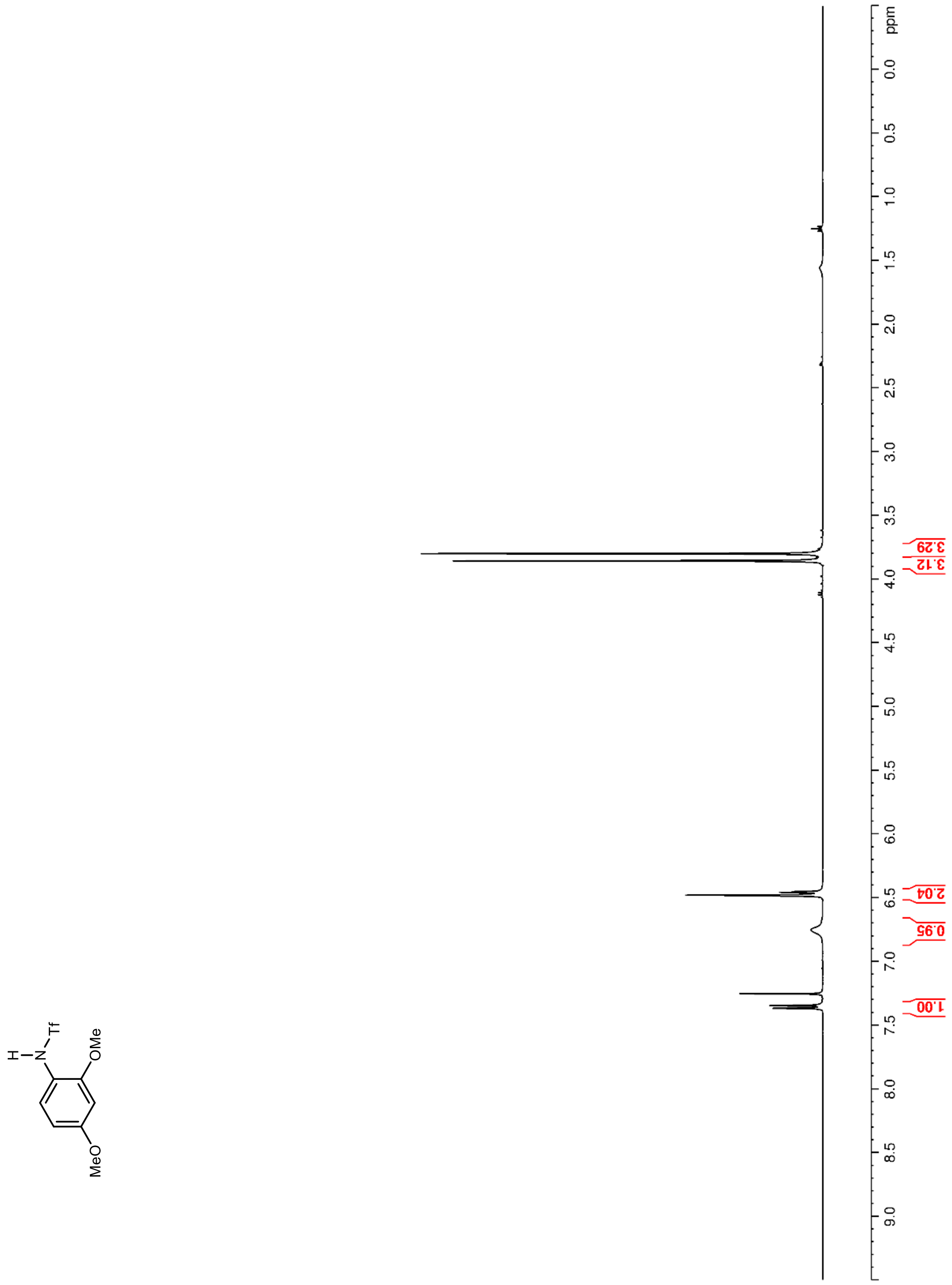

Figure 23. ^{13}C NMR (150 MHz, $CDCl_3$) of **B4**

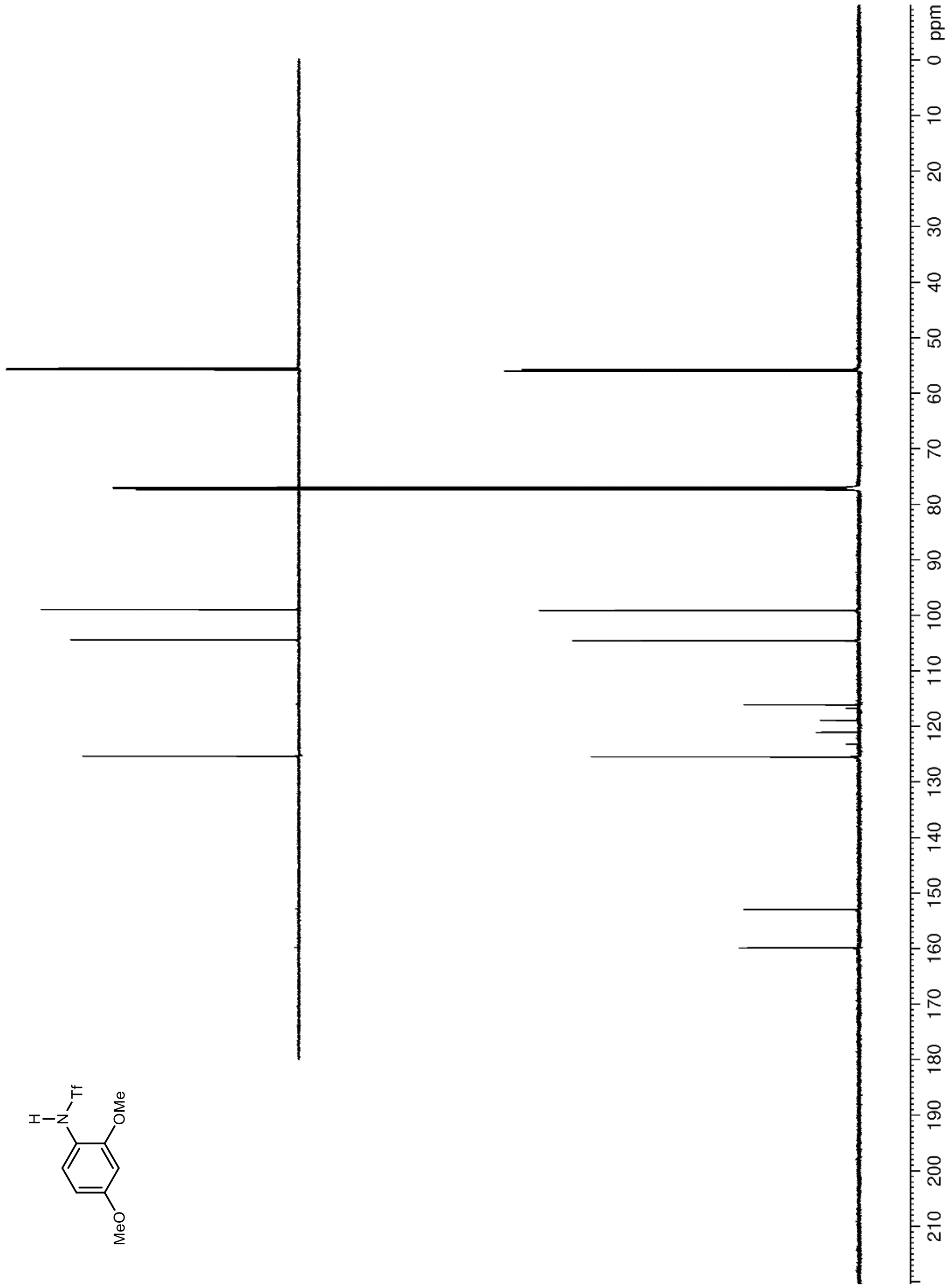

Figure 24. ^{19}F NMR (282 MHz, $CDCl_3$) of **B4**

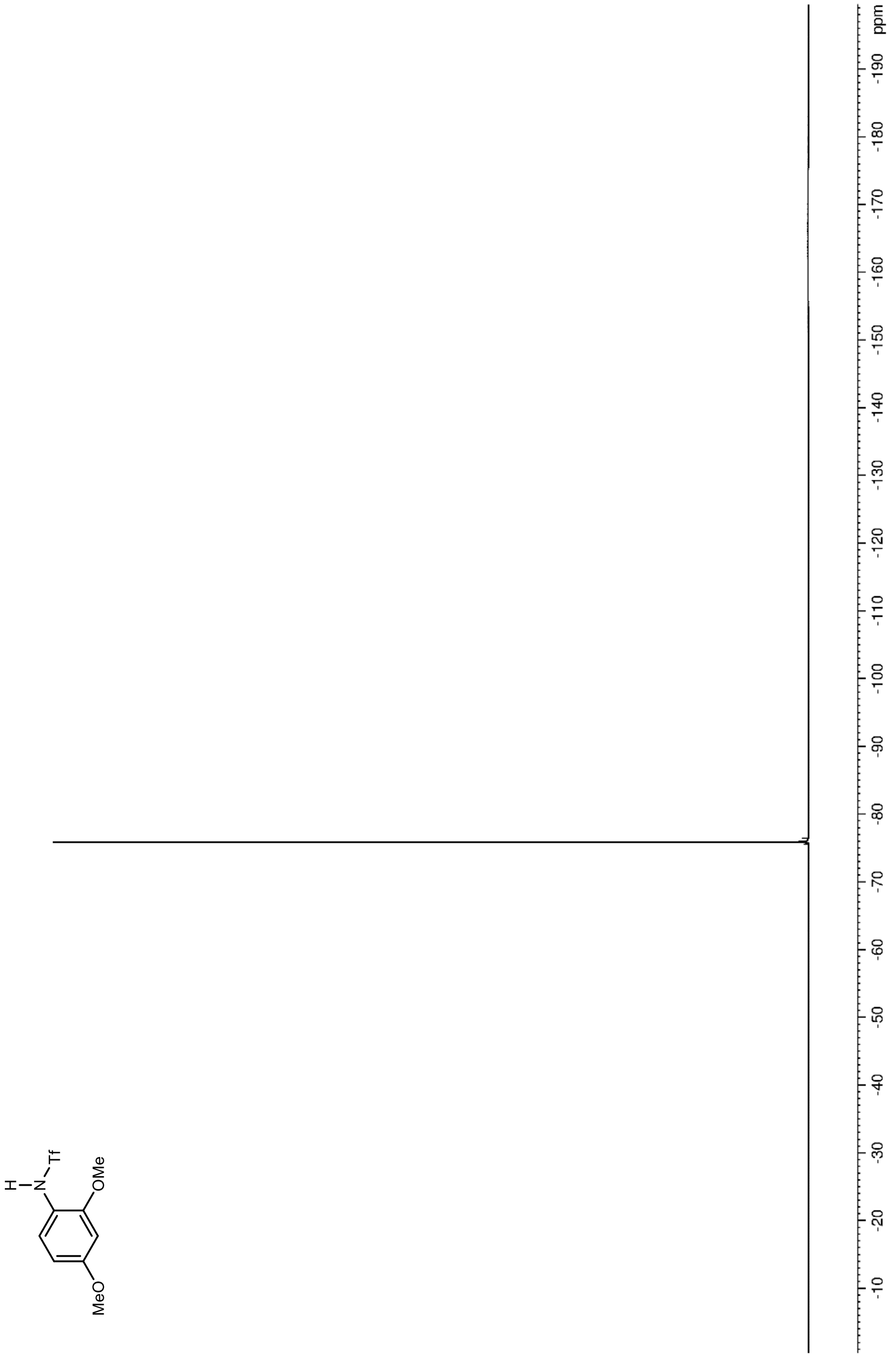

Figure 25. ^{1}H NMR (400 MHz, $CDCl_3$) of **B5**

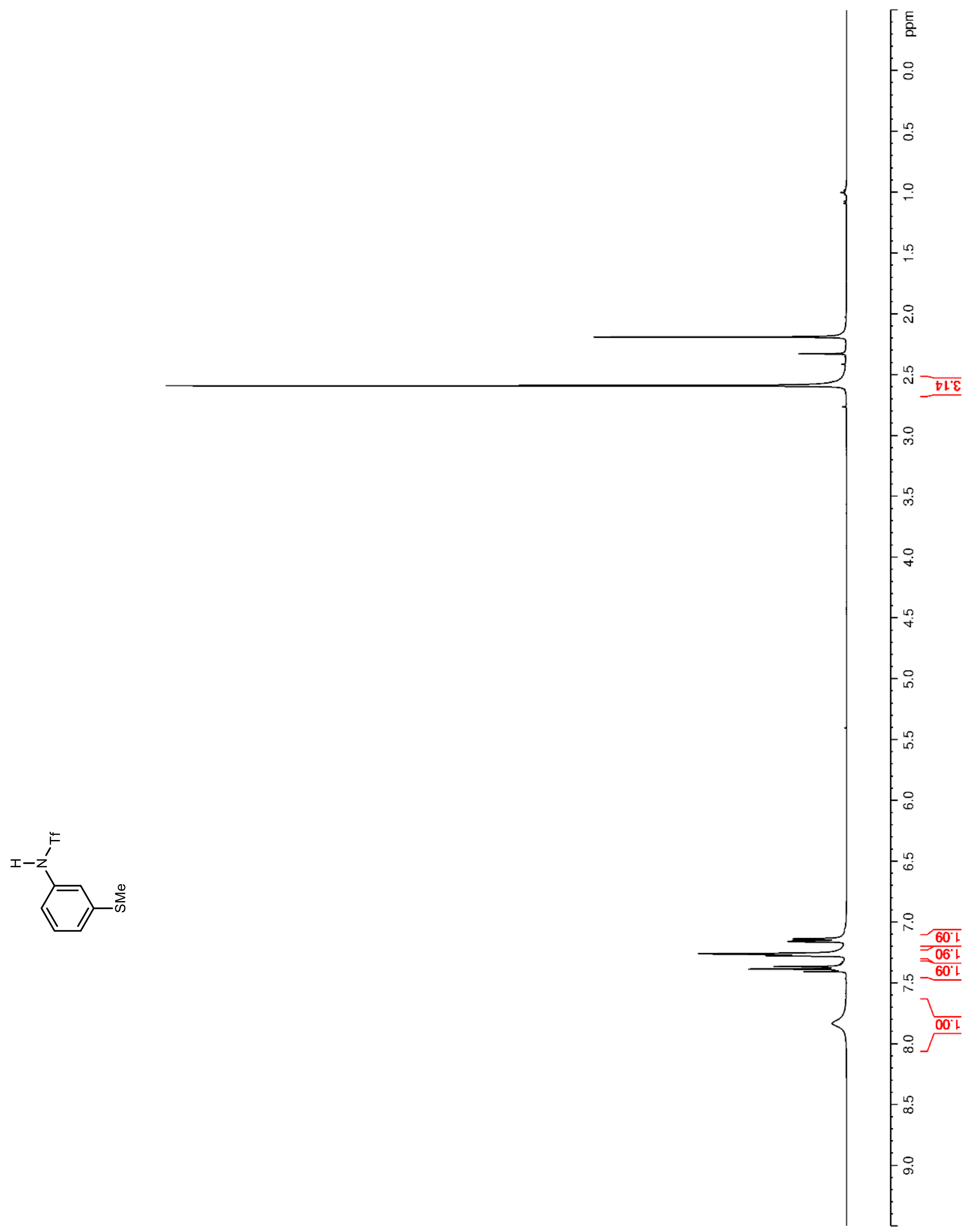

Figure 26. ^{13}C NMR (150 MHz, $CDCl_3$) of **B5**

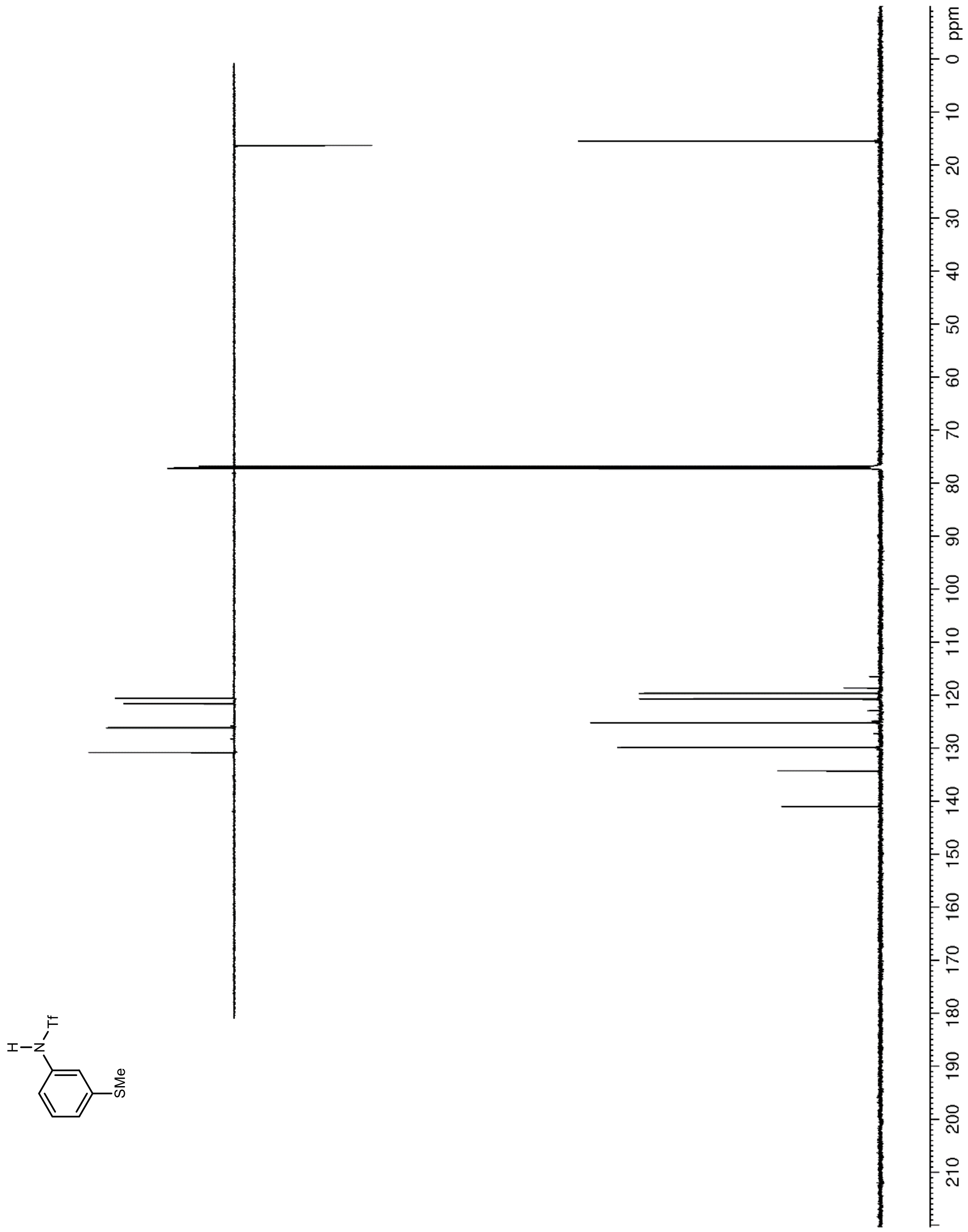

Figure 27. ^{19}F NMR (282 MHz, $CDCl_3$) of **B5**

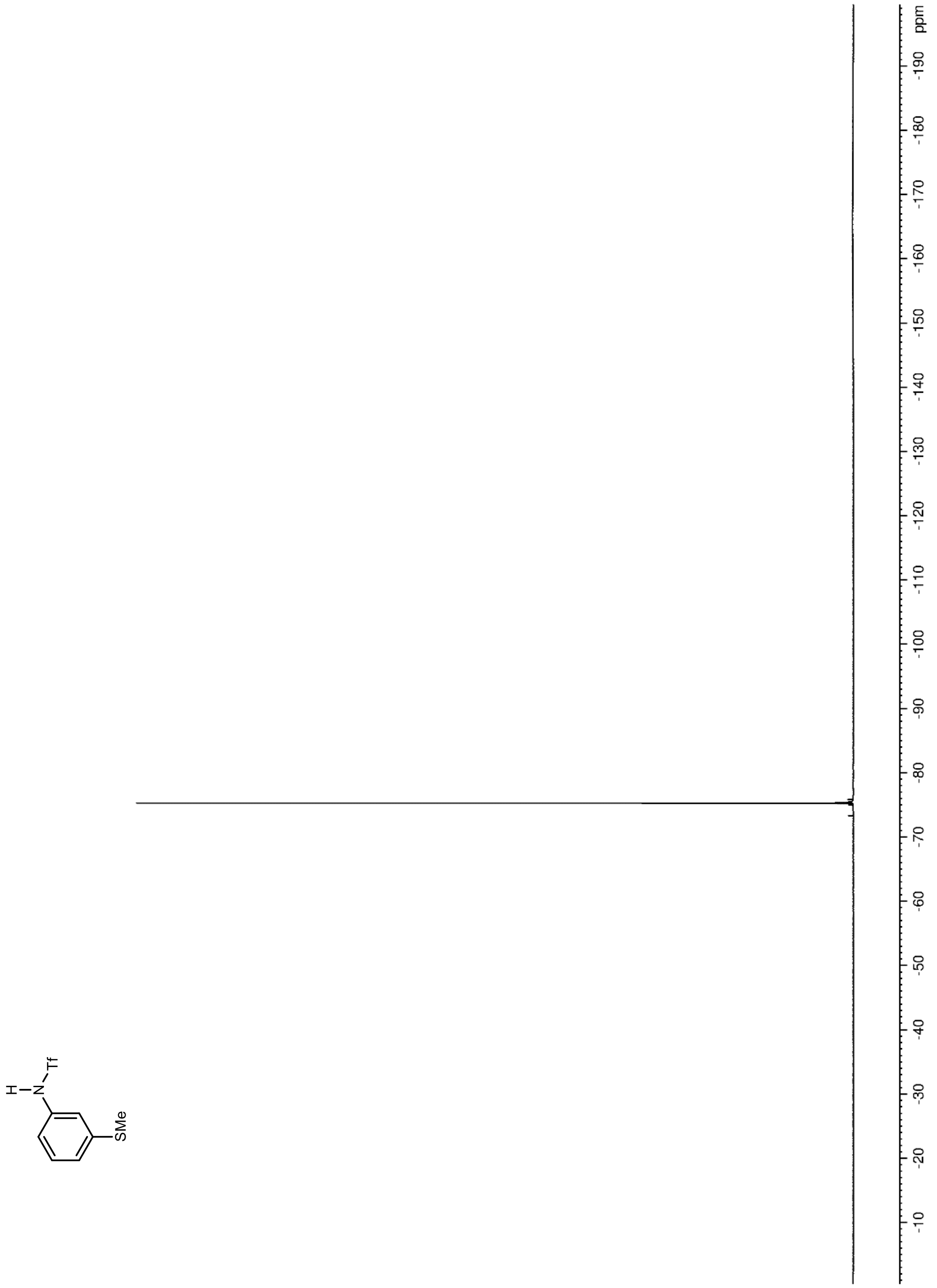

Figure 28. ^{1}H NMR (400 MHz, $CDCl_3$) of **B6**

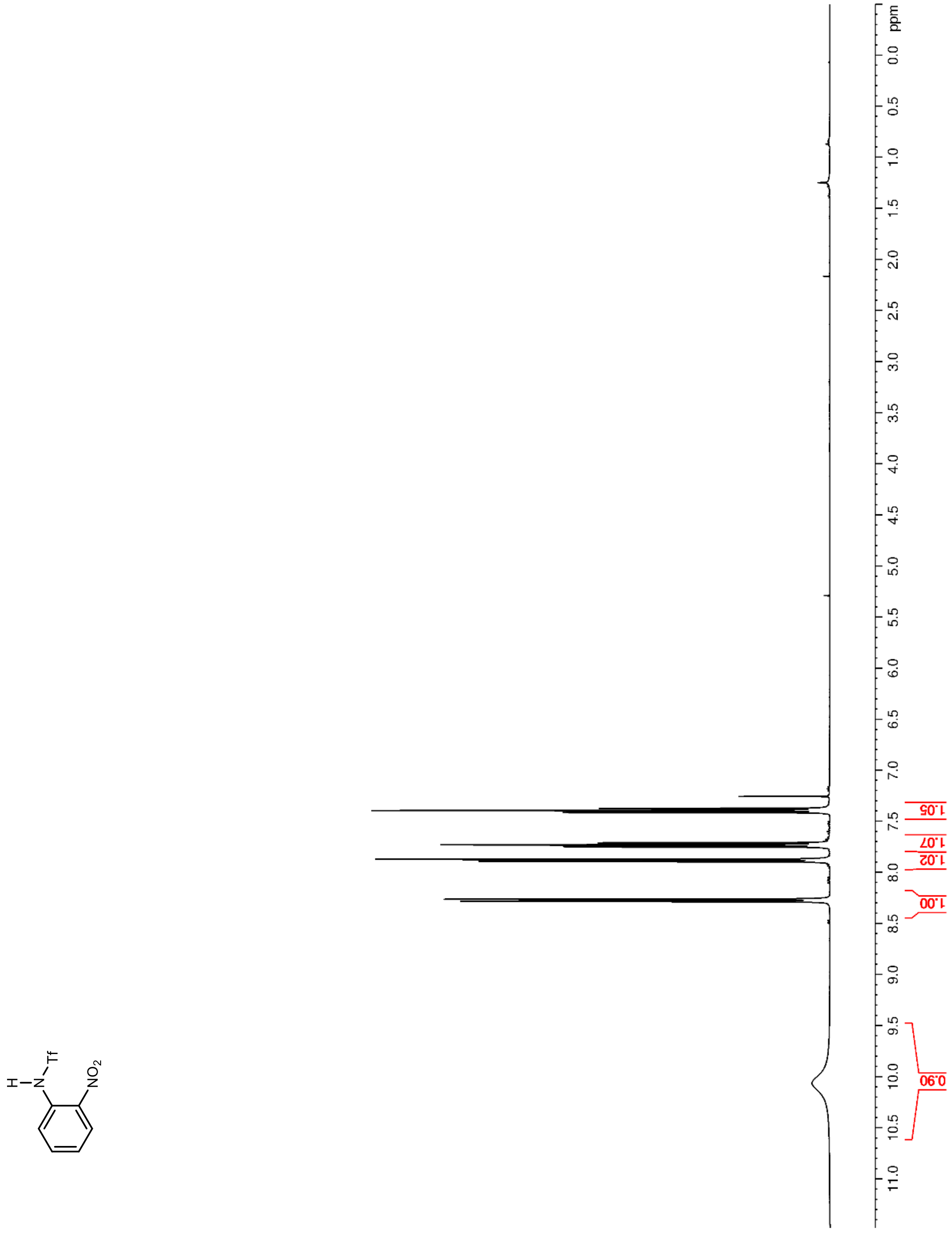

Figure 29. ^{13}C NMR (150 MHz, $CDCl_3$) of **B6**

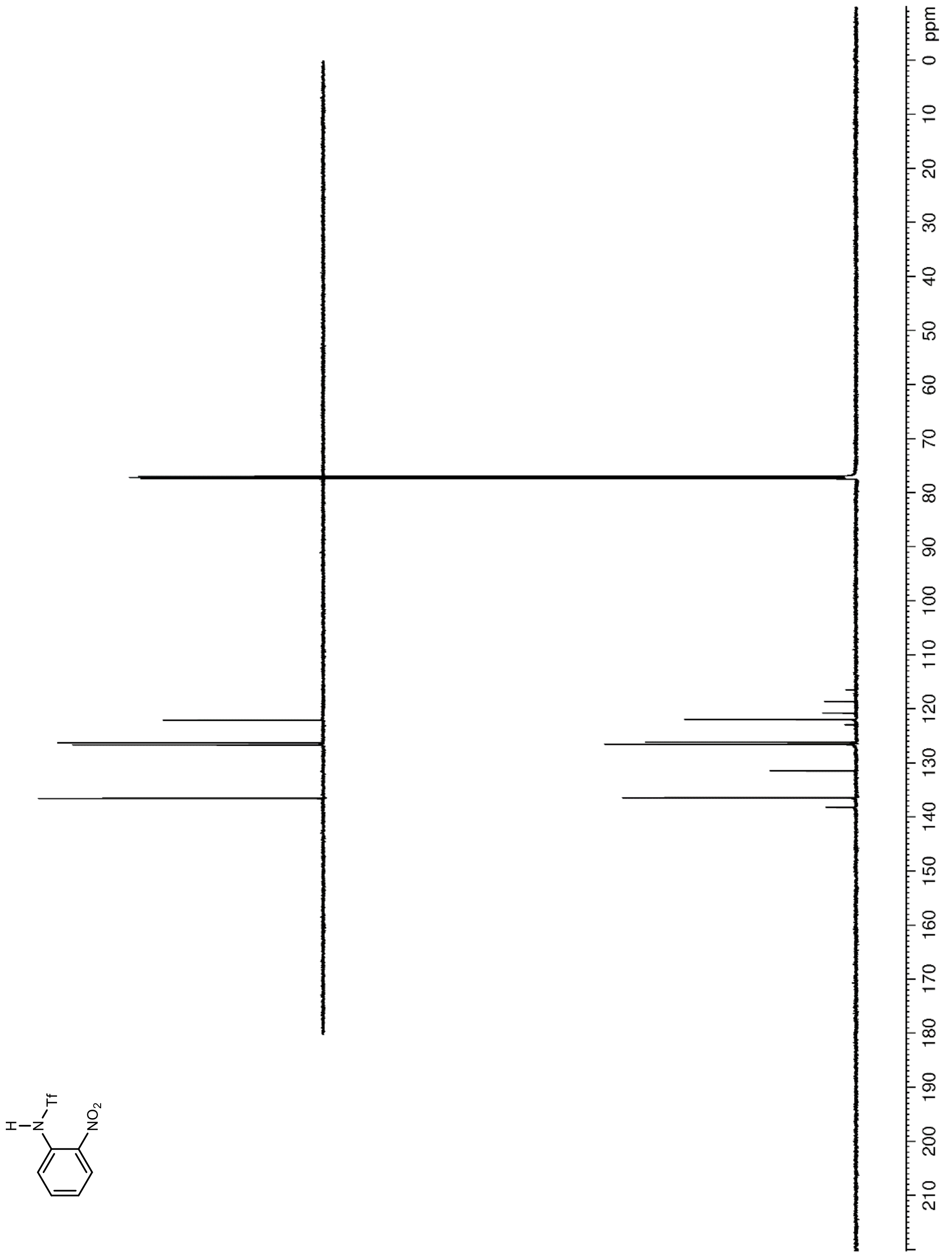

Figure 30. ^{19}F NMR (282 MHz, $CDCl_3$) of **B6**

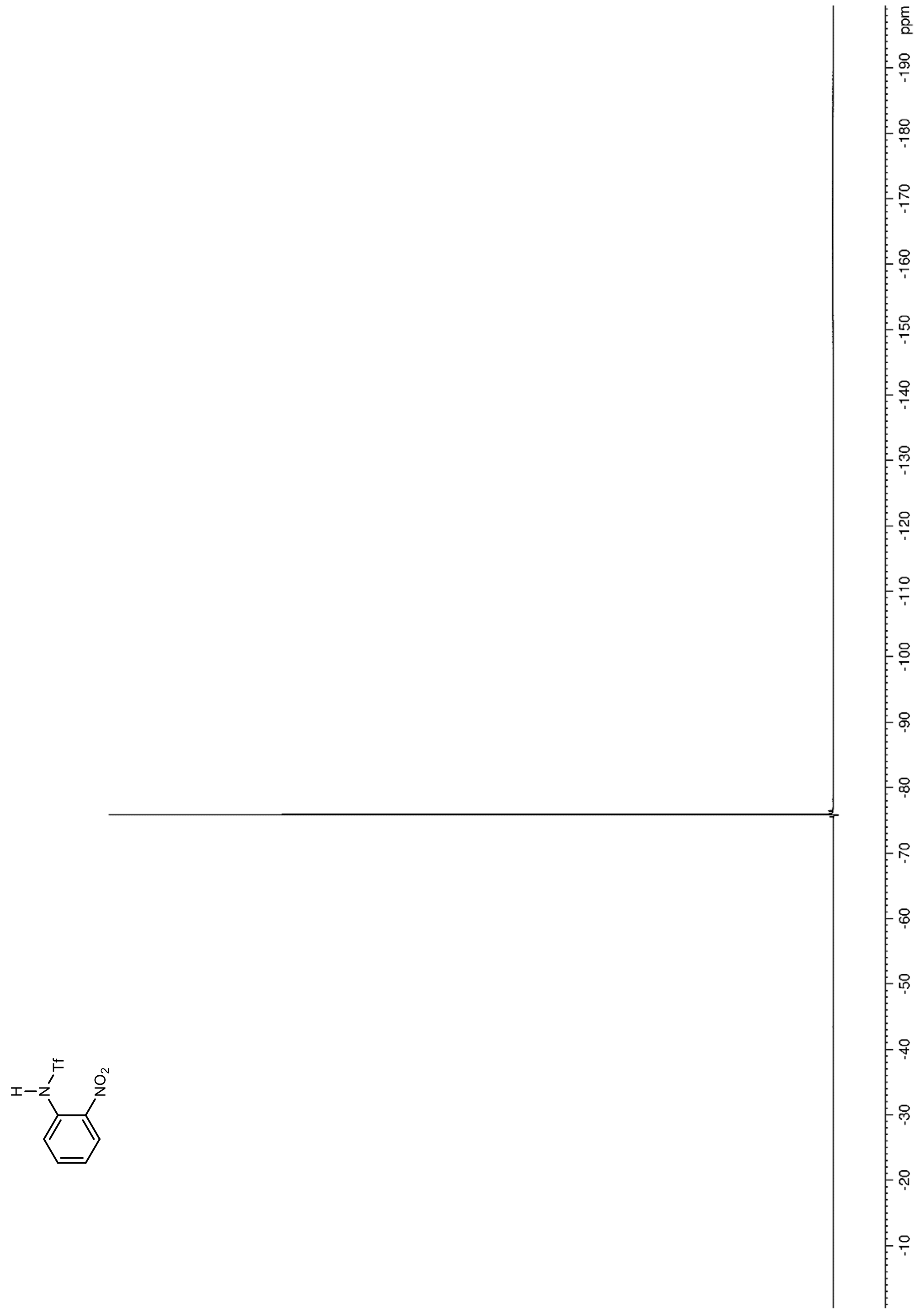

Figure 31. ^{1}H NMR (400 MHz, $CDCl_3$) of **C5**

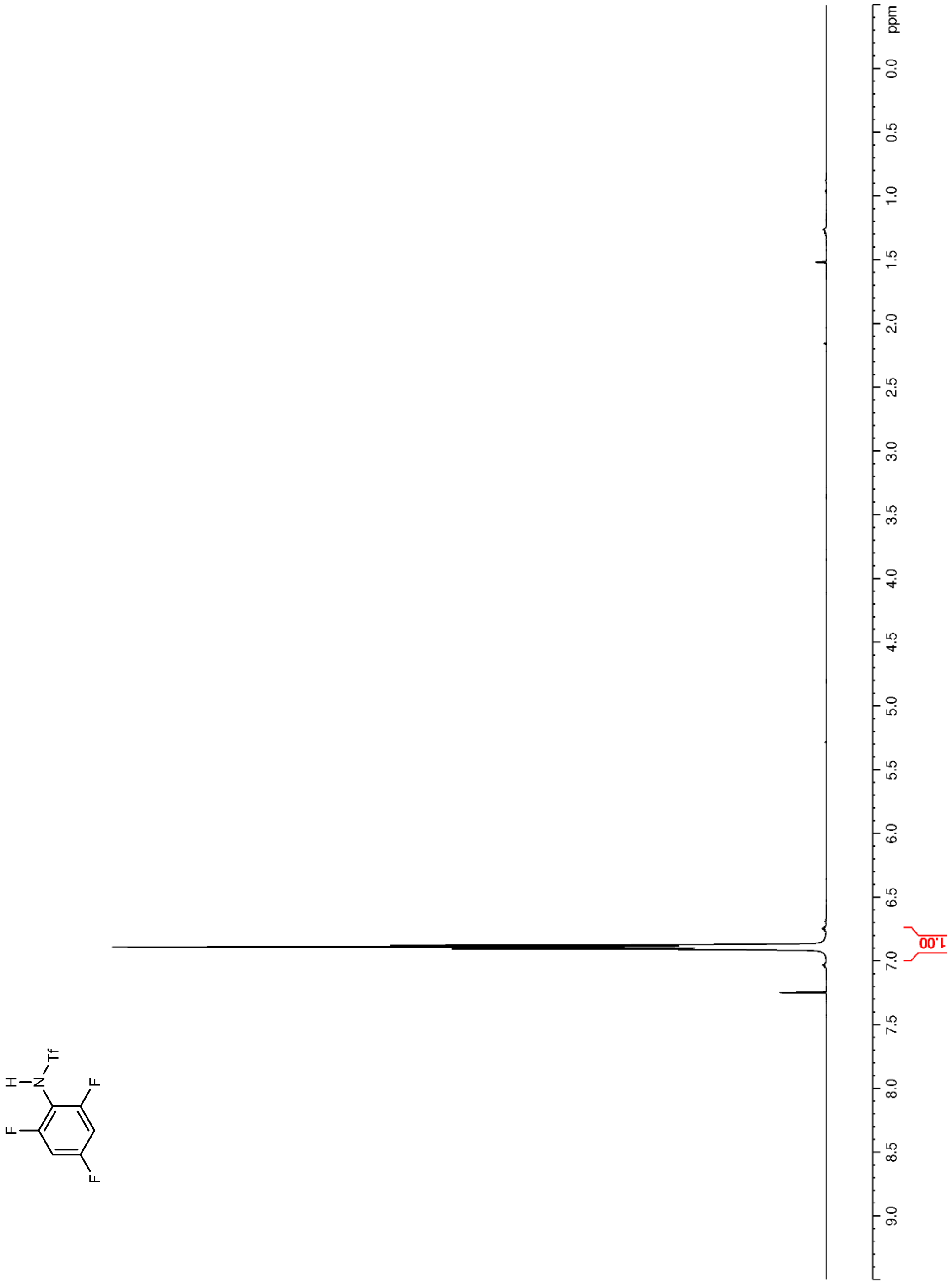

Figure 32. ^{13}C NMR (150 MHz, $CDCl_3$) of **C5**

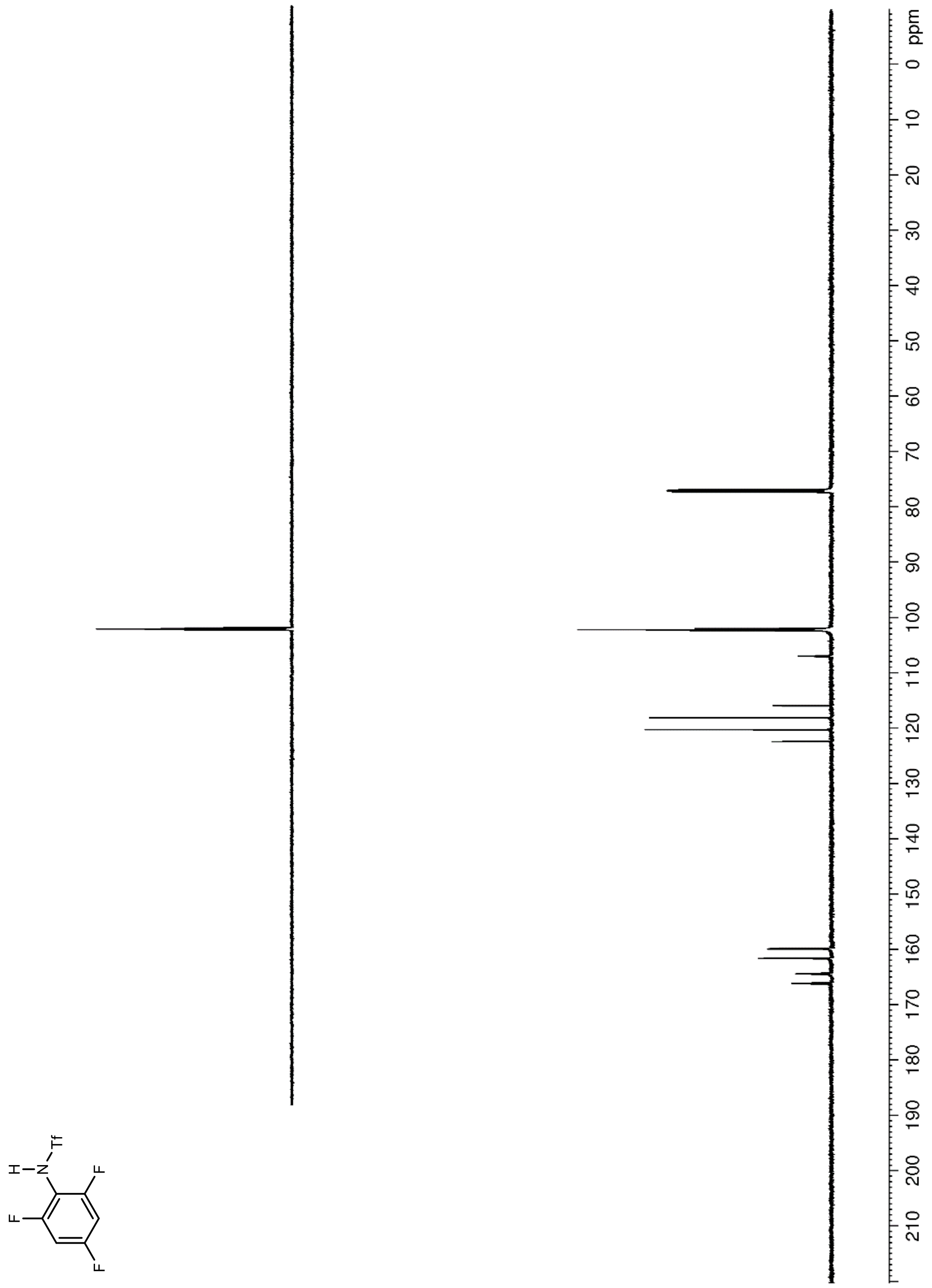

Figure 33. ^{19}F NMR (282 MHz, $CDCl_3$) of **C5**

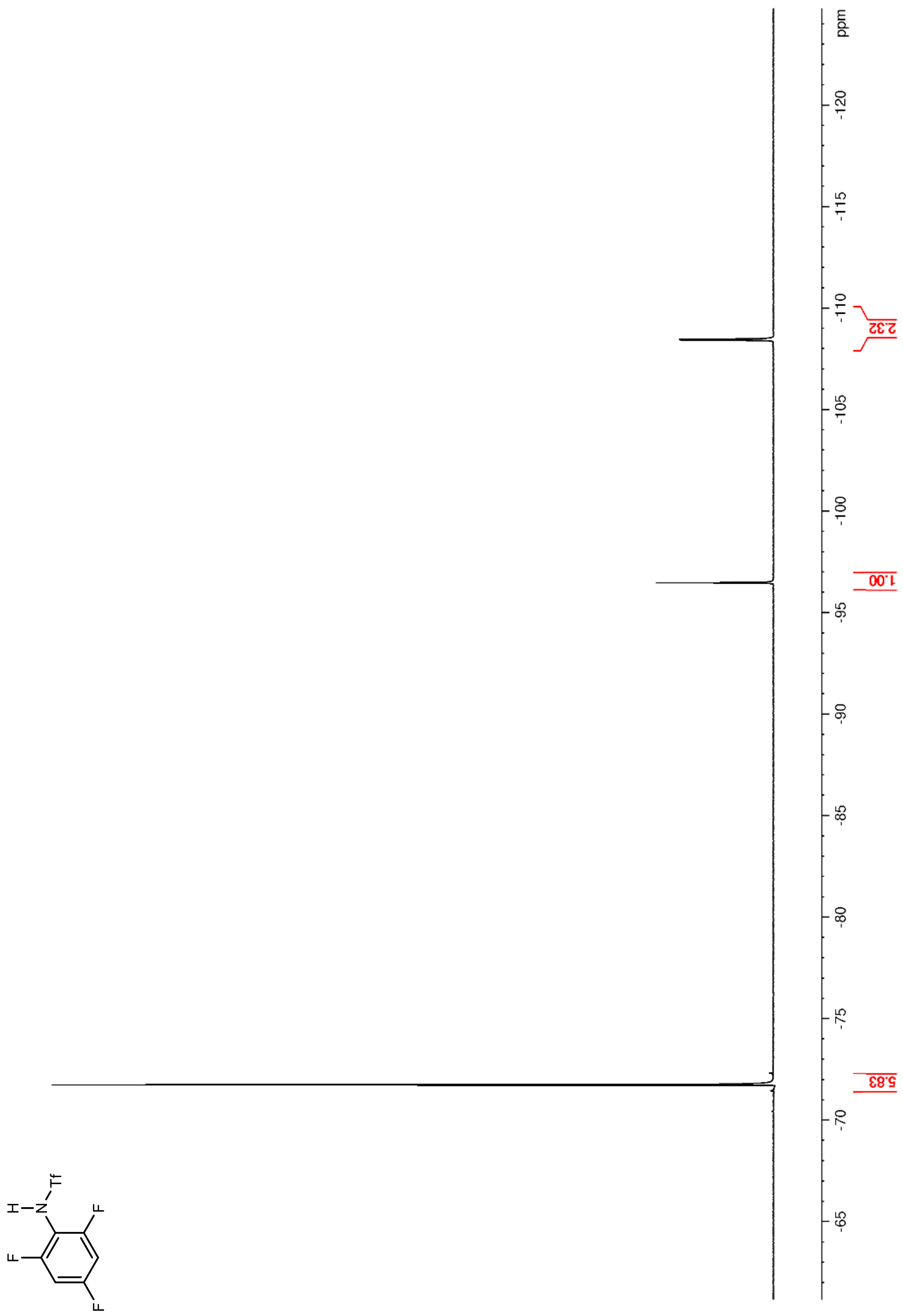

Figure 34. ^{1}H NMR (400 MHz, $CDCl_3$) of **C6**

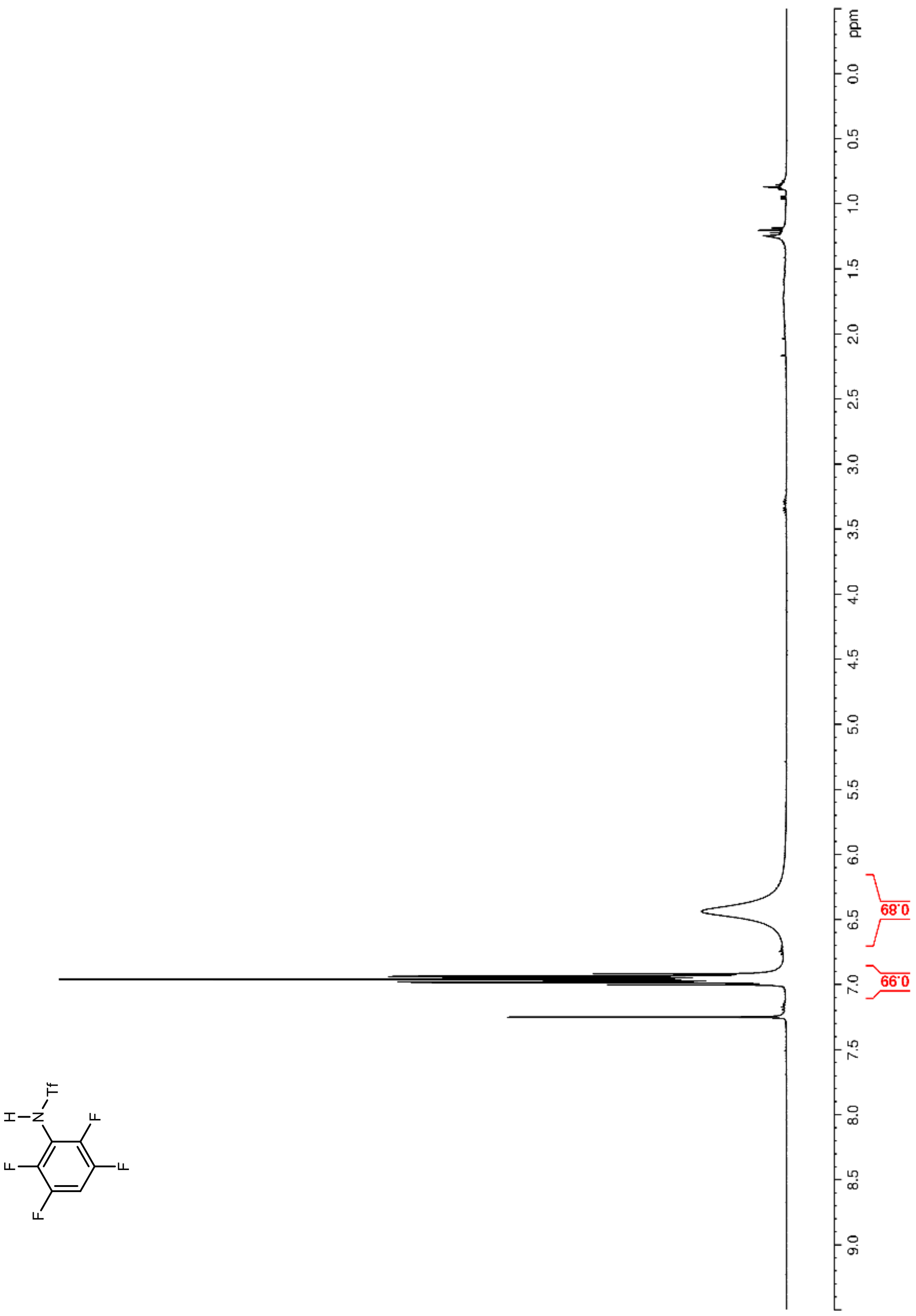

Figure 35. ^{13}C NMR (150 MHz, acetone-d_6) of **C6**

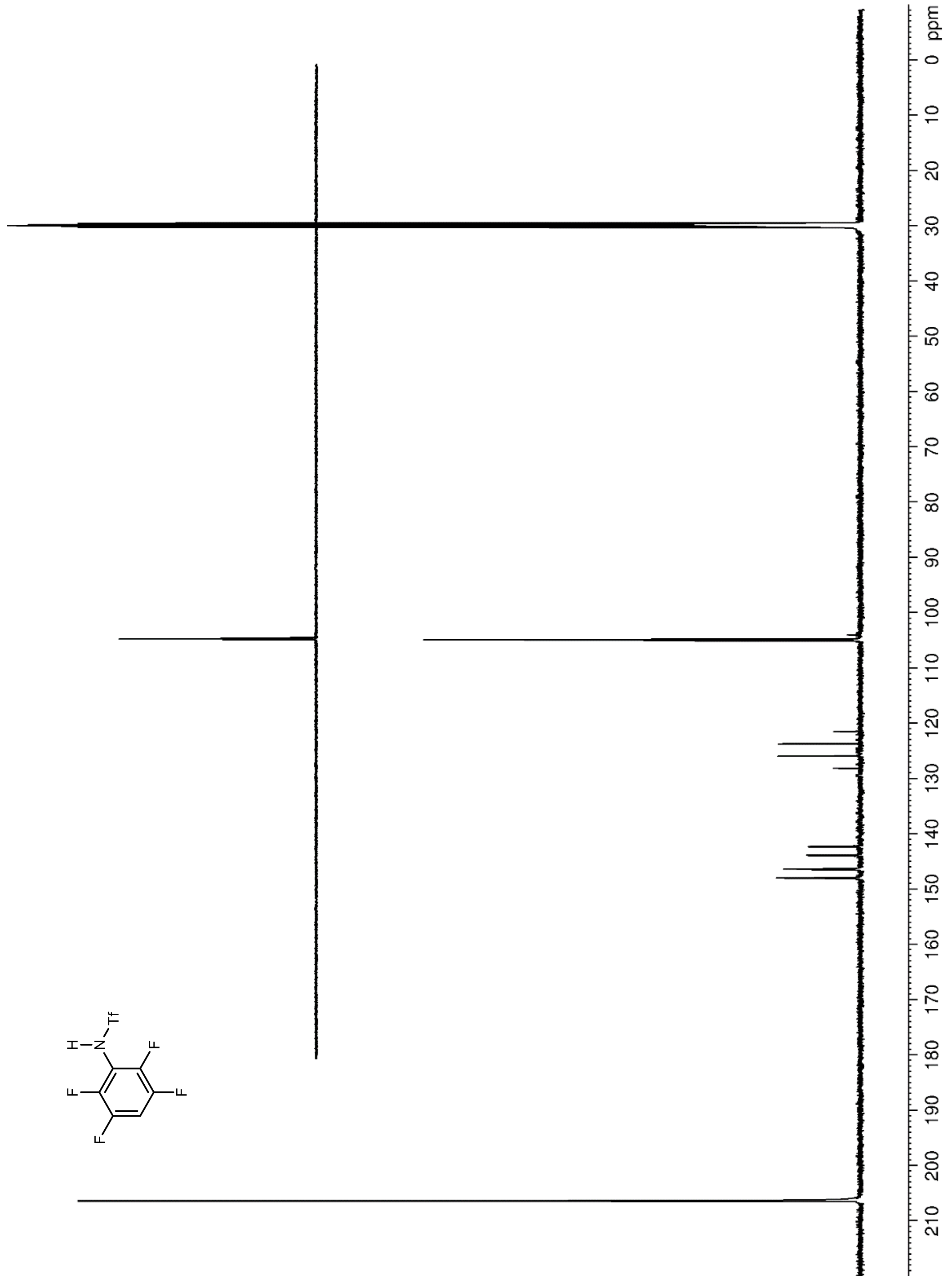

Figure 36. ^{19}F NMR (282 MHz, $CDCl_3$) of **C6**

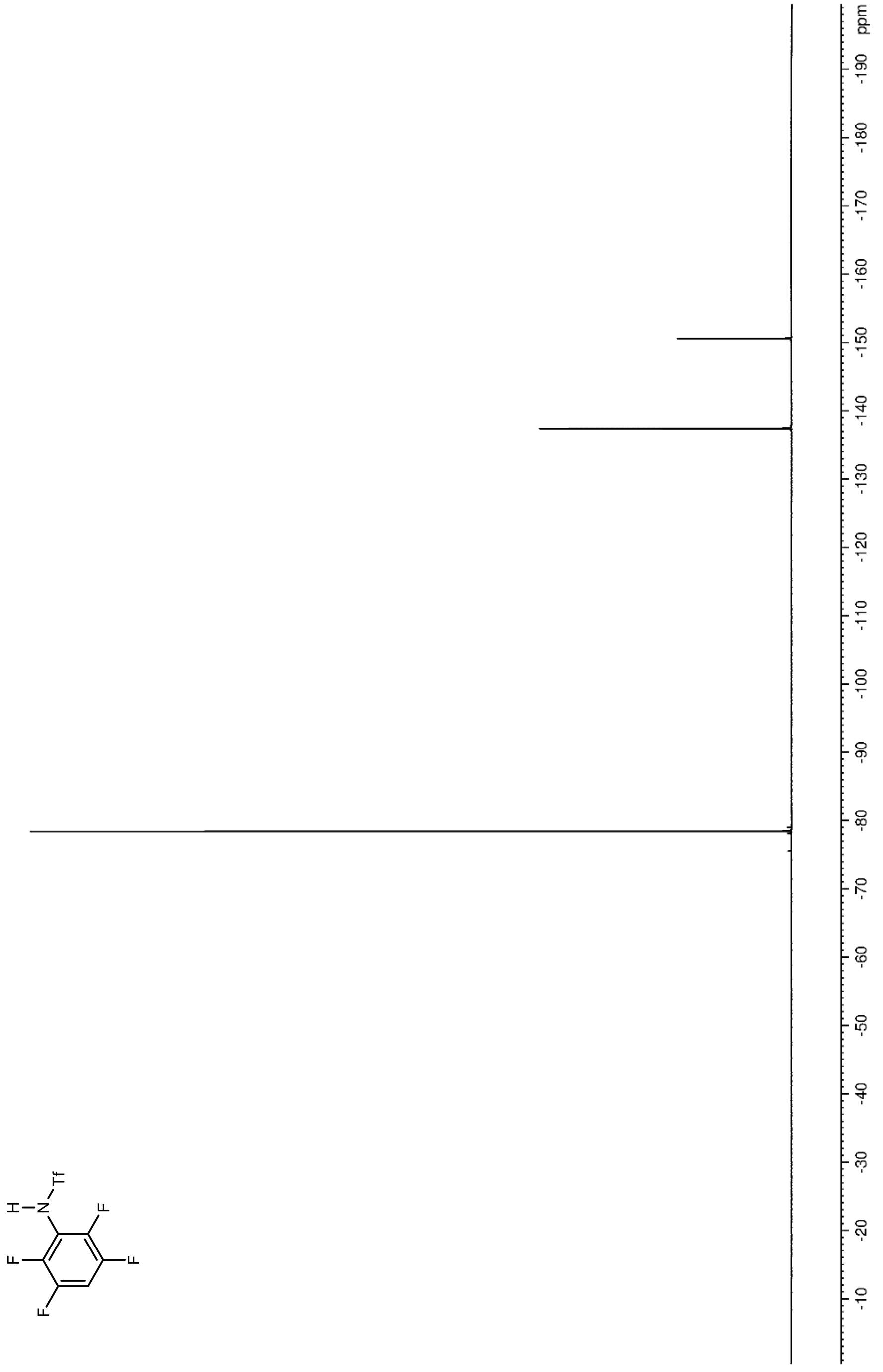

Figure 37. ^{1}H NMR (400 MHz, $CDCl_3$) of **D4**

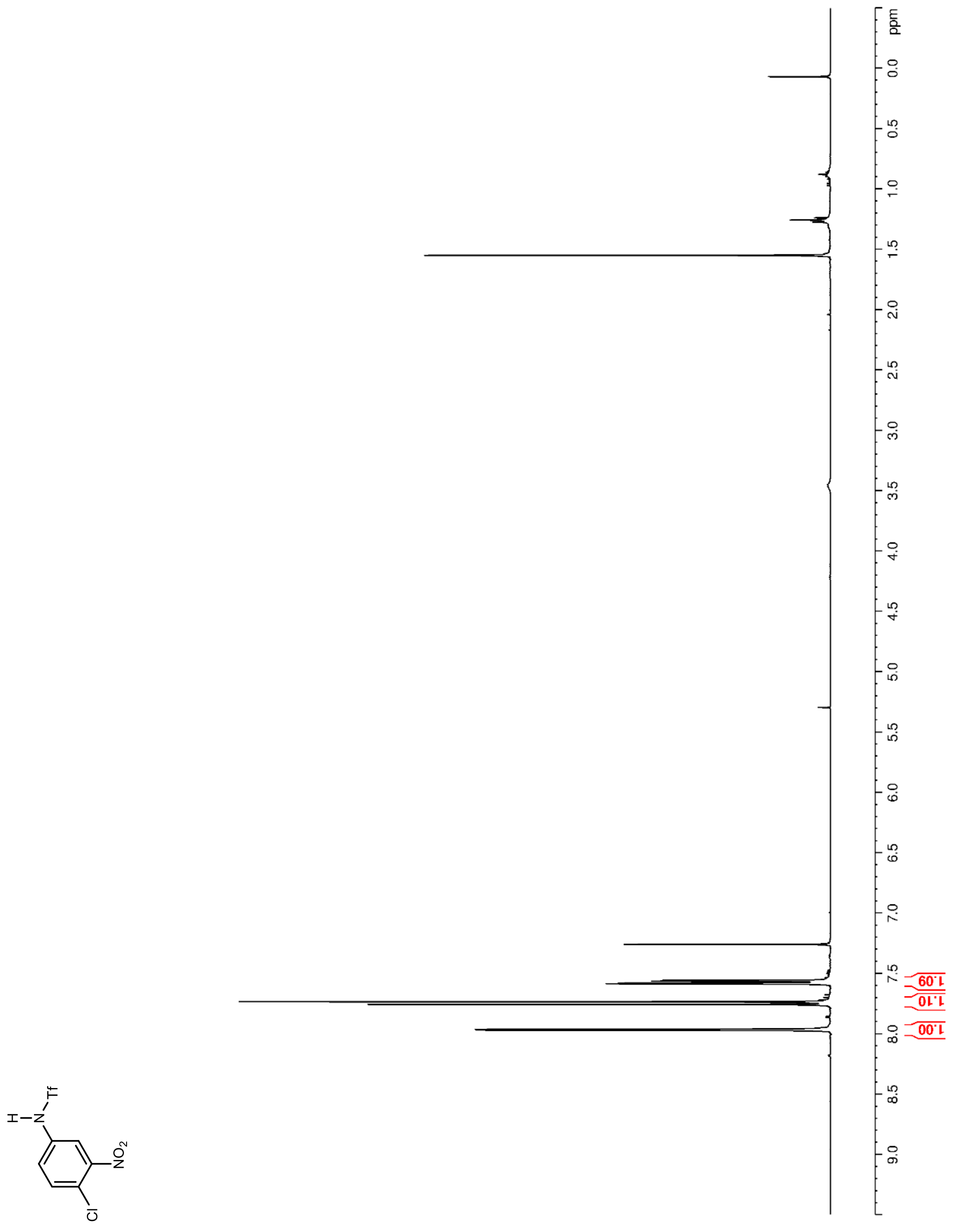

Figure 38. ^{13}C NMR (150 MHz, $CDCl_3$) of **D4**

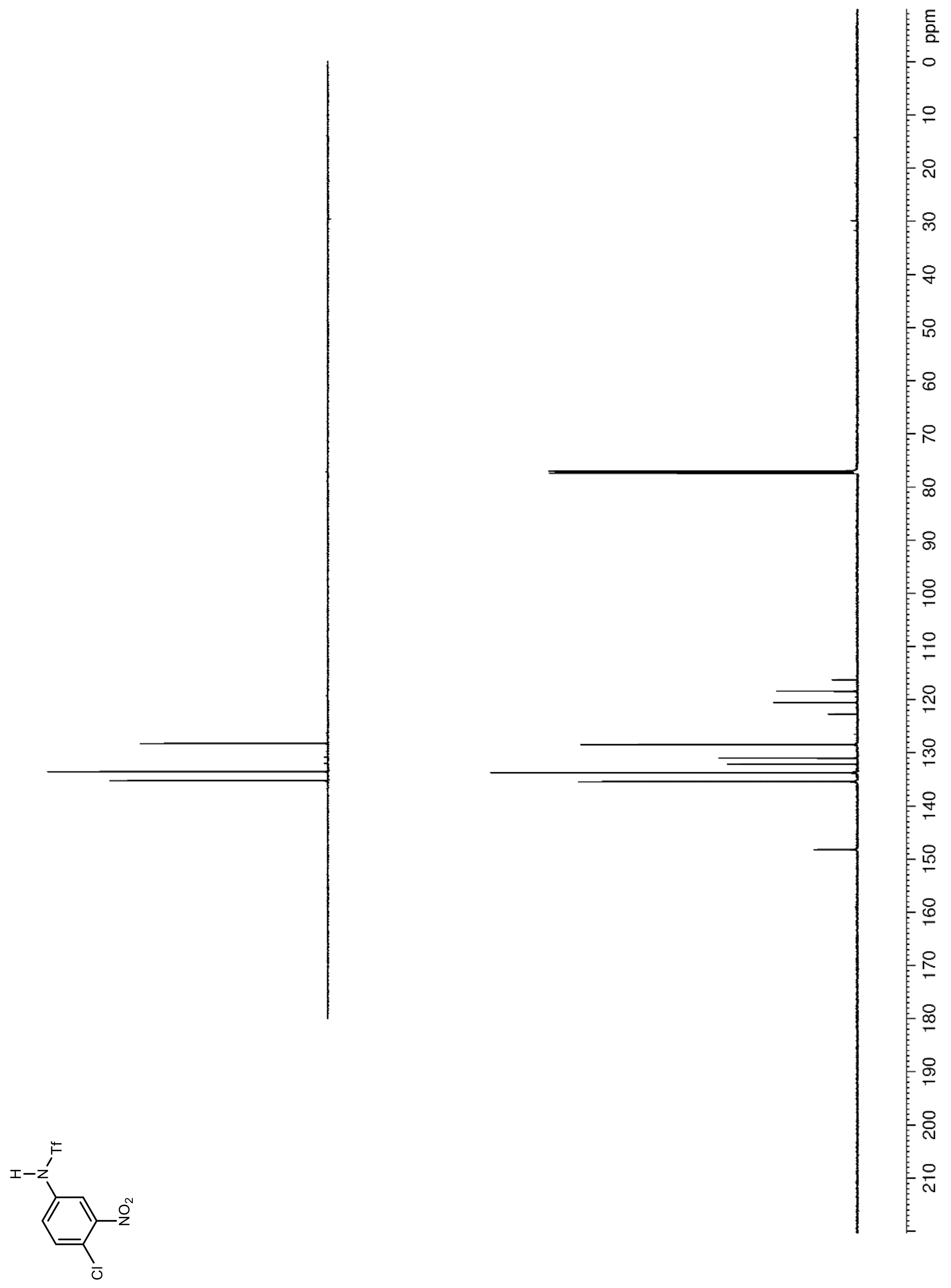

Figure 39. ^{19}F NMR (282 MHz, $CDCl_3$) of **D4**

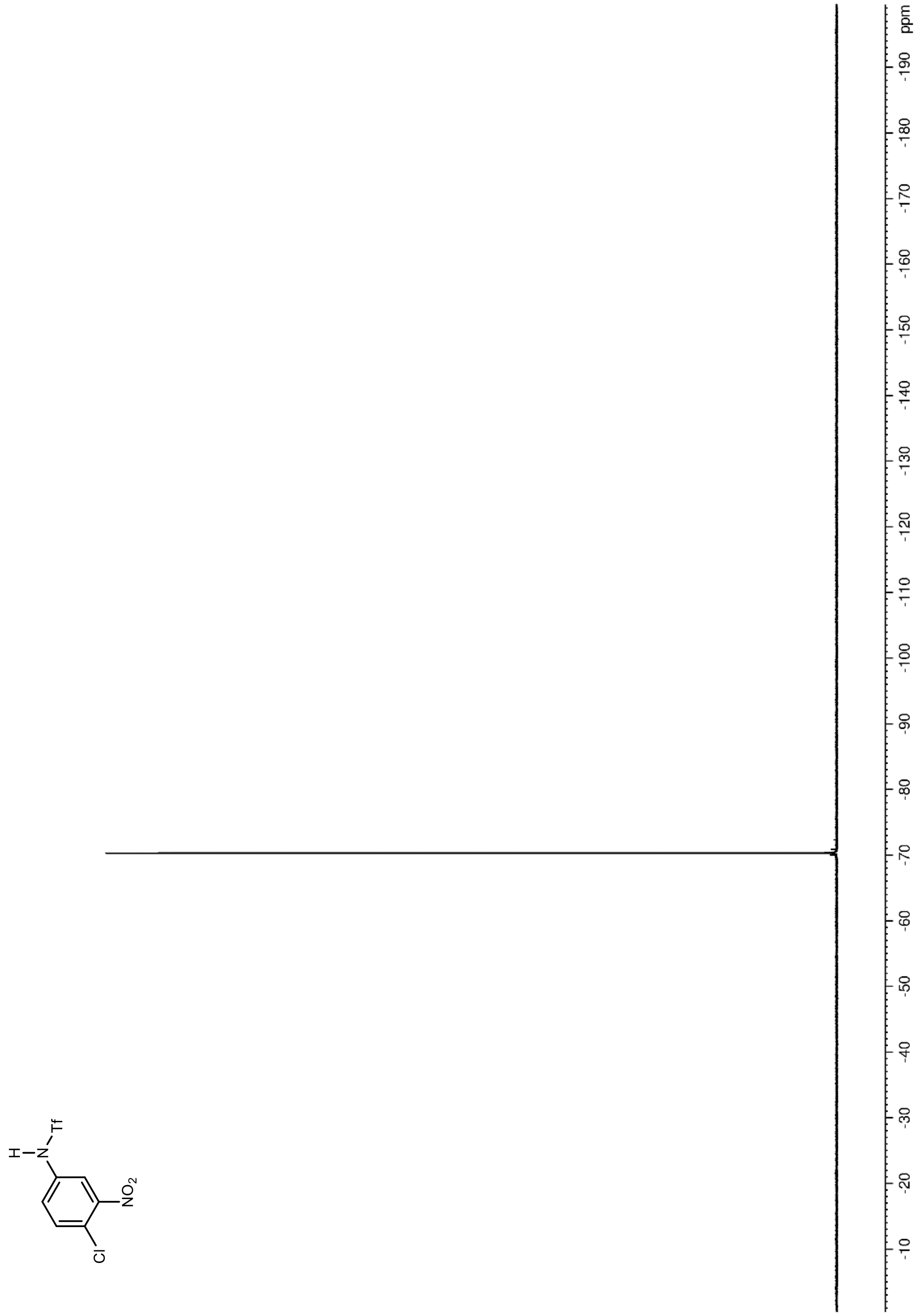

Figure 40. ^{1}H NMR (400 MHz, $CDCl_3$) of **D5**

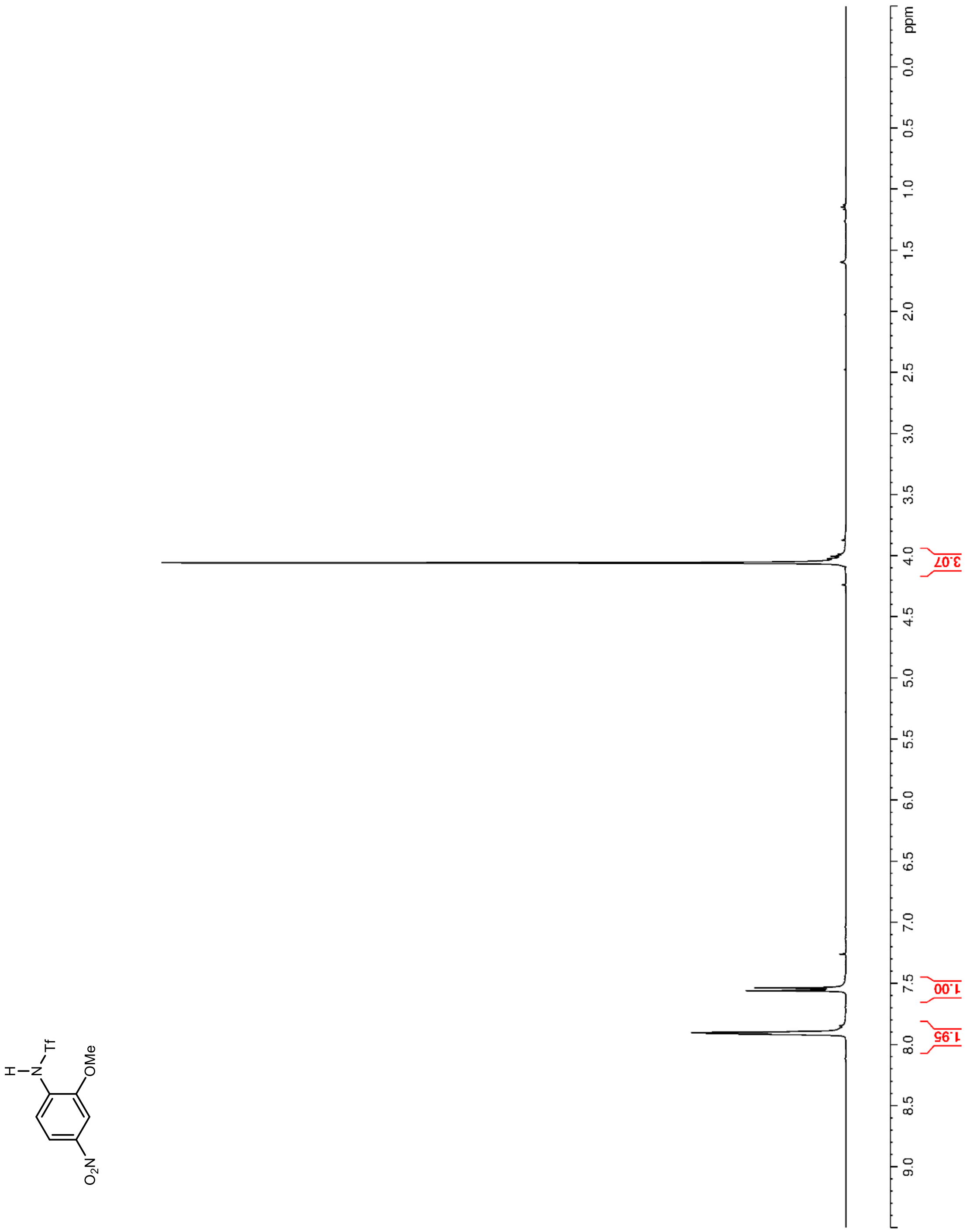

Figure 41. ^{13}C NMR (150 MHz, $CDCl_3$) of **D5**

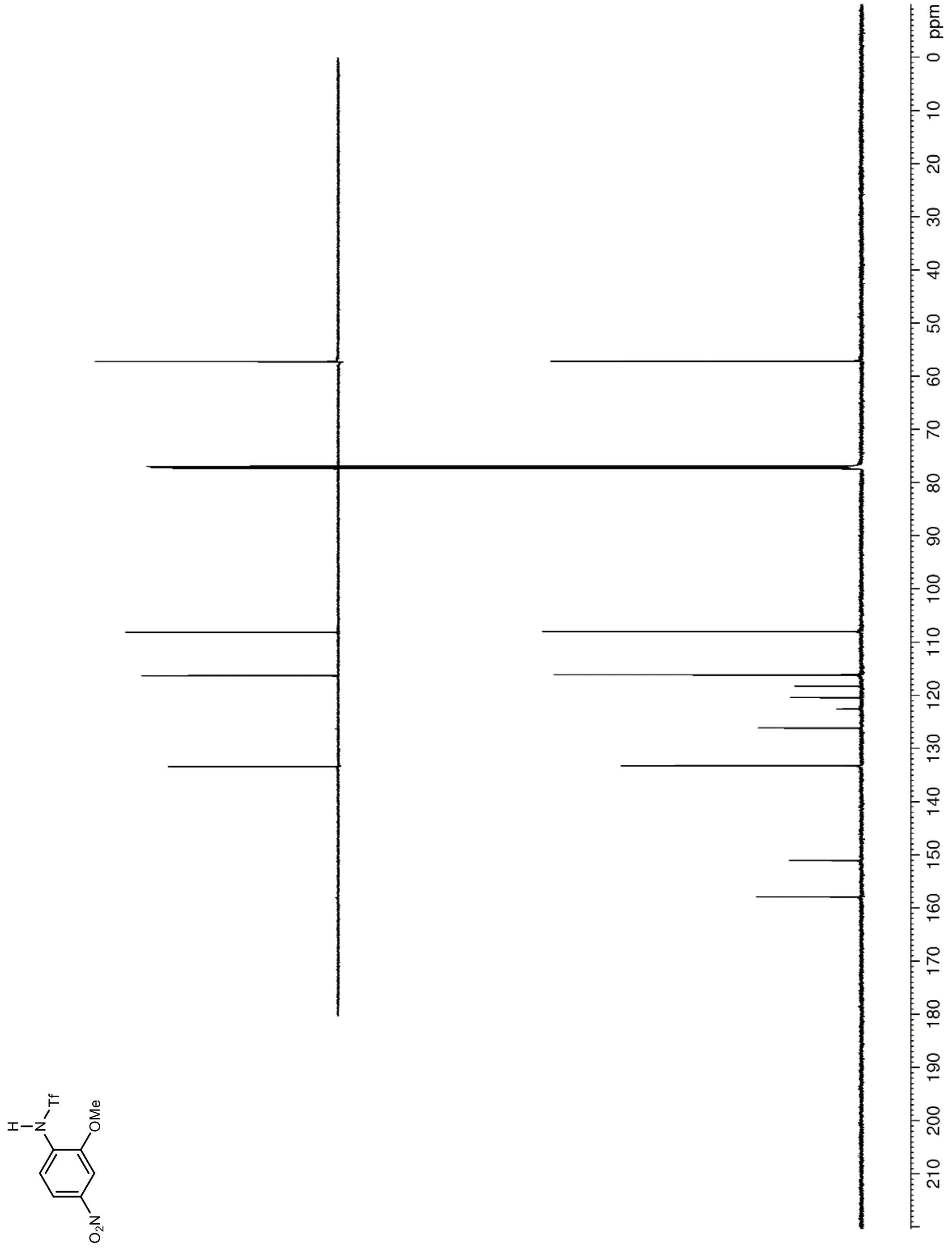

Figure 42. ^{19}F NMR (282 MHz, $CDCl_3$) of **D5**

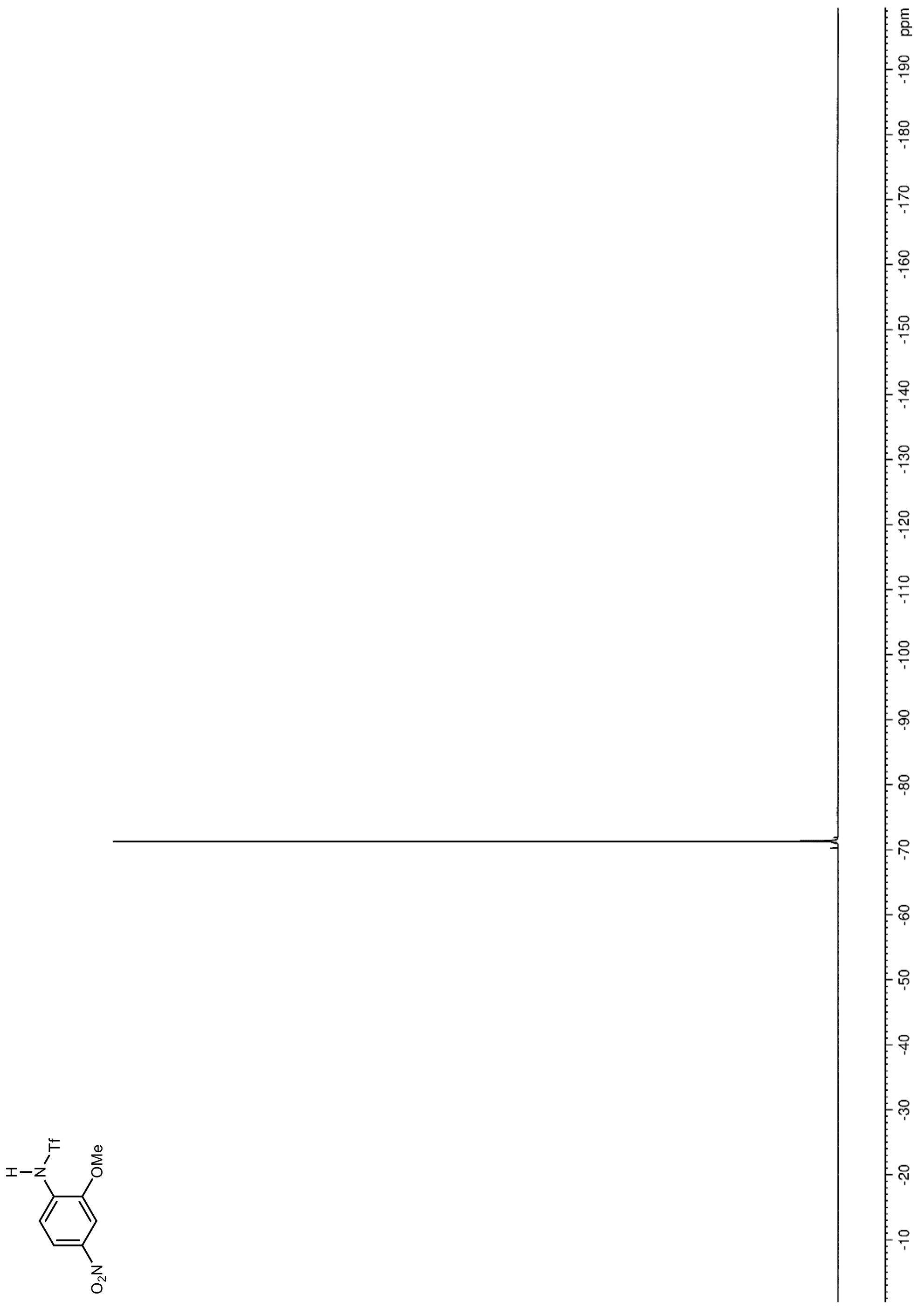

Figure 43. ^{1}H NMR (400 MHz, $CDCl_3$) of **D6**

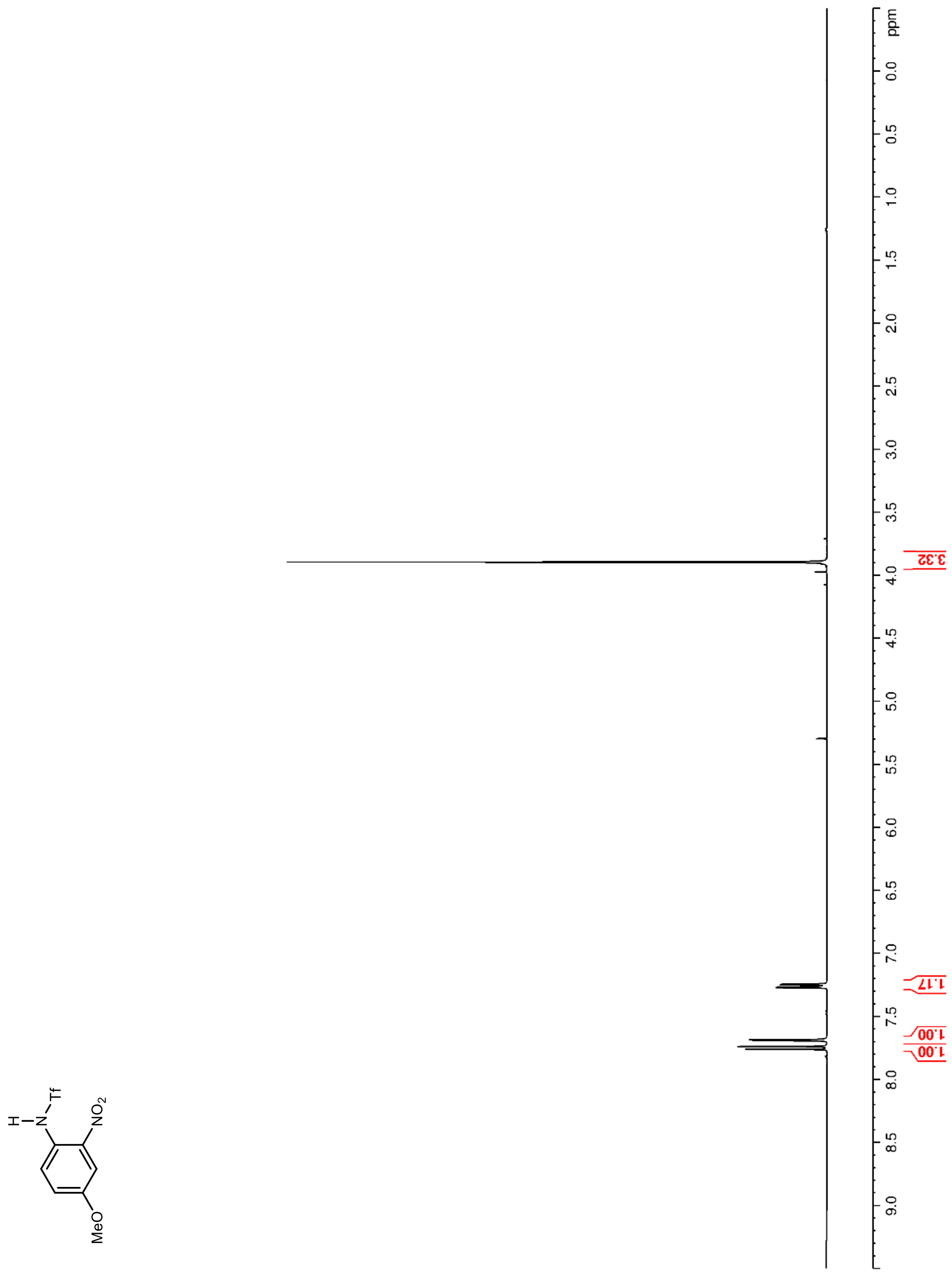

Figure 44. ^{13}C NMR (150 MHz, $CDCl_3$) of **D6**

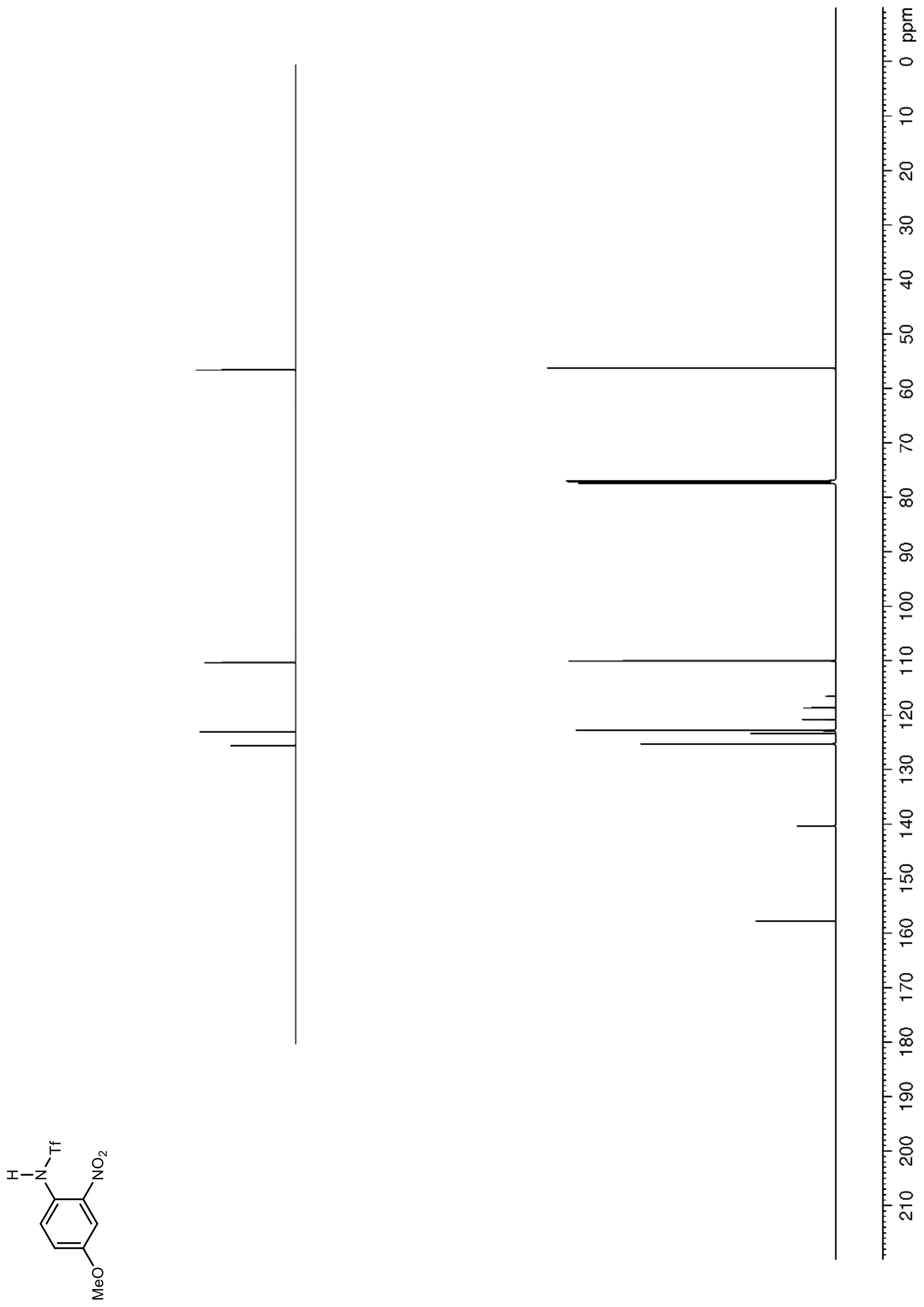

Figure 45. ^{19}F NMR (282 MHz, $CDCl_3$) of **D6**

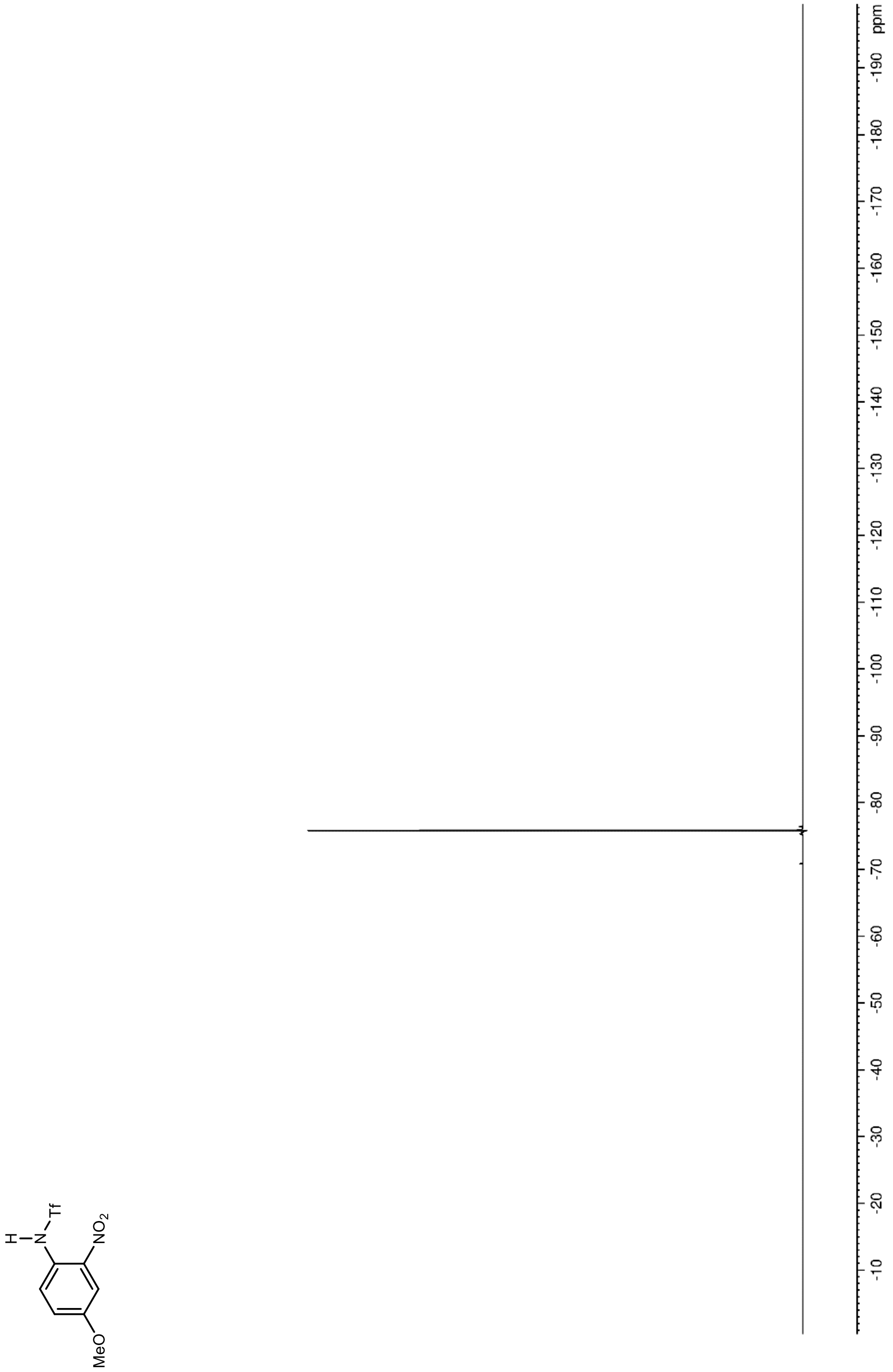

Figure 46. ^{1}H NMR (400 MHz, $CDCl_3$) of **D7**

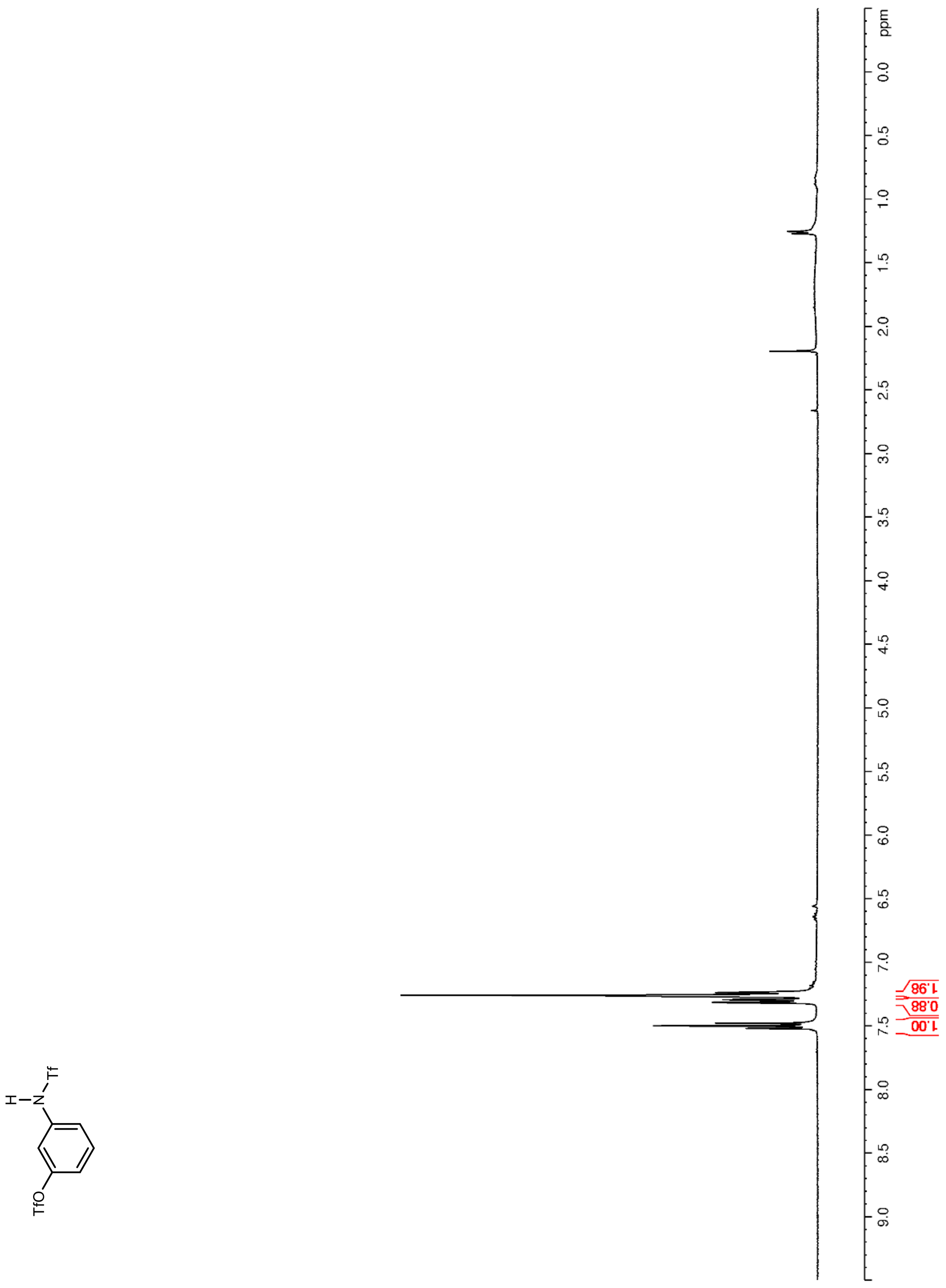

Figure 47. ^{13}C NMR (150 MHz, $CDCl_3$) of **D7**

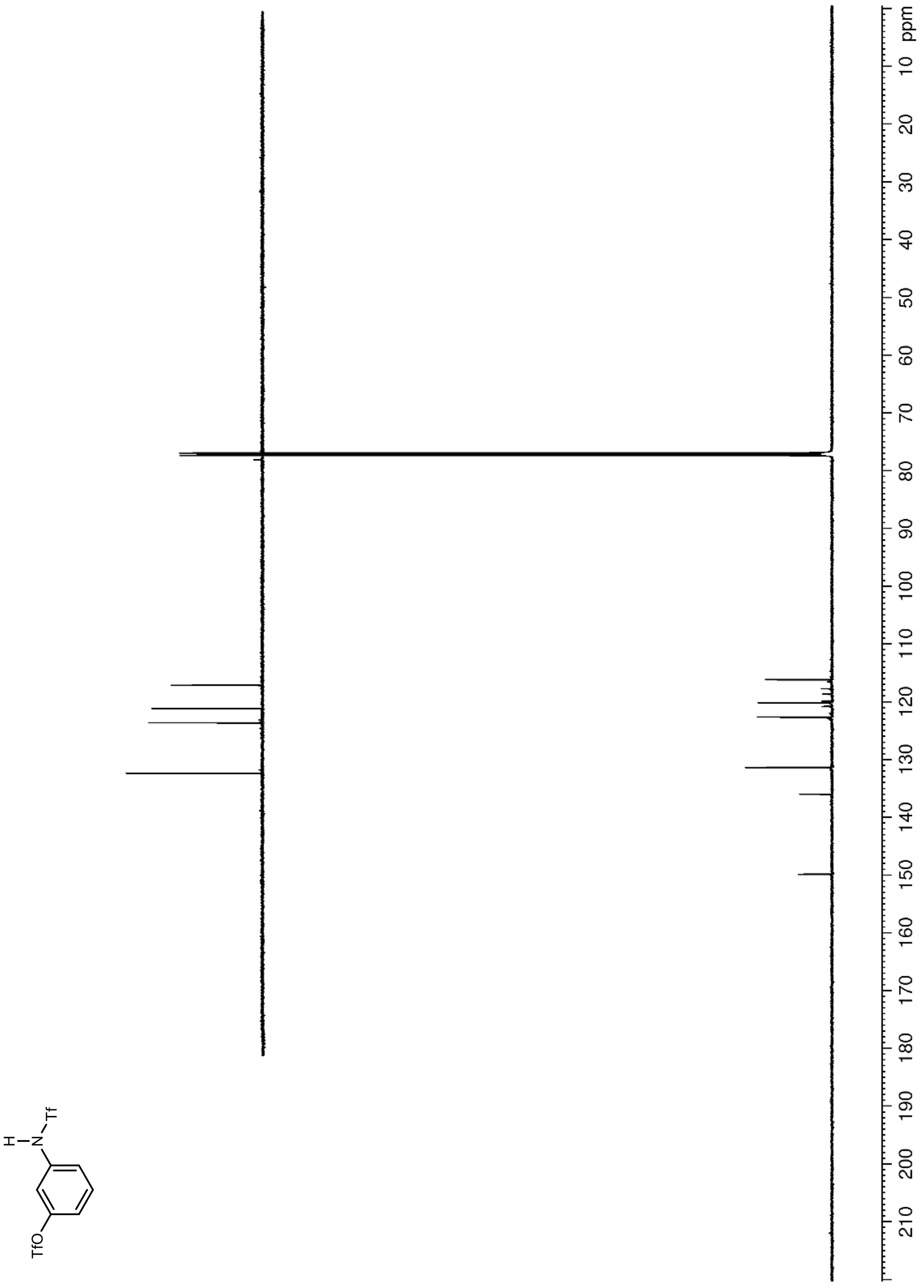

Figure 48. ^{19}F NMR (282 MHz, $CDCl_3$) of **D7**

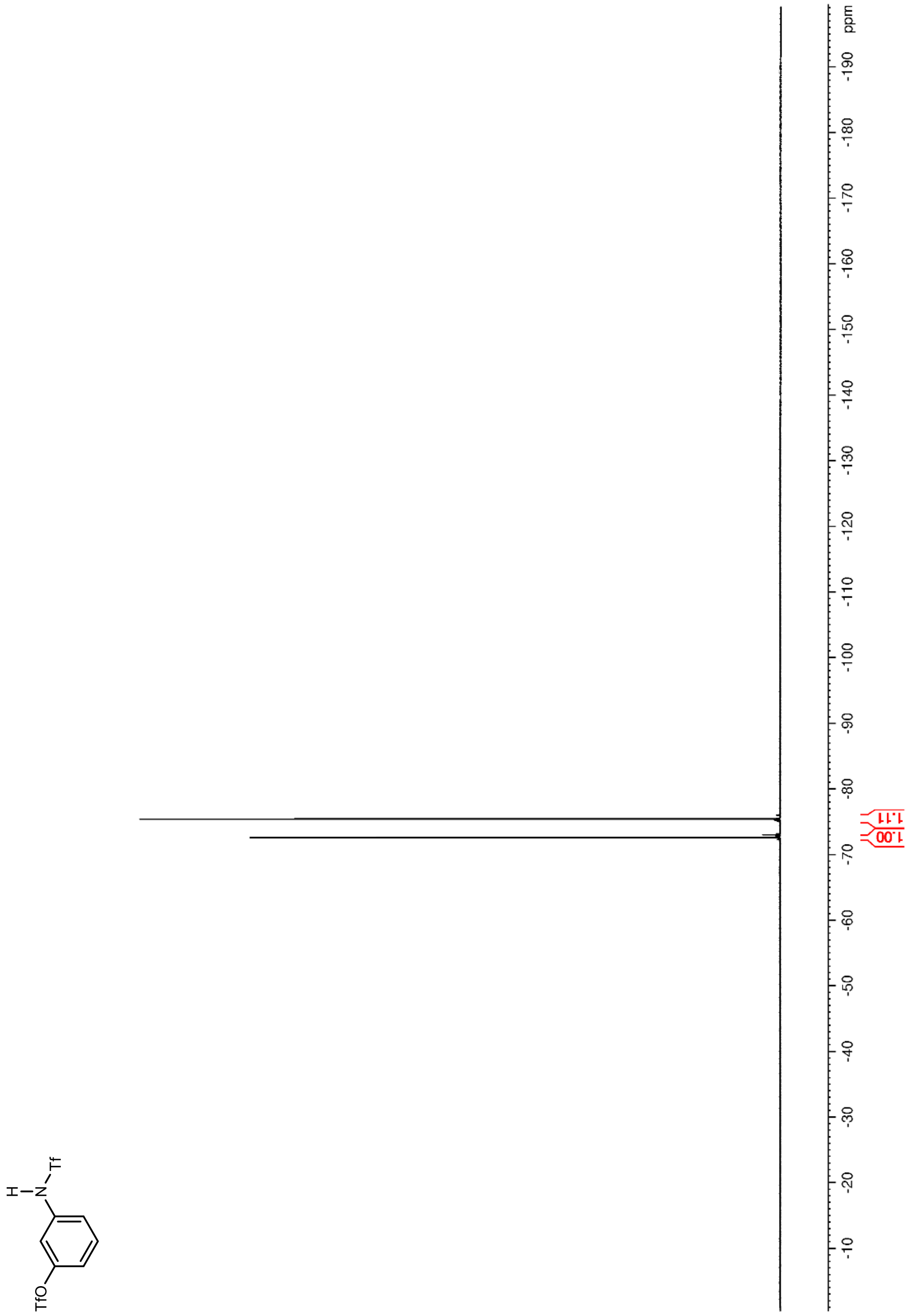

Figure 49. ^{1}H NMR (400 MHz, $CDCl_3$) of **D8**

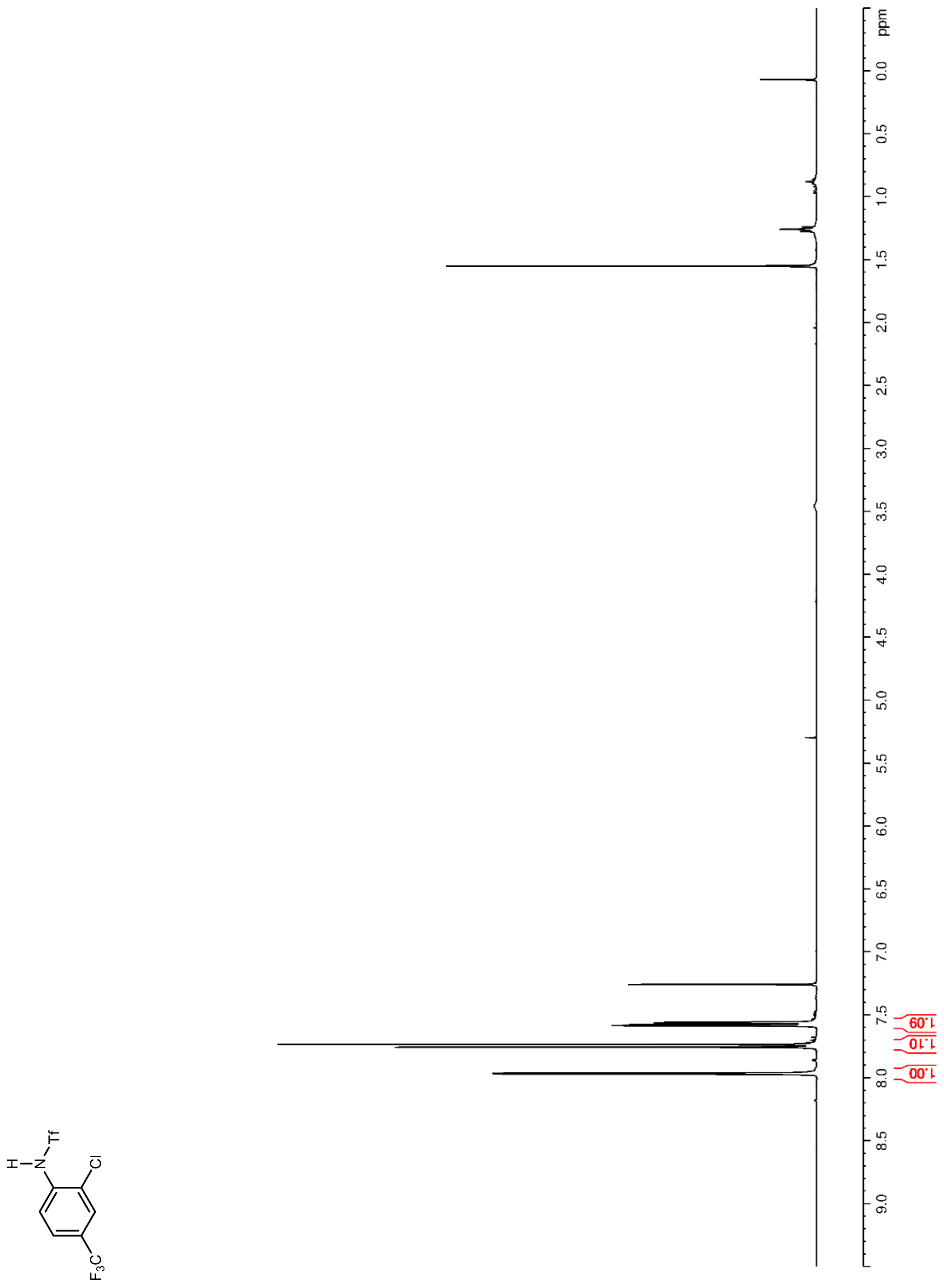

Figure 50. ^{13}C NMR (150 MHz, $CDCl_3$) of **D8**

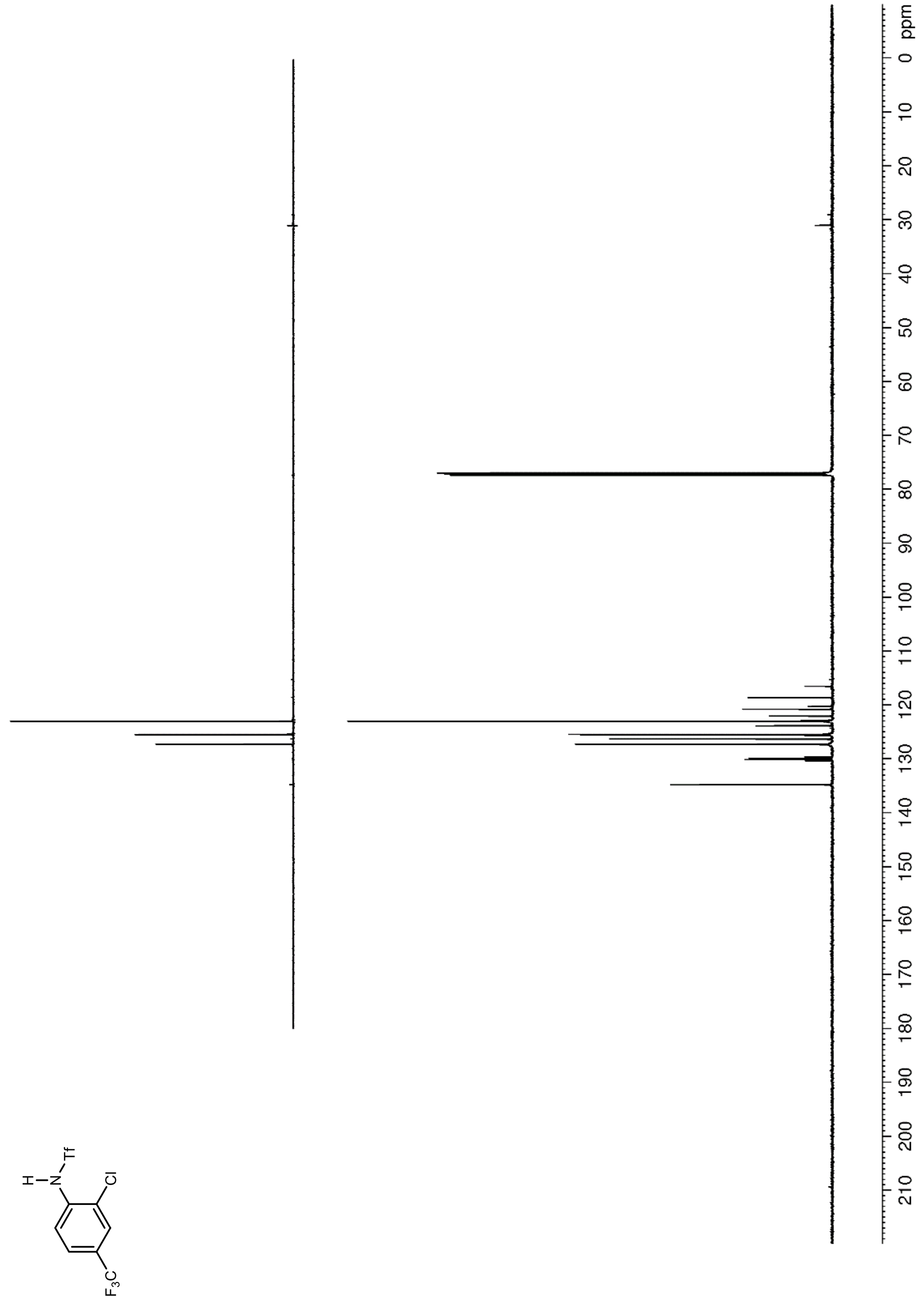

Figure 51. ^{19}F NMR (282 MHz, $CDCl_3$) of **D8**

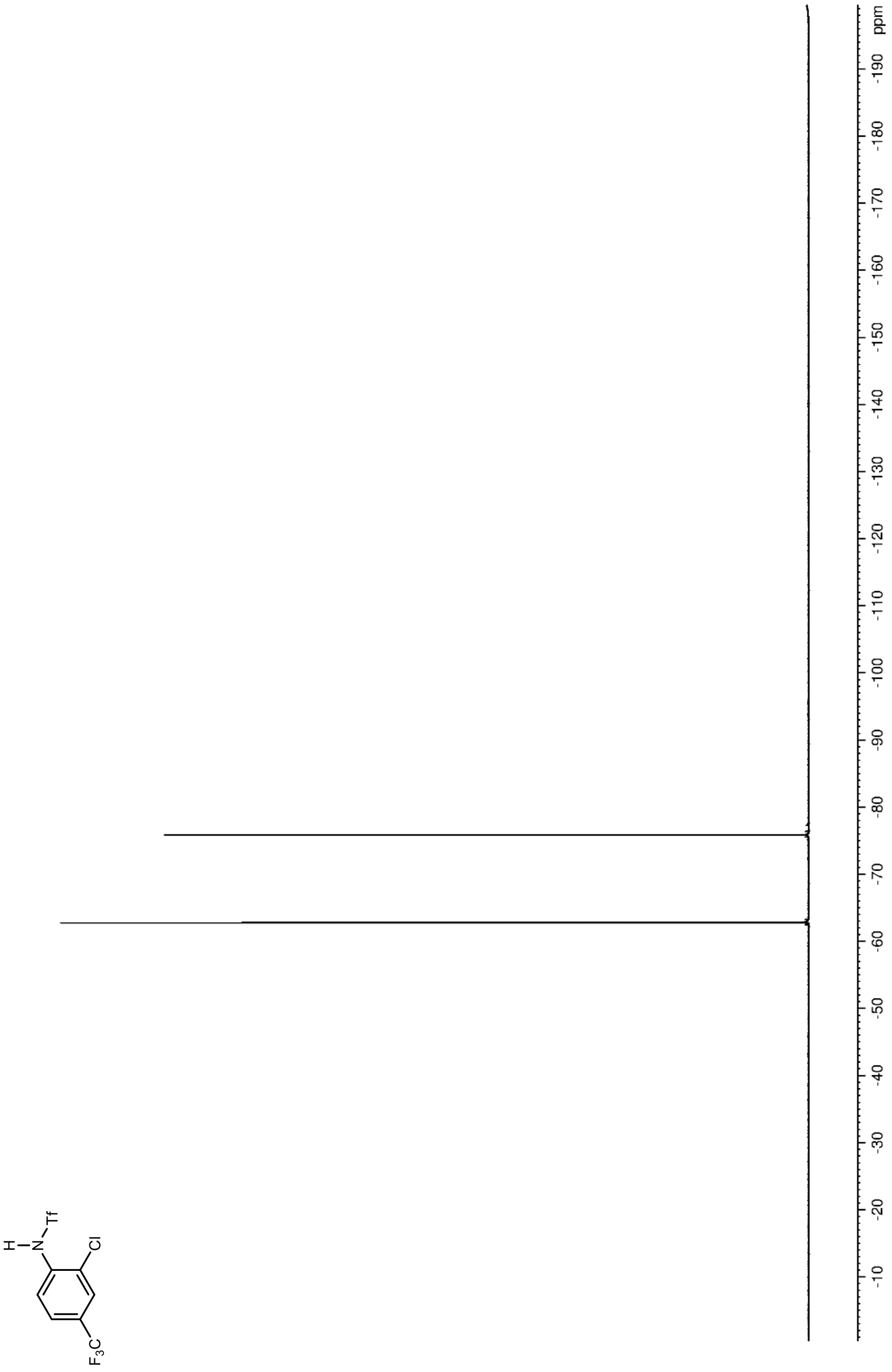

Figure 52. ^{1}H NMR (400 MHz, $CDCl_3$) of **E1**

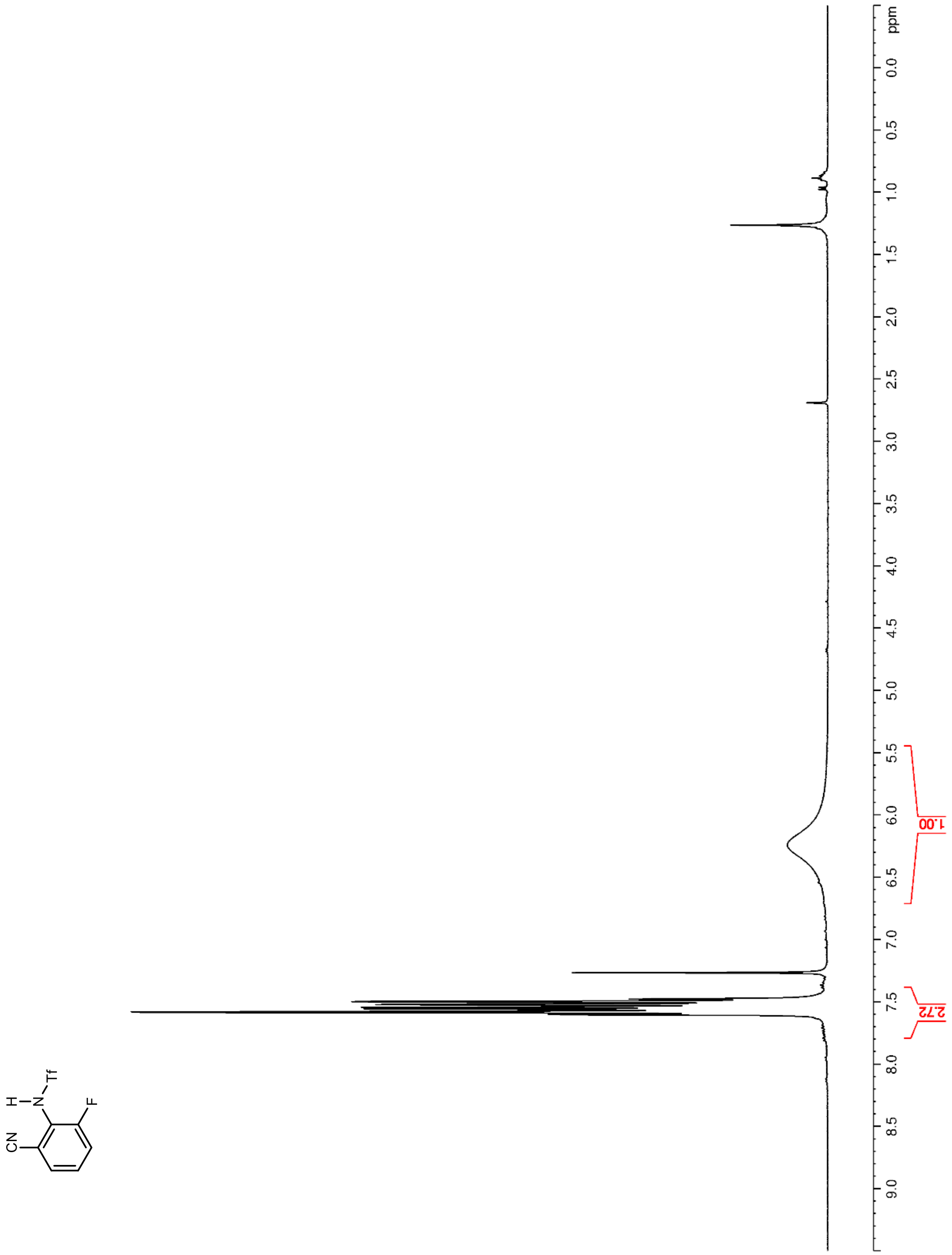

Figure 53. ^{13}C NMR (150 MHz, $CDCl_3$) of **E1**

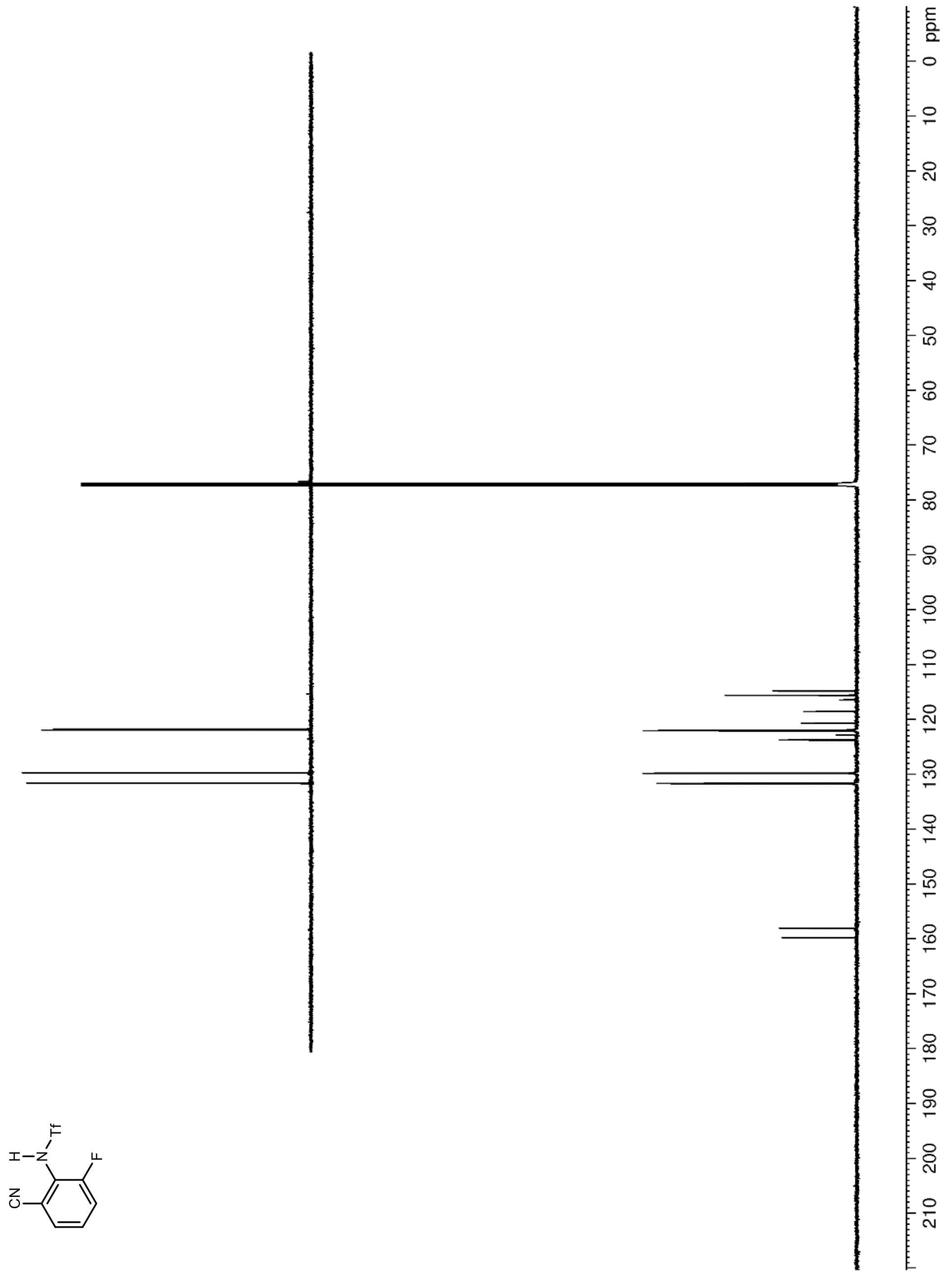

Figure 54. ^{19}F NMR (282 MHz, $CDCl_3$) of **E1**

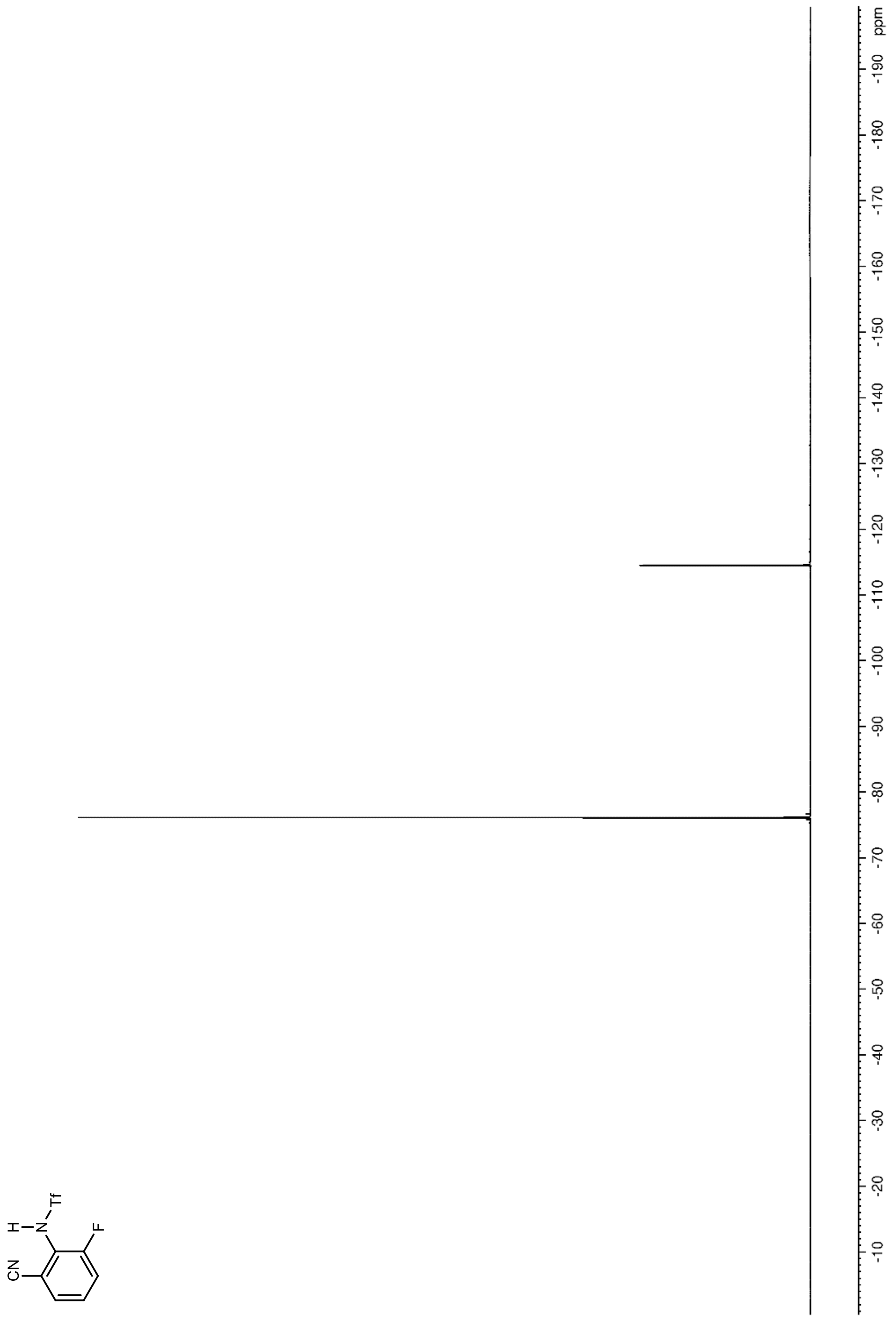

Figure 55. ^{1}H NMR (400 MHz, acetone-d_6) of **E2**

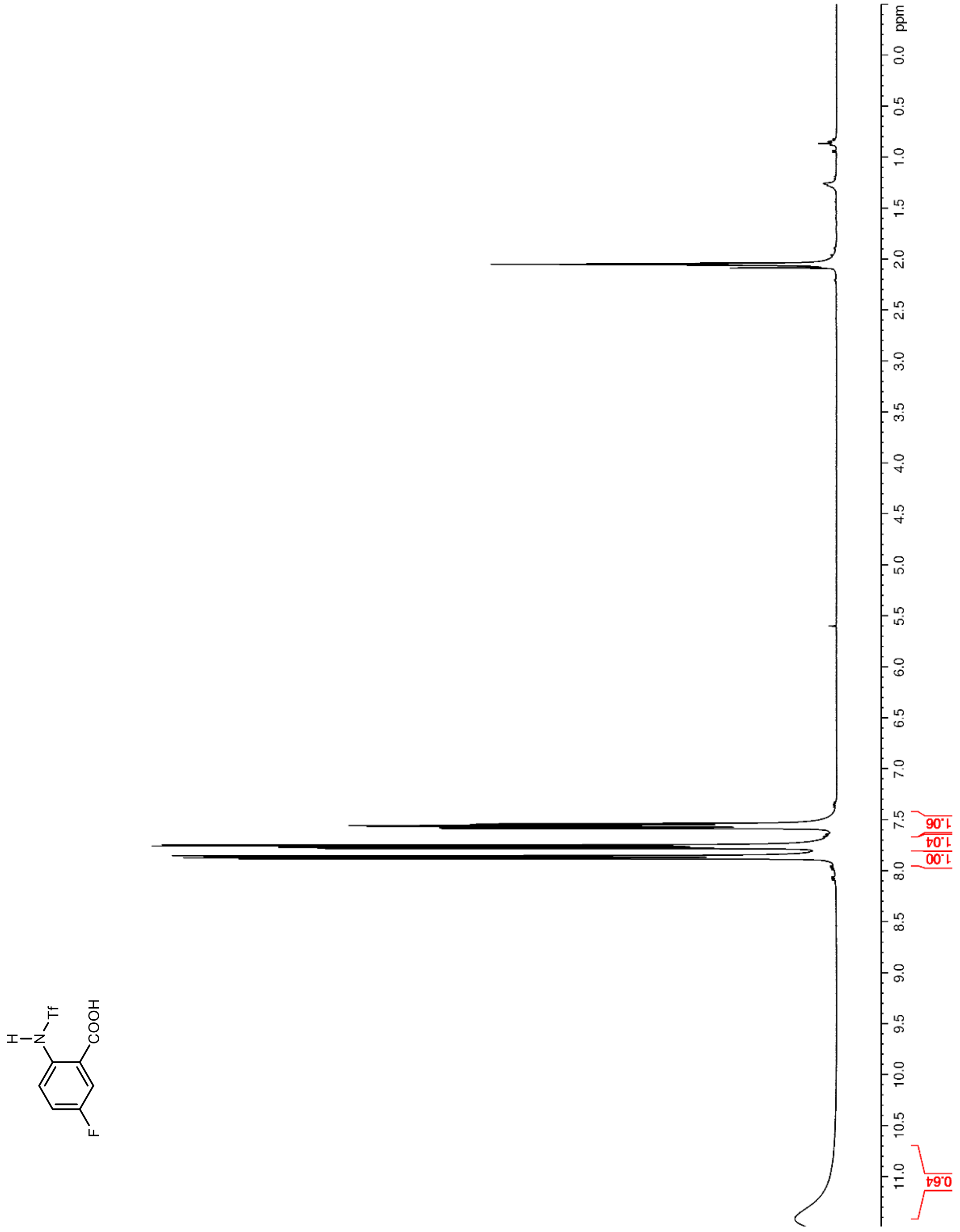

Figure 56. ^{13}C NMR (150 MHz, acetone-d_6) of **E2**

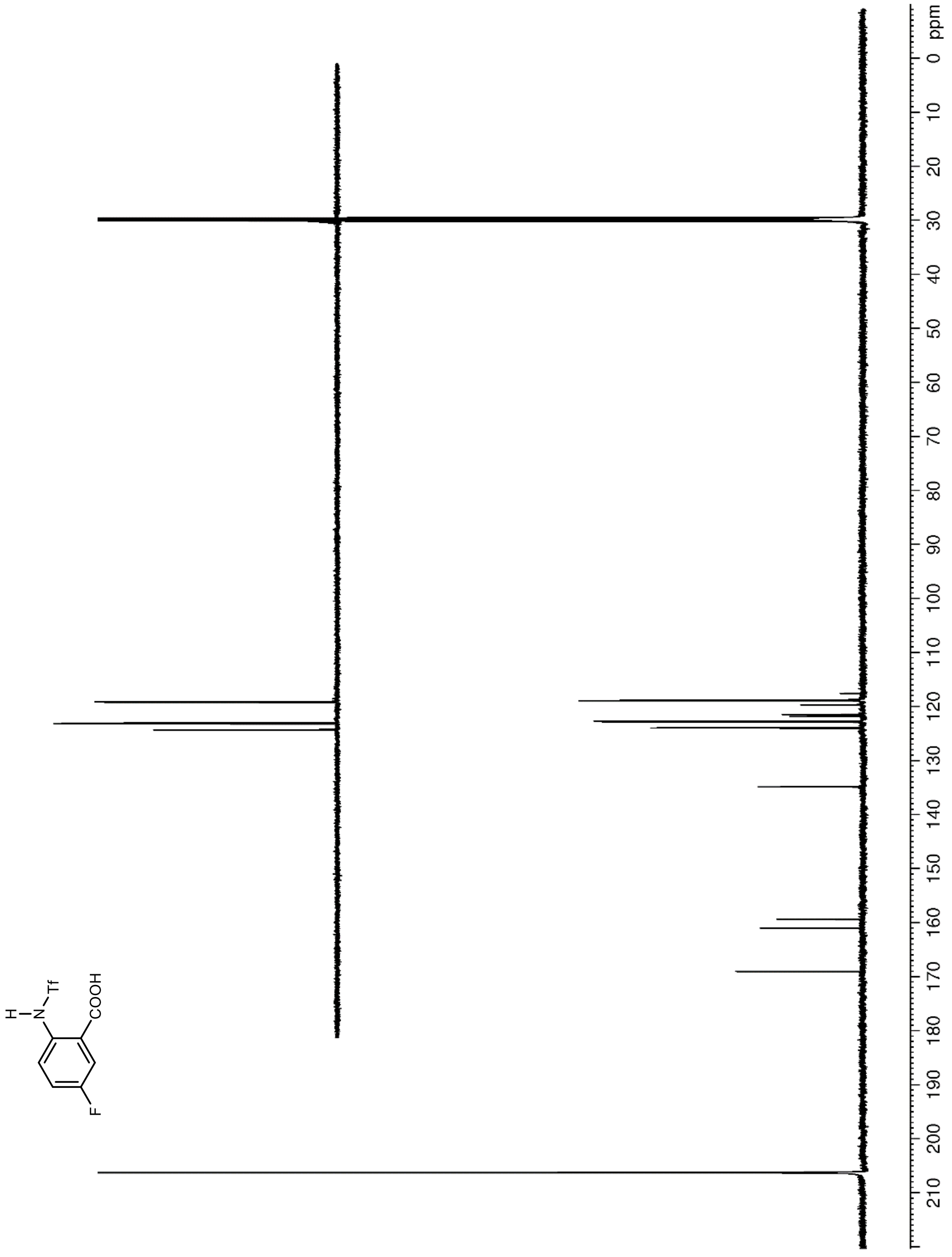

Figure 57. ^{19}F NMR (282 MHz, acetone-d_6) of **E2**

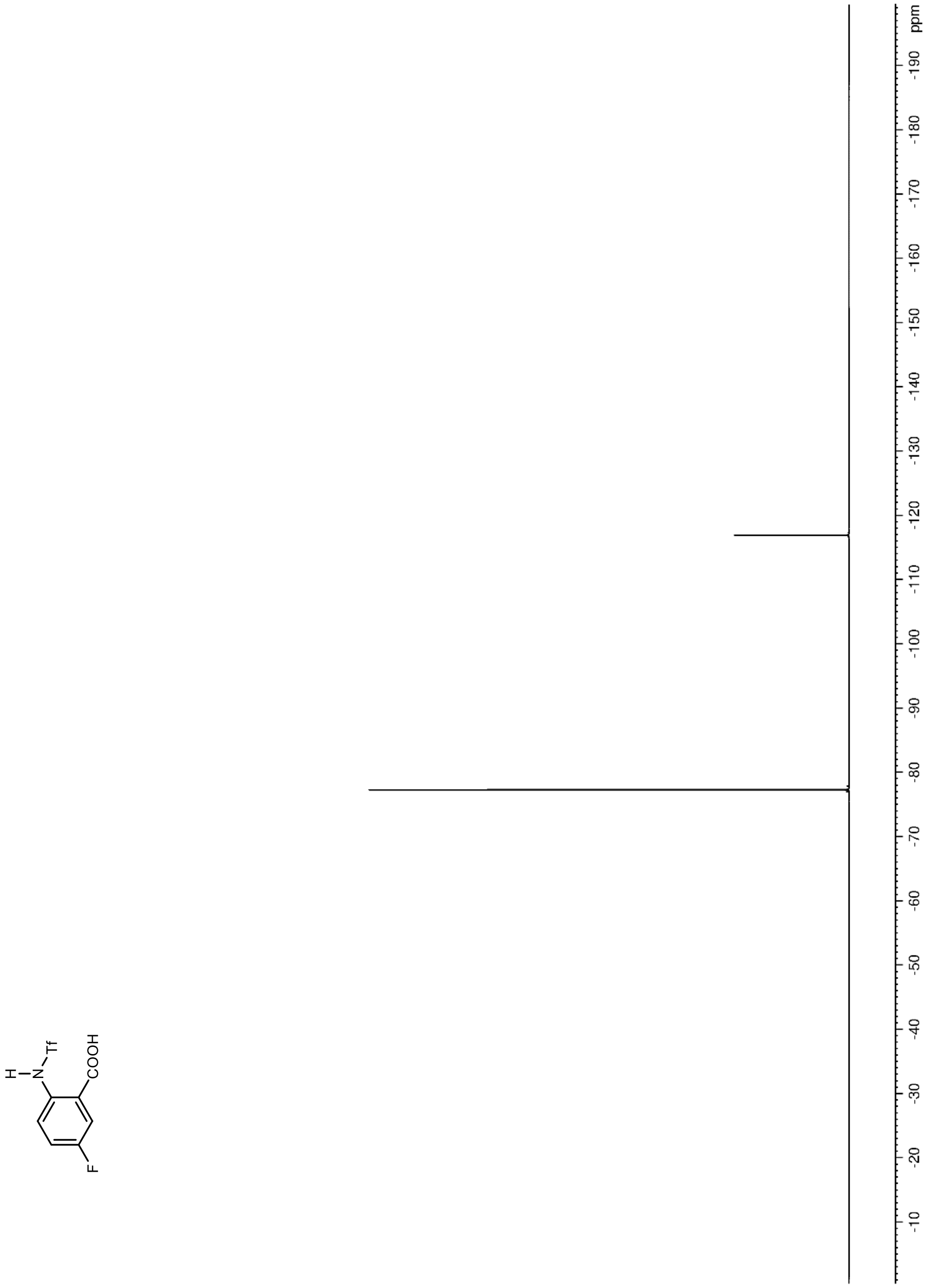

Figure 58. ^{1}H NMR (400 MHz, $CDCl_3$) of **E3**

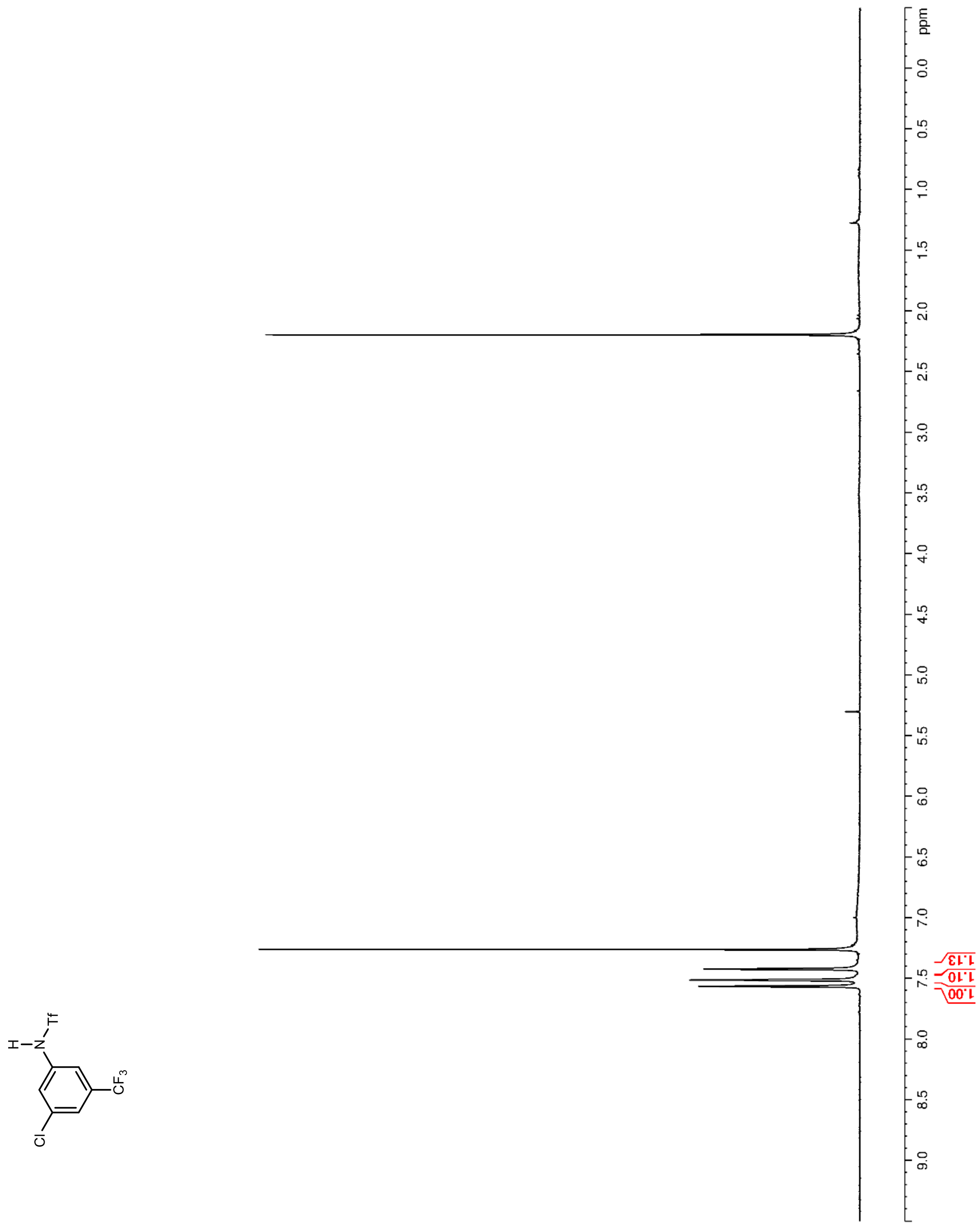

Figure 59. ^{13}C NMR (150 MHz, $CDCl_3$) of **E3**

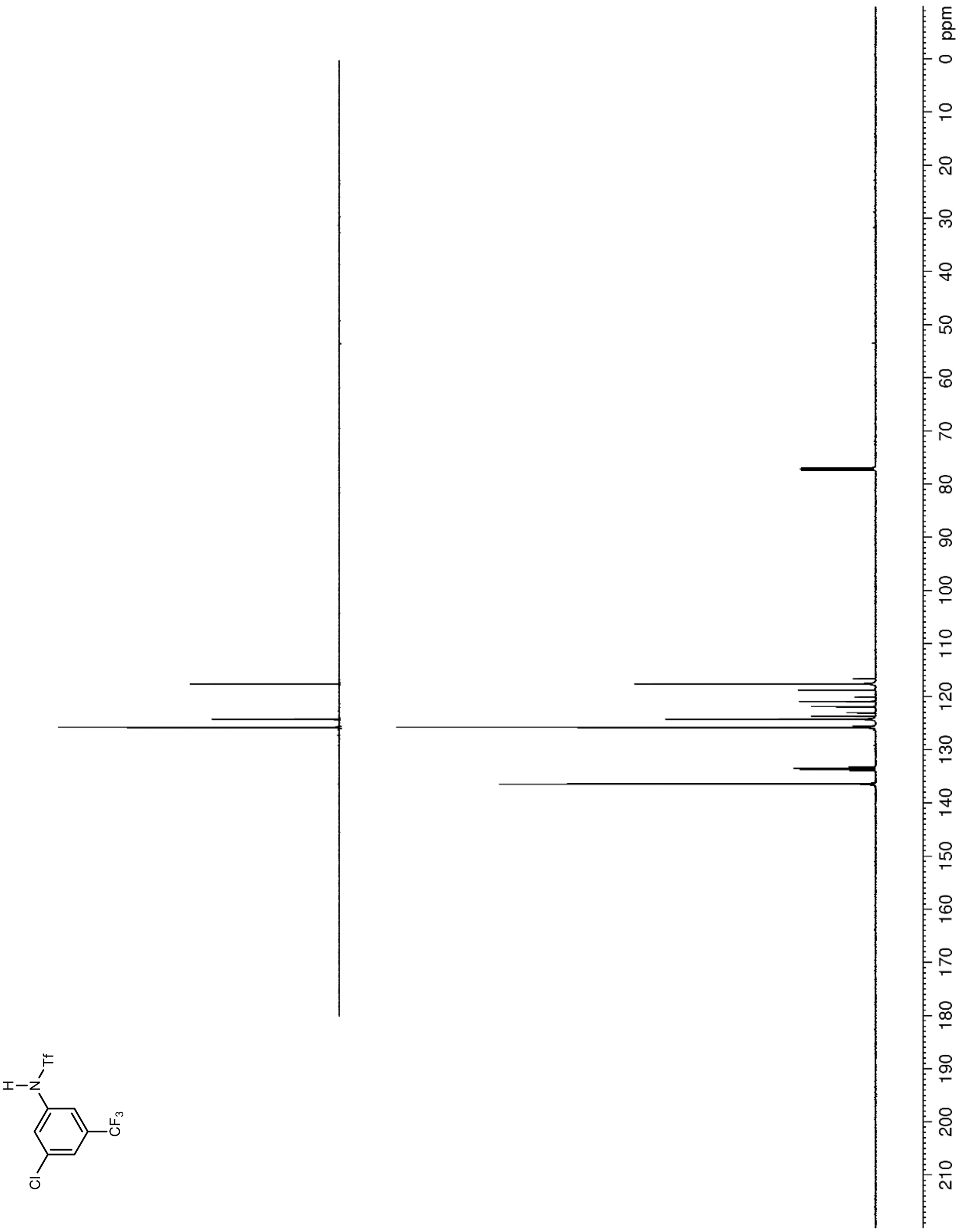

Figure 60. ^{19}F NMR (282 MHz, $CDCl_3$) of **E3**

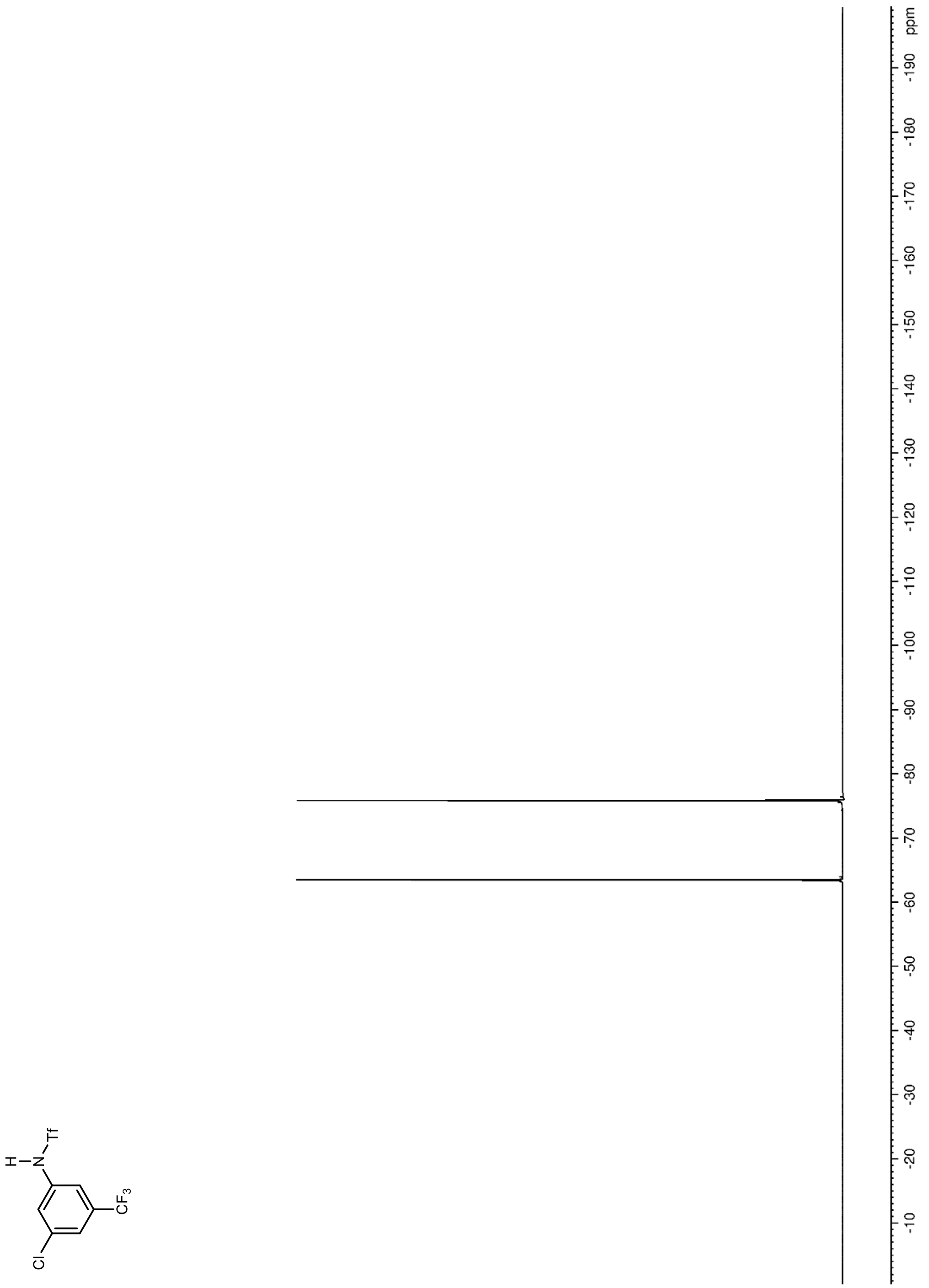

Figure 61. ^{1}H NMR (400 MHz, acetone-d_6) of **E4**

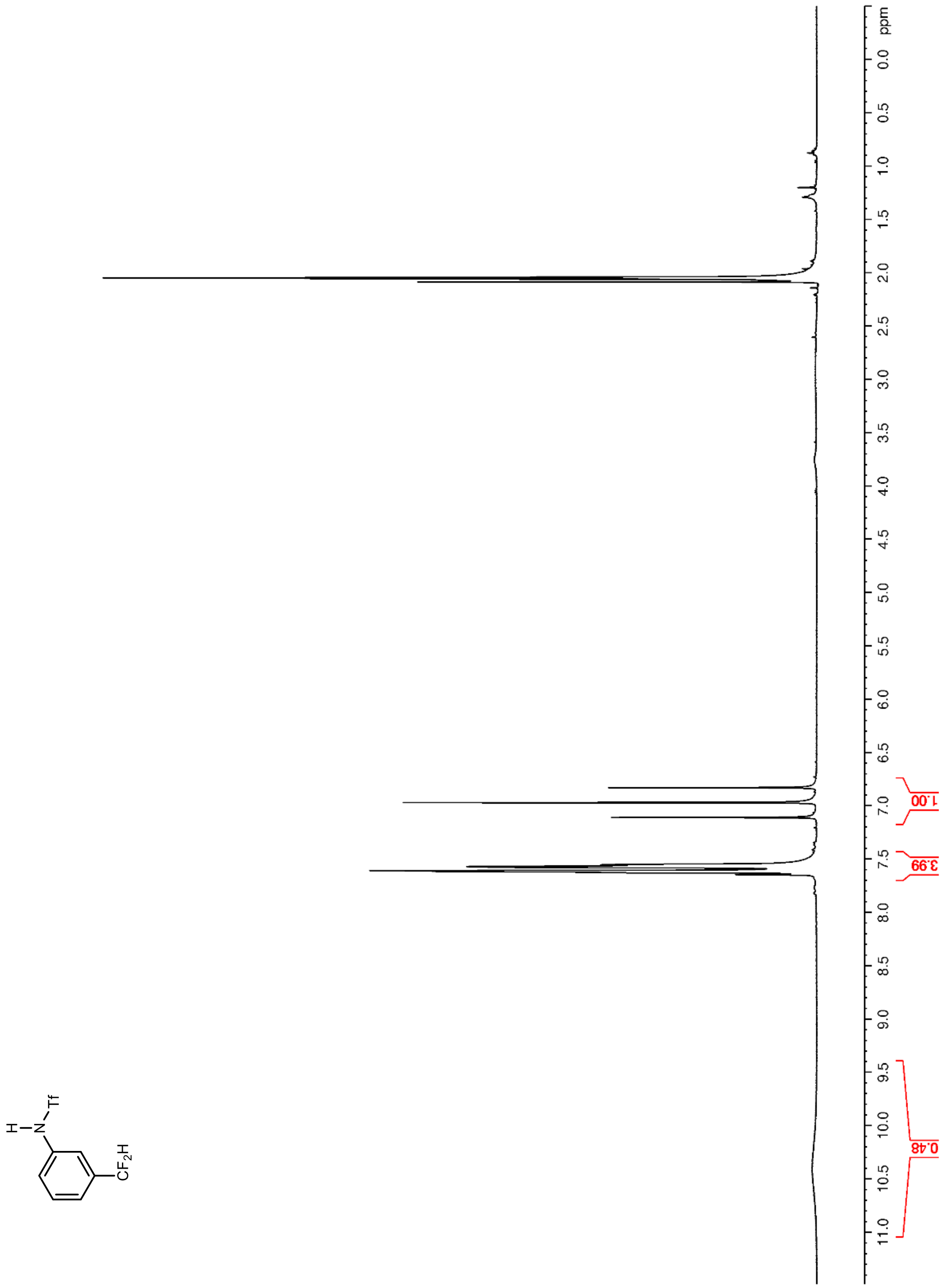

Figure 62. ^{13}C NMR (150 MHz, $CDCl_3$) of **E4**

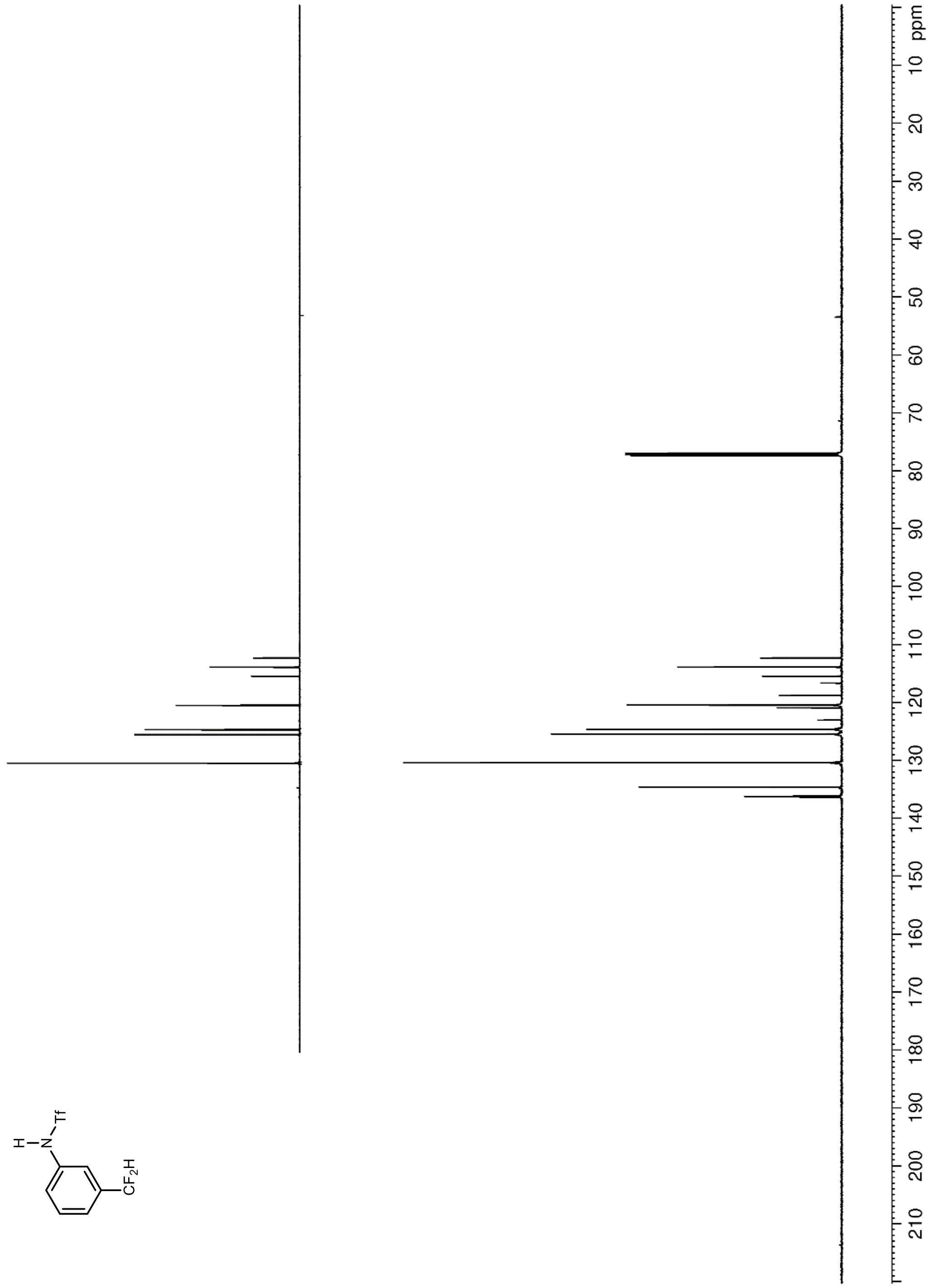

Figure 63. ^{19}F NMR (282 MHz, acetone-d_6) of **E4**

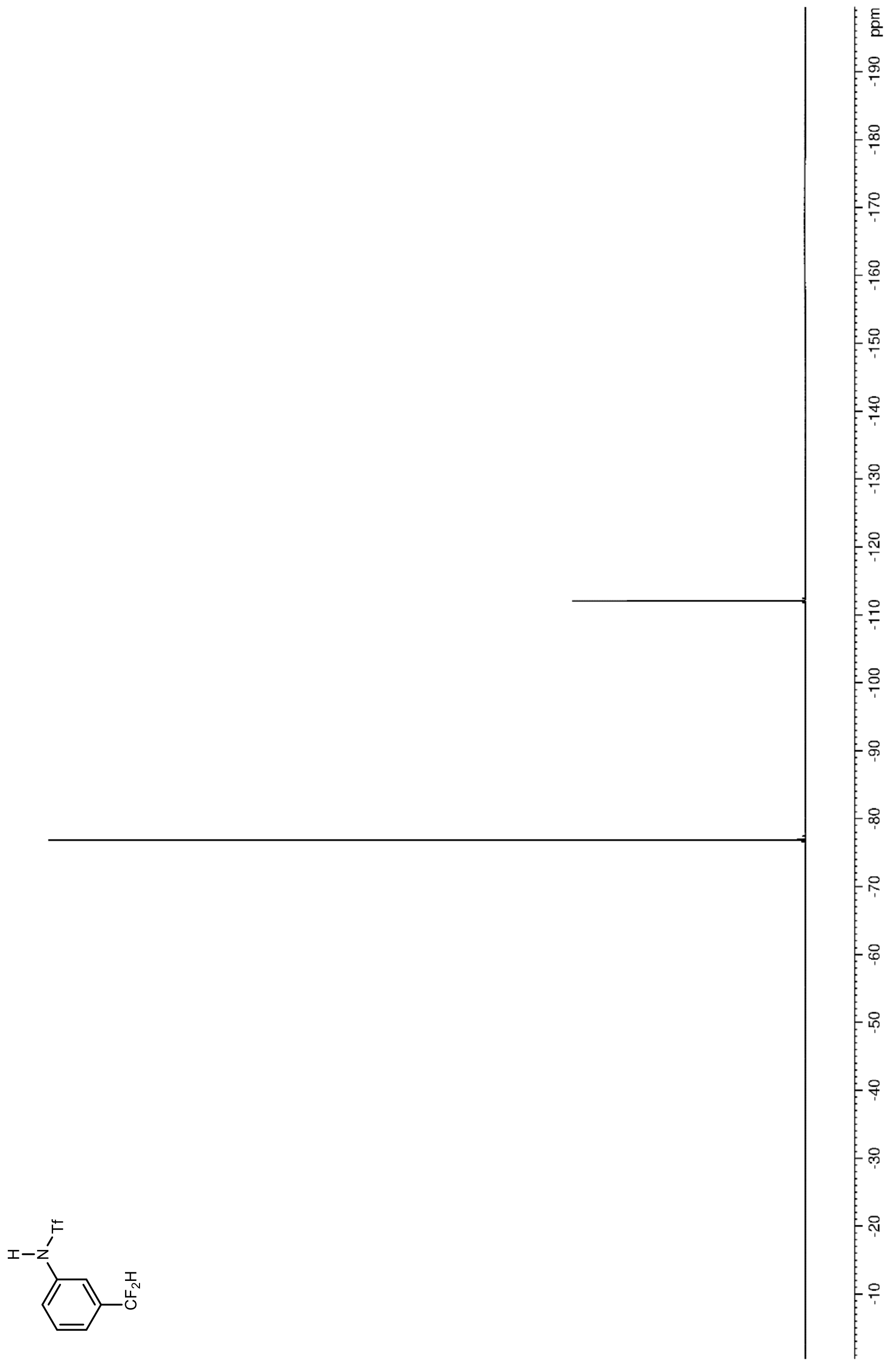

Figure 64. ^{1}H NMR (400 MHz, $CDCl_3$) of **E5**

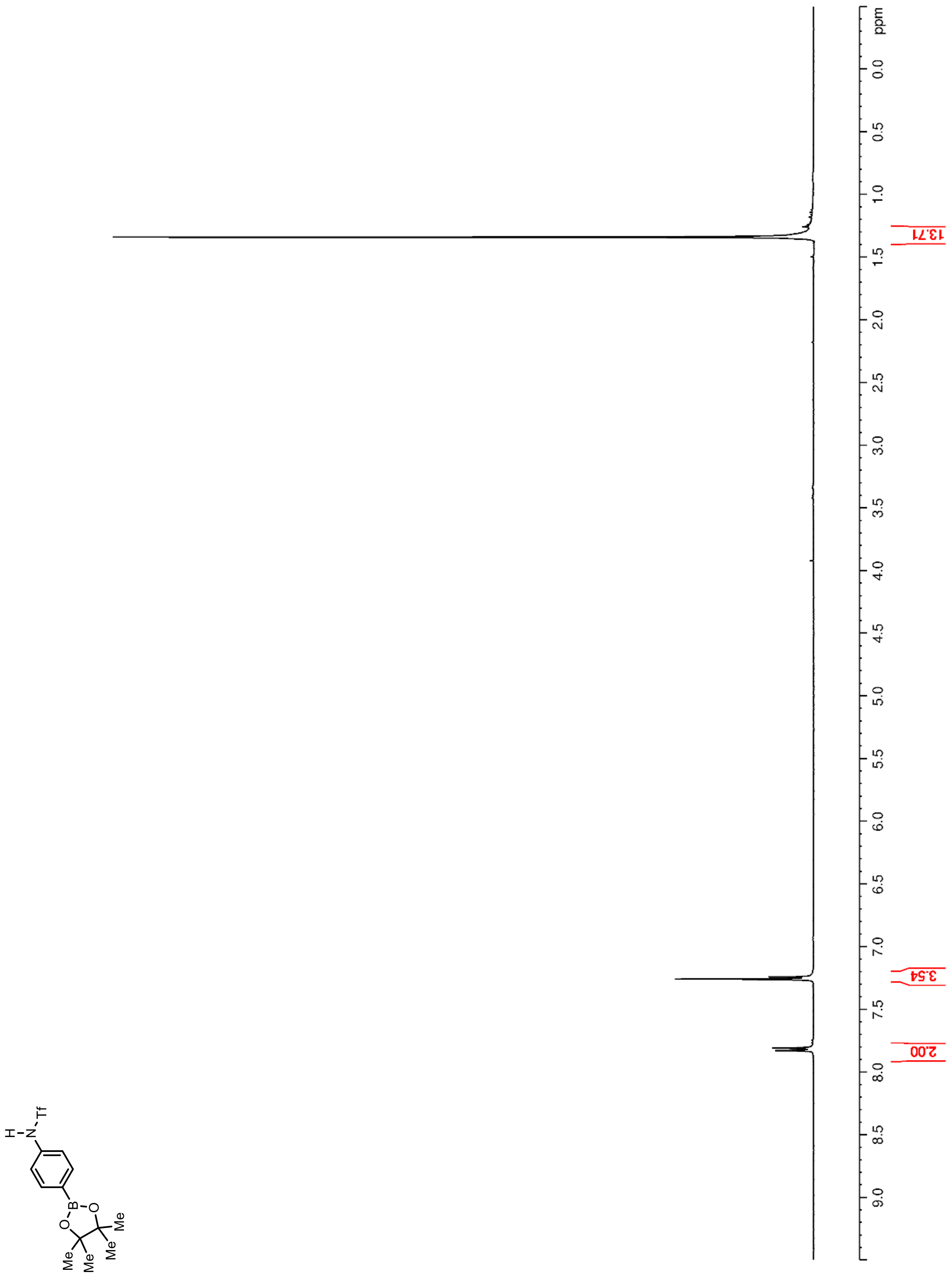

Figure 65. ^{13}C NMR (150 MHz, $CDCl_3$) of **E5**

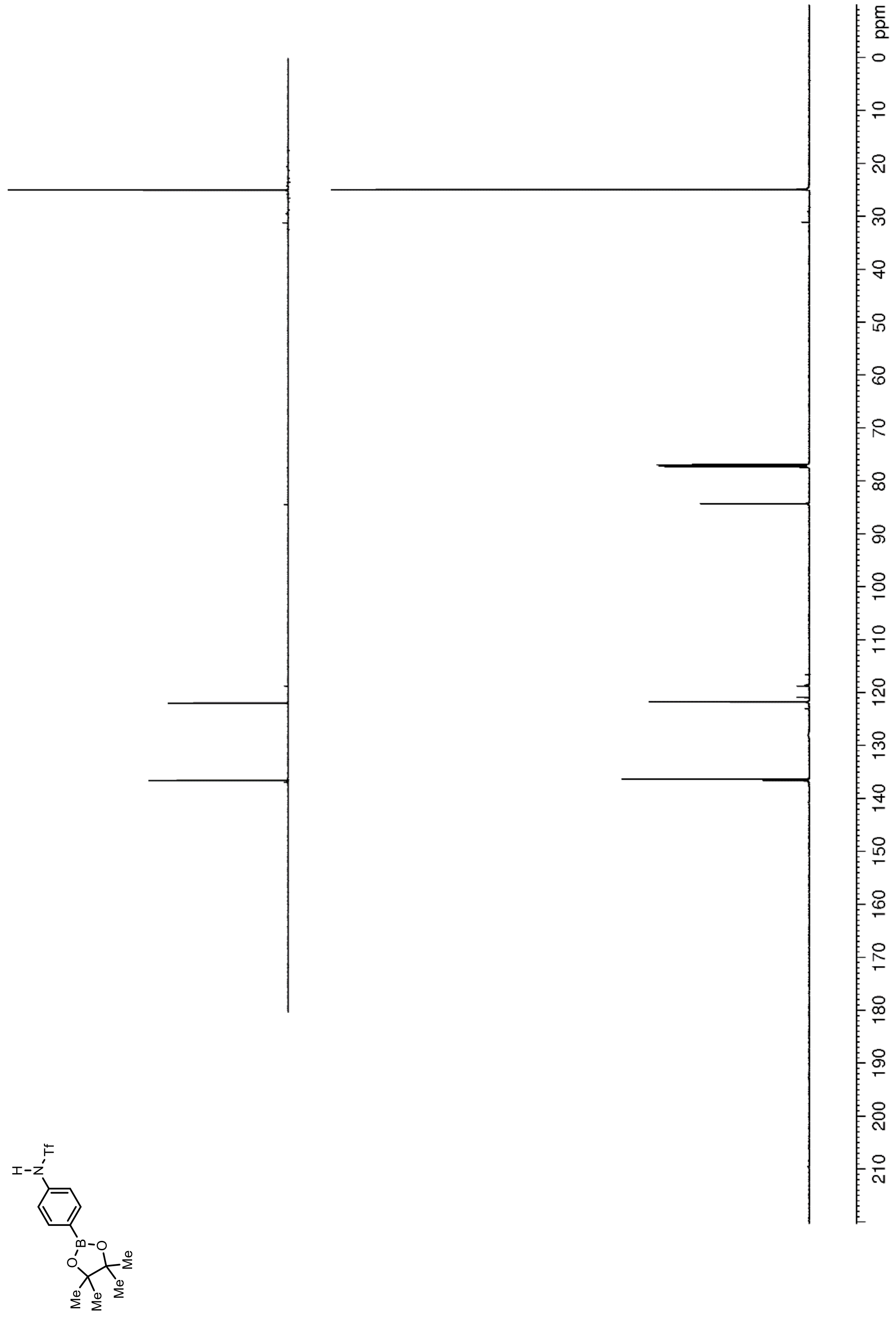

Figure 66. ^{19}F NMR (282 MHz, $CDCl_3$) of **E5**

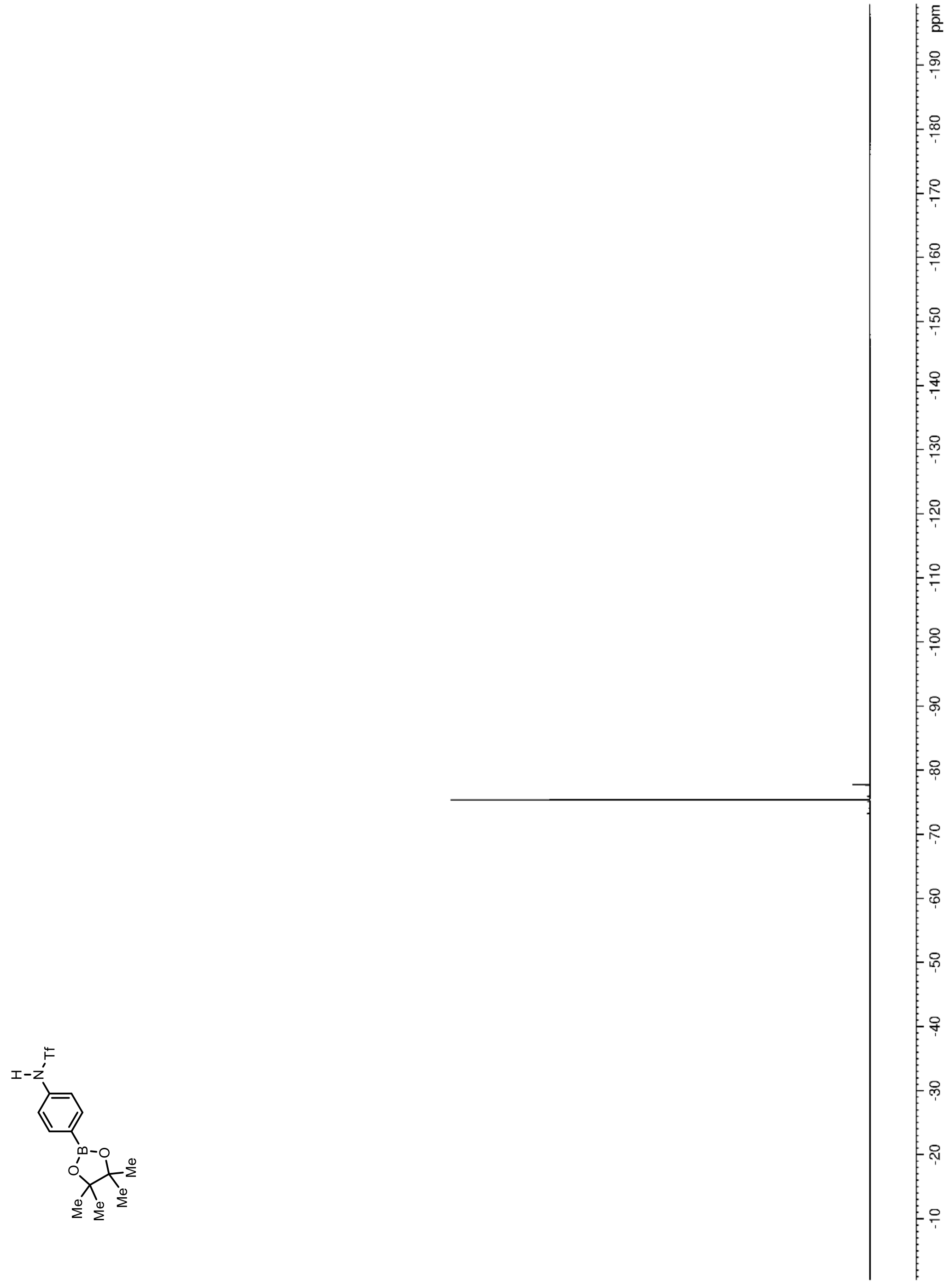

Figure 67. ^{1}H NMR (400 MHz, acetone-d_6) of **E6**

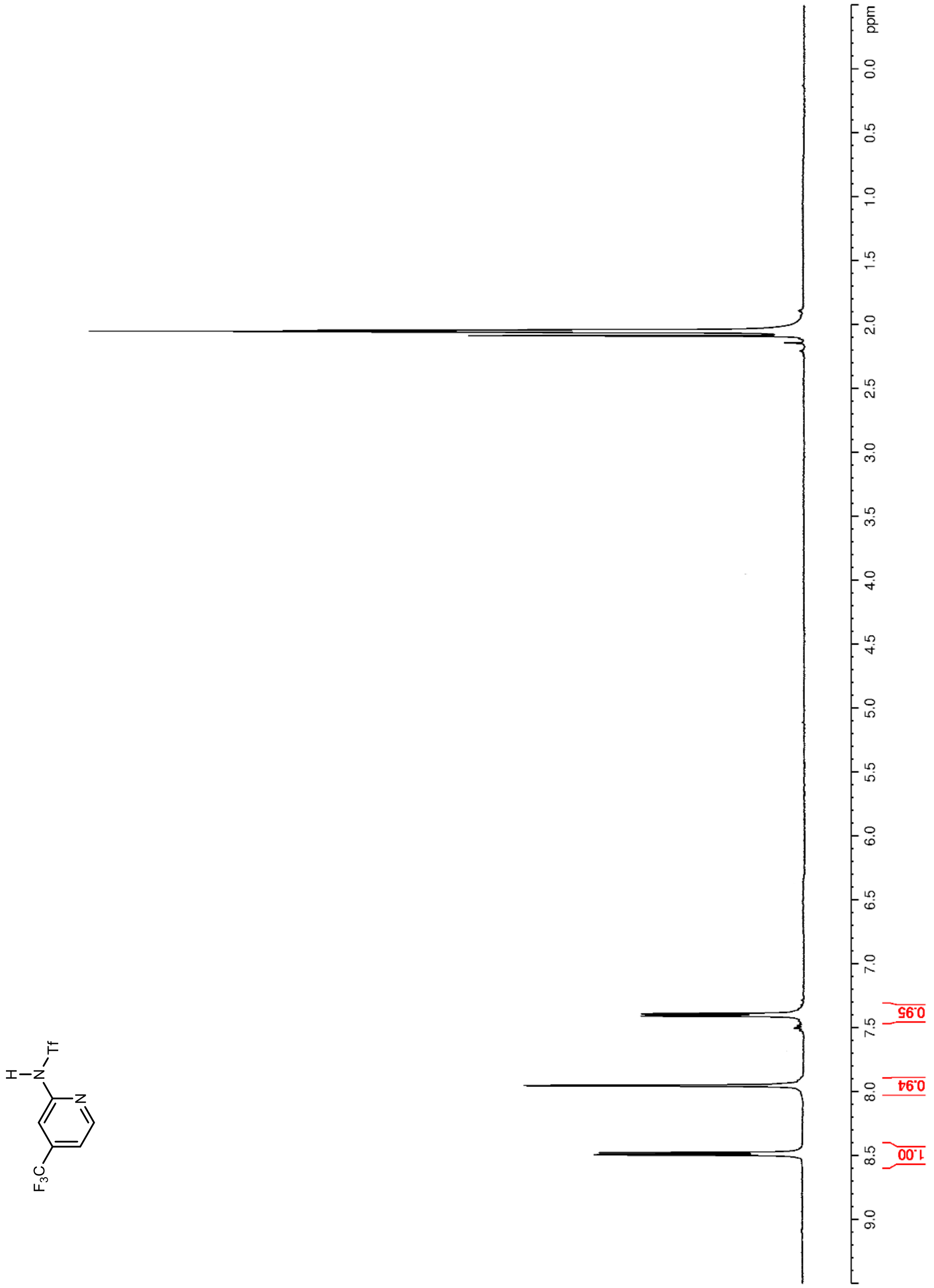

Figure 68. ^{13}C NMR (150 MHz, acetone-d_6) of **E6**

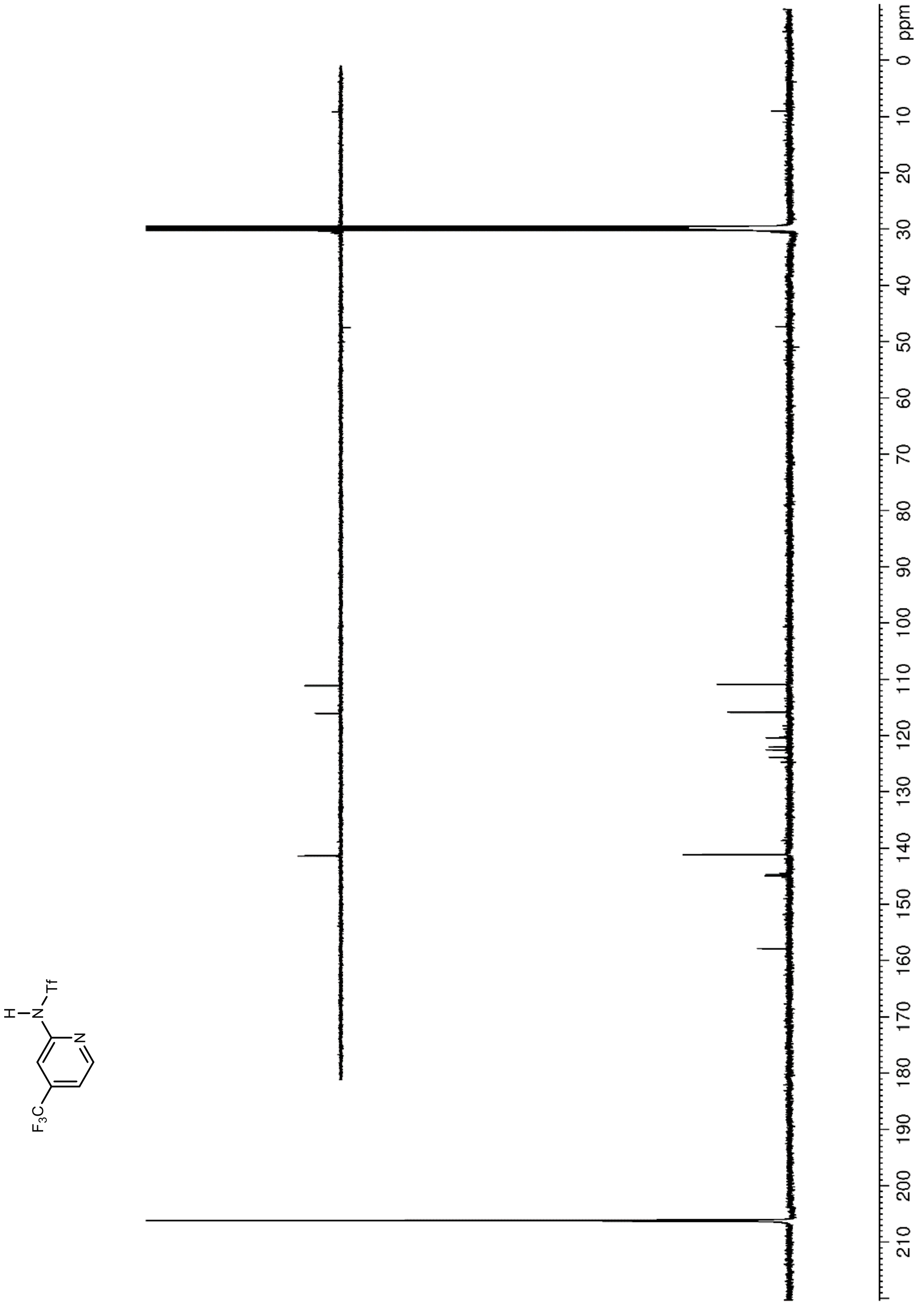

Figure 69. ^{19}F NMR (282 MHz, acetone-d_6) of **E6**

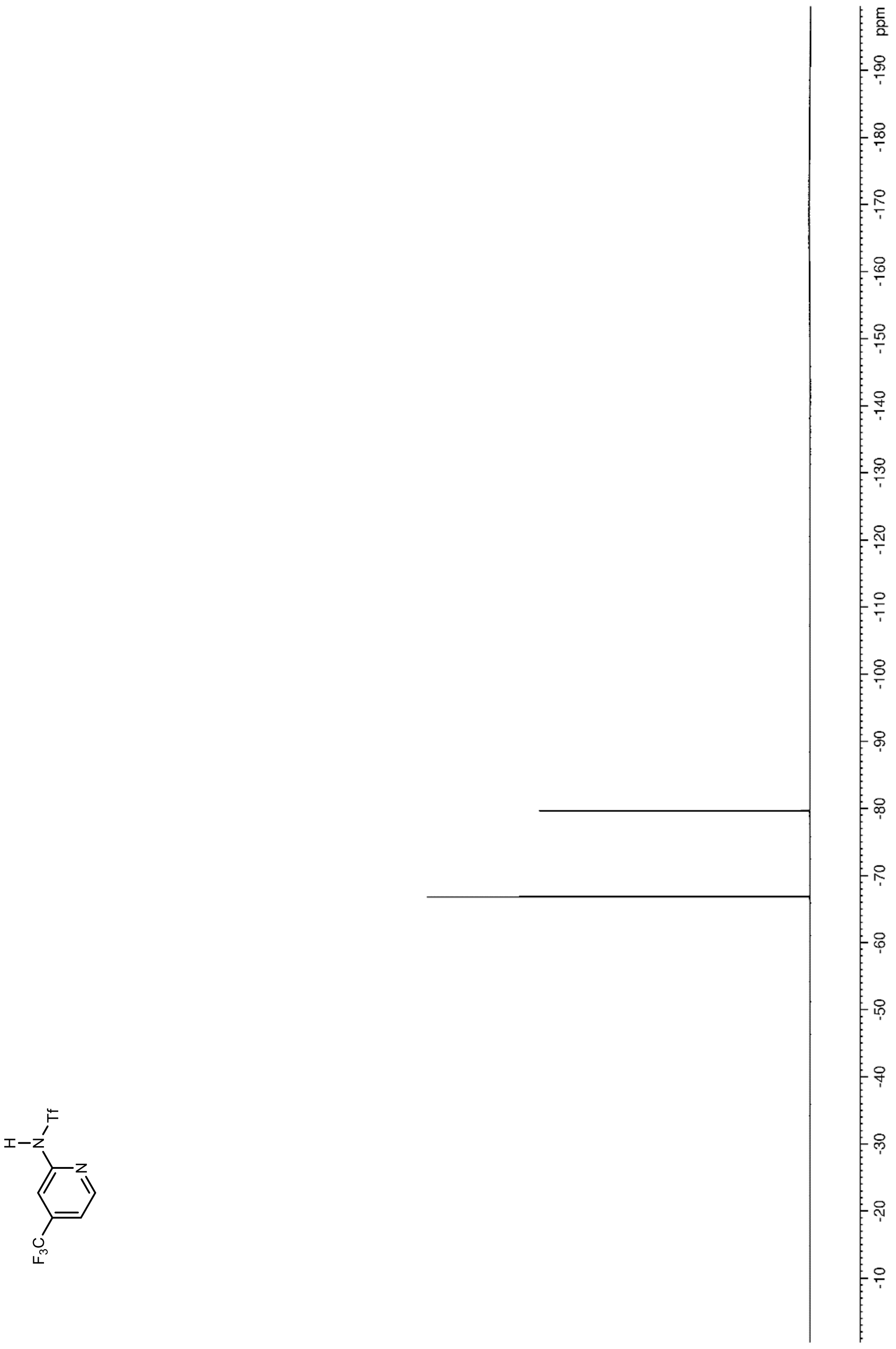

Figure 70. ^{1}H NMR (400 MHz, $CDCl_3$) of **E7**

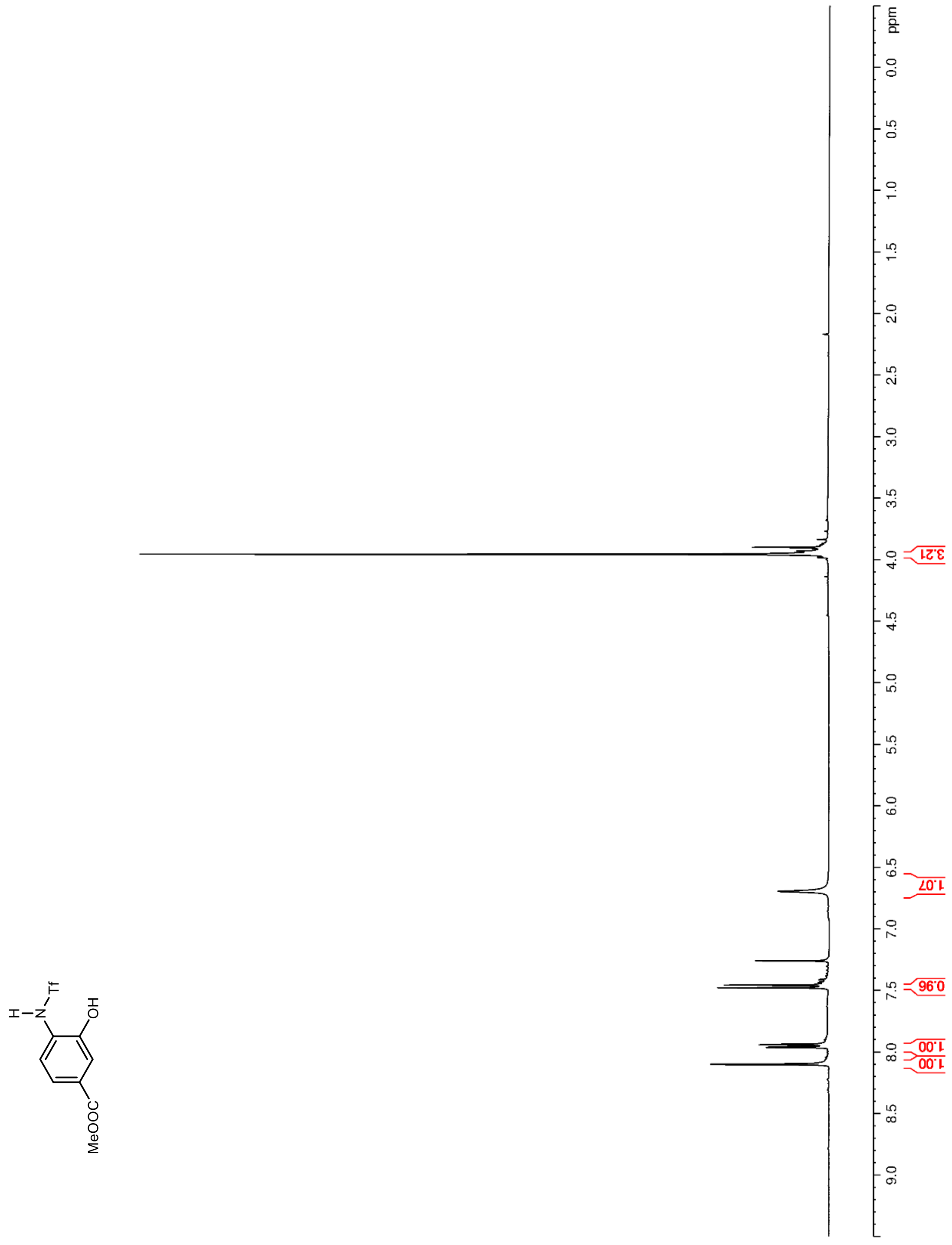

Figure 71. ^{13}C NMR (150 MHz, $CDCl_3$) of **E7**

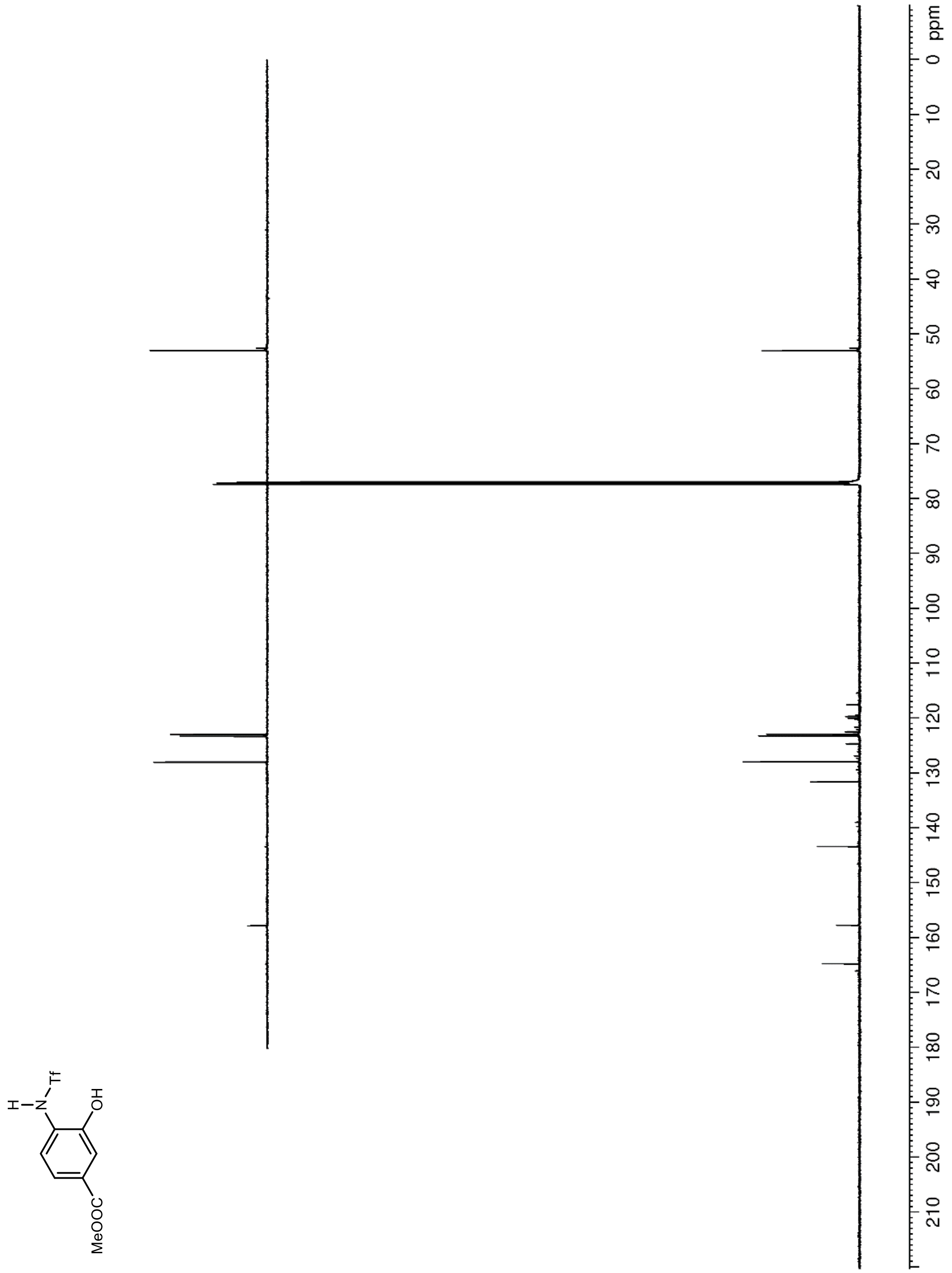

Figure 72. ^{19}F NMR (282 MHz, $CDCl_3$) of **E7**

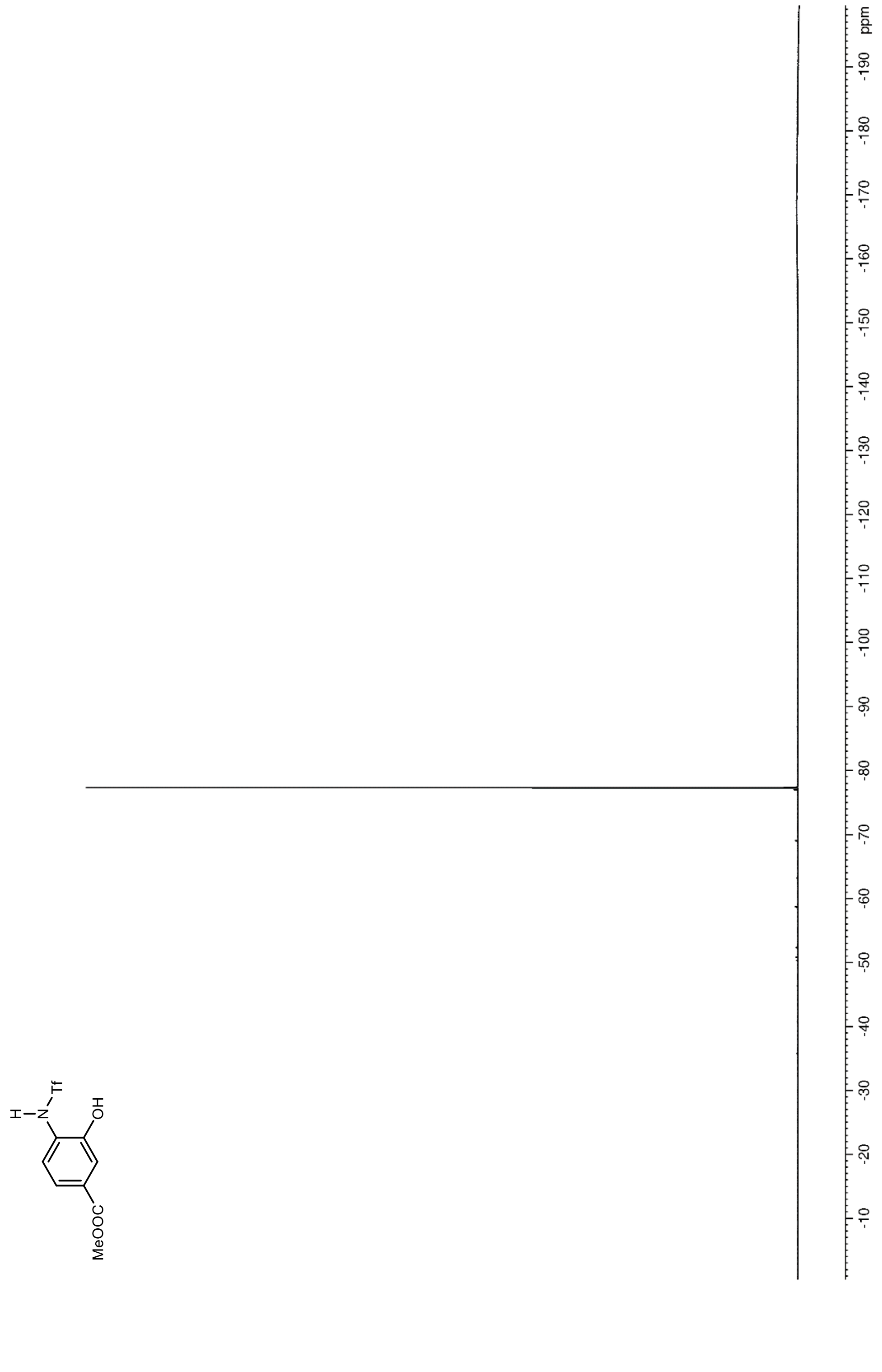

Figure 73. ^{1}H NMR (400 MHz, $CDCl_3$) of **E8**

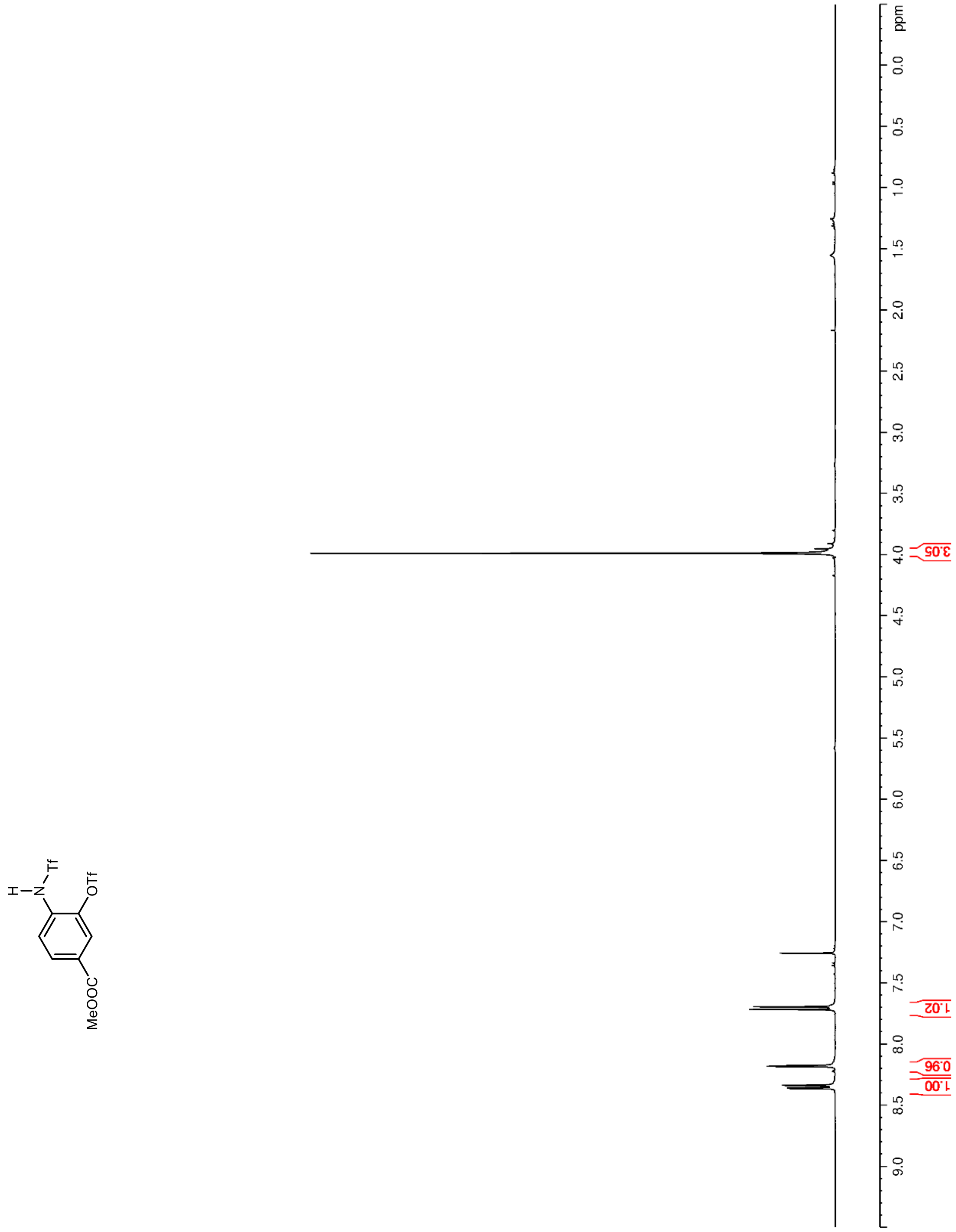

Figure 74. ^{13}C NMR (150 MHz, $CDCl_3$) of **E8**

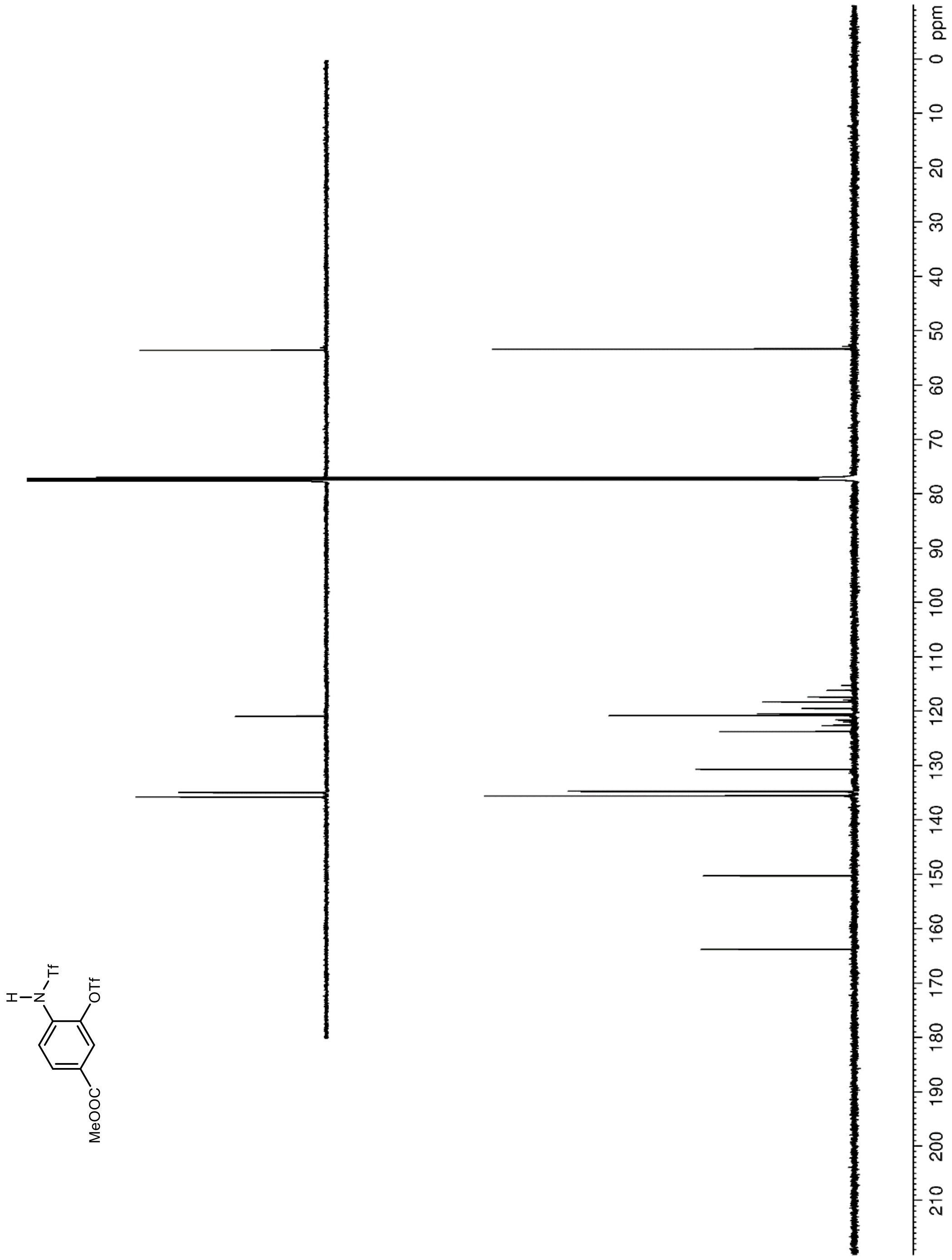

Figure 75. ^{19}F NMR (282 MHz, $CDCl_3$) of **E8**

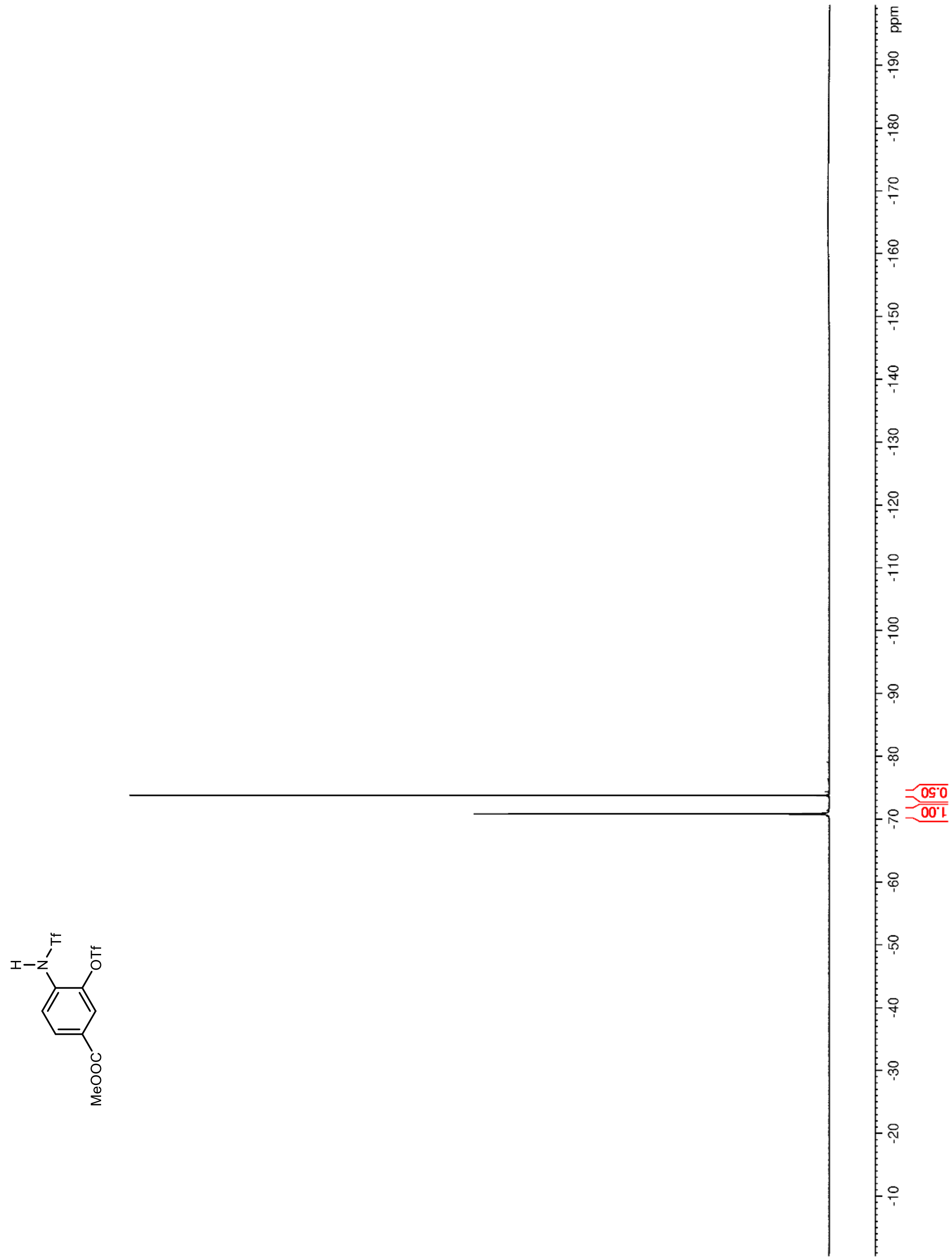

Figure 76. ^{1}H NMR (400 MHz, $CDCl_3$) of **F2**

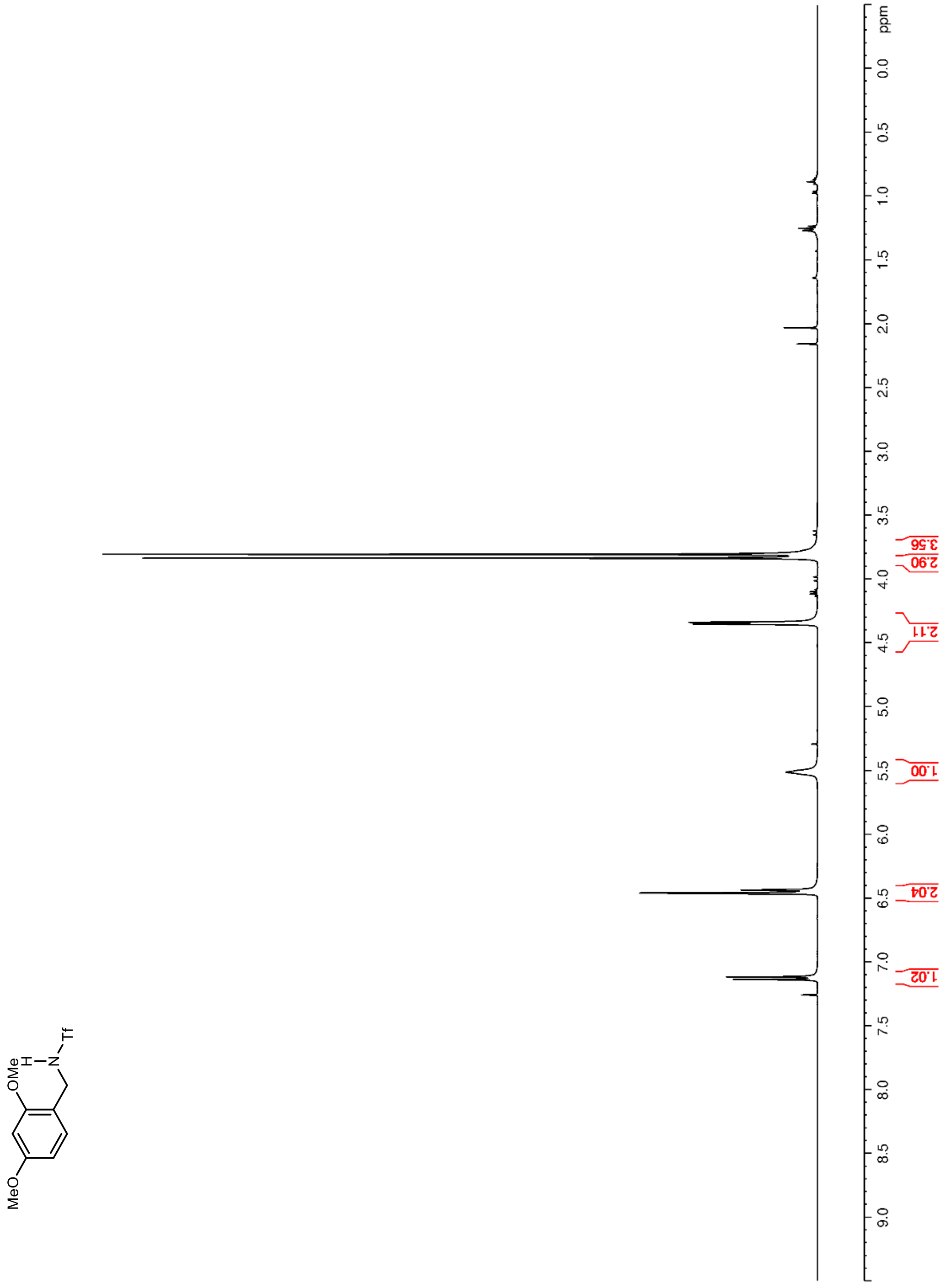

Figure 77. ^{13}C NMR (150 MHz, $CDCl_3$) of **F2**

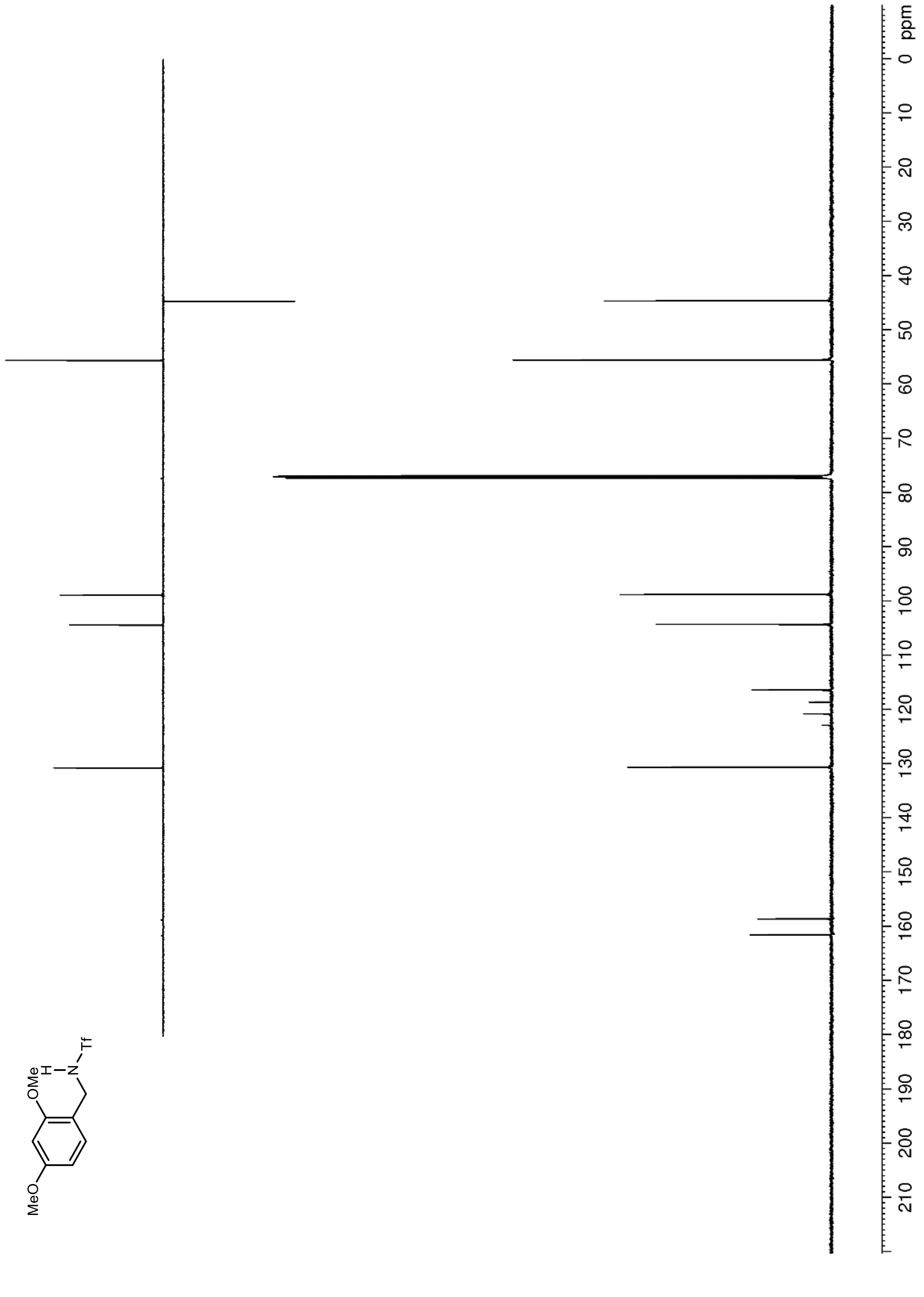

Figure 78. ^{19}F NMR (282 MHz, $CDCl_3$) of **F2**

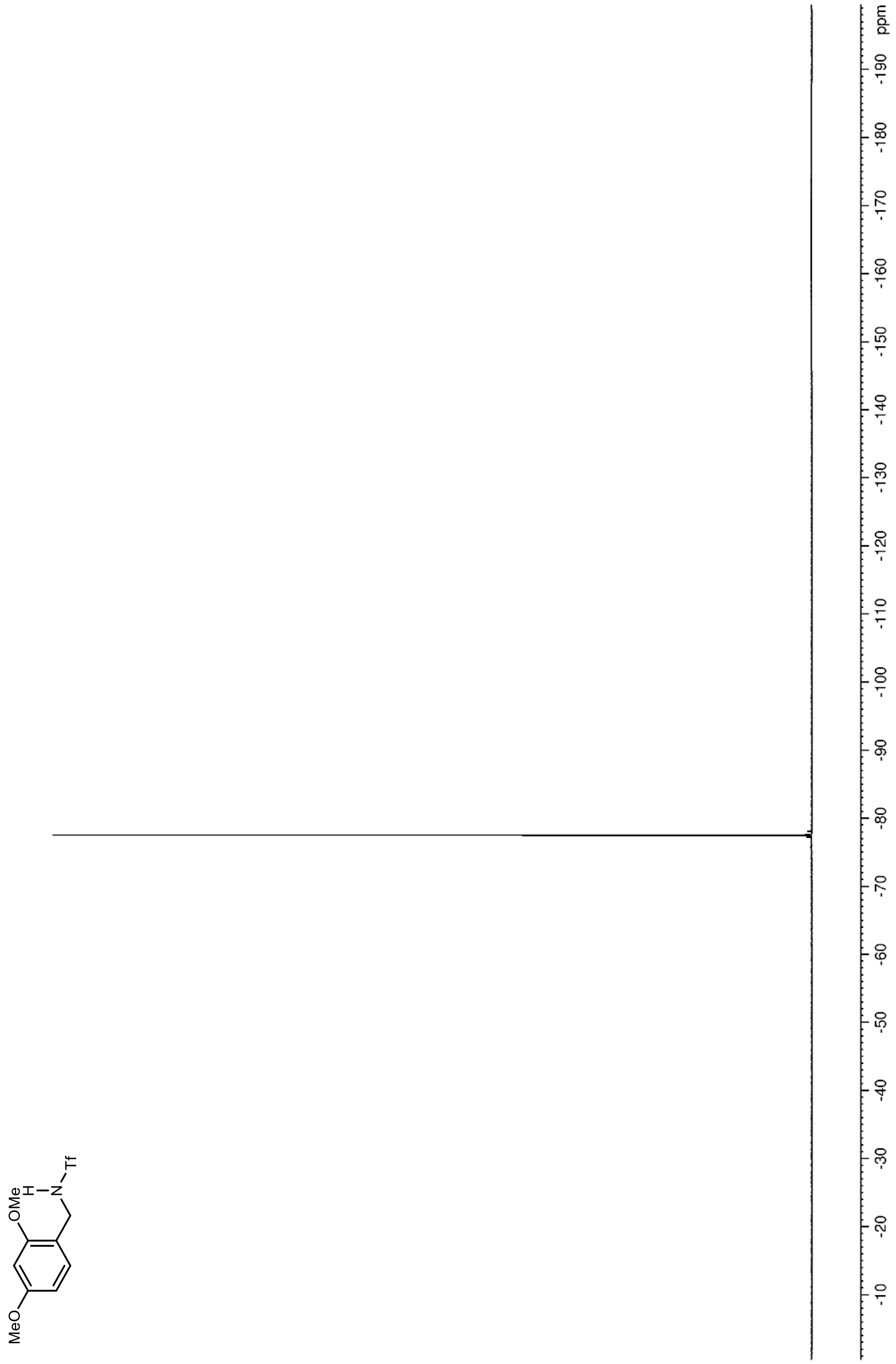

Figure 79. ^{1}H NMR (400 MHz, acetone-d_6) of **F6**

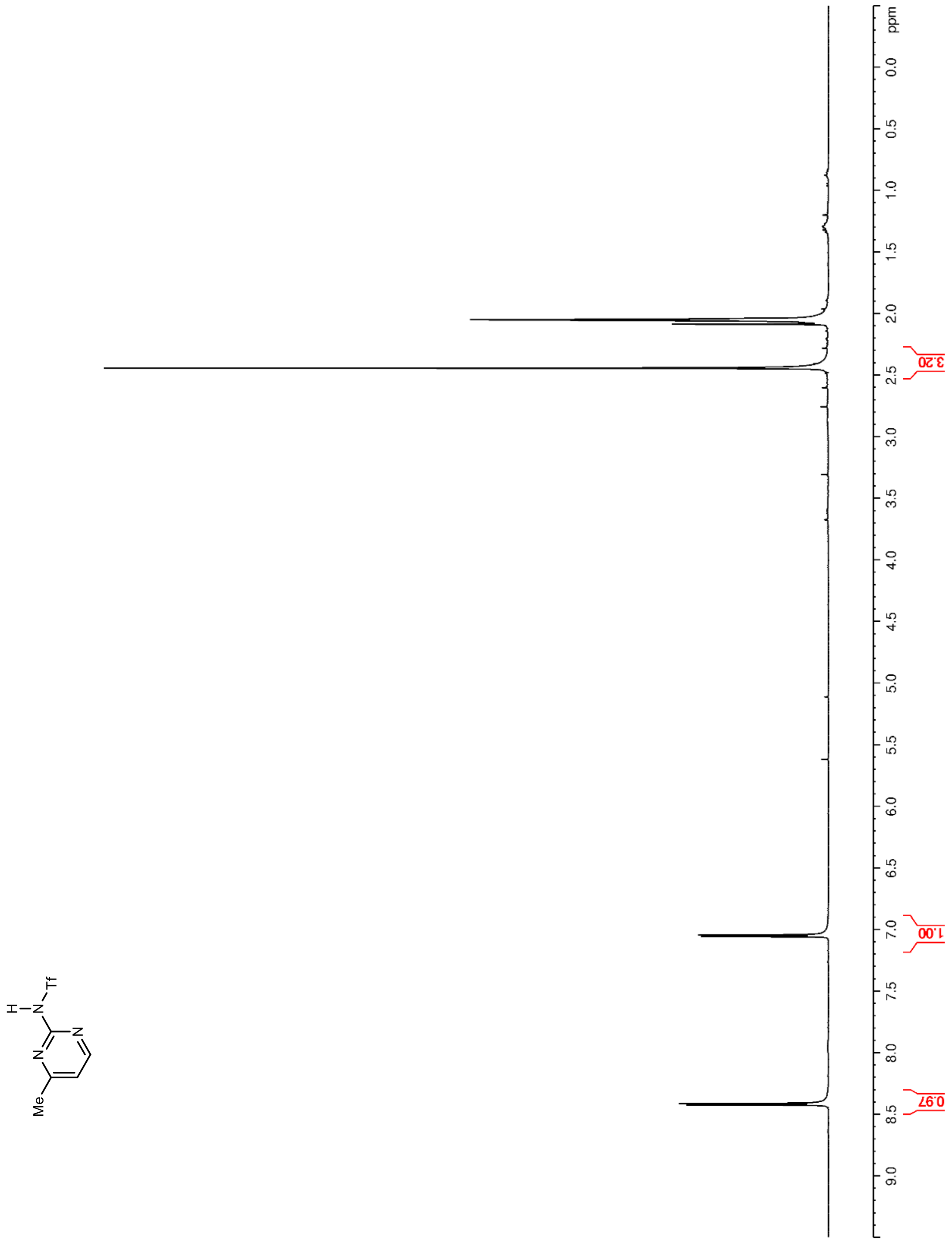

Figure 80. ^{13}C NMR (150 MHz, acetone-d_6) of **F6**

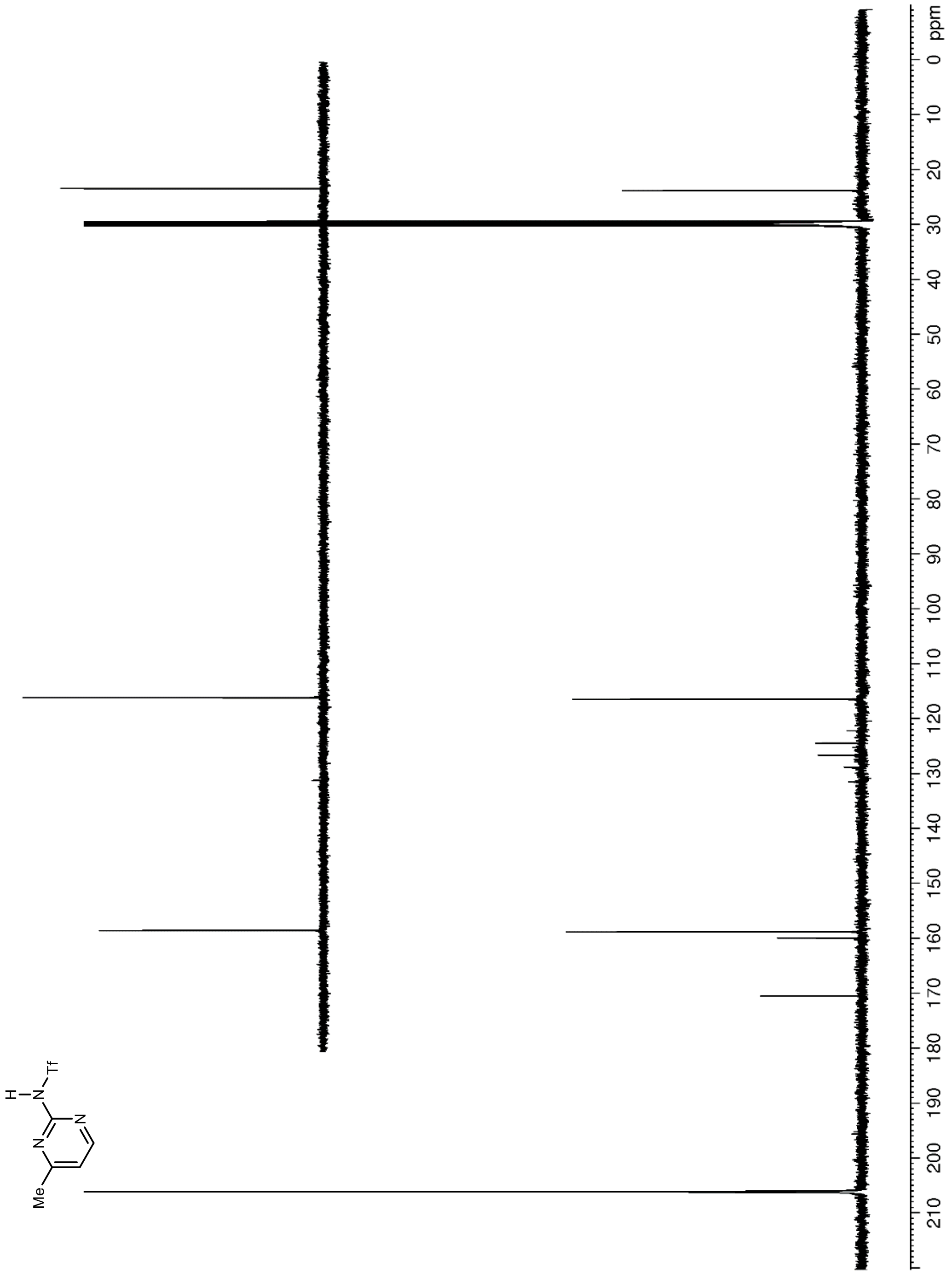

Figure 81. ^{19}F NMR (282 MHz, acetone-d_6) of **F6**

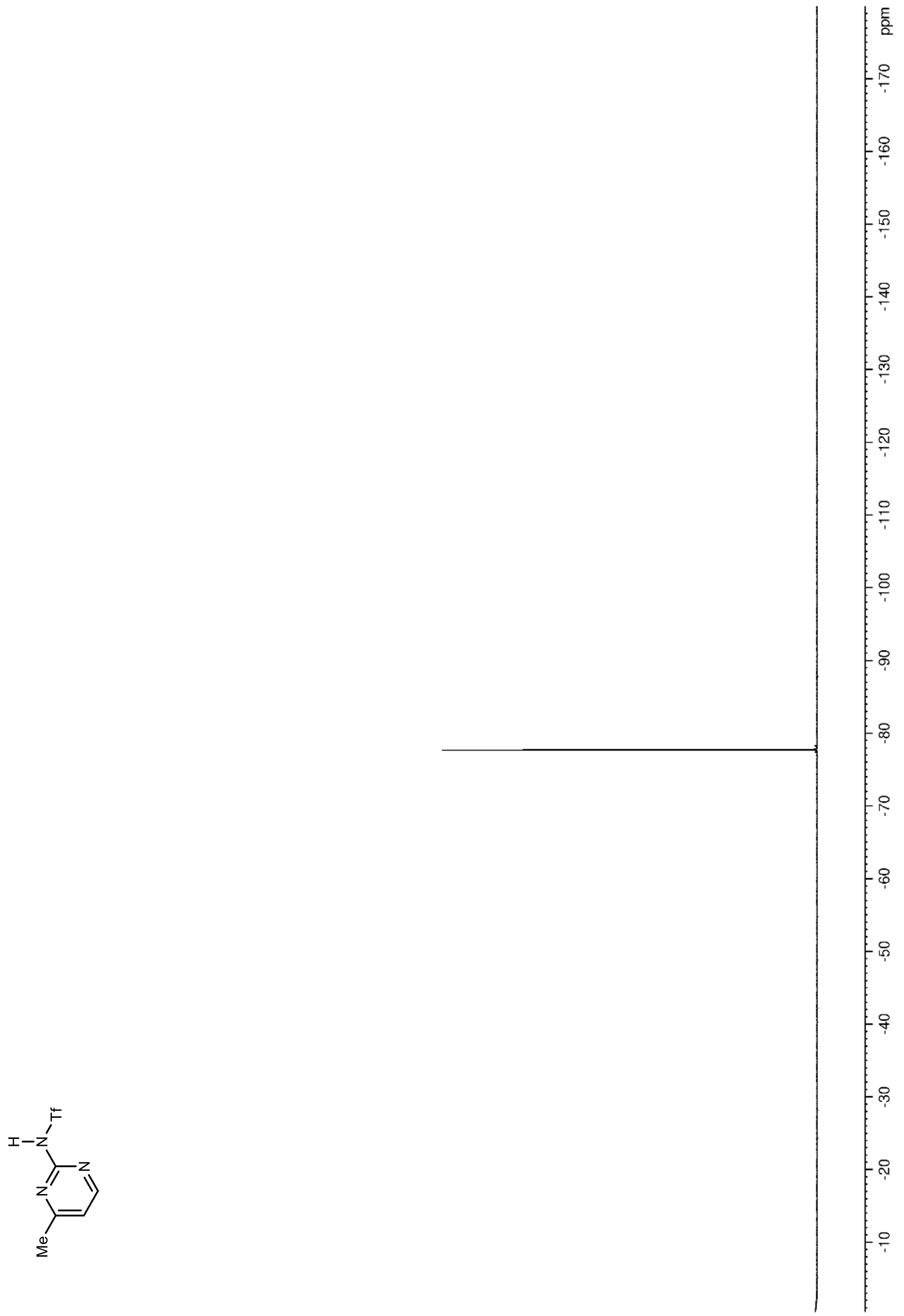

Figure 82. ^{1}H NMR (400 MHz, $CDCl_3$) of **F7**

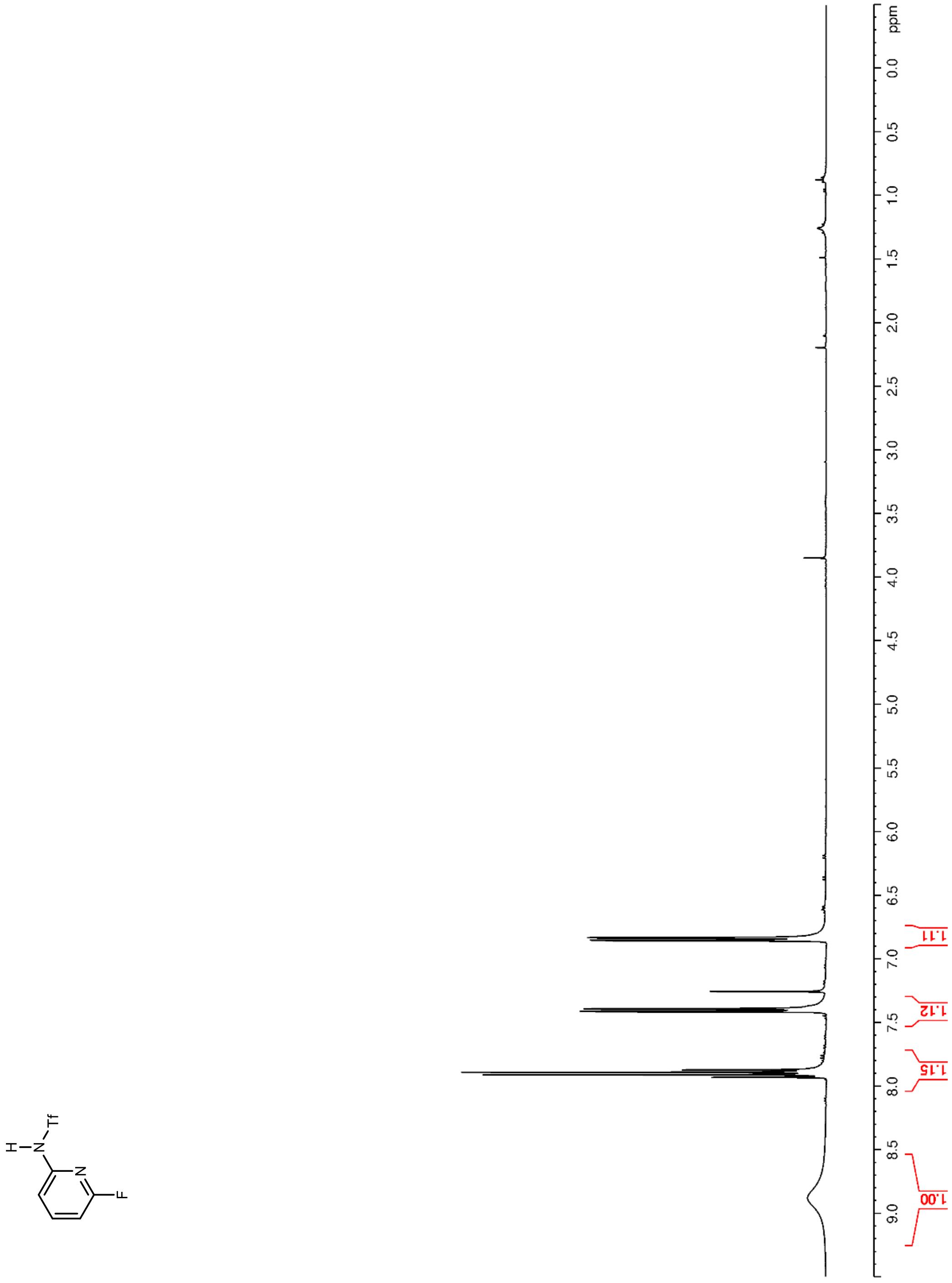

Figure 83. ^{13}C NMR (150 MHz, acetone-d_6) of **F7**

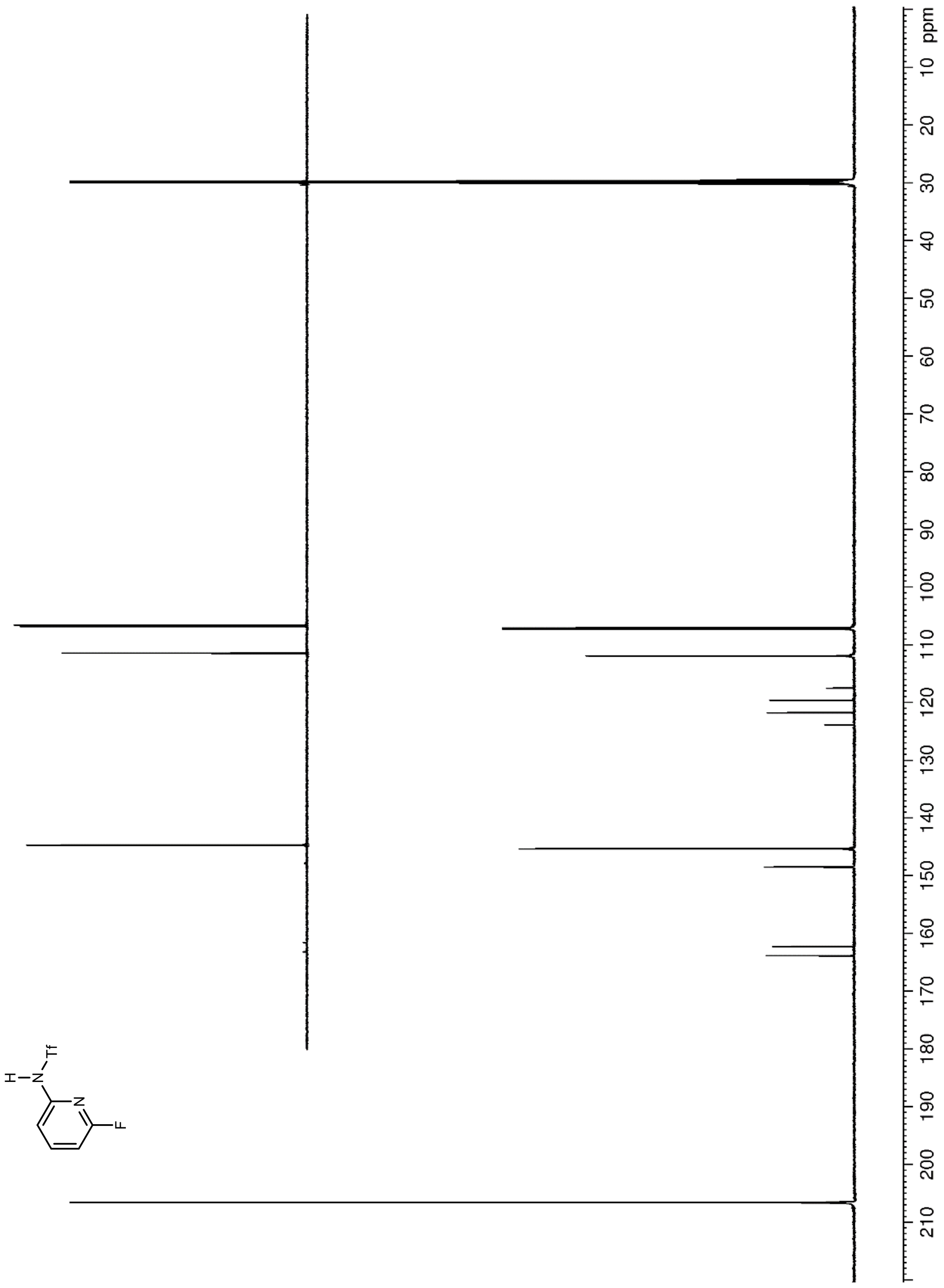

Figure 84. ^{19}F NMR (282 MHz, $CDCl_3$) of **F7**

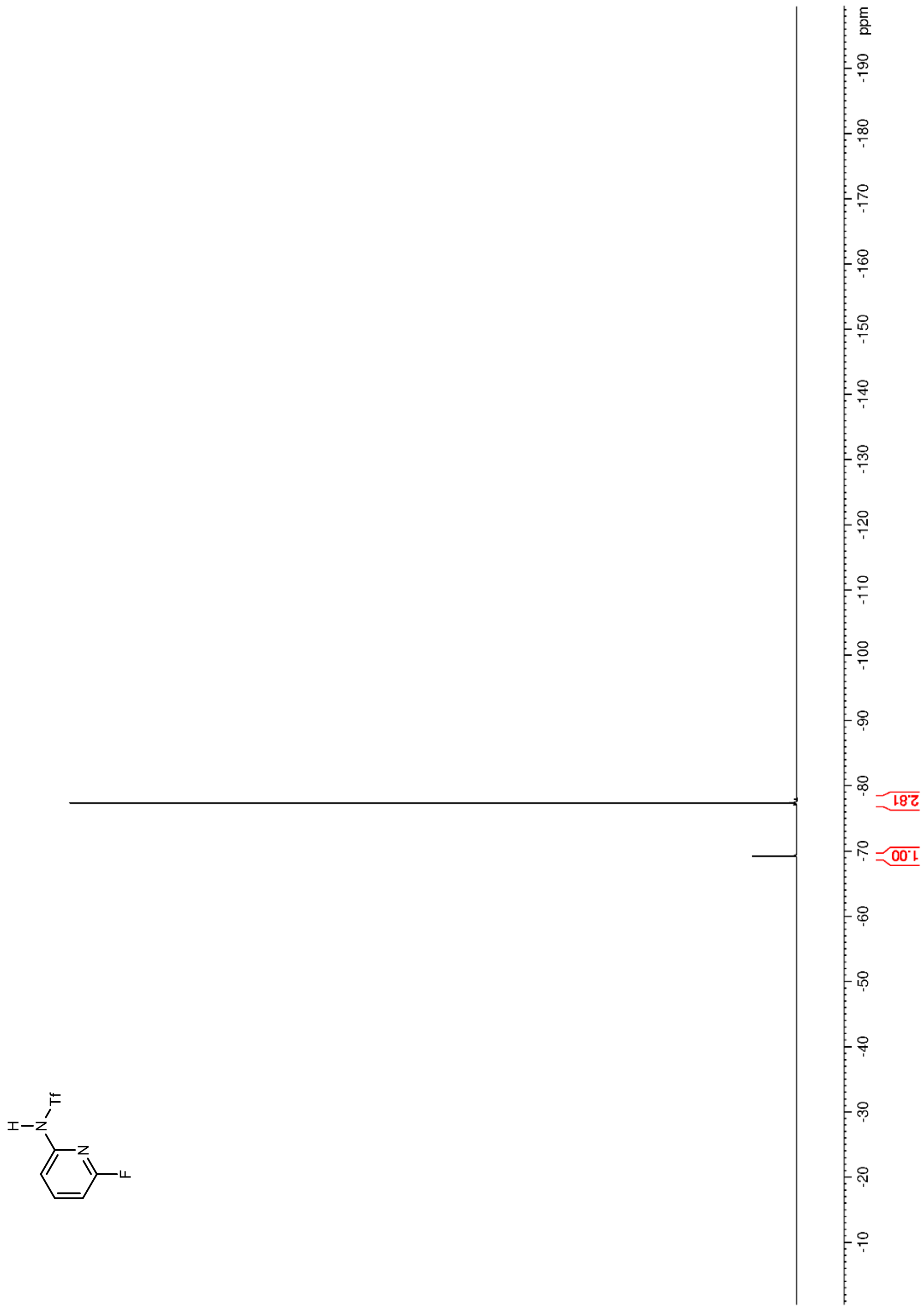

Figure 85. ^{1}H NMR (400 MHz, $CDCl_3$) of **F8**

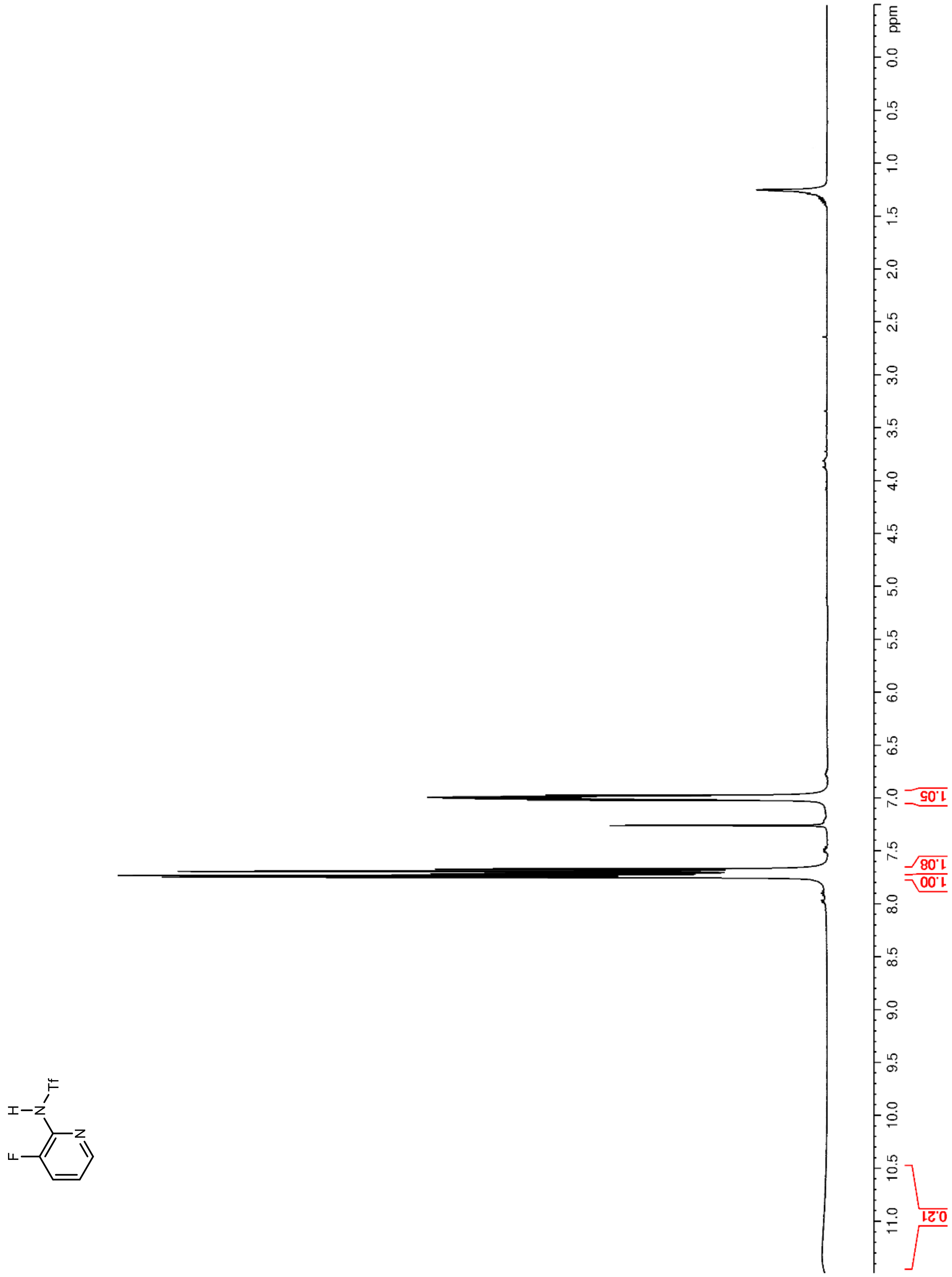

Figure 86. ^{13}C NMR (150 MHz, acetone-d_6) of **F8**

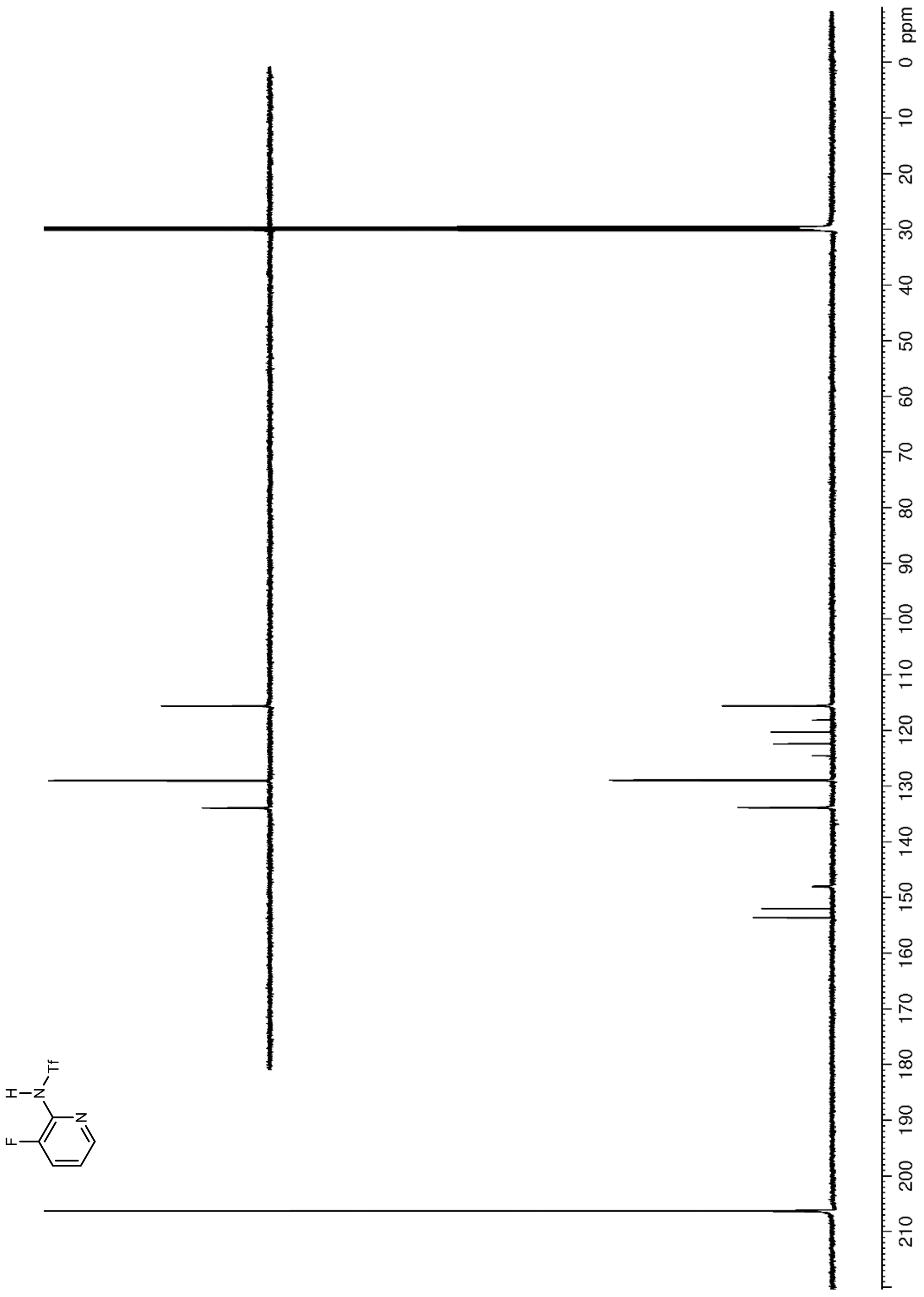

Figure 87. ^{19}F NMR (282 MHz, $CDCl_3$) of **F8**

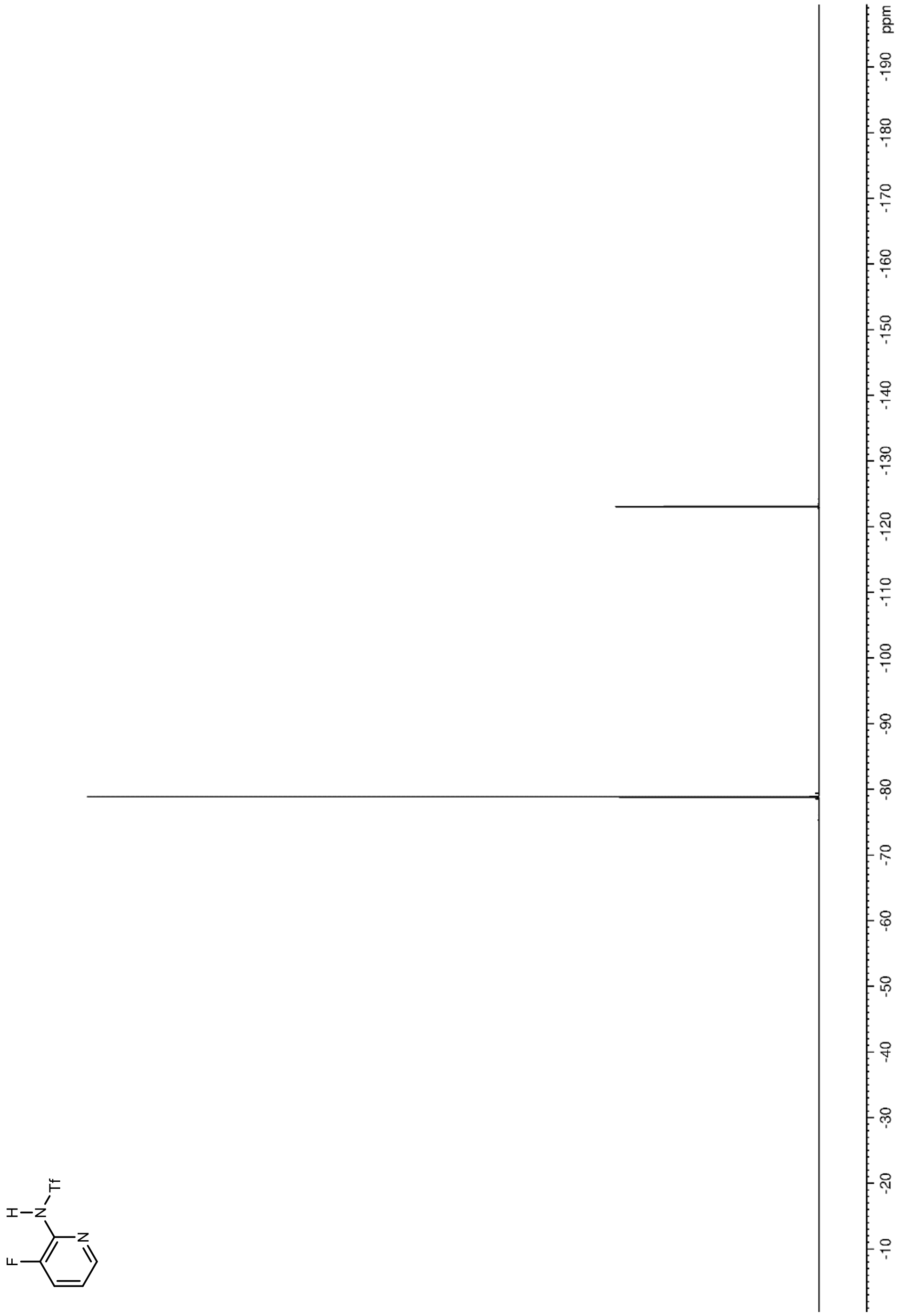

Figure 88. ^{1}H NMR (400 MHz, acetone-d_6) of **G1**

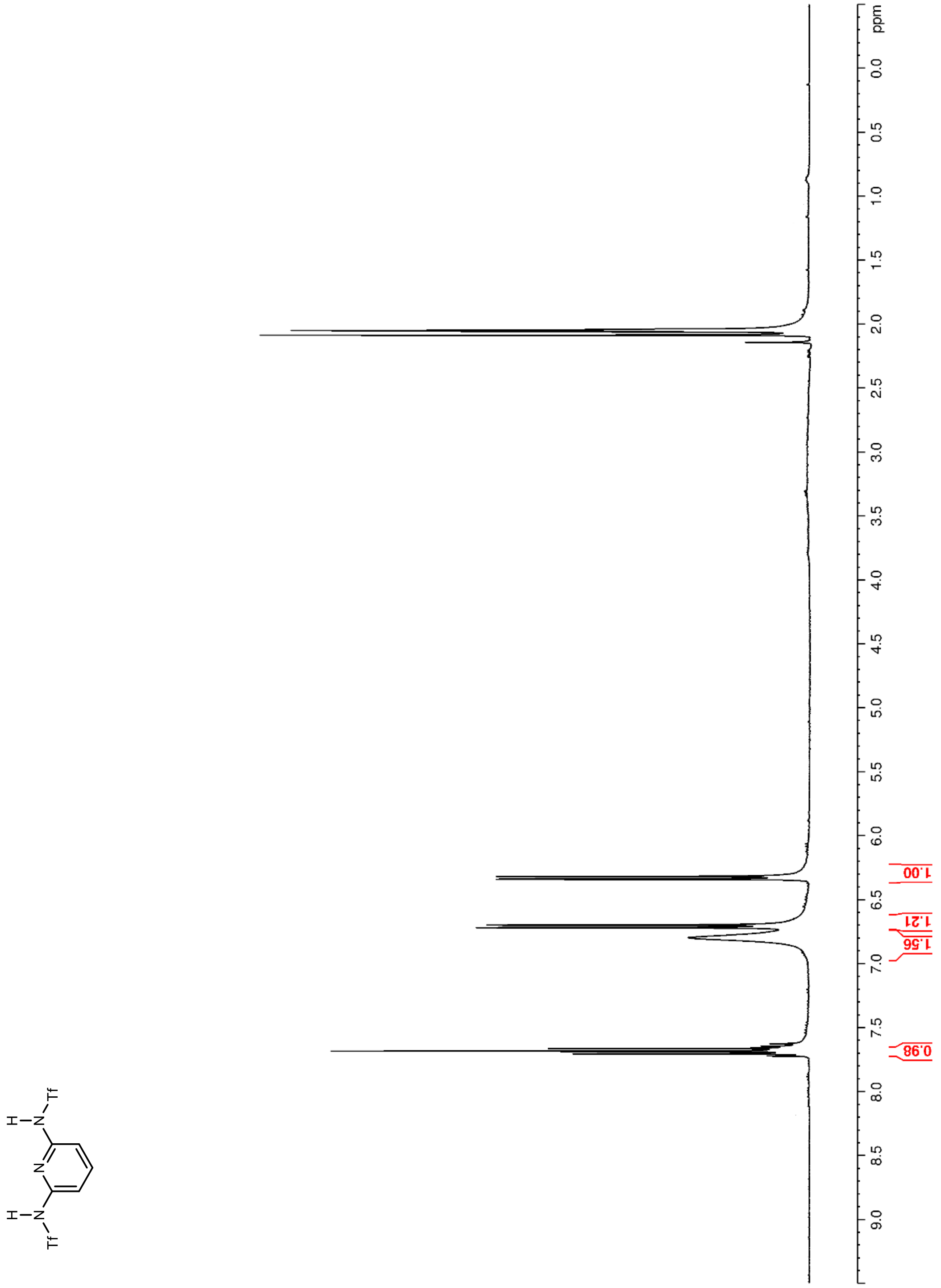

Figure 89. ^{13}C NMR (150 MHz, acetone-d_6) of **G1**

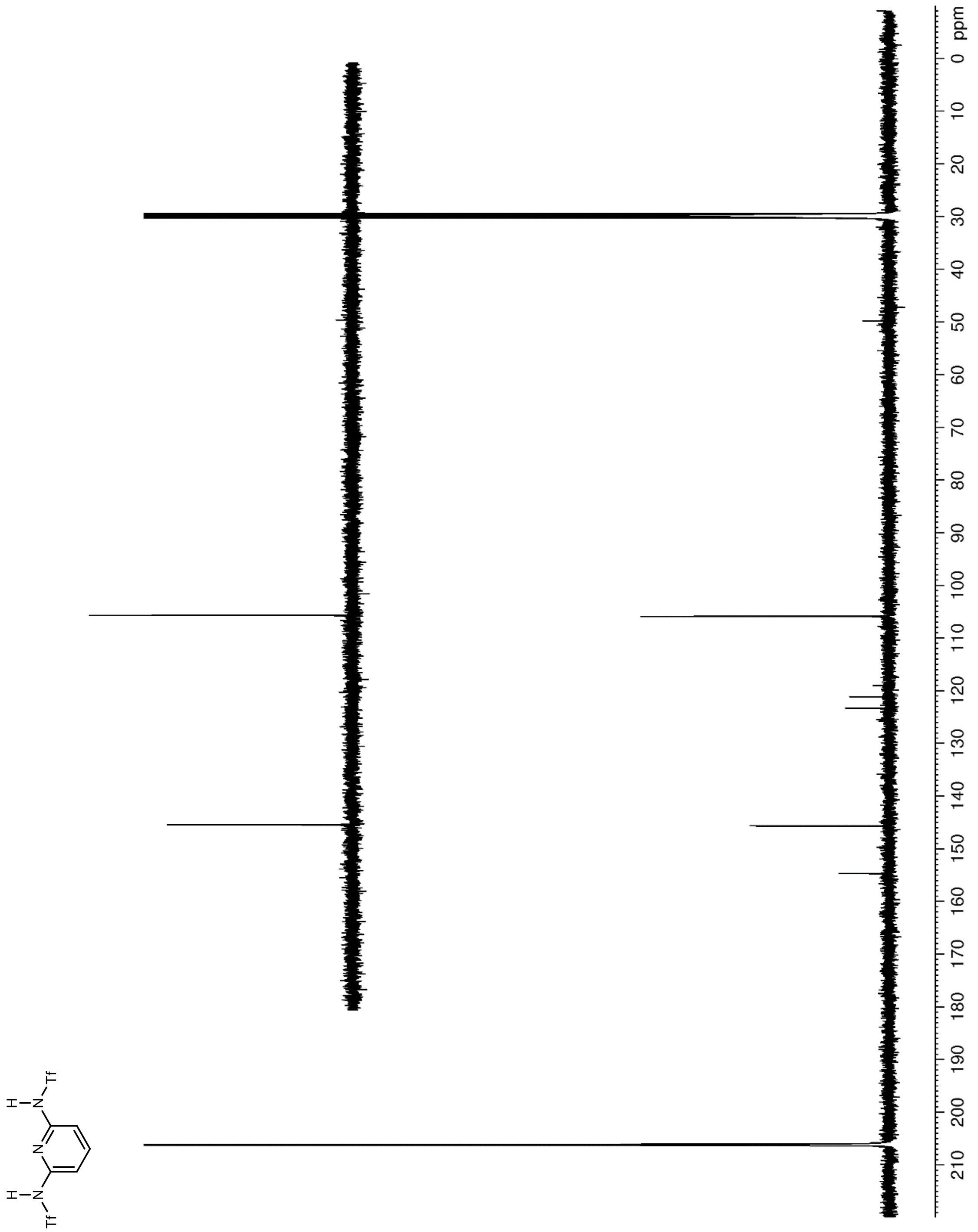

Figure 90. ^{19}F NMR (282 MHz, acetone-d_6) of **G1**

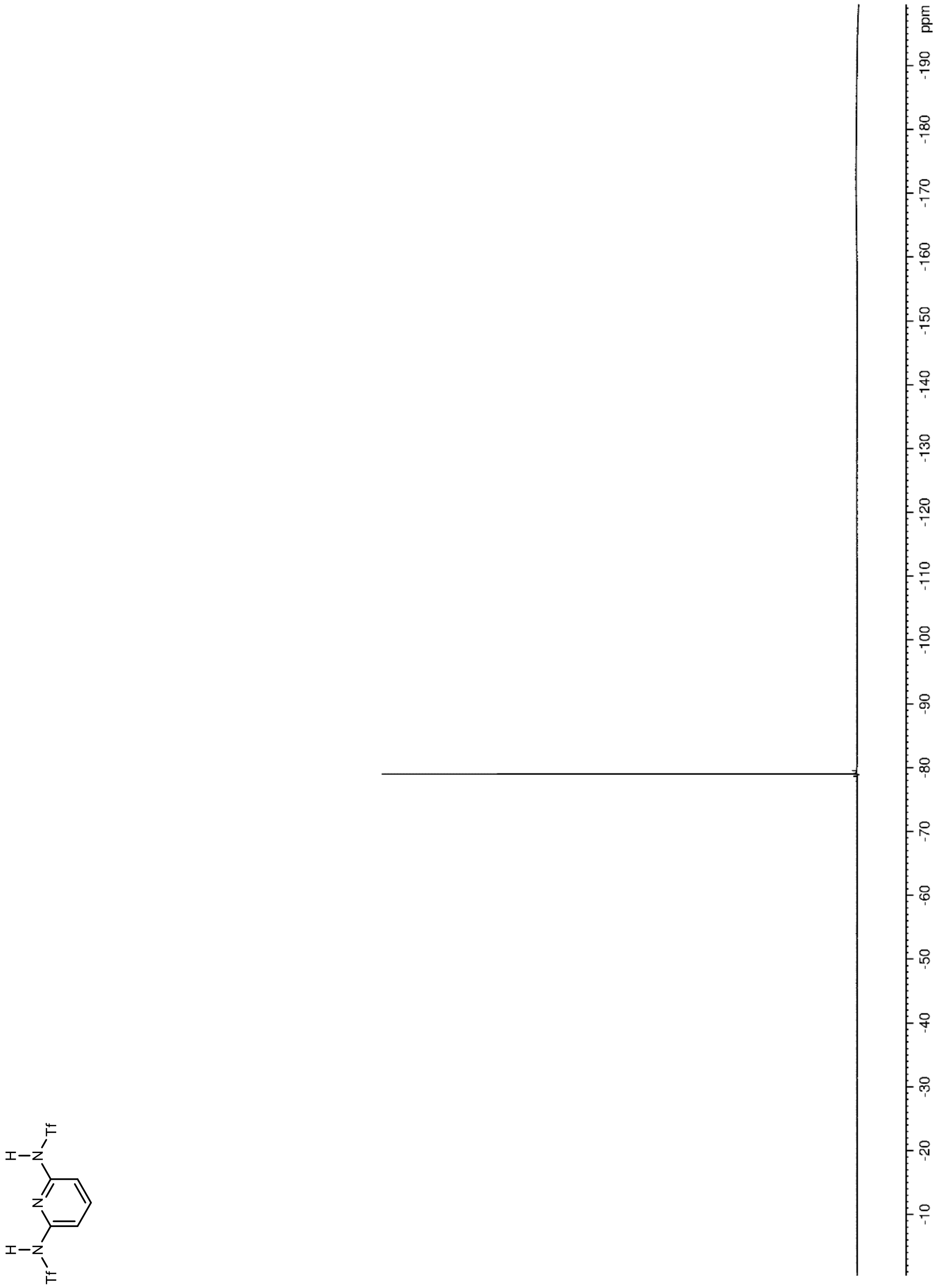

Figure 91. ^{1}H NMR (400 MHz, acetone-d_6) of **G2**

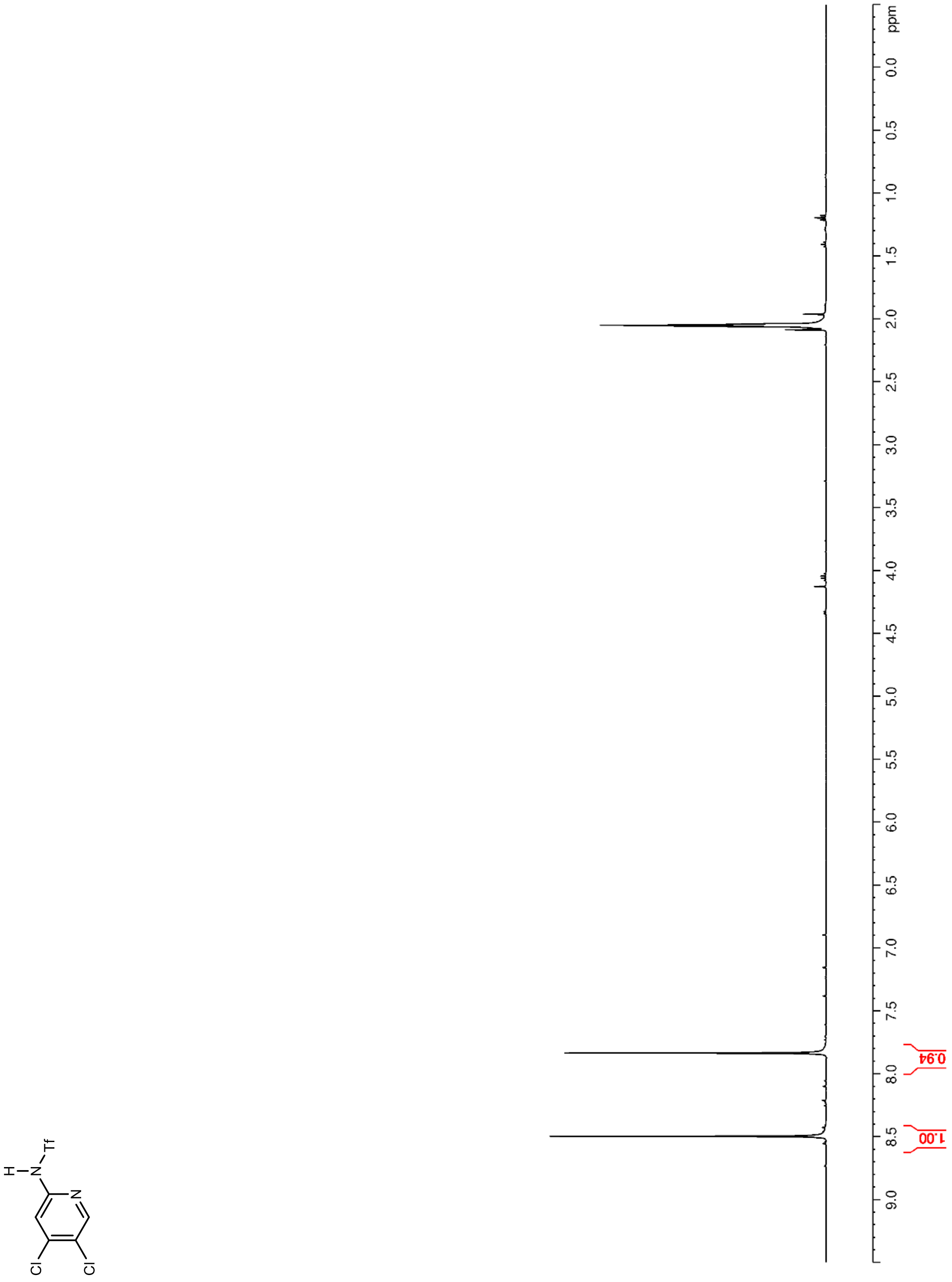

Figure 92. ^{13}C NMR (150 MHz, acetone-d_6) of **G2**

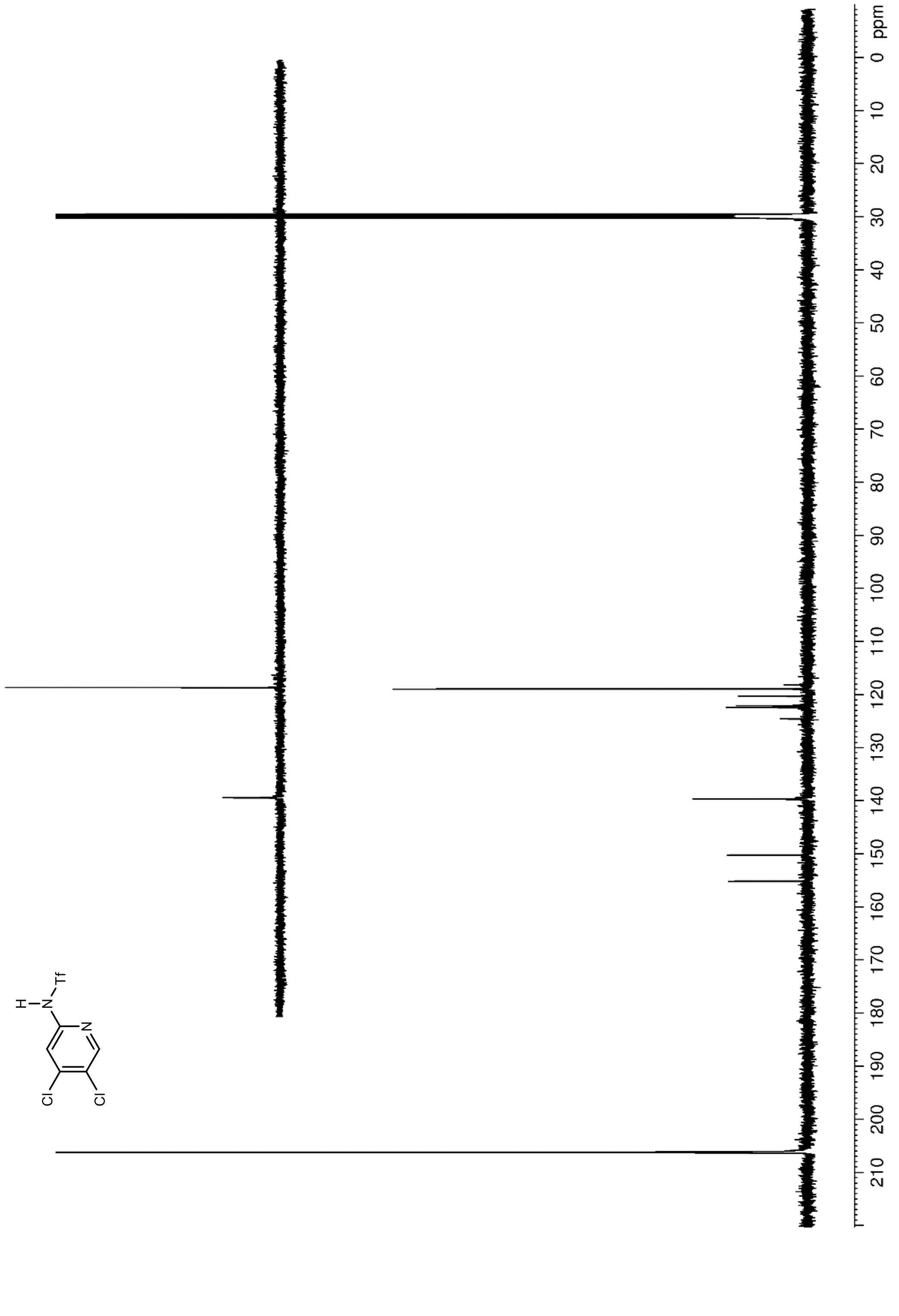

Figure 93. ^{19}F NMR (282 MHz, acetone-d_6) of **G2**

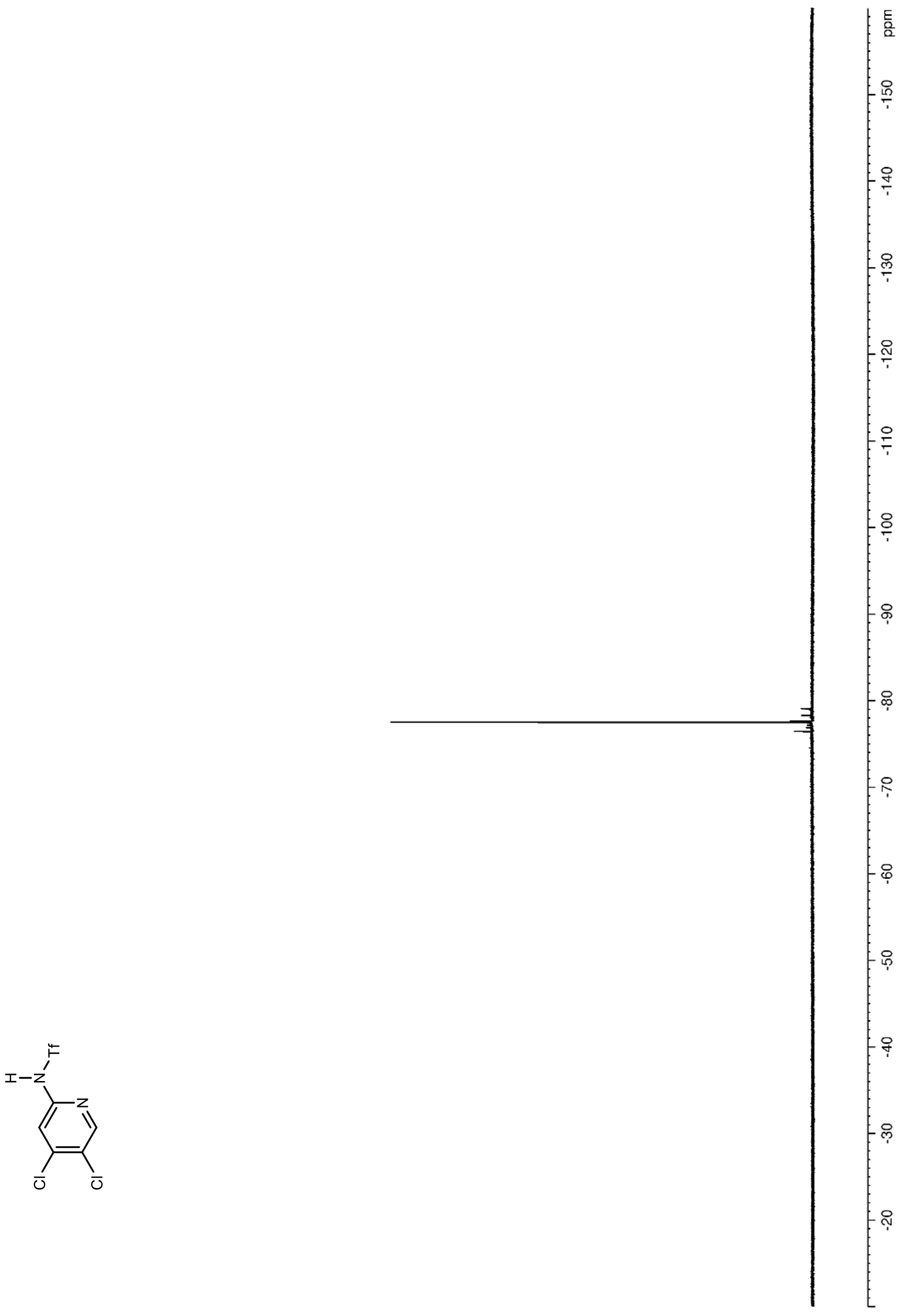

Figure 94. ^{1}H NMR (400 MHz, $CDCl_3$) of **G3**

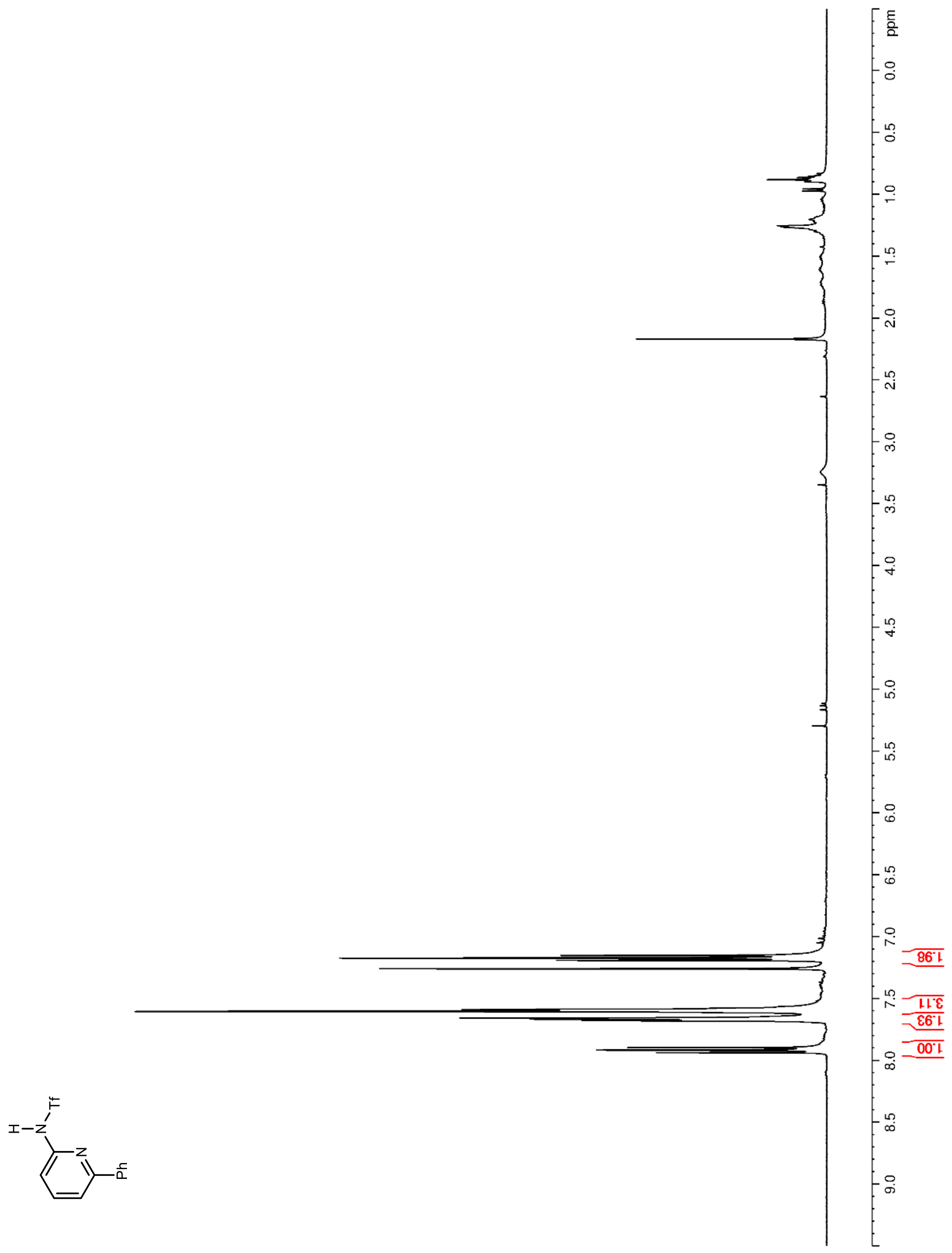

Figure 95. ^{13}C NMR (150 MHz, acetone-d_6) of **G3**

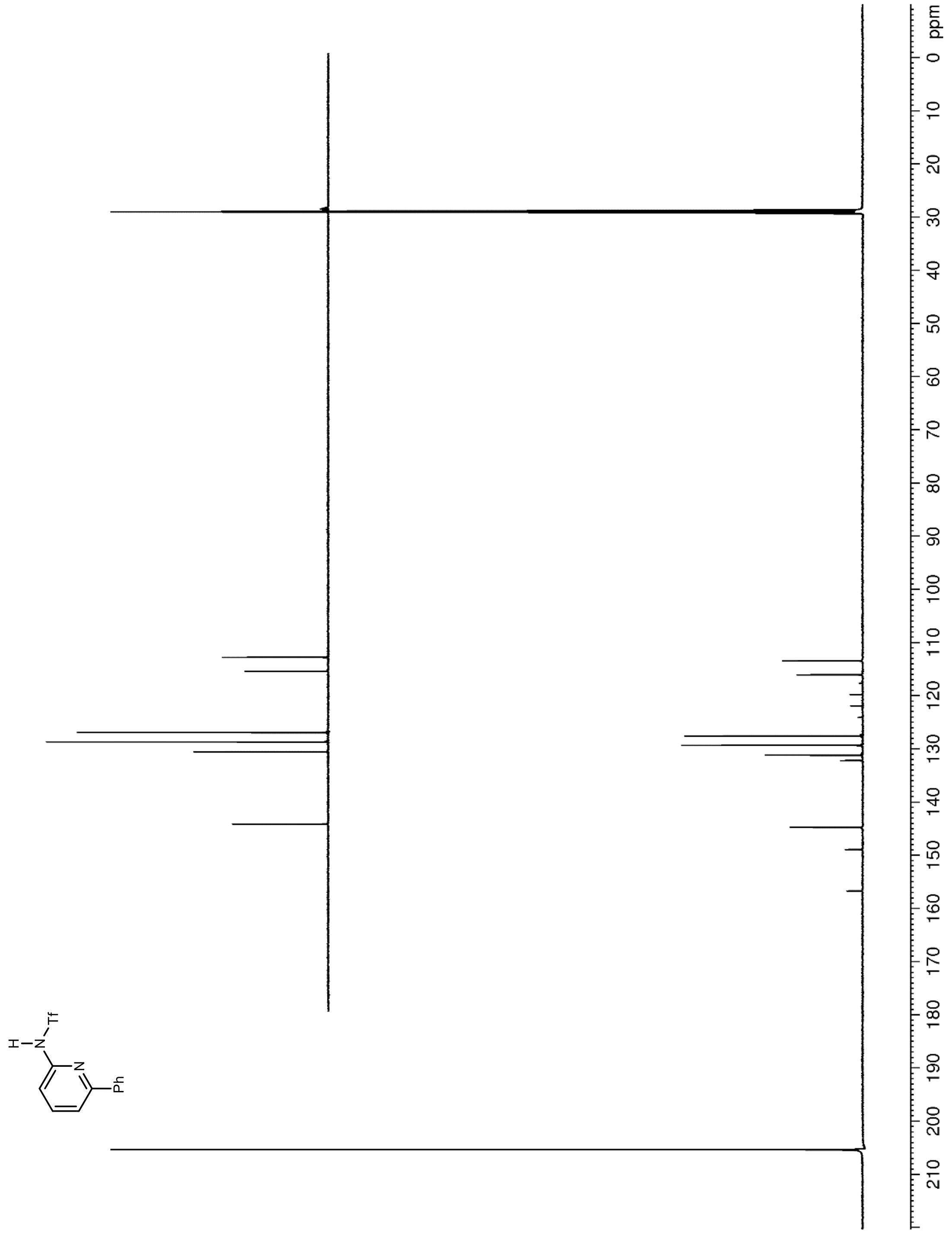

Figure 96. ^{19}F NMR (282 MHz, $CDCl_3$) of **G3**

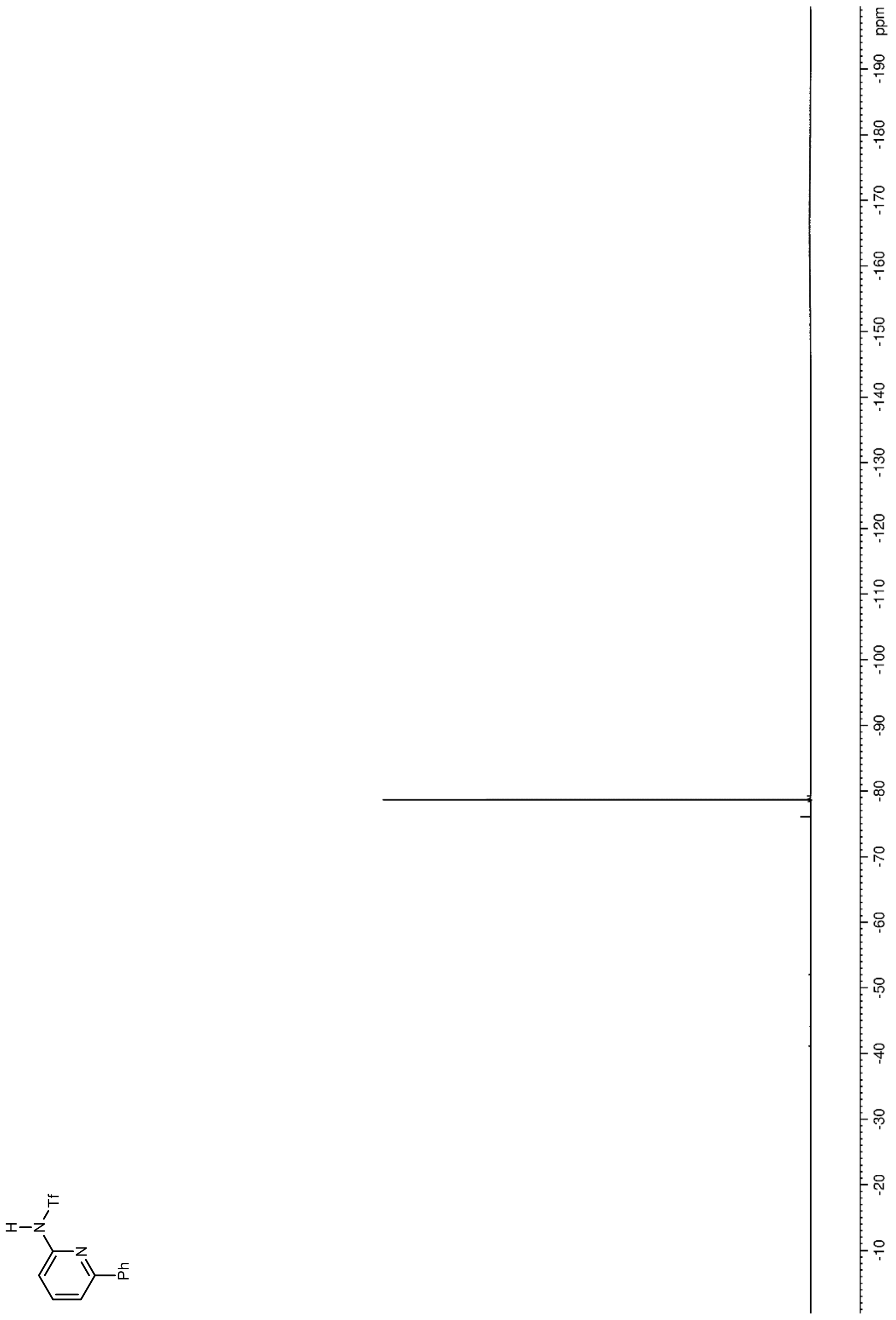

Figure 97. ^{1}H NMR (400 MHz, acetone-d_6) of **G4**

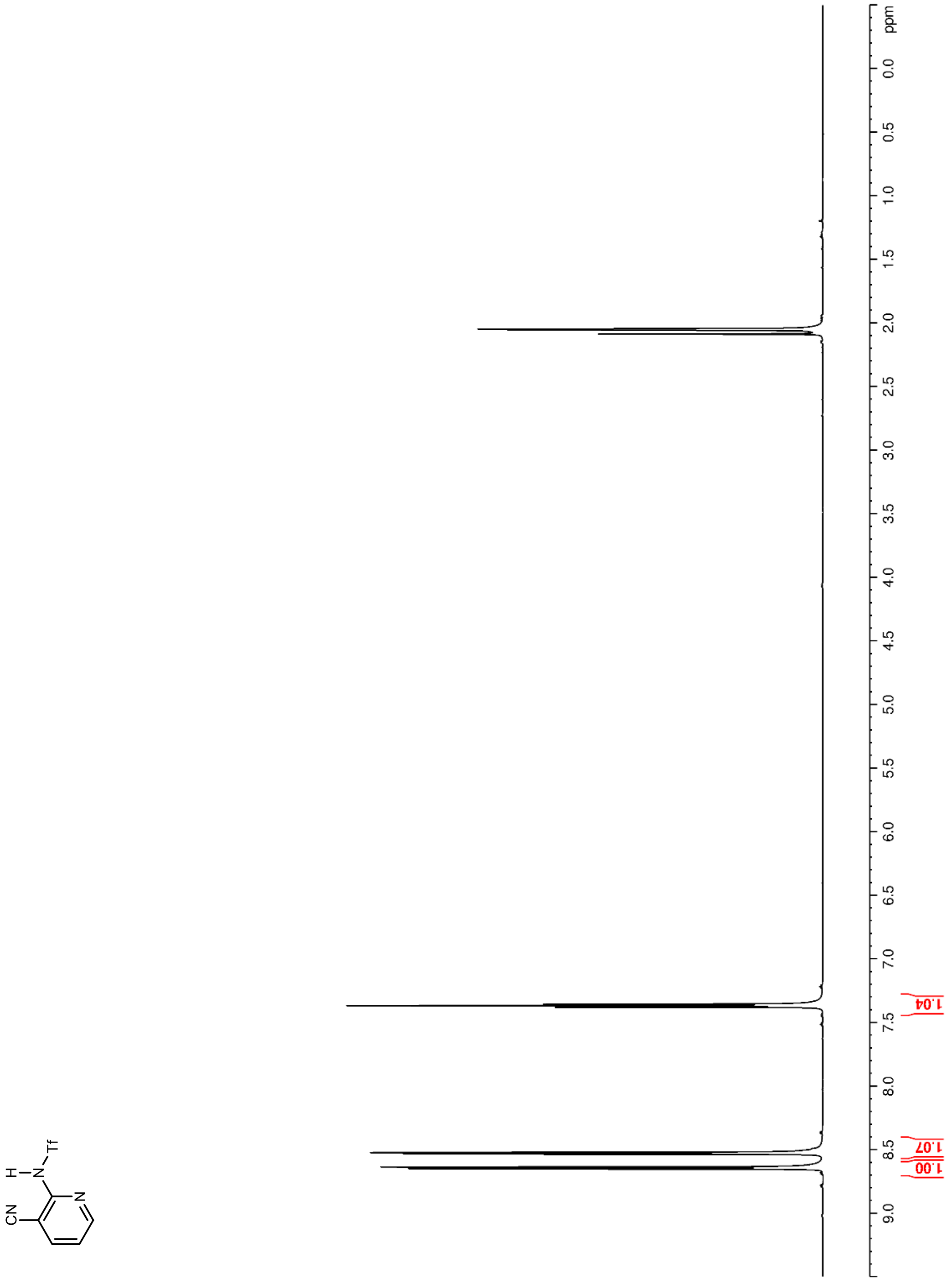

Figure 98. ^{13}C NMR (150 MHz, acetone-d_6) of **G4**

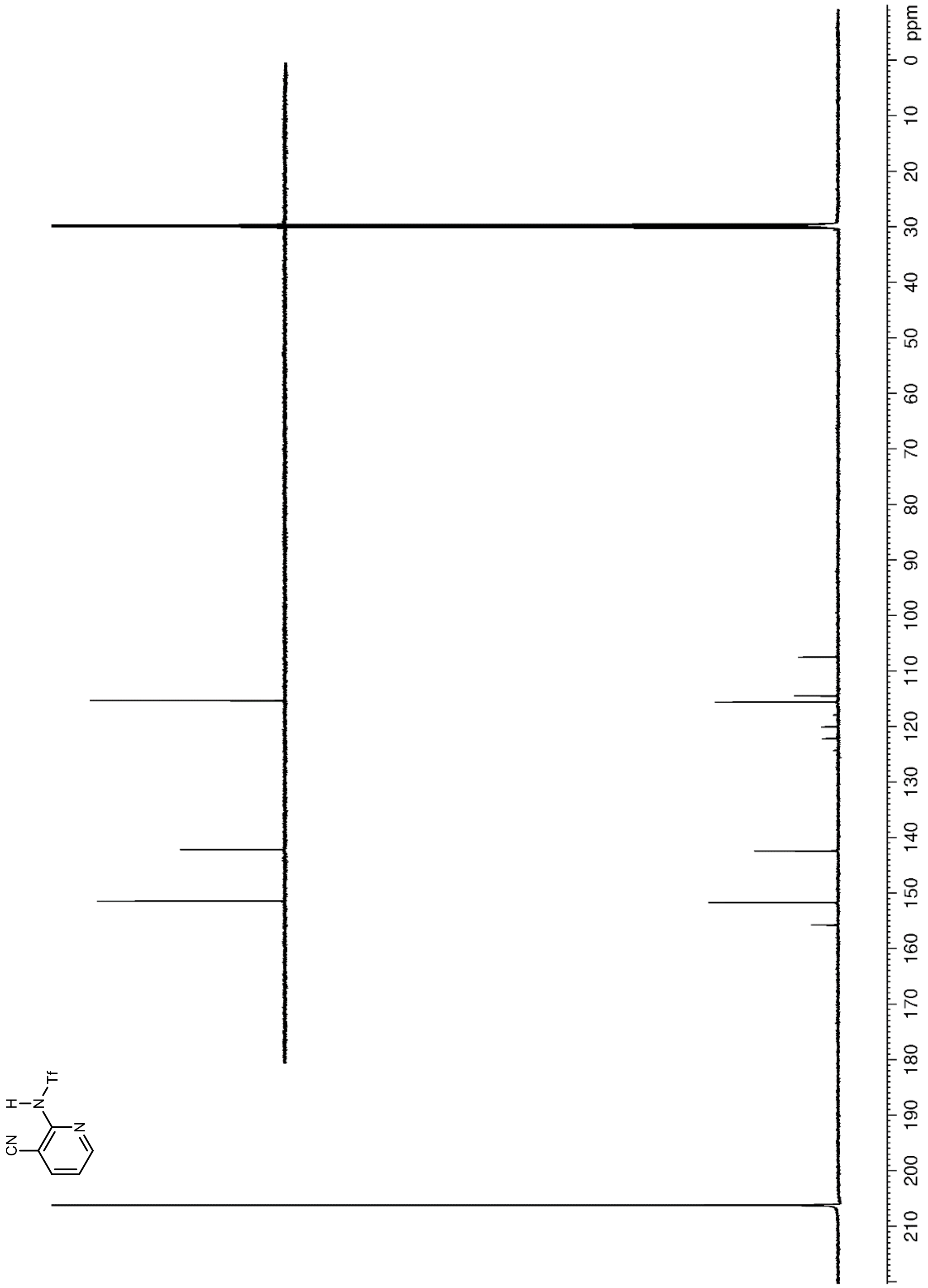

Figure 99. ^{19}F NMR (282 MHz, acetone-d_6) of **G4**

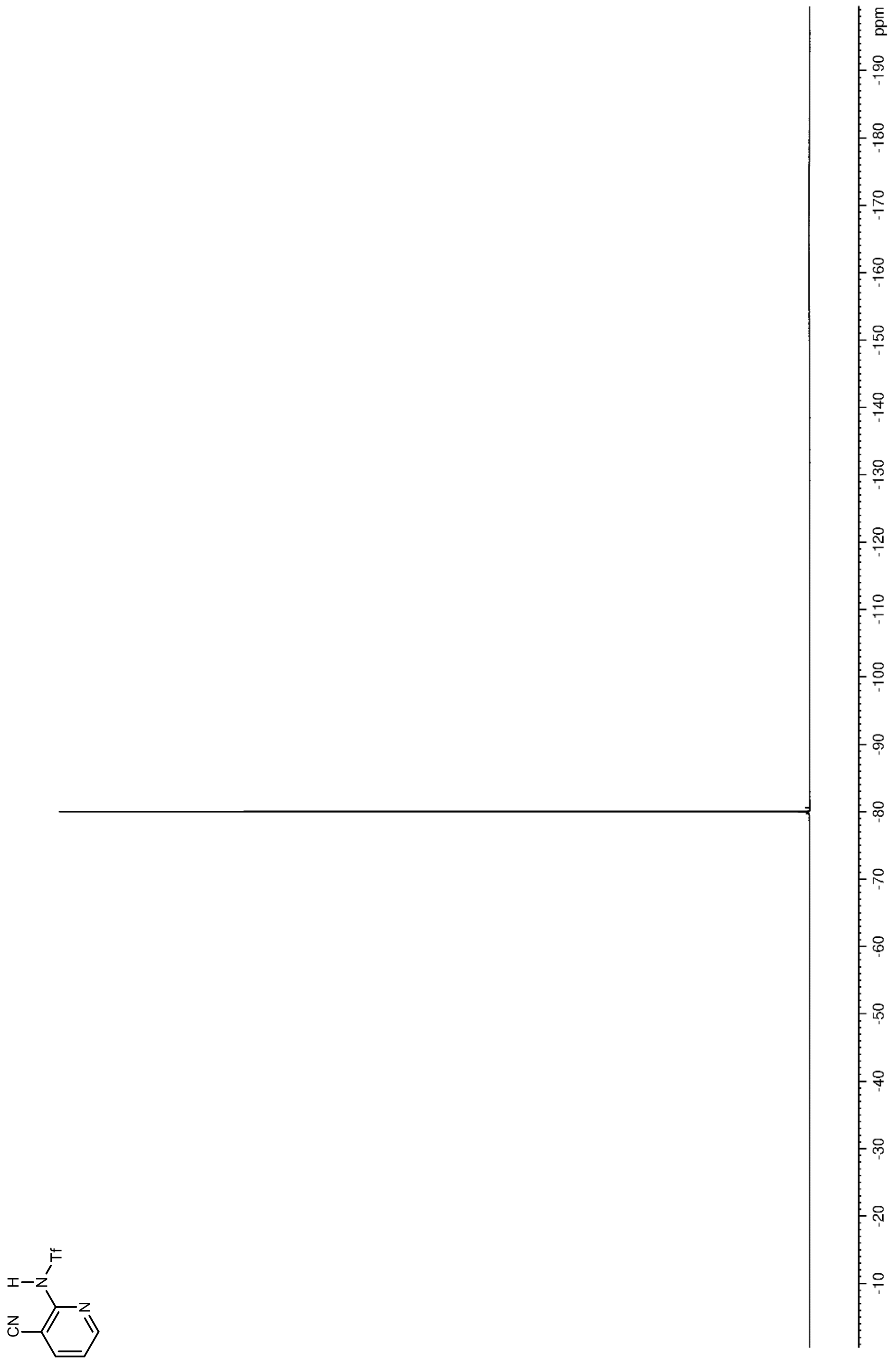

Figure 100. ^{1}H NMR (400 MHz, acetone-d_6) of **G5**

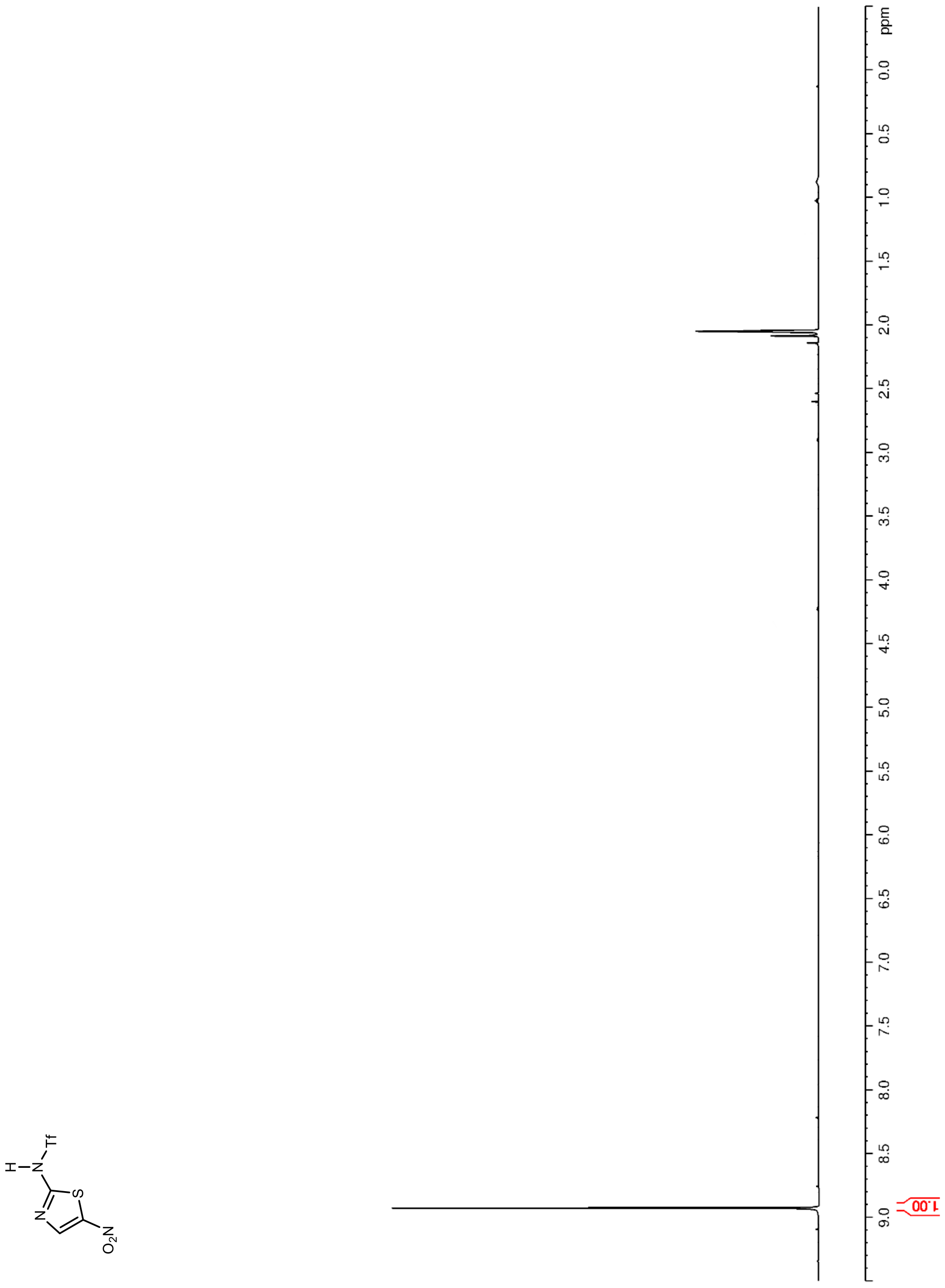

Figure 101. ^{13}C NMR (150 MHz, acetone-d_6) of **G5**

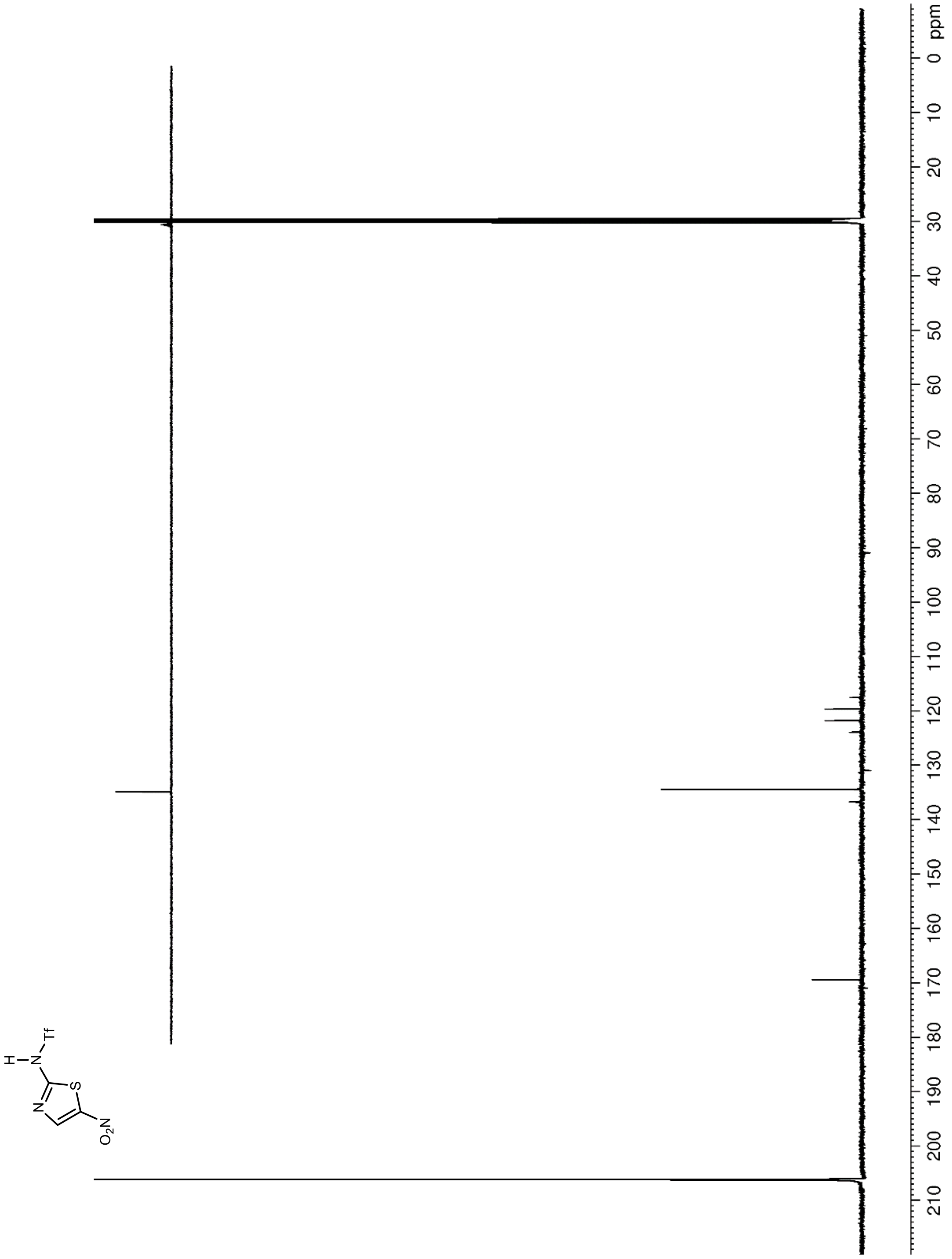

Figure 102. ^{19}F NMR (282 MHz, acetone-d_6) of **G5**

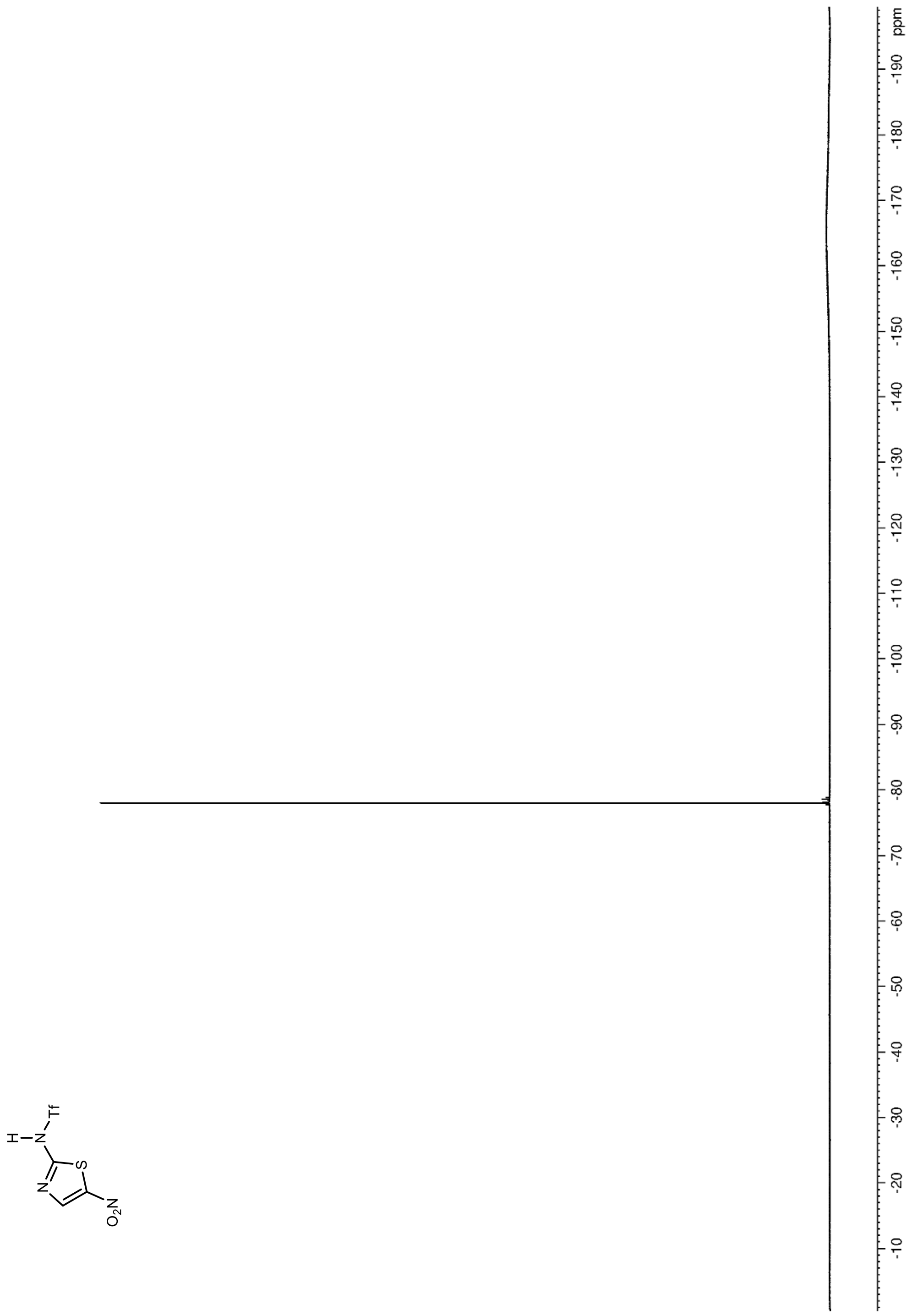

Figure 103. ^{1}H NMR (400 MHz, $CDCl_3$) of **G7**

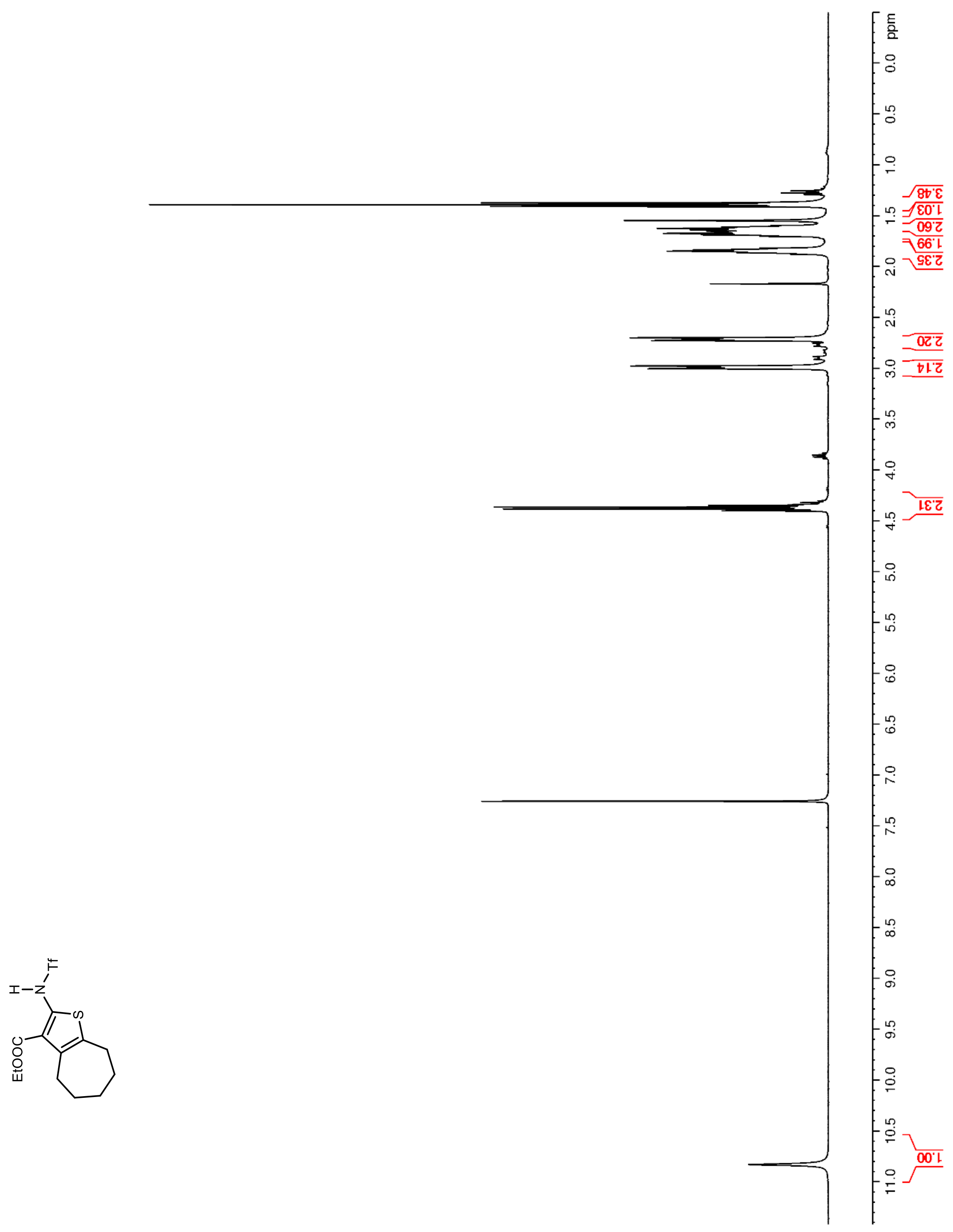

Figure 104. ^{13}C NMR (150 MHz, $CDCl_3$) of **G7**

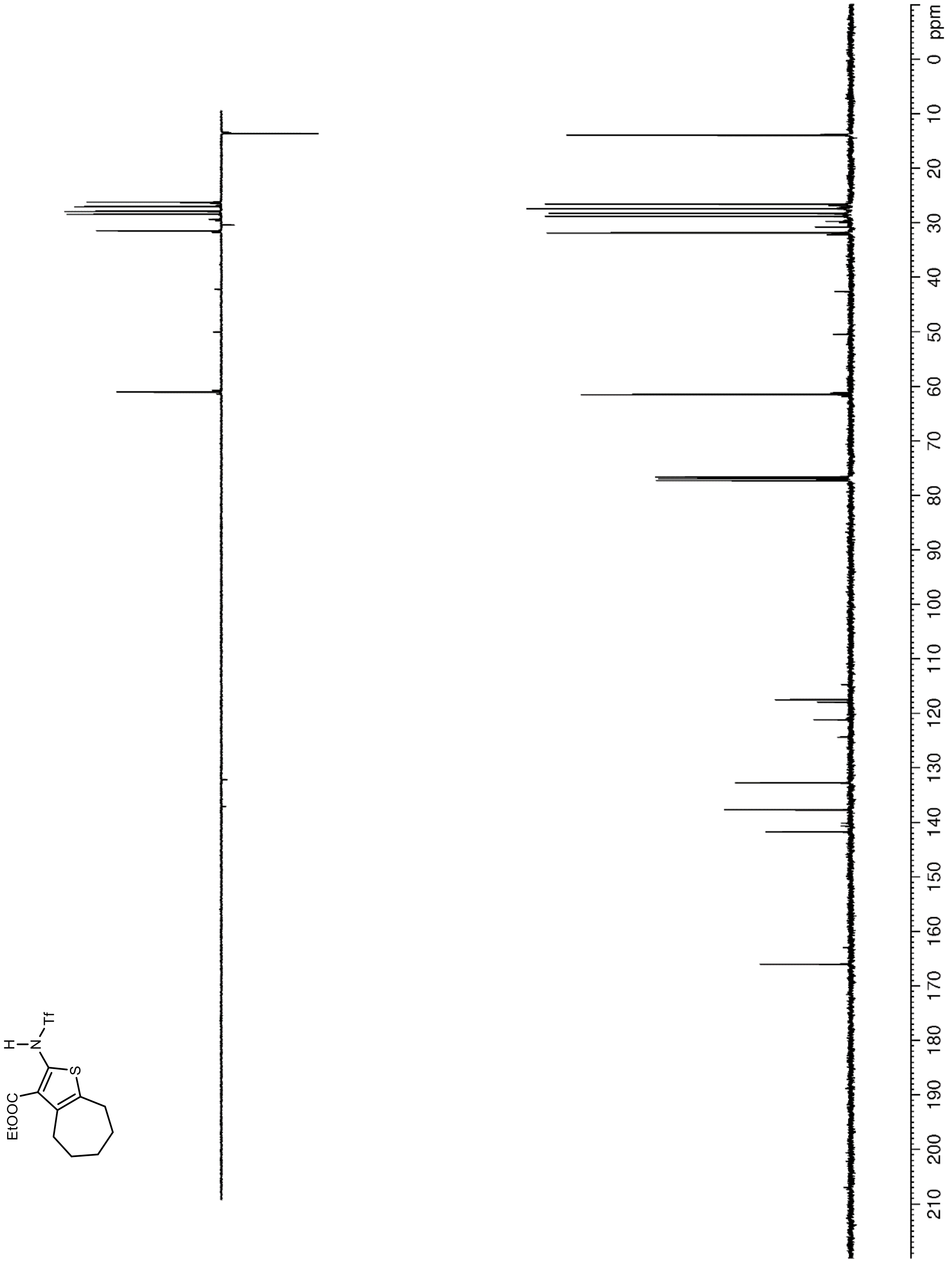

Figure 105. ^{19}F NMR (282 MHz, $CDCl_3$) of **G7**

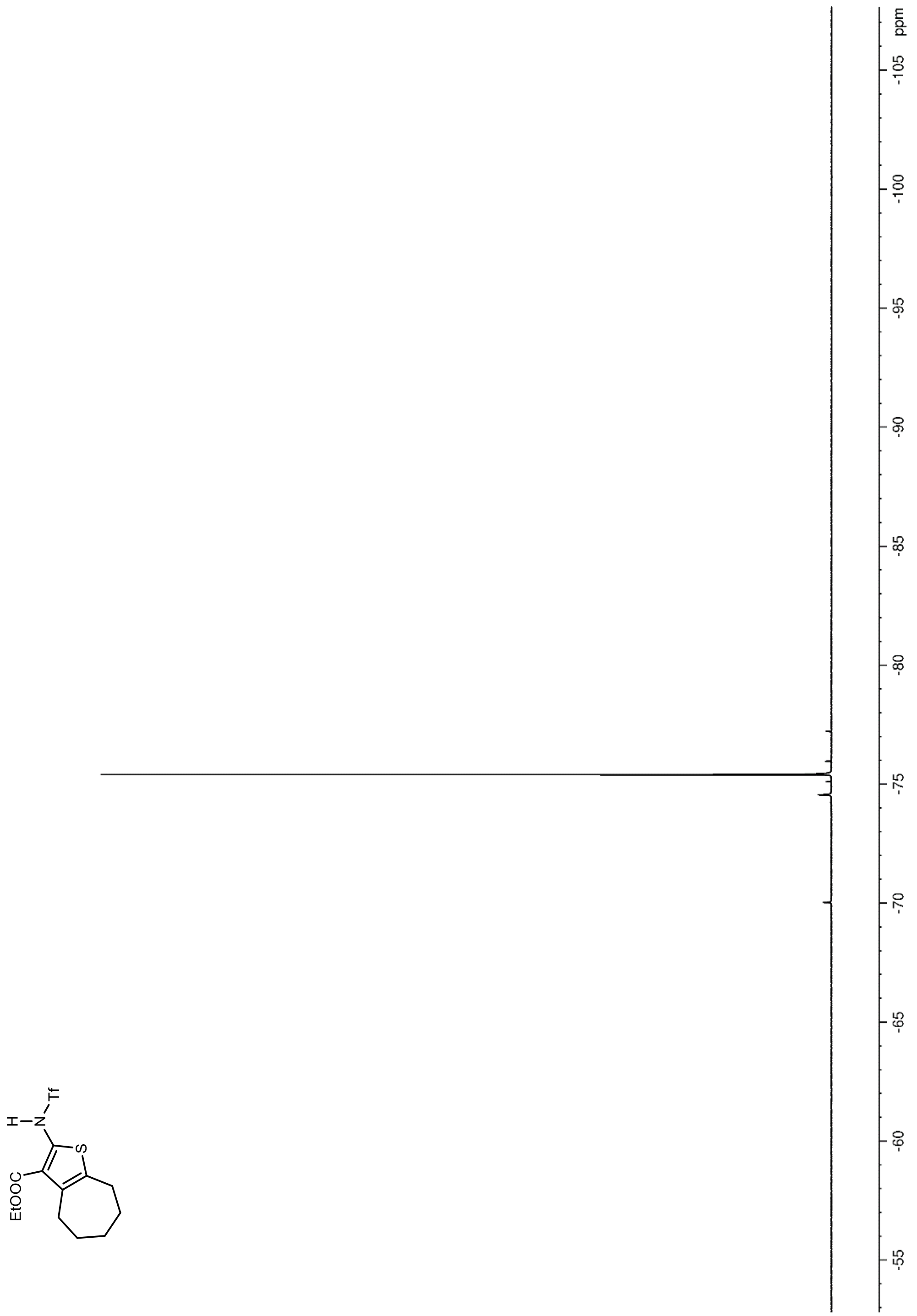

Figure 106. ^{1}H NMR (400 MHz, $CDCl_3$) of **G8**

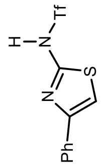

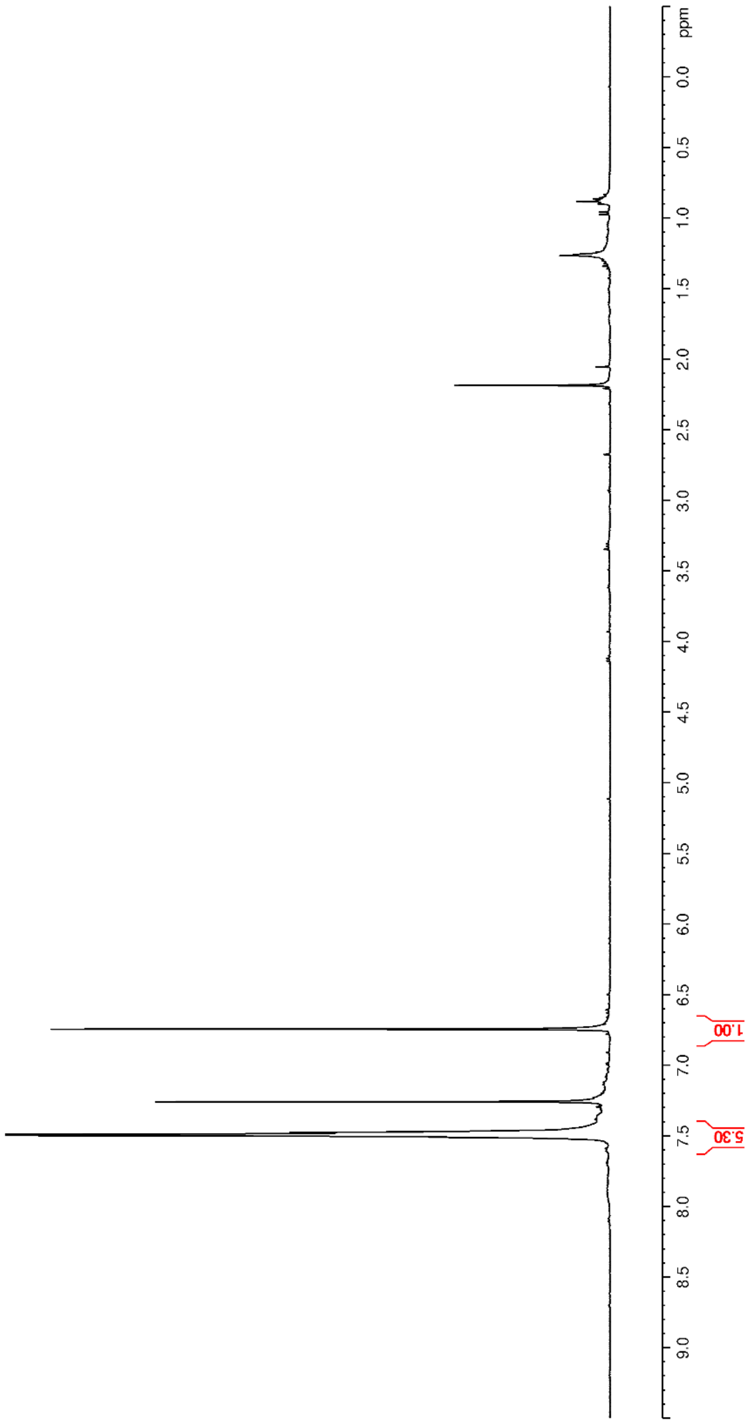

Figure 107. ^{13}C NMR (150 MHz, acetone-d_6) of **G8**

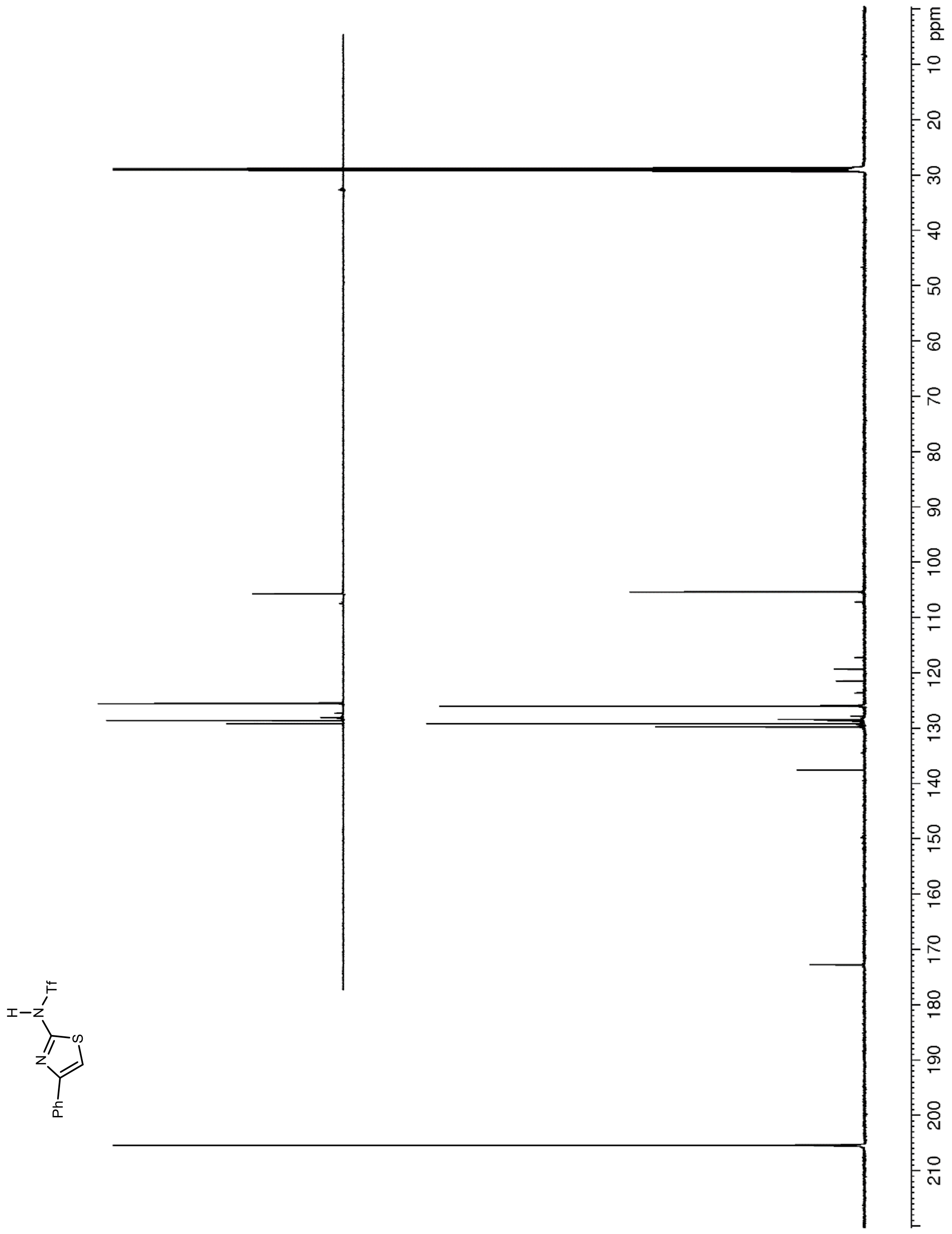

Figure 108. ^{19}F NMR (282 MHz, $CDCl_3$) of **G8**

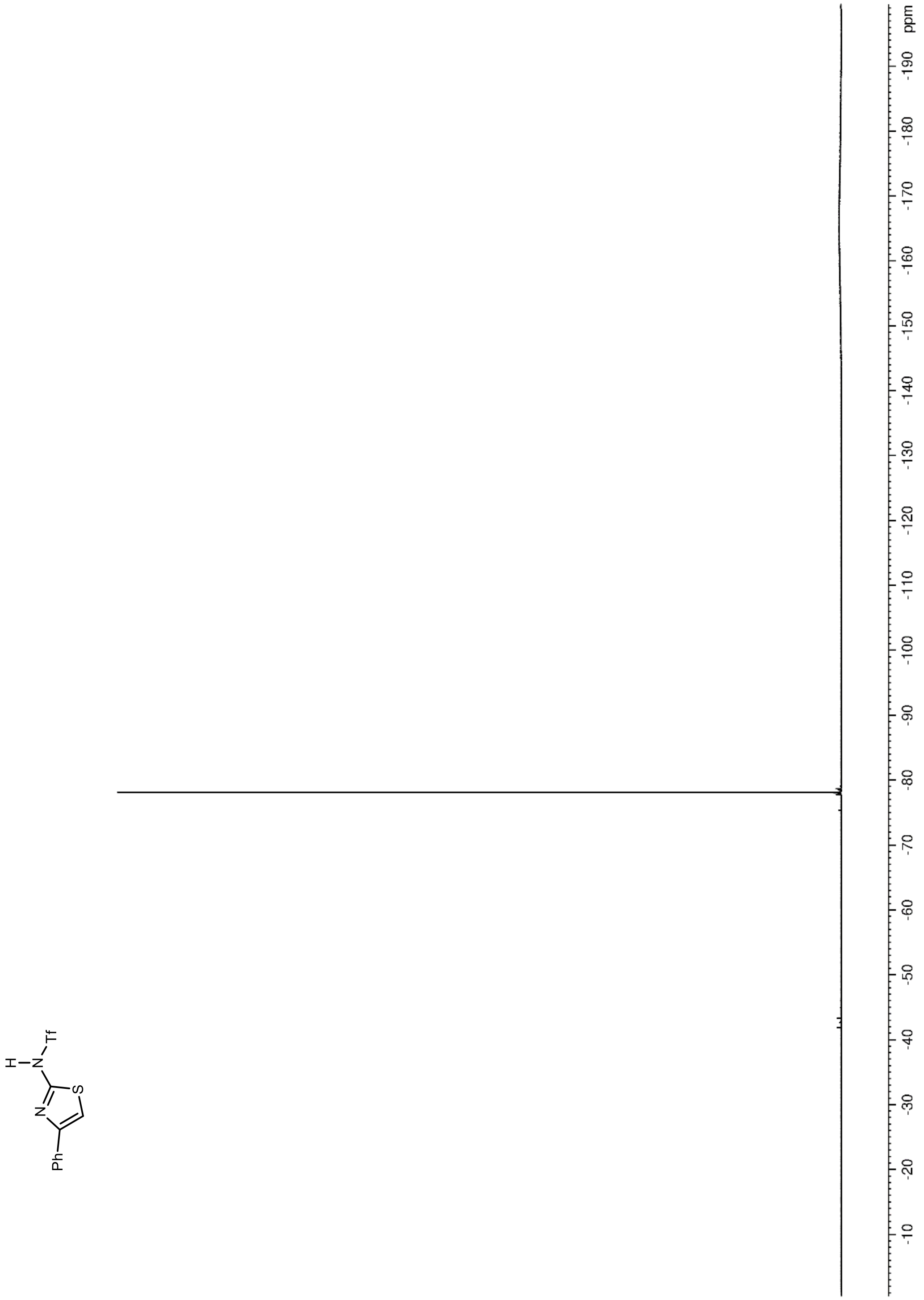

Figure 109. ^{1}H NMR (400 MHz, $CDCl_3$) of **H1**

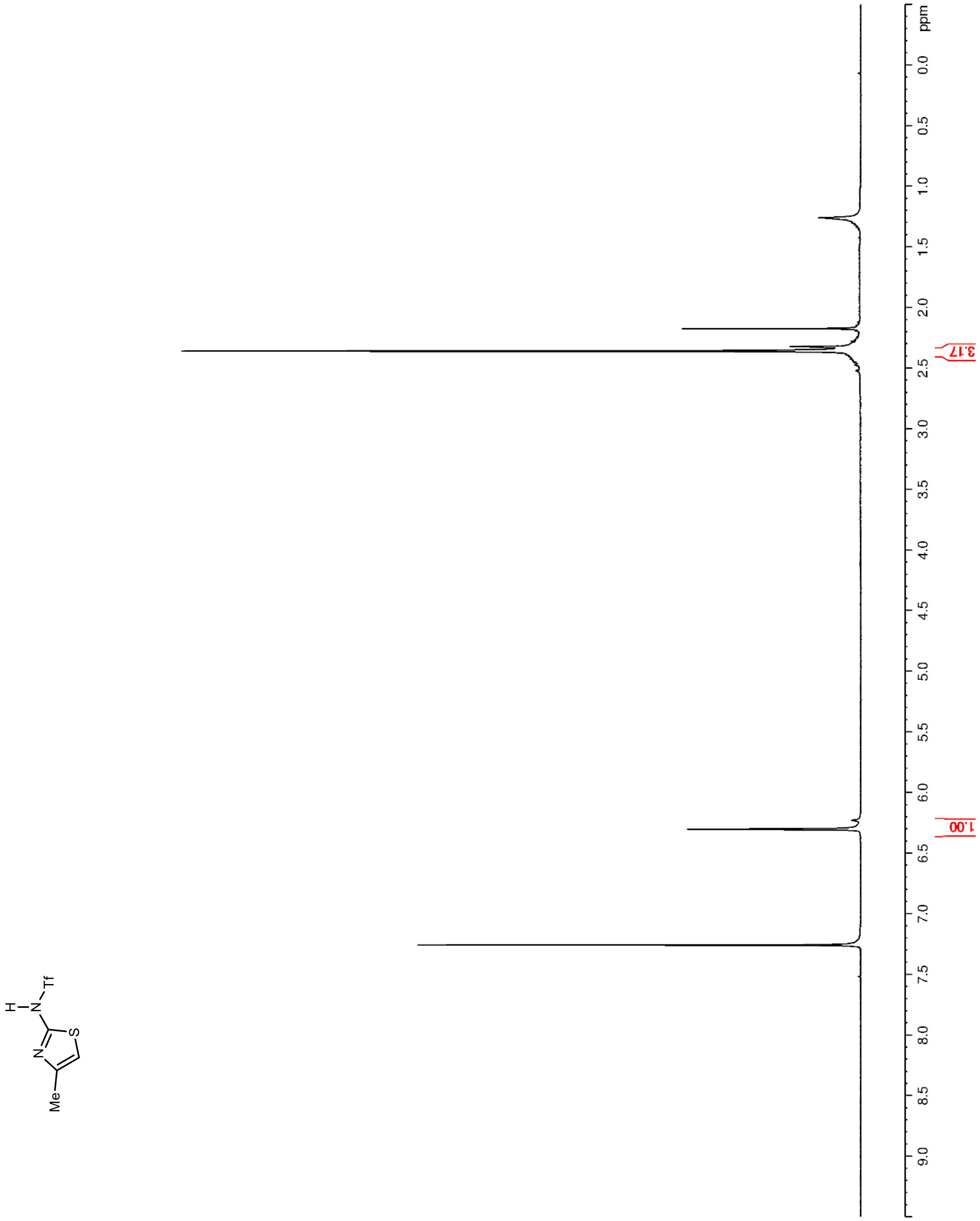

Figure 110. ^{13}C NMR (150 MHz, acetone-d_6) of **H1**

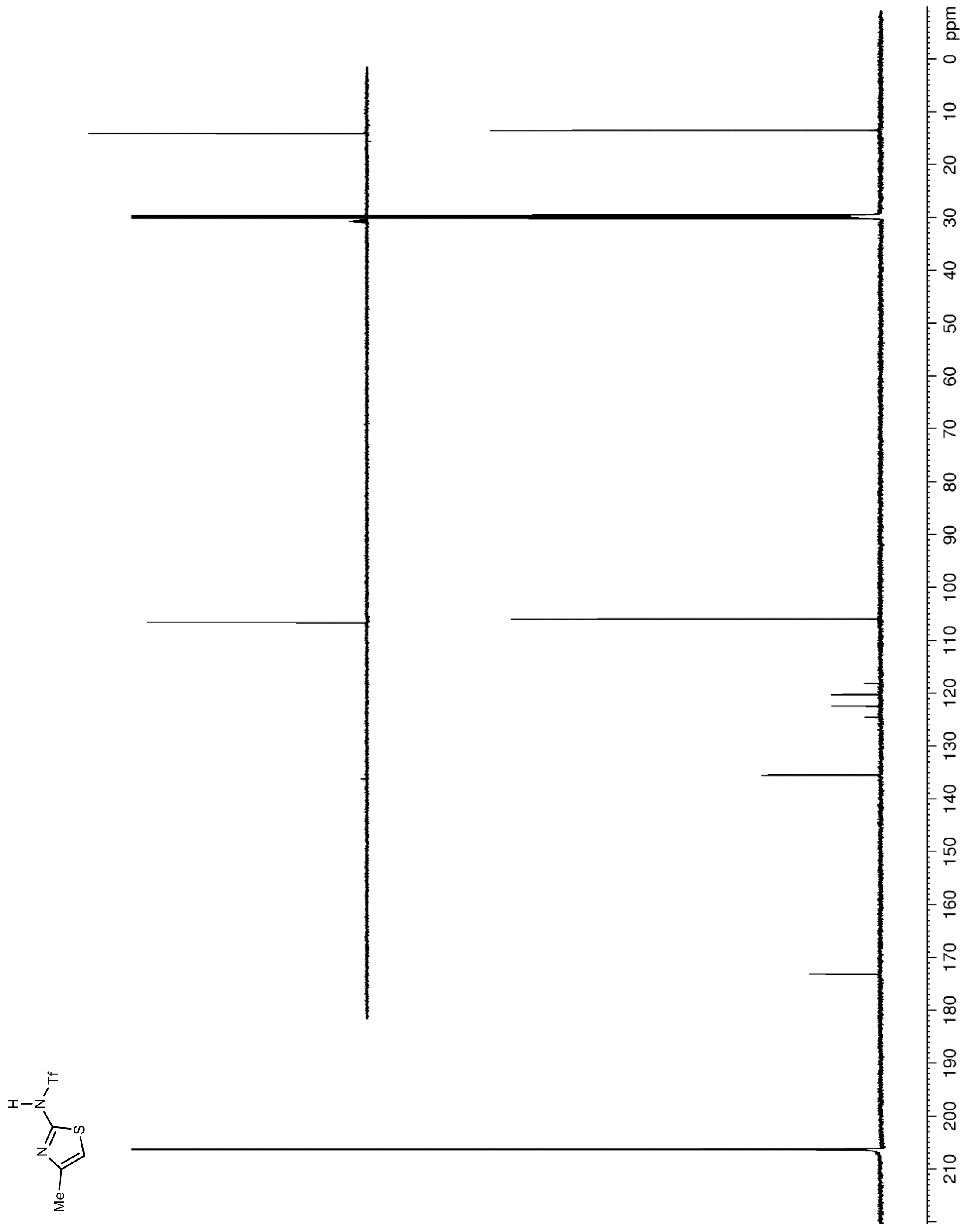

Figure 111. ^{19}F NMR (282 MHz, $CDCl_3$) of **H1**

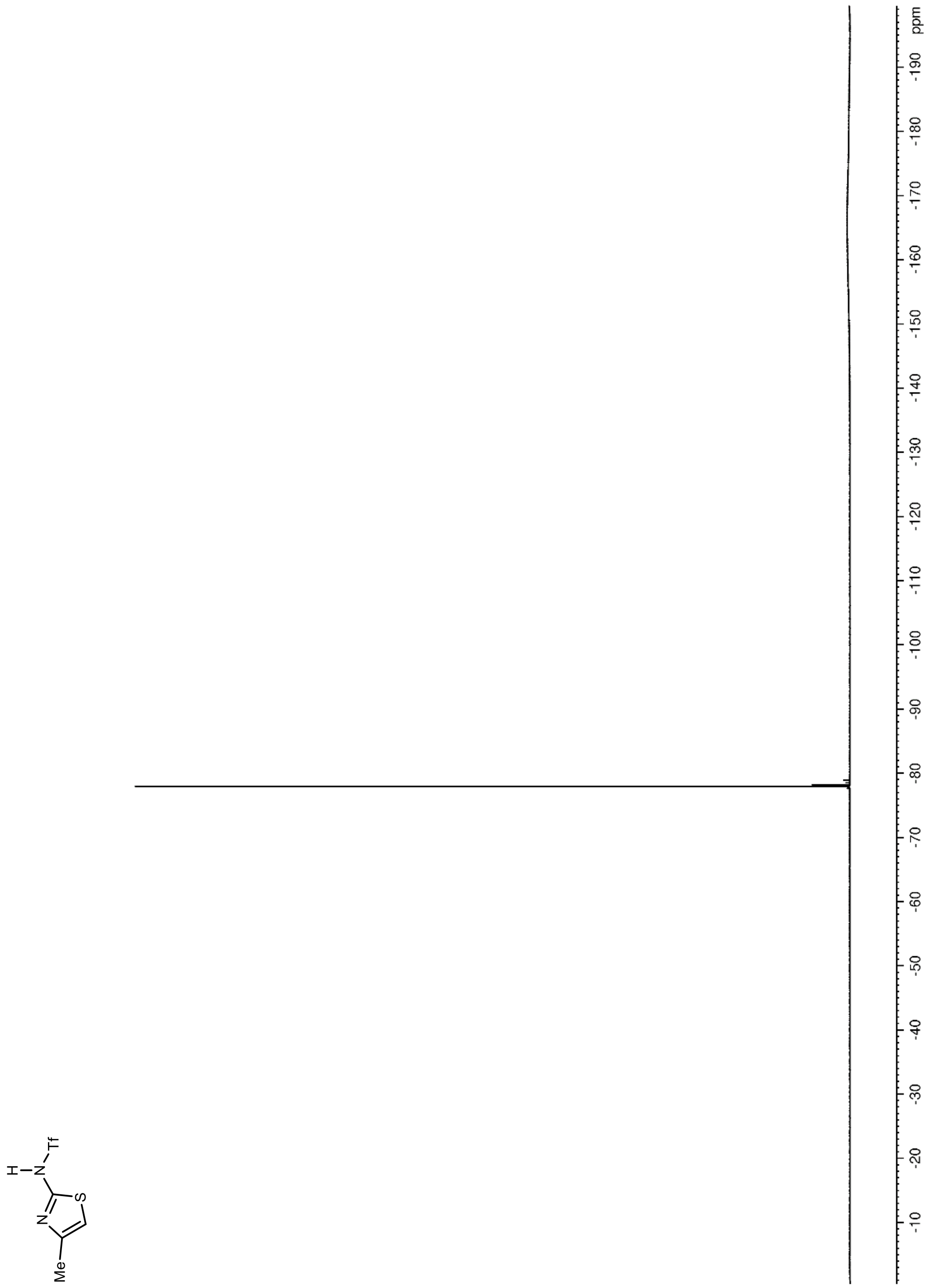

Figure 112. ^{1}H NMR (400 MHz, acetone-d_6) of **H2**

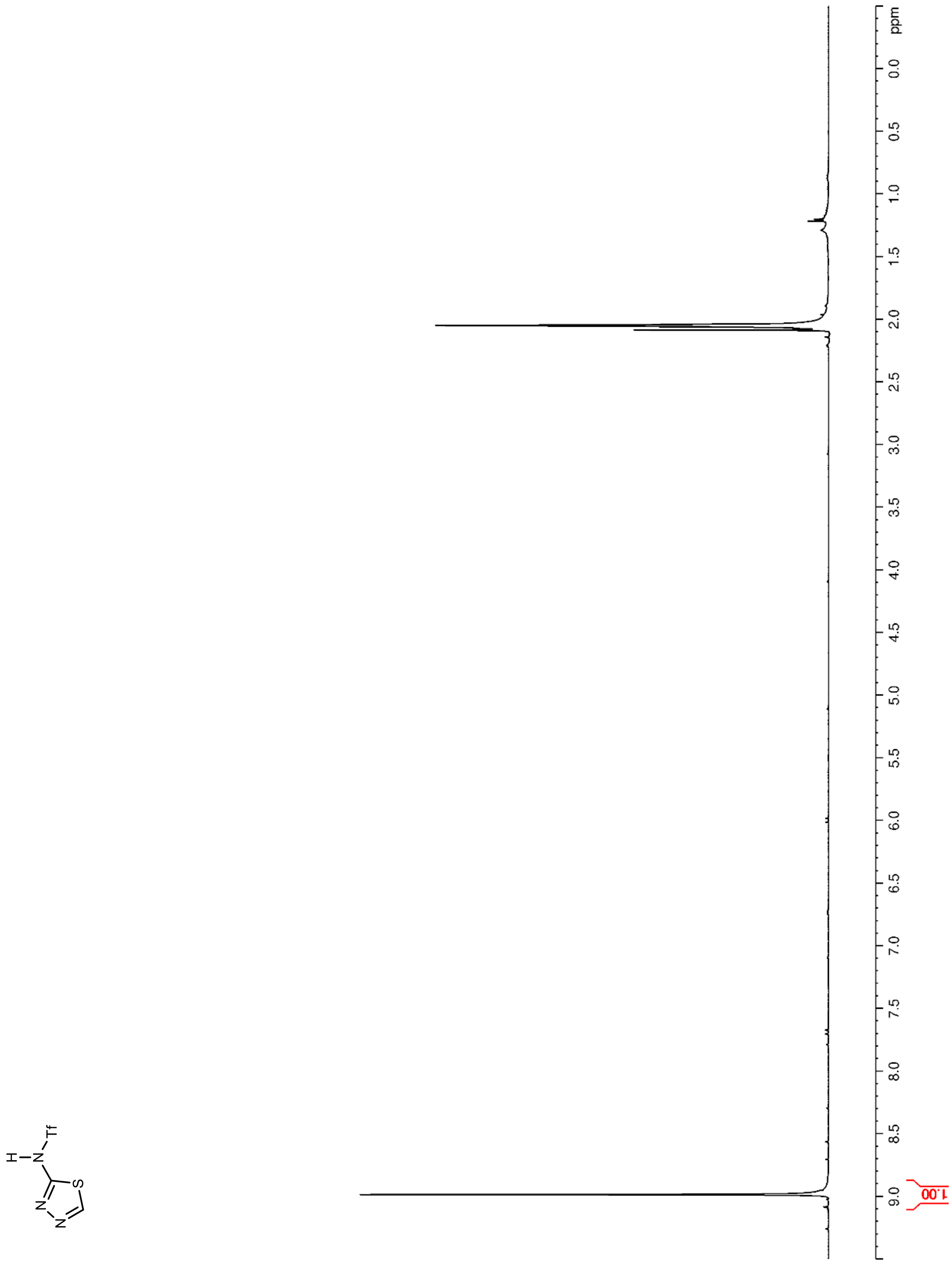

Figure 113. ^{13}C NMR (150 MHz, acetone-d_6) of **H2**

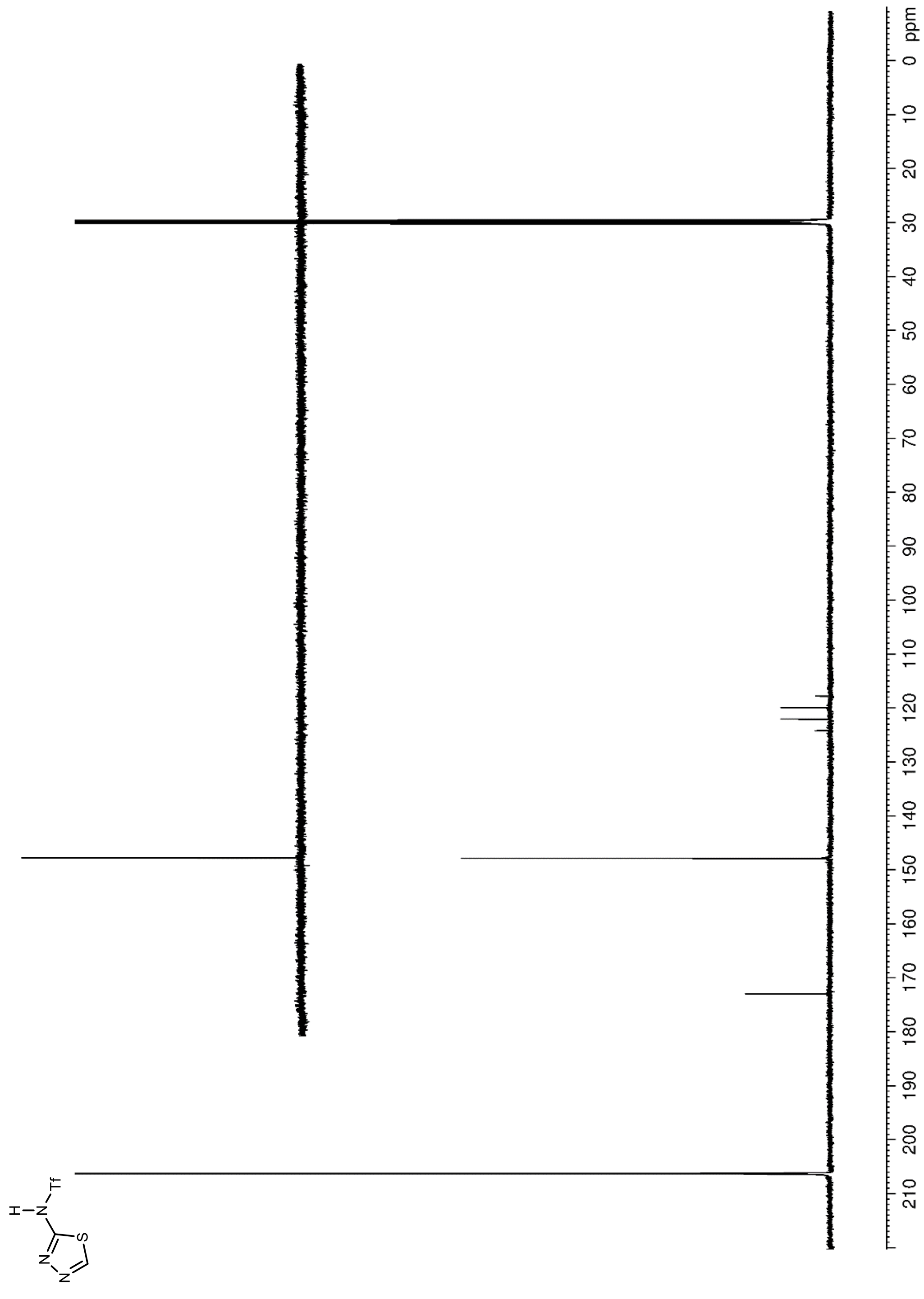

Figure 114. ^{19}F NMR (282 MHz, acetone-d_6) of **H2**

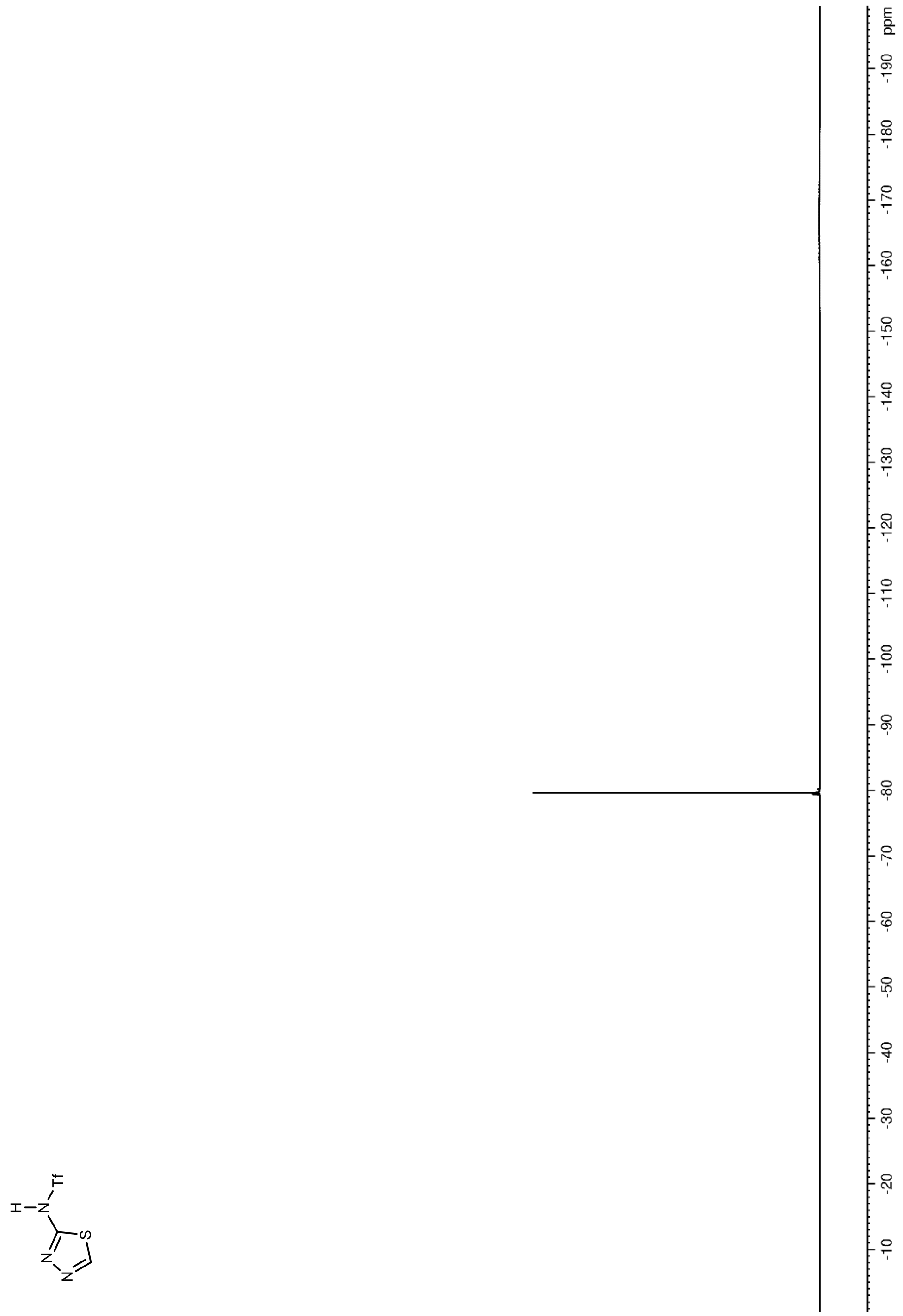

Figure 115. ^{1}H NMR (400 MHz, acetone-d_6) of **H3**

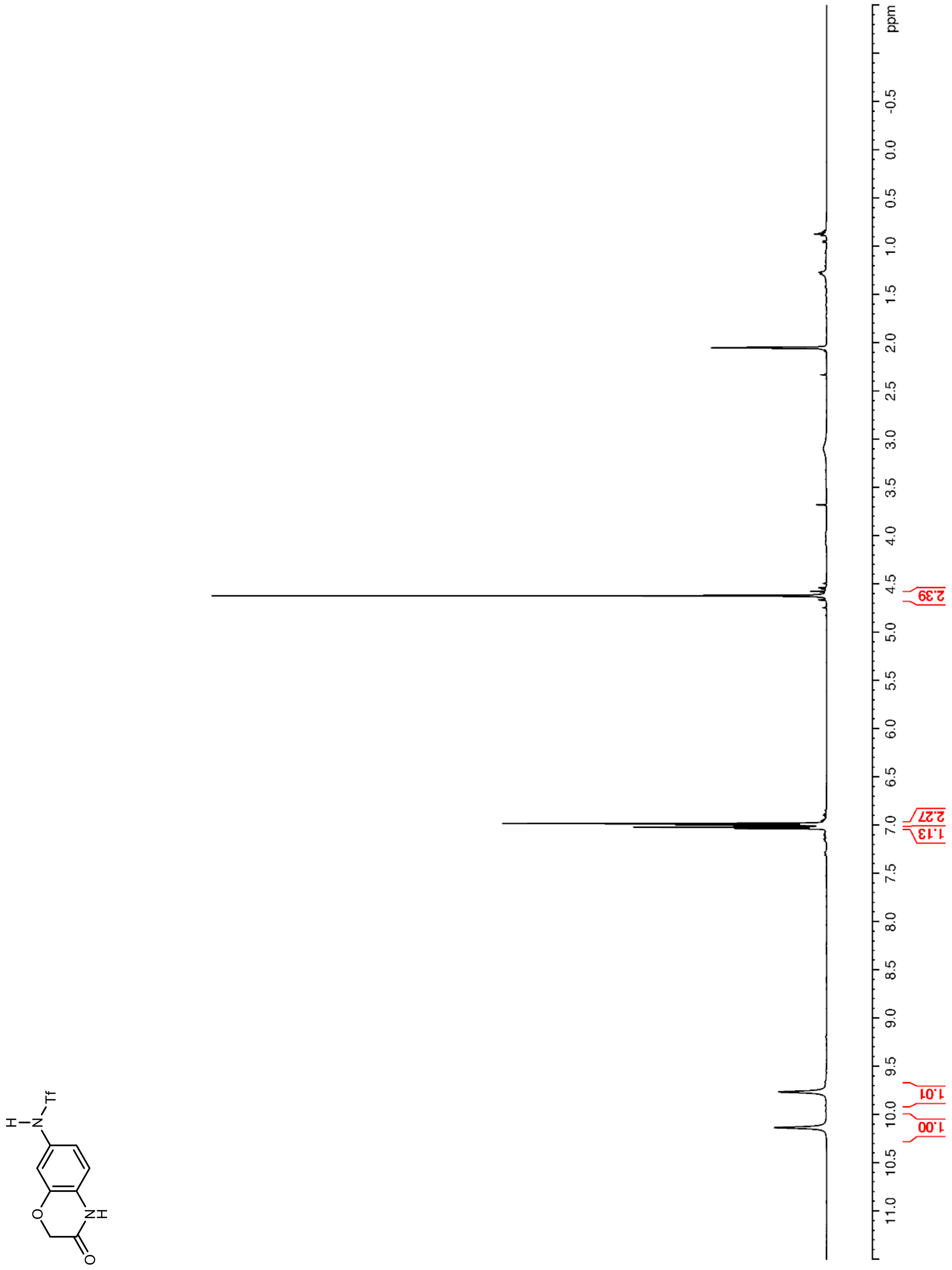

Figure 116. ^{13}C NMR (150 MHz, acetone-d_6) of **H3**

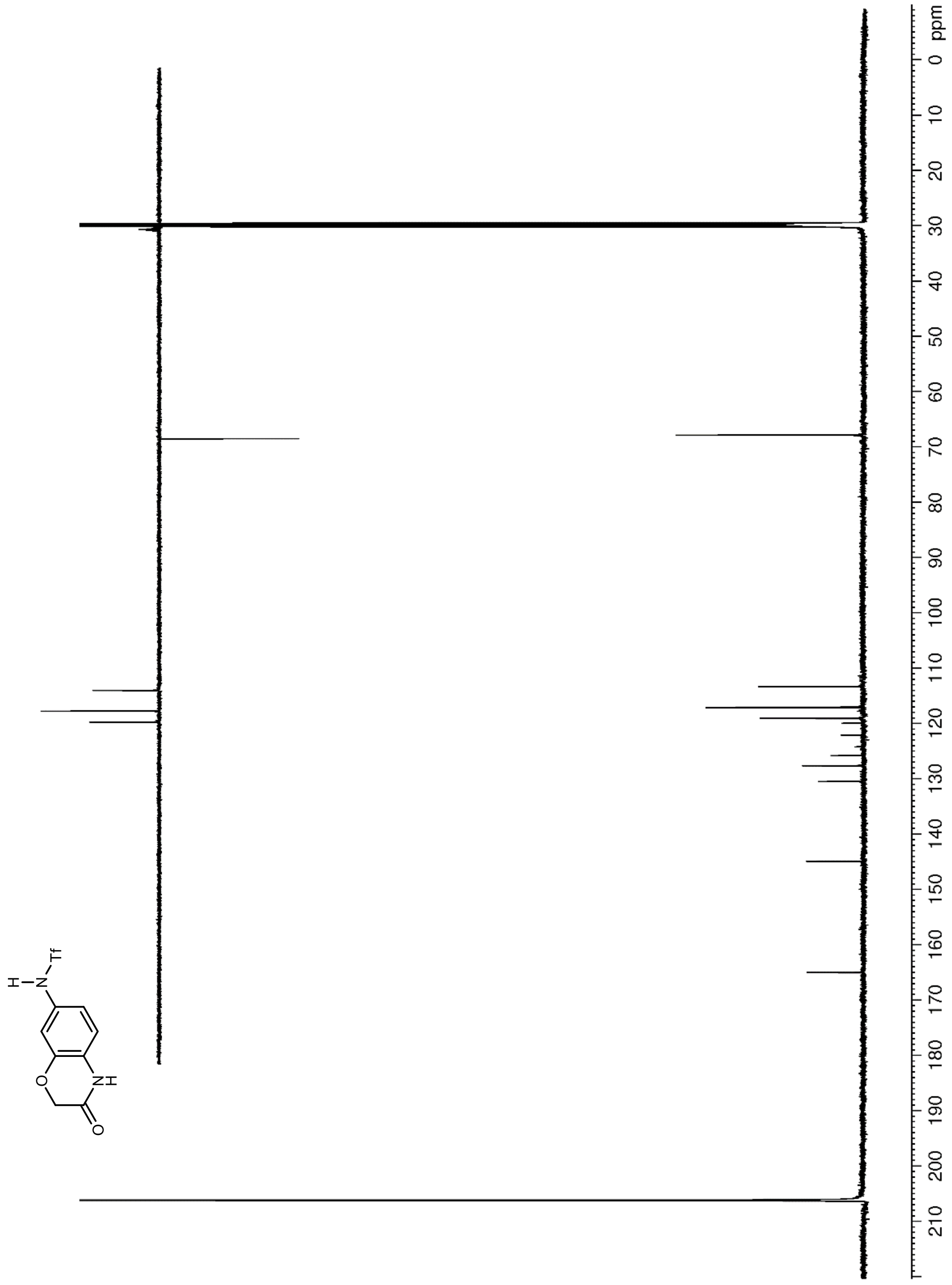

Figure 117. ^{19}F NMR (282 MHz, acetone-d_6) of **H3**

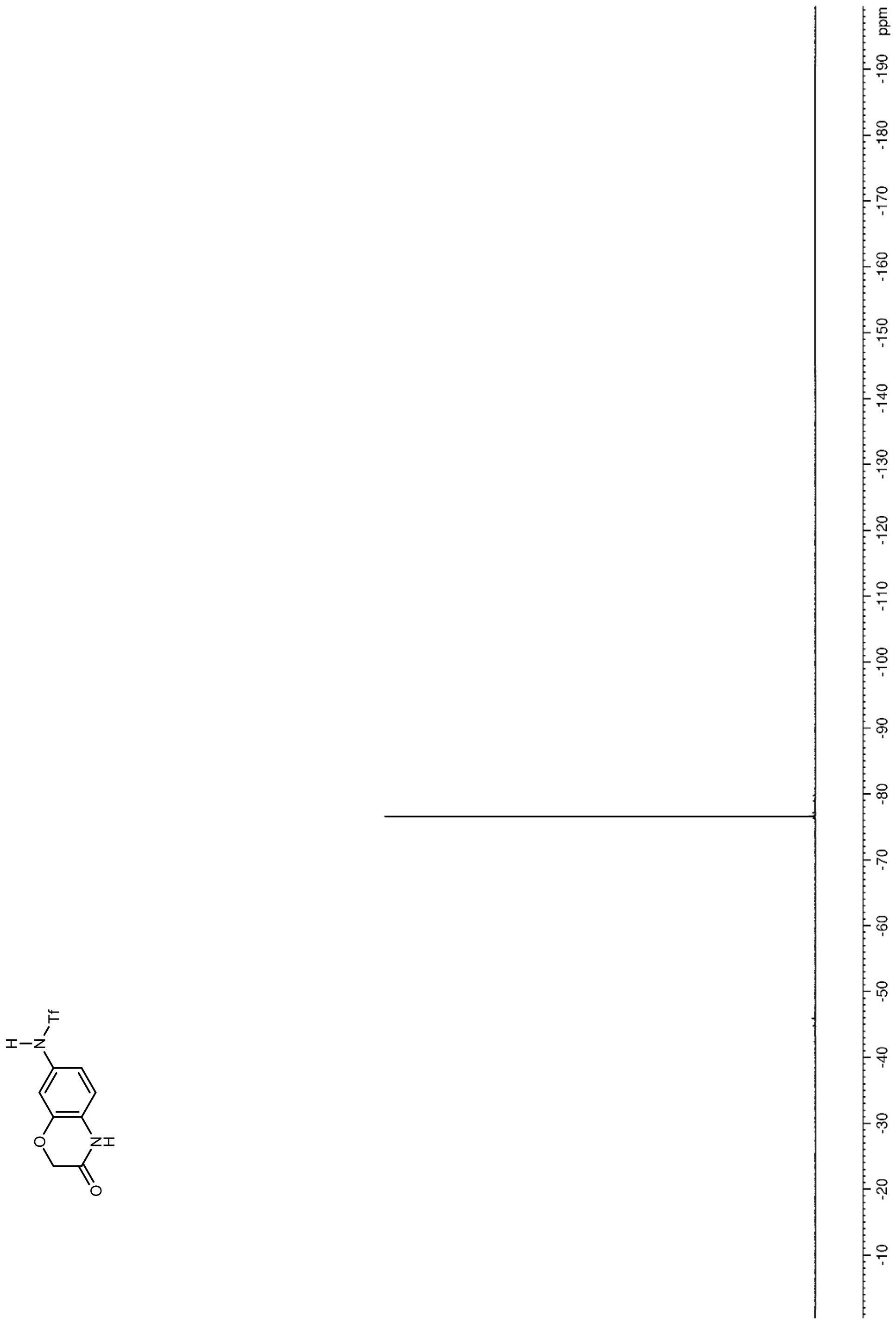

Figure 118. ^{1}H NMR (400 MHz, $CDCl_3$) of **H4**

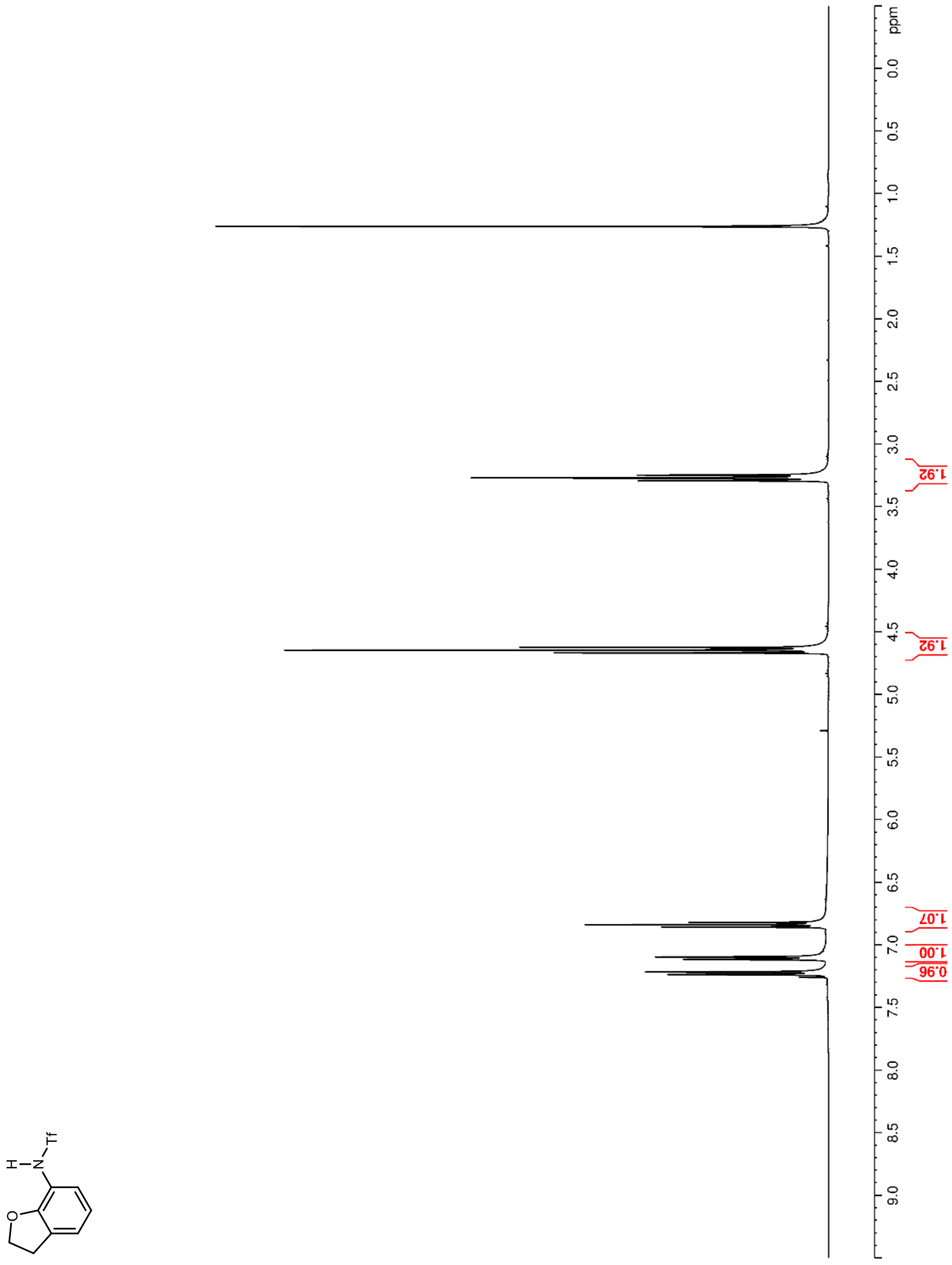

Figure 119. ^{13}C NMR (150 MHz, $CDCl_3$) of **H4**

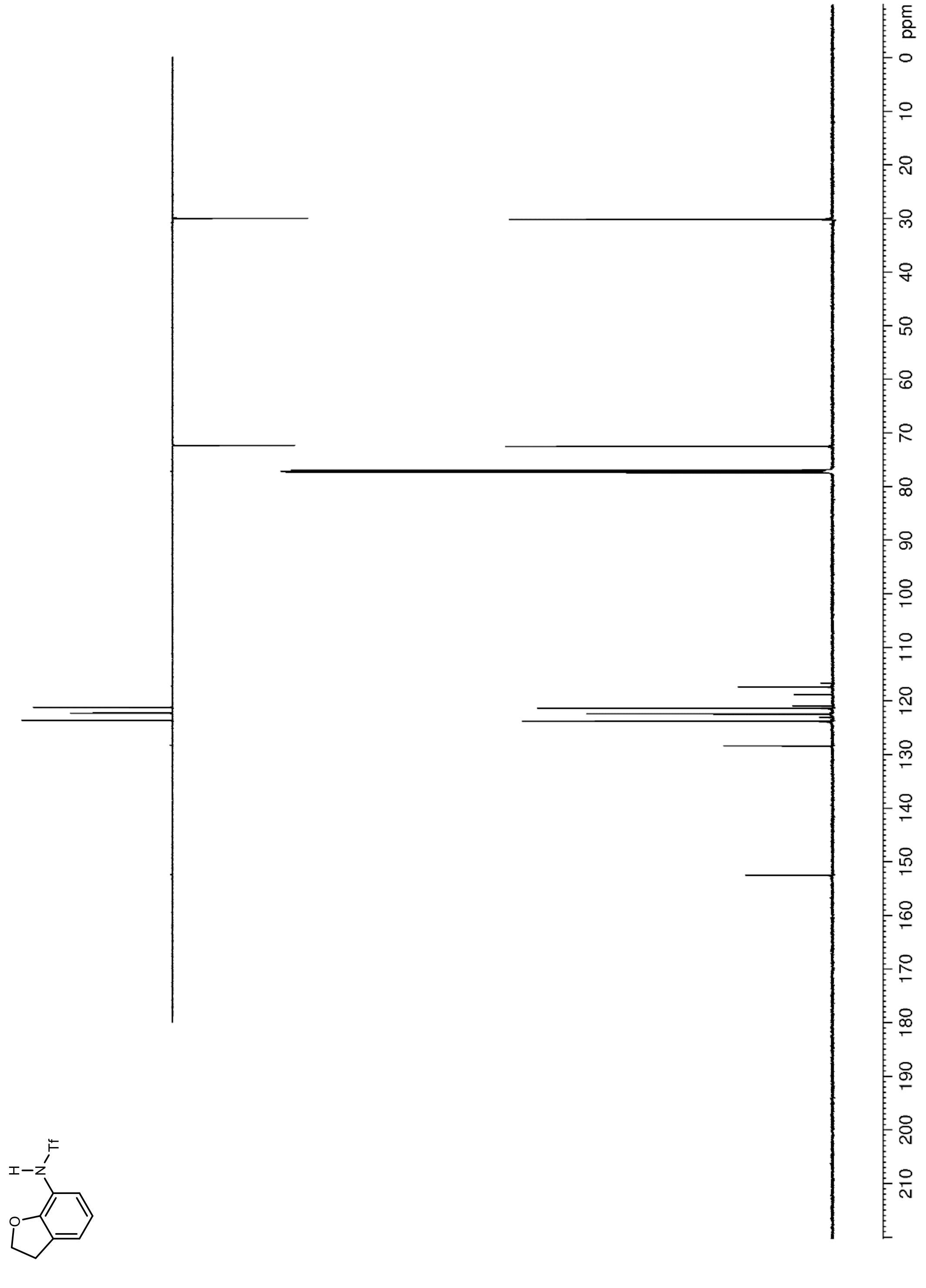

Figure 120. ^{19}F NMR (282 MHz, $CDCl_3$) of **H4**

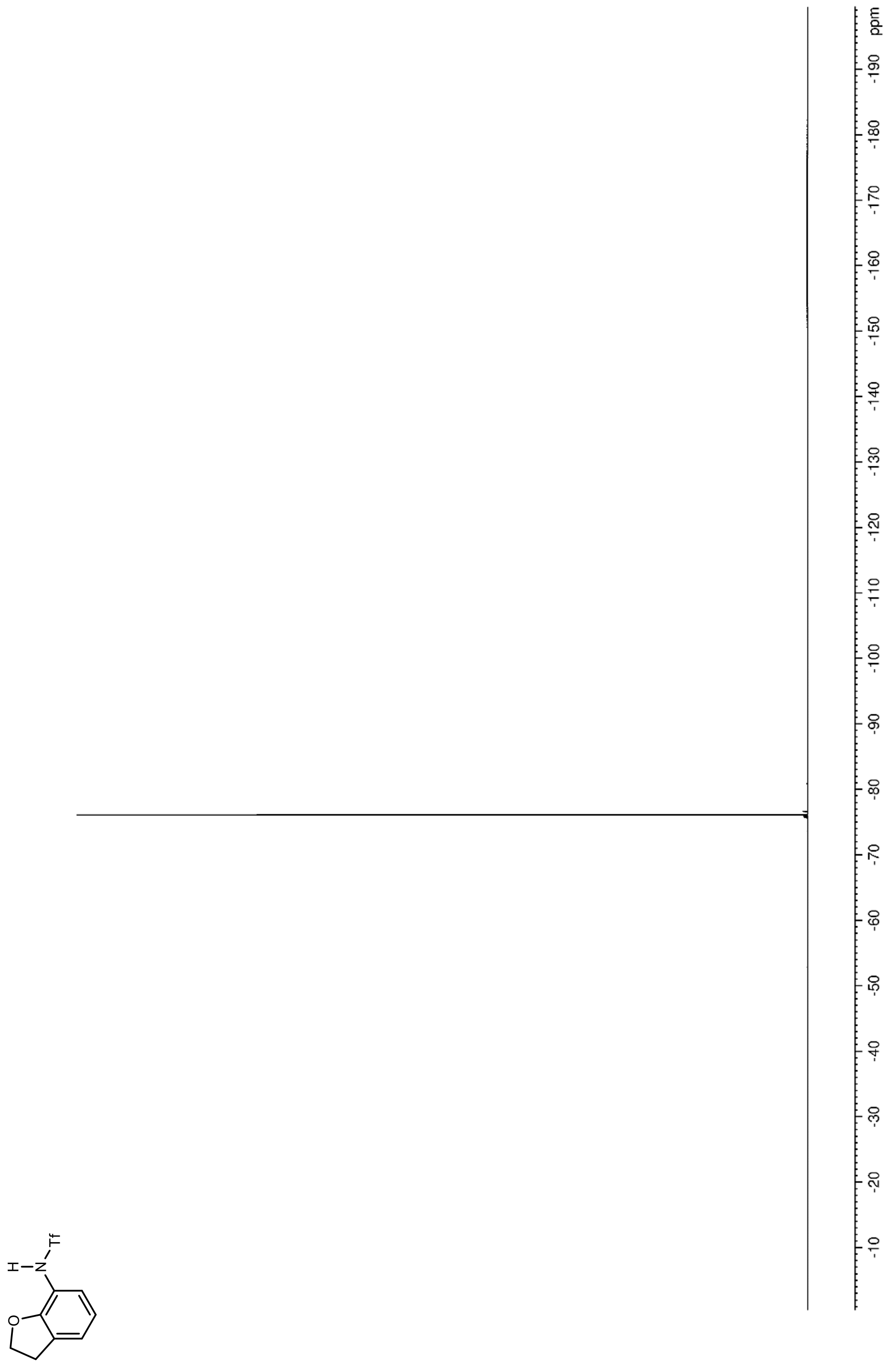

Figure 121. ^{1}H NMR (400 MHz, $CDCl_3$) of **H5**

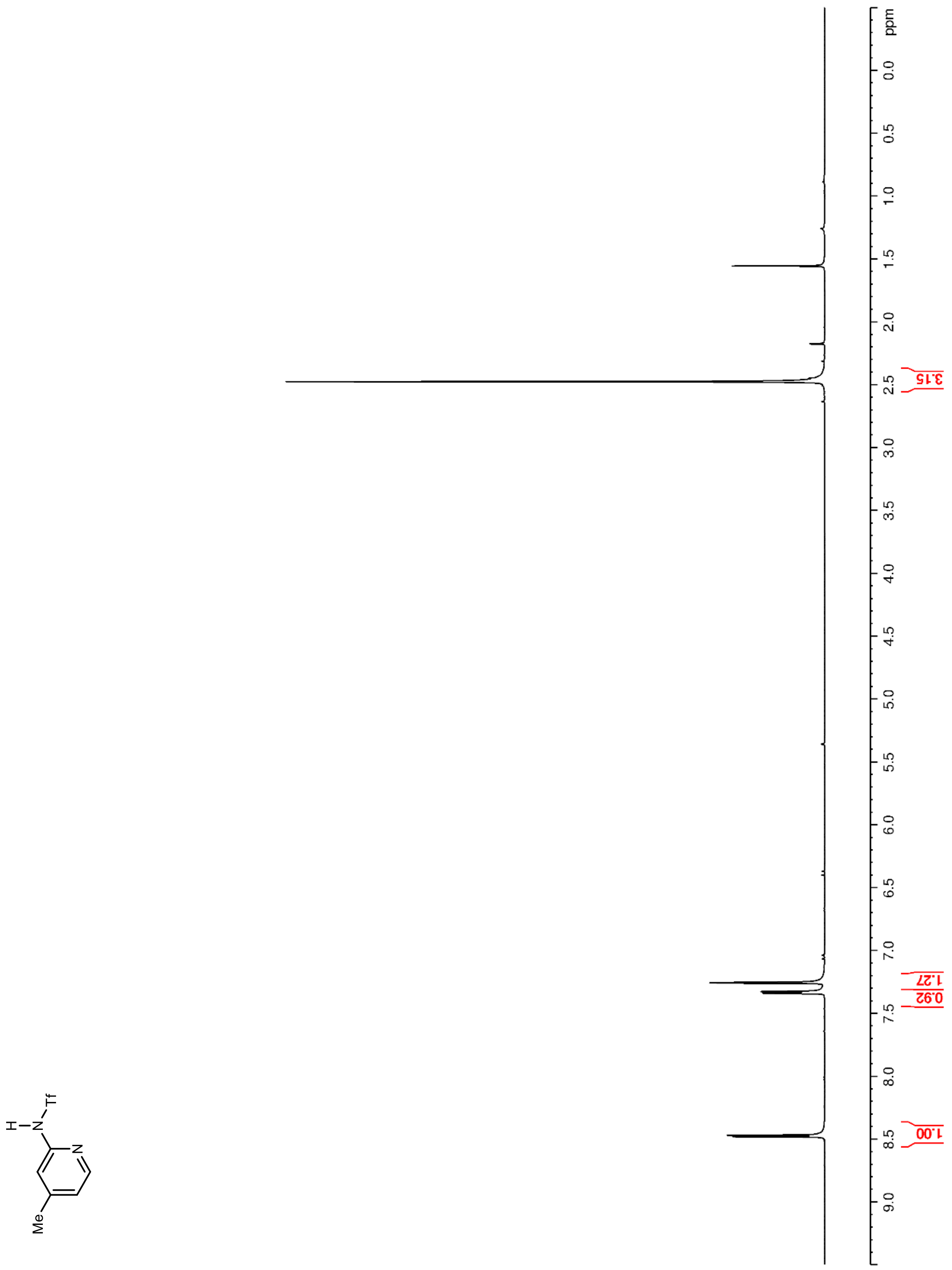

Figure 122. ^{13}C NMR (150 MHz, acetone-d_6) of **H5**

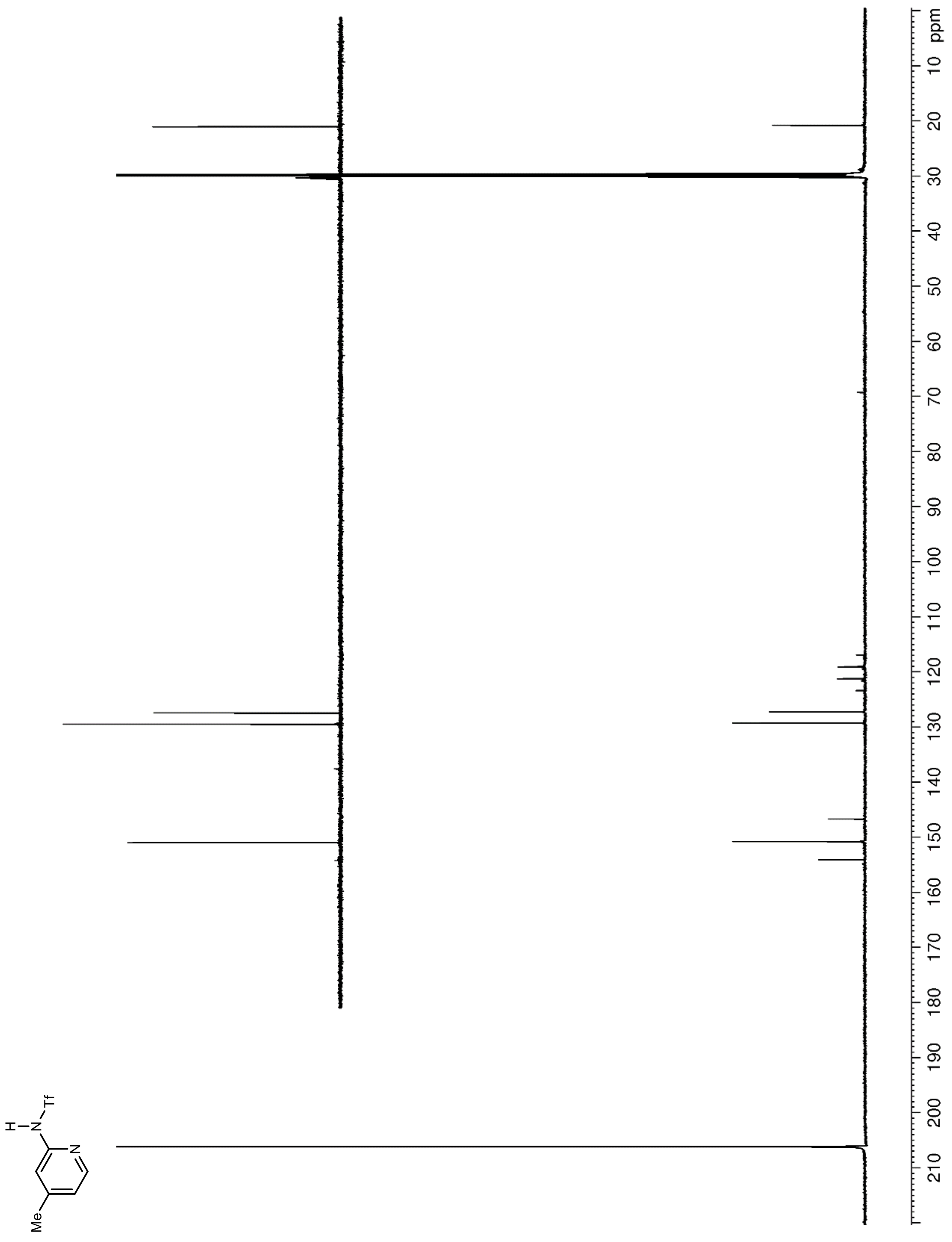

Figure 123. ^{19}F NMR (282 MHz, acetone-d_6) of **H5**

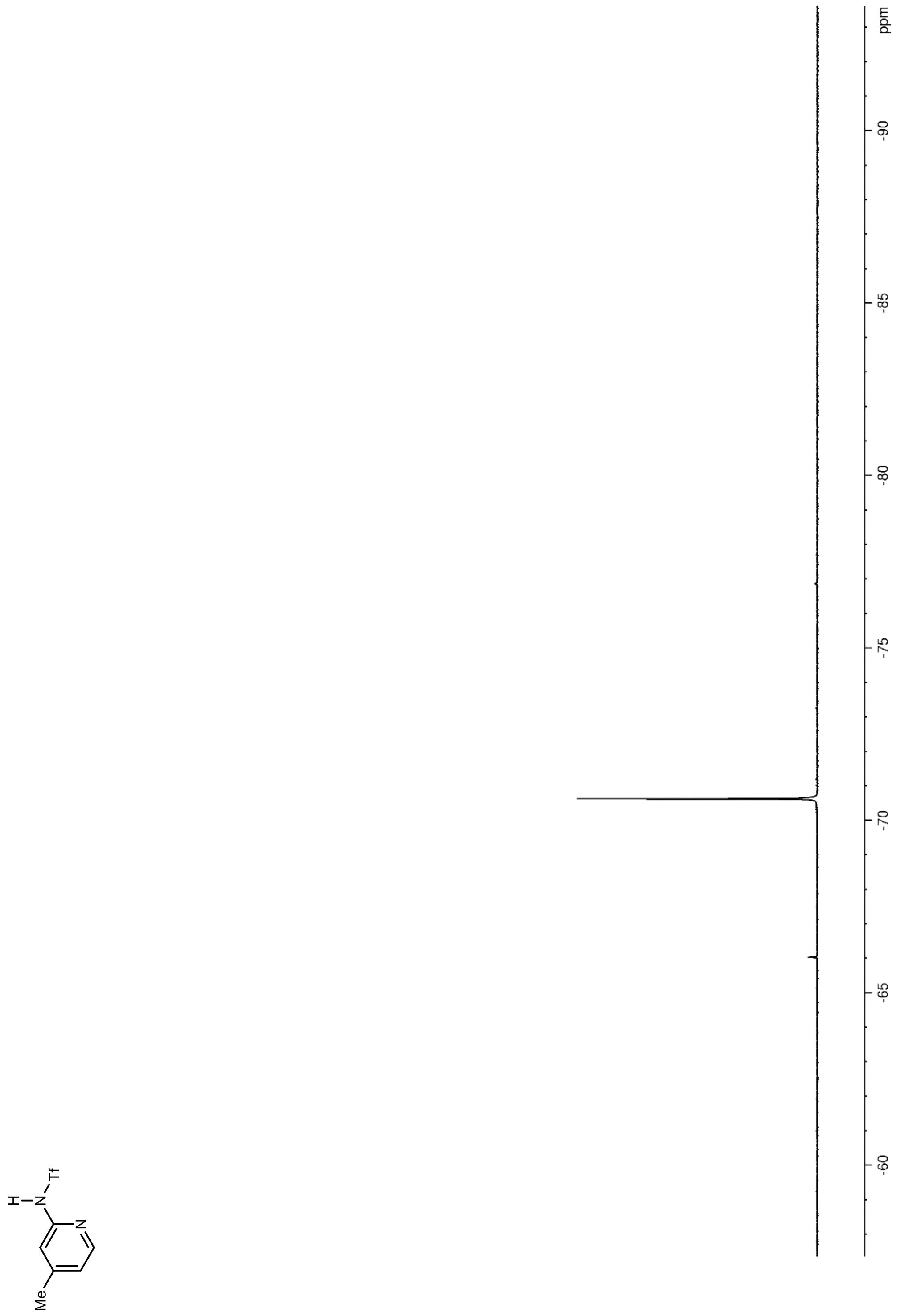

Figure 124. ^{1}H NMR (400 MHz, $CDCl_3$) of **H6**

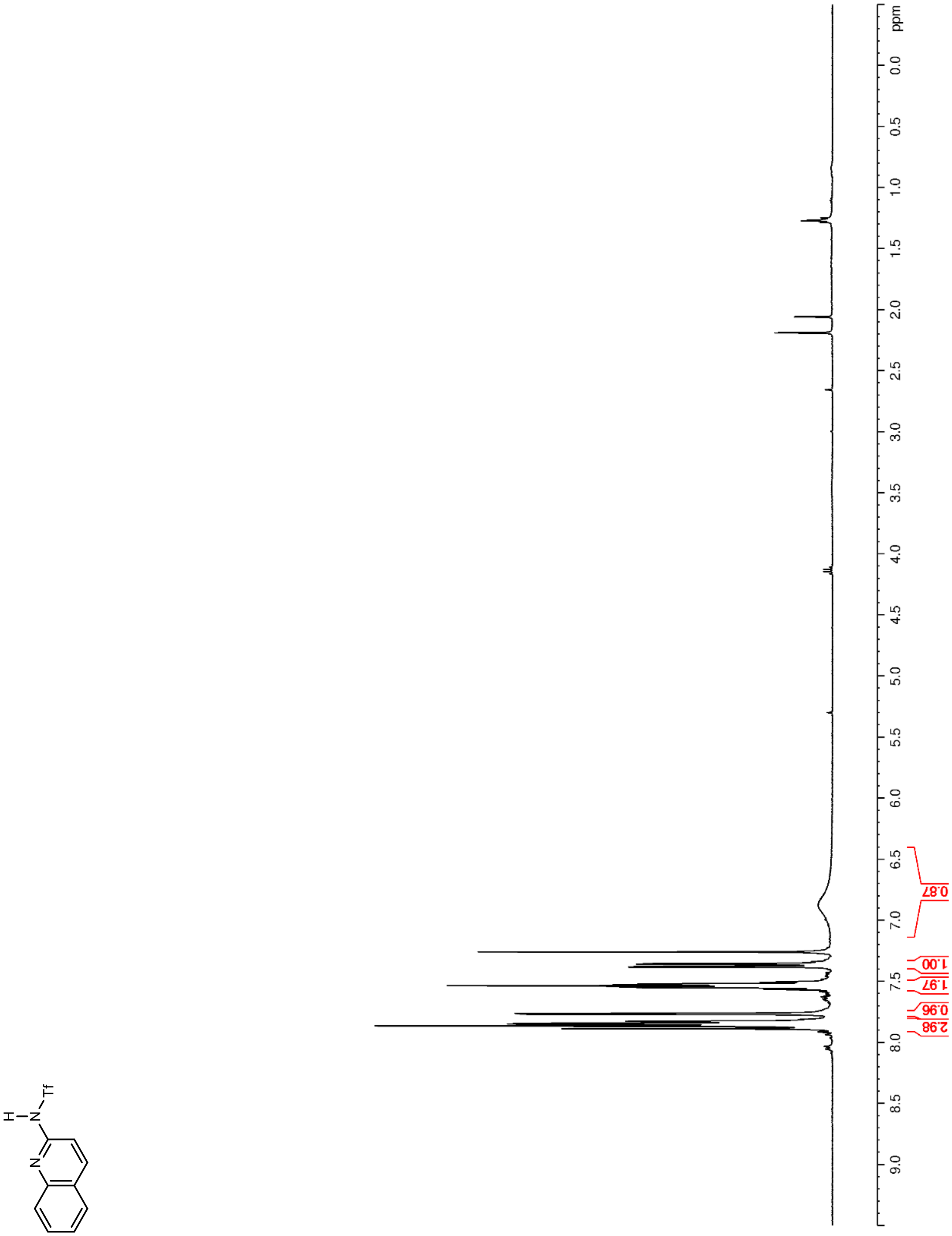

Figure 125. ^{13}C NMR (150 MHz, acetone-d_6) of **H6**

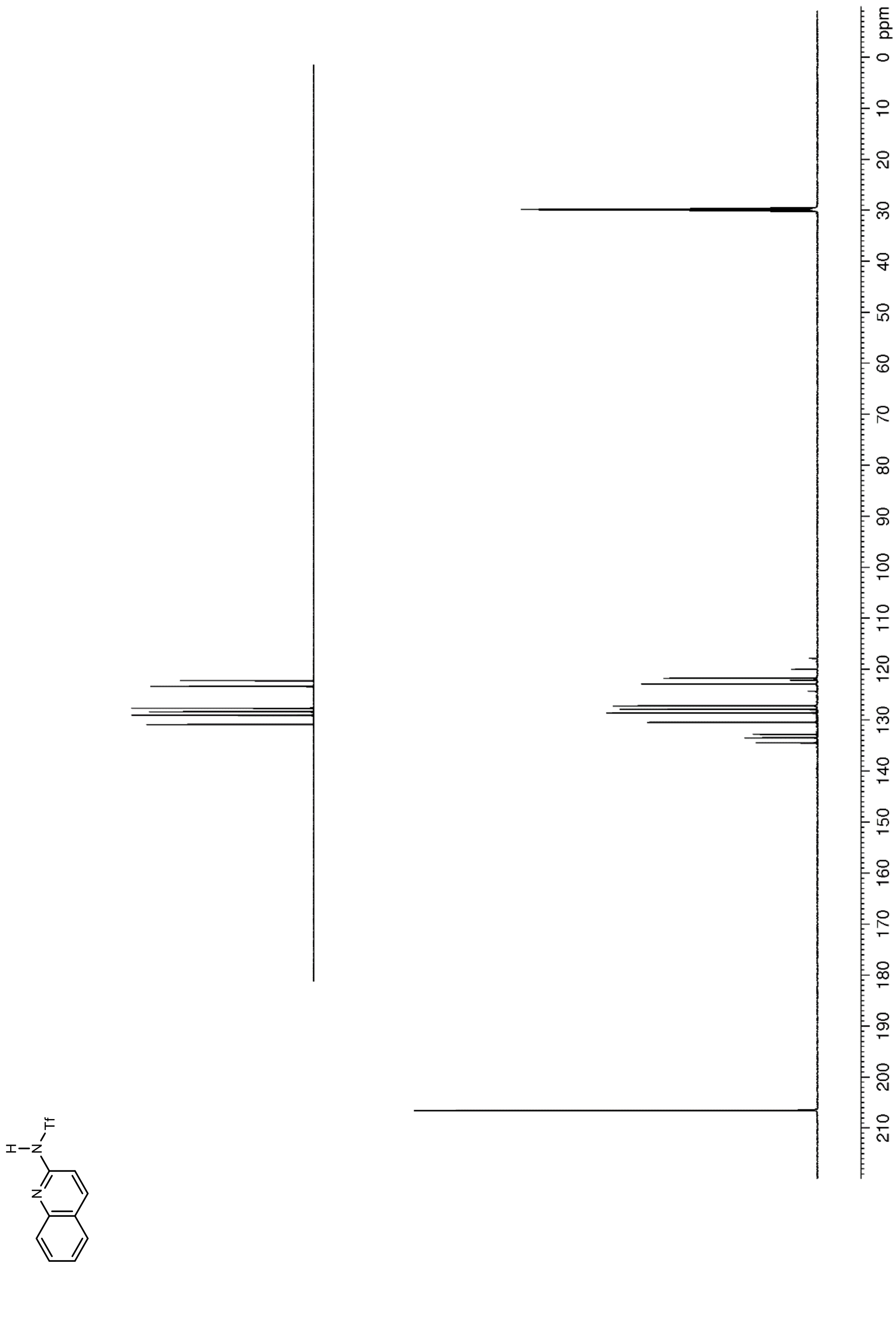

Figure 126. ^{19}F NMR (282 MHz, acetone-d_6) of **H6**

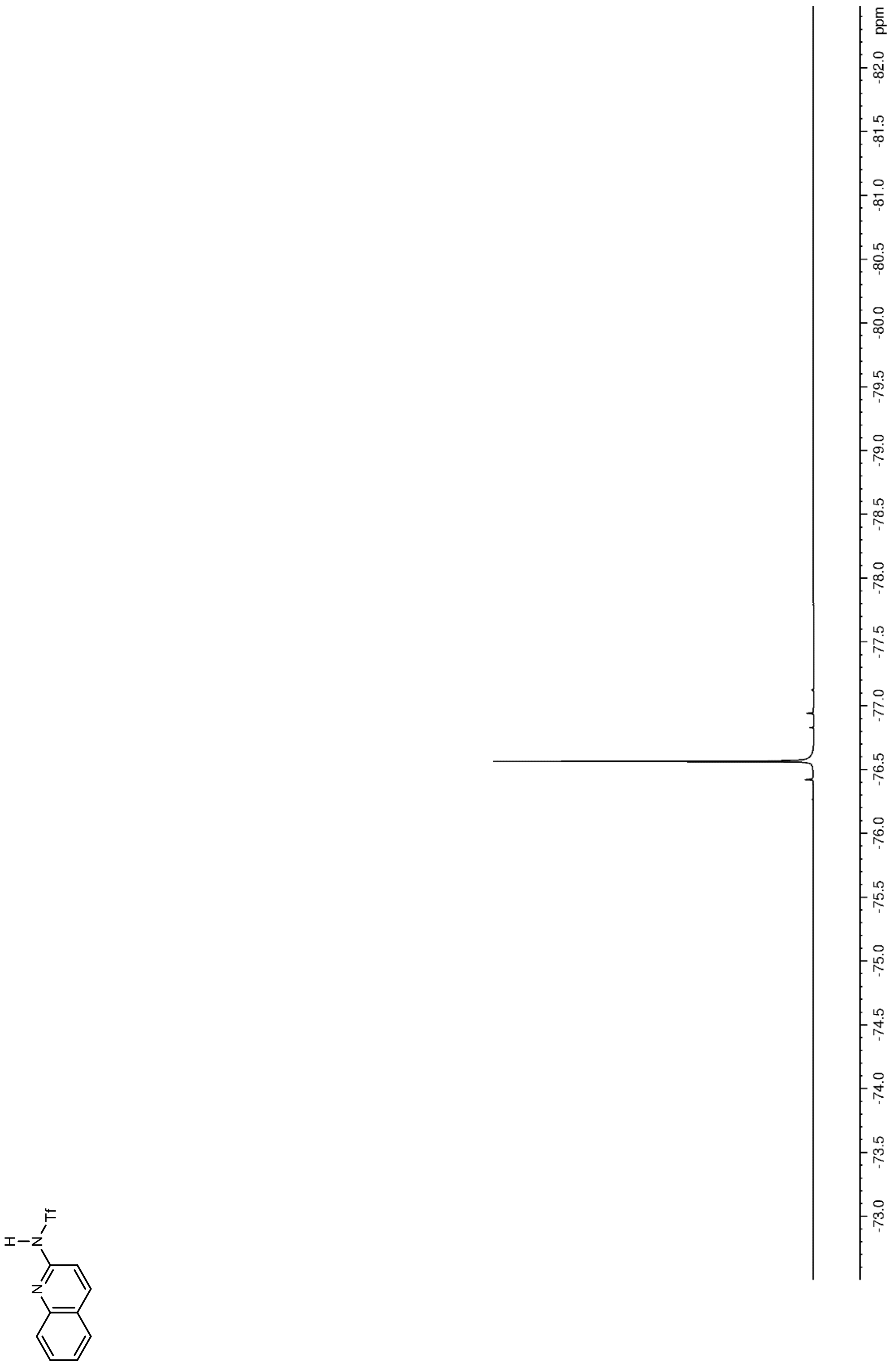

Figure 127. ^{1}H NMR (400 MHz, acetone-d_6) of **H7**

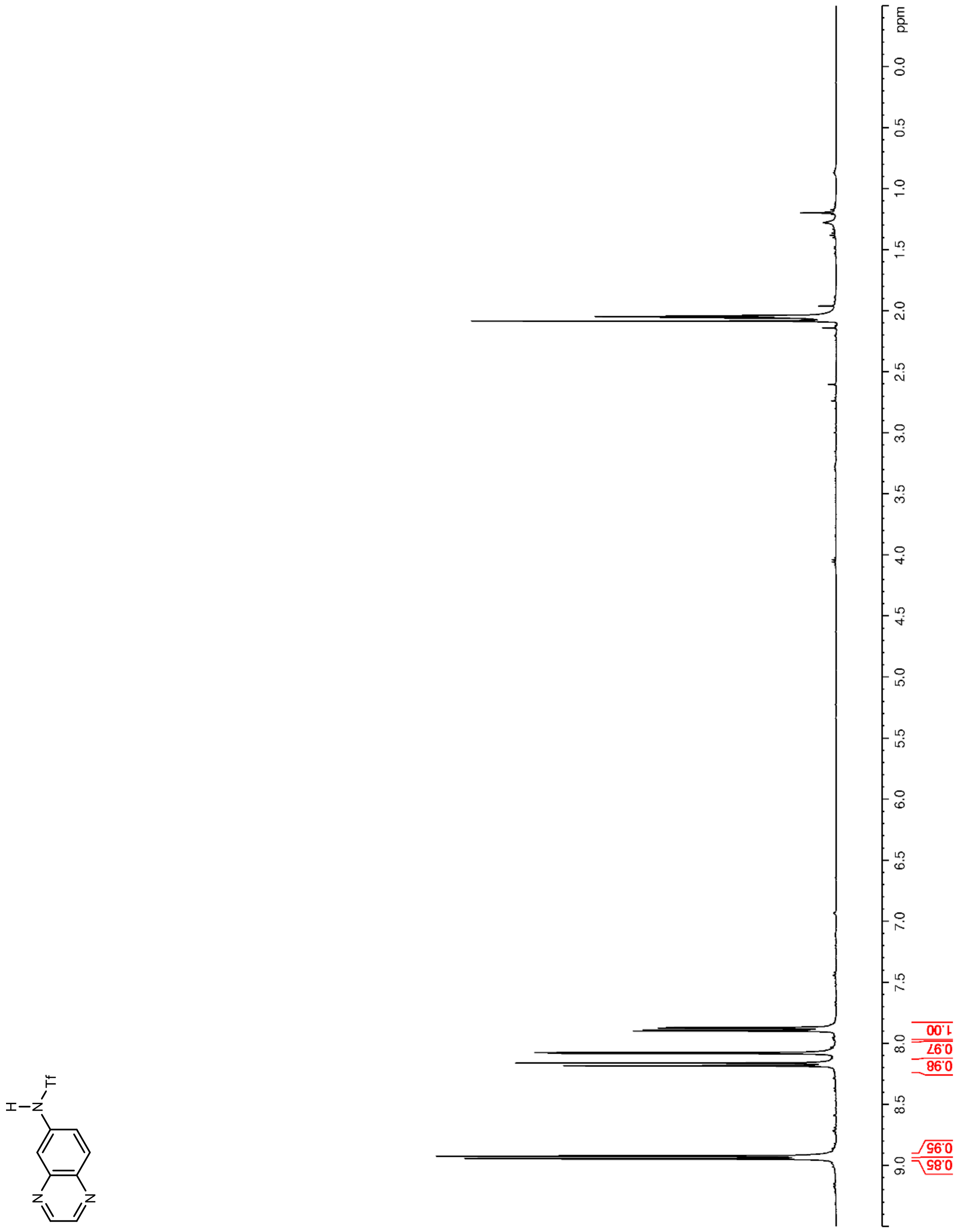

Figure 128. ^{13}C NMR (150 MHz, acetone-d_6) of **H7**

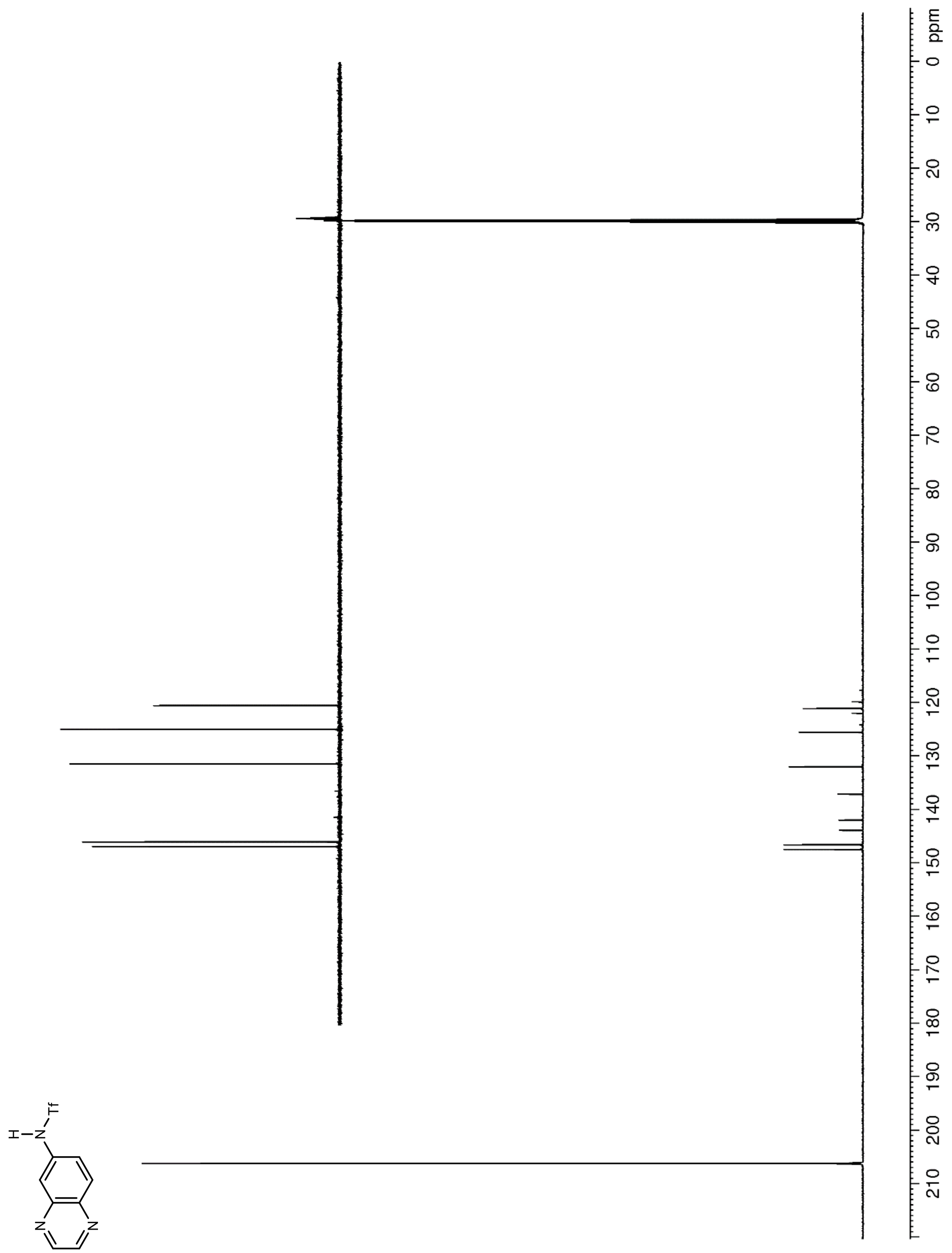

Figure 129. ^{19}F NMR (282 MHz, acetone-d_6) of **H7**

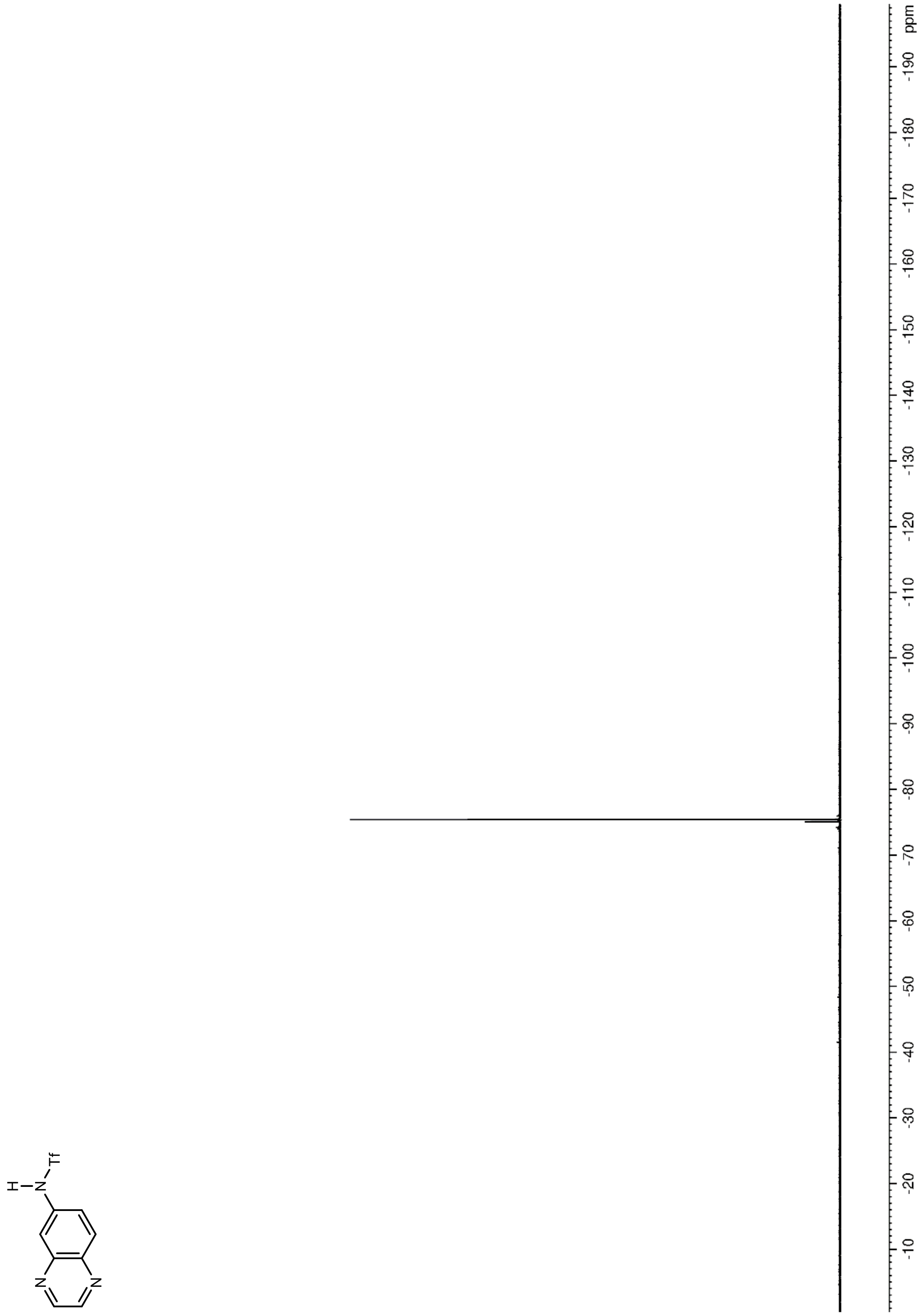

Figure 130. ^{1}H NMR (400 MHz, acetone-d_6) of **H8**

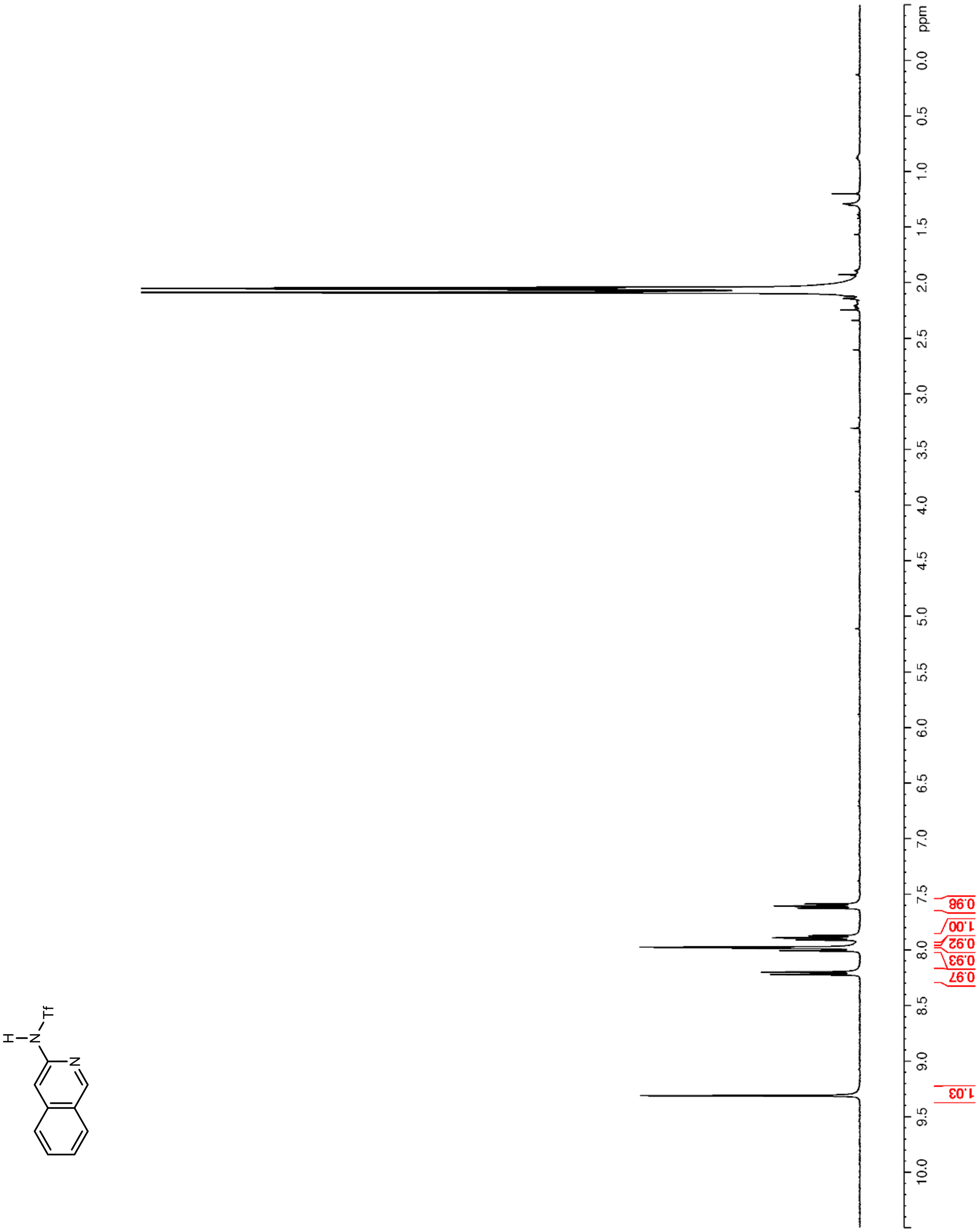

Figure 131. ^{13}C NMR (150 MHz, acetone-d_6) of **H8**

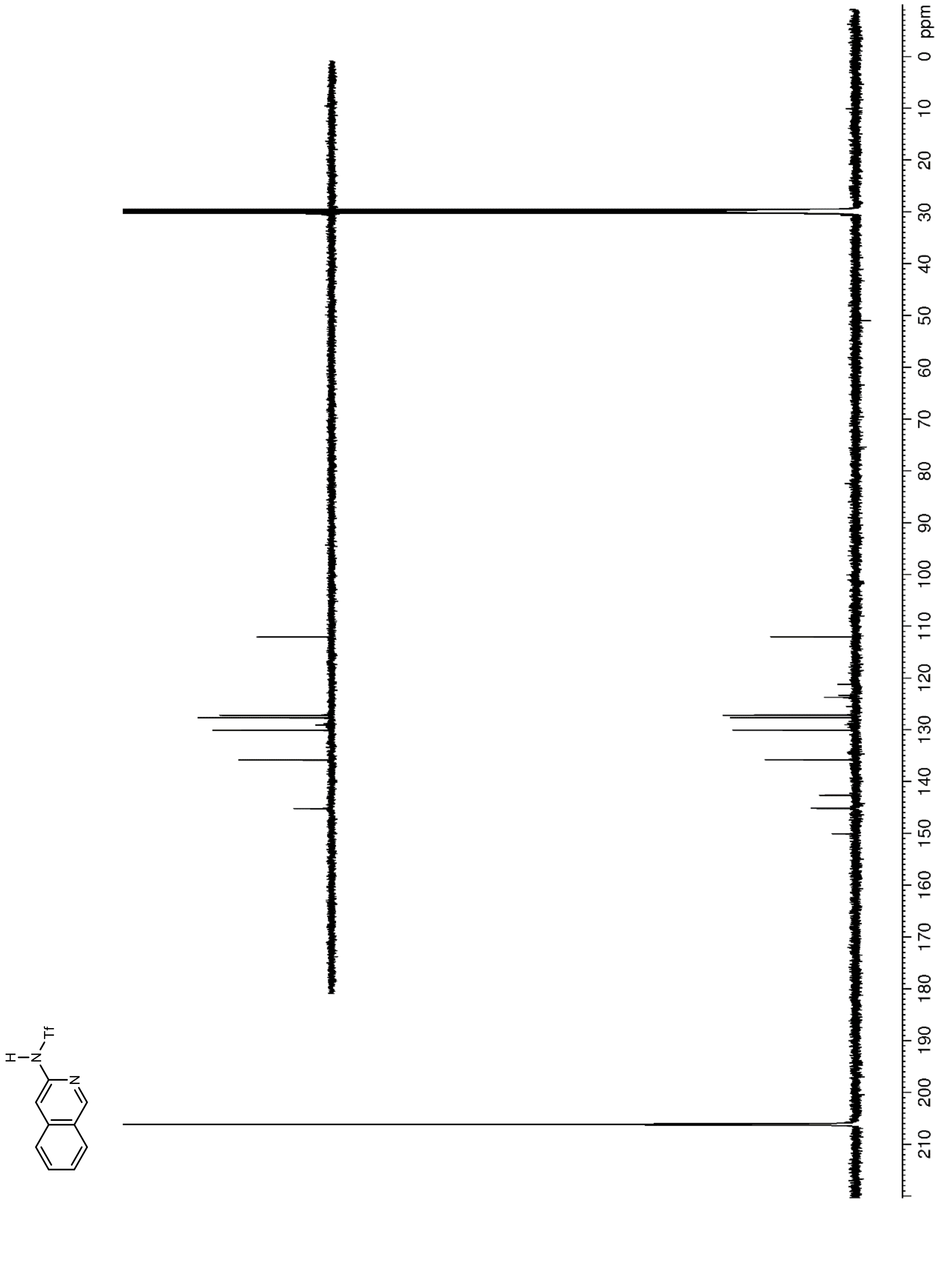

Figure 132. ^{19}F NMR (282 MHz, acetone-d_6) of **H8**

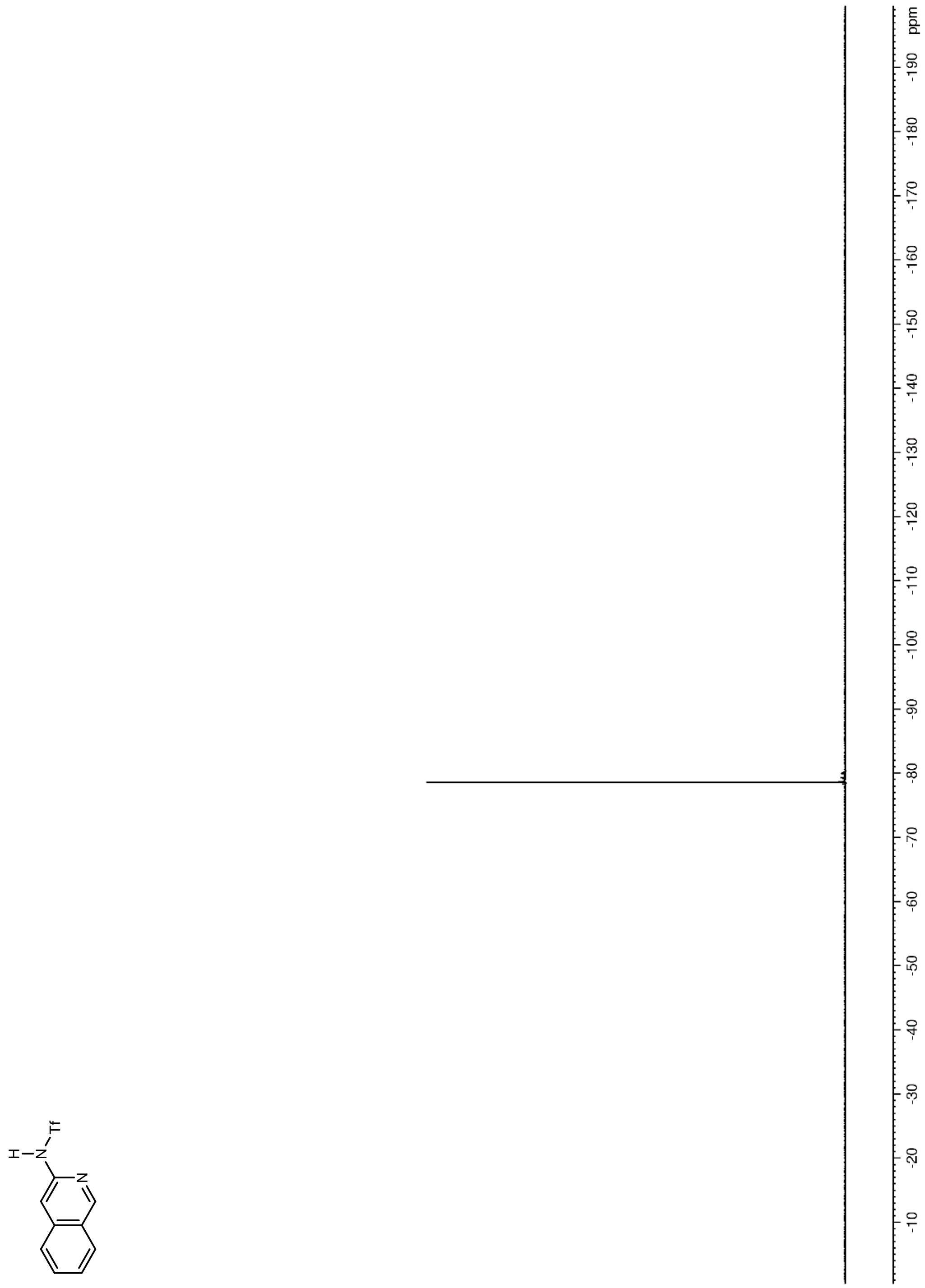

Figure 133. ^{1}H NMR (400 MHz, acetone-d_6) of **I3**

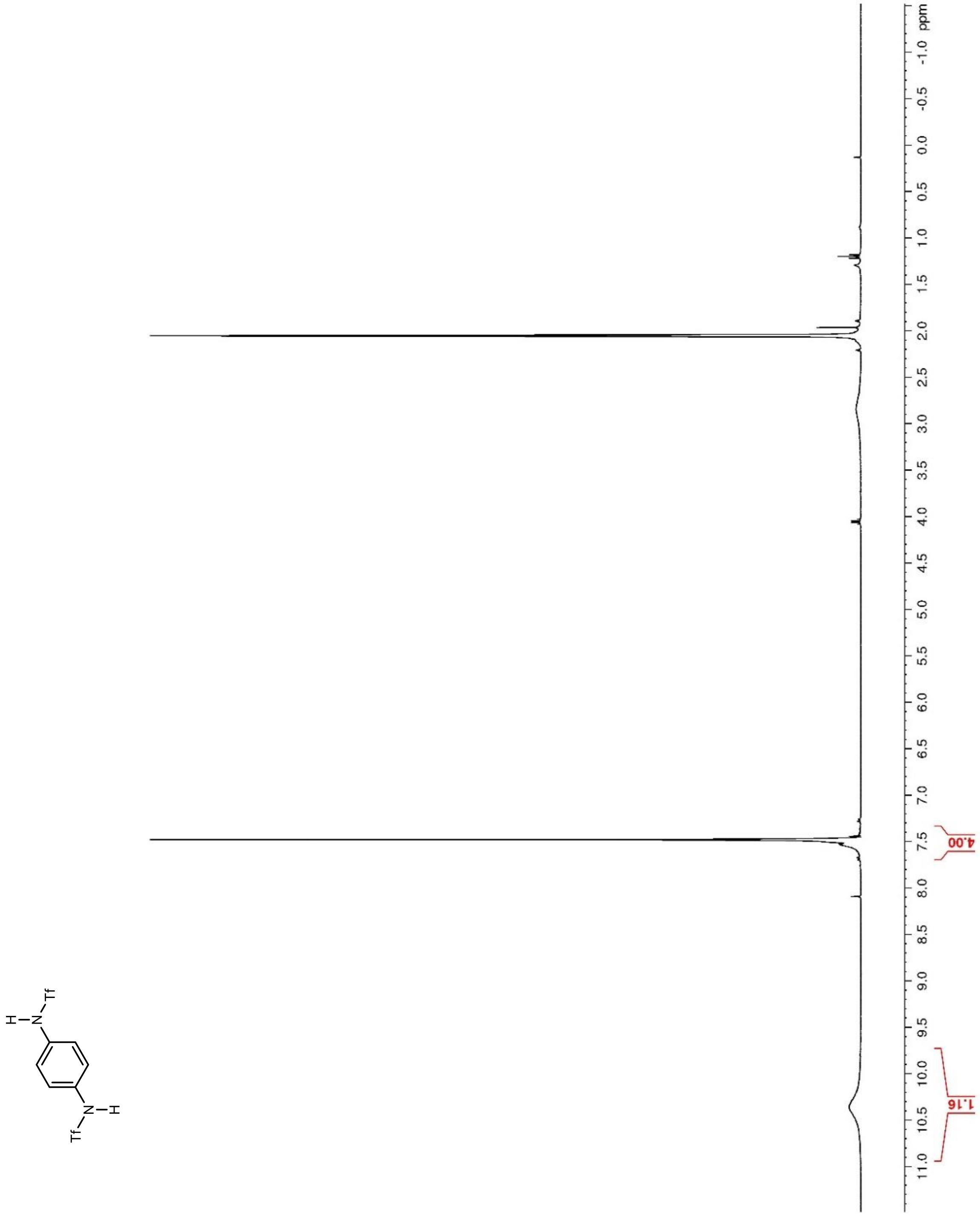

Figure 134. ^{13}C NMR (150 MHz, acetone-d_6) of **I3**

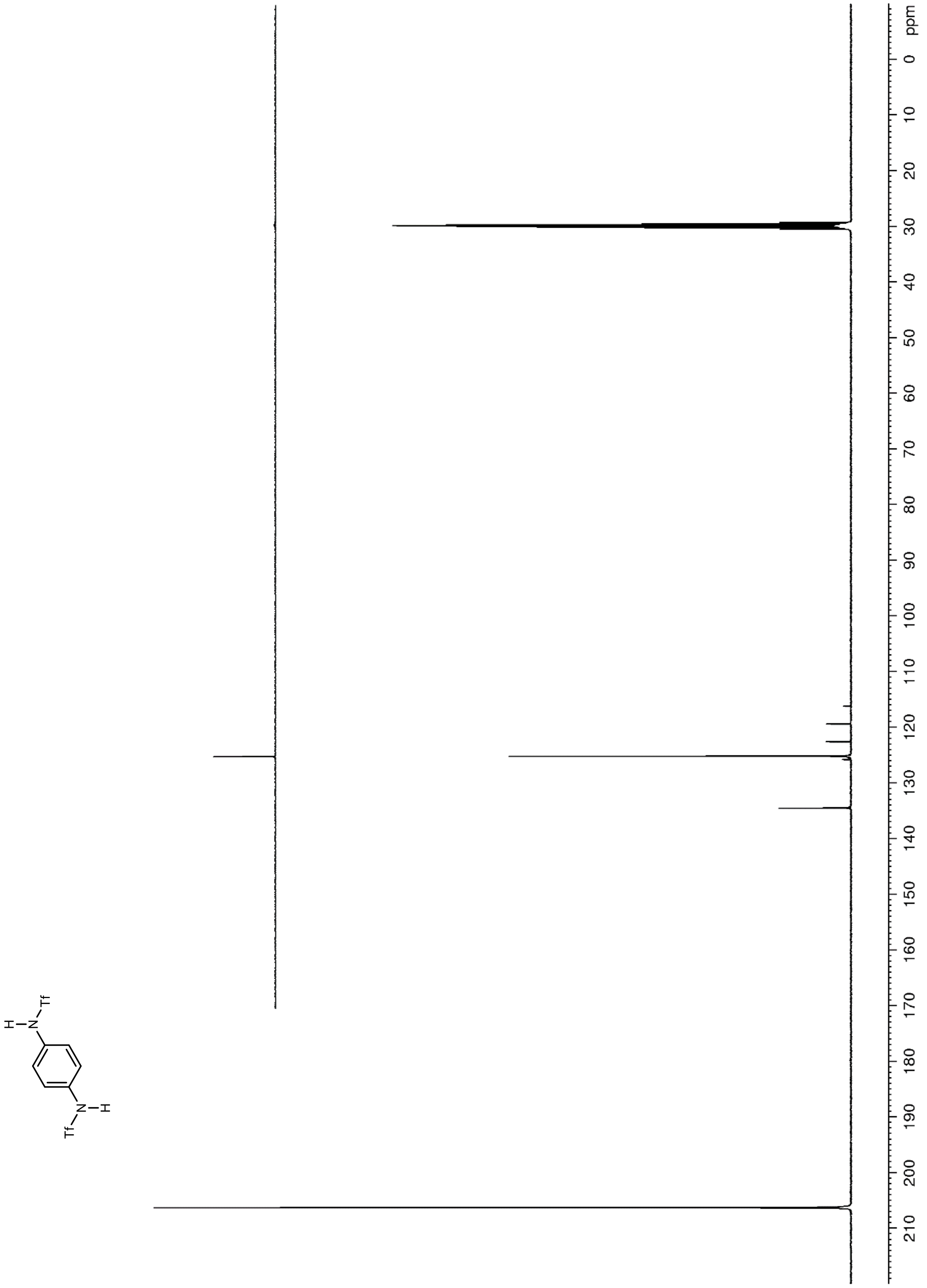

Figure 135. ^{19}F NMR (282 MHz, acetone-d_6) of **I3**

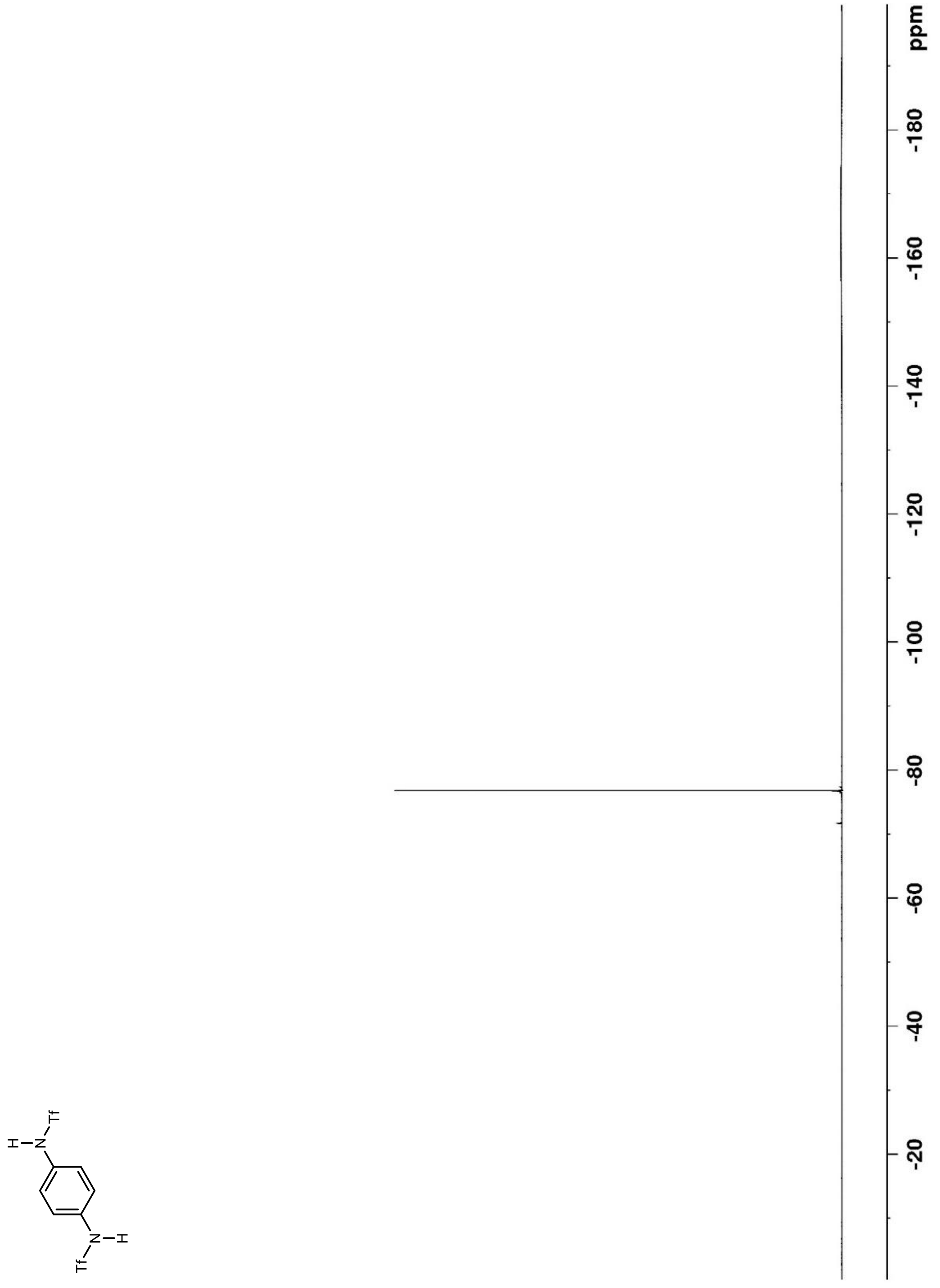

Figure 136. ^{1}H NMR (400 MHz, acetone-d_6) of **I4**

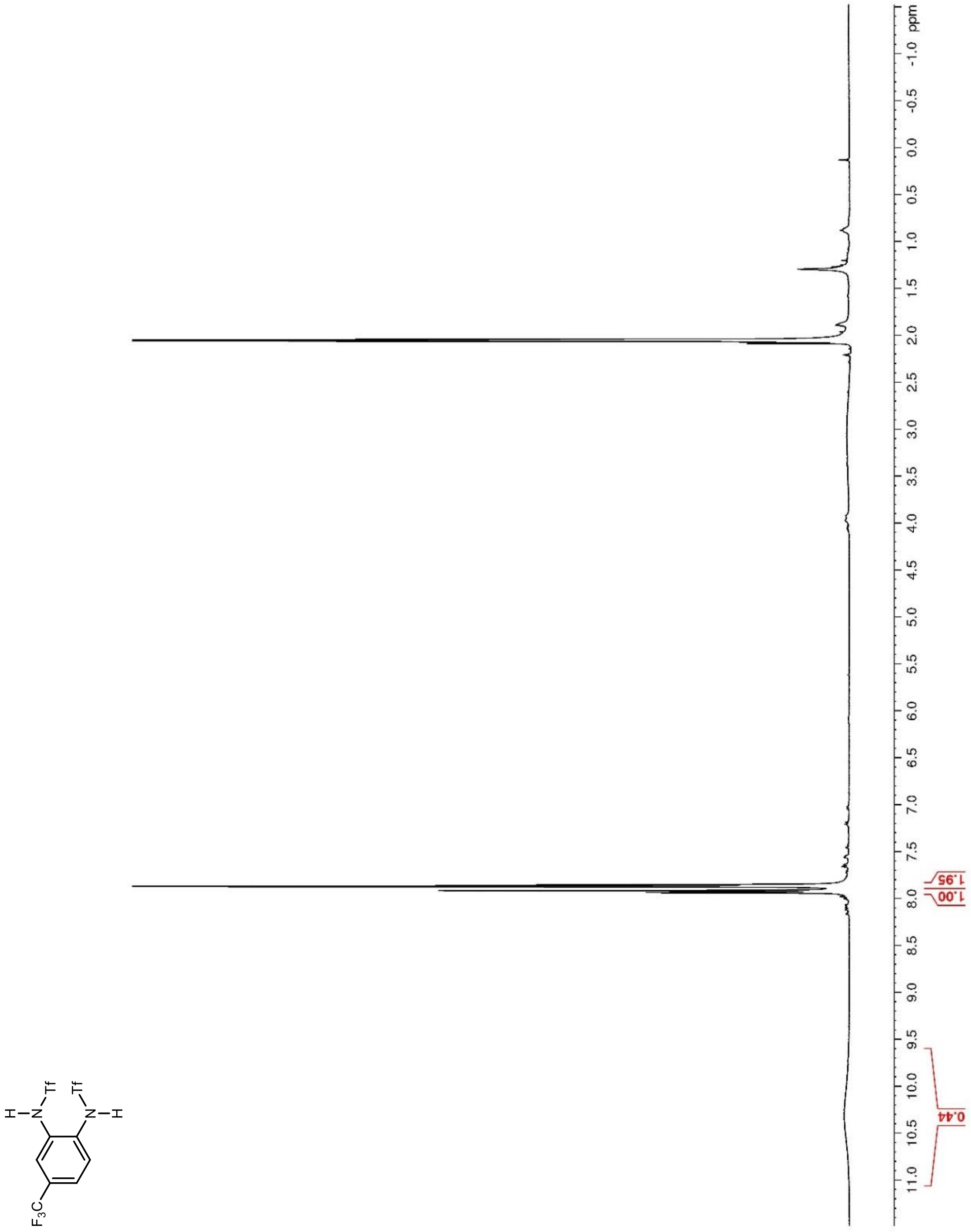

Figure 137. ^{13}C NMR (150 MHz, acetone-d_6) of **I4**

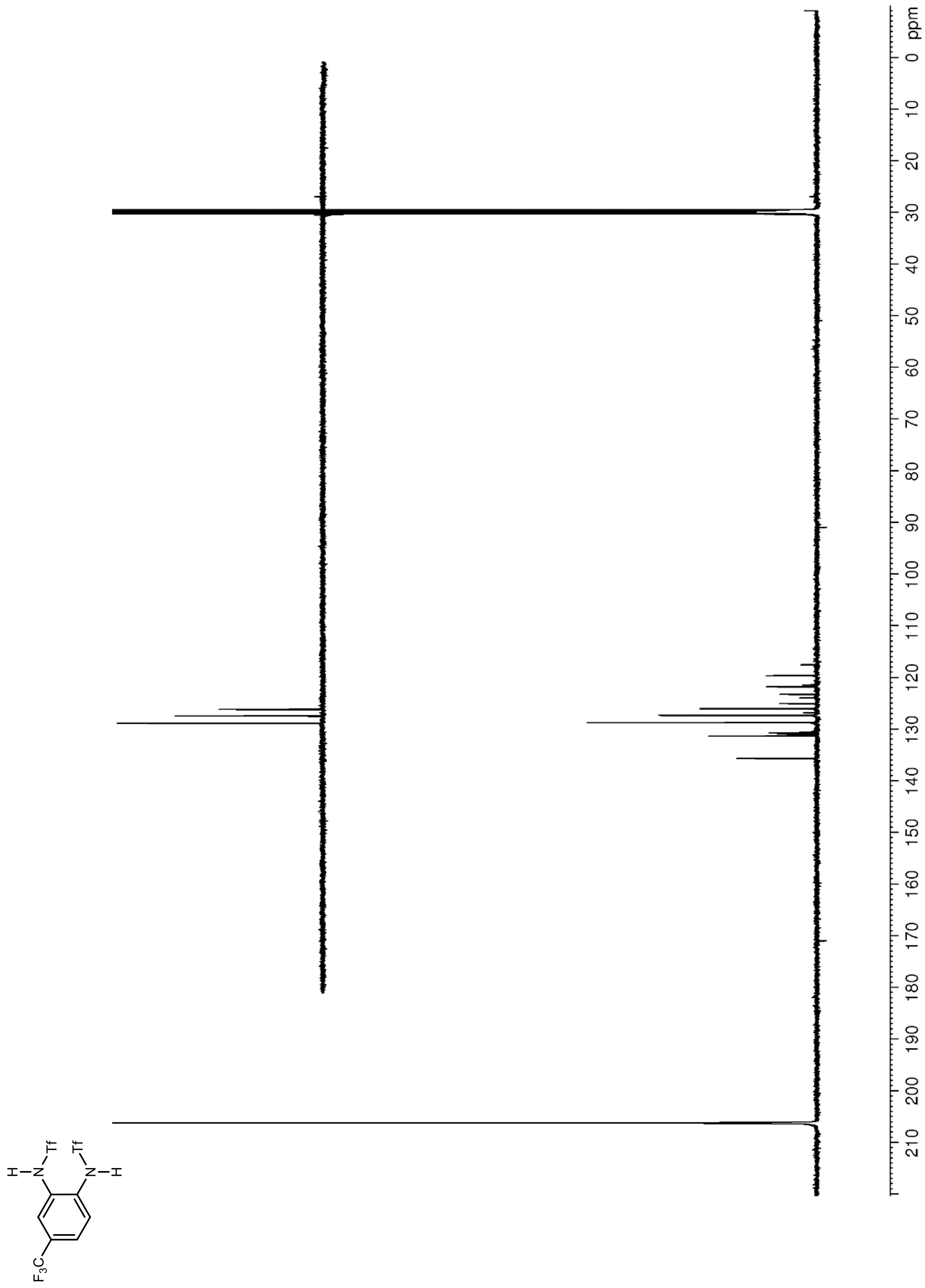

Figure 138. ^{19}F NMR (282 MHz, acetone-d_6) of **I4**

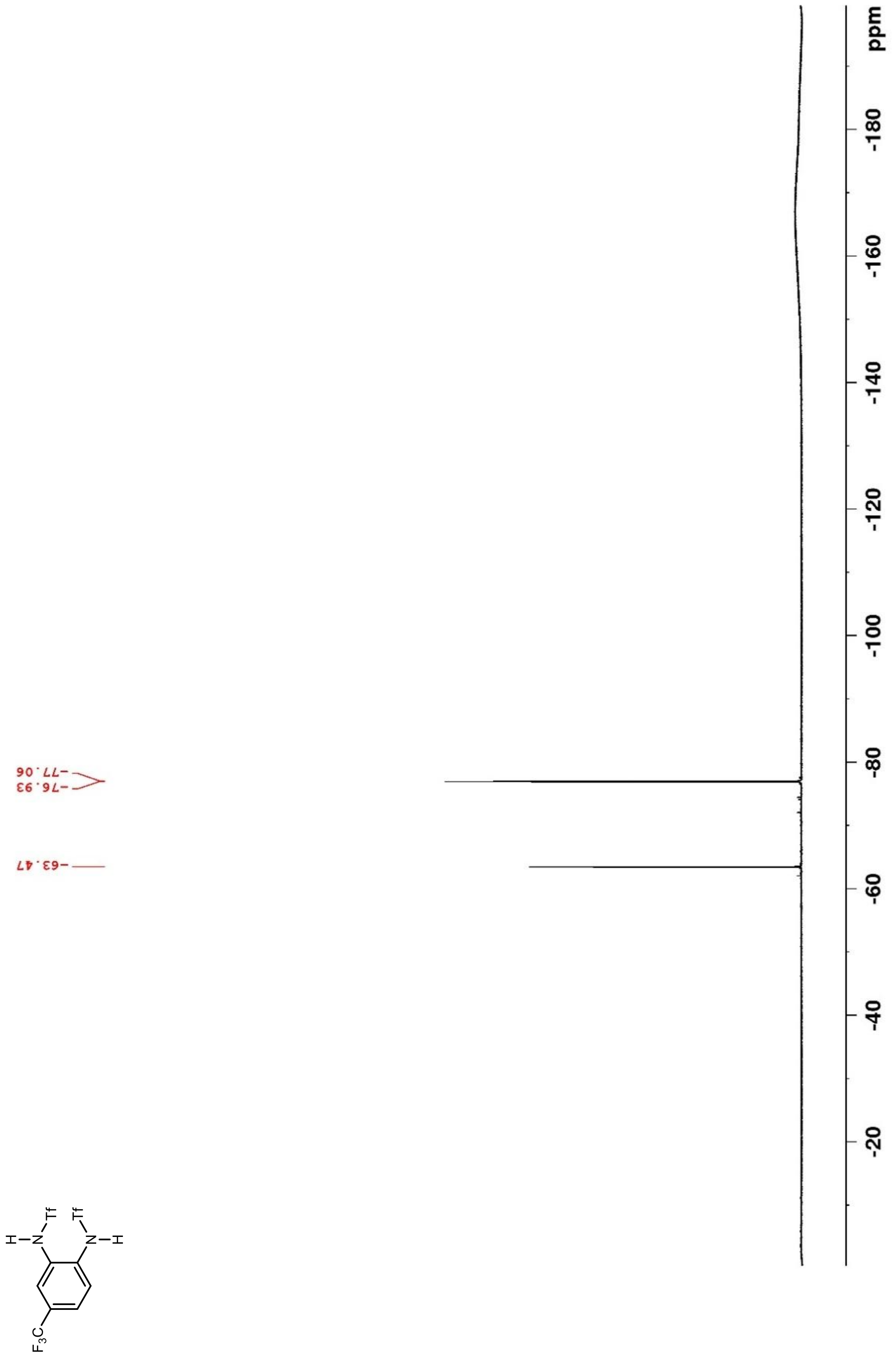

Figure 139. ^{1}H NMR (400 MHz, acetone-d_6) of **I6**

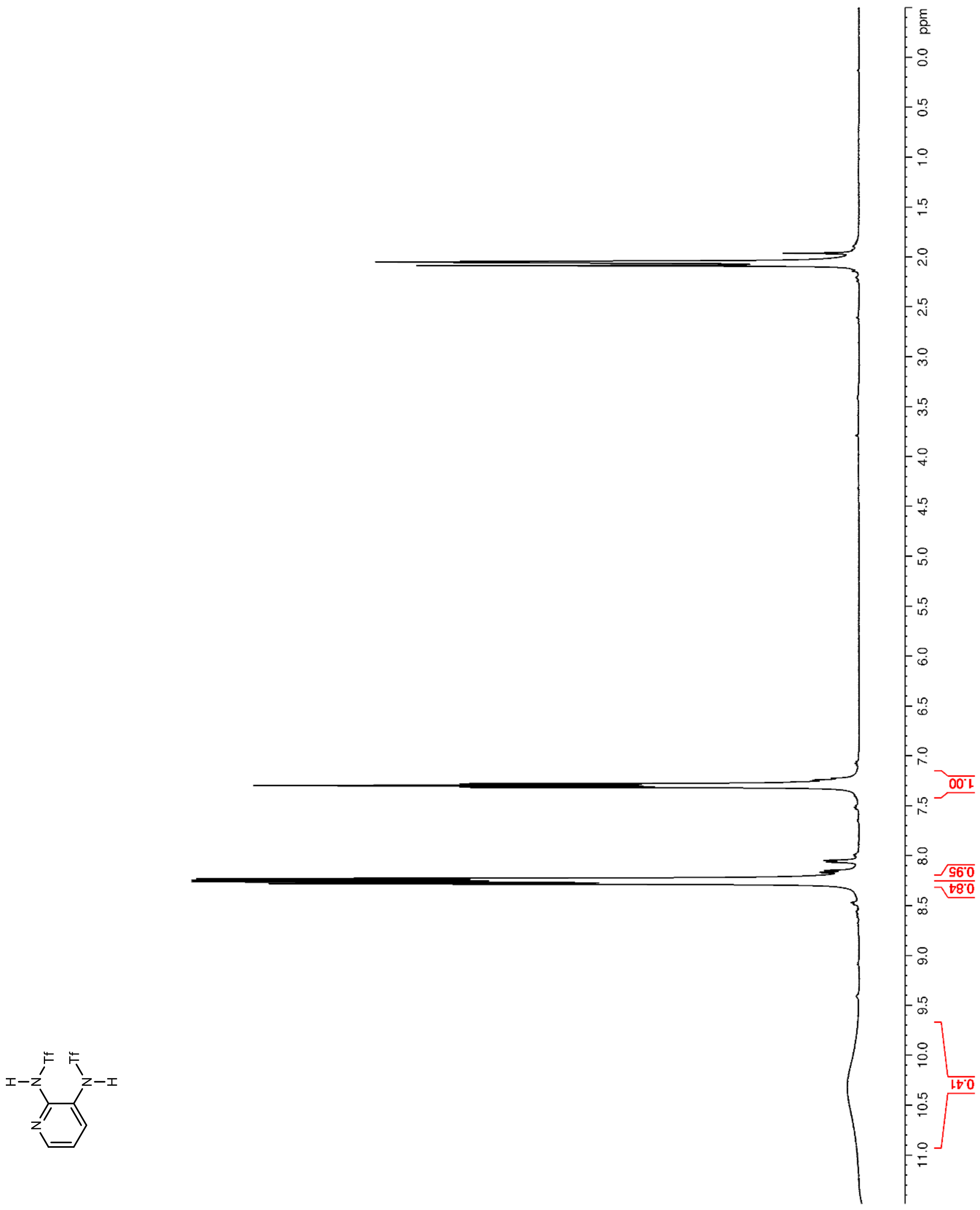

Figure 140. ^{13}C NMR (150 MHz, acetone-d_6) of **I6**

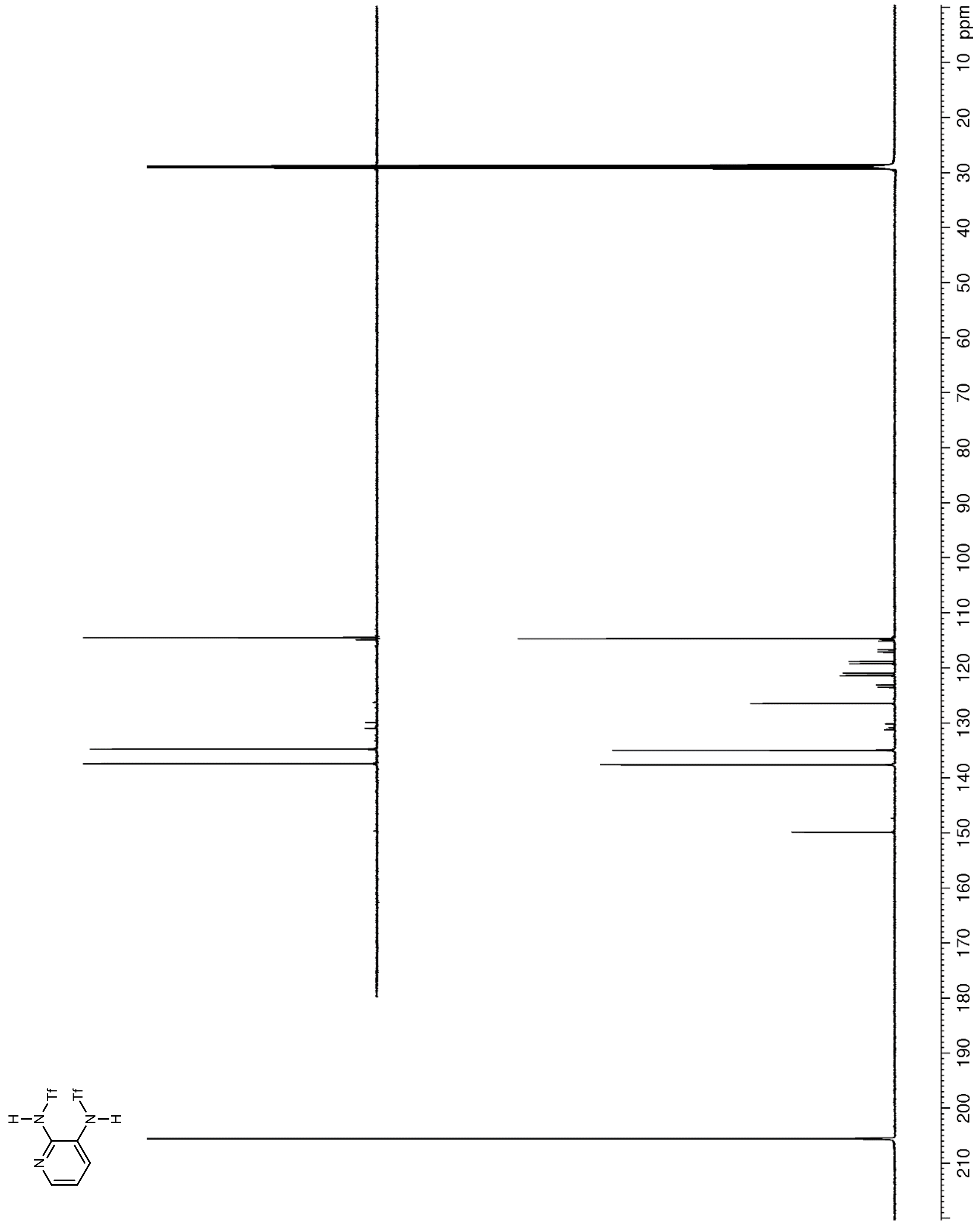

Figure 141. ^{19}F NMR (282 MHz, acetone-d_6) of **I6**

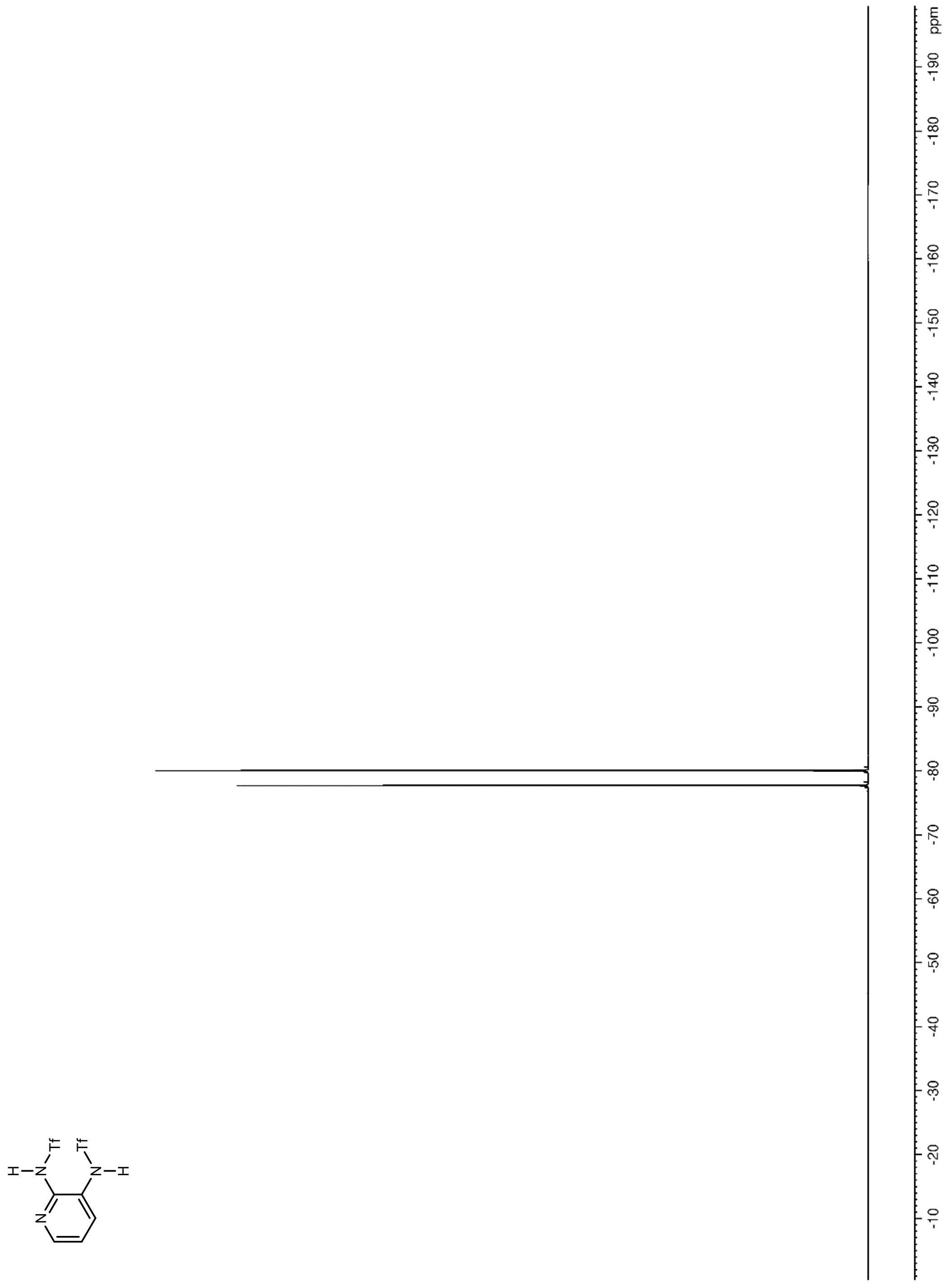

Figure 142. ^{1}H NMR (400 MHz, acetone-d_6) of **I8**

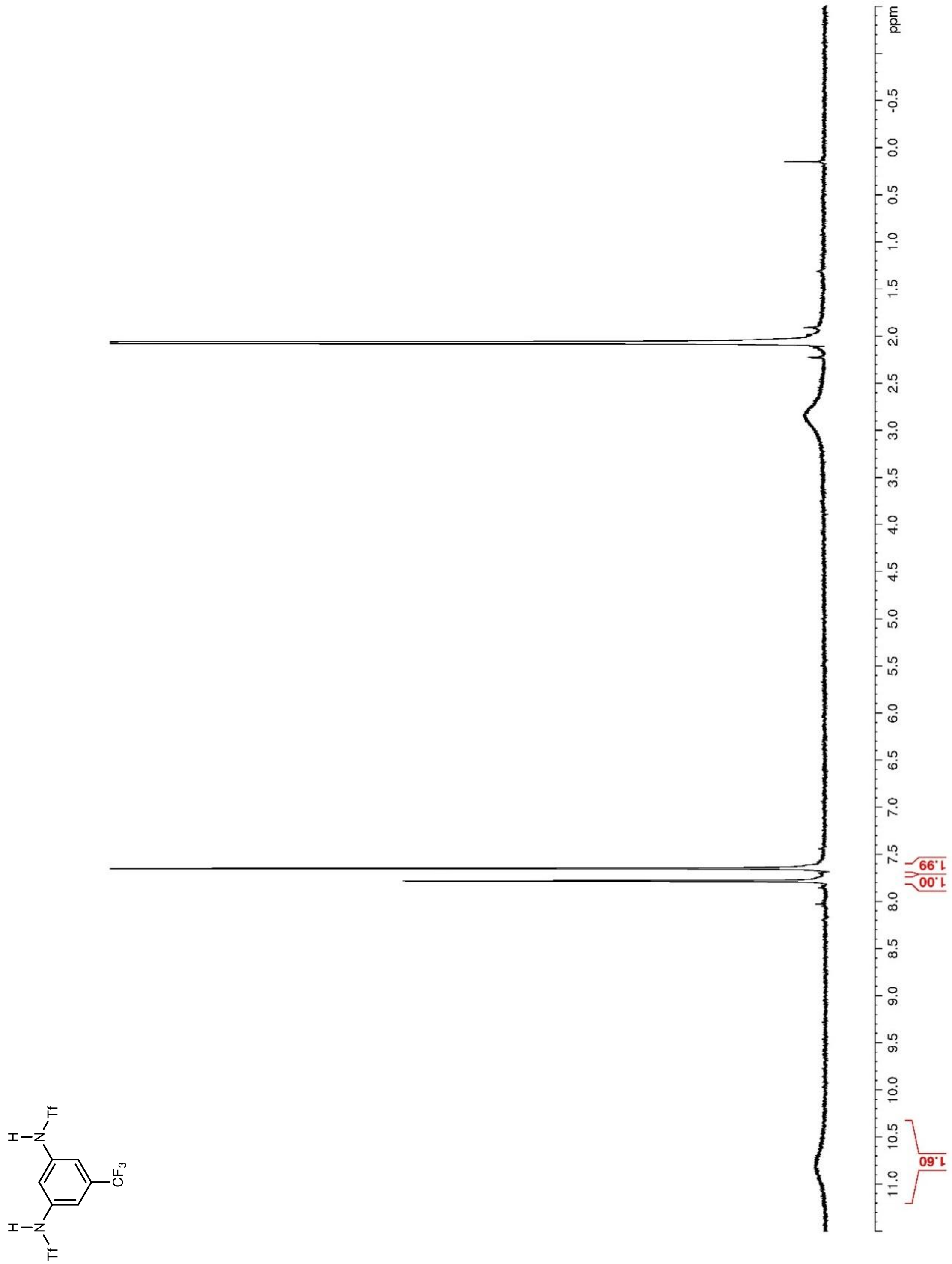

Figure 143. ^{13}C NMR (150 MHz, acetone-d_6) of **I8**

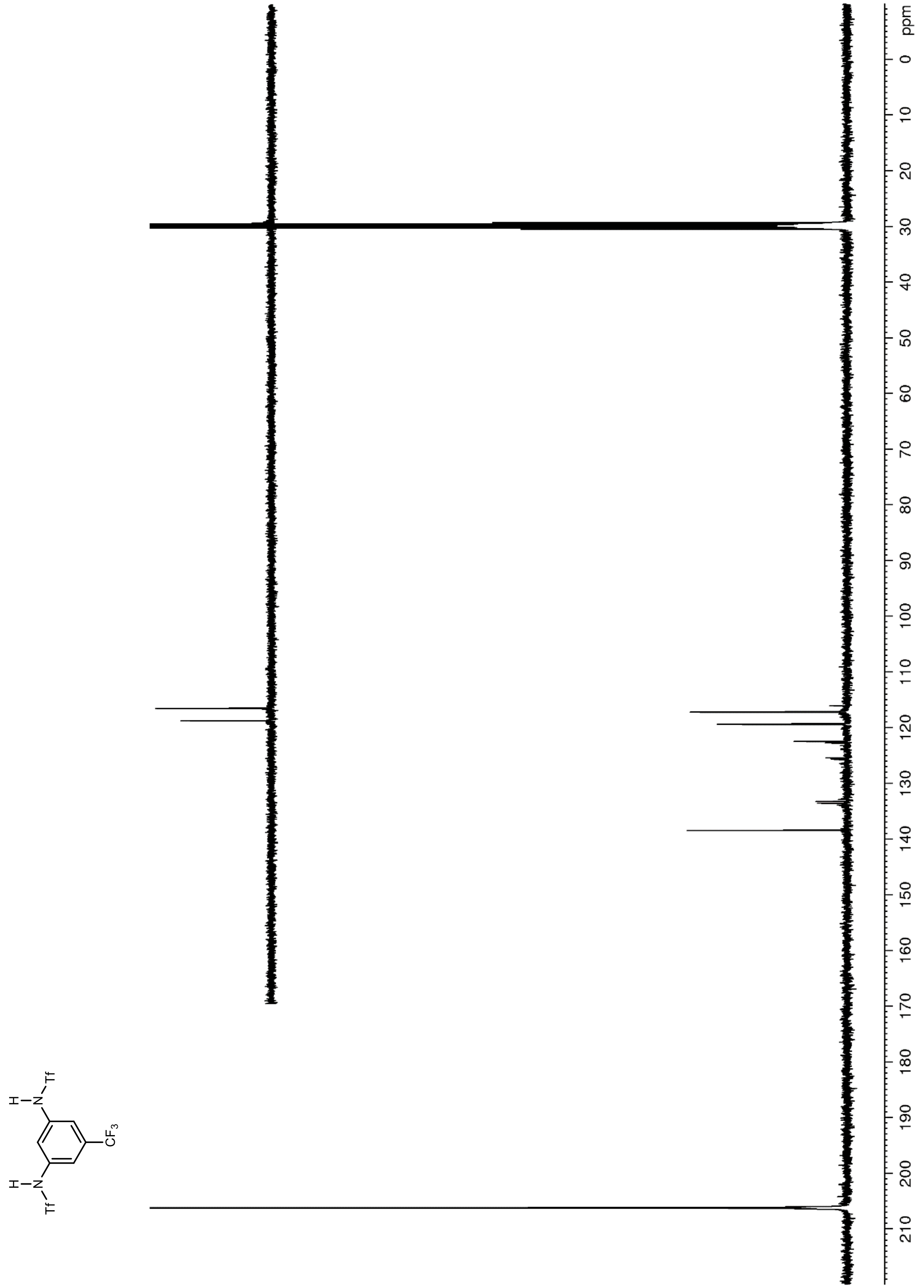

Figure 144. ^{19}F NMR (282 MHz, acetone-d_6) of **I8**

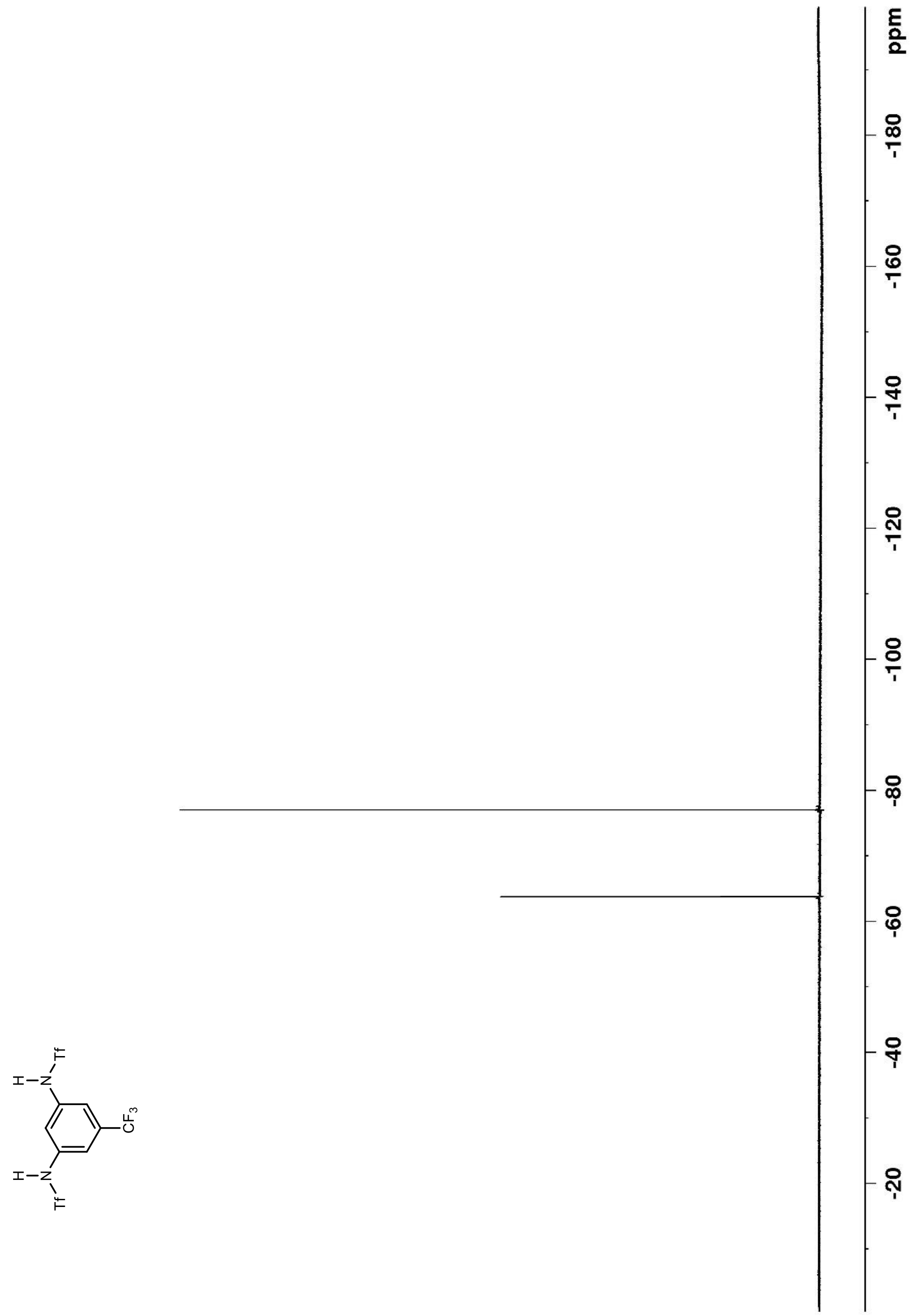

Figure 145. ^{1}H NMR (400 MHz, acetone-d_6) of **J1**

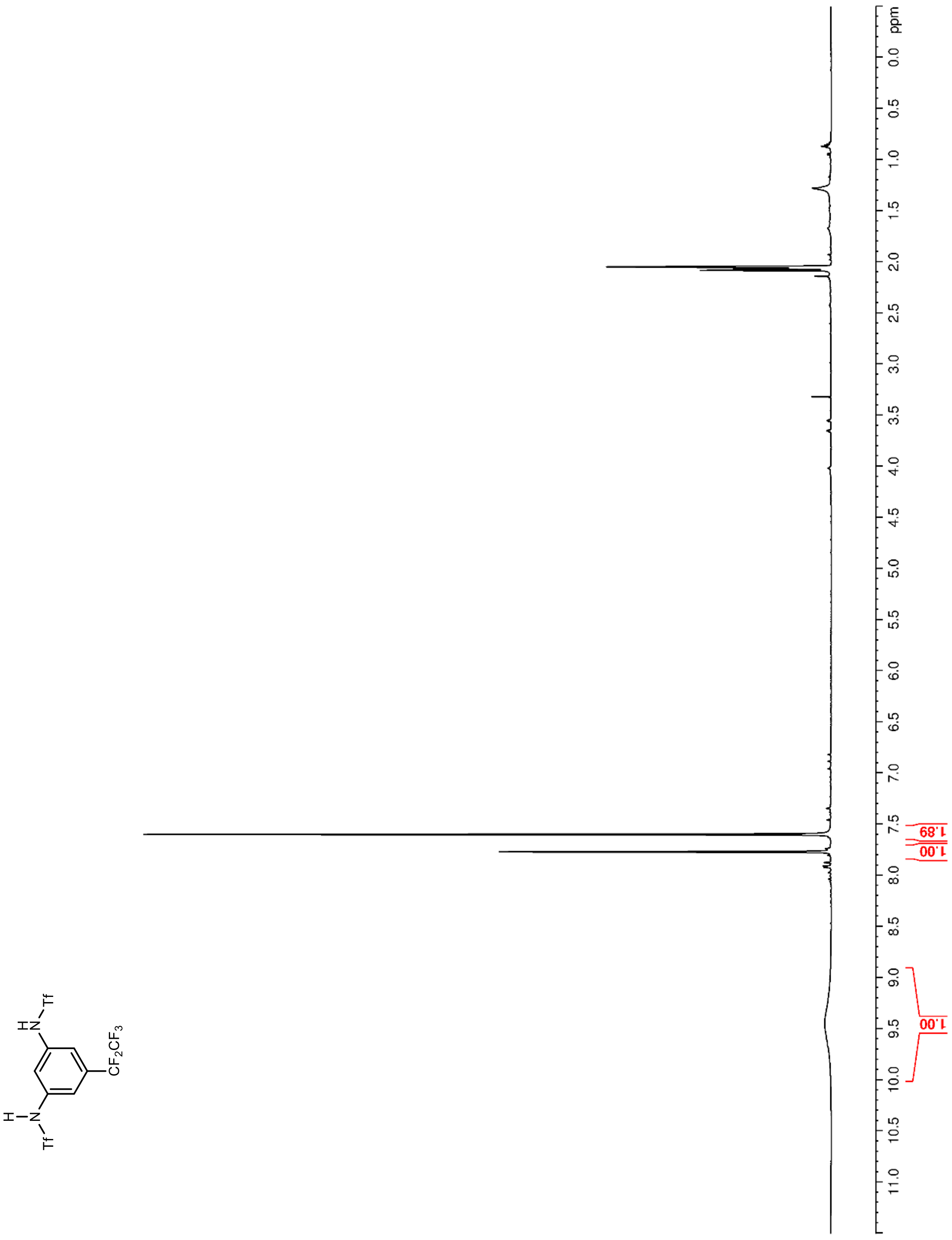

Figure 146. ^{13}C NMR (150 MHz, acetone-d_6) of **J1**

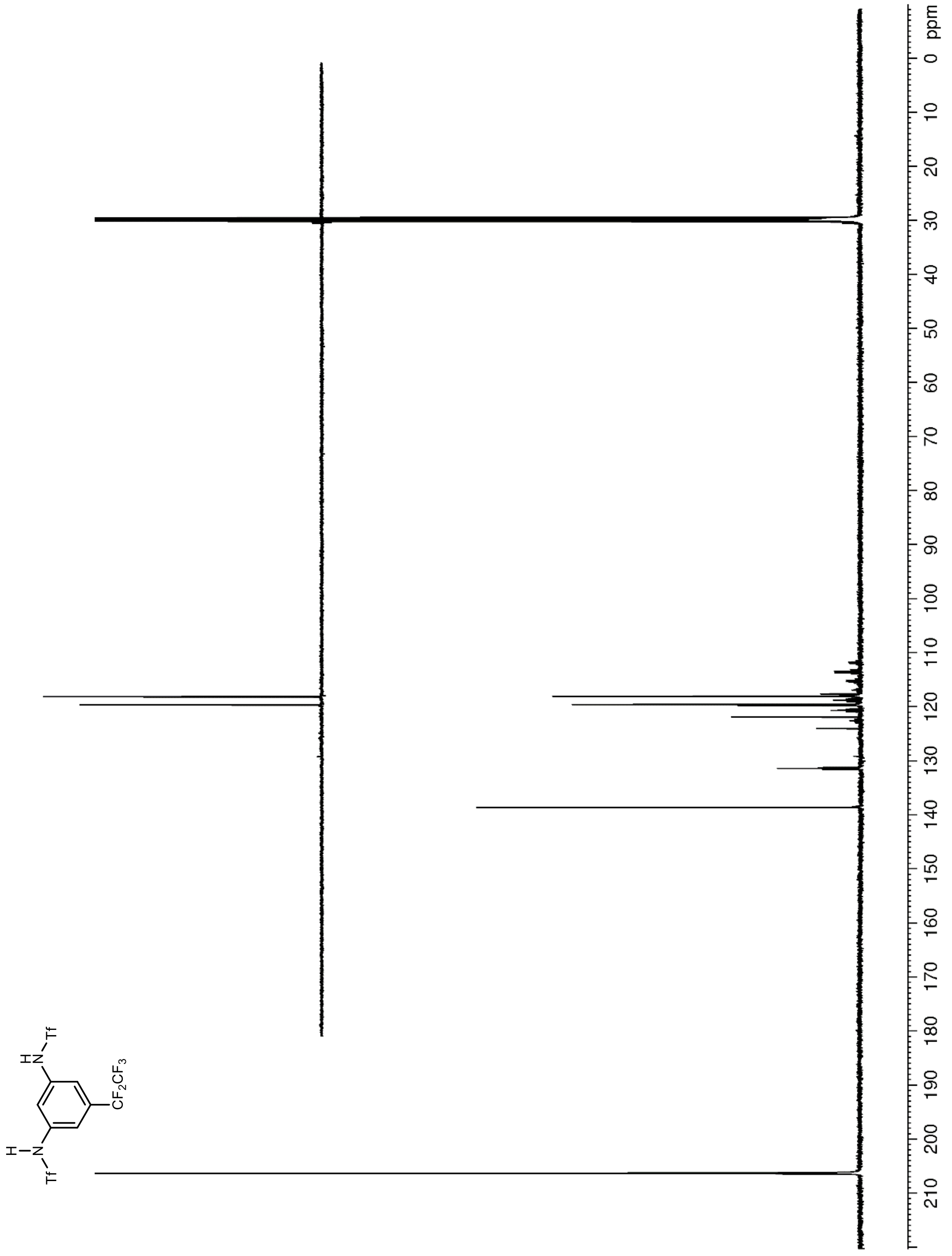

Figure 147. ^{19}F NMR (282 MHz, acetone-d_6) of **J1**

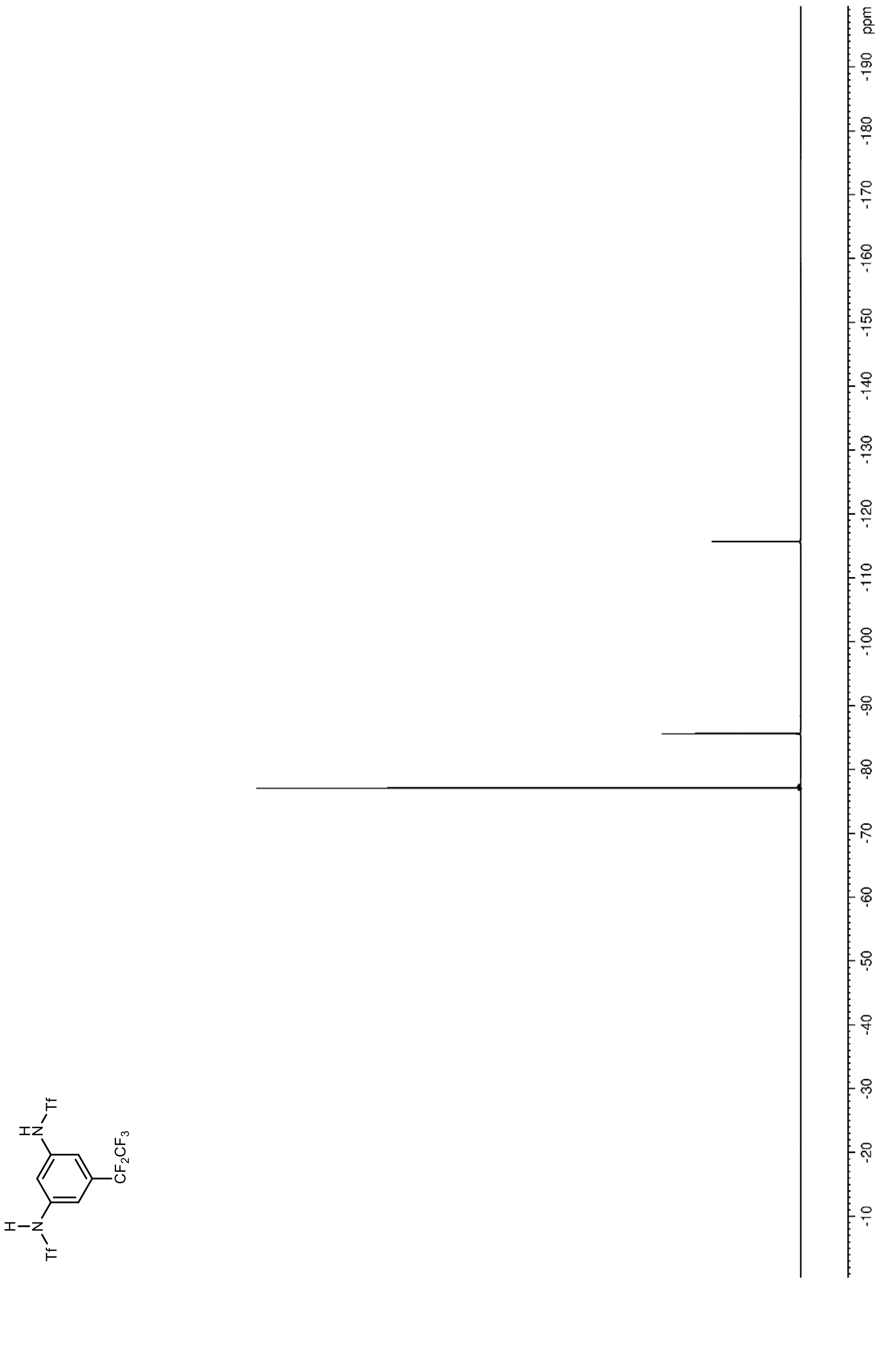

Figure 148. ^{1}H NMR (400 MHz, acetone-d_6) of **J2**

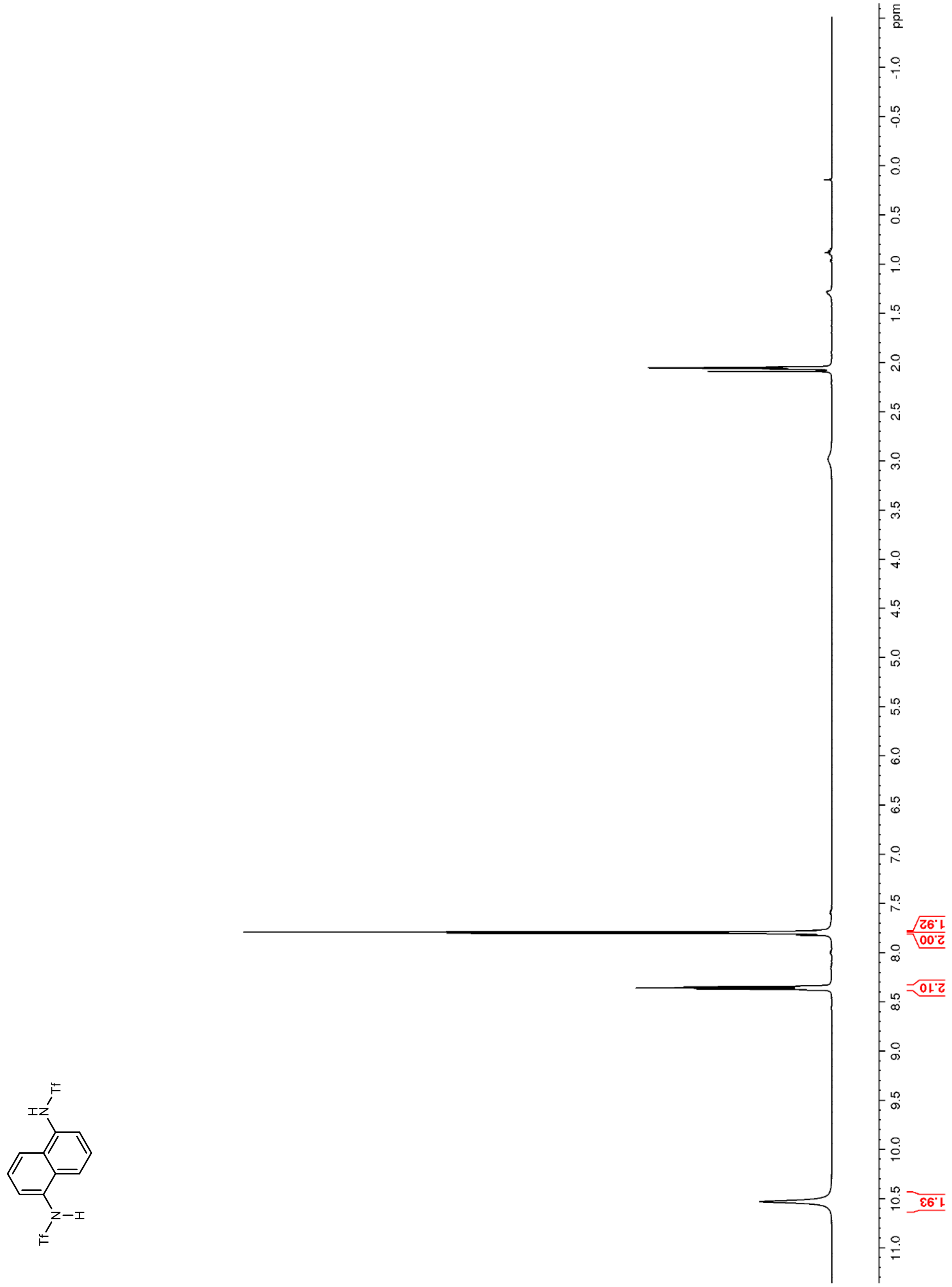

Figure 149. ^{13}C NMR (150 MHz, acetone-d_6) of **J2**

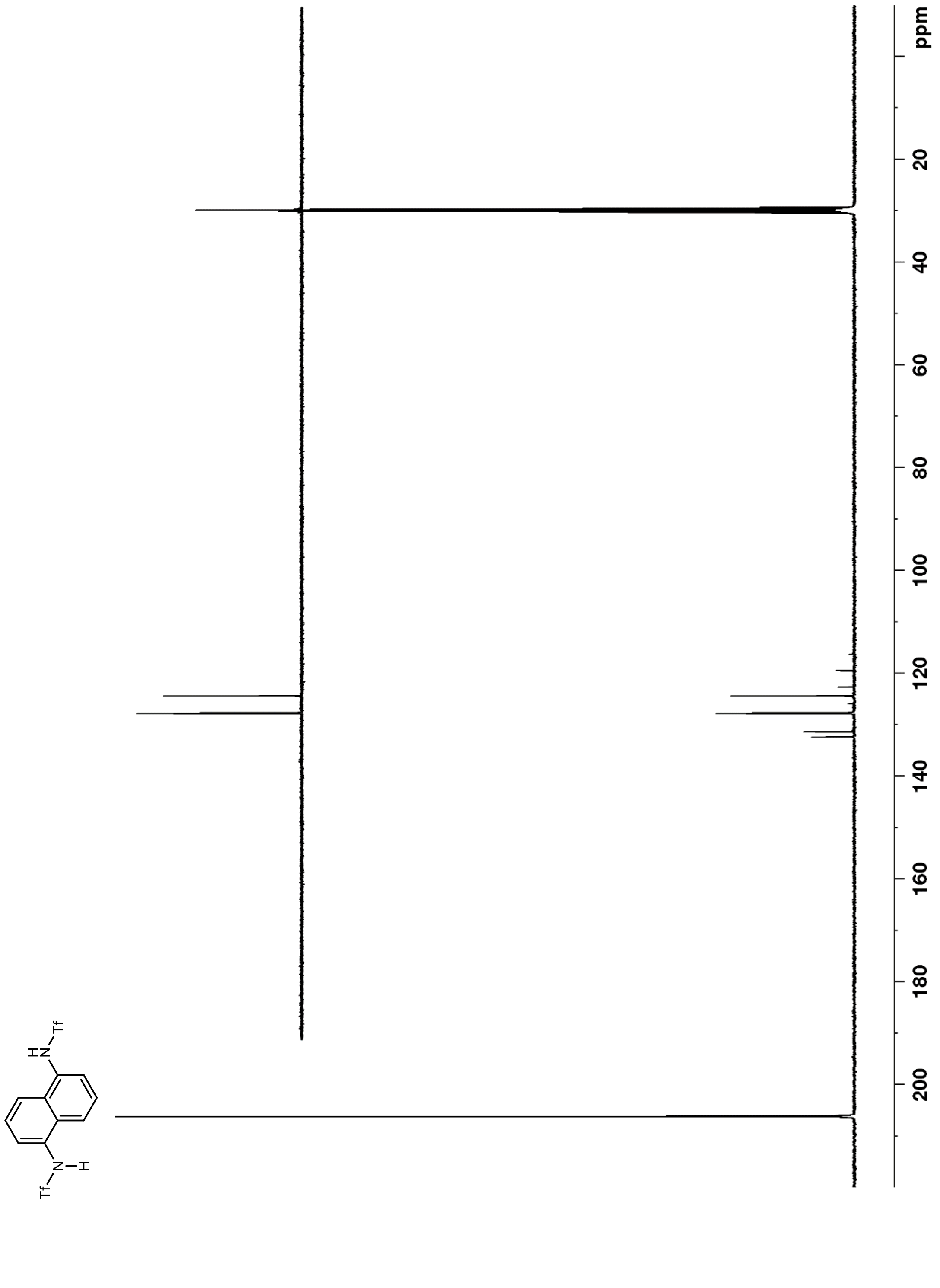

Figure 150. ^{19}F NMR (282 MHz, acetone-d_6) of **J2**

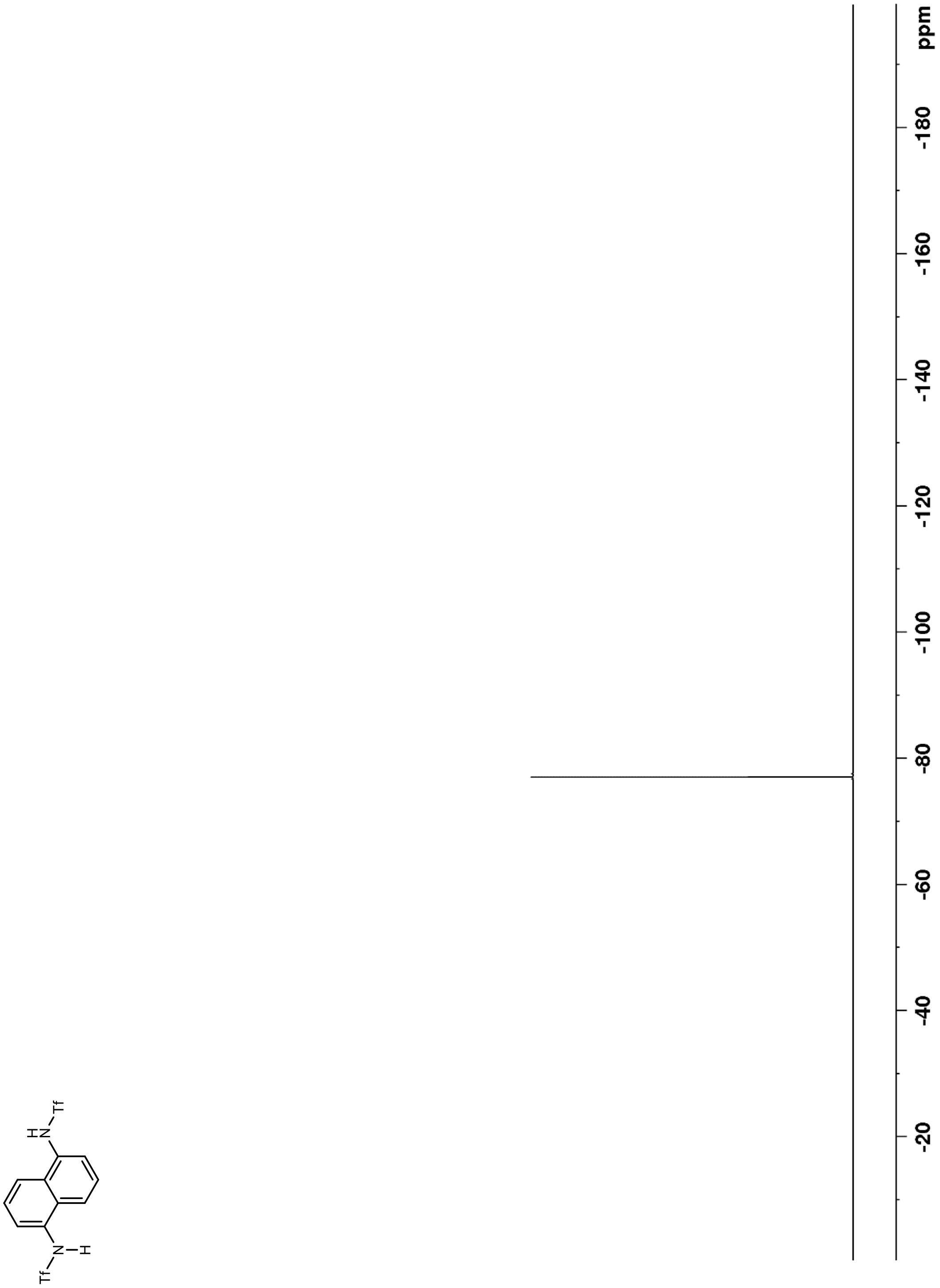

Figure 151. ^{1}H NMR (400 MHz, $CDCl_3$) of **J4**

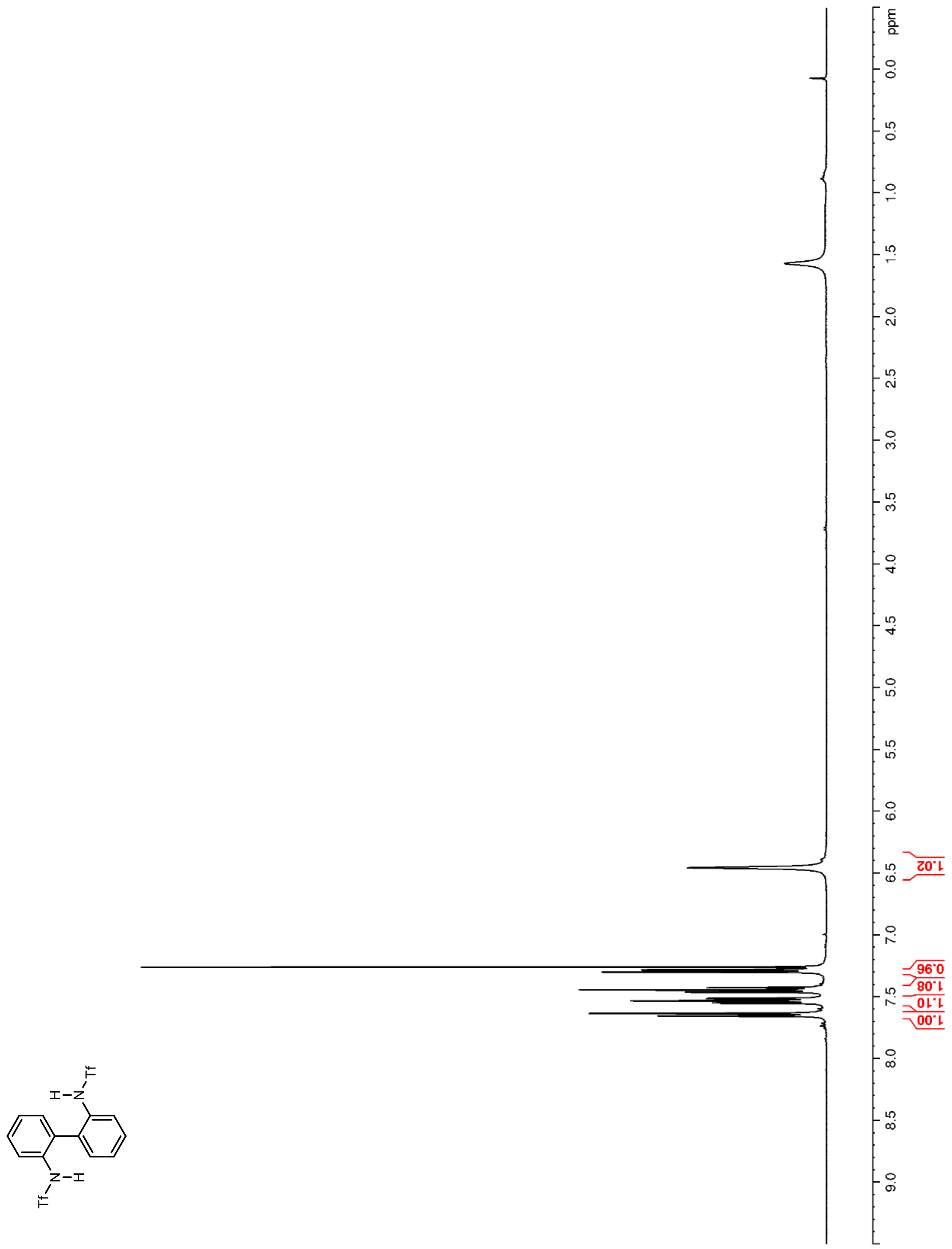

Figure 152. ^{13}C NMR (150 MHz, $CDCl_3$) of **J4**

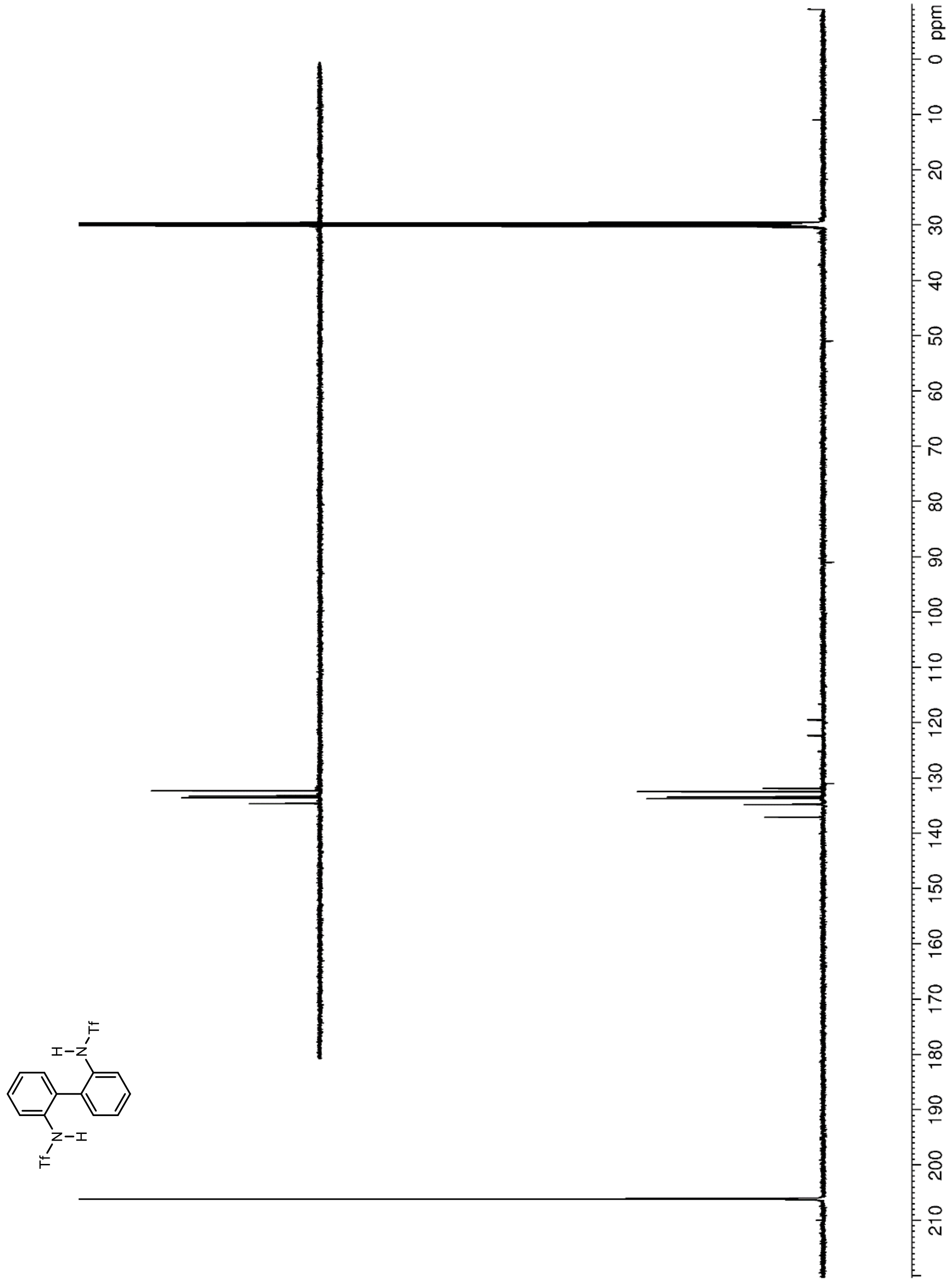

Figure 153. ^{19}F NMR (282 MHz, $CDCl_3$) of **J4**

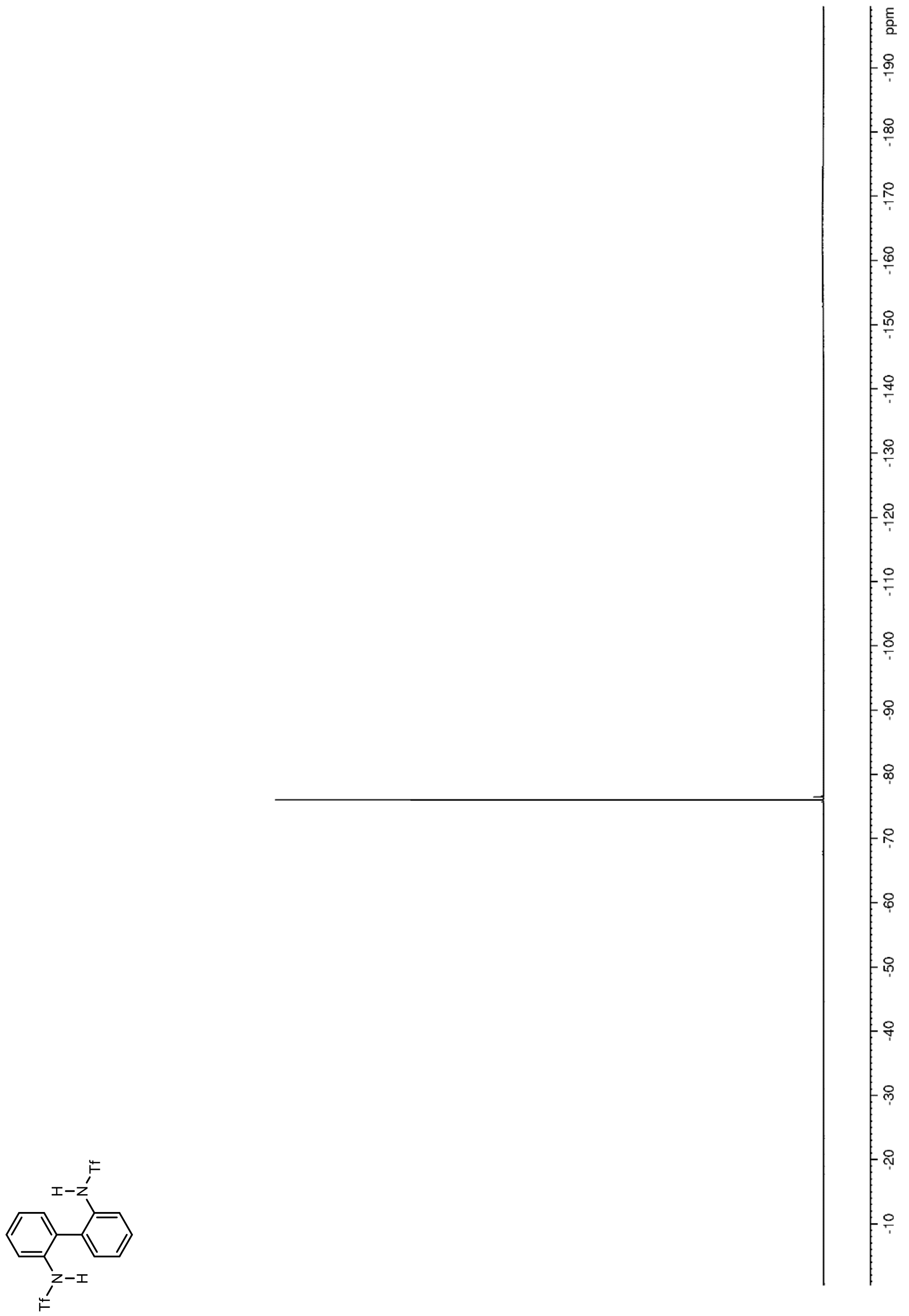

Figure 154. ^{1}H NMR (400 MHz, acetone-d_6) of **J5**

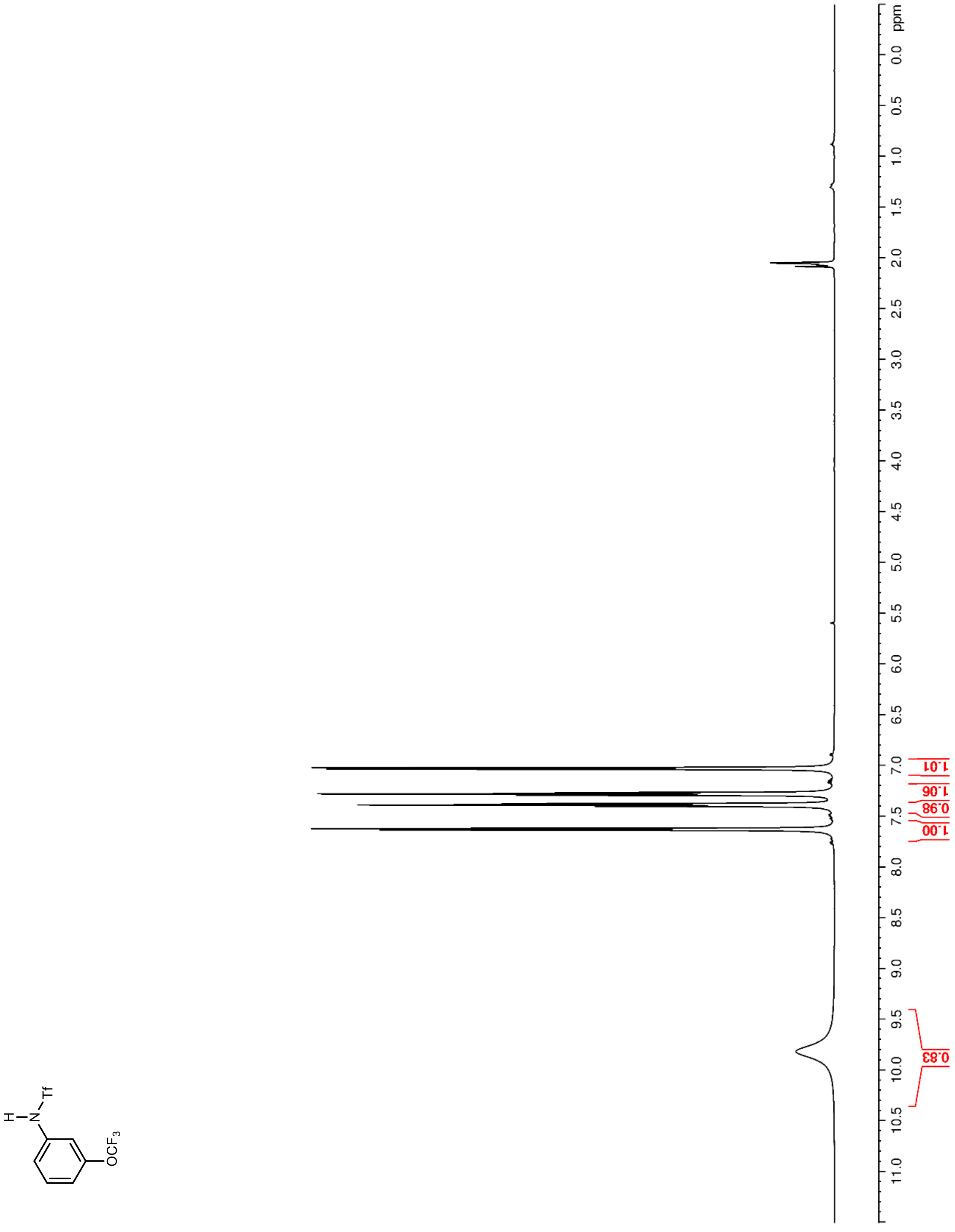

Figure 155. ^{13}C NMR (150 MHz, acetone-d_6) of **J5**

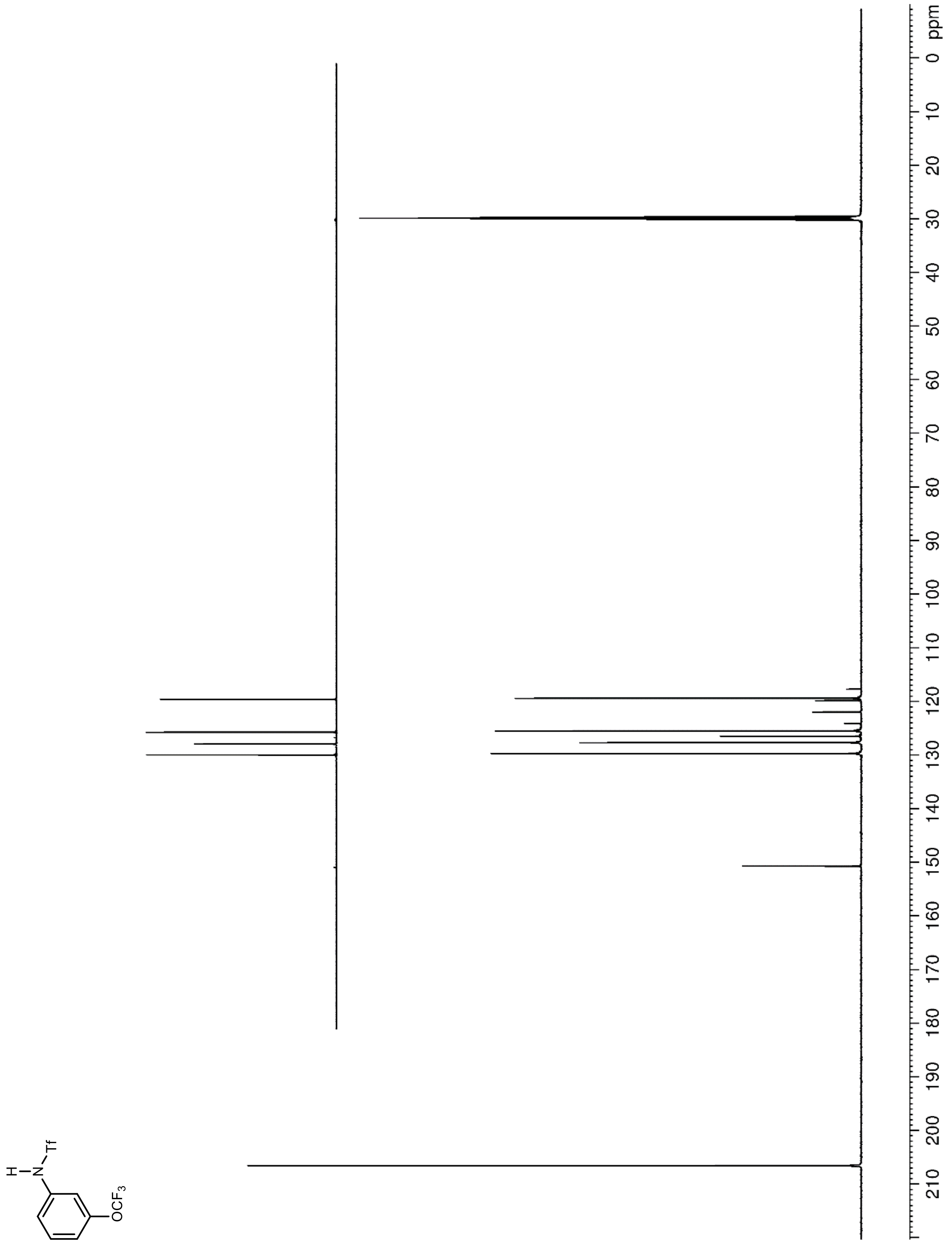

Figure 156. ^{19}F NMR (282 MHz, acetone-d_6) of **J5**

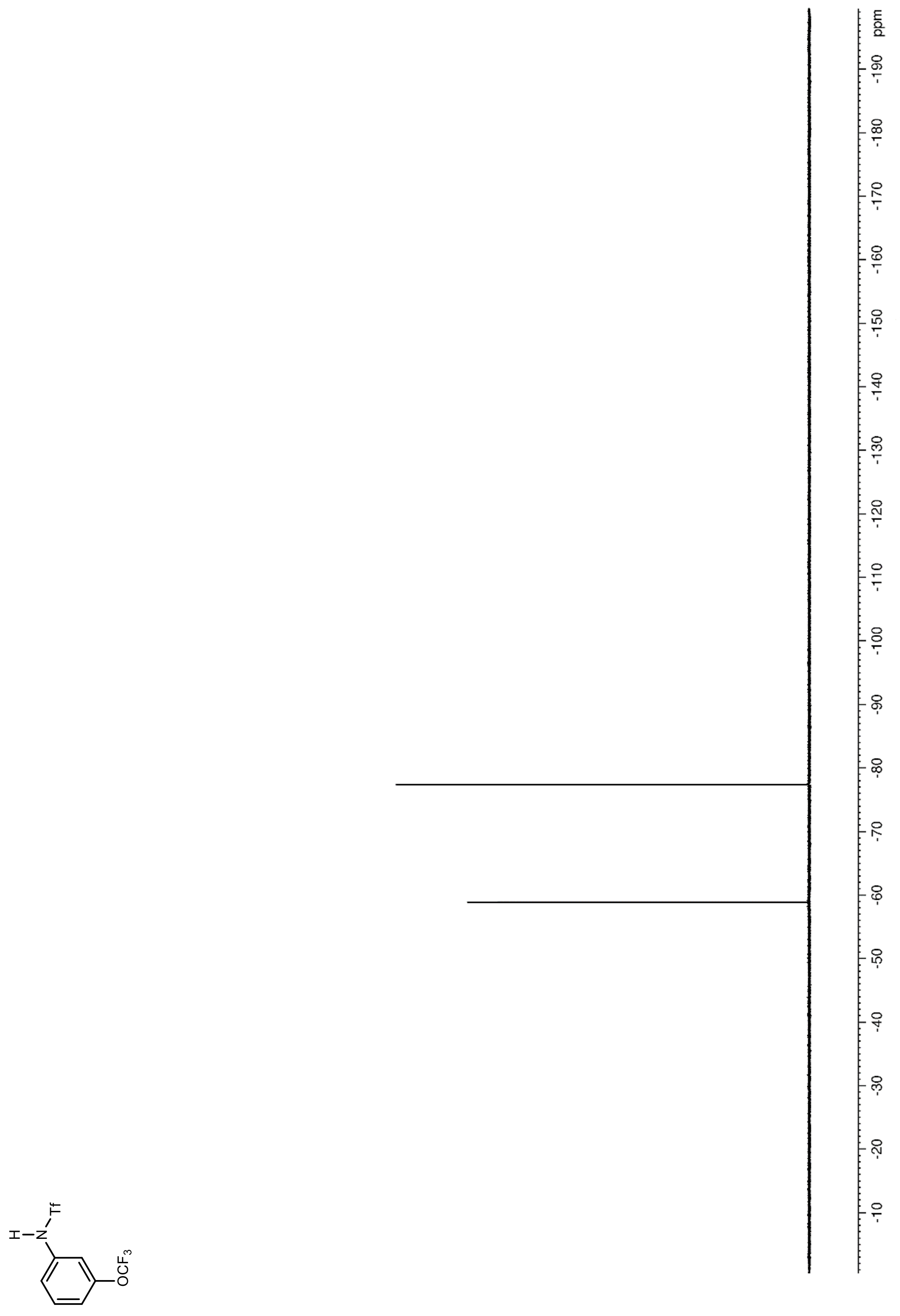

Figure 157. ^{1}H NMR (400 MHz, $CDCl_3$) of **J6**

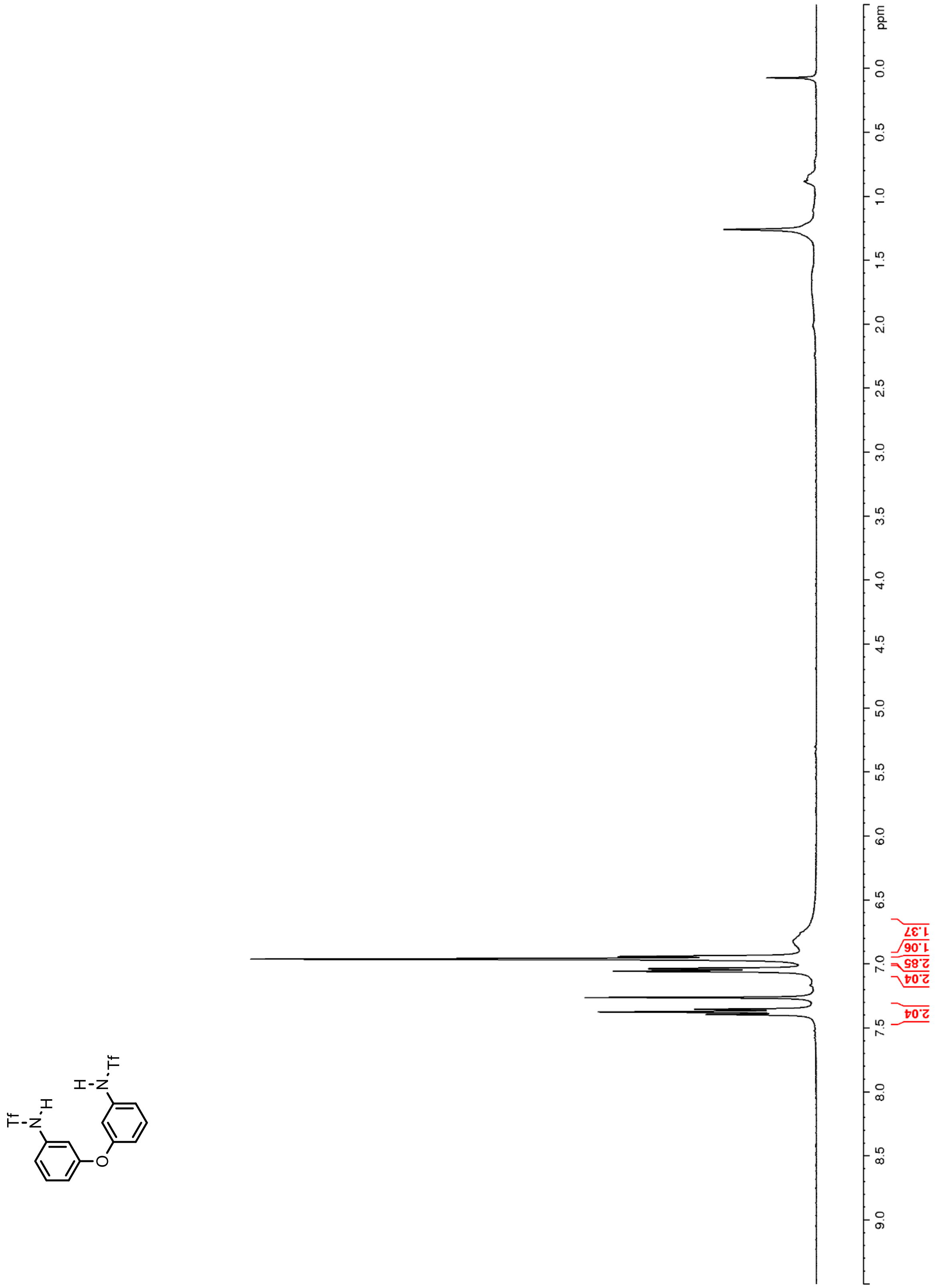

Figure 158. ^{13}C NMR (150 MHz, $CDCl_3$) of **J6**

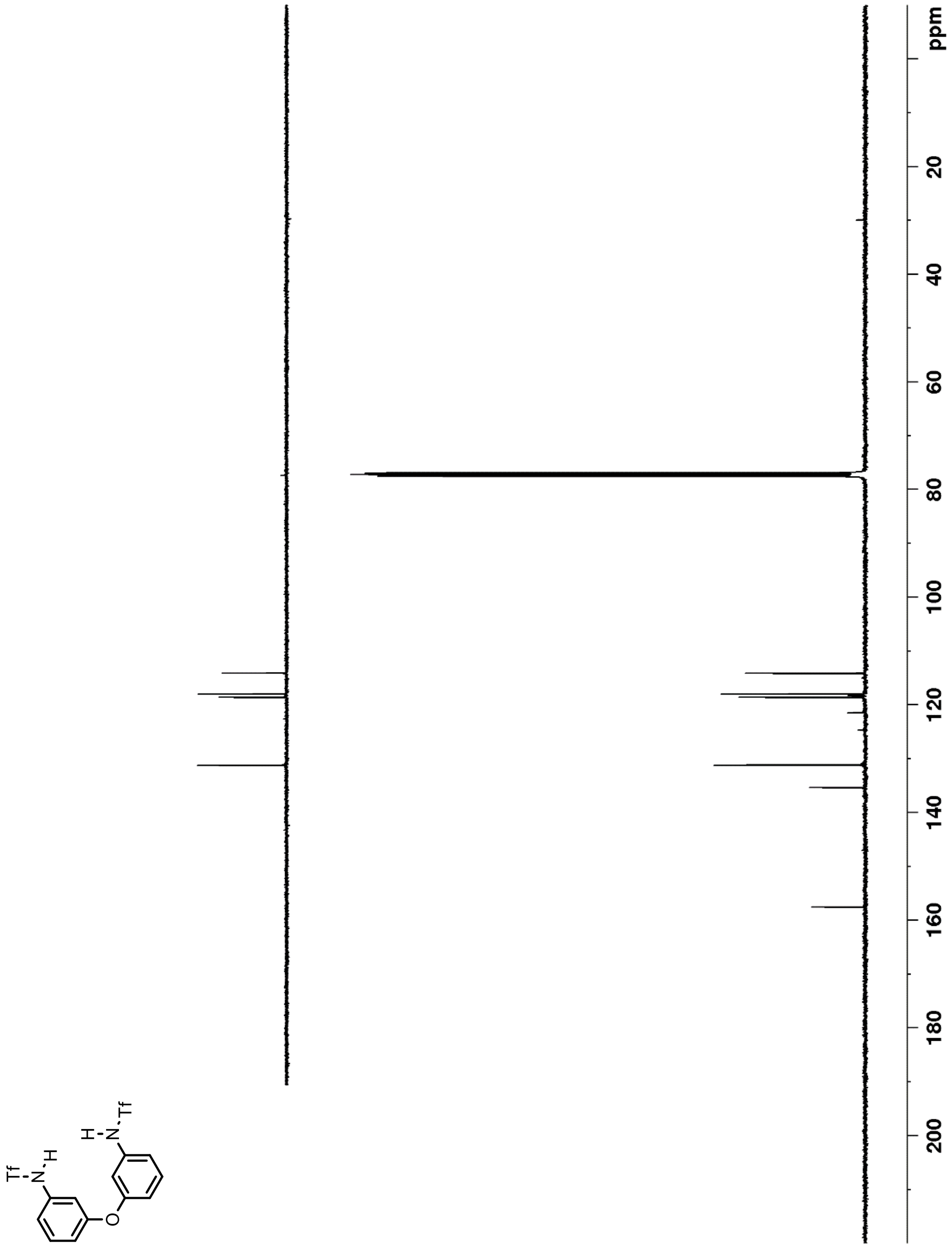

Figure 159. ^{19}F NMR (282 MHz, $CDCl_3$) of **J6**

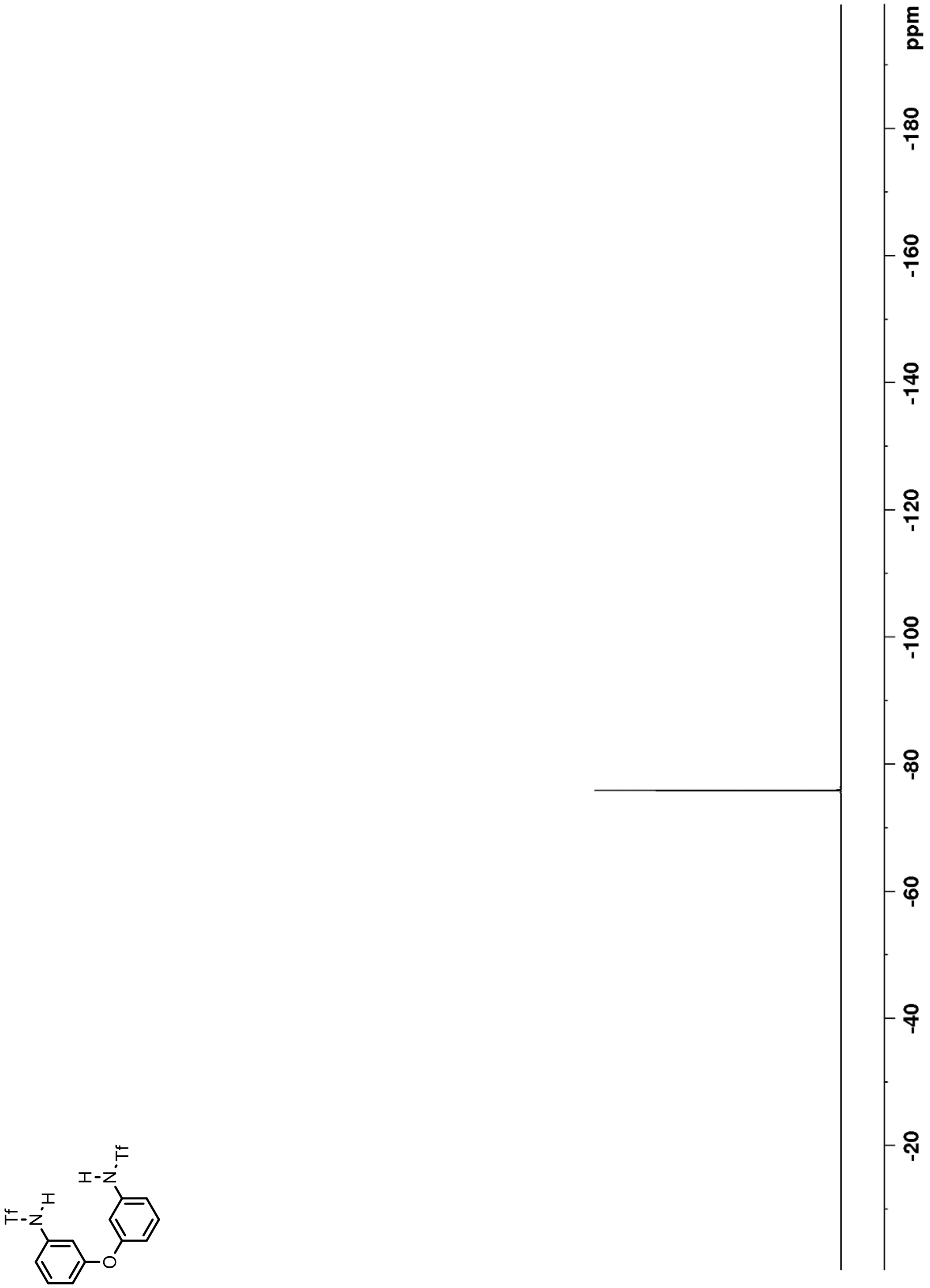

Figure 160. ^{1}H NMR (400 MHz, $CDCl_3$) of **J7**

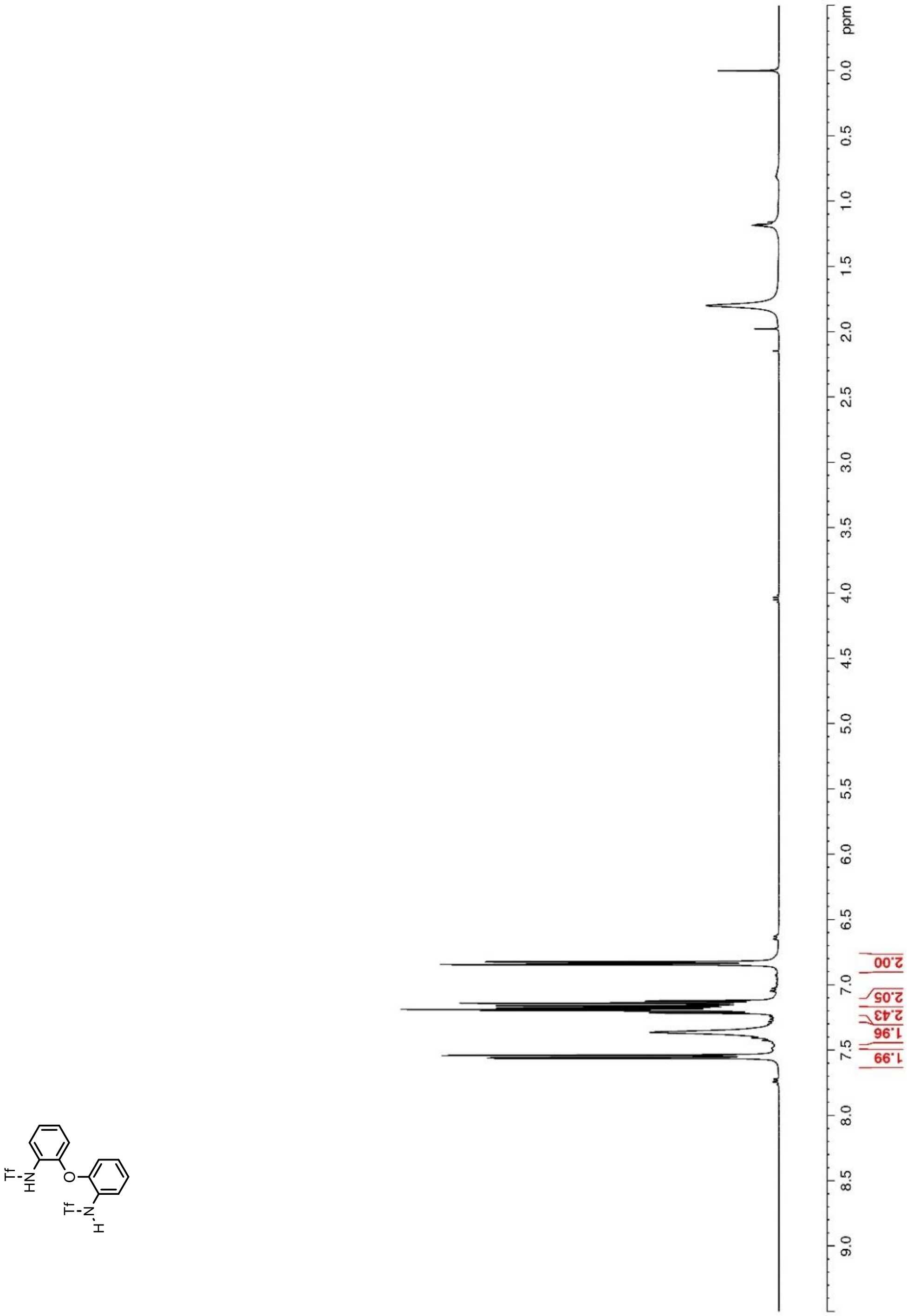

Figure 161. ^{13}C NMR (150 MHz, $CDCl_3$) of **J7**

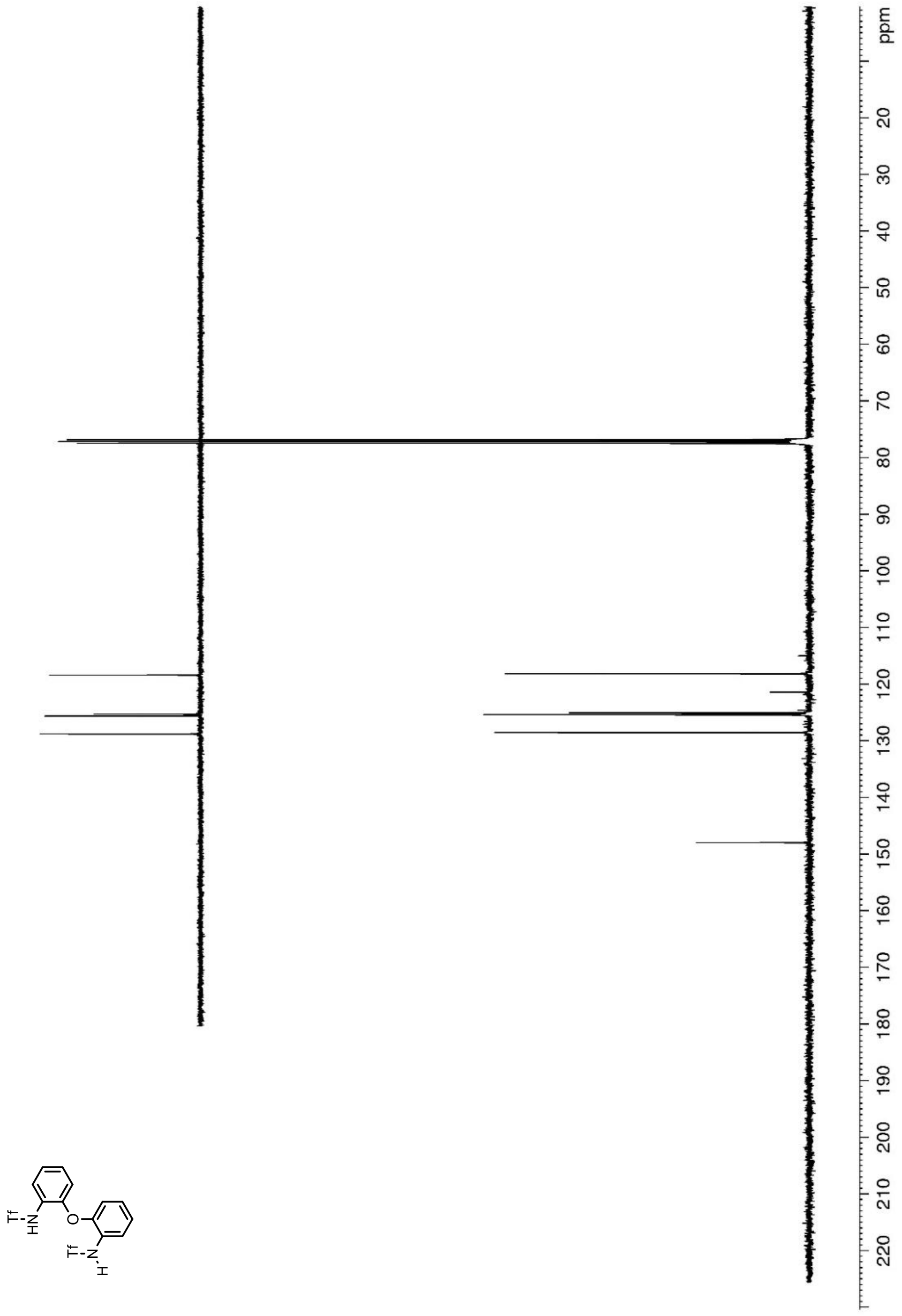

Figure 162. ^{19}F NMR (282 MHz, $CDCl_3$) of **J7**

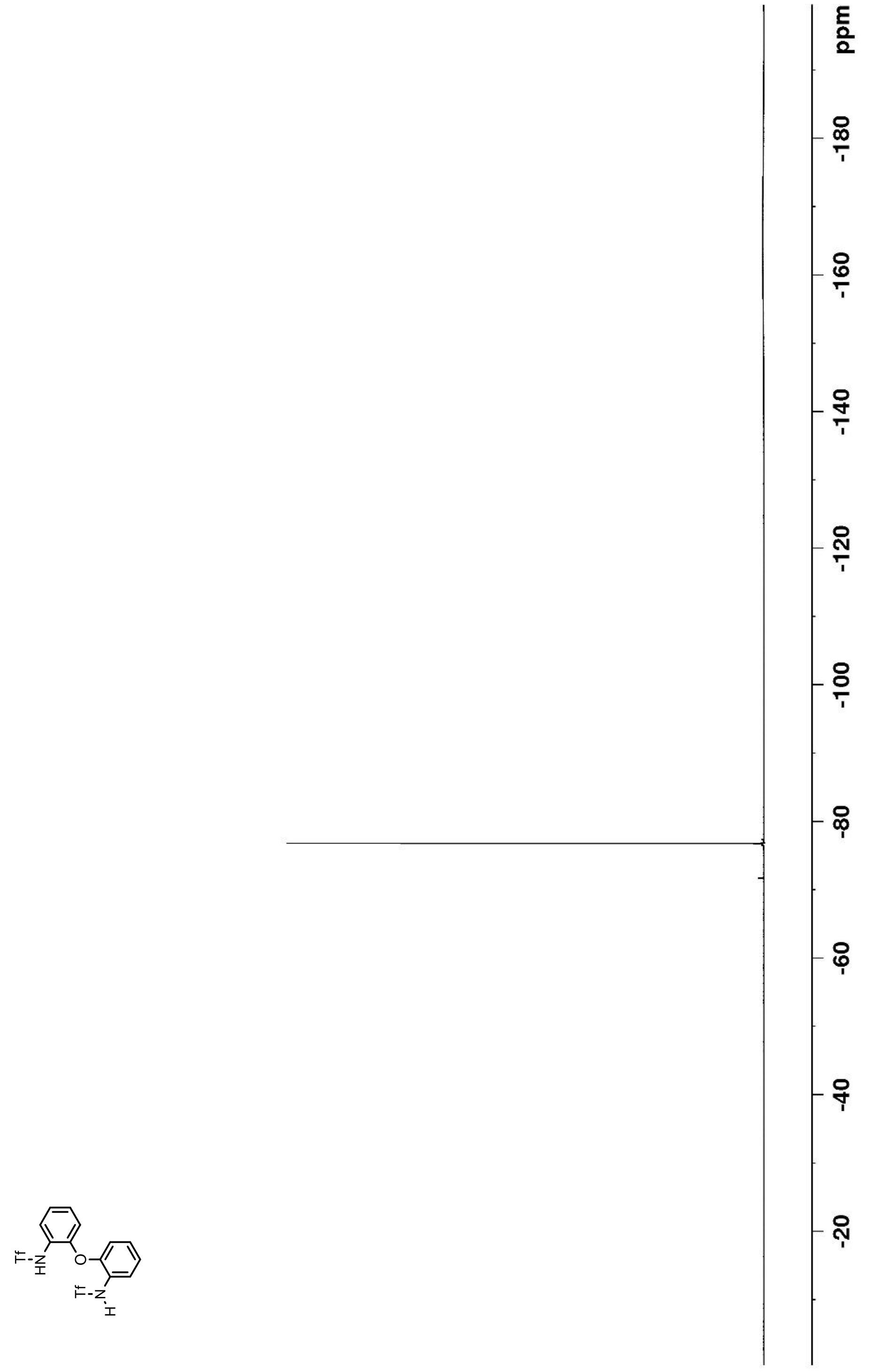

Figure 163. ^{1}H NMR (400 MHz, acetone-d_6) of **J8**

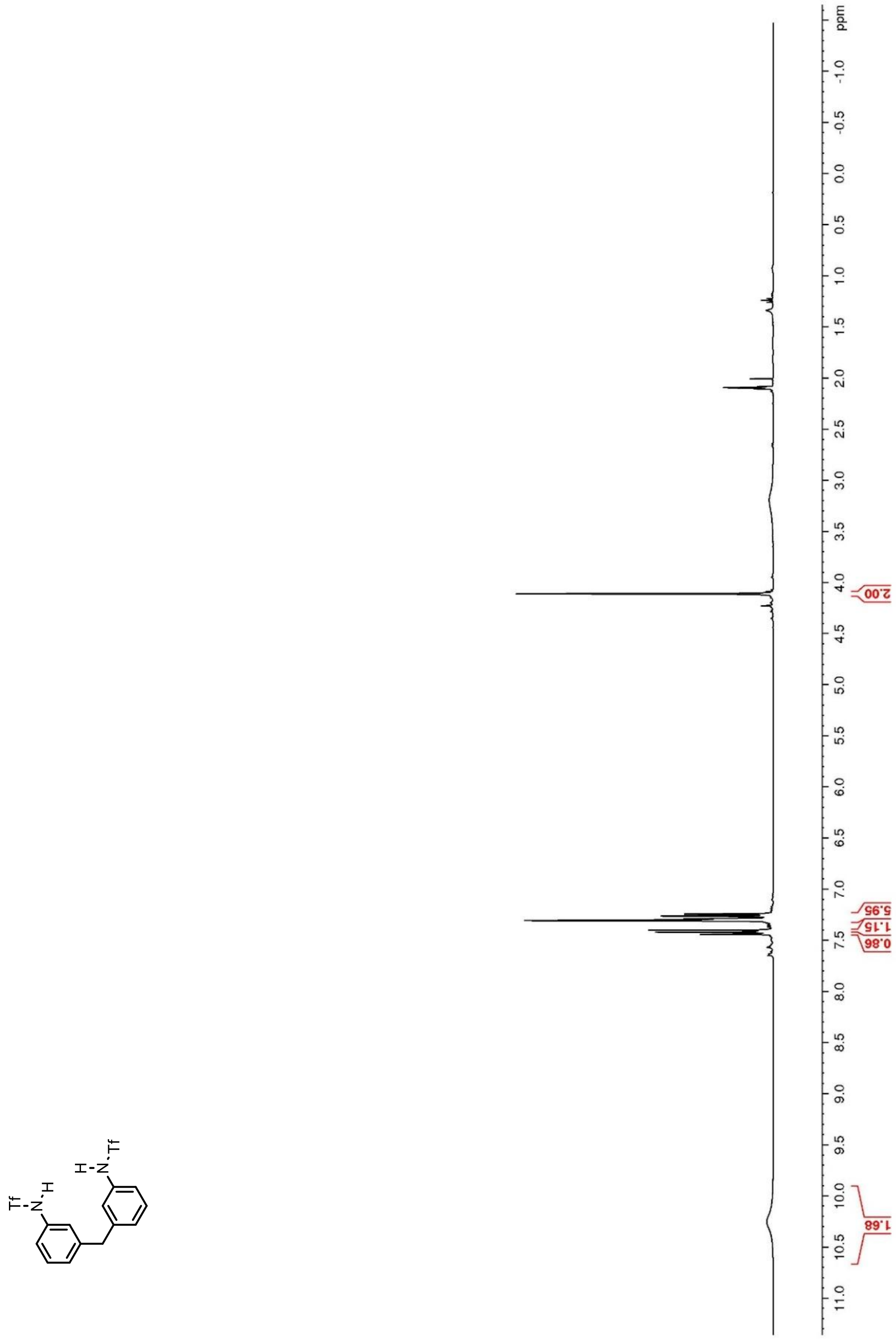

Figure 164. ^{13}C NMR (150 MHz, acetone-d_6) of **J8**

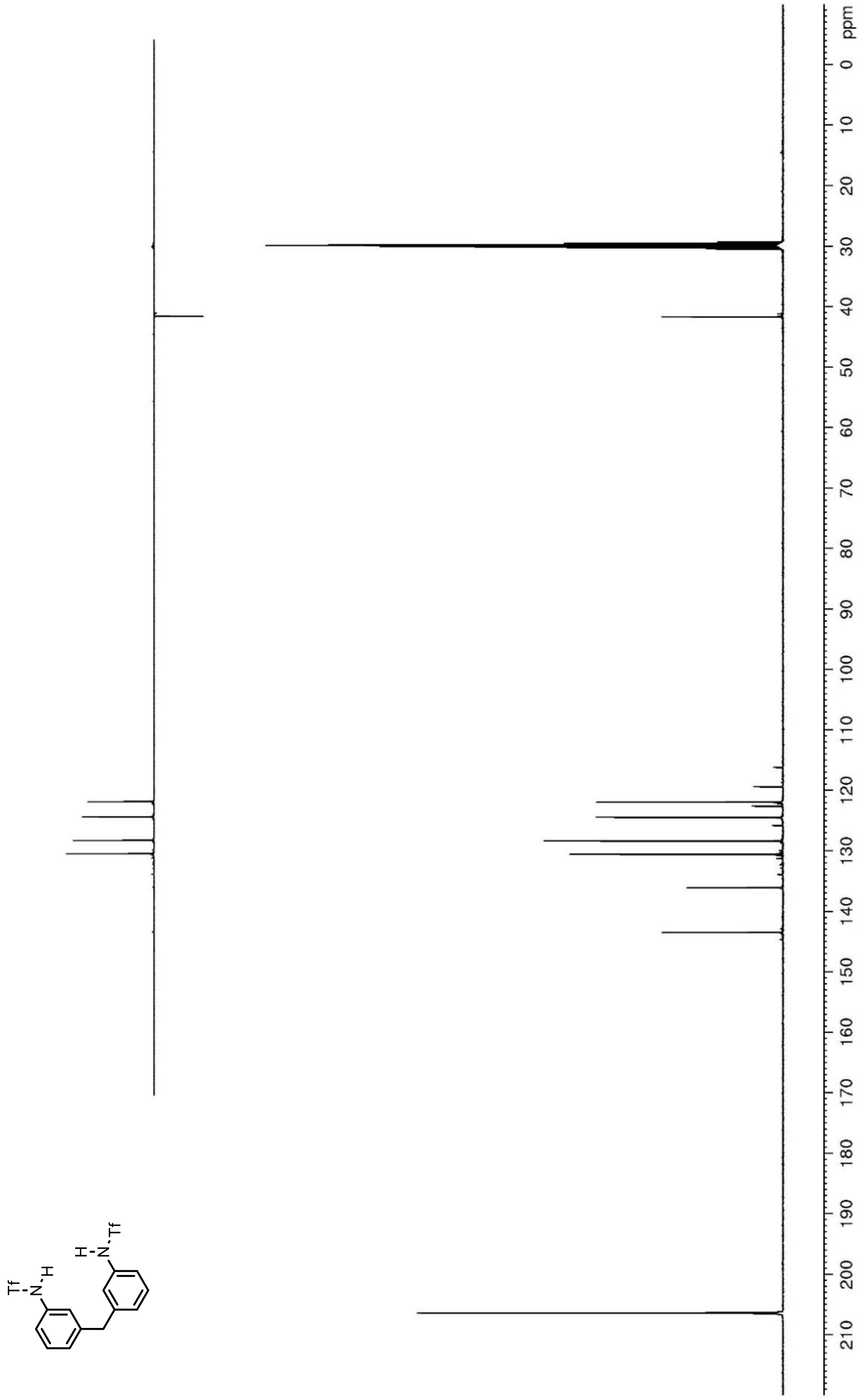

Figure 165. ^{19}F NMR (282 MHz, acetone-d_6) of **J8**

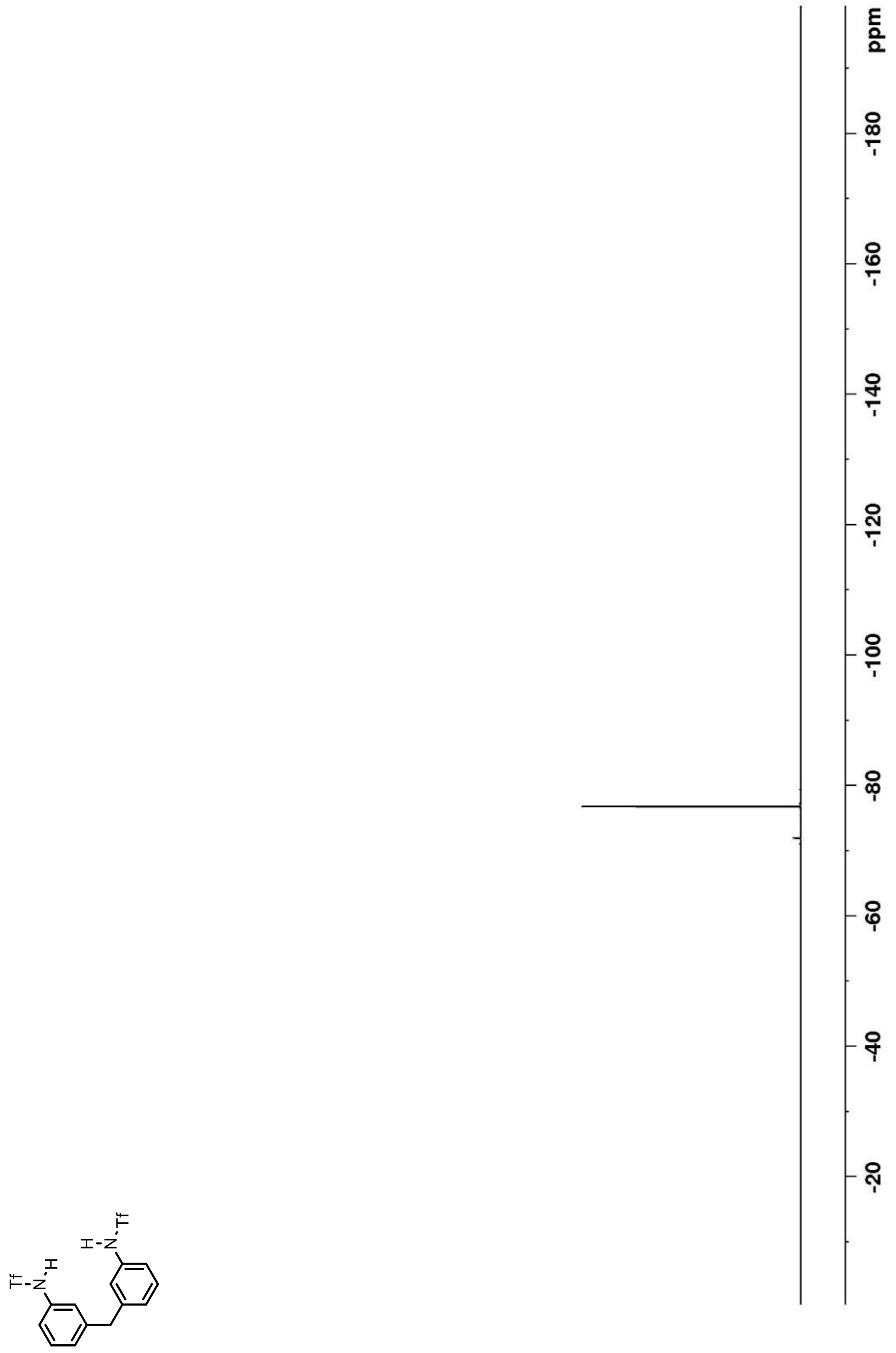

Figure 166. ^{1}H NMR (400 MHz, acetone-d_6) of **K1**

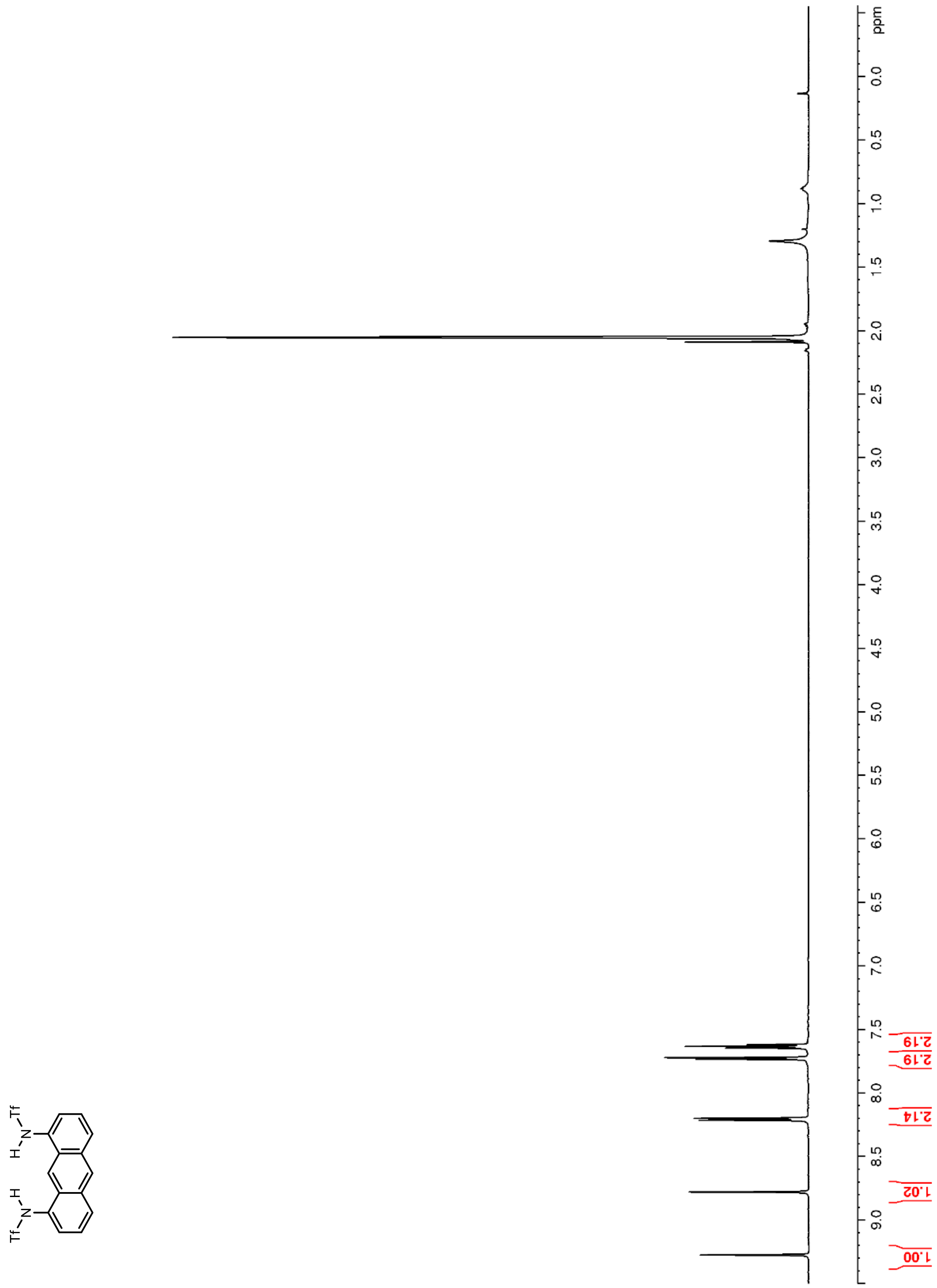

Figure 167. ^{13}C NMR (150 MHz, acetone-d_6) of **K1**

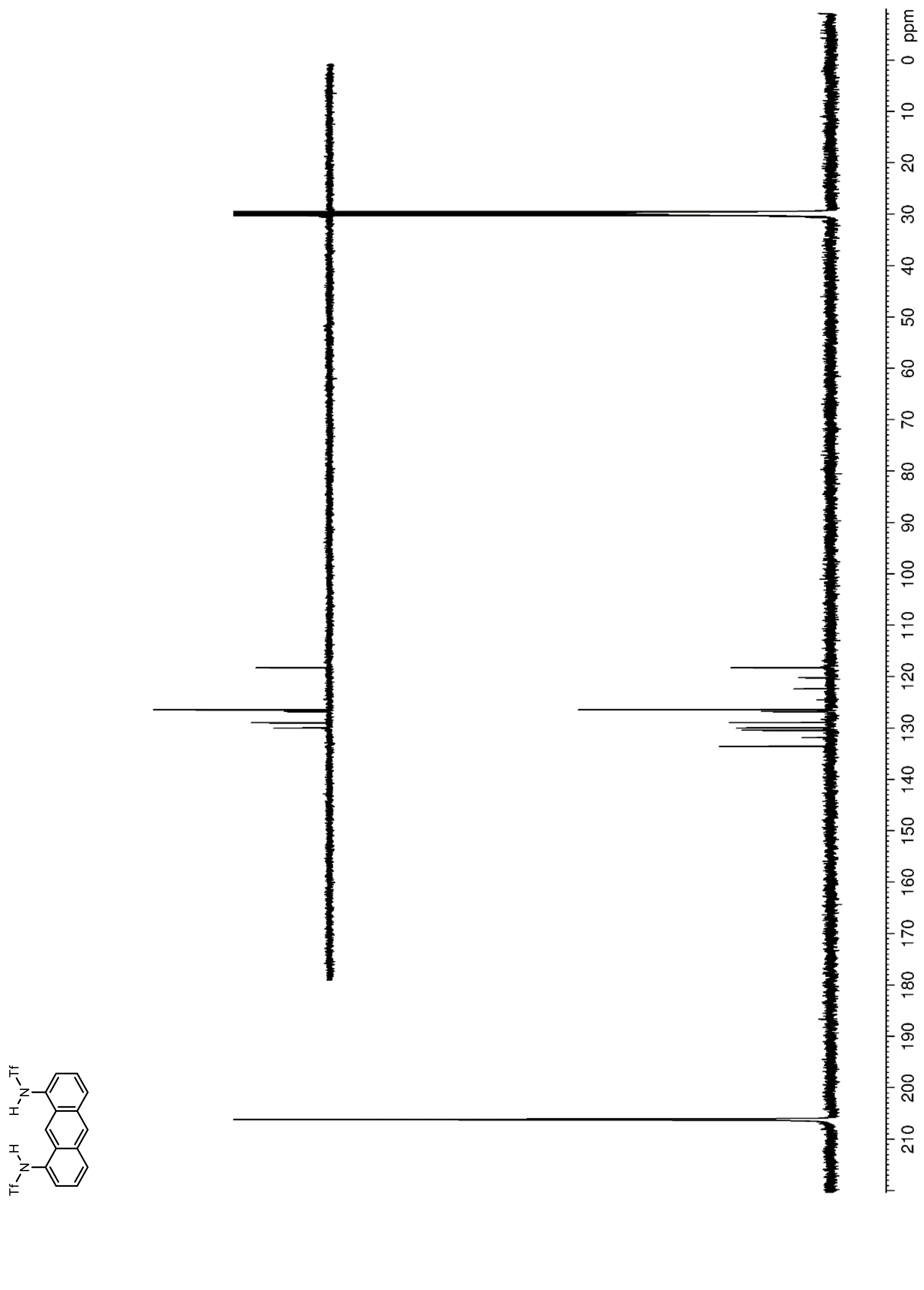

Figure 168. ^{19}F NMR (282 MHz, acetone-d_6) of **K1**

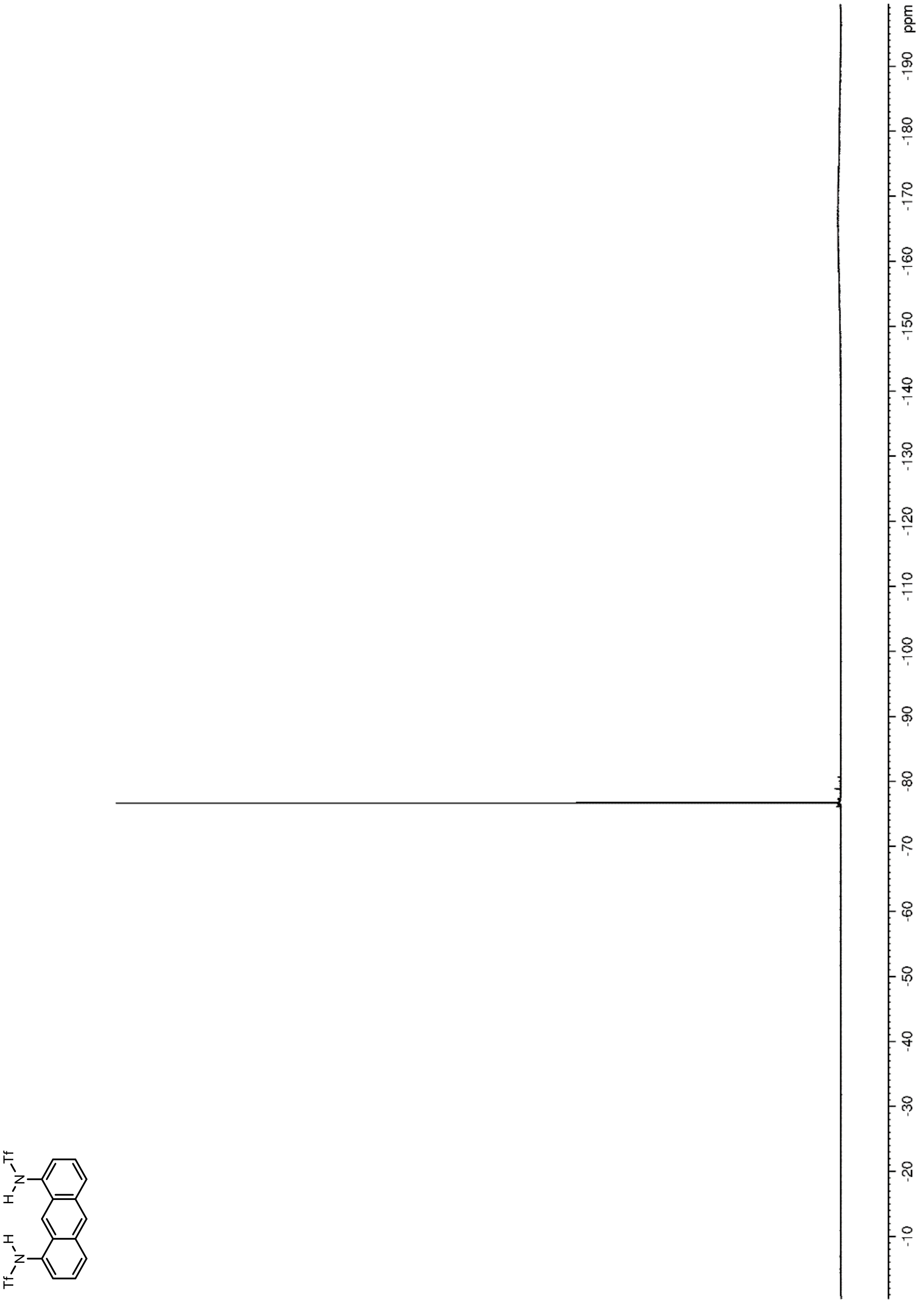

Figure 169. ^{1}H NMR (400 MHz, acetone-d_6) of **K2**

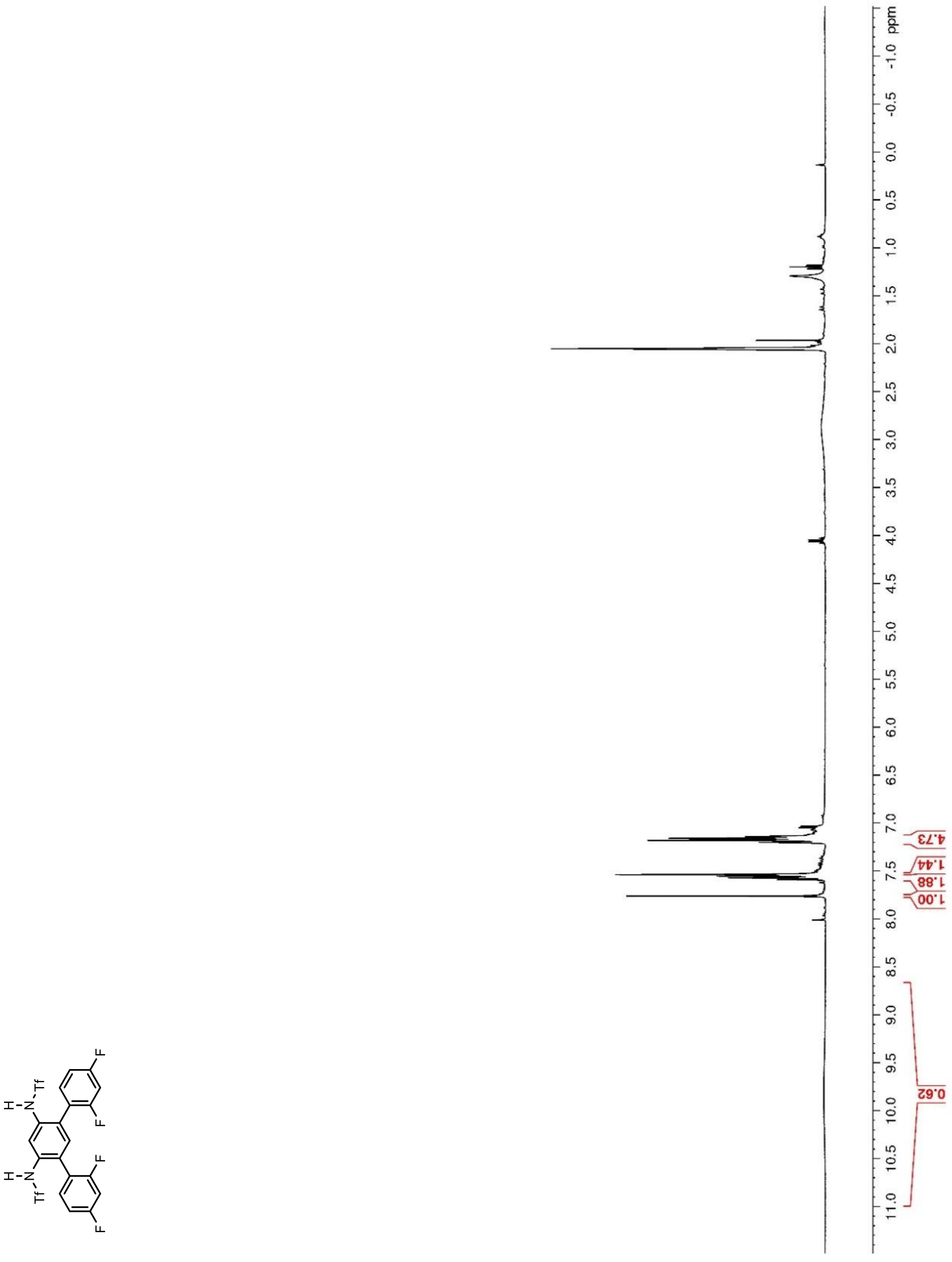

Figure 170. ^{13}C NMR (150 MHz, acetone-d_6) of **K2**

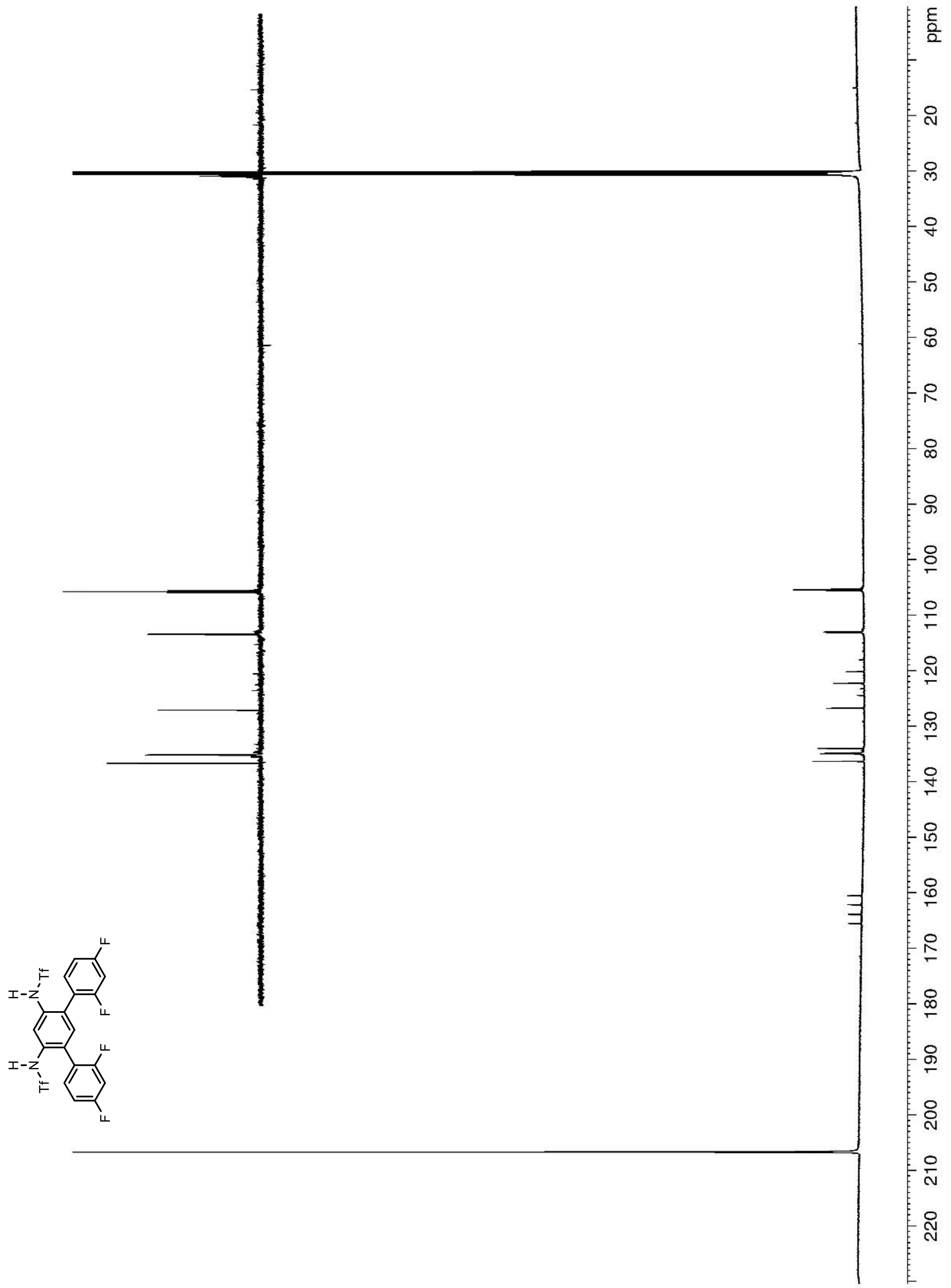

Figure 171. ^{19}F NMR (282 MHz, acetone-d_6) of **K2**

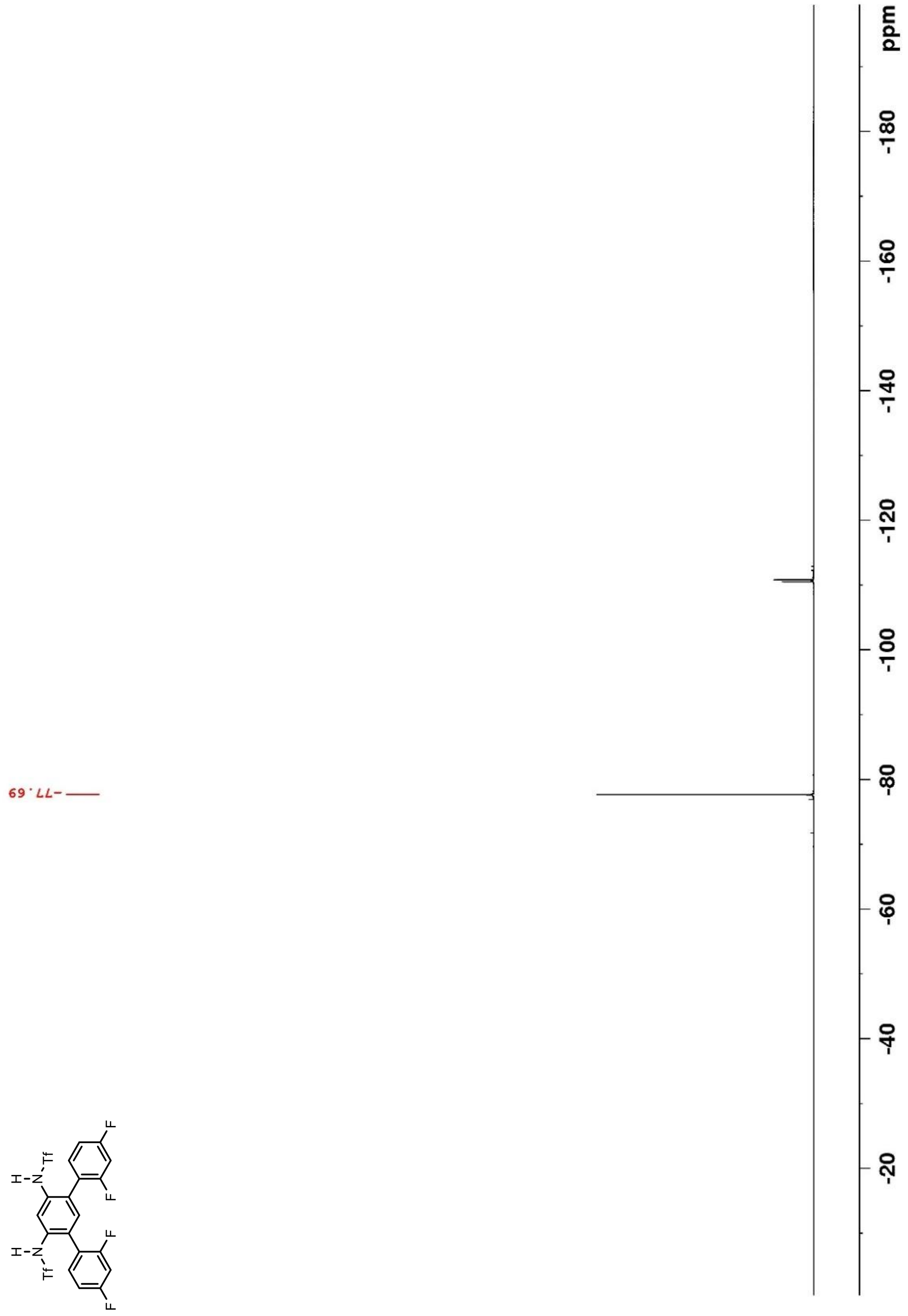

Figure 172. ^{1}H NMR (400 MHz, acetone-d_6) of **K3**

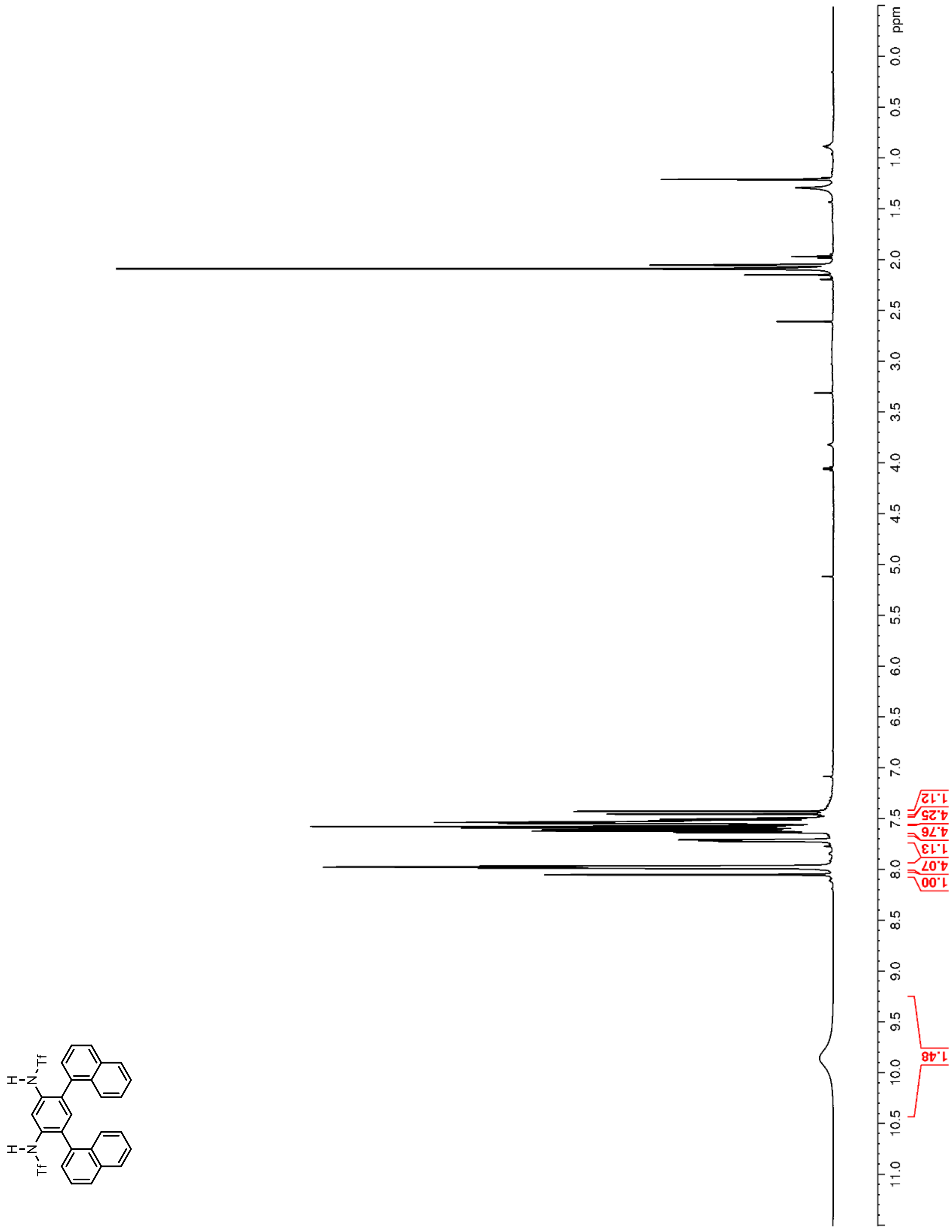

Figure 173. ^{13}C NMR (150 MHz, acetone-d_6) of **K3**

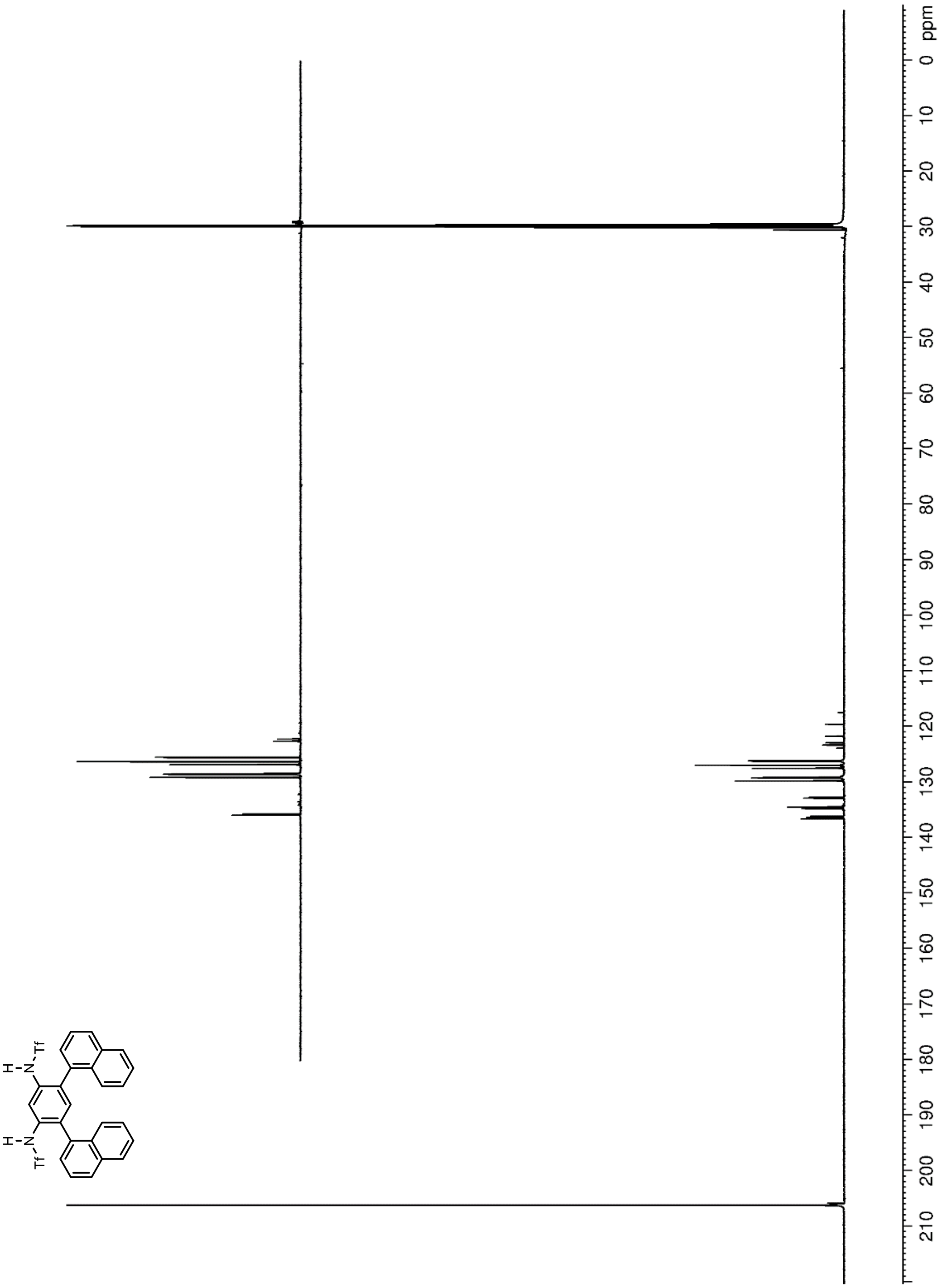

Figure 174. ^{19}F NMR (282 MHz, acetone-d_6) of **K3**

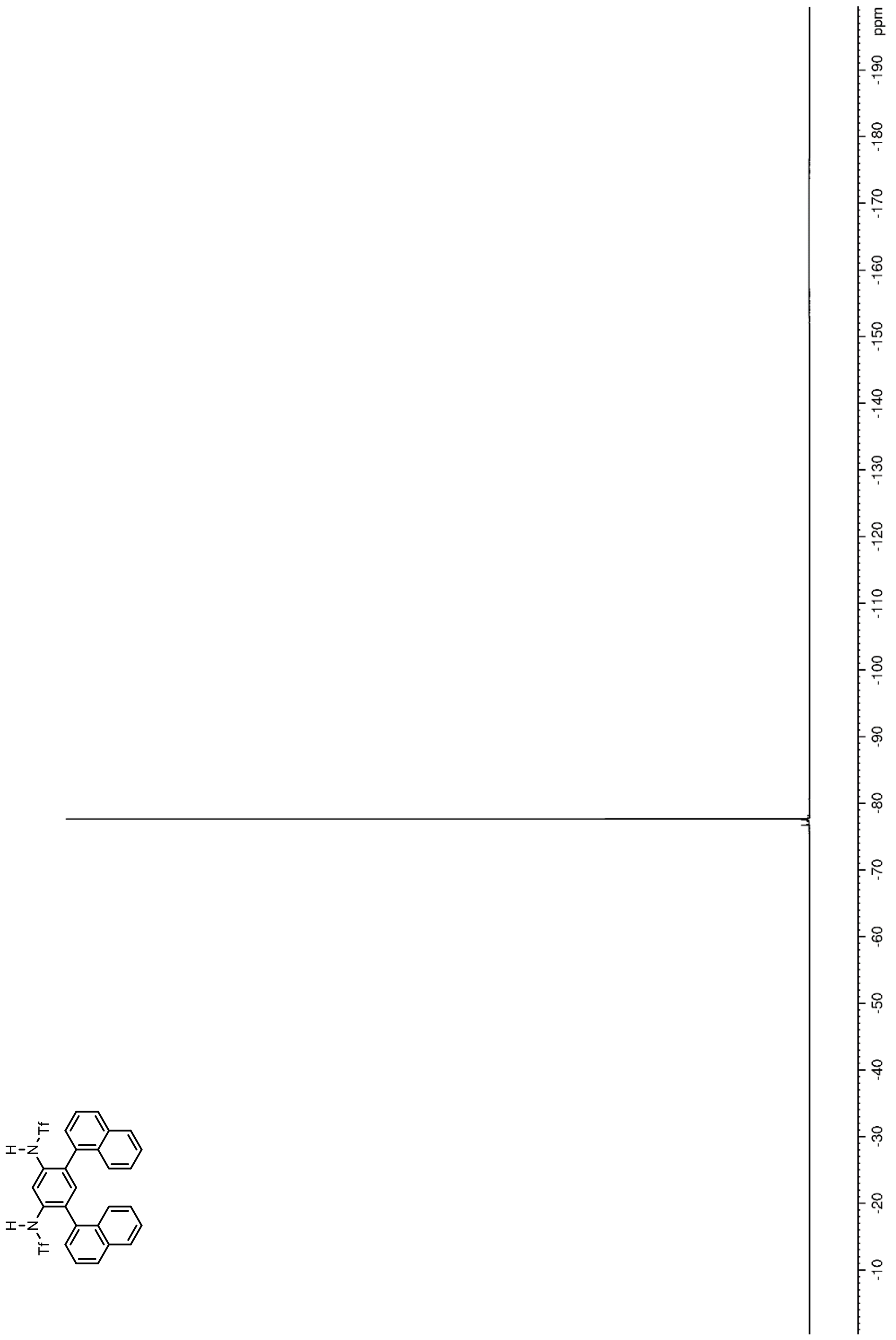

Figure 175. ^{1}H NMR (400 MHz, $CDCl_3$) of **K4**

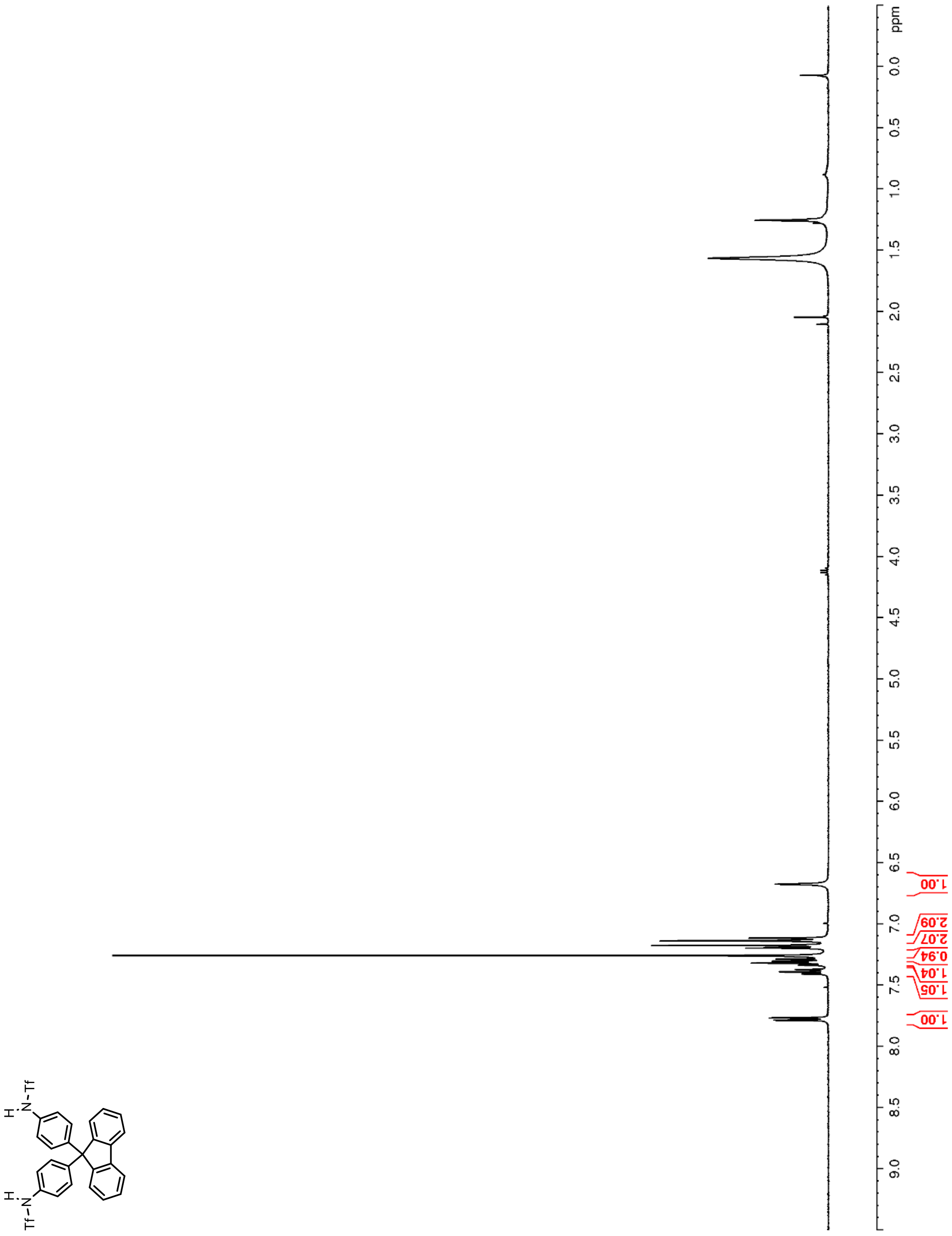

Figure 176. ^{13}C NMR (150 MHz, acetone-d_6) of **K4**

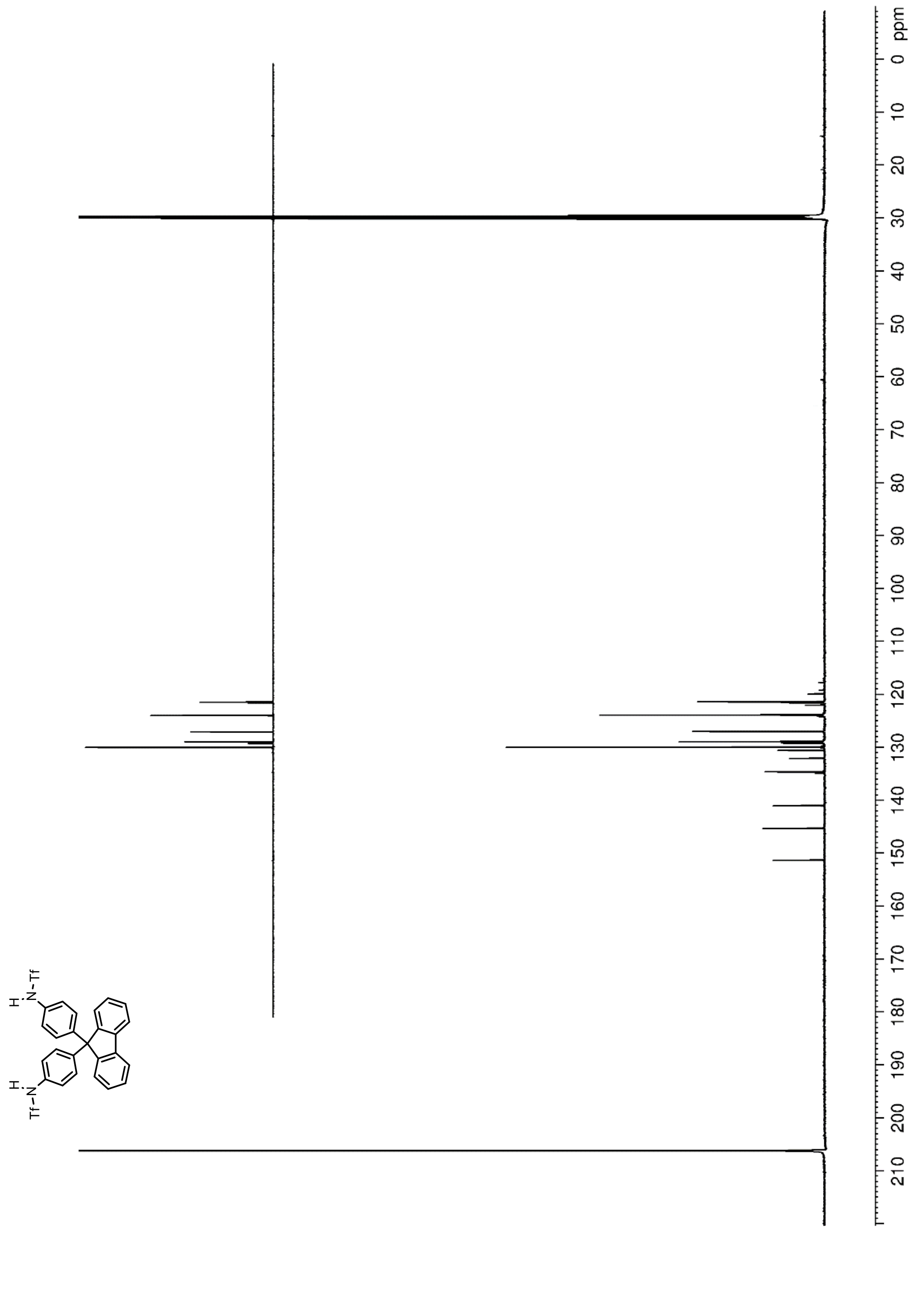

Figure 177. ^{19}F NMR (282 MHz, $CDCl_3$) of **K4**

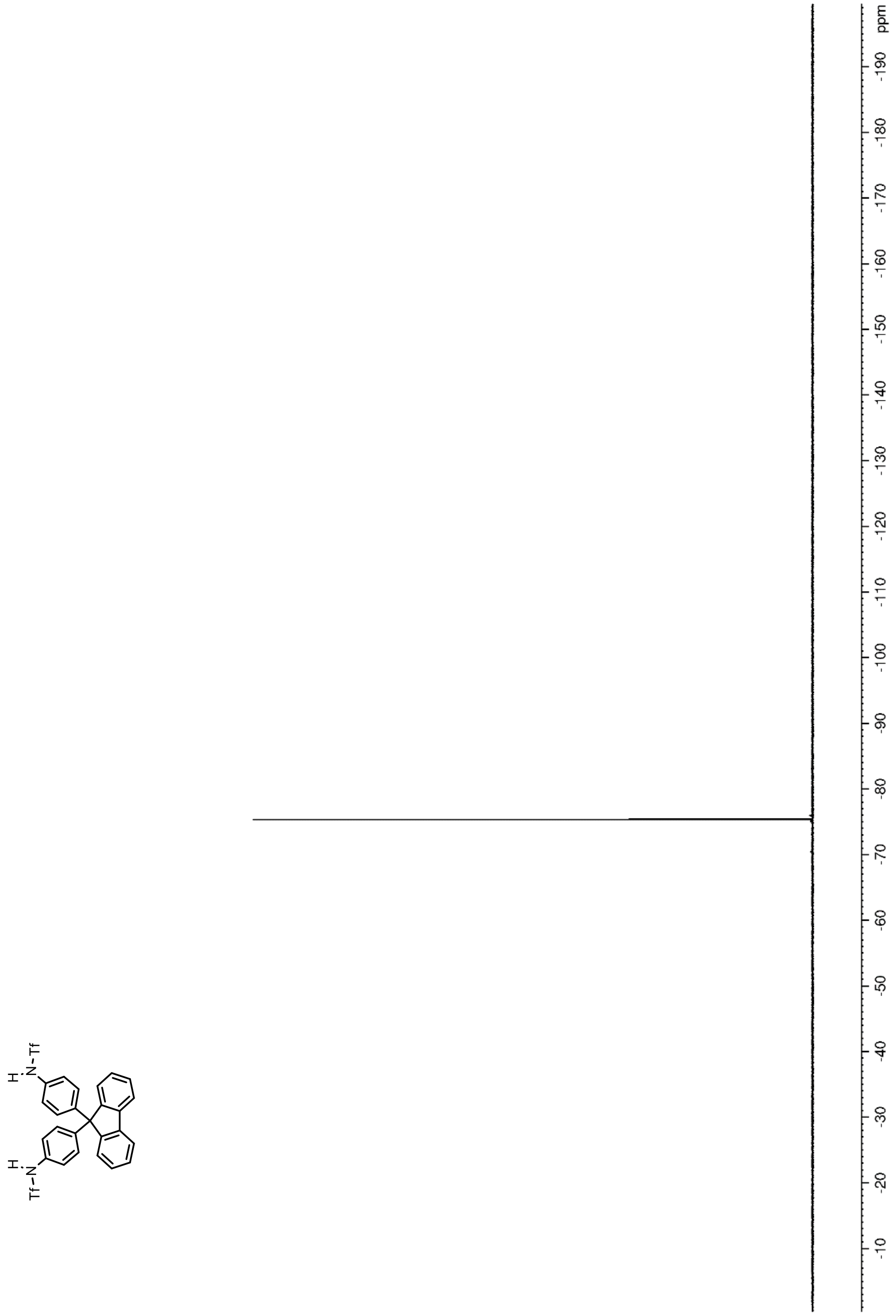

Figure 178. ^{1}H NMR (400 MHz, acetone-d_6) of **K5**

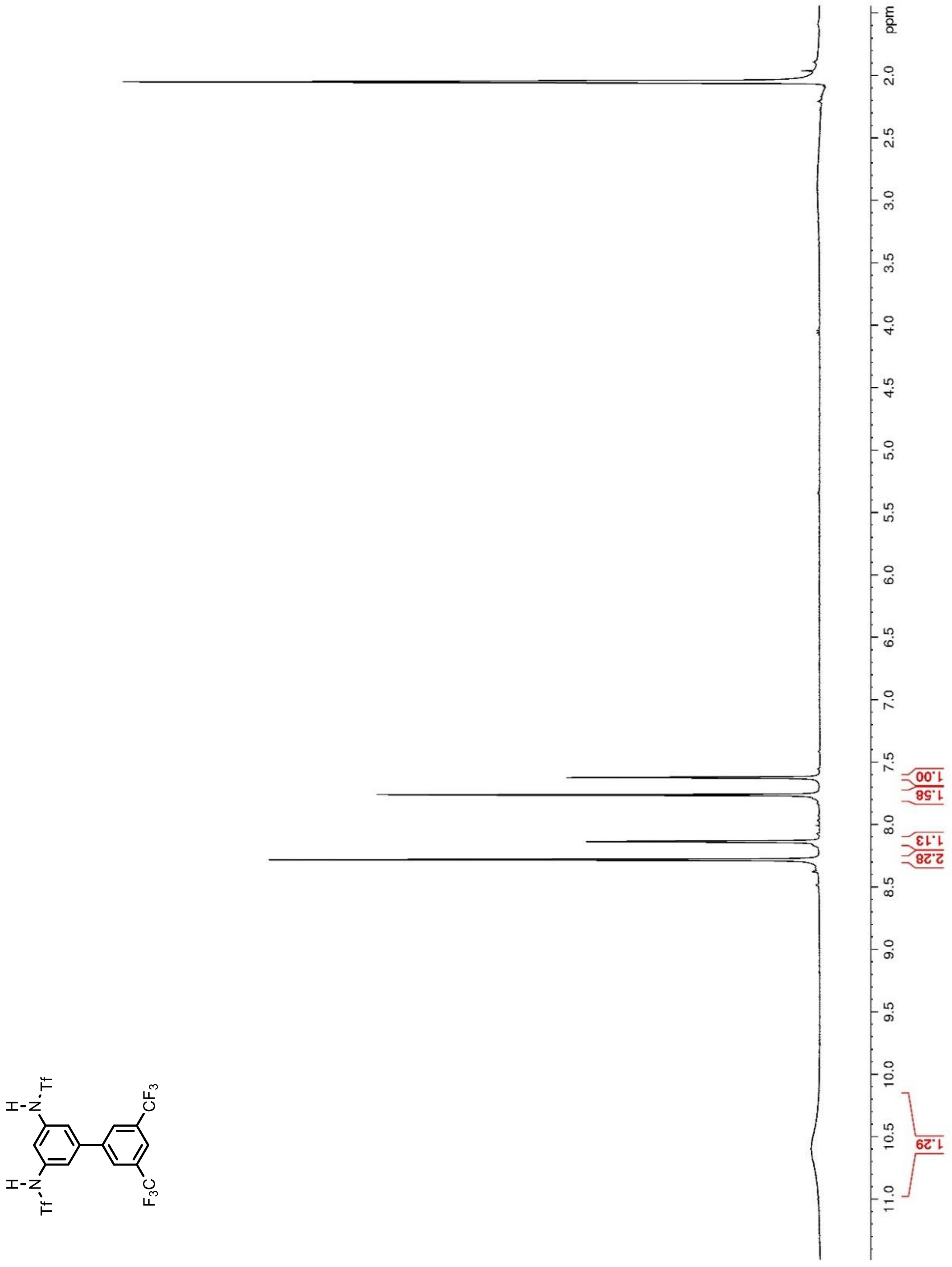

Figure 179. ^{13}C NMR (150 MHz, acetone-d_6) of **K5**

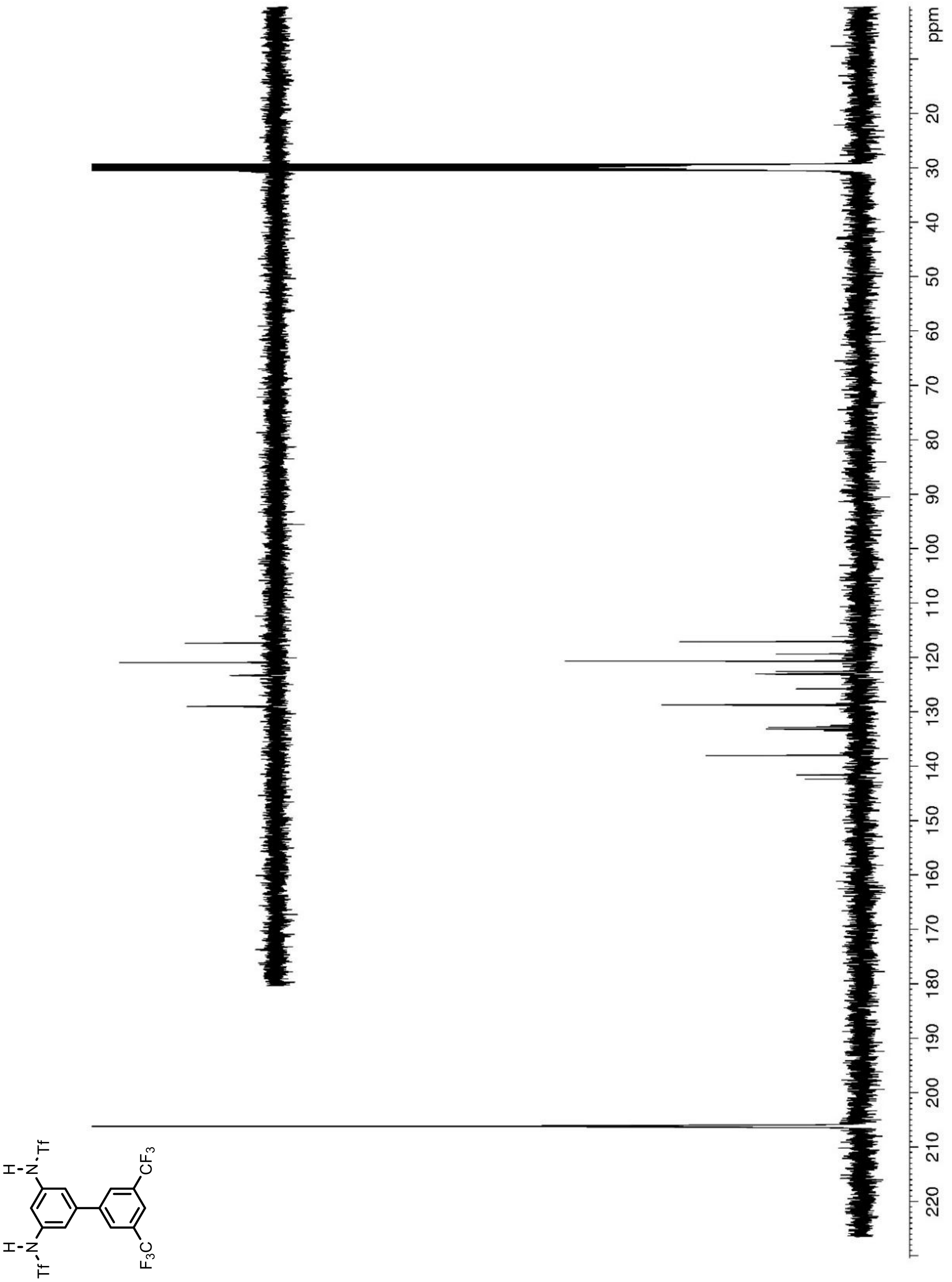

Figure 180. ^{19}F NMR (282 MHz, acetone-d_6) of **K5**

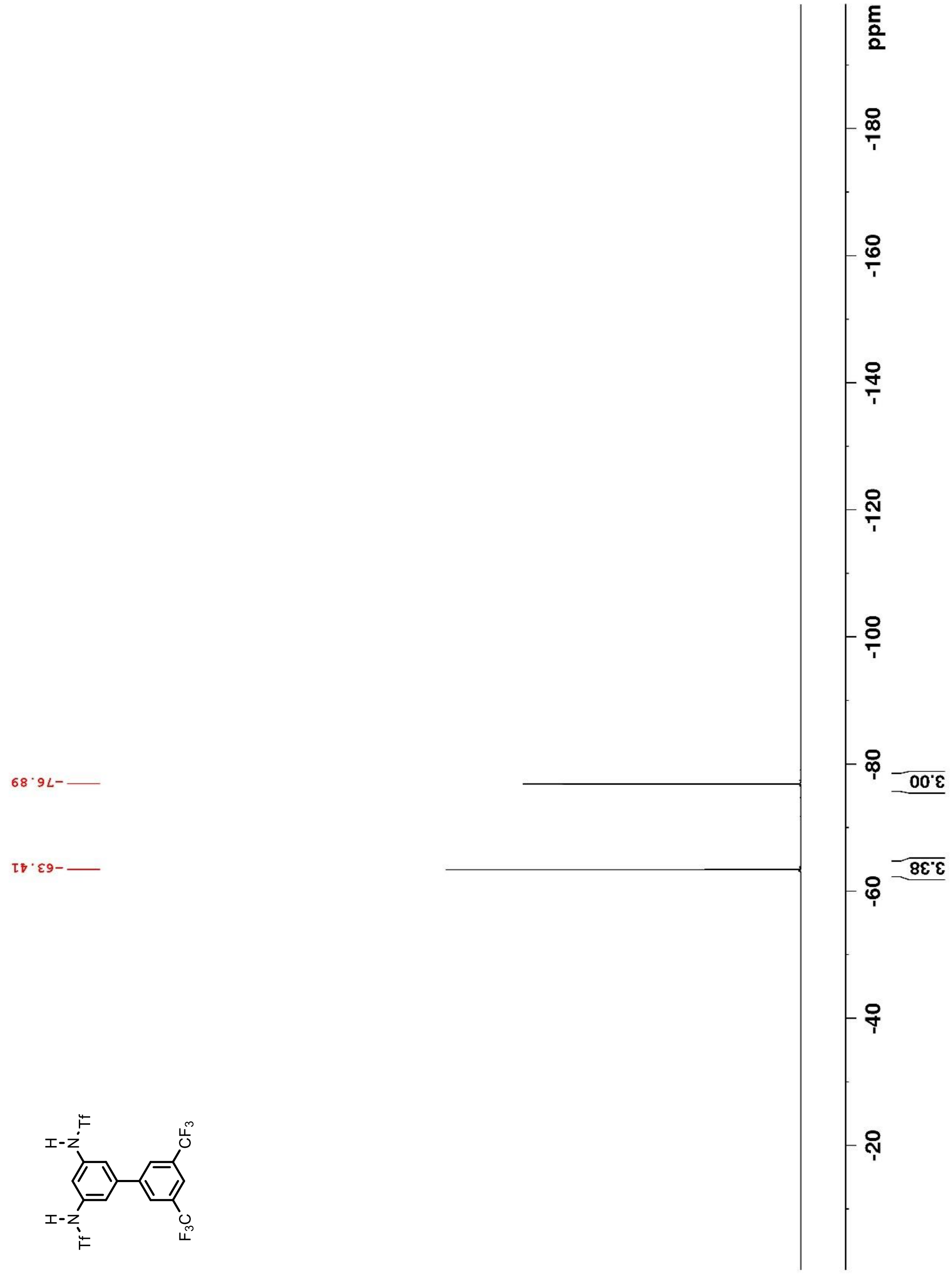

Figure 181. ^{1}H NMR (400 MHz, acetone-d_6) of **K6**

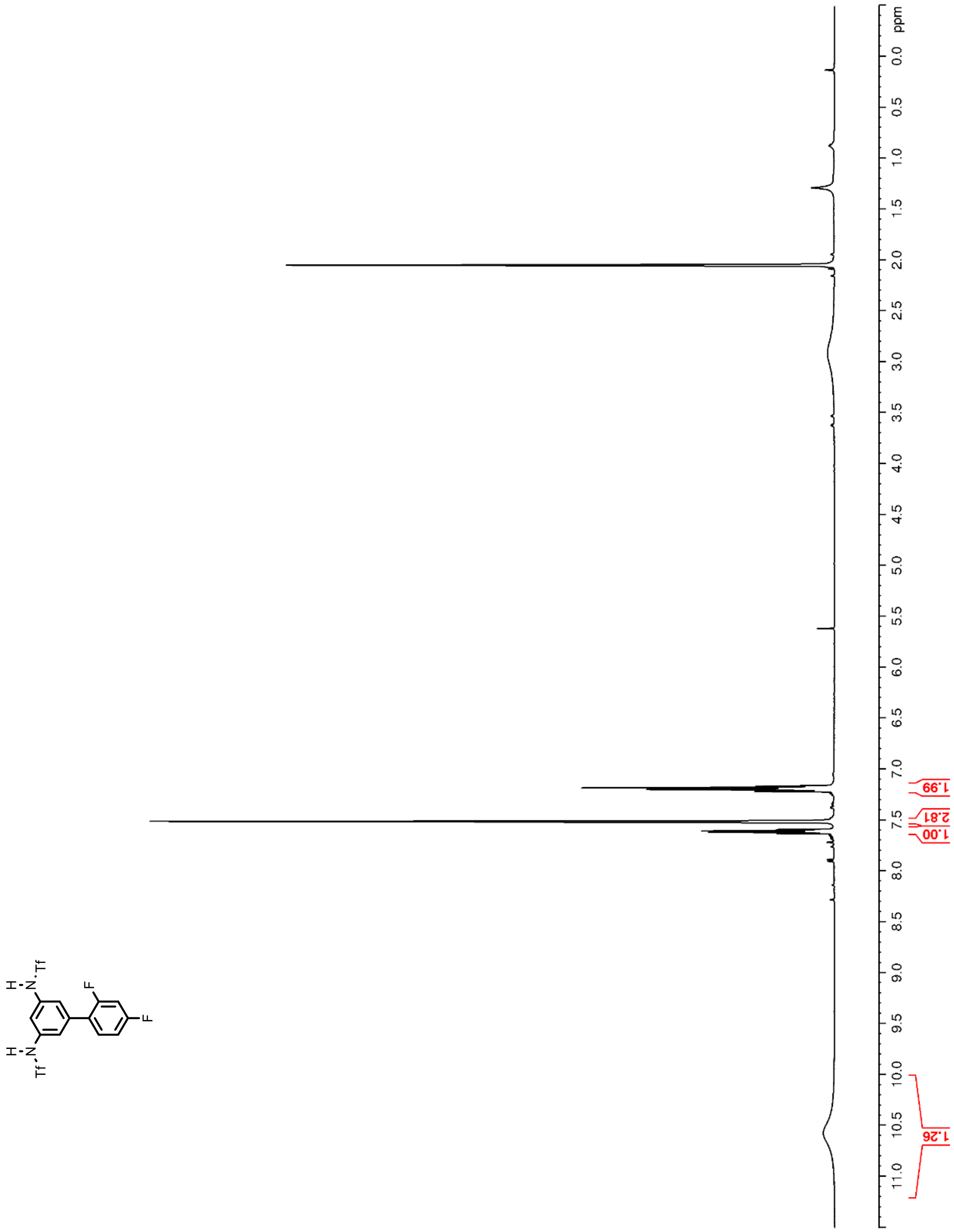

Figure 182. ^{13}C NMR (150 MHz, acetone-d_6) of **K6**

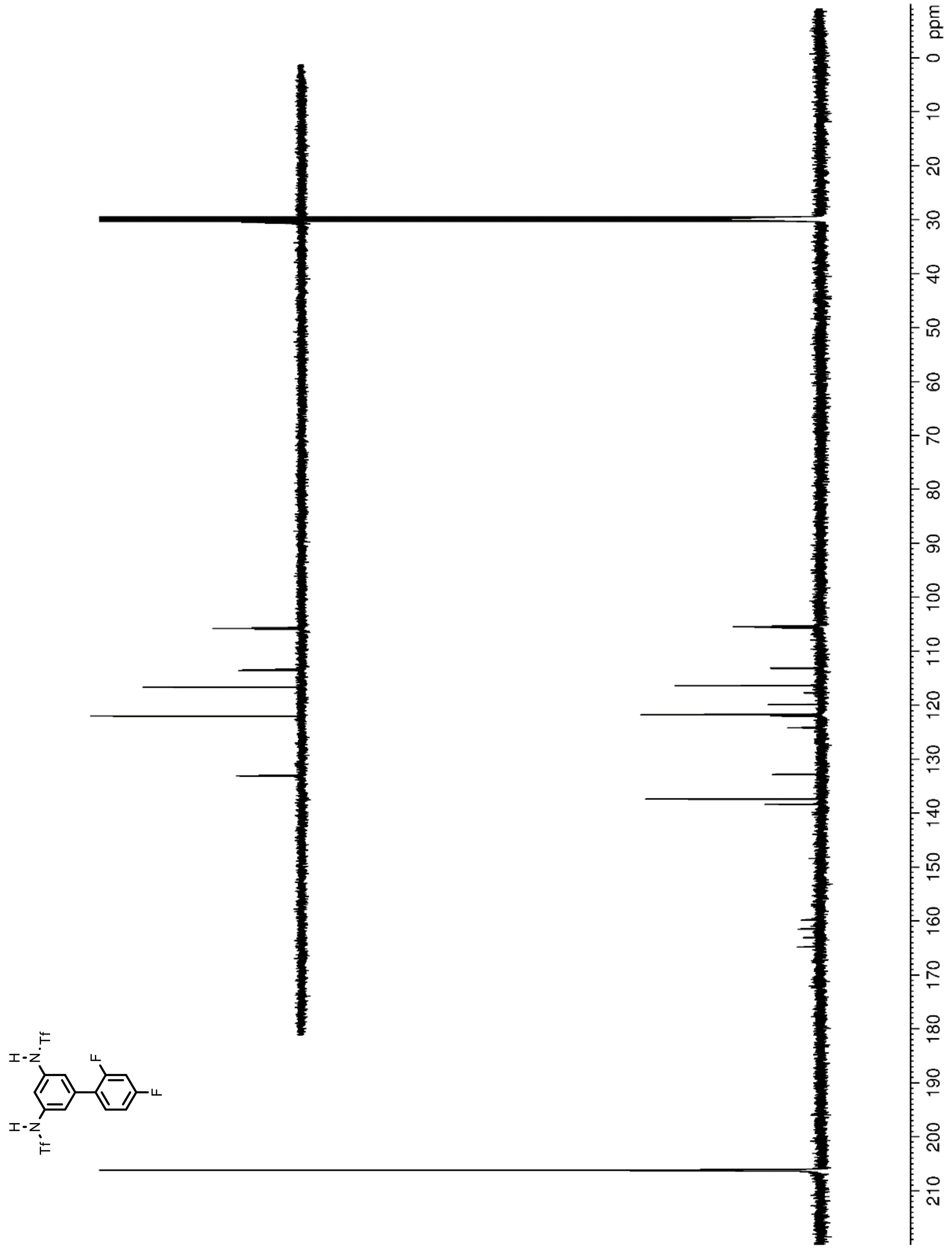

Figure 183. ^{19}F NMR (282 MHz, acetone-d_6) of **K6**

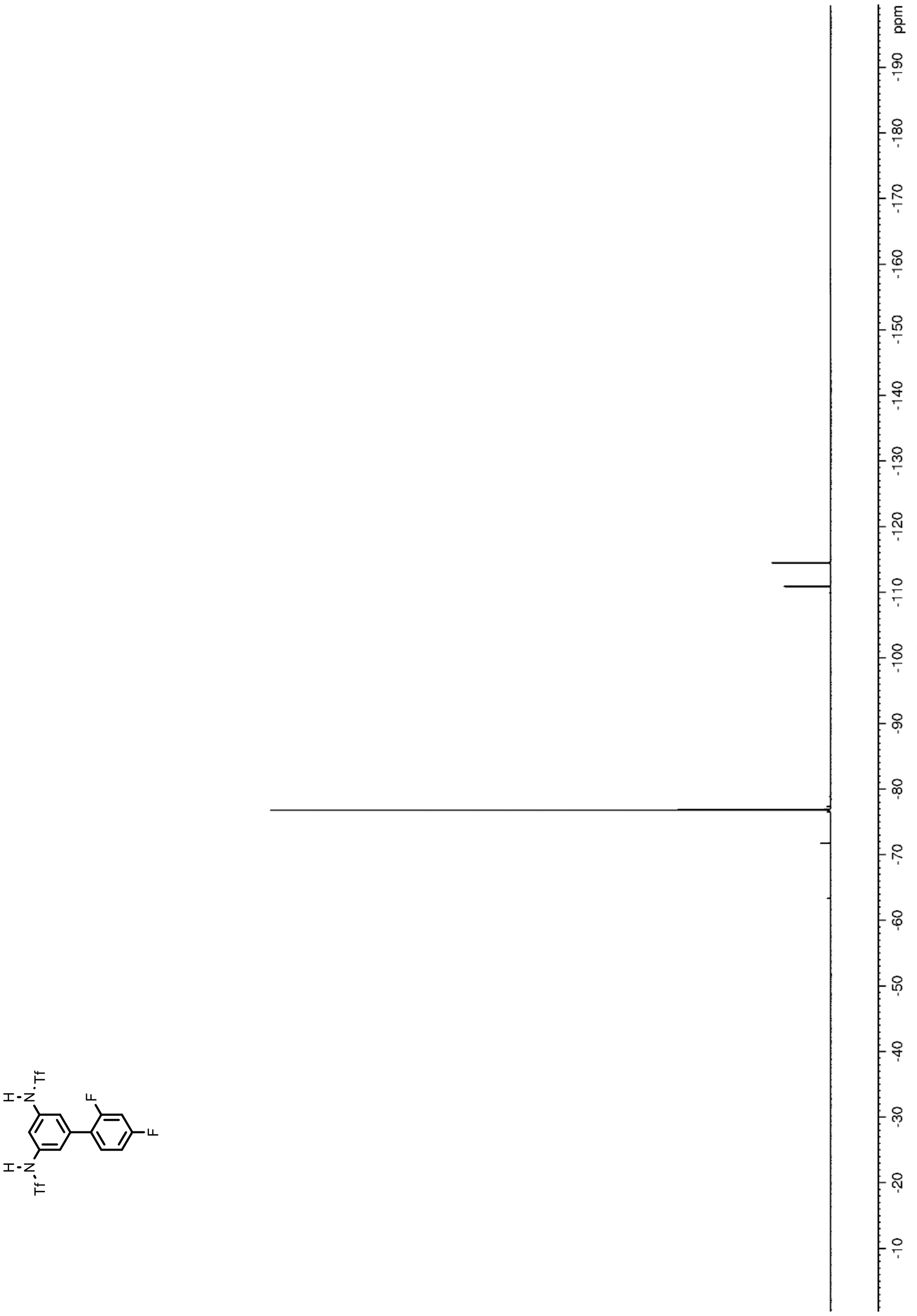

Figure 184. ^{1}H NMR (400 MHz, acetone-d_6) of **K7**

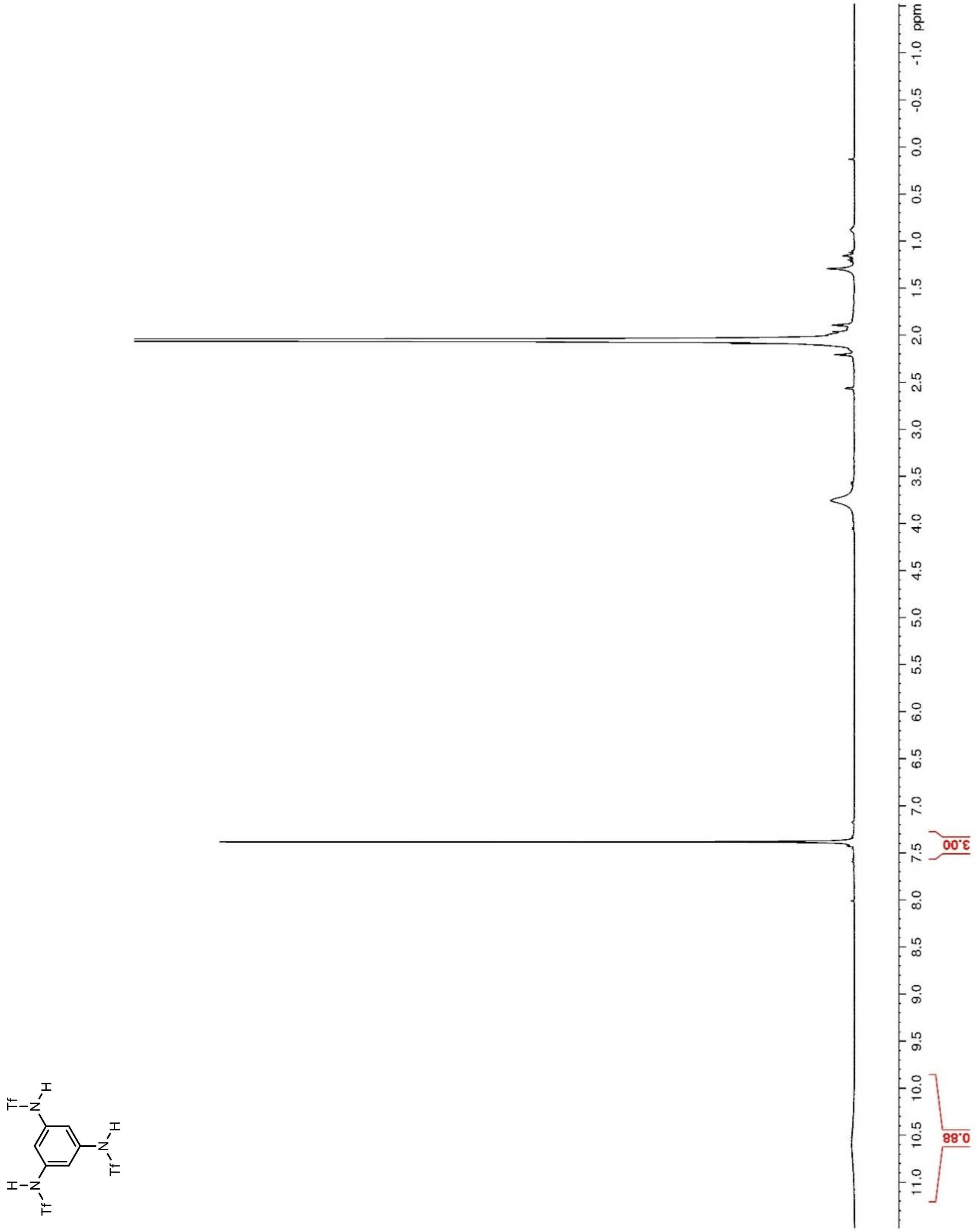

Figure 185. ^{13}C NMR (150 MHz, acetone-d_6) of **K7**

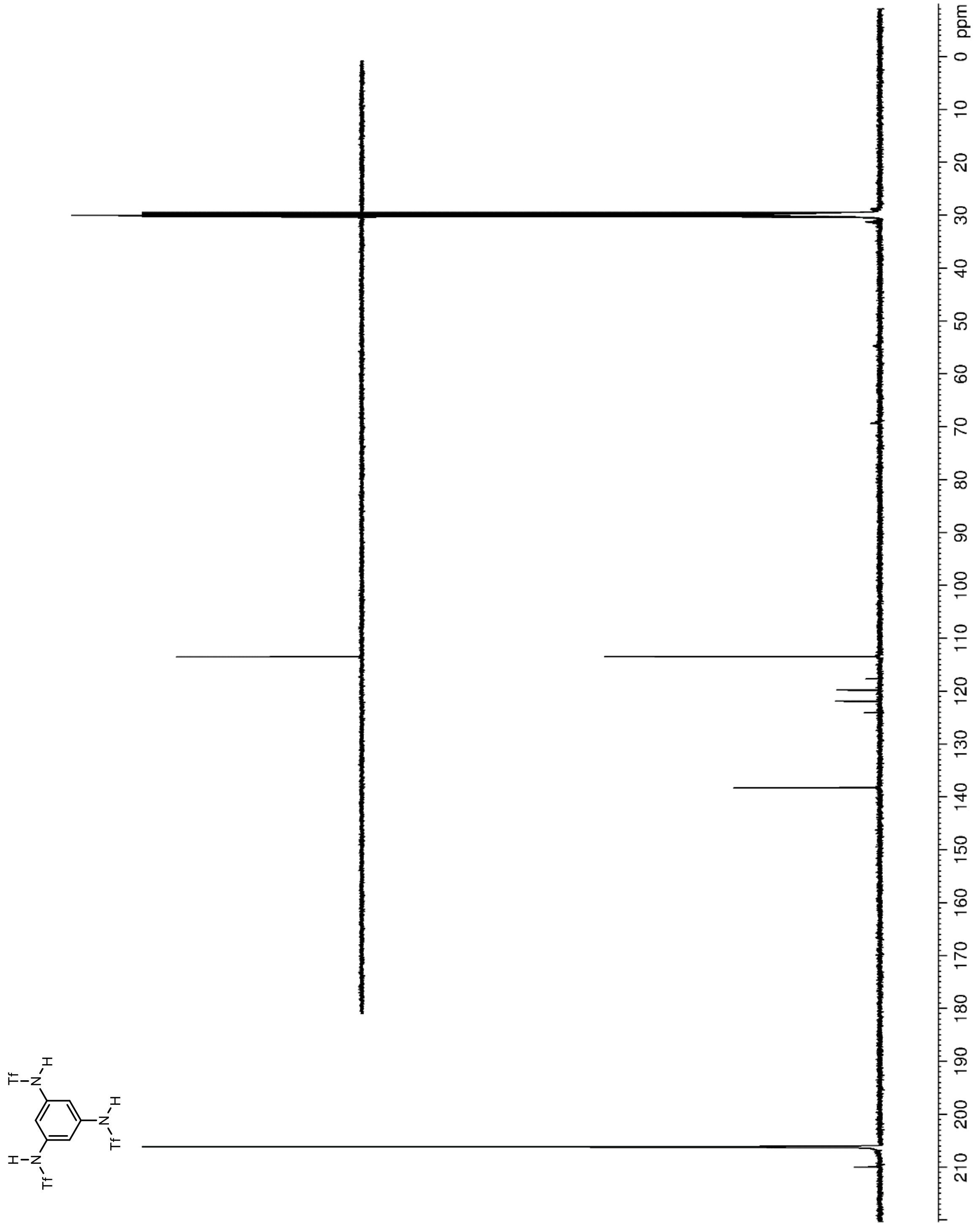

Figure 186. ^{19}F NMR (282 MHz, acetone-d_6) of **K7**

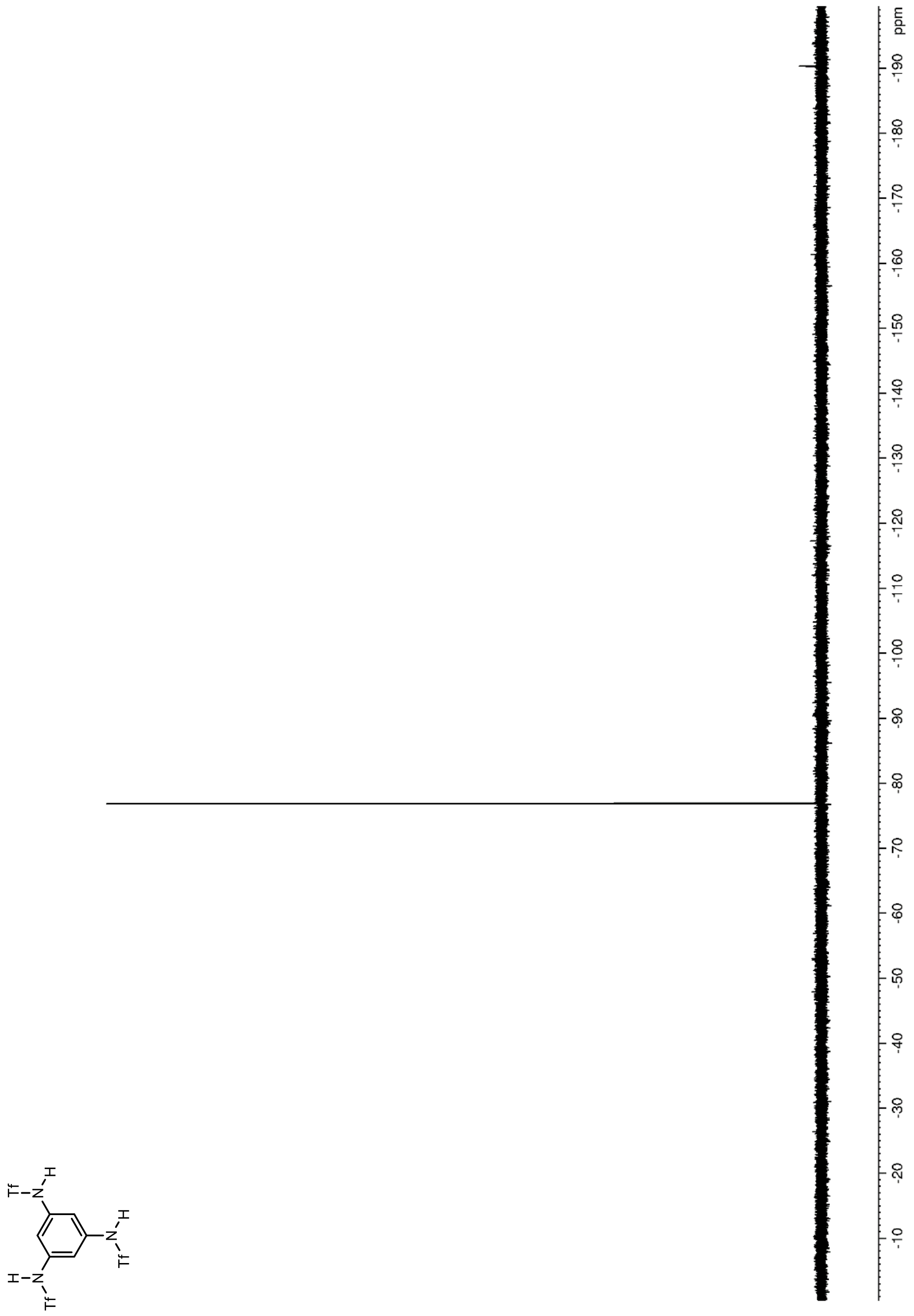

Figure 187. ^{1}H NMR (400 MHz, acetone-d_6) of **K8**

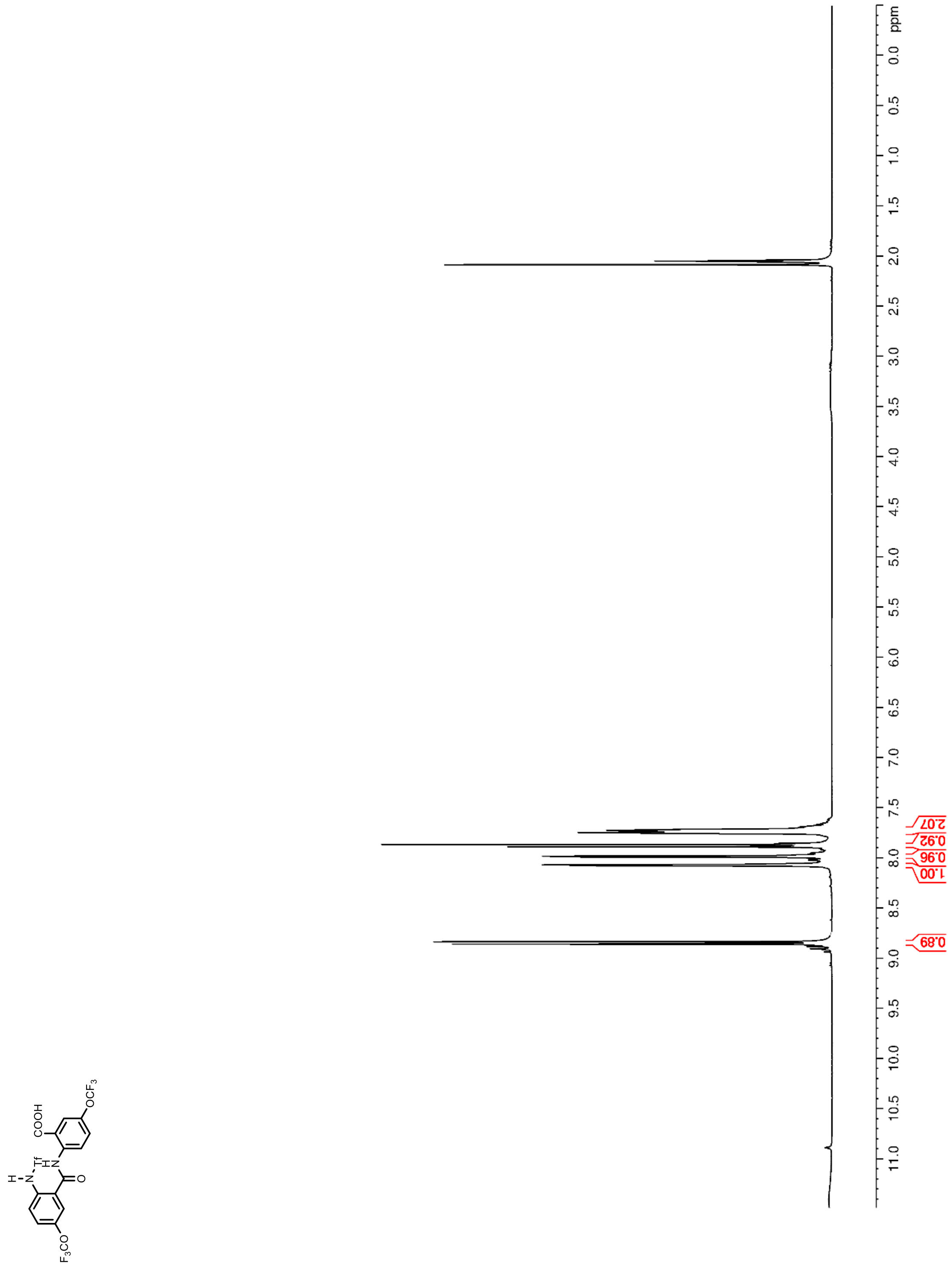

Figure 188. ^{13}C NMR (150 MHz, acetone-d_6) of **K8**

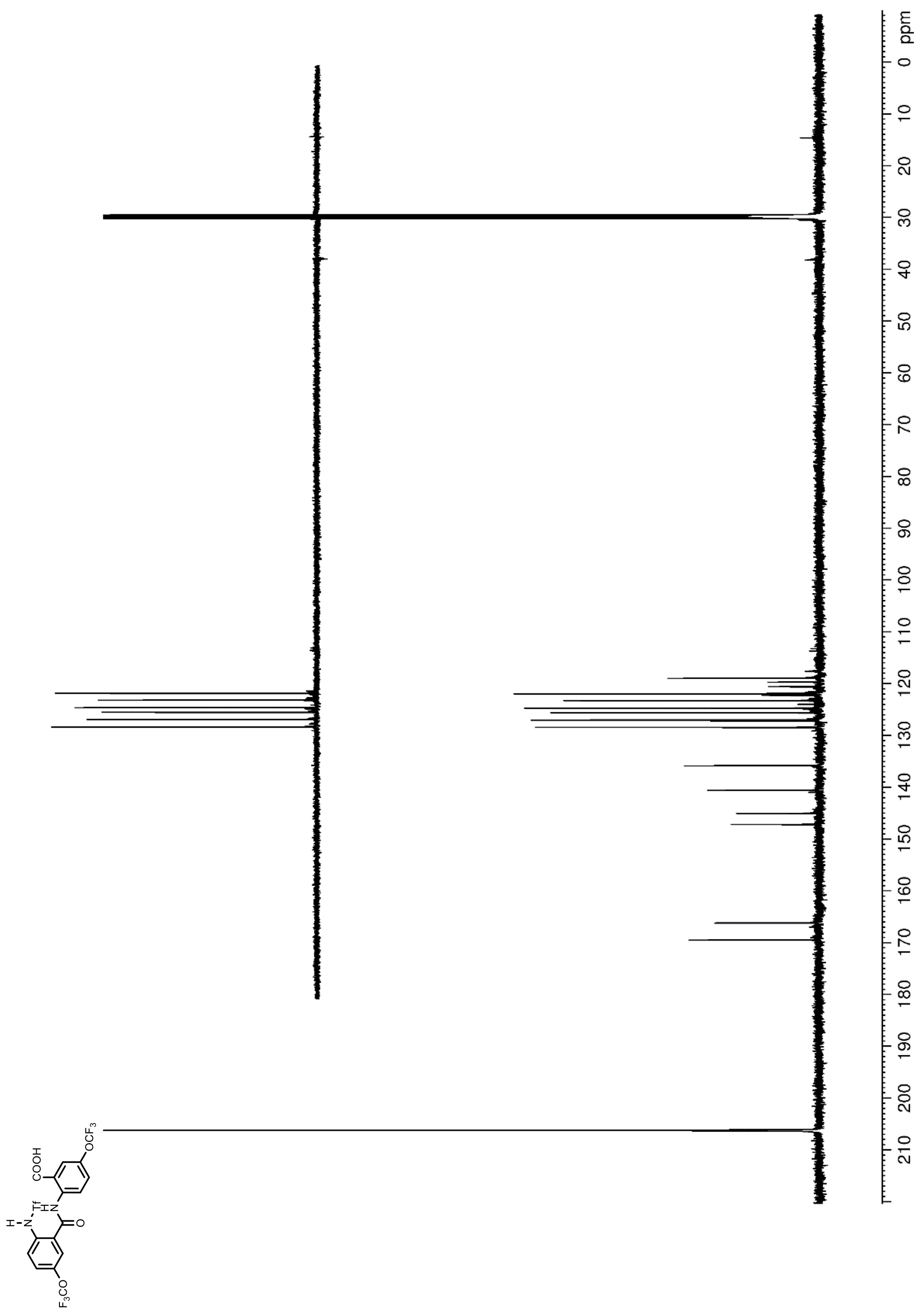

Figure 189. ^{19}F NMR (282 MHz, acetone-d_6) of **K8**

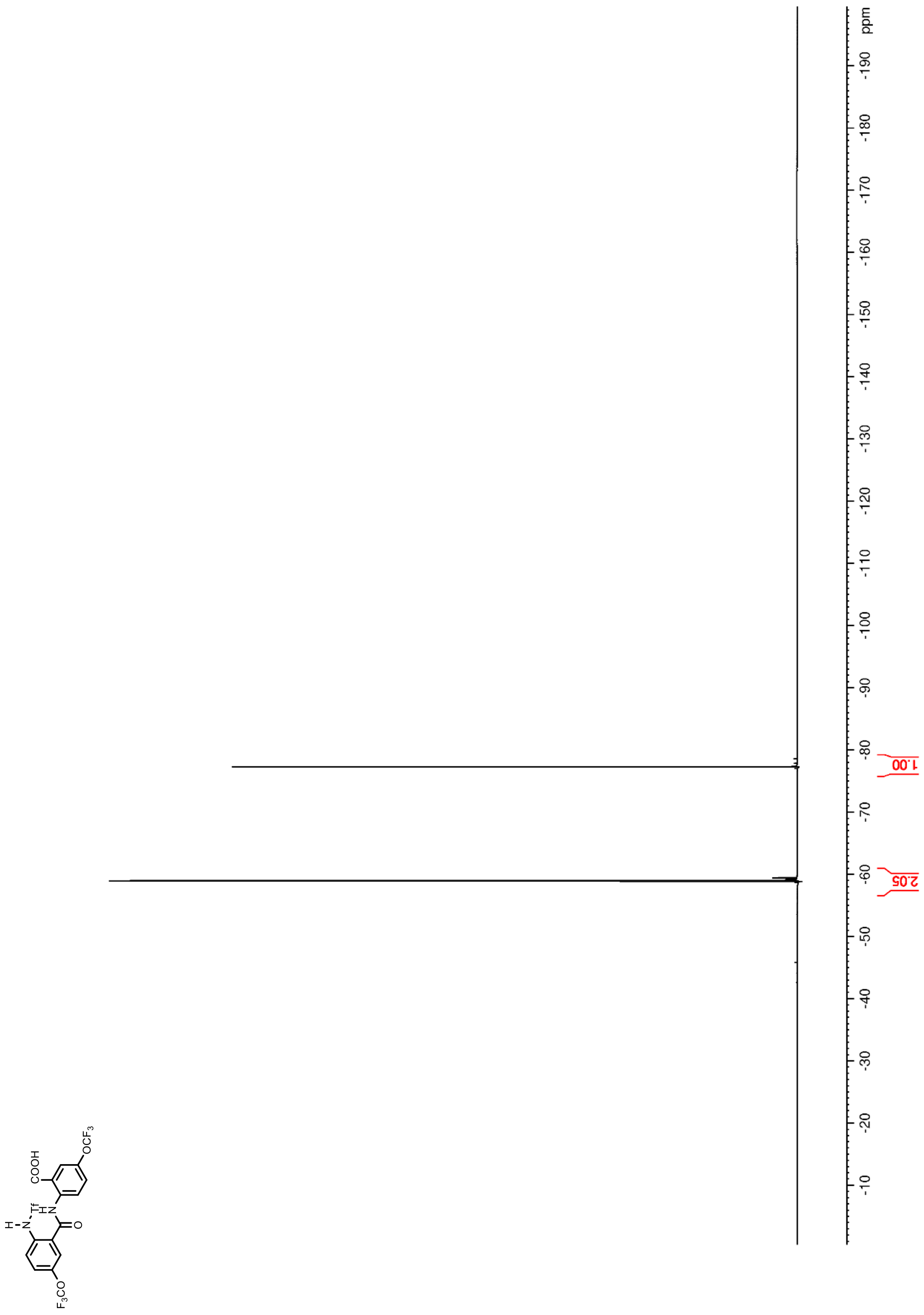

Figure 190. ^{1}H NMR (400 MHz, $CDCl_3$) of **L2**

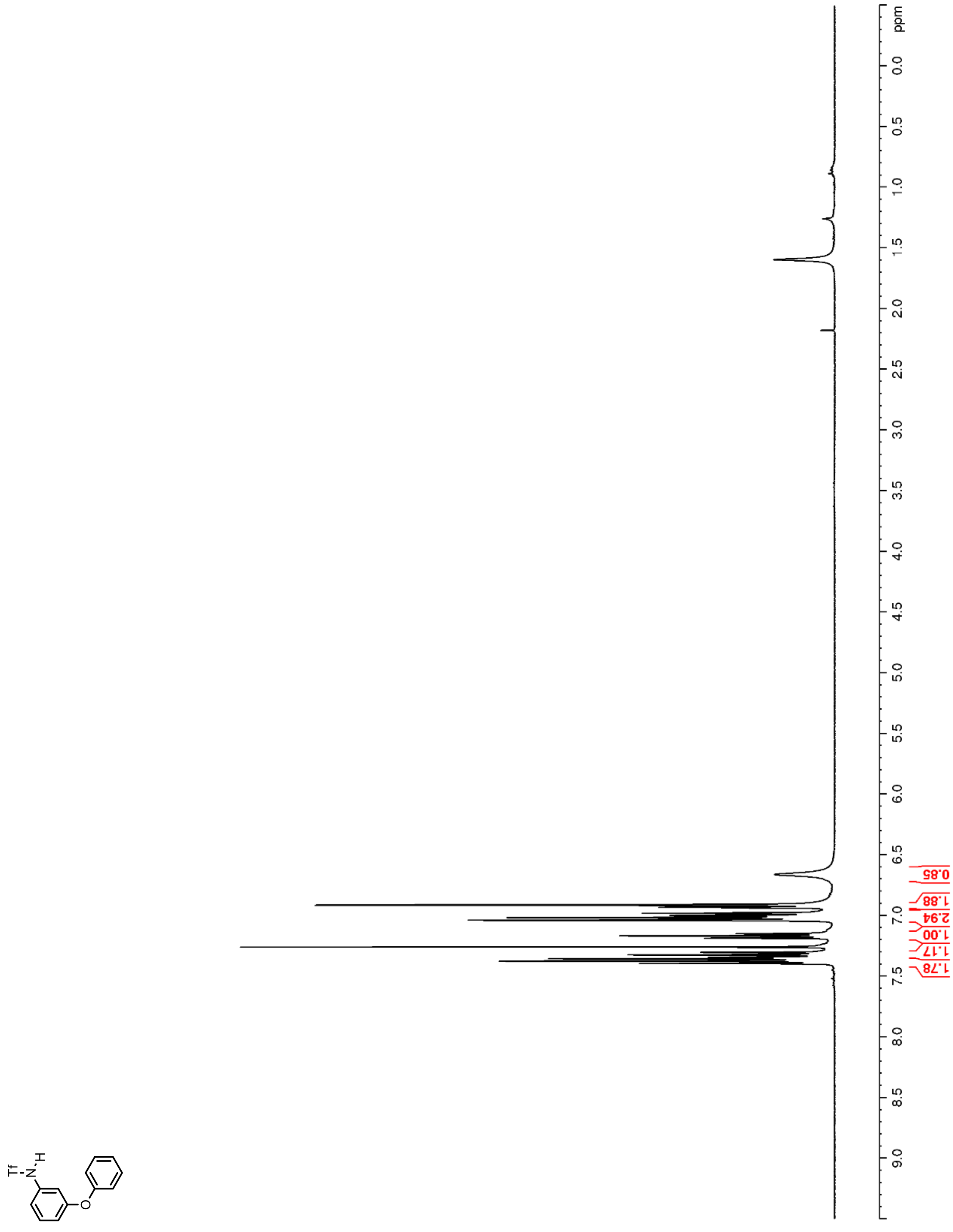

Figure 191. ^{13}C NMR (150 MHz, $CDCl_3$) of **L2**

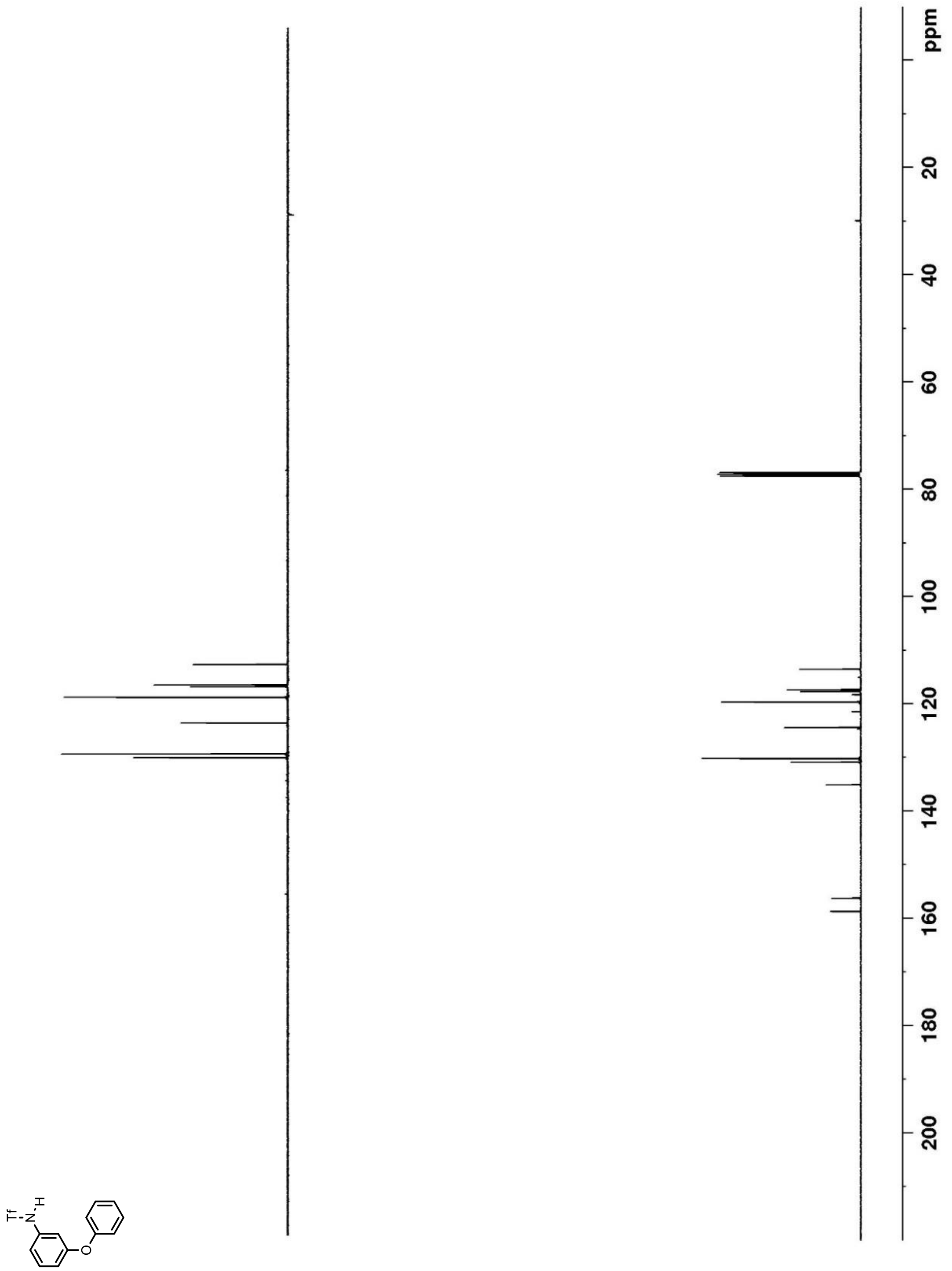

Figure 192. ^{19}F NMR (282 MHz, $CDCl_3$) of **L2**

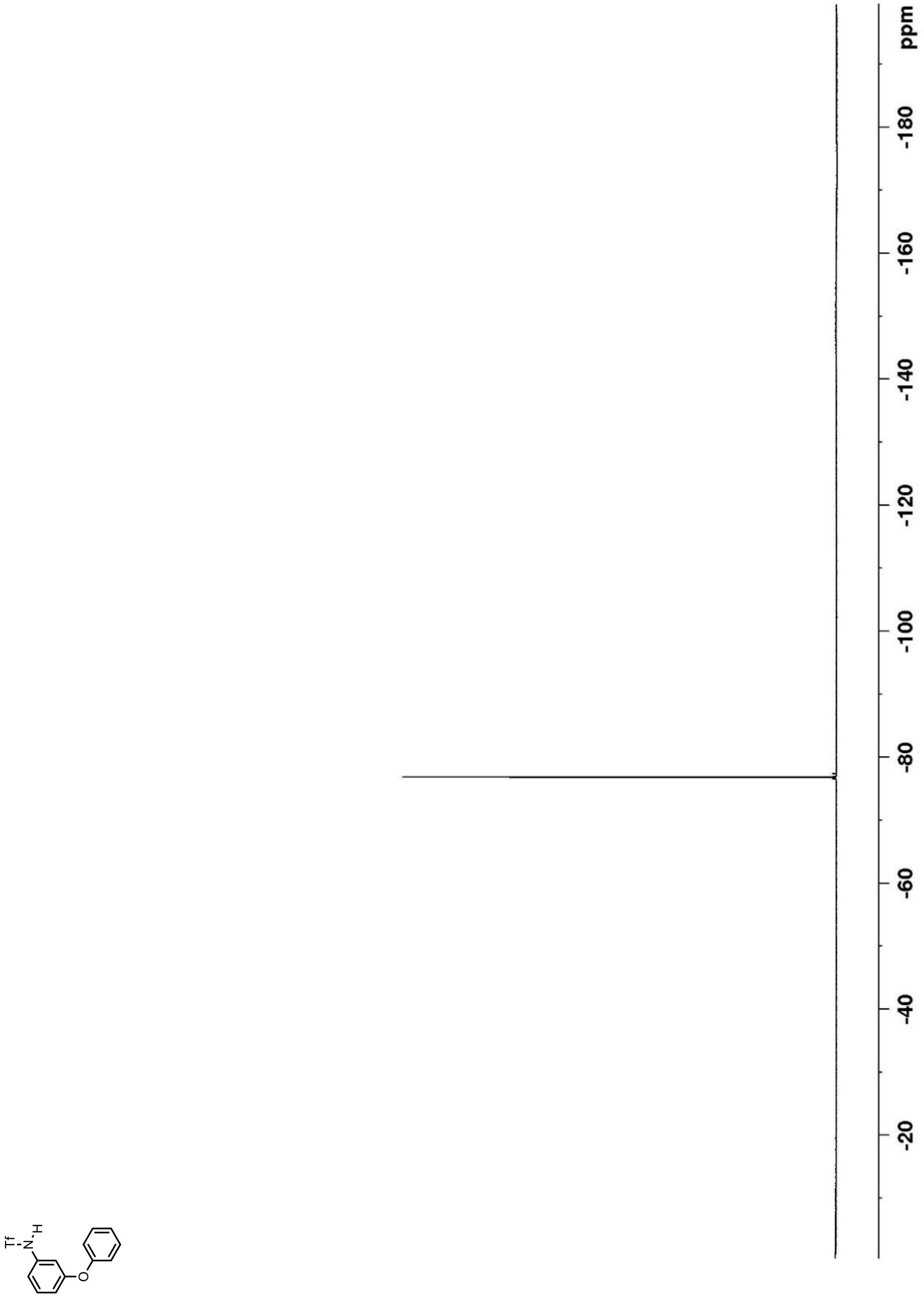

Figure 193. ^{1}H NMR (400 MHz, $CDCl_3$) of **L3**

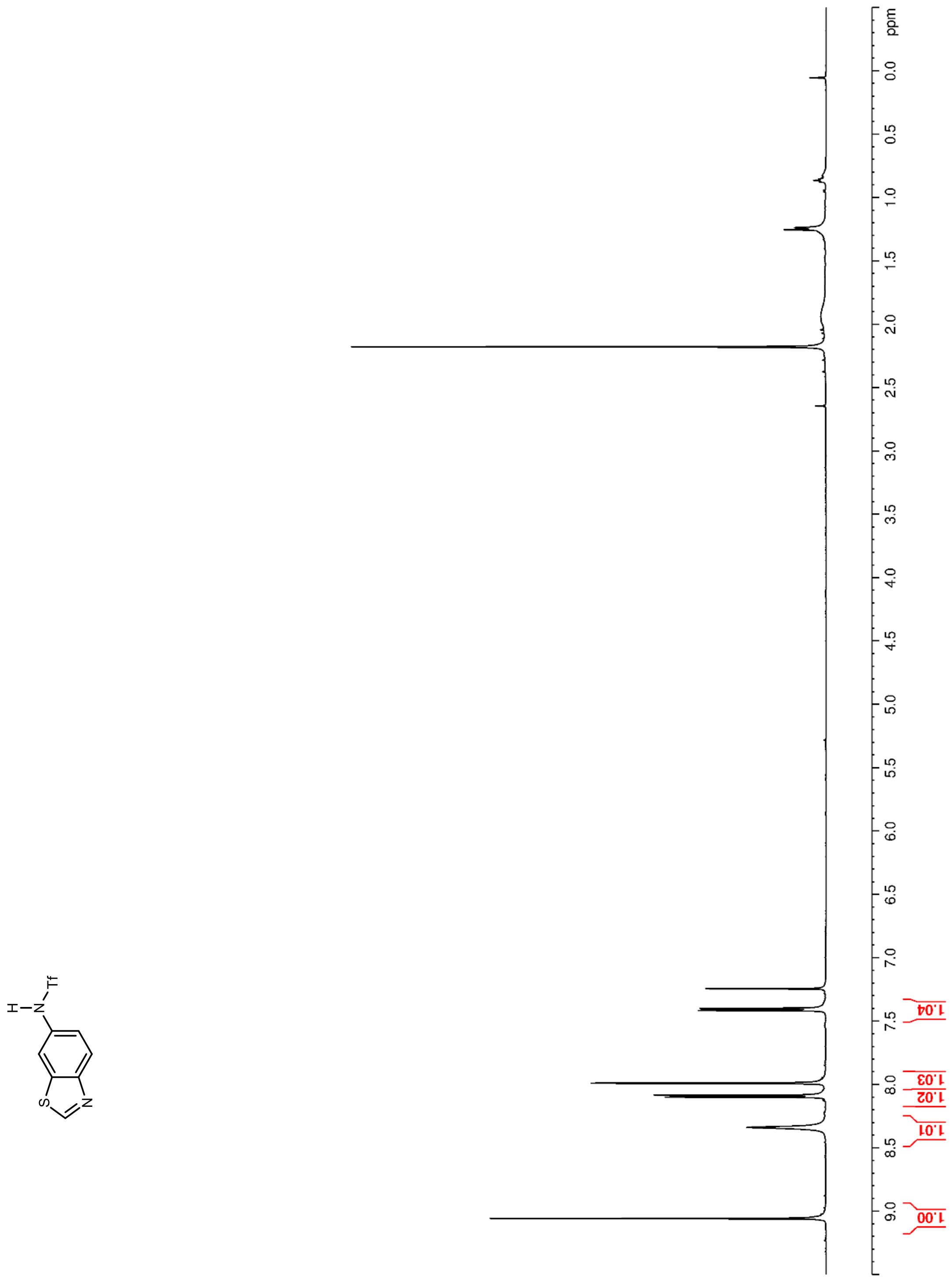

Figure 194. ^{13}C NMR (150 MHz, $CDCl_3$) of **L3**

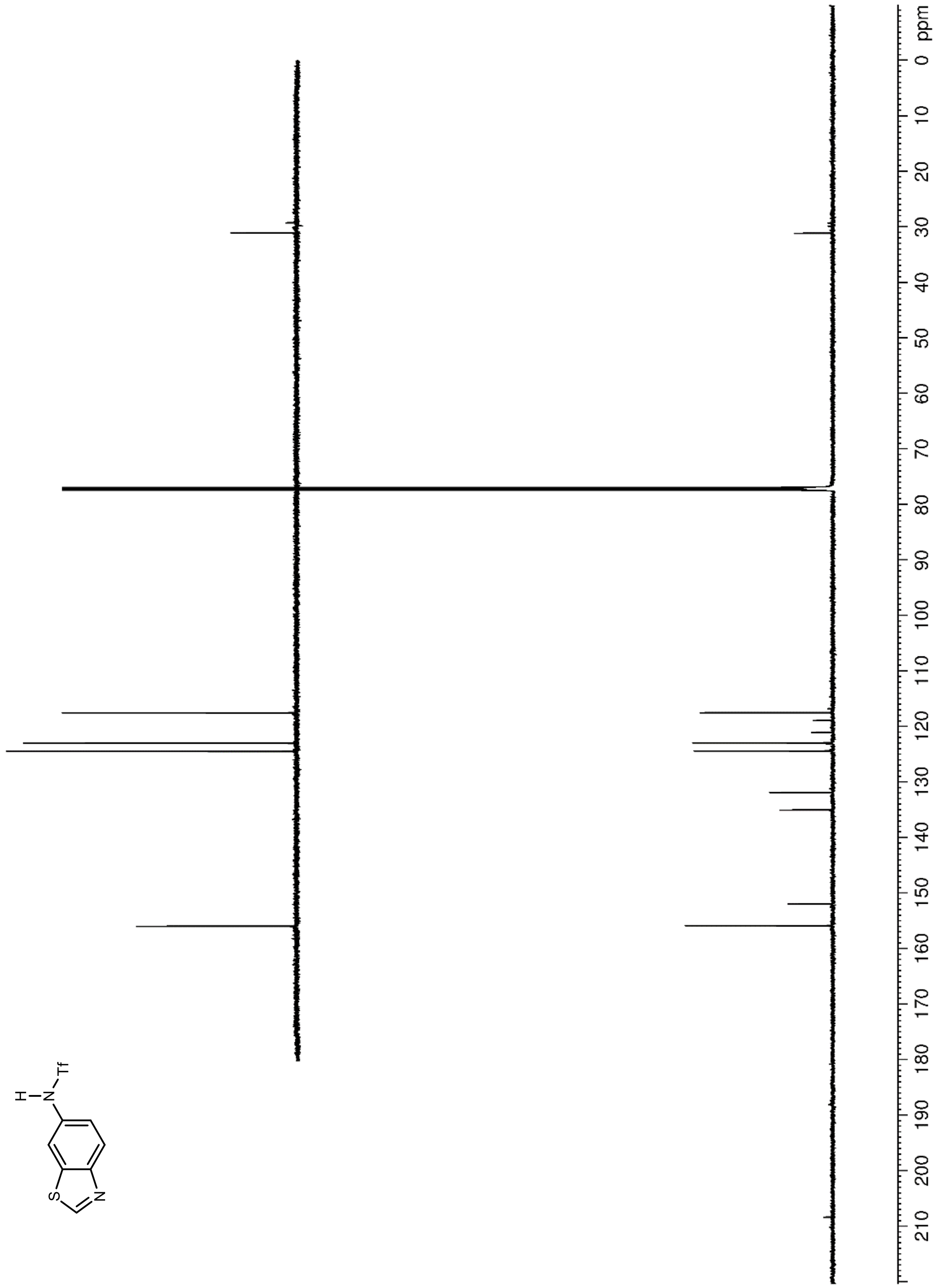

Figure 195. ^{19}F NMR (282 MHz, $CDCl_3$) of **L3**

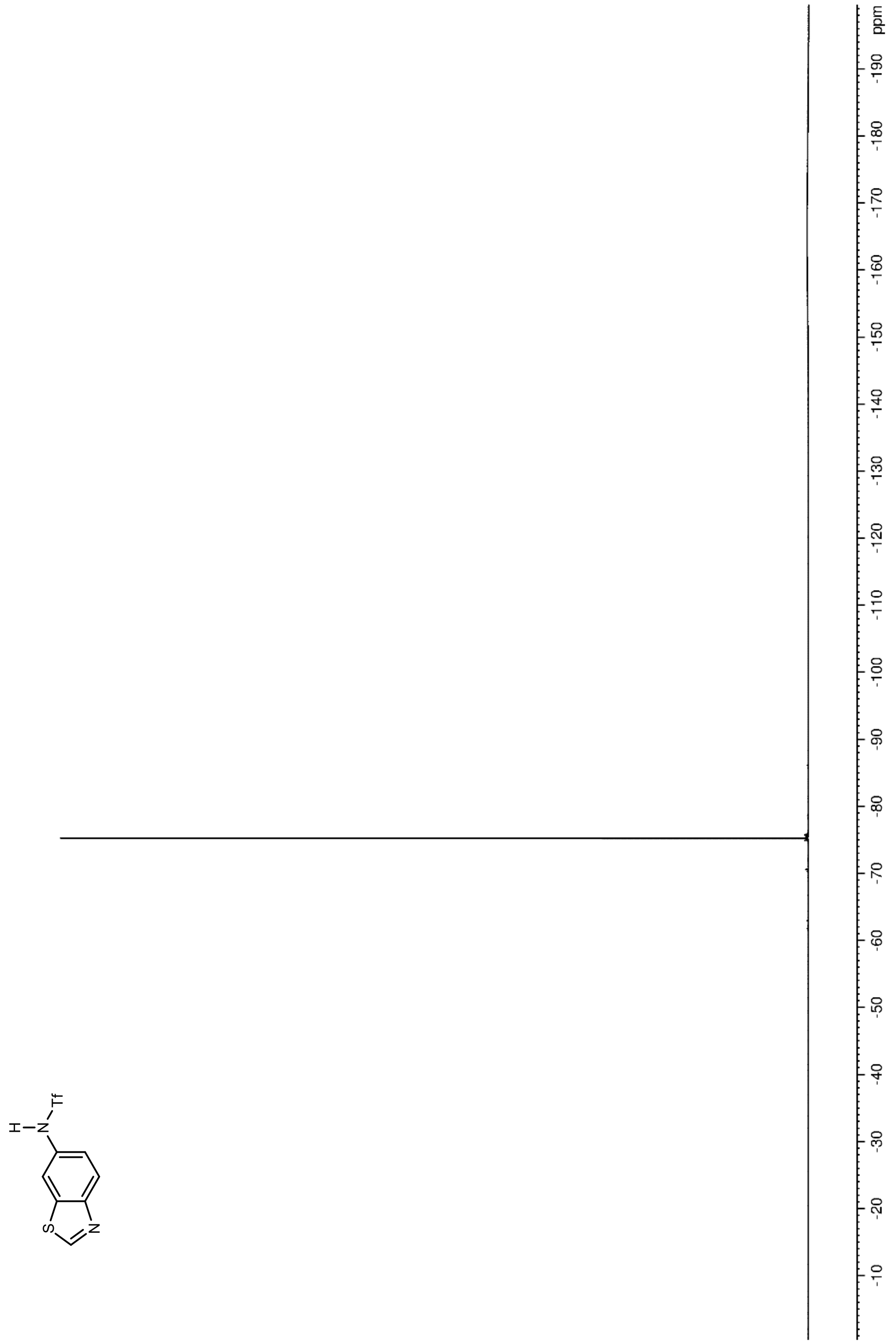

Figure 196. ^{1}H NMR (400 MHz, acetone-d_6) of **L4**

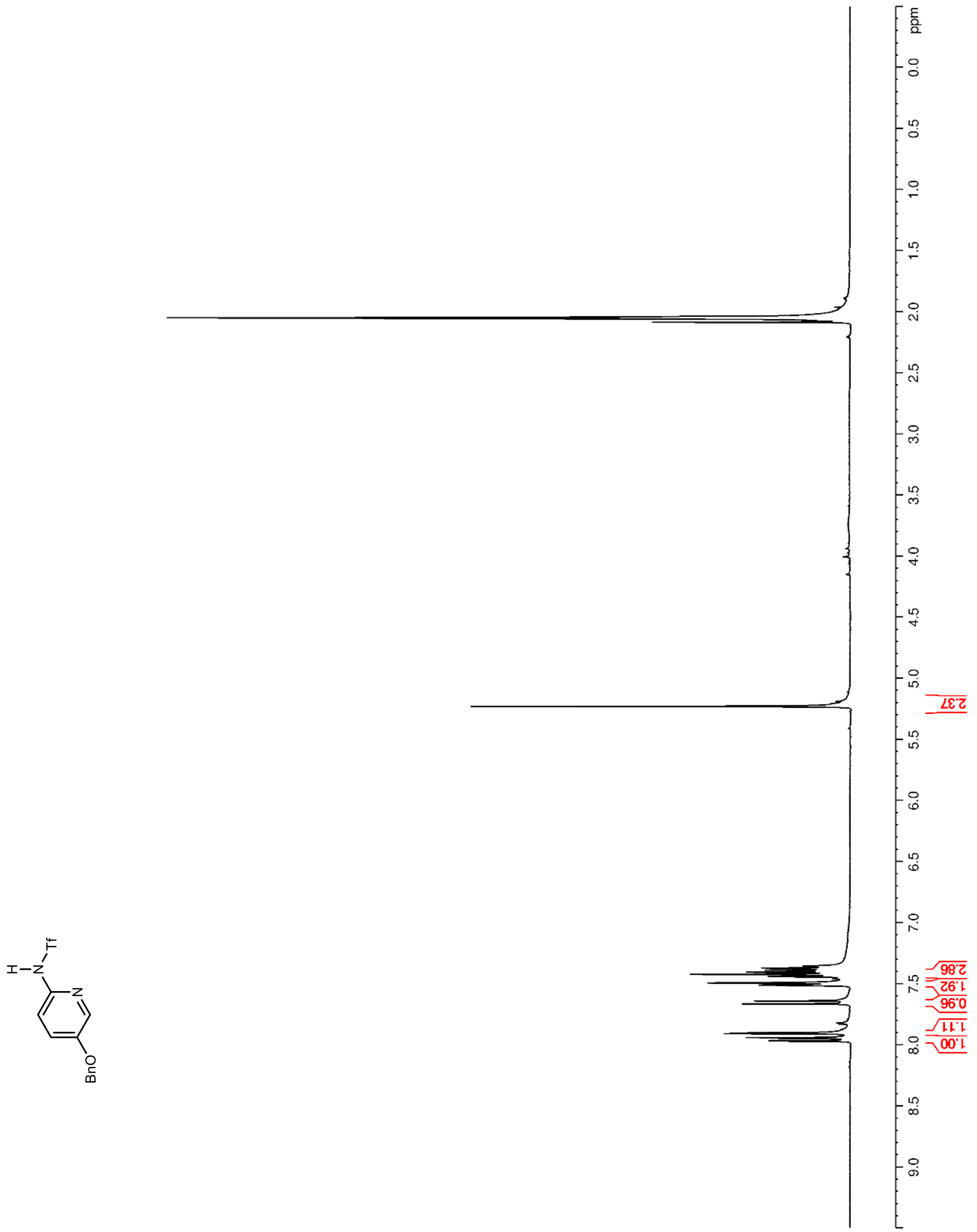

Figure 197. ^{13}C NMR (150 MHz, acetone-d_6) of **L4**

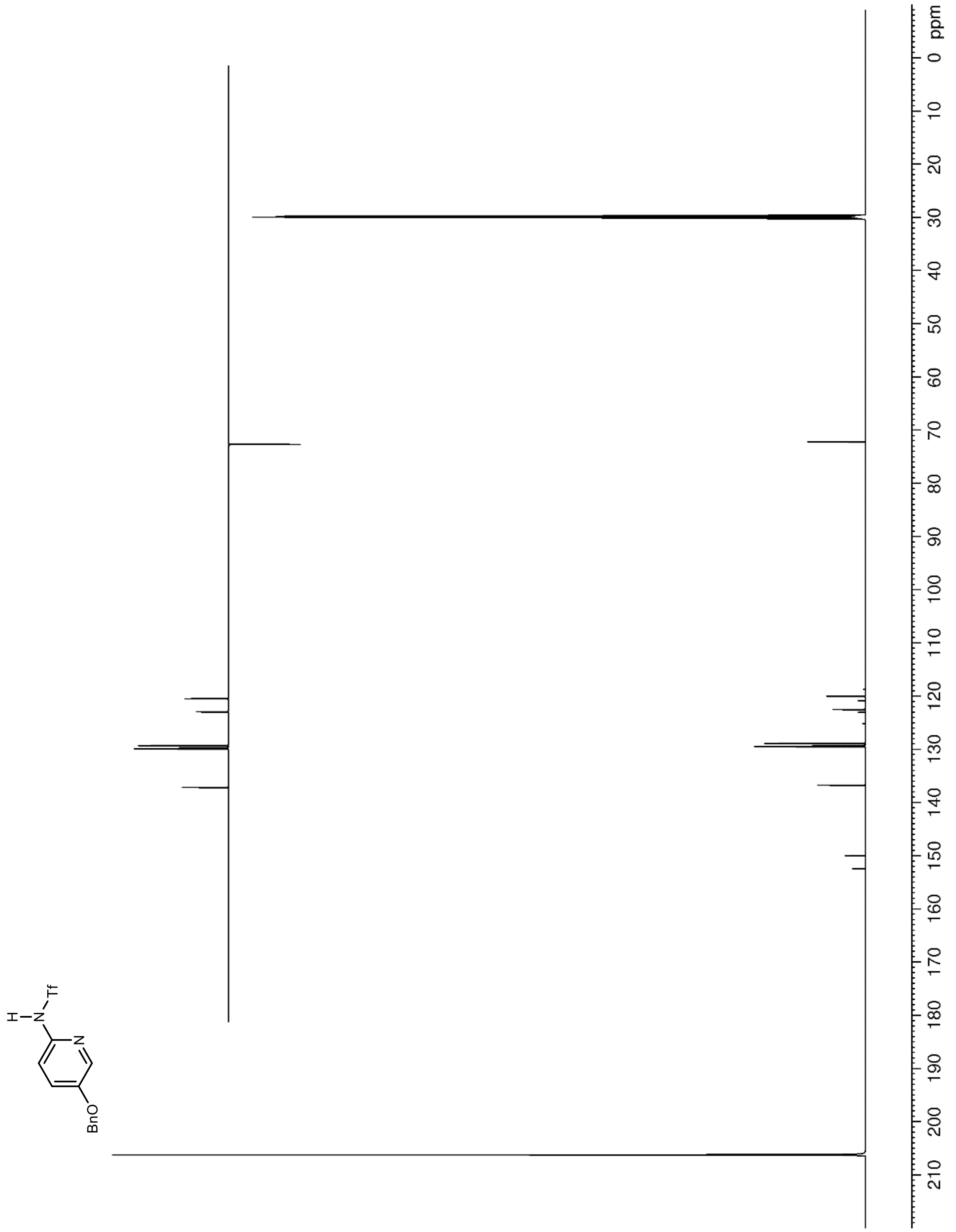

Figure 198. ^{19}F NMR (282 MHz, acetone-d_6) of **L4**

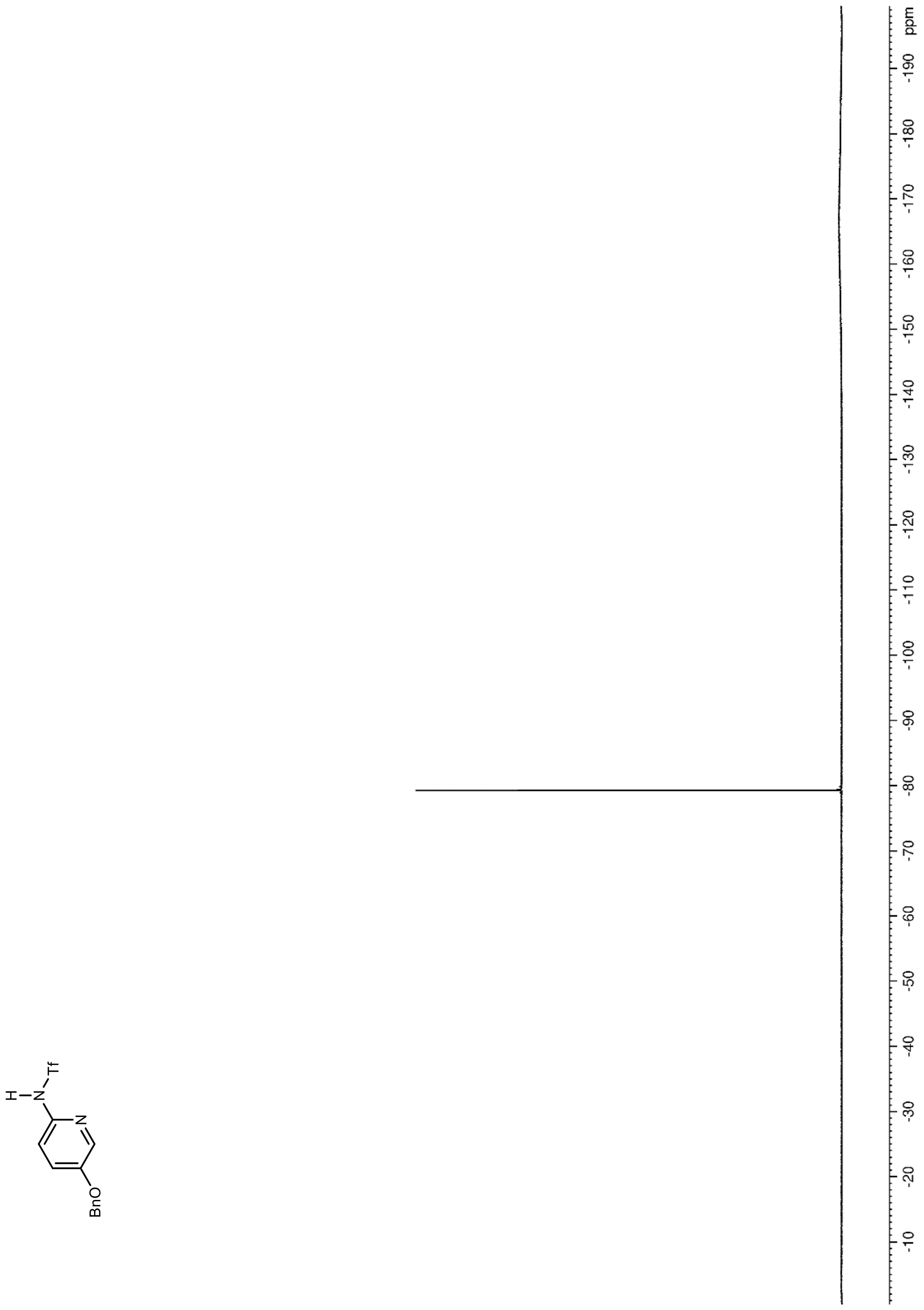

Figure 199. ^{1}H NMR (400 MHz, $CDCl_3$) of **L7**

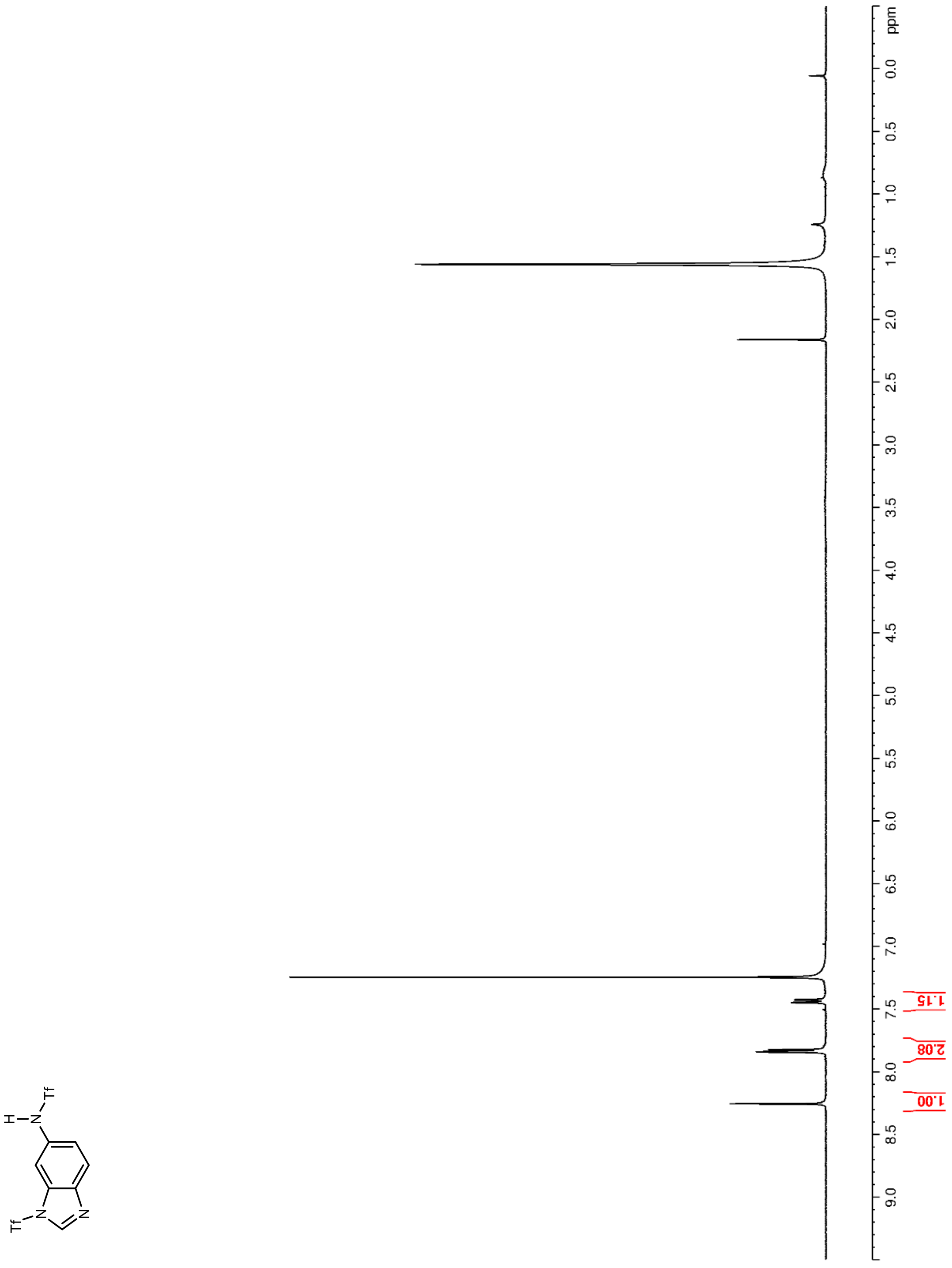

Figure 200. ^{13}C NMR (150 MHz, acetone-d_6) of **L7**

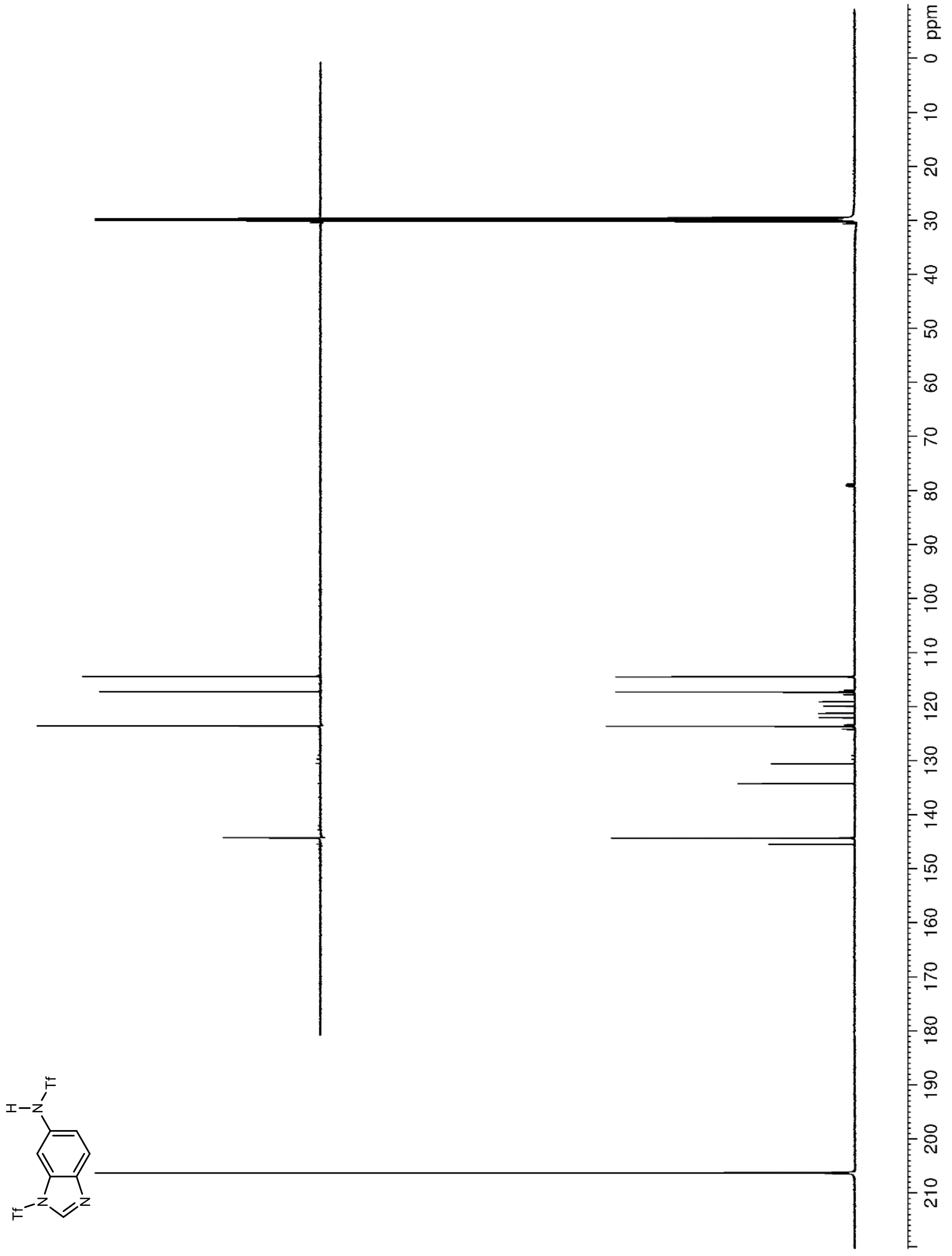

Figure 201. ^{19}F NMR (282 MHz, $CDCl_3$) of **L7**

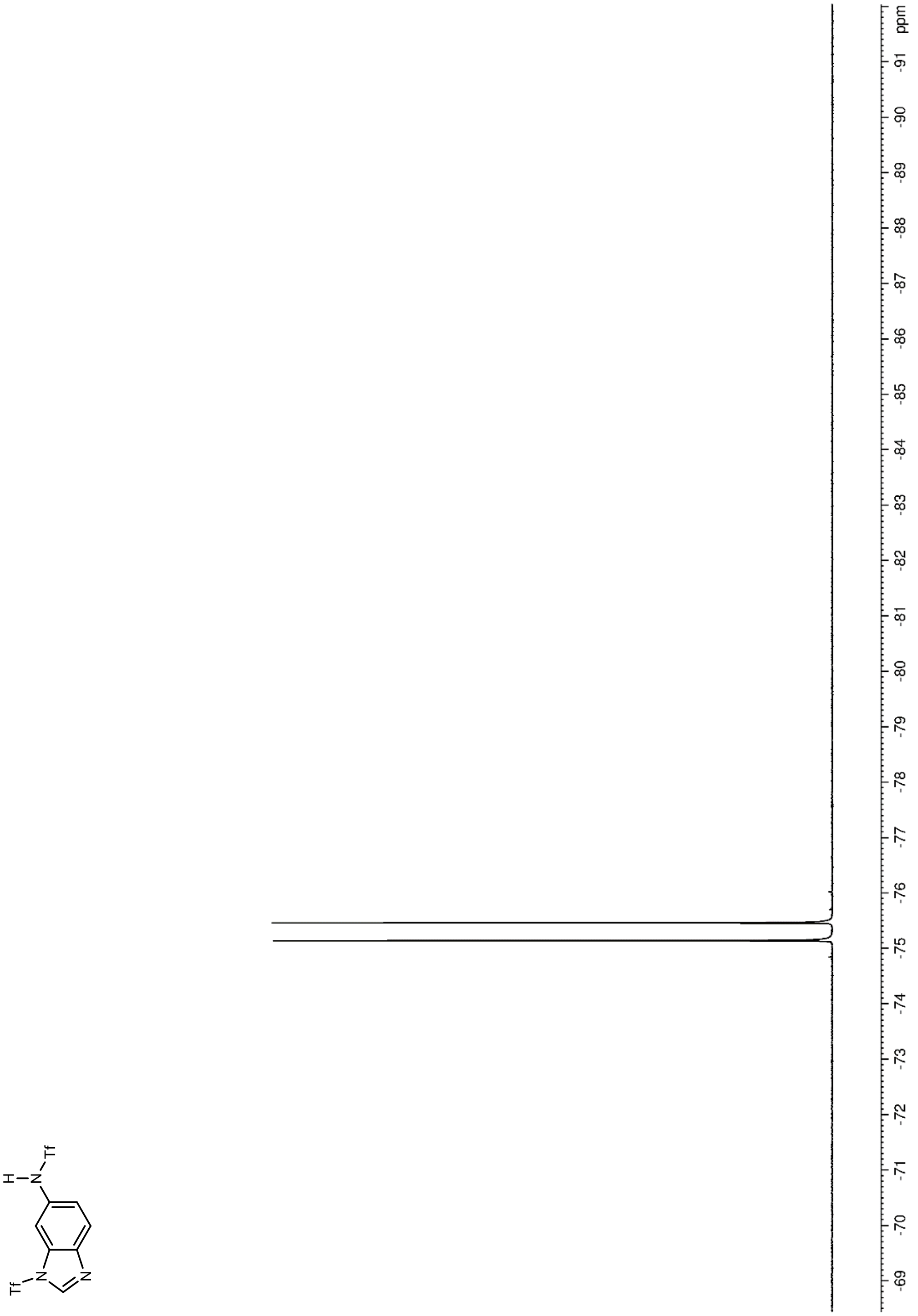

Figure 202. ^{1}H NMR (400 MHz, CD_3OD) of **L8**

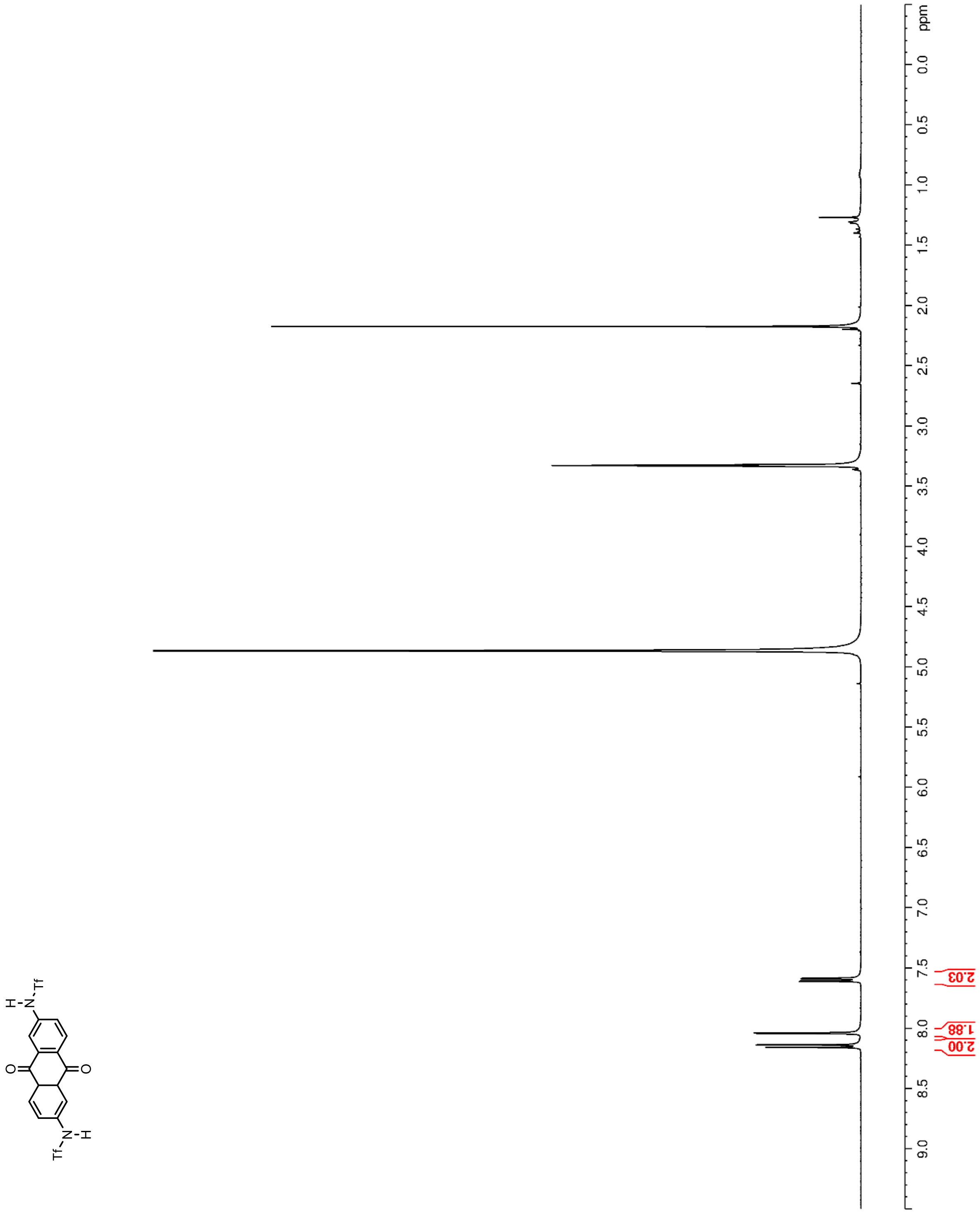

Figure 203. ^{13}C NMR (150 MHz, acetone-d_6) of **L8**

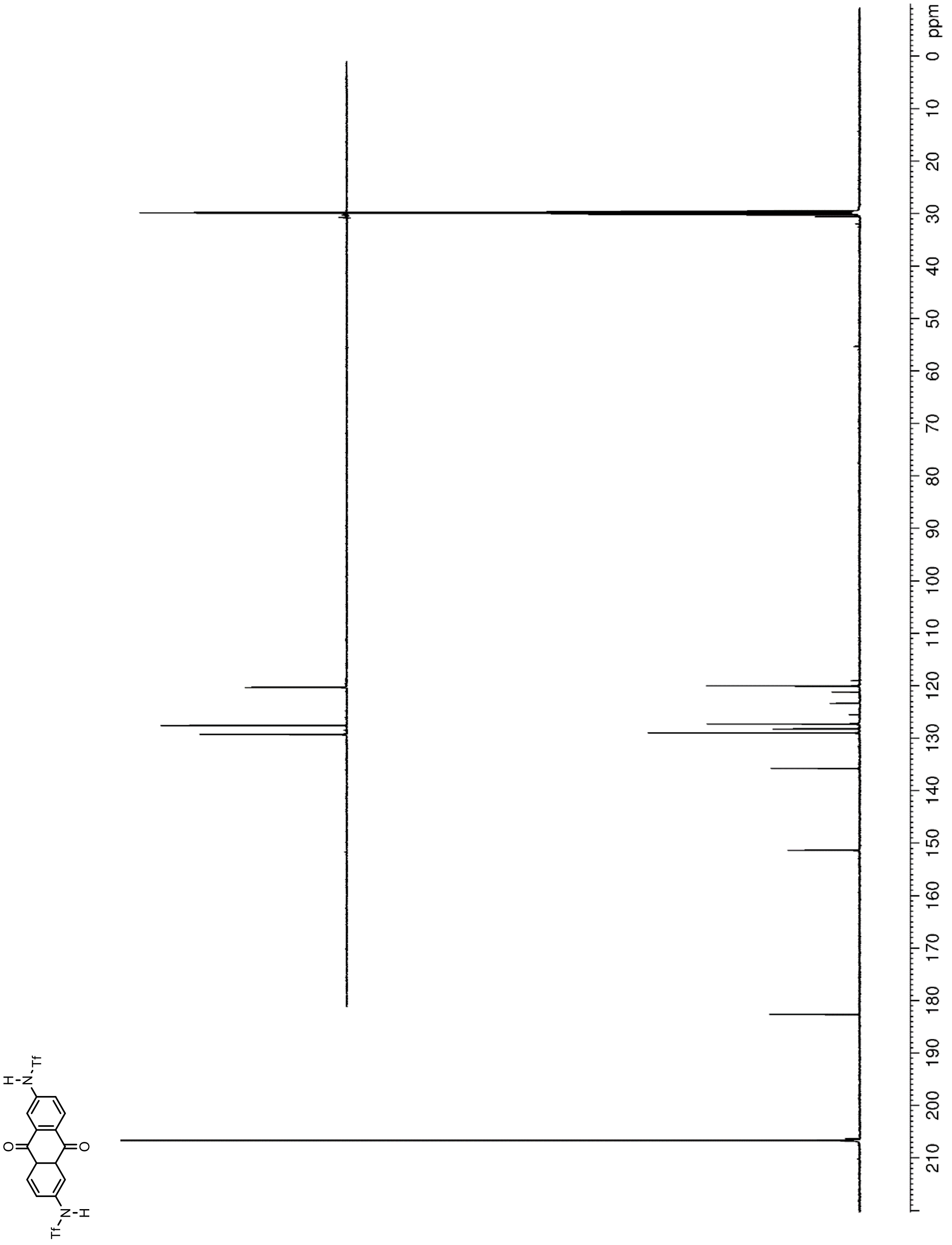

Figure 204. ^{19}F NMR (282 MHz, CD_3OD) of **L8**

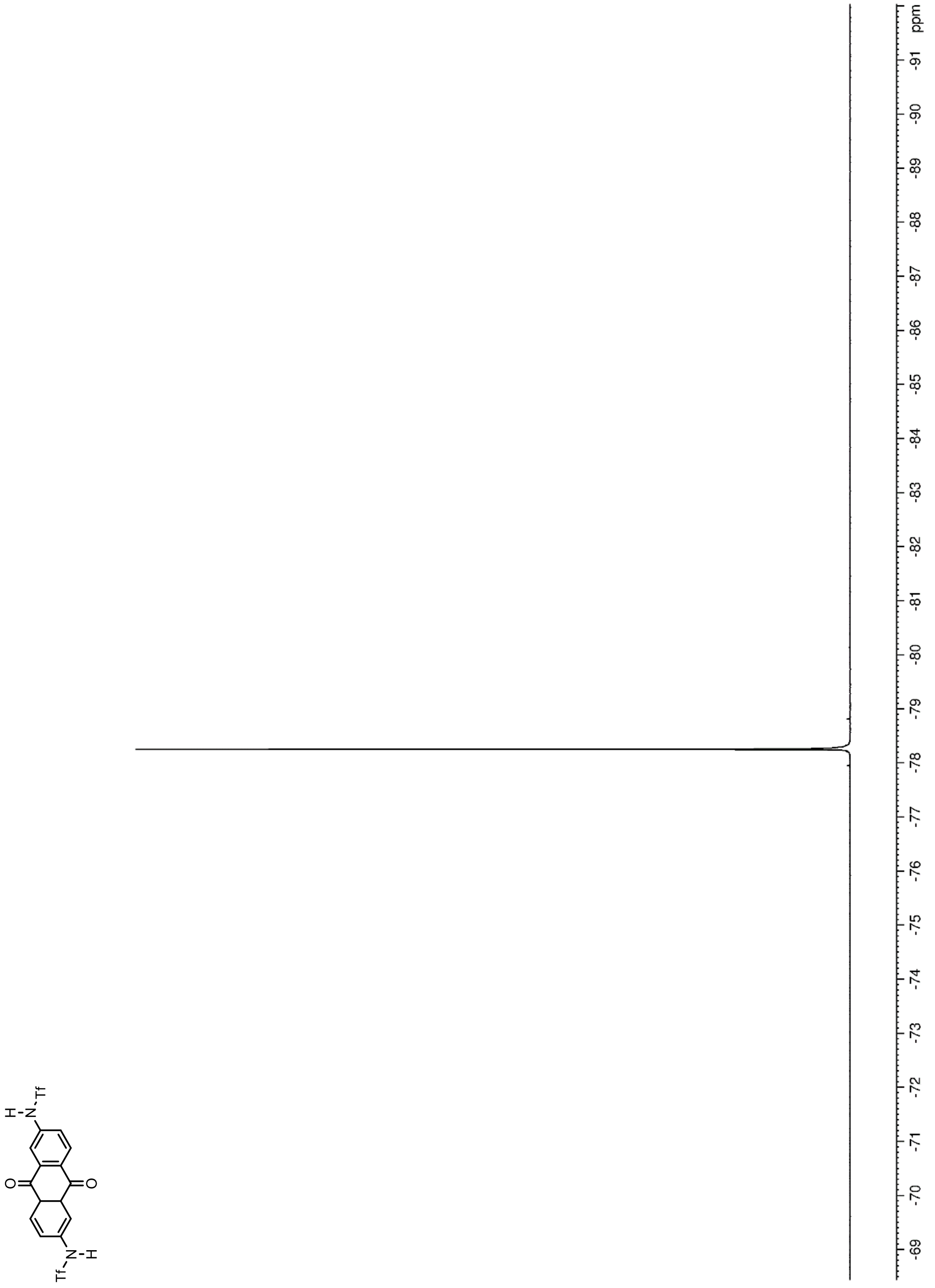

Figure 205. ^{1}H NMR (400 MHz, acetone-d_6) of **M1**

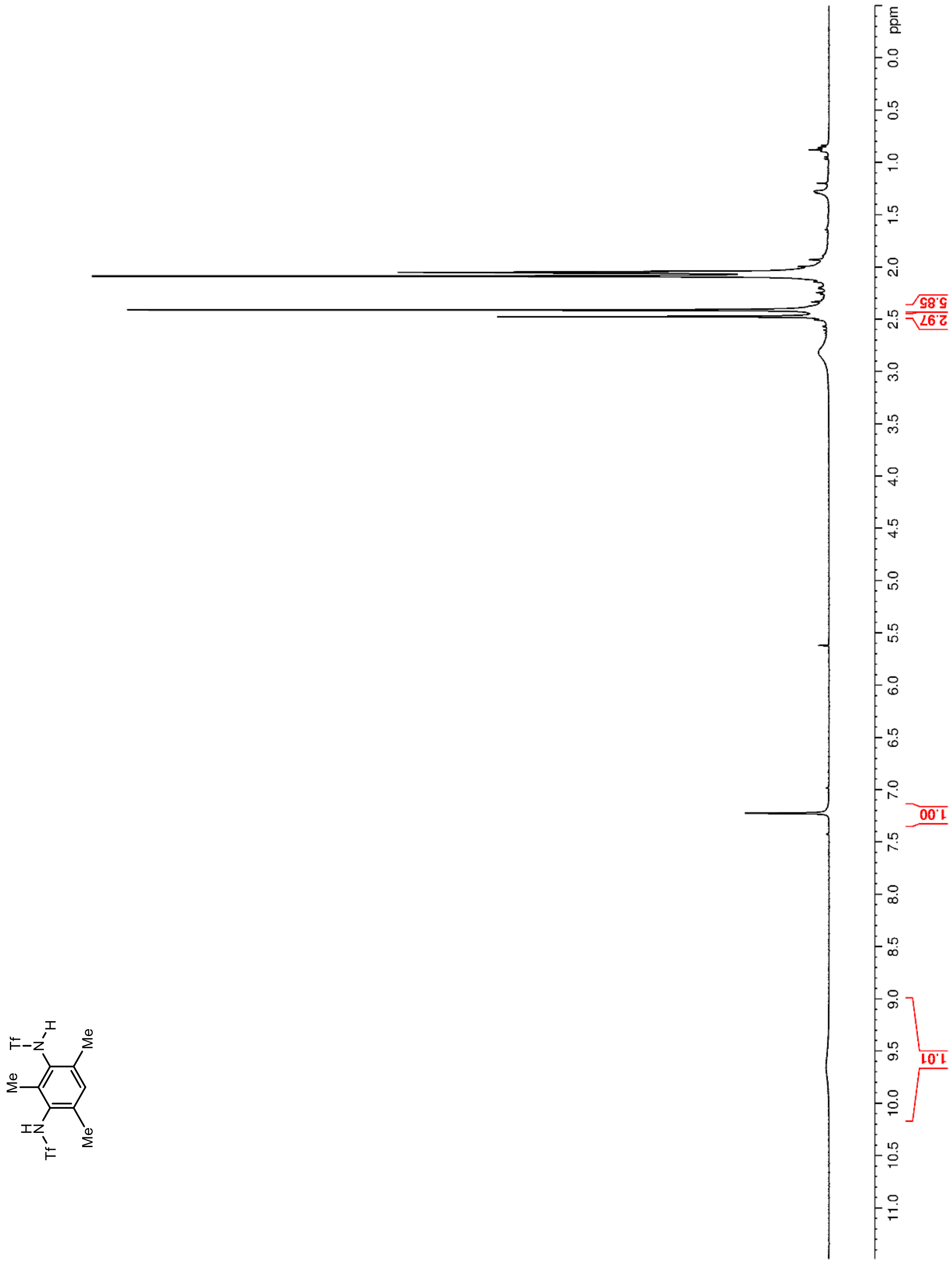

Figure 206. ^{13}C NMR (150 MHz, acetone-d_6) of **M1**

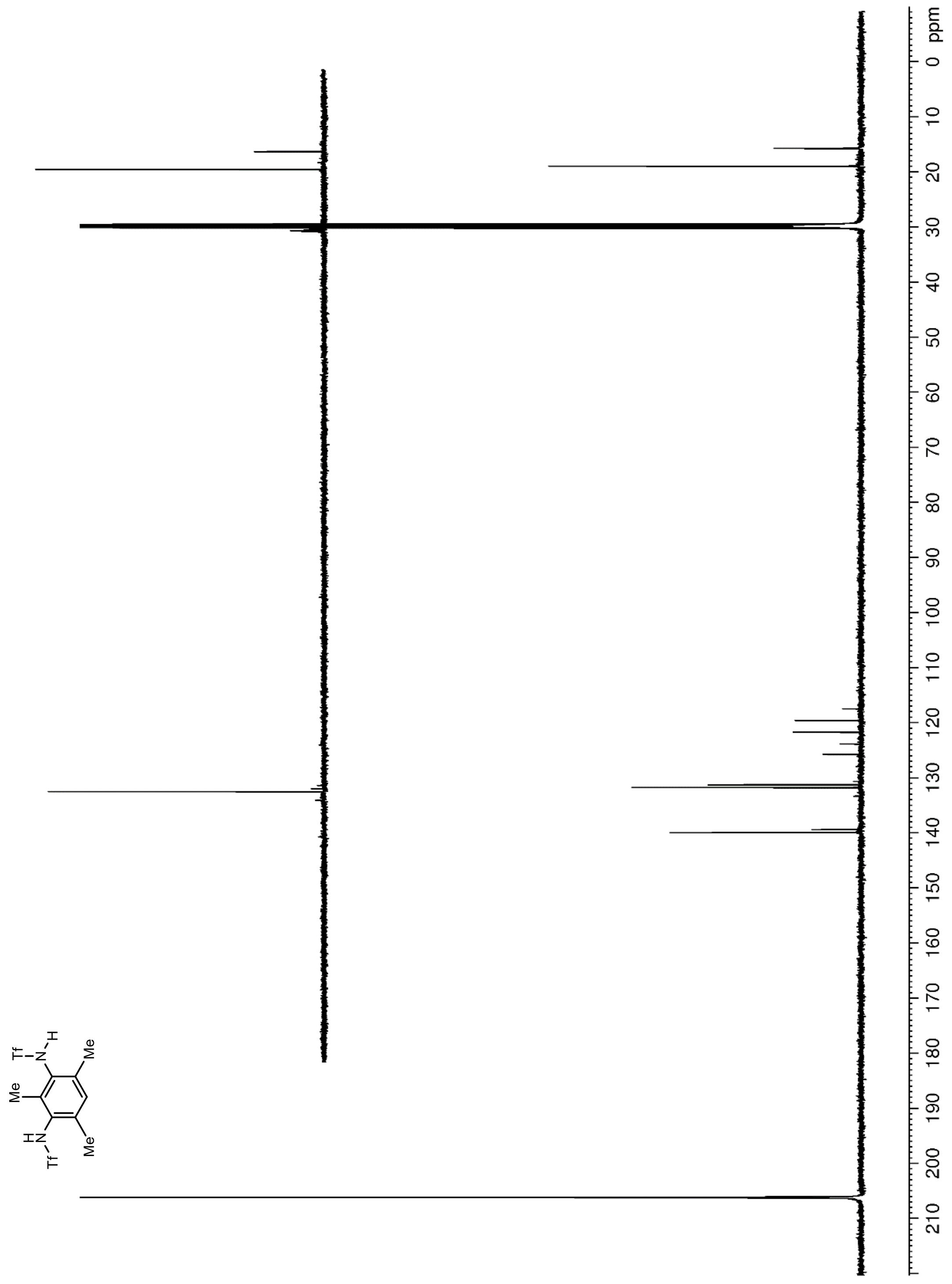

Figure 207. ^{19}F NMR (282 MHz, acetone-d_6) of **M1**

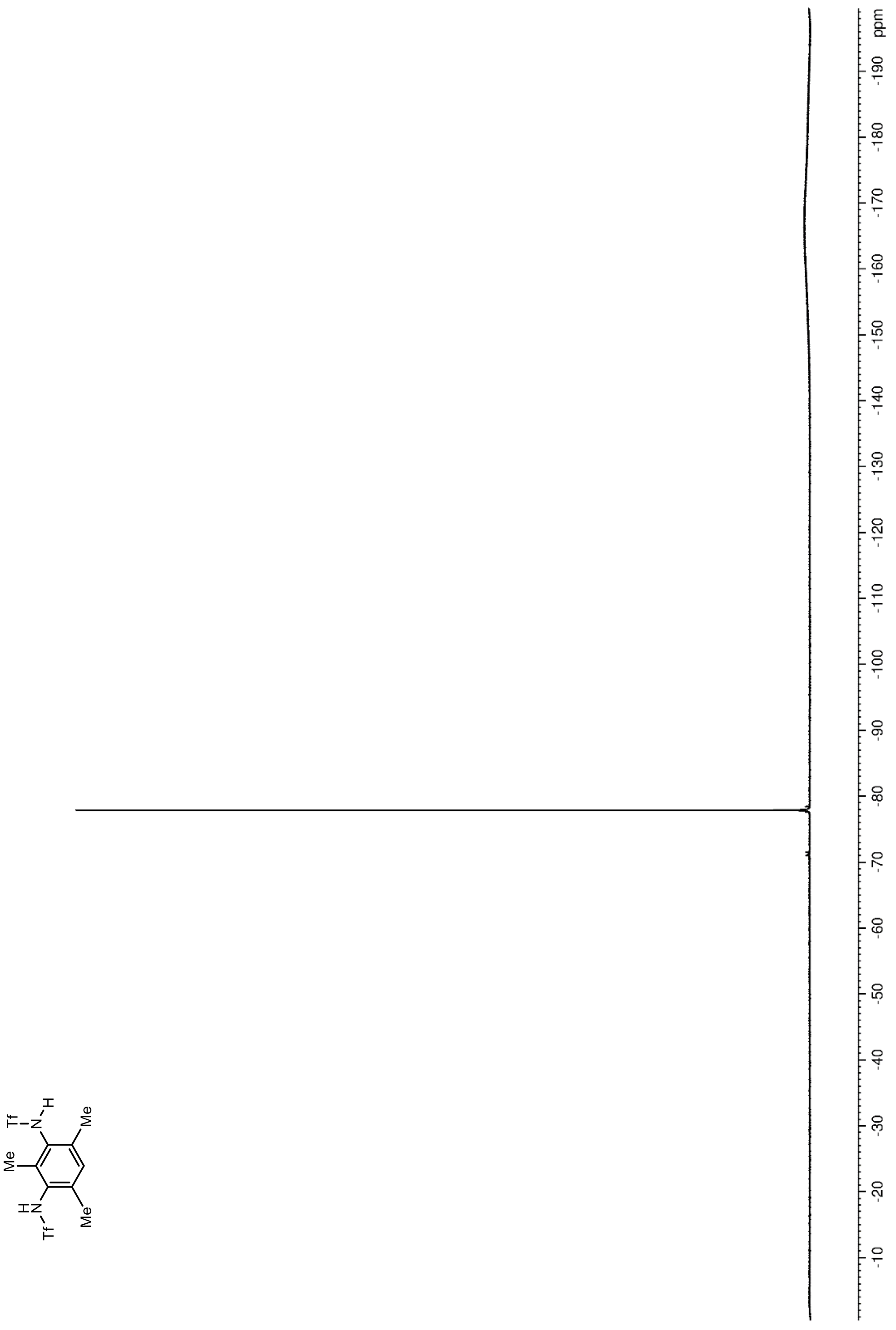

Figure 208. ^{1}H NMR (400 MHz, acetone-d_6) of **M2**

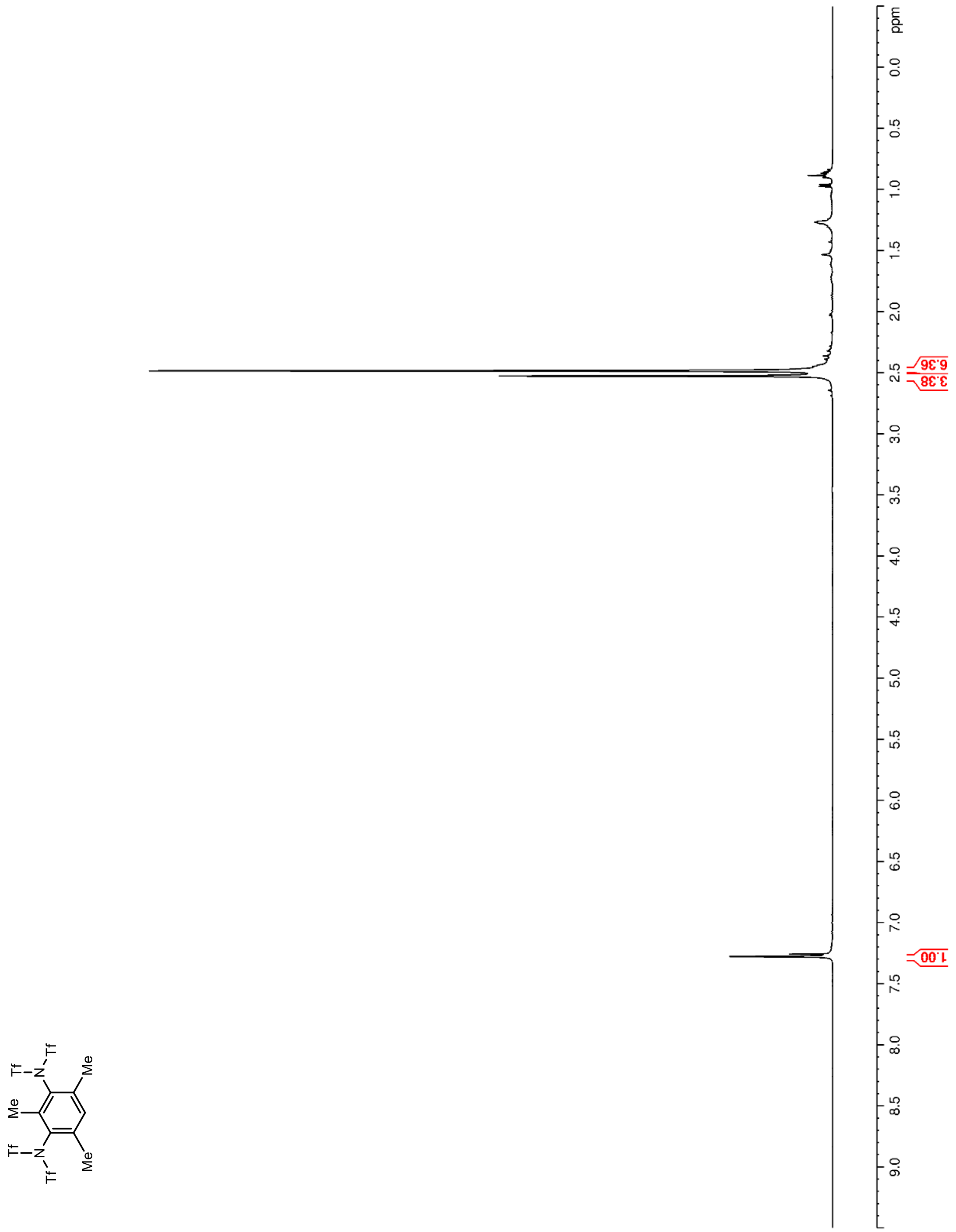

Figure 209. ^{13}C NMR (150 MHz, acetone-d_6) of **M2**

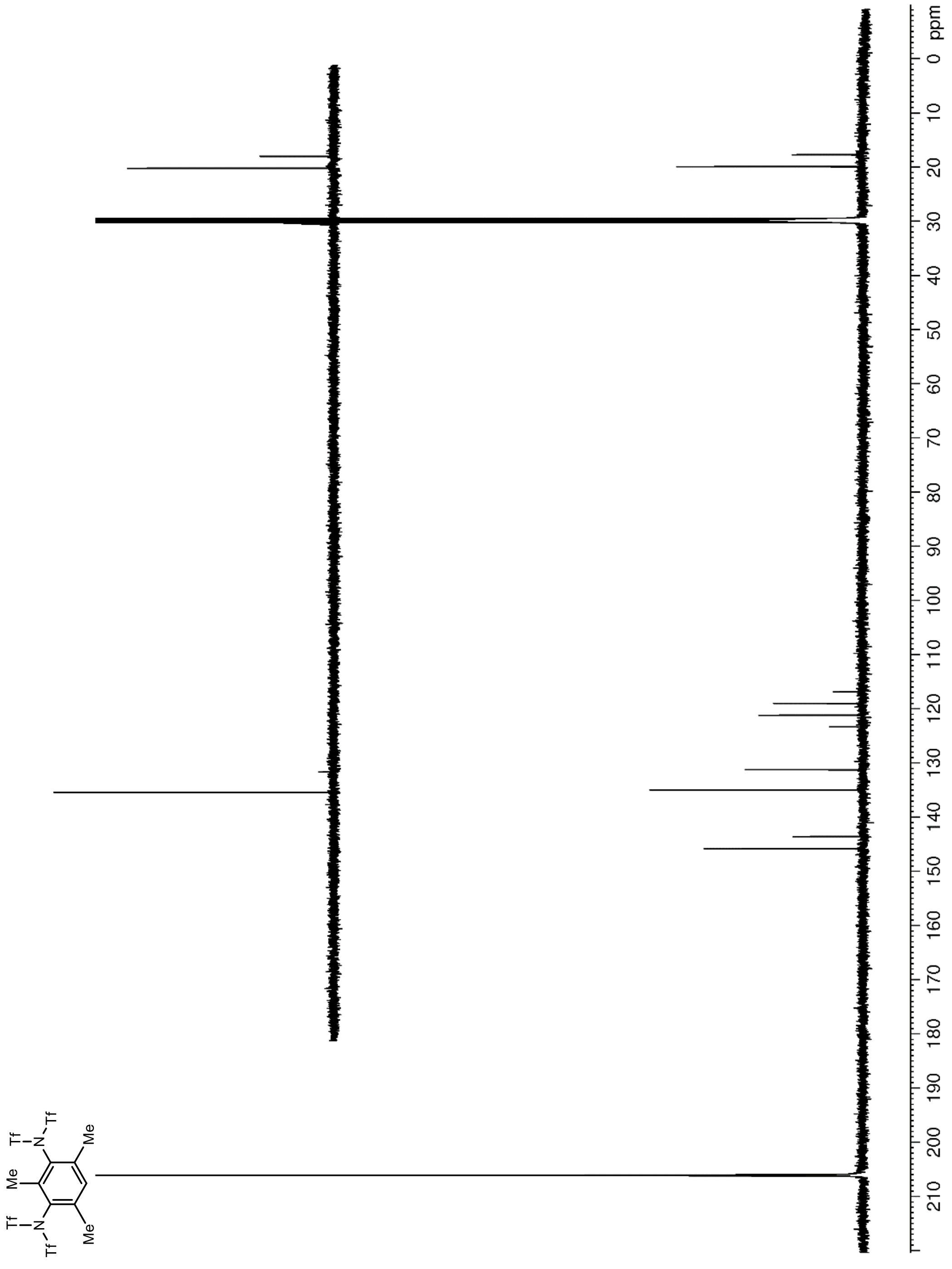

Figure 210. ^{19}F NMR (282 MHz, acetone-d_6) of **M2**

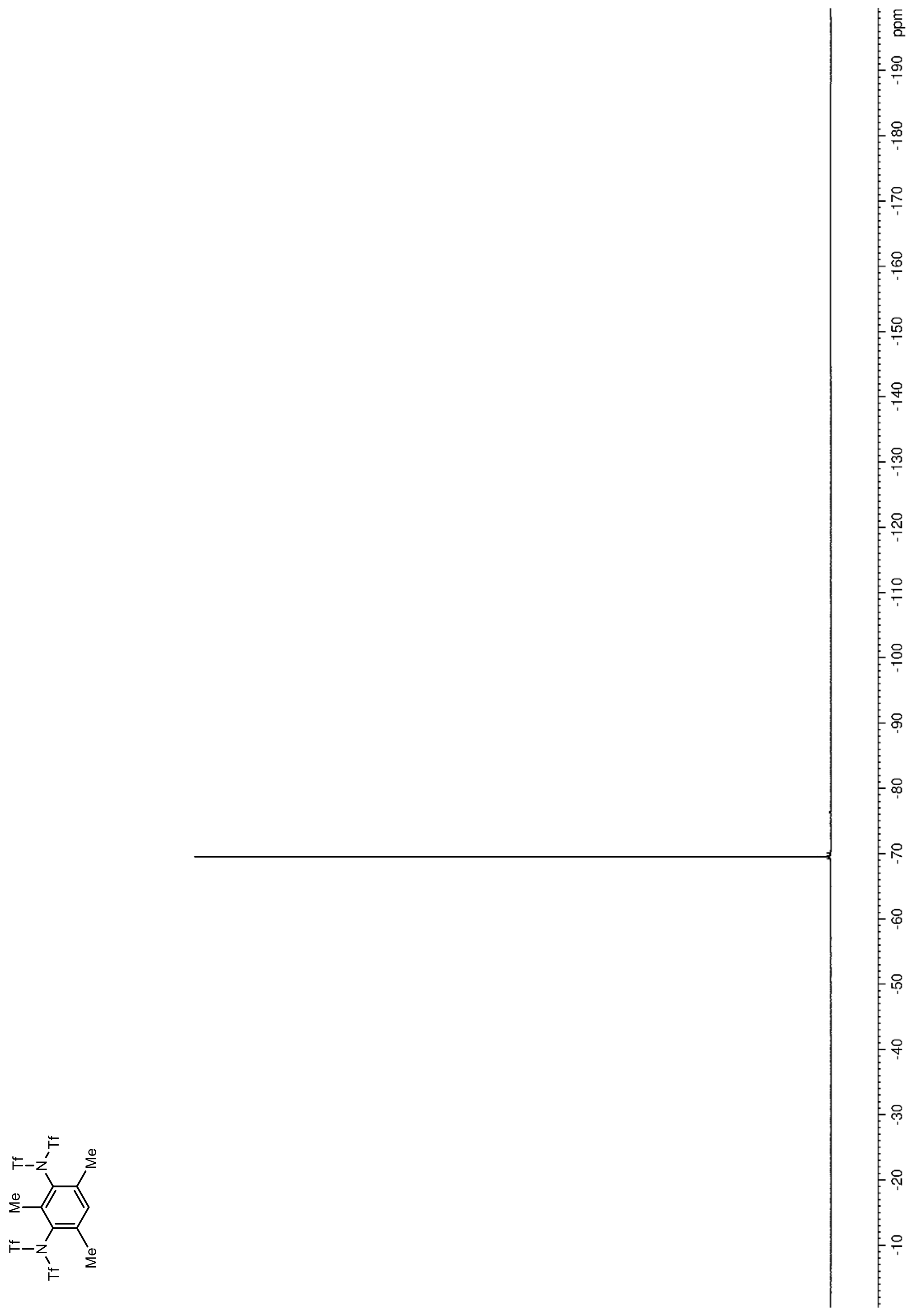

Figure 211. ^{1}H NMR (400 MHz, acetone-d_6) of **M3**

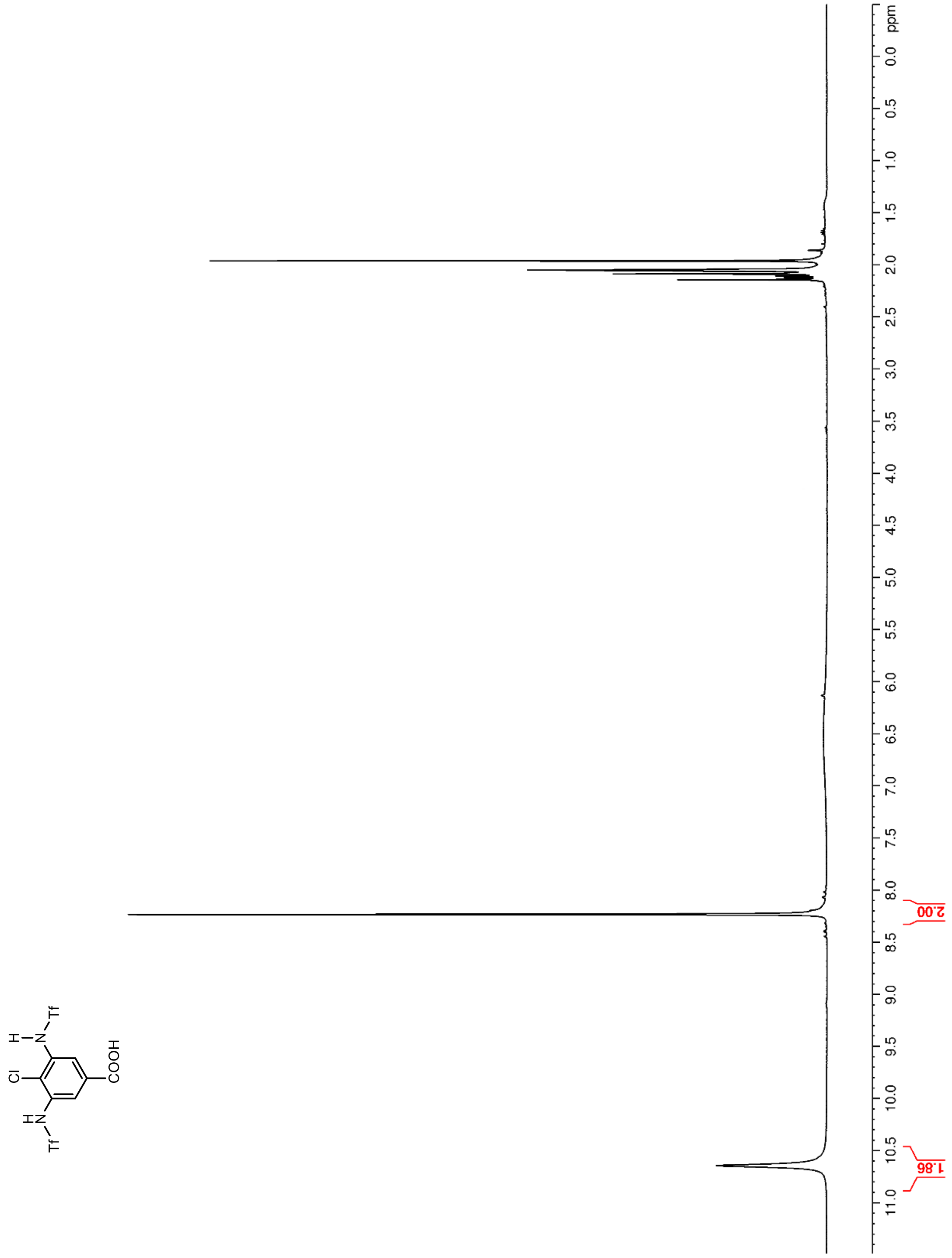

Figure 212. ^{13}C NMR (150 MHz, acetone-d_6) of **M3**

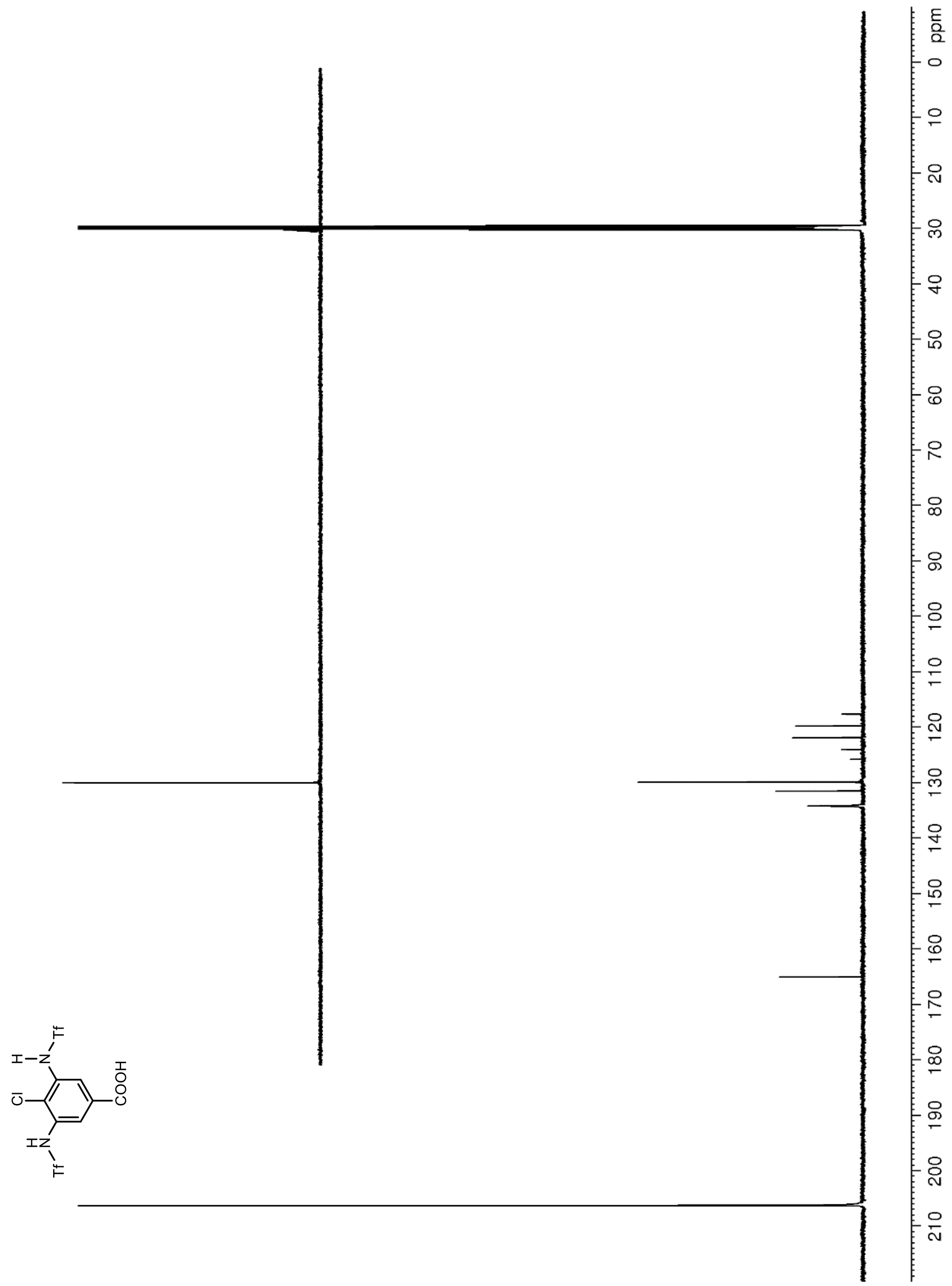

Figure 213. ^{19}F NMR (282 MHz, acetone-d_6) of **M3**

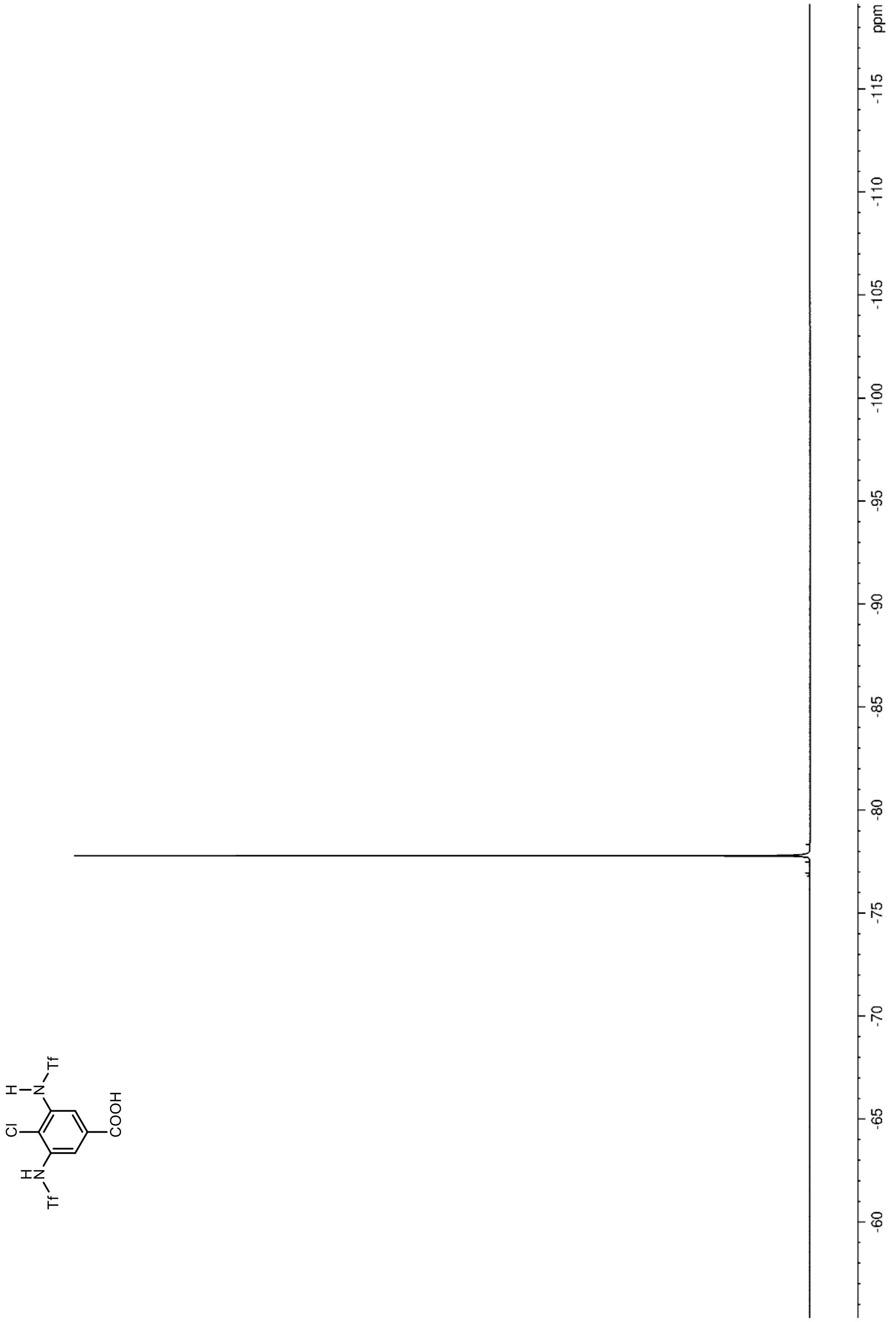

Figure 214. ^{1}H NMR (400 MHz, acetone-d_6) of **M4**

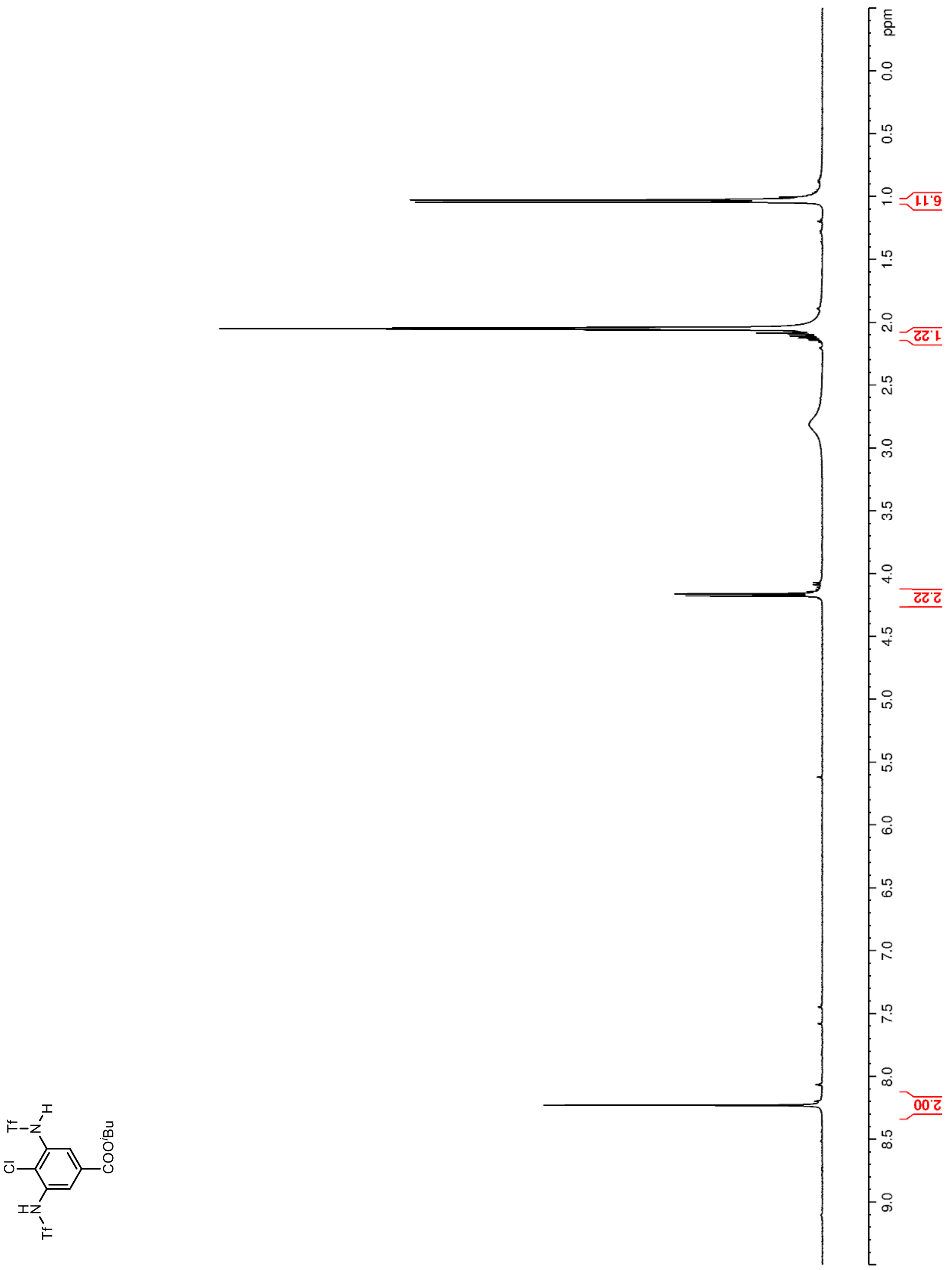

Figure 215. ^{13}C NMR (150 MHz, acetone-d_6) of **M4**

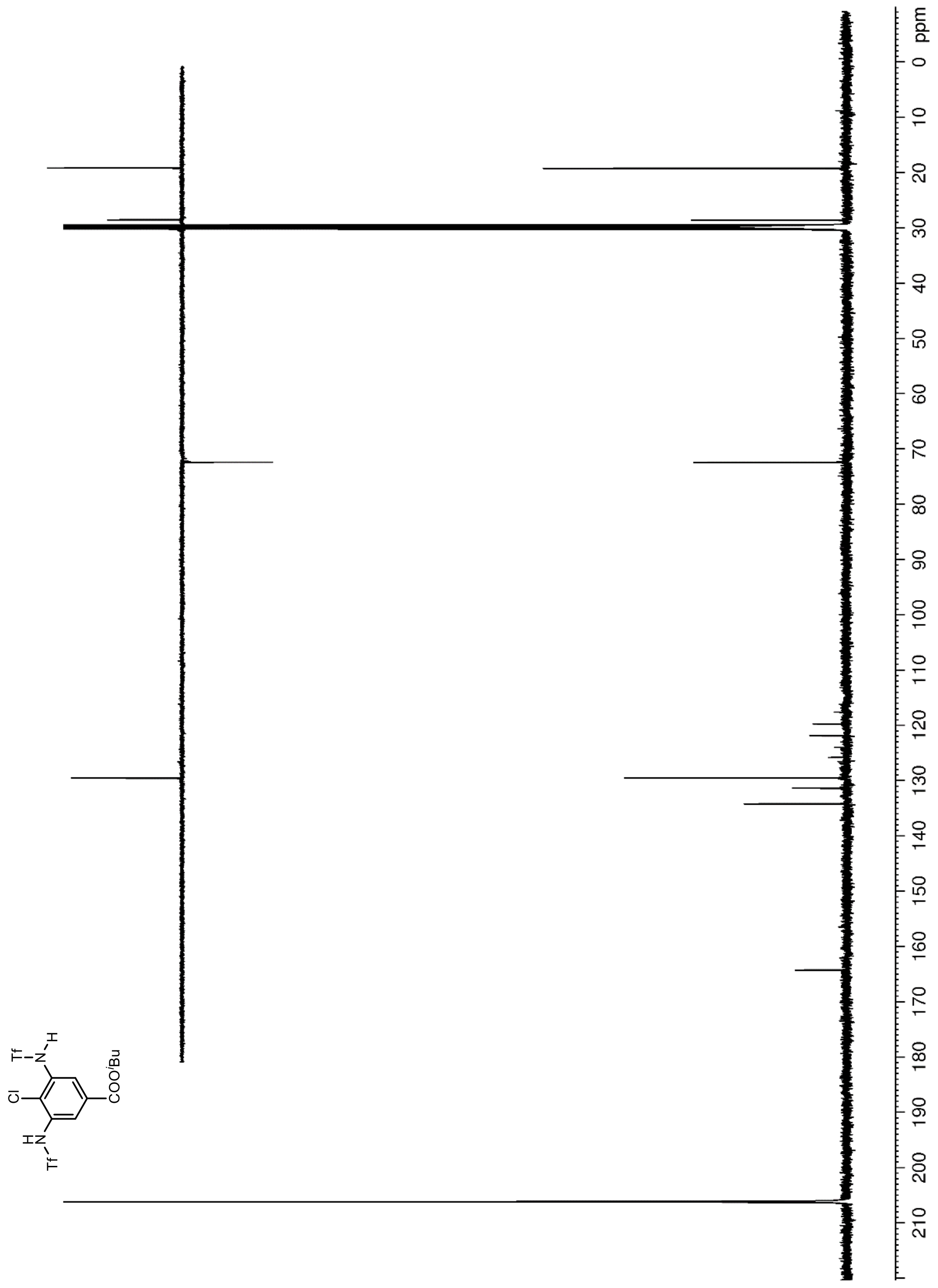

Figure 216. ^{19}F NMR (282 MHz, acetone-d_6) of **M4**

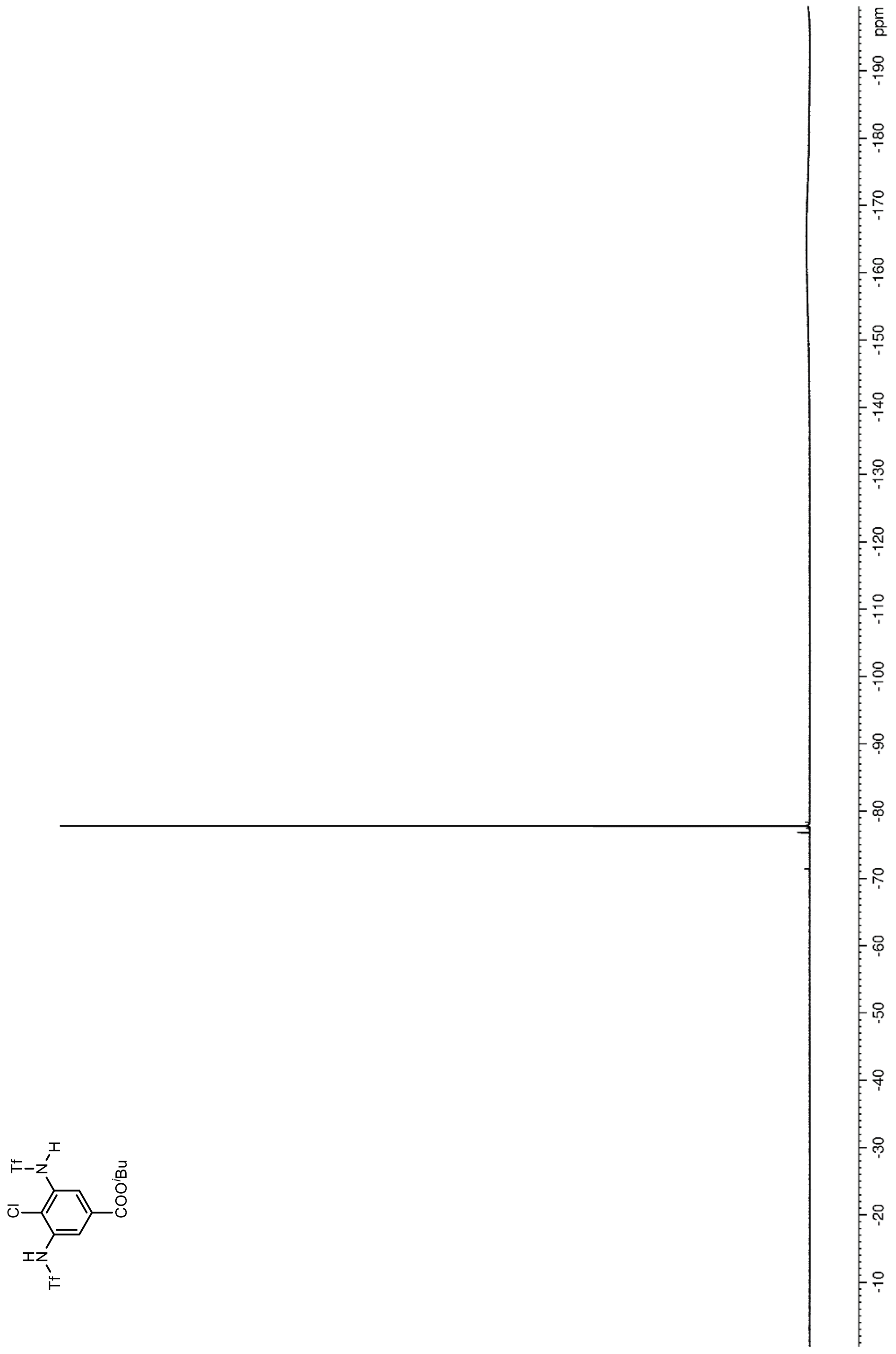

Figure 217. ^{1}H NMR (400 MHz, acetone-d_6) of **M5**

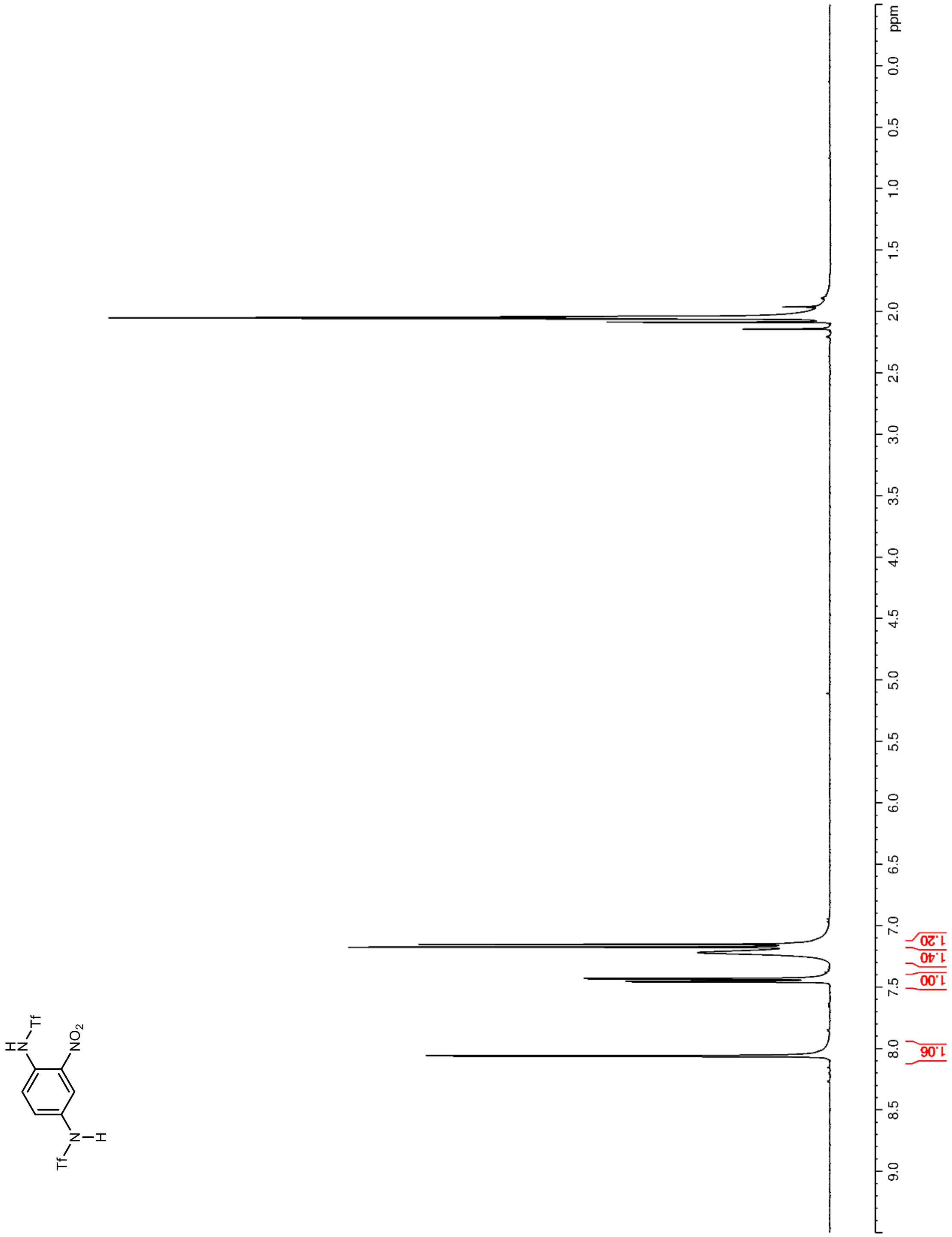

Figure 218. ^{13}C NMR (150 MHz, acetone-d_6) of **M5**

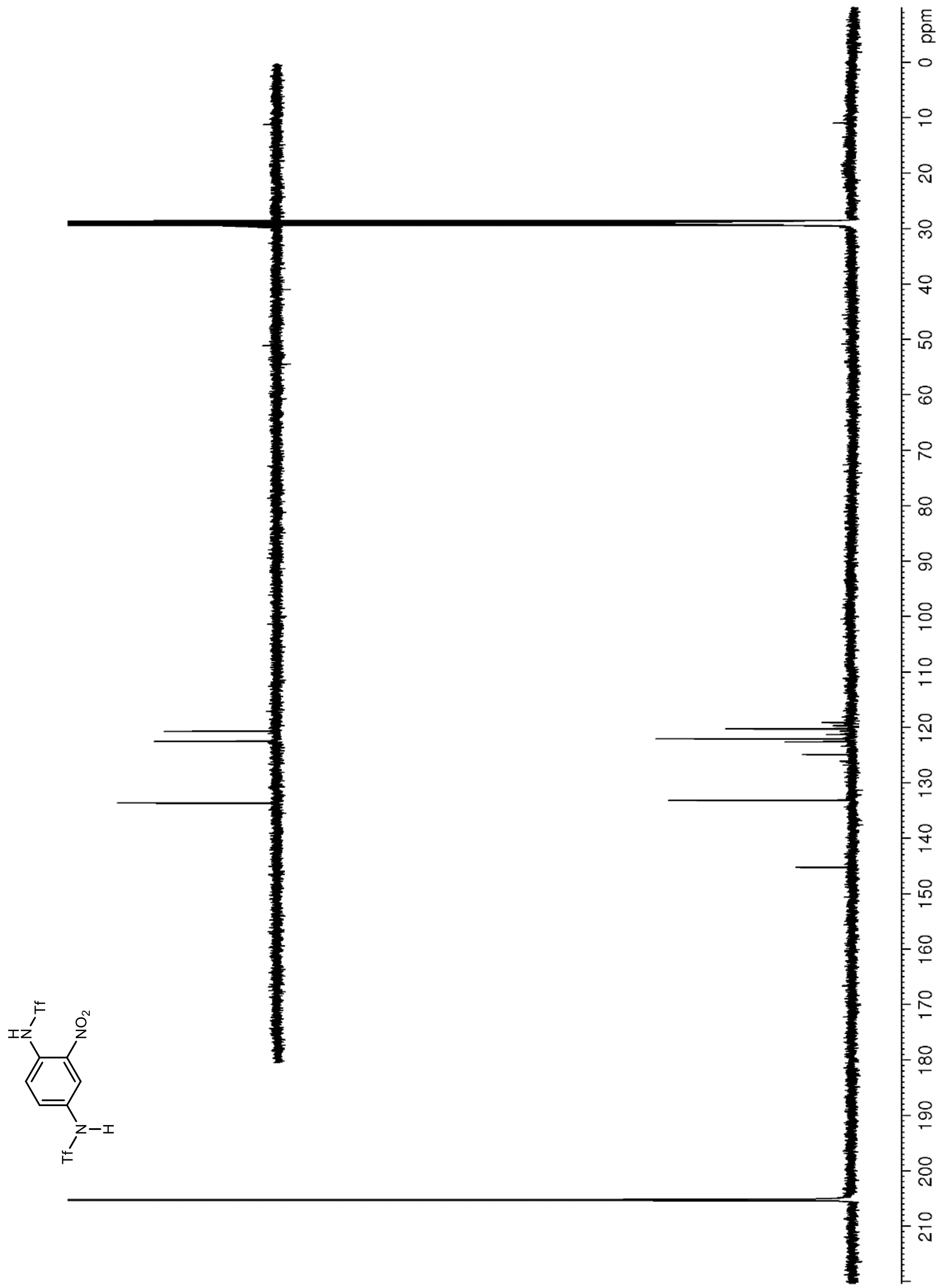

Figure 219. ^{19}F NMR (282 MHz, acetone-d_6) of **M5**

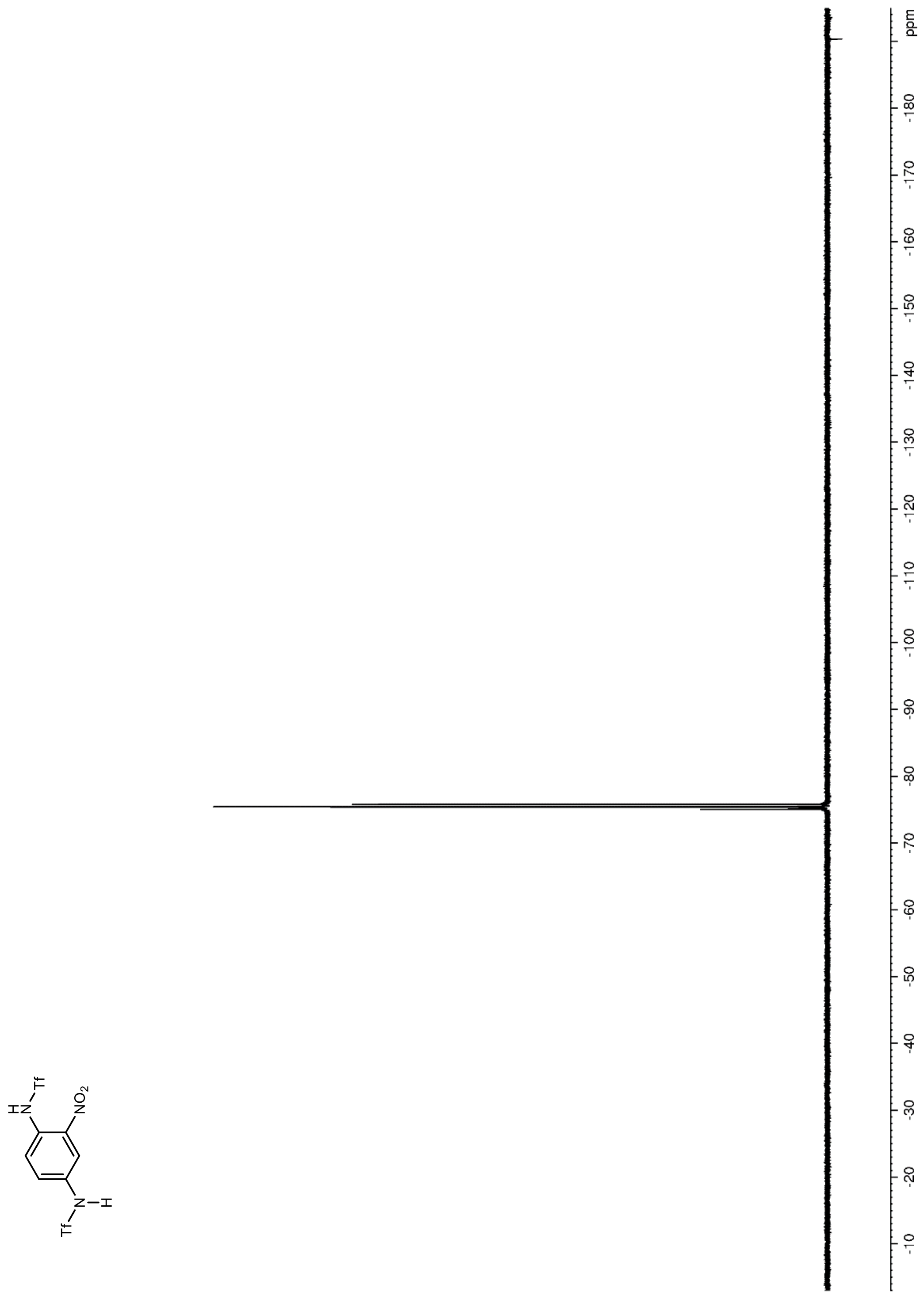

Figure 220. ^{1}H NMR (400 MHz, $CDCl_3$) of **M7**

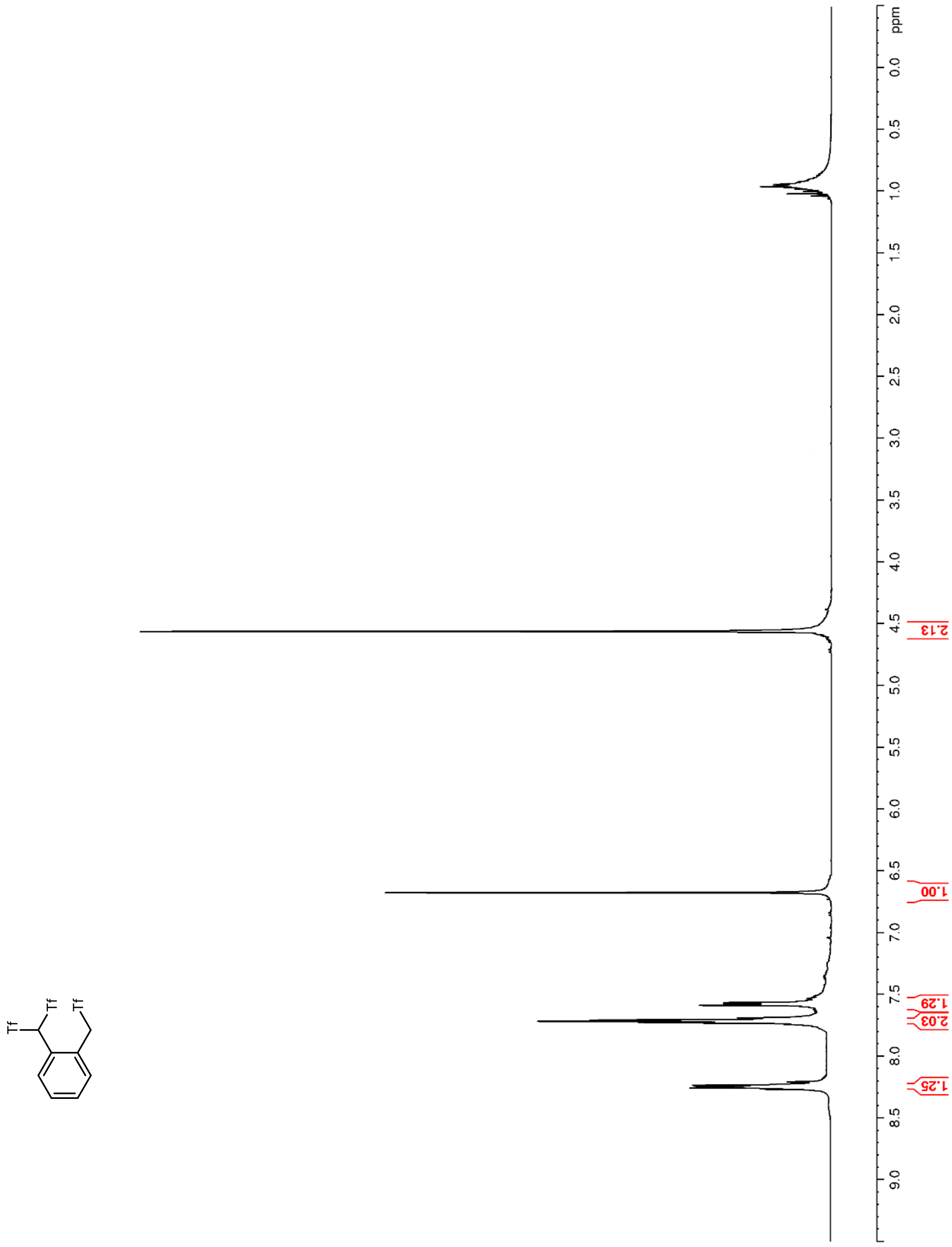

Figure 221. ^{13}C NMR (150 MHz, acetone-d_6) of **M7**

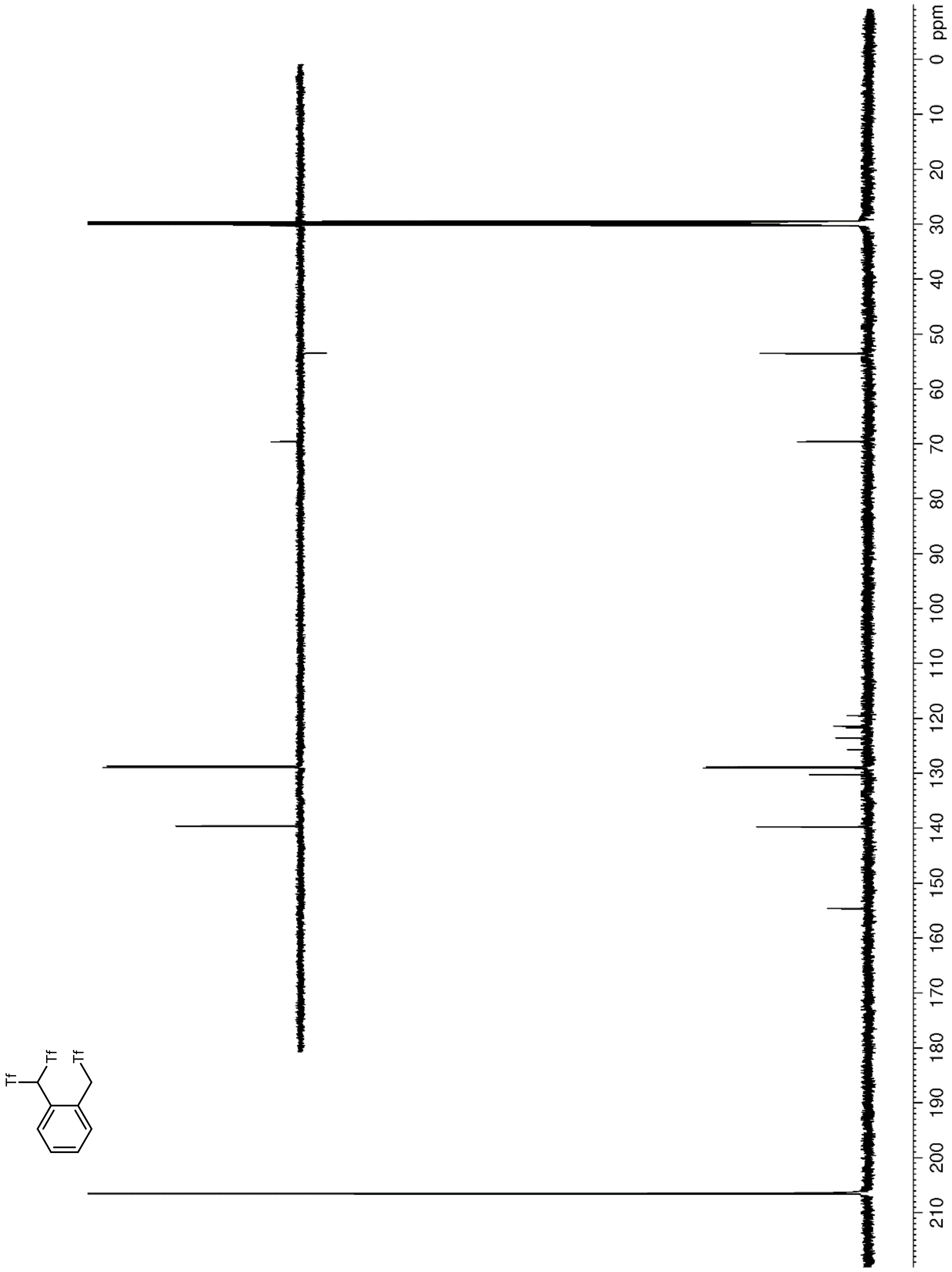

Figure 222. ^{19}F NMR (282 MHz, $CDCl_3$) of **M7**

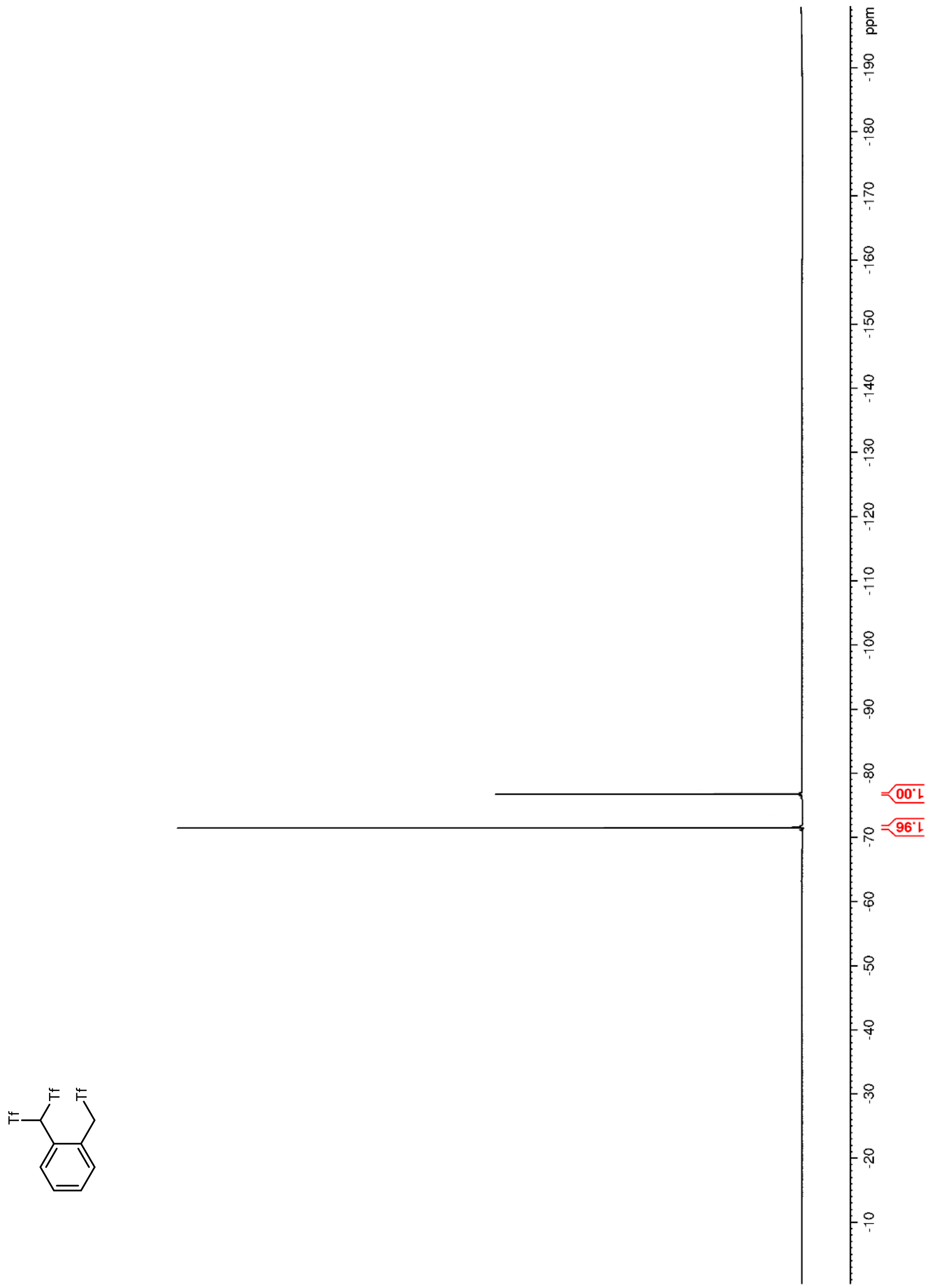

Figure 223. ^{1}H NMR (400 MHz, $CDCl_3$) of **M8**

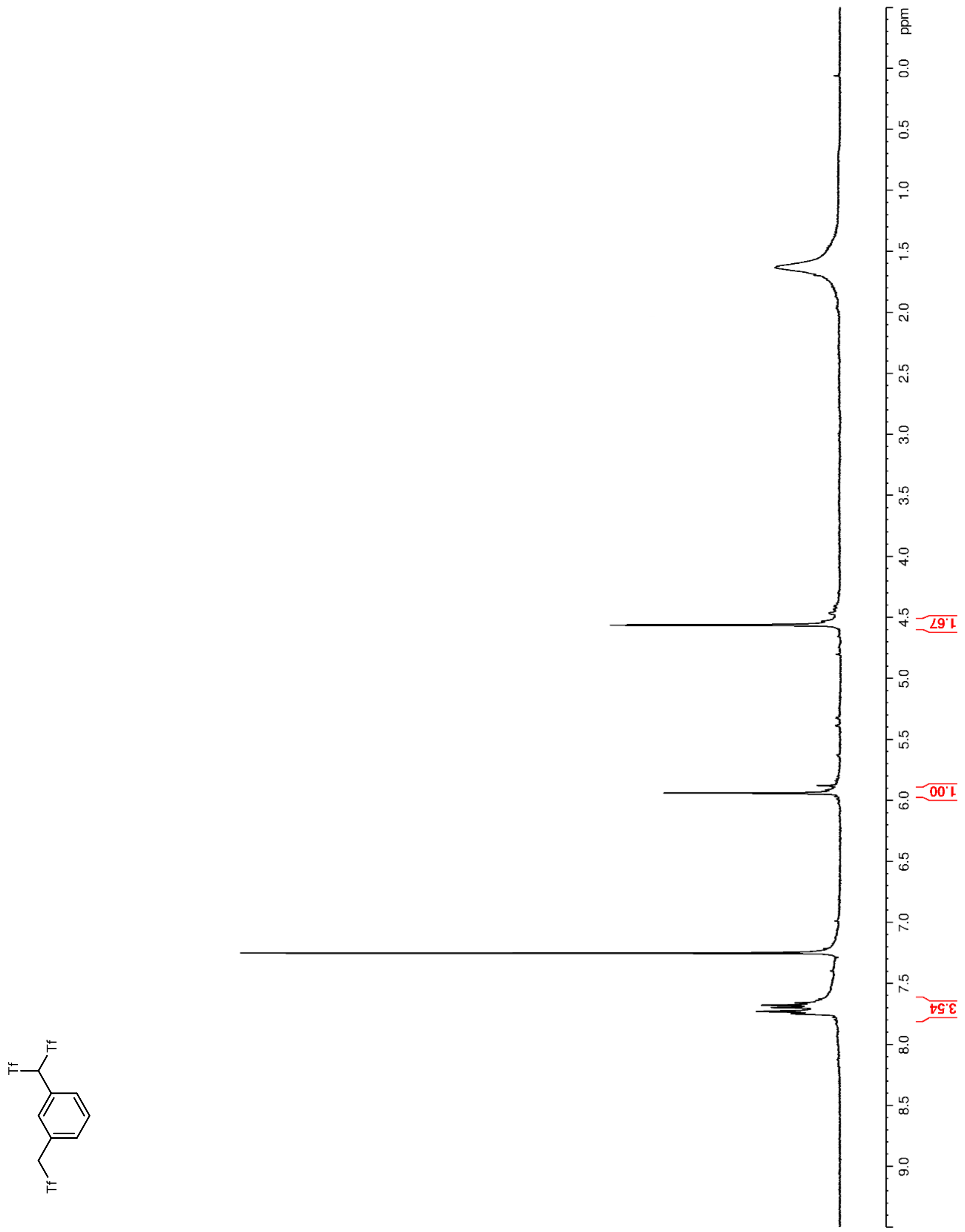

Figure 224. ^{13}C NMR (150 MHz, acetone-d_6) of **M8**

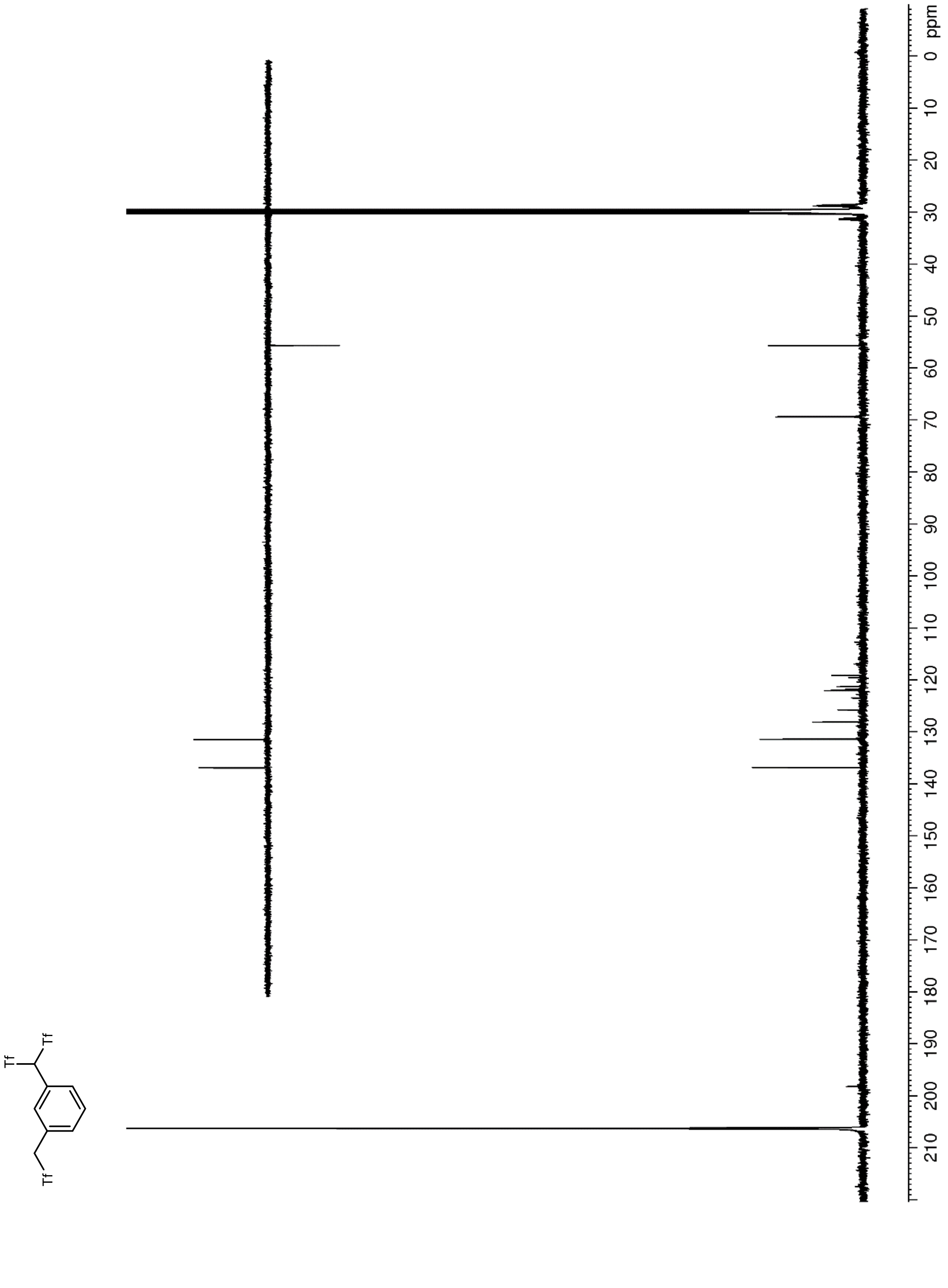

Figure 225. ^{19}F NMR (282 MHz, $CDCl_3$) of **M8**

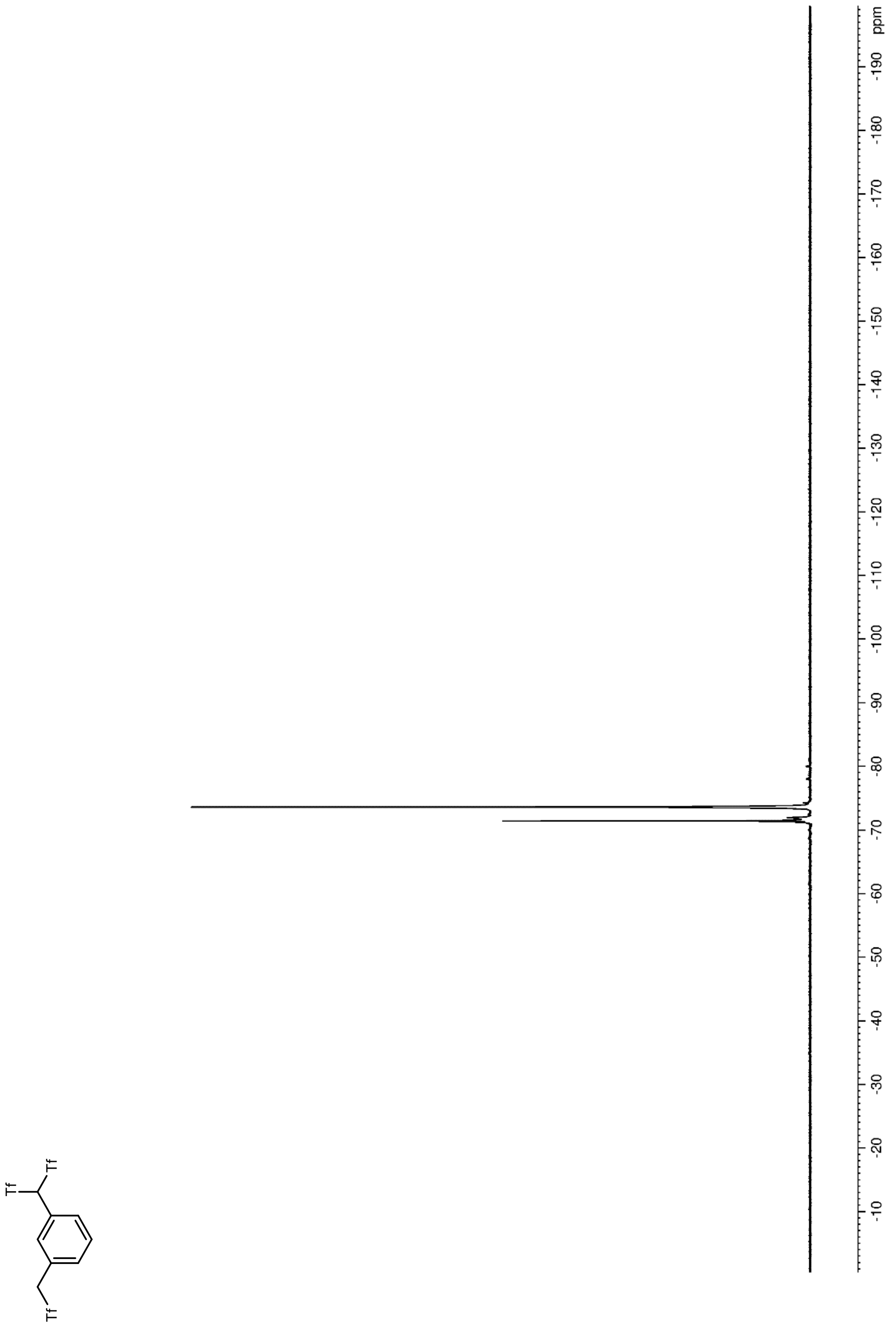

Figure 226. ^{1}H NMR (400 MHz, $CDCl_3$) of **2b**

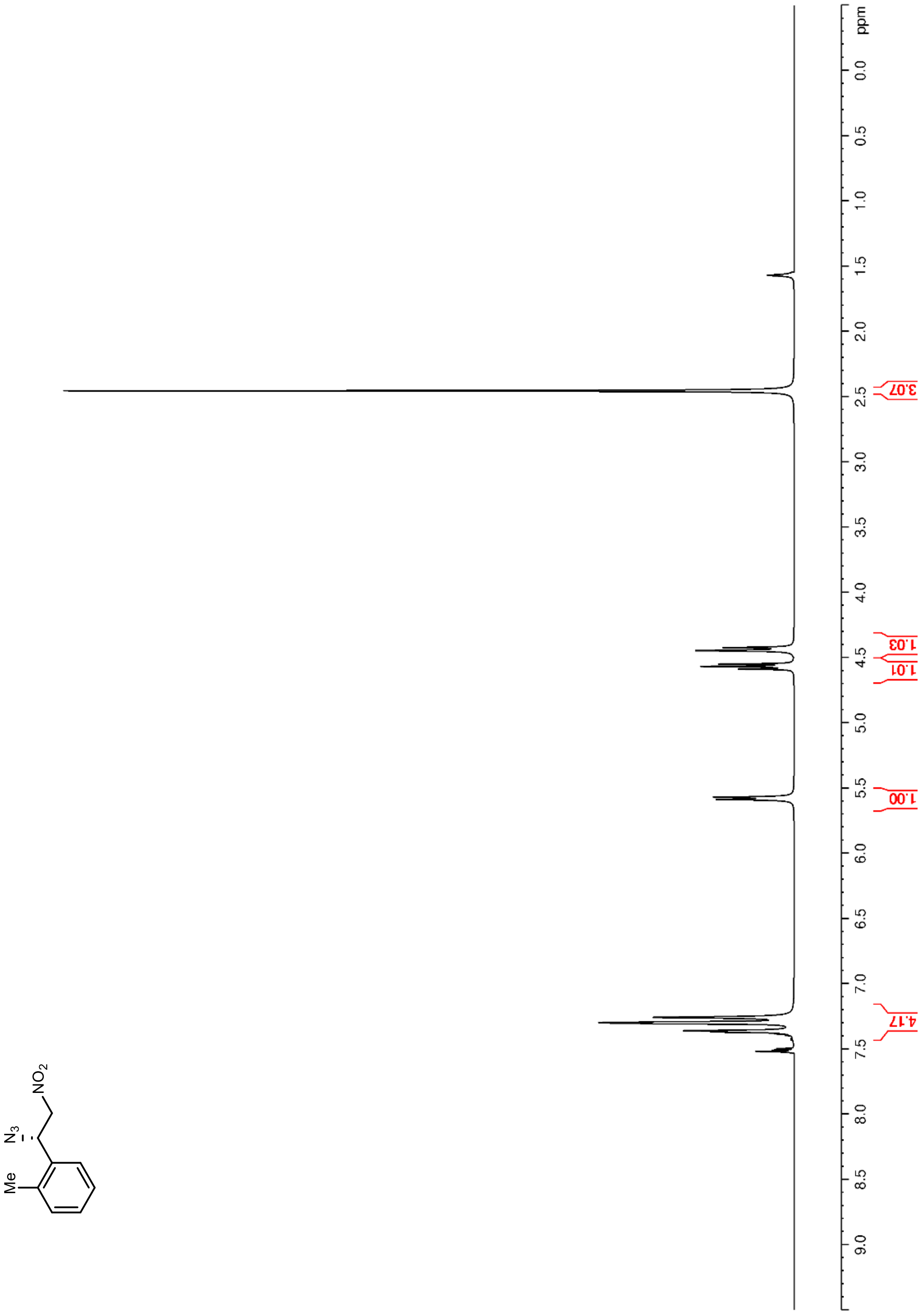

Figure 227. ^{13}C NMR (150 MHz, $CDCl_3$) of **2b**

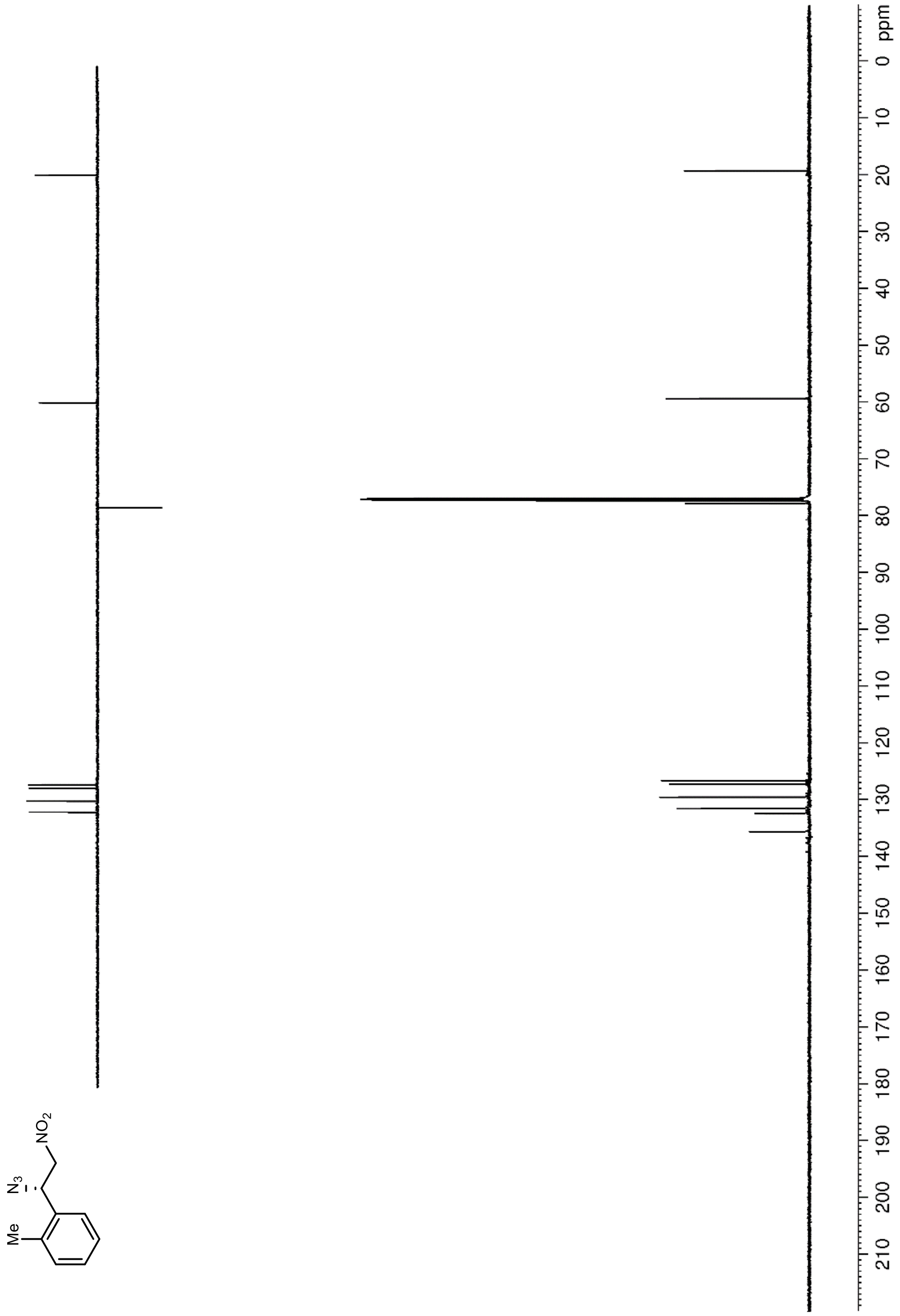

Figure 228. ^{1}H NMR (400 MHz, $CDCl_3$) of **2c**

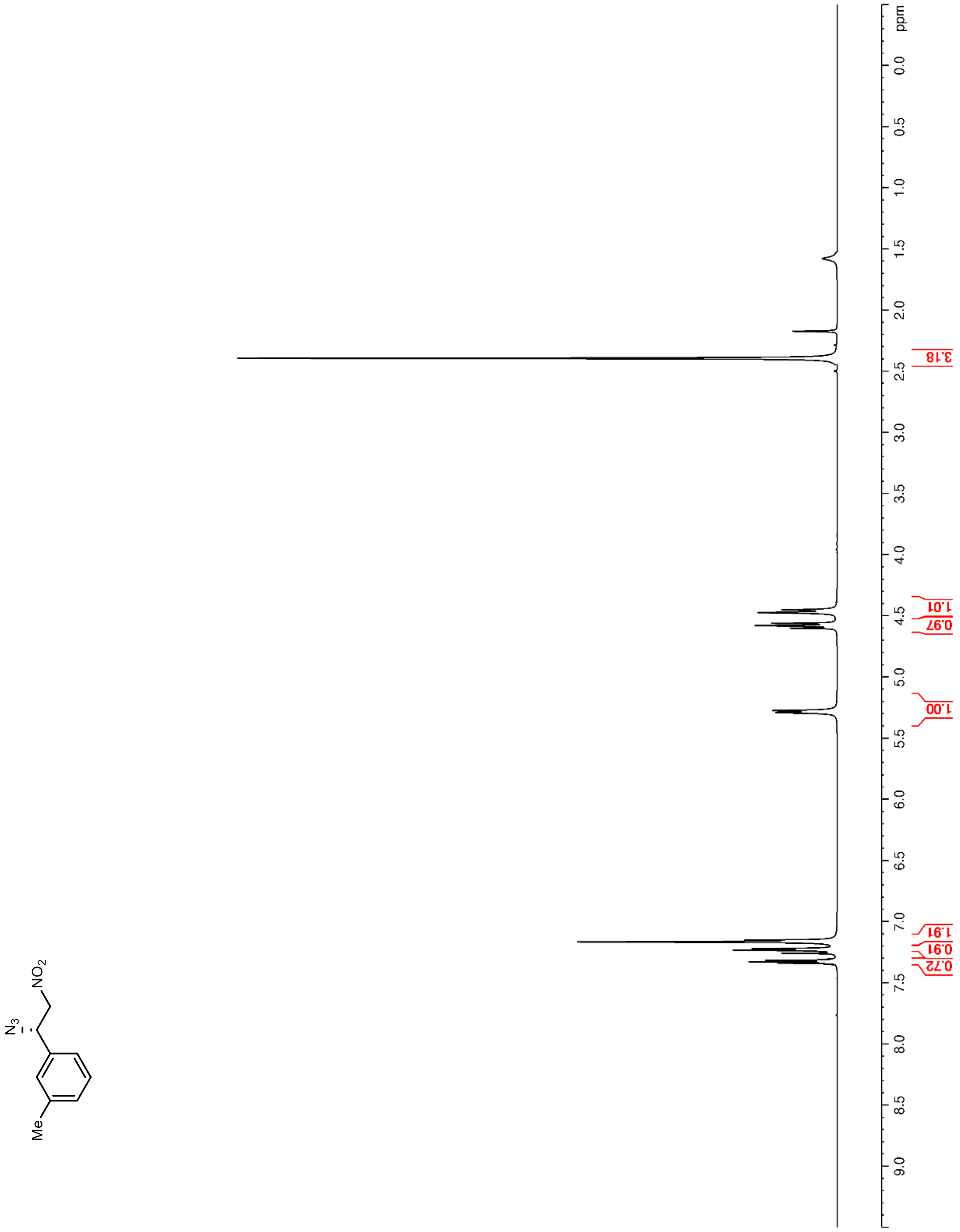

Figure 229. ^{13}C NMR (150 MHz, $CDCl_3$) of **2c**

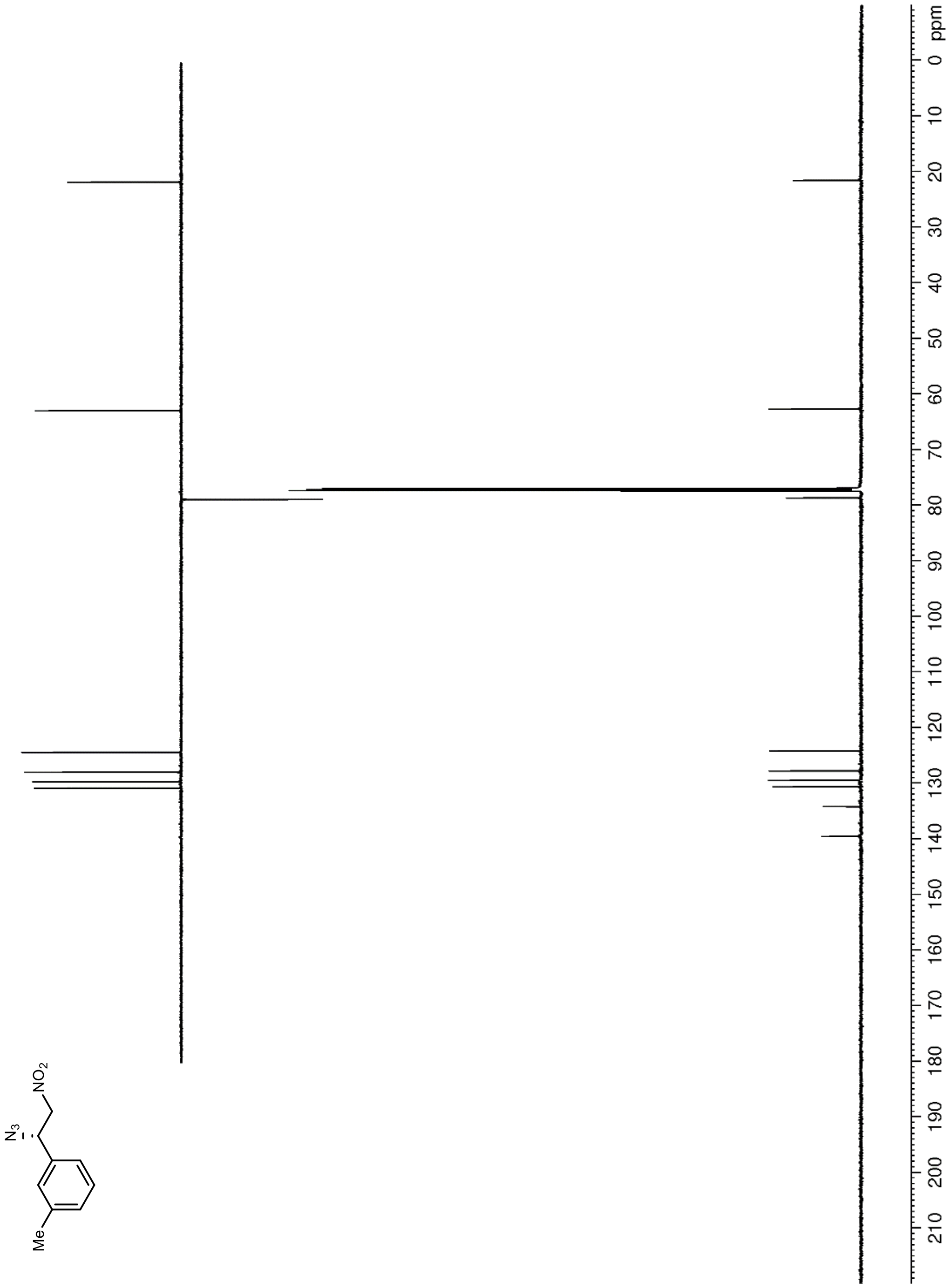

Figure 230. ^{1}H NMR (400 MHz, $CDCl_3$) of **2d**

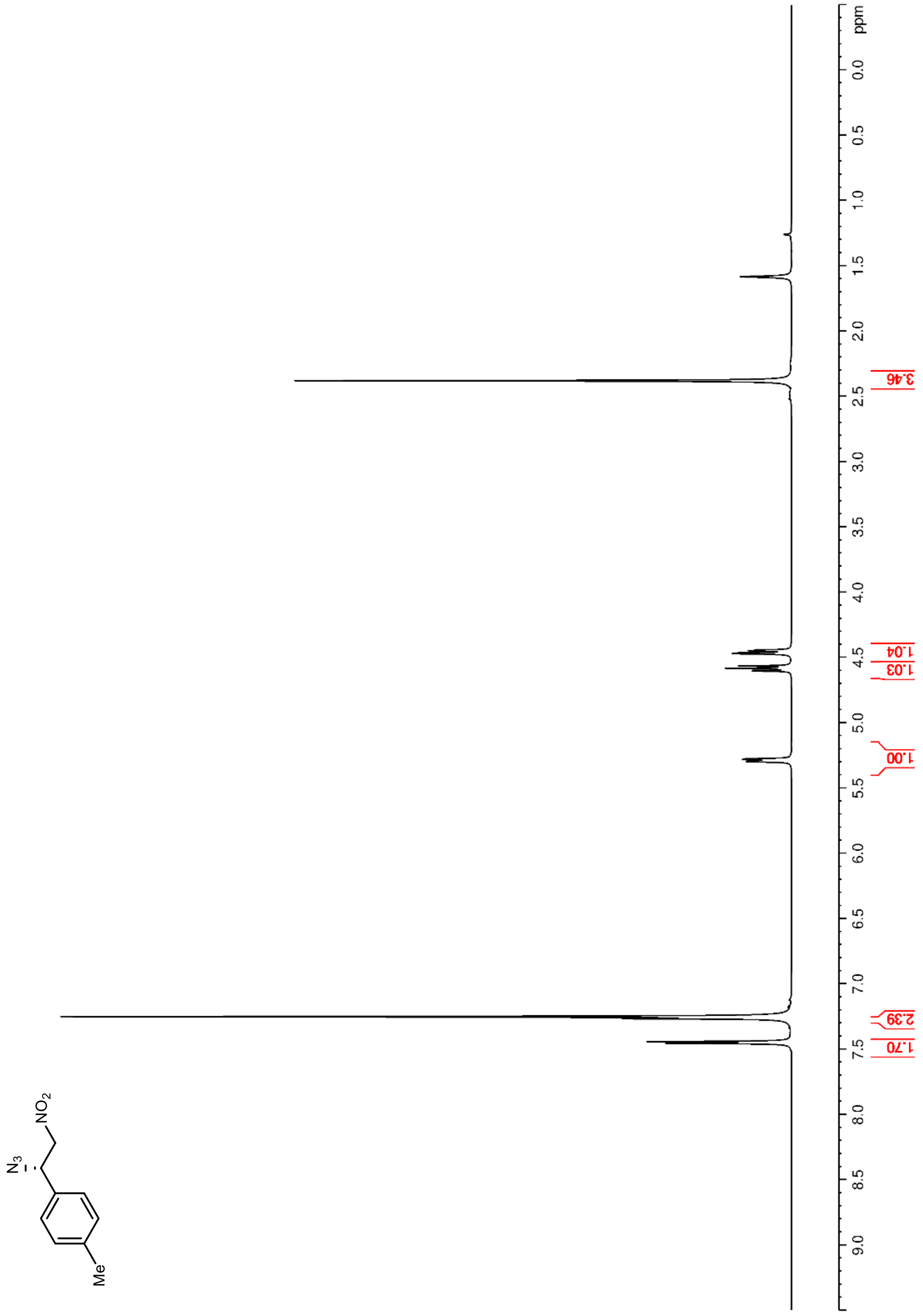

Figure 231. ^{13}C NMR (150 MHz, $CDCl_3$) of **2d**

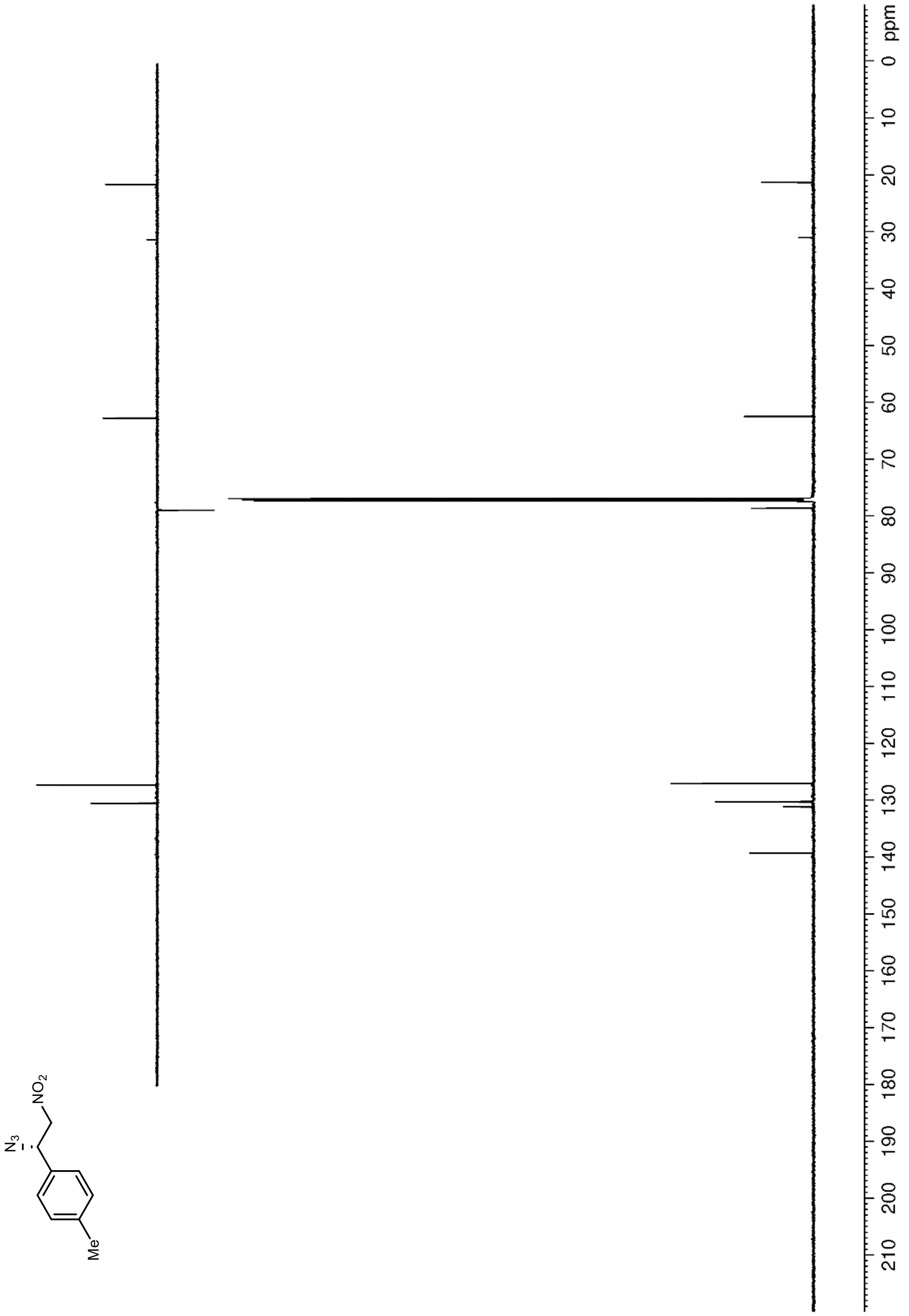

Figure 232. ^{1}H NMR (400 MHz, $CDCl_3$) of **2e**

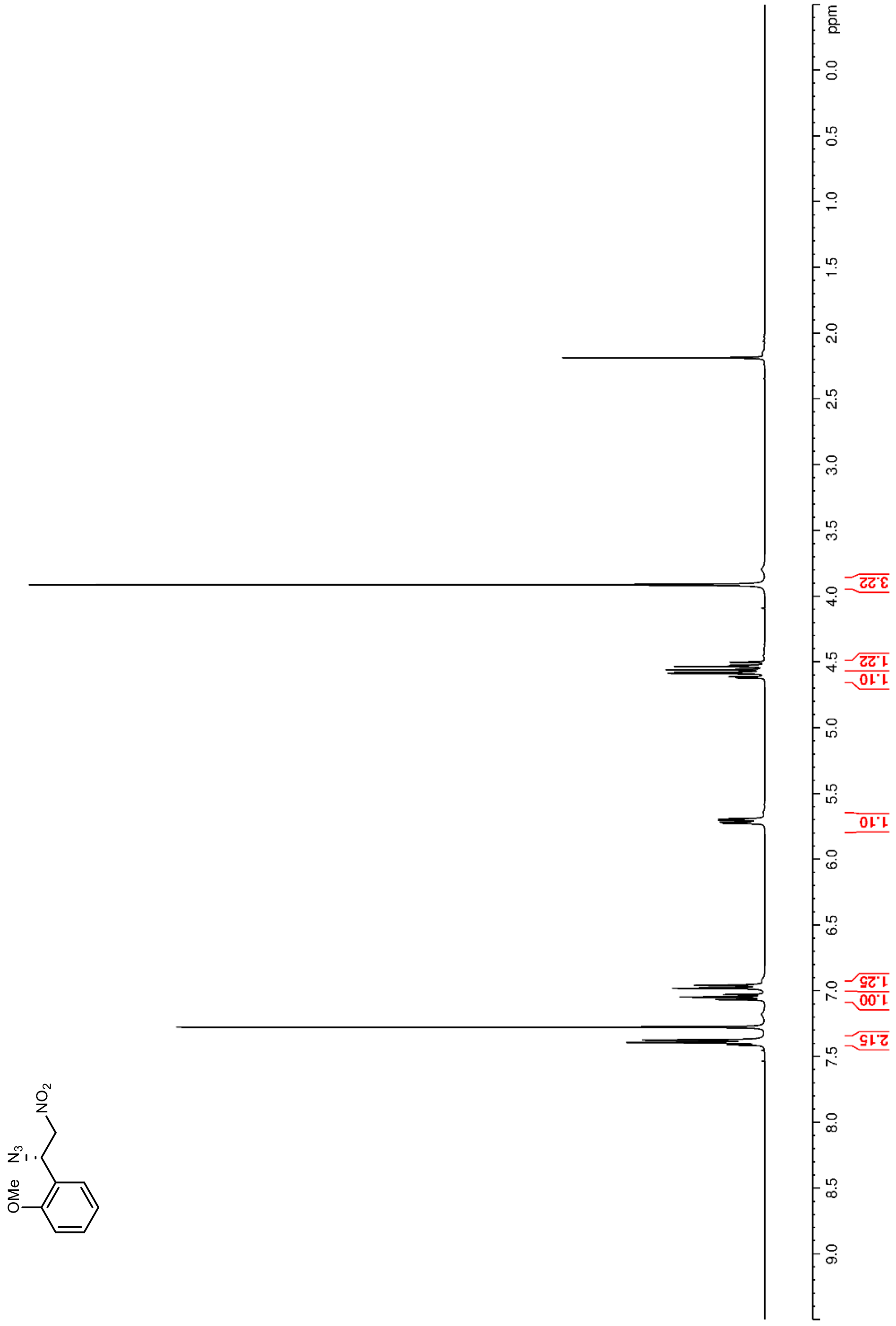

Figure 233. ^{13}C NMR (150 MHz, $CDCl_3$) of **2e**

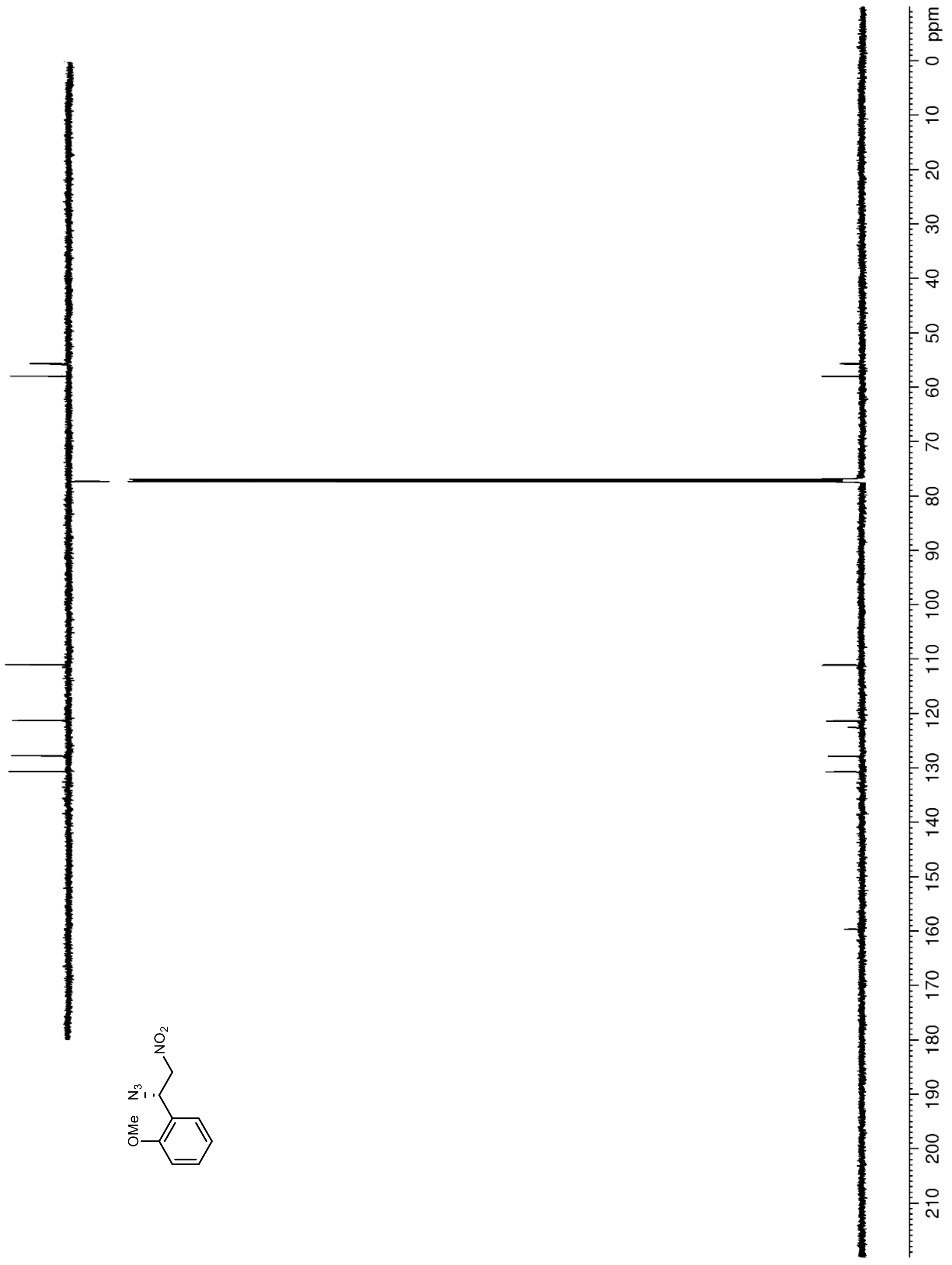

Figure 234. ^{1}H NMR (400 MHz, $CDCl_3$) of **2f**

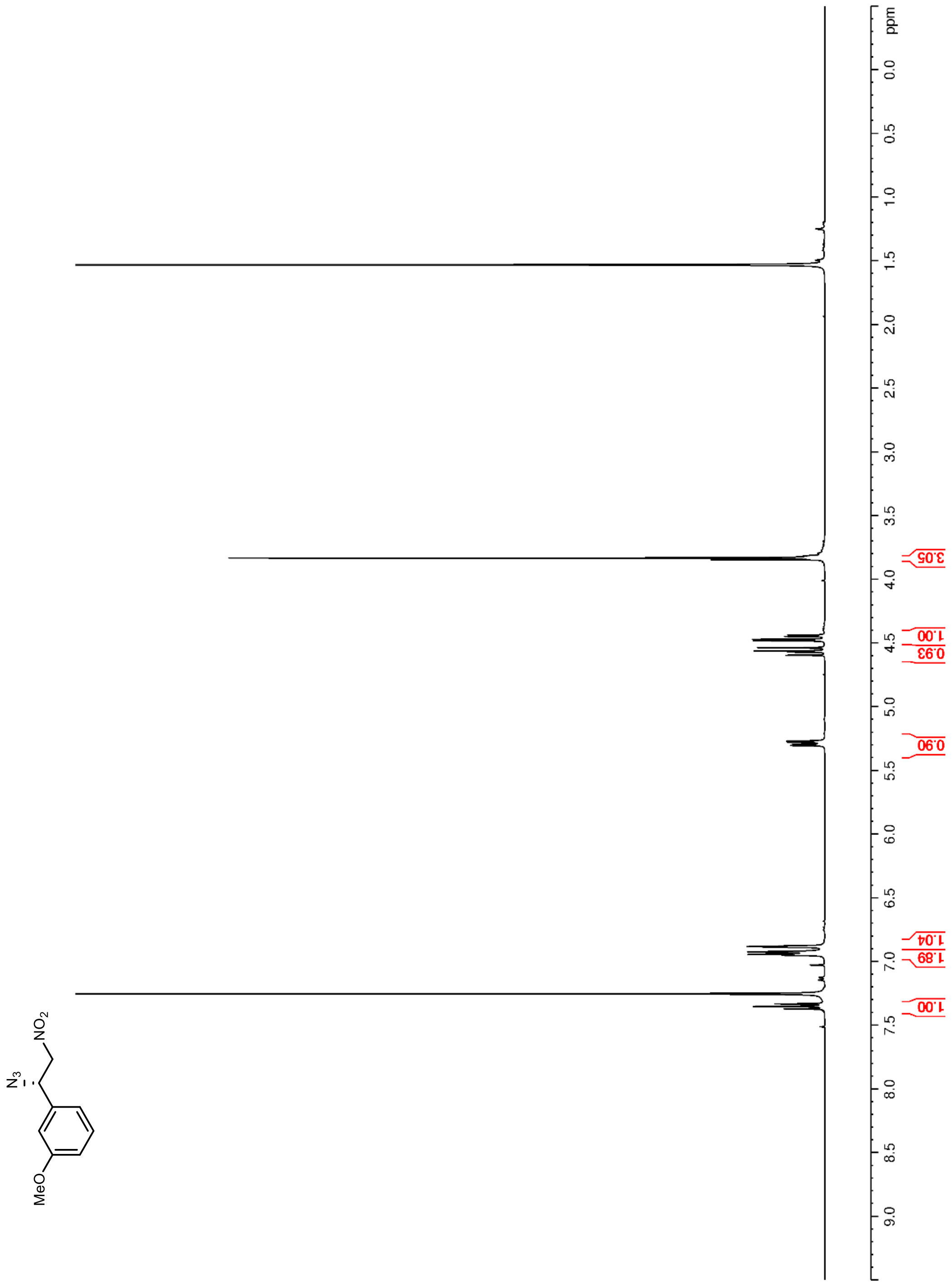

Figure 235. ^{13}C NMR (150 MHz, $CDCl_3$) of **2f**

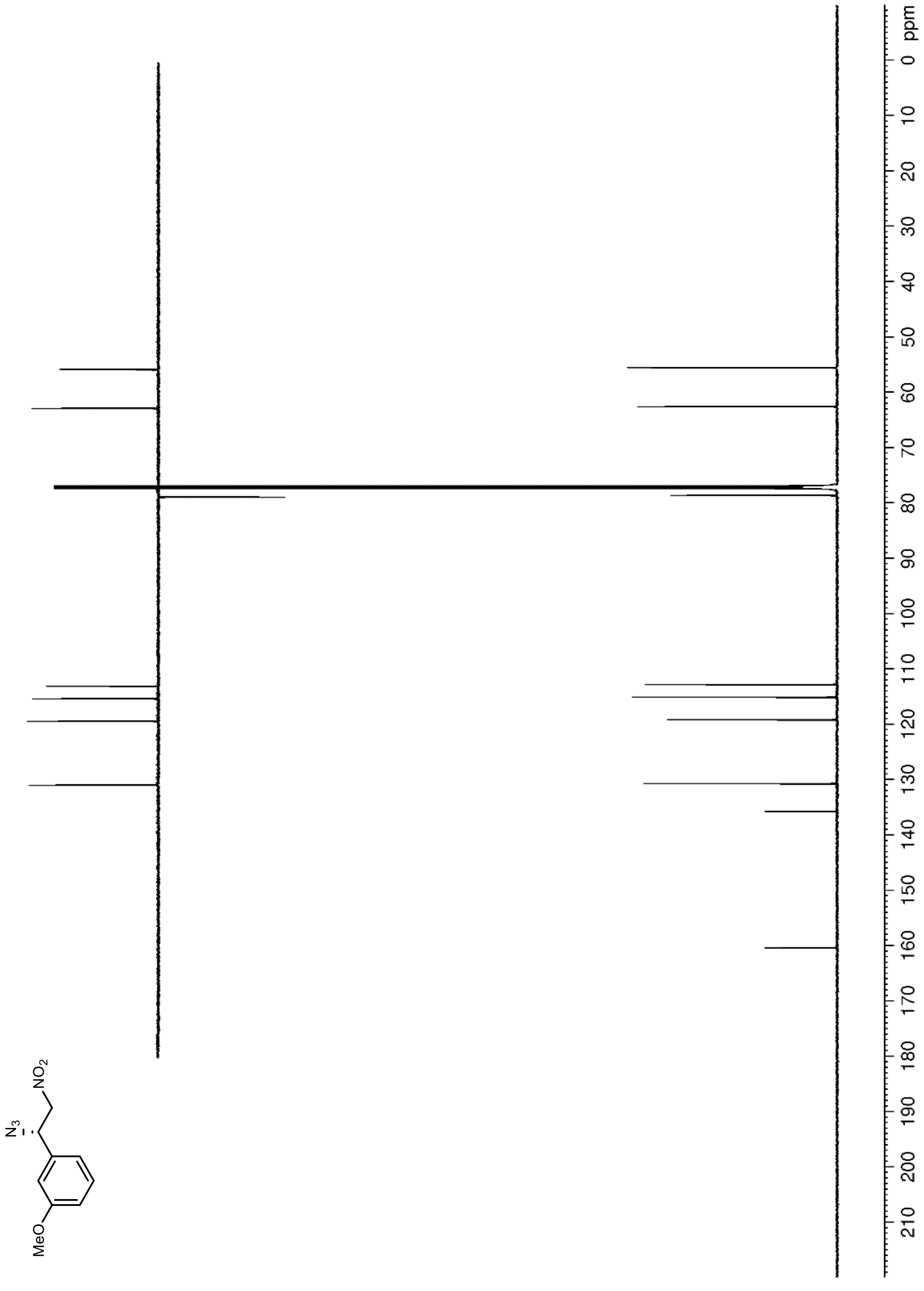

Figure 236. ^{1}H NMR (400 MHz, $CDCl_3$) of **2g**

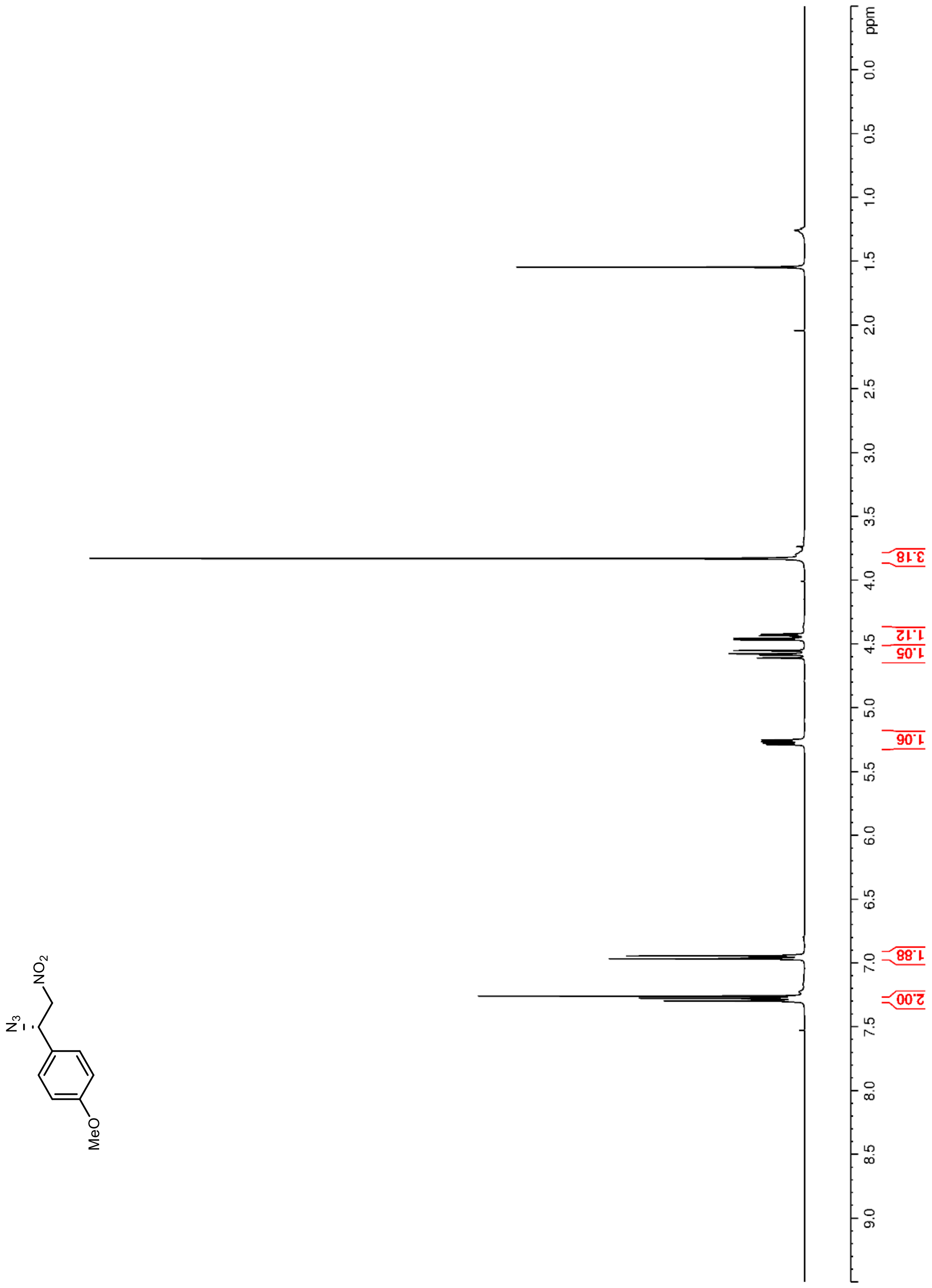

Figure 237. ^{13}C NMR (150 MHz, $CDCl_3$) of **2g**

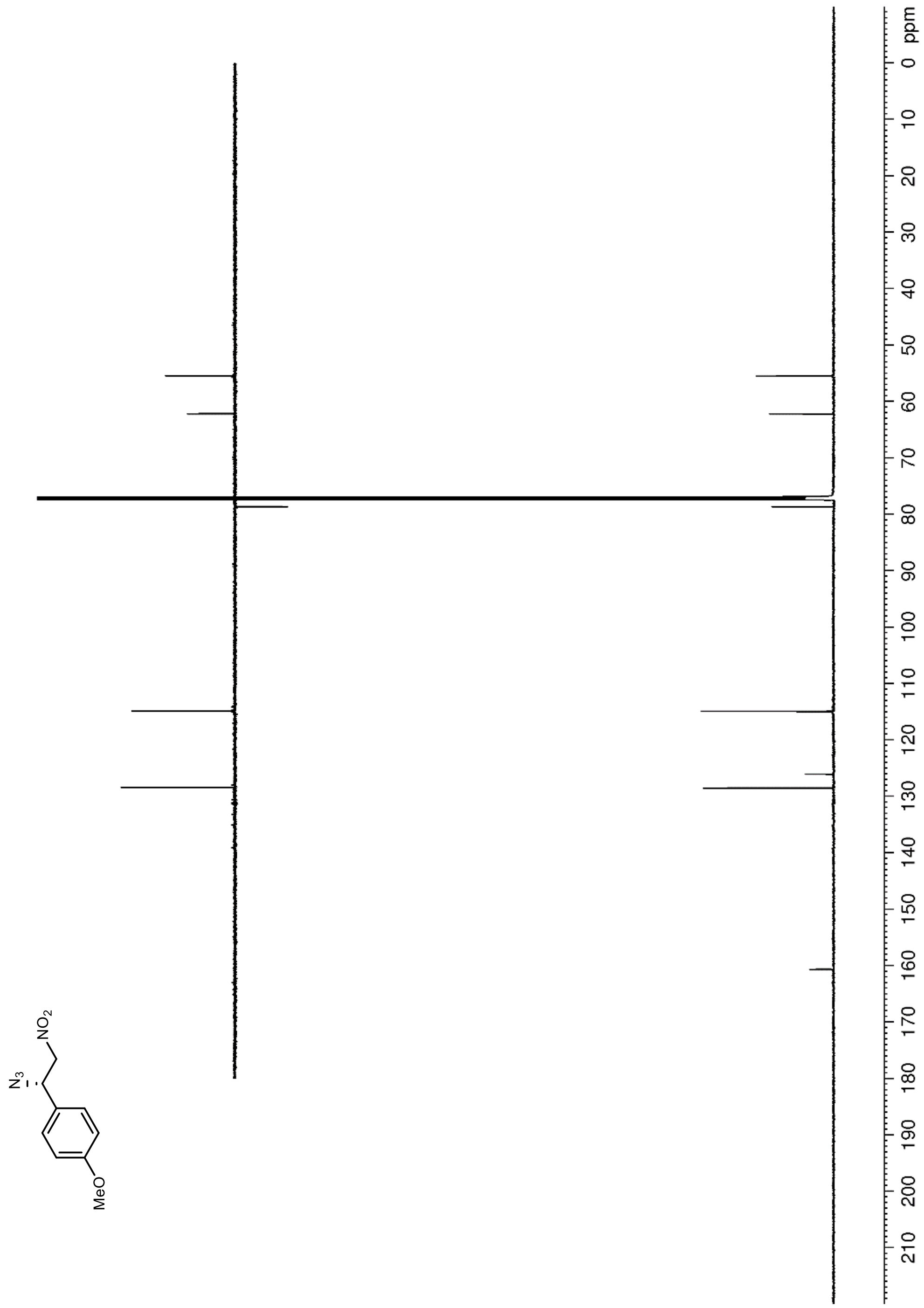

Figure 238. ^{1}H NMR (400 MHz, $CDCl_3$) of **2h**

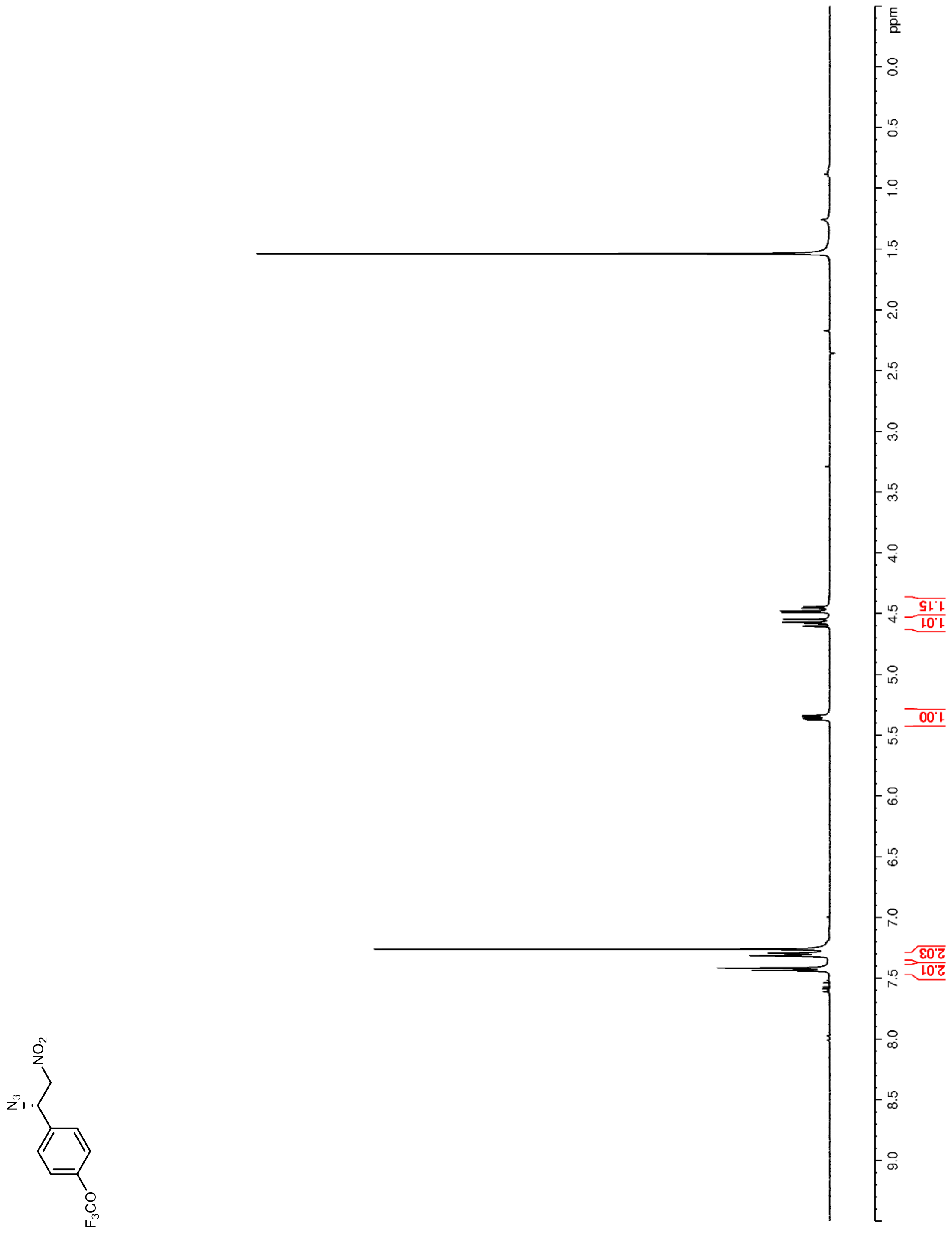

Figure 239. ^{13}C NMR (150 MHz, $CDCl_3$) of **2h**

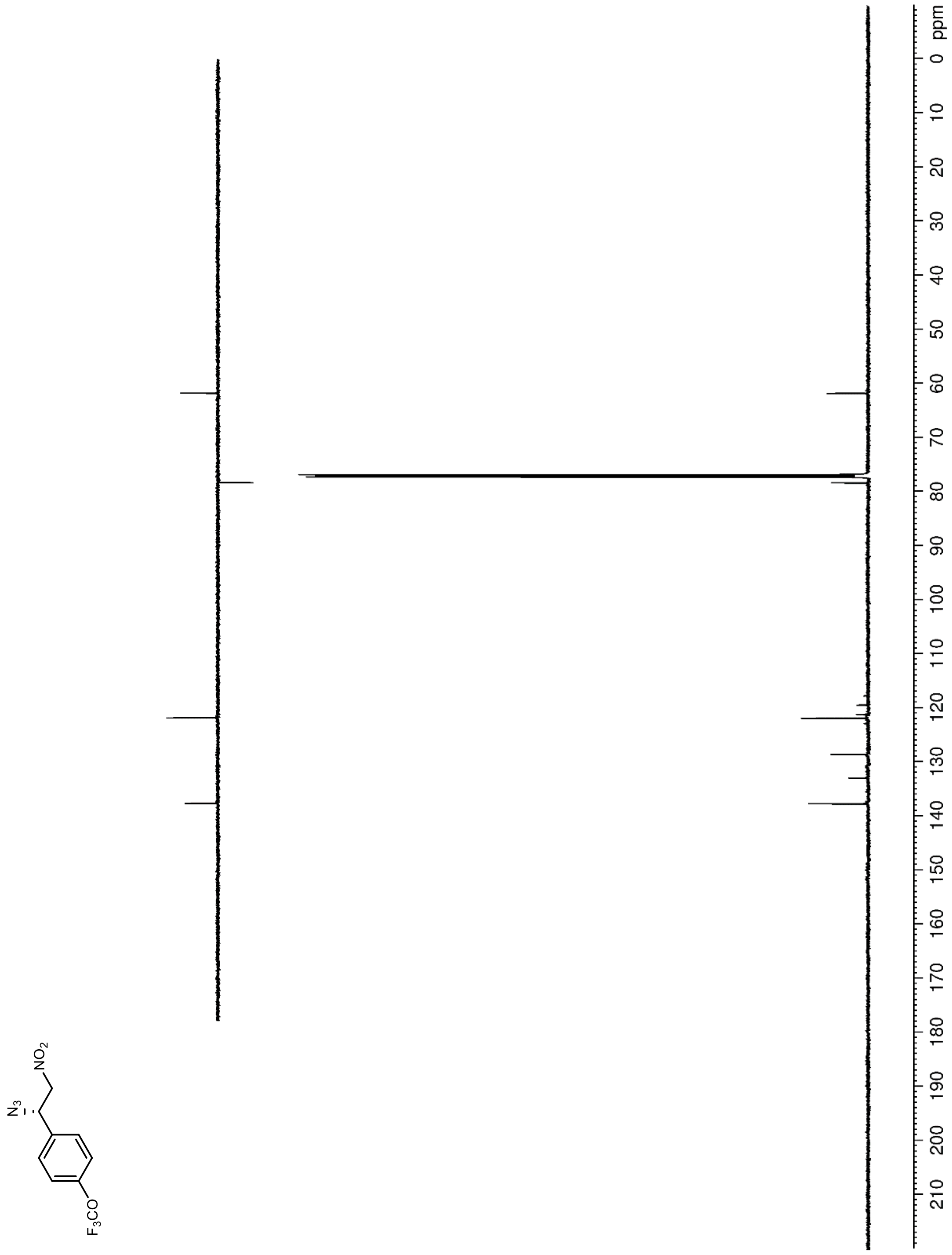

Figure 240. ^{19}F NMR **(**282 MHz, $CDCl_3$**)** of **2h**

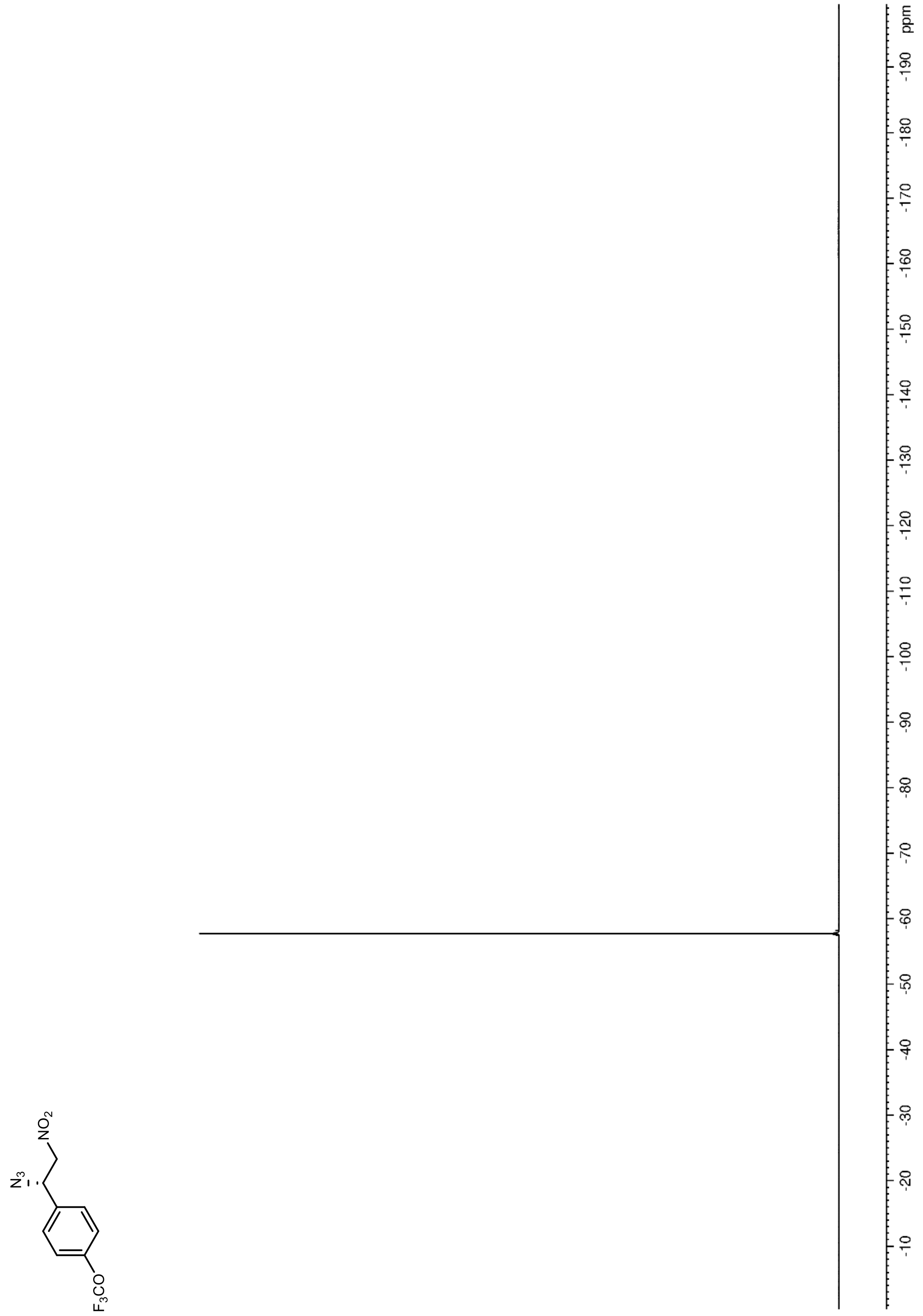

Figure 241. ^{1}H NMR (400 MHz, $CDCl_3$) of **2i**

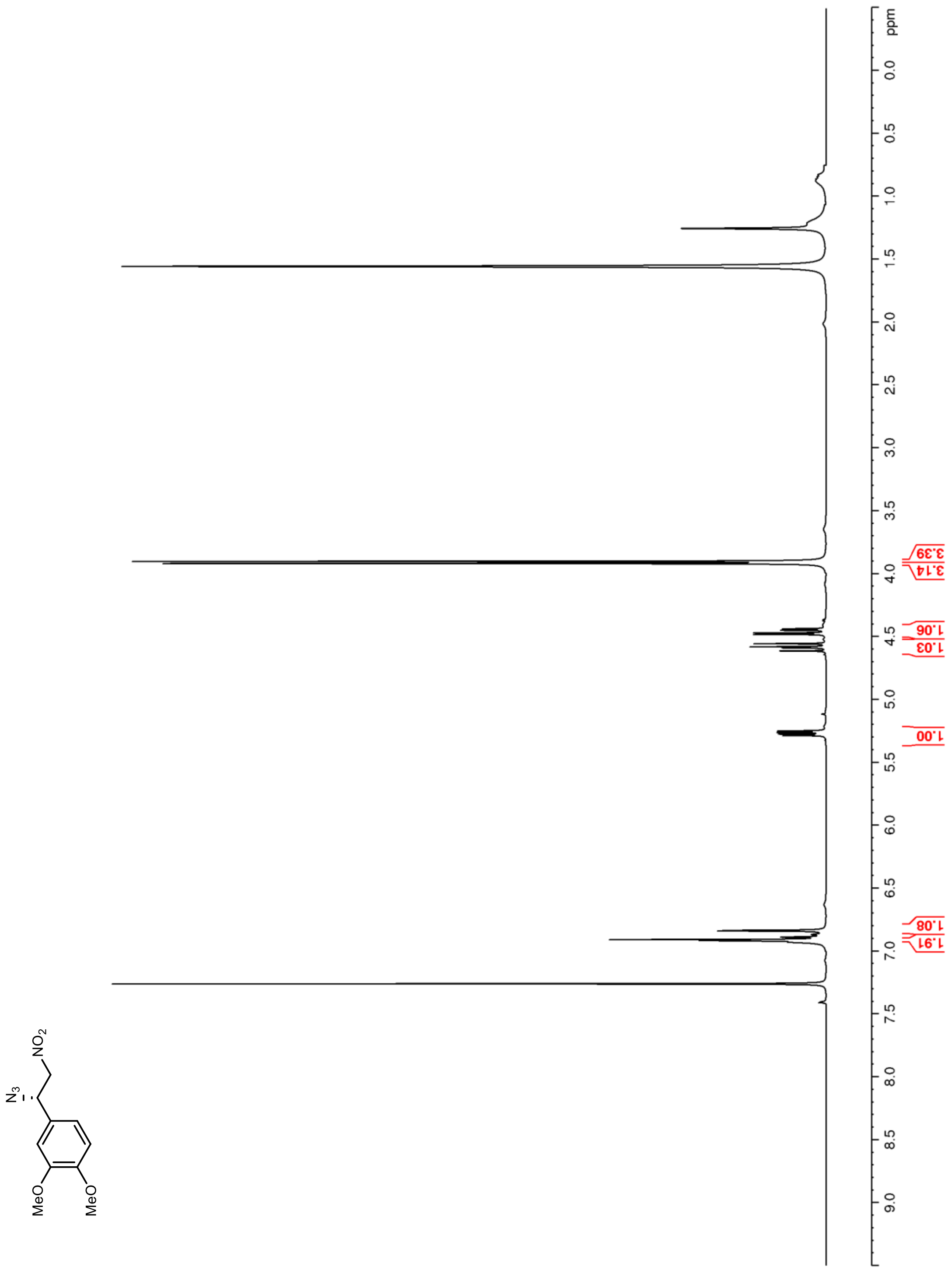

Figure 242. ^{13}C NMR (150 MHz, $CDCl_3$) of **2i**

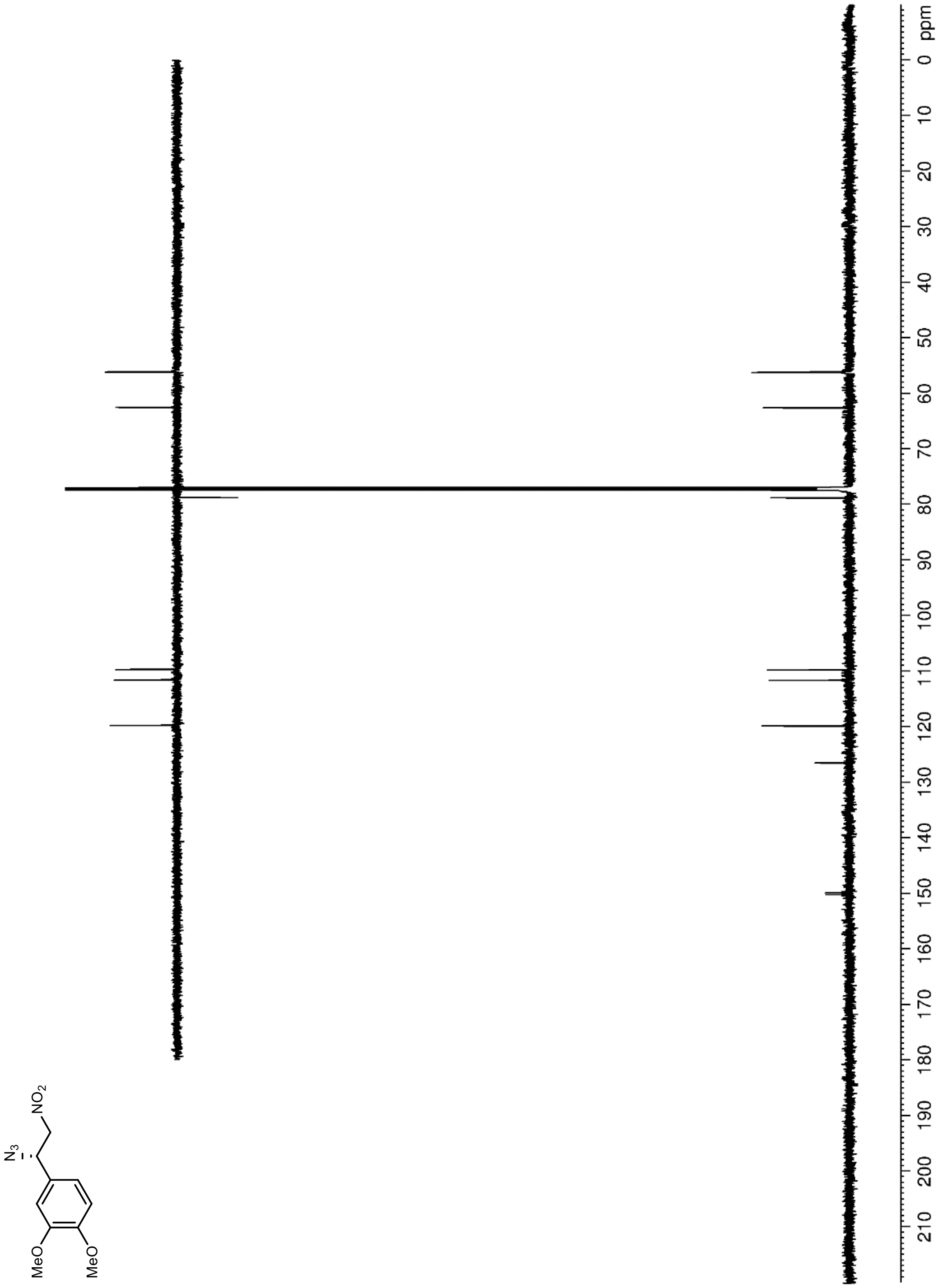

Figure 243. ^{1}H NMR (400 MHz, $CDCl_3$) of **2j**

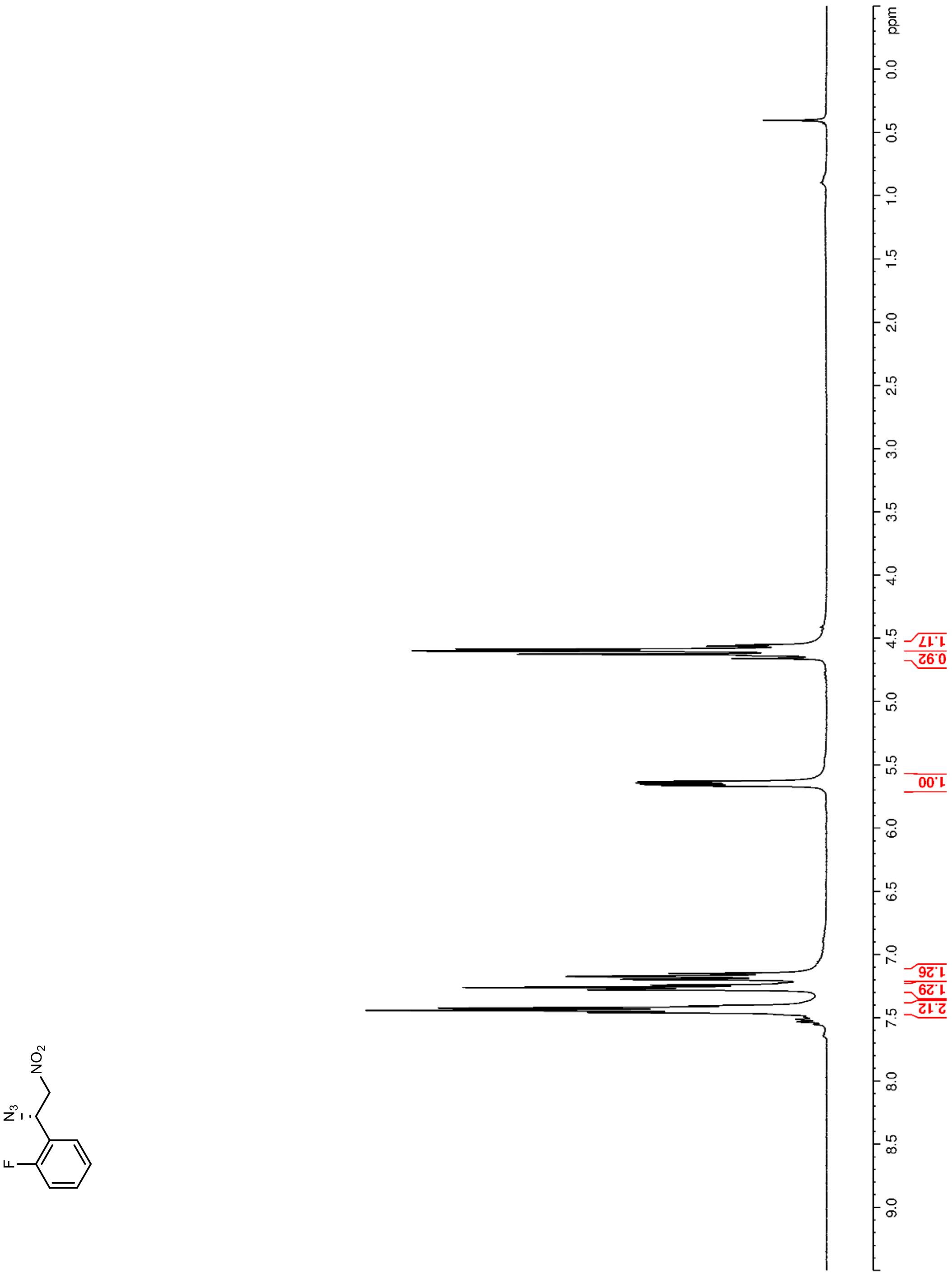

Figure 244. ^{13}C NMR (150 MHz, $CDCl_3$) of **2j**

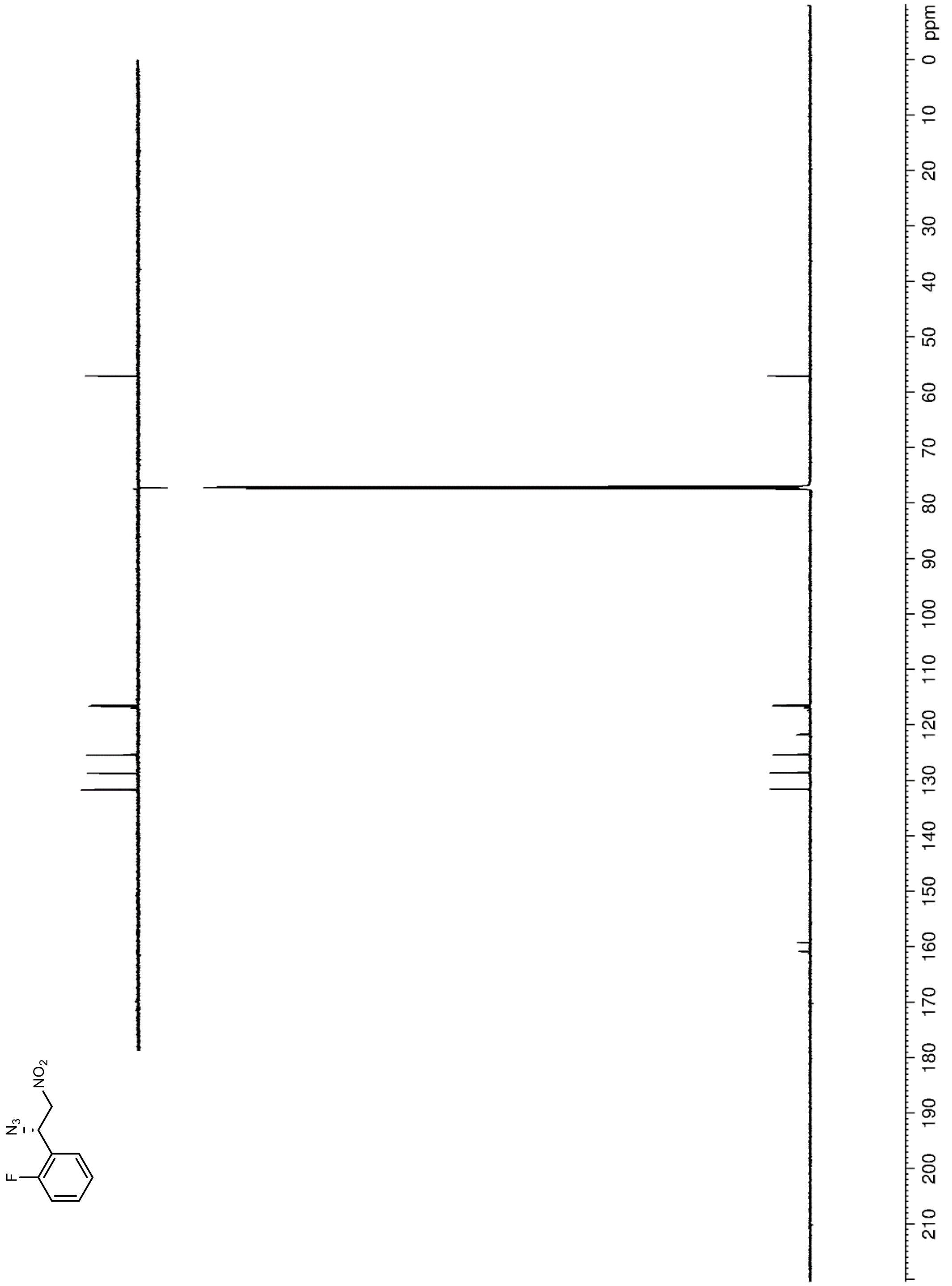

Figure 245. ^{19}F NMR **(**282 MHz, $CDCl_3$**)** of **2j**

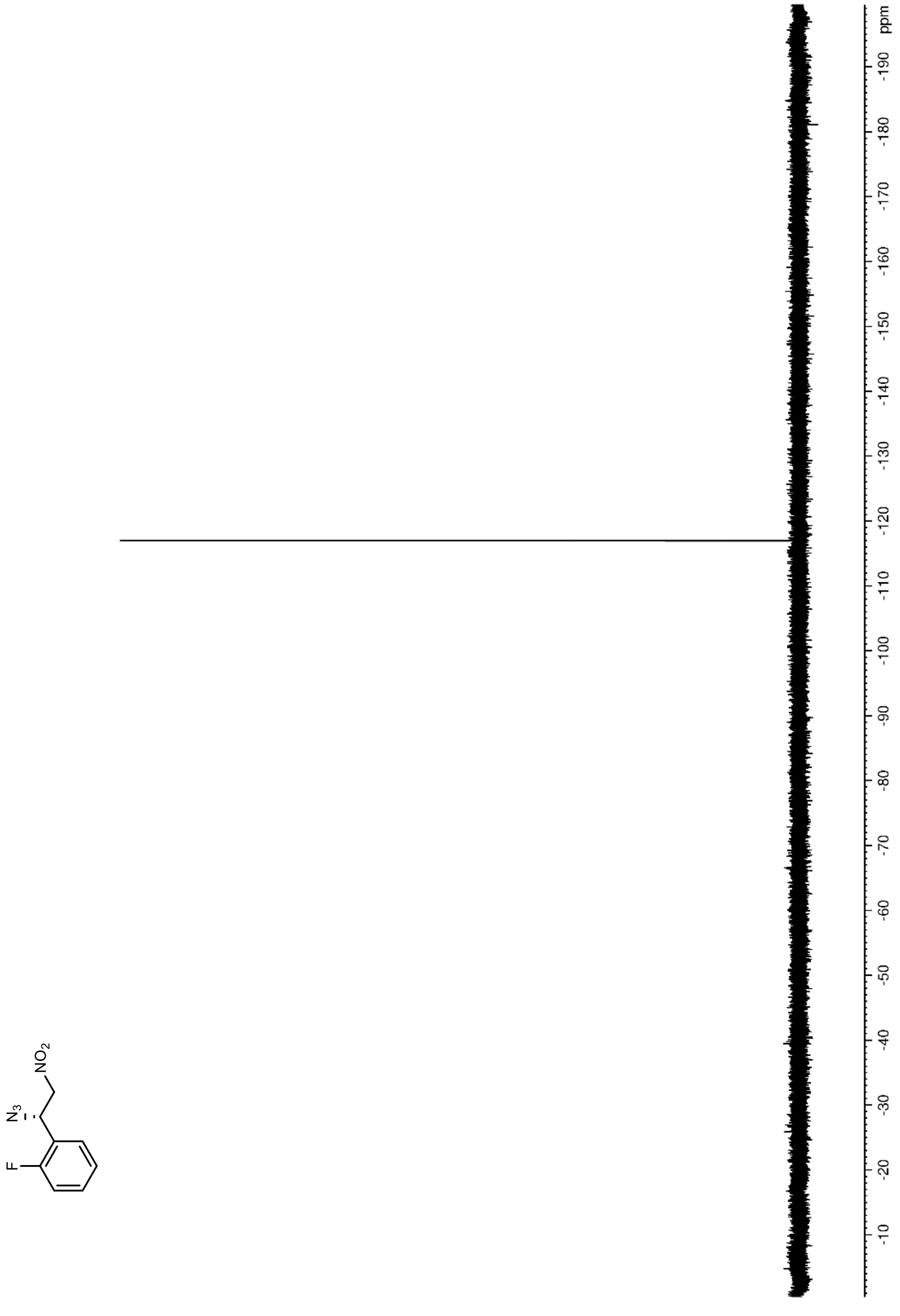

Figure 246. ^{1}H NMR (400 MHz, $CDCl_3$) of **2k**

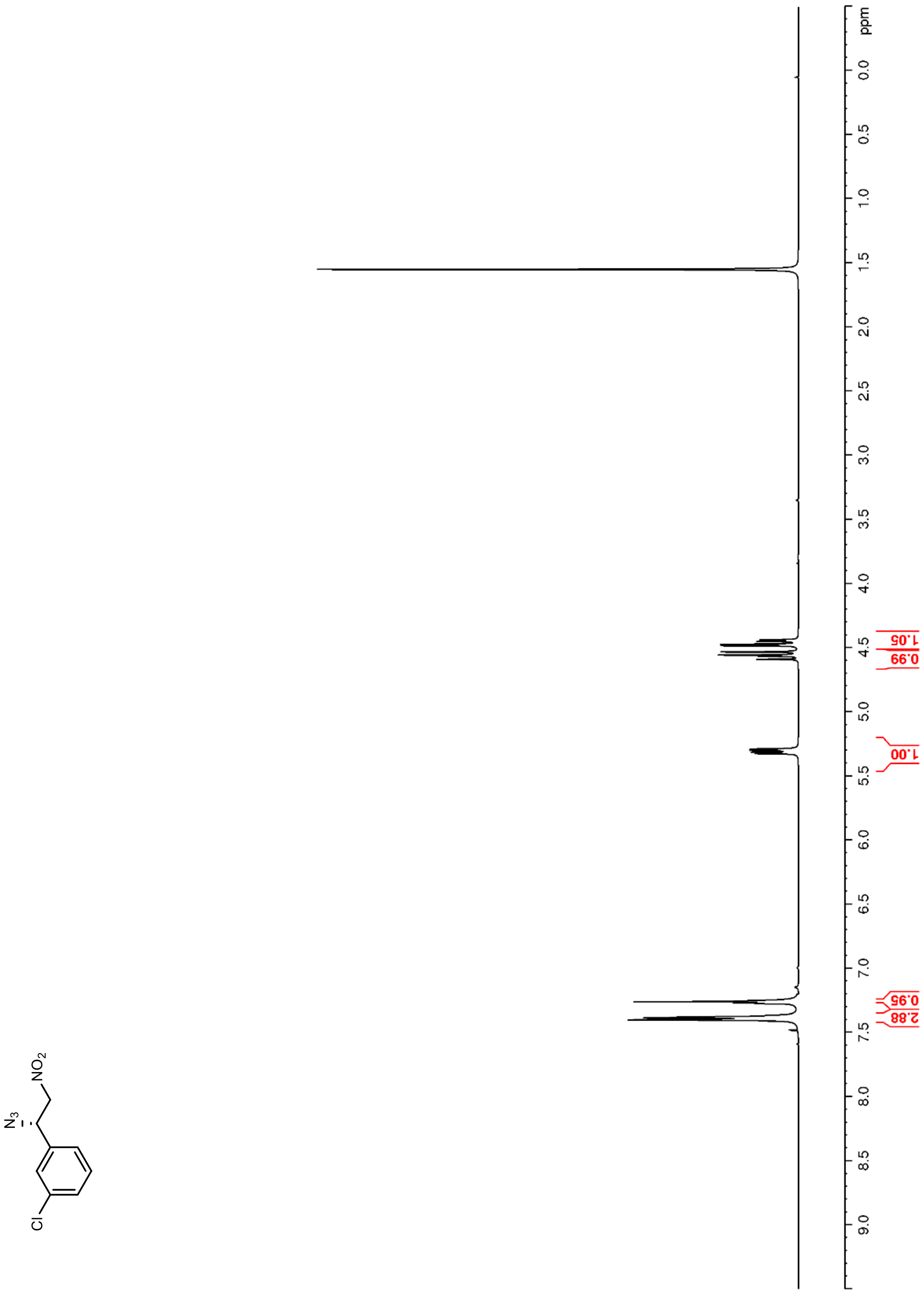

Figure 247. ^{13}C NMR (150 MHz, $CDCl_3$) of **2k**

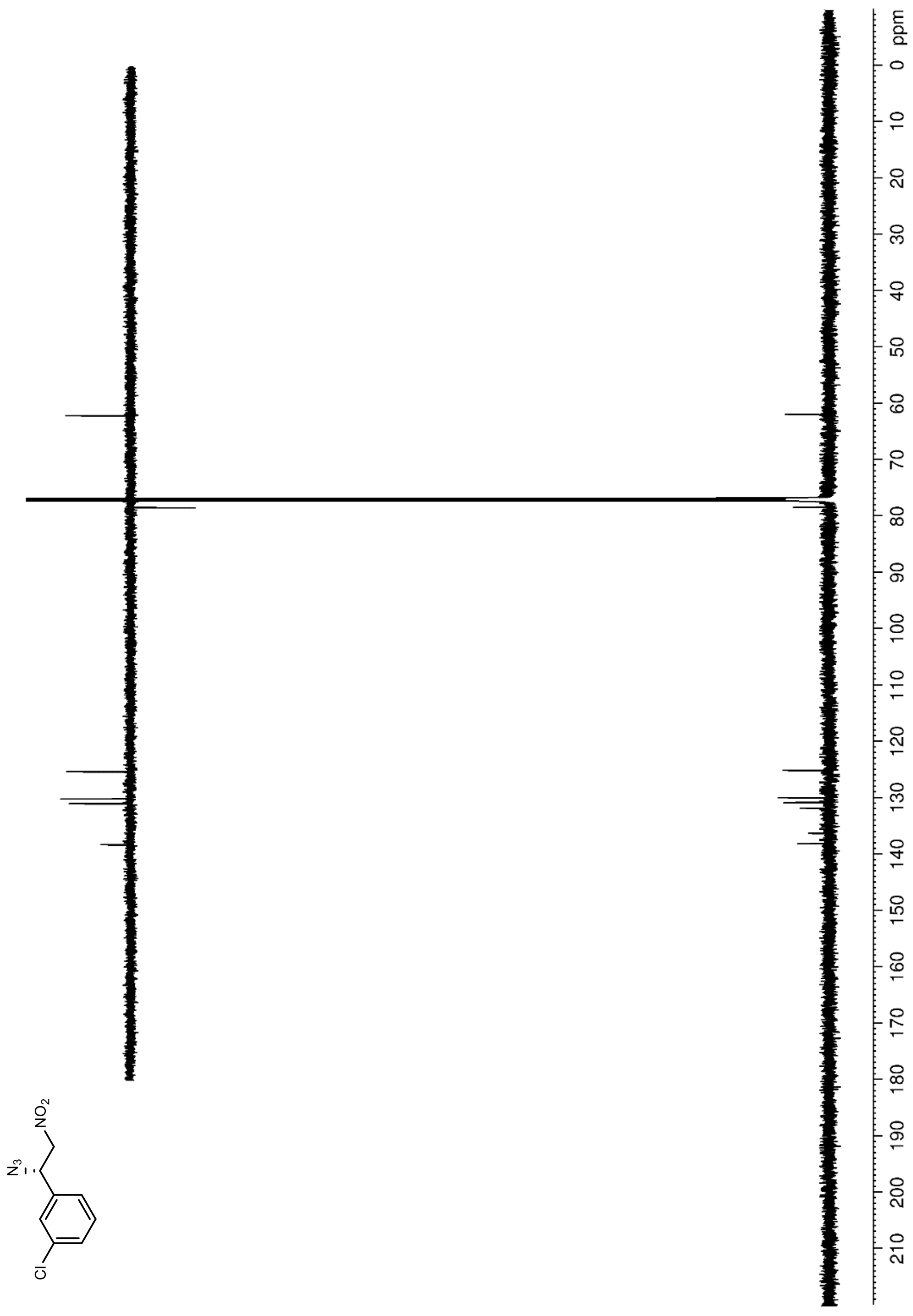

Figure 248. ^{1}H NMR (400 MHz, $CDCl_3$) of **2l**

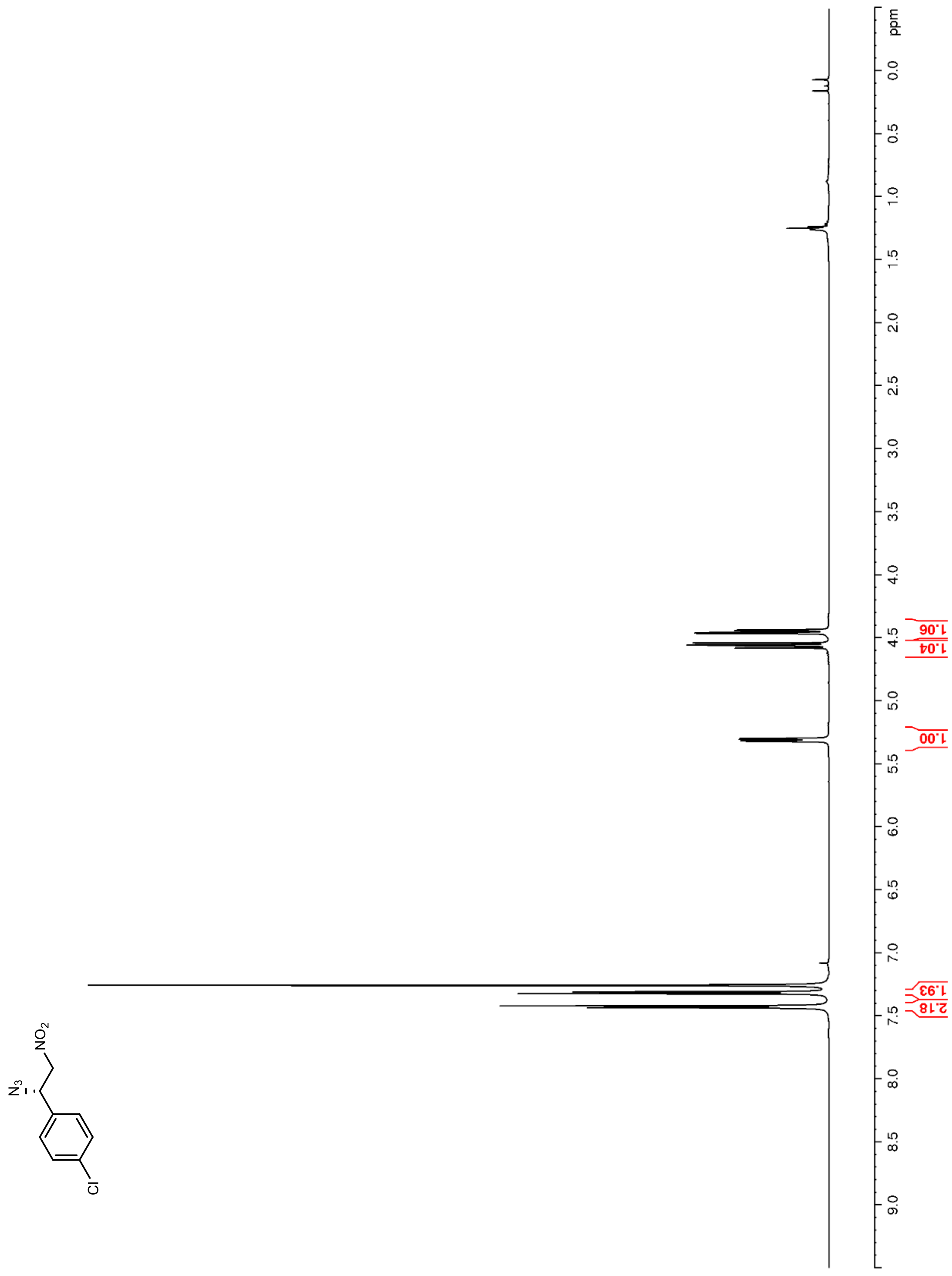

Figure 249. ^{13}C NMR (150 MHz, $CDCl_3$) of **2l**

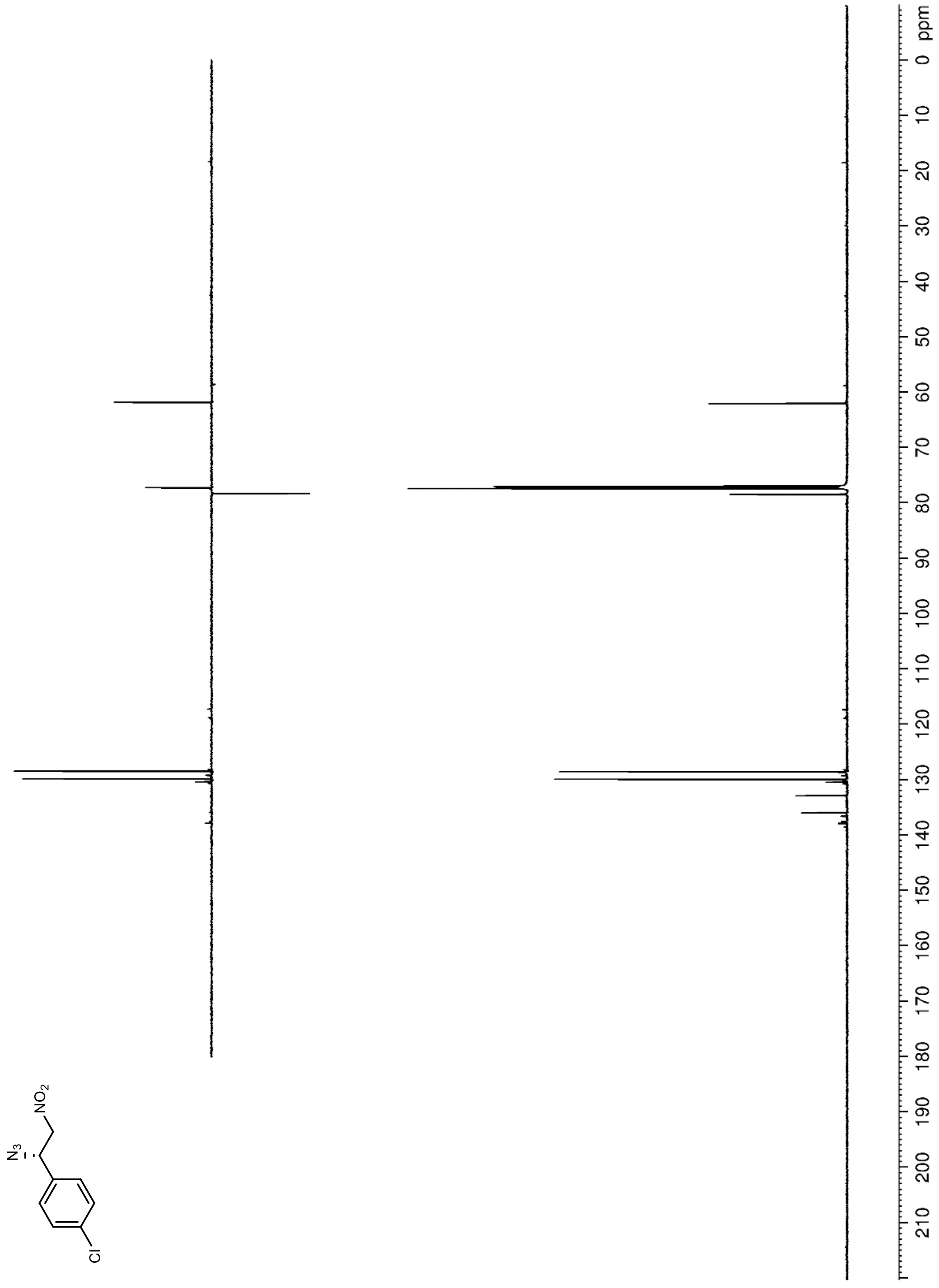

Figure 250. ^{1}H NMR (400 MHz, $CDCl_3$) of **2m**

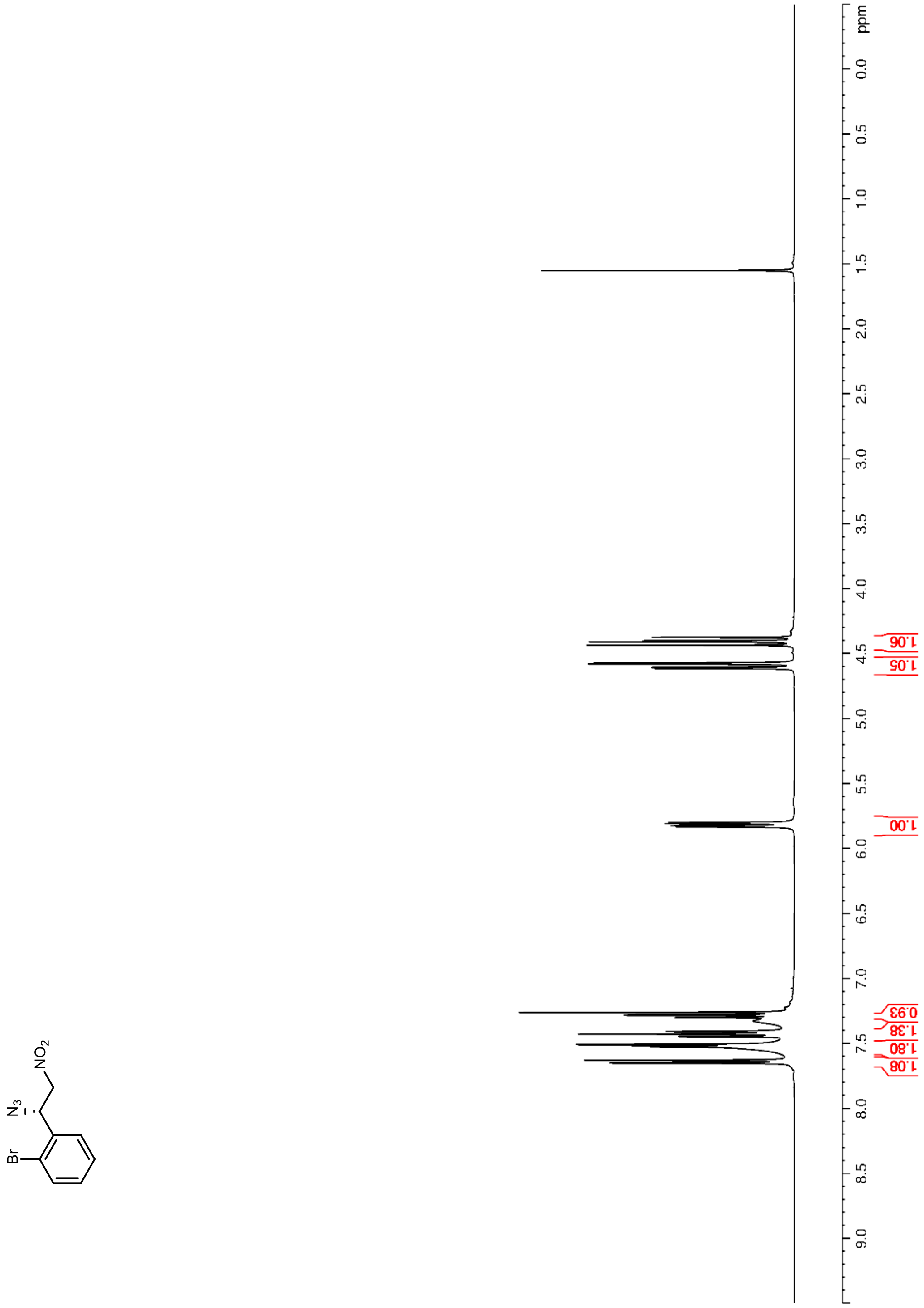

Figure 251. ^{13}C NMR (150 MHz, $CDCl_3$) of **2m**

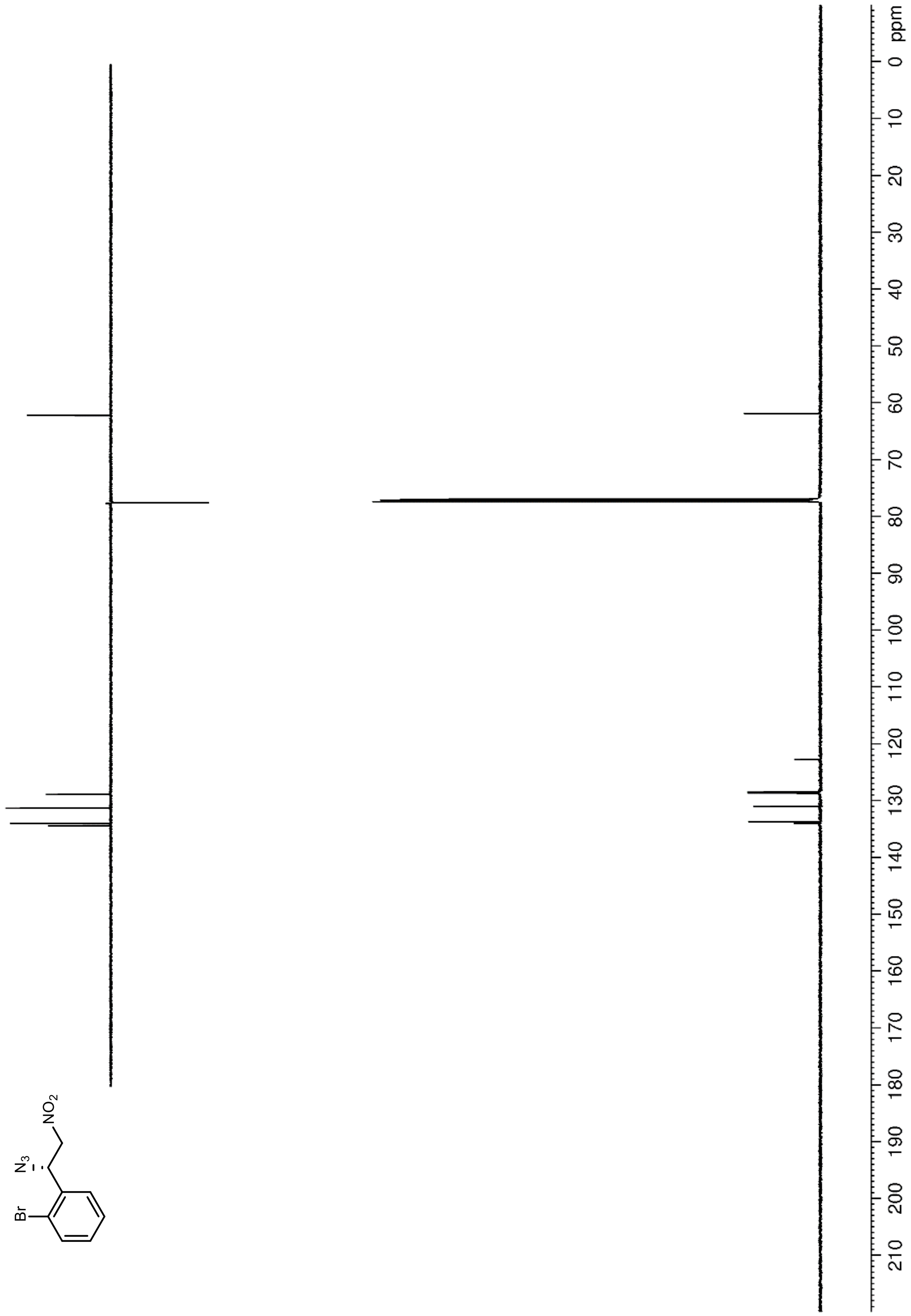

Figure 252. ^{1}H NMR (400 MHz, $CDCl_3$) of **2n**

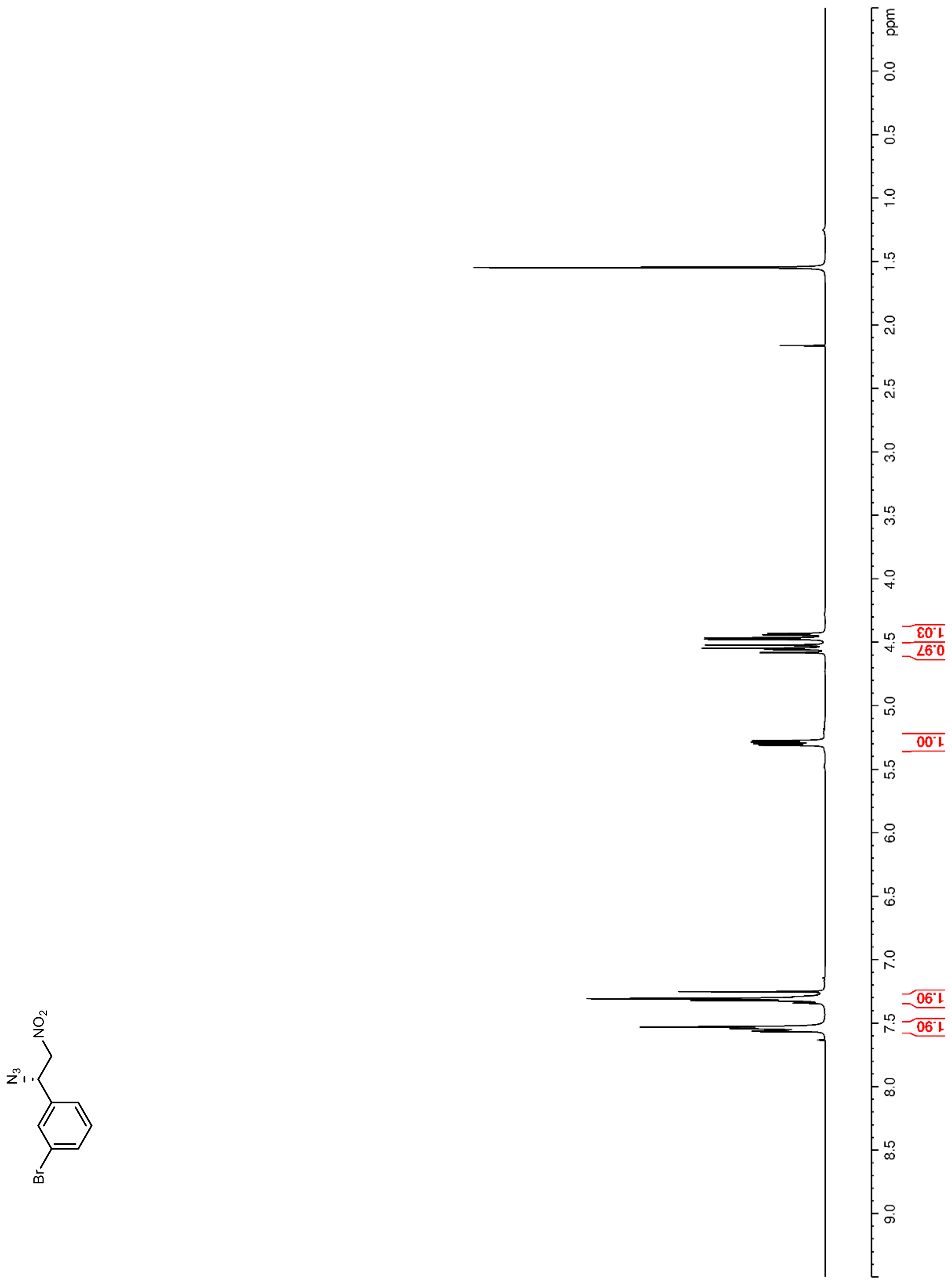

Figure 253. ^{13}C NMR (150 MHz, $CDCl_3$) of **2n**

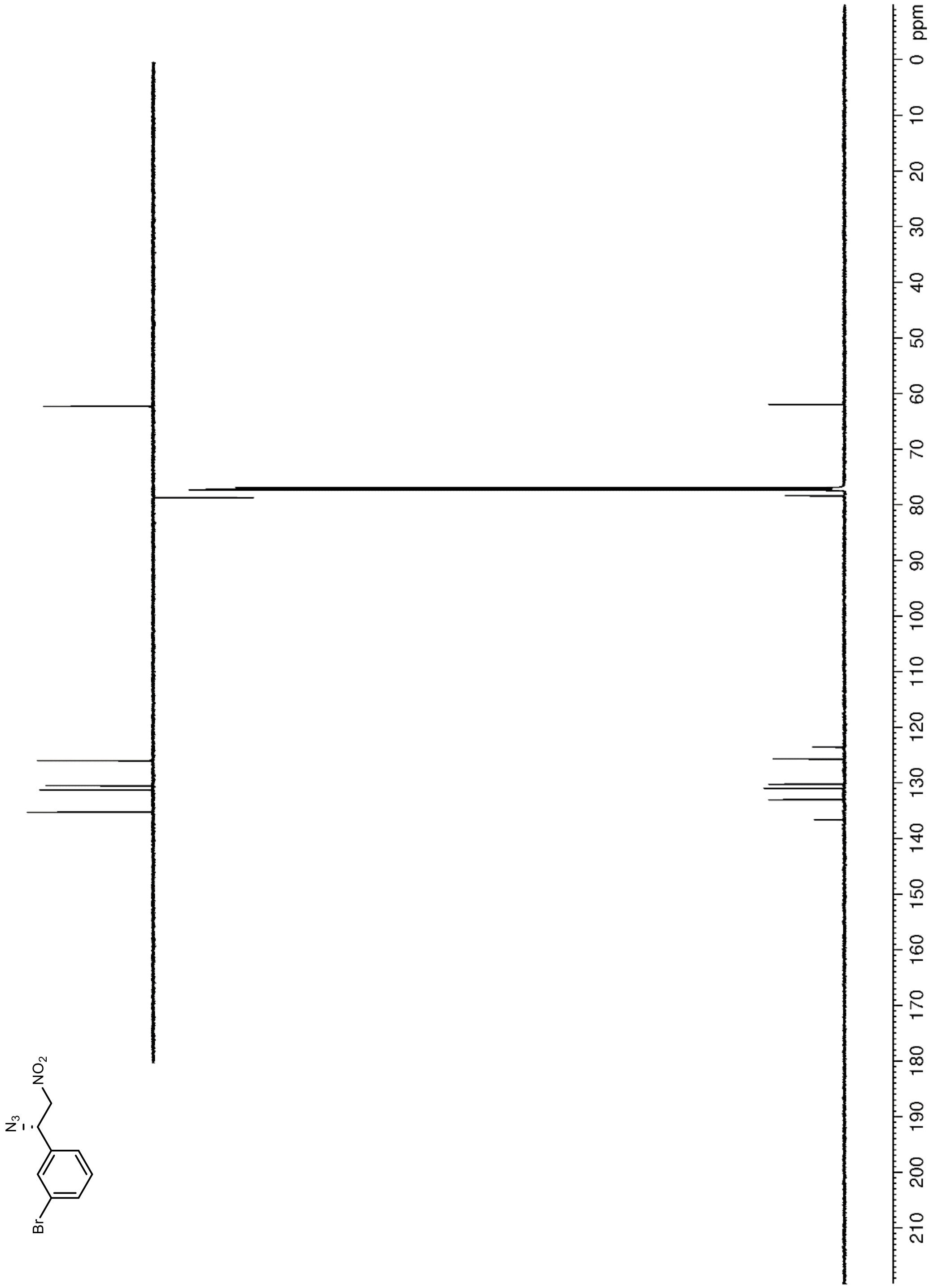

Figure 254. ^{1}H NMR (400 MHz, $CDCl_3$) of **2o**

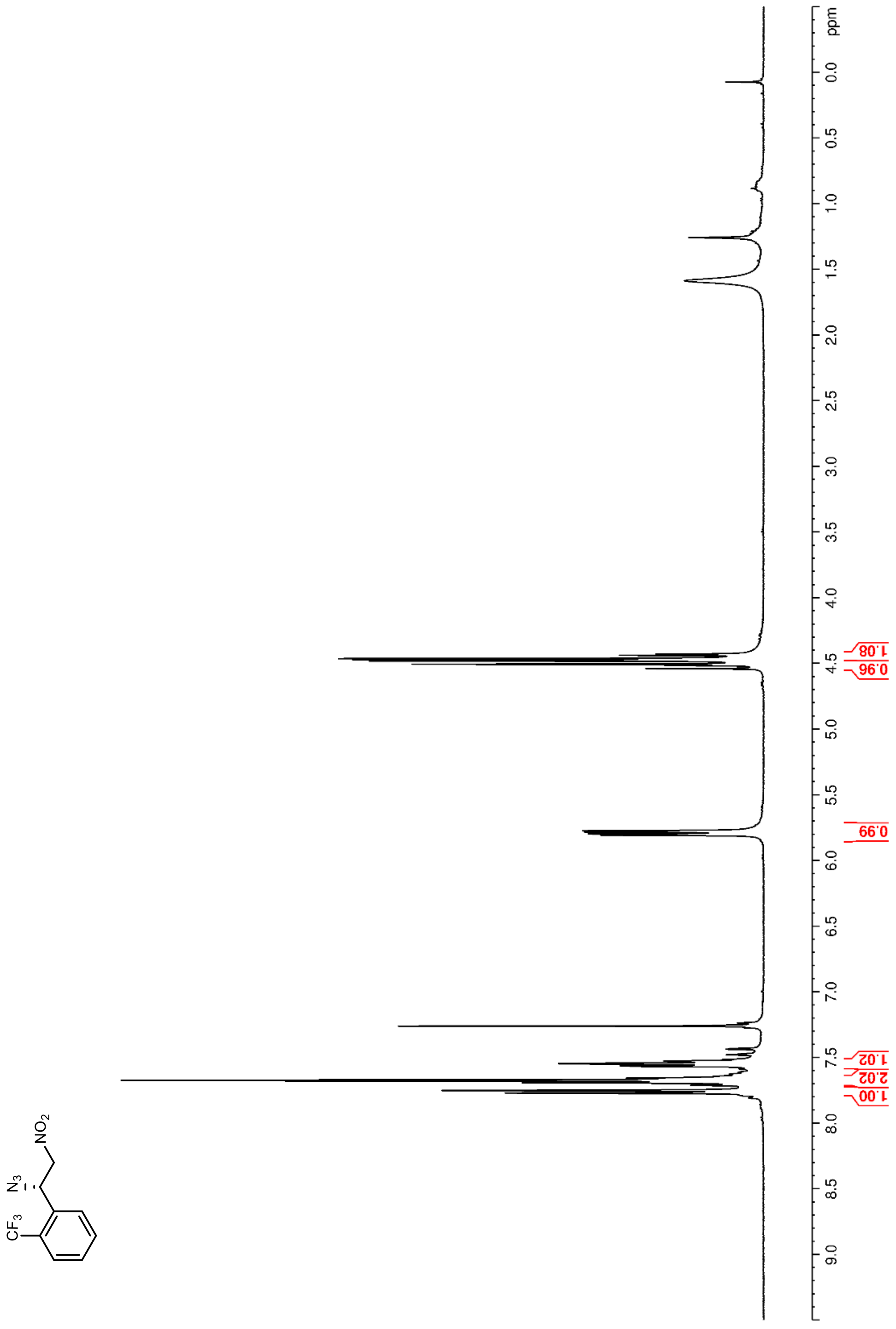

Figure 255. ^{13}C NMR (150 MHz, $CDCl_3$) of **2o**

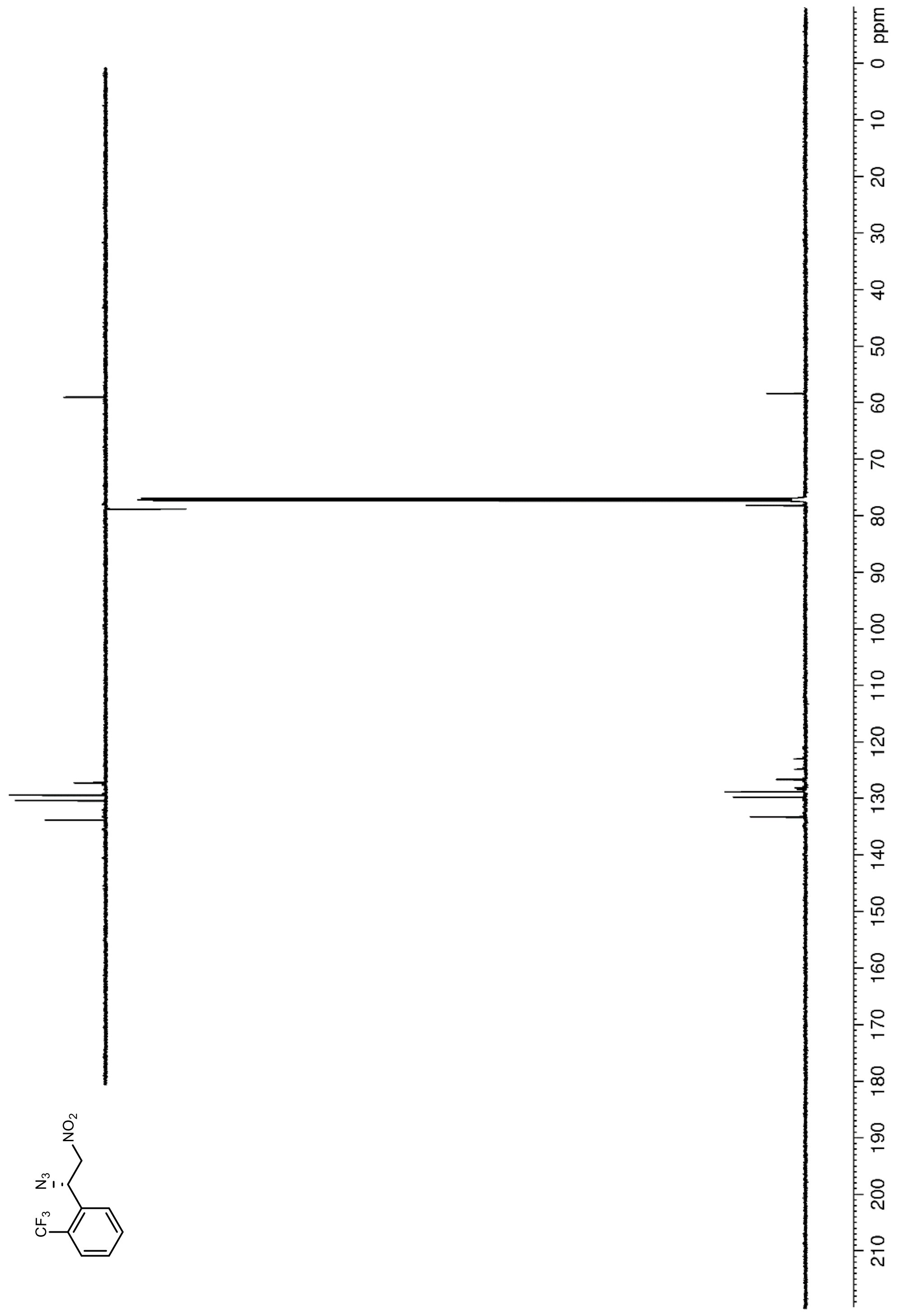

Figure 256. ^{19}F NMR **(**282 MHz, $CDCl_3$**)** of **2o**

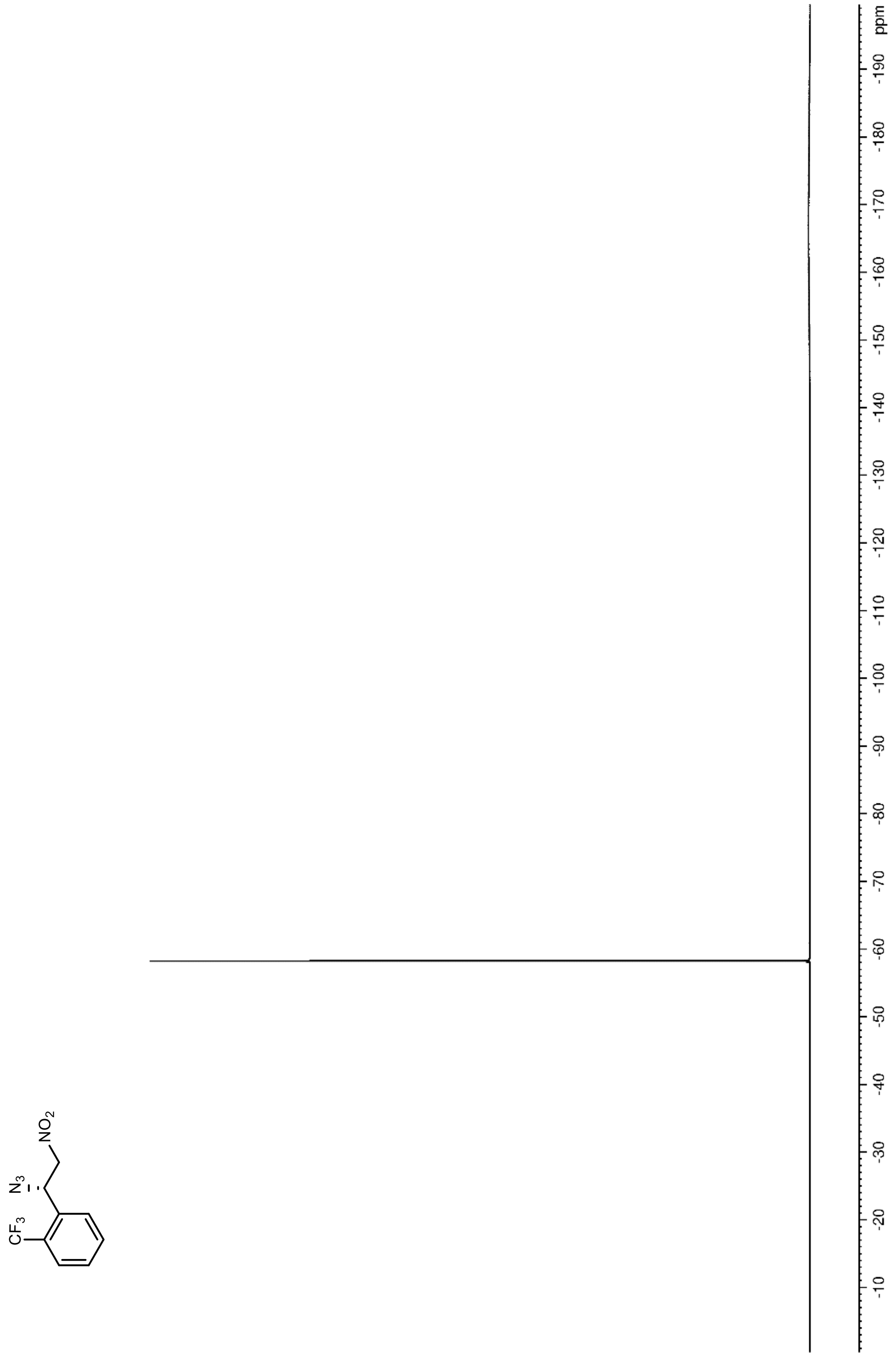

Figure 257. ^{1}H NMR (400 MHz, $CDCl_3$) of **2p**

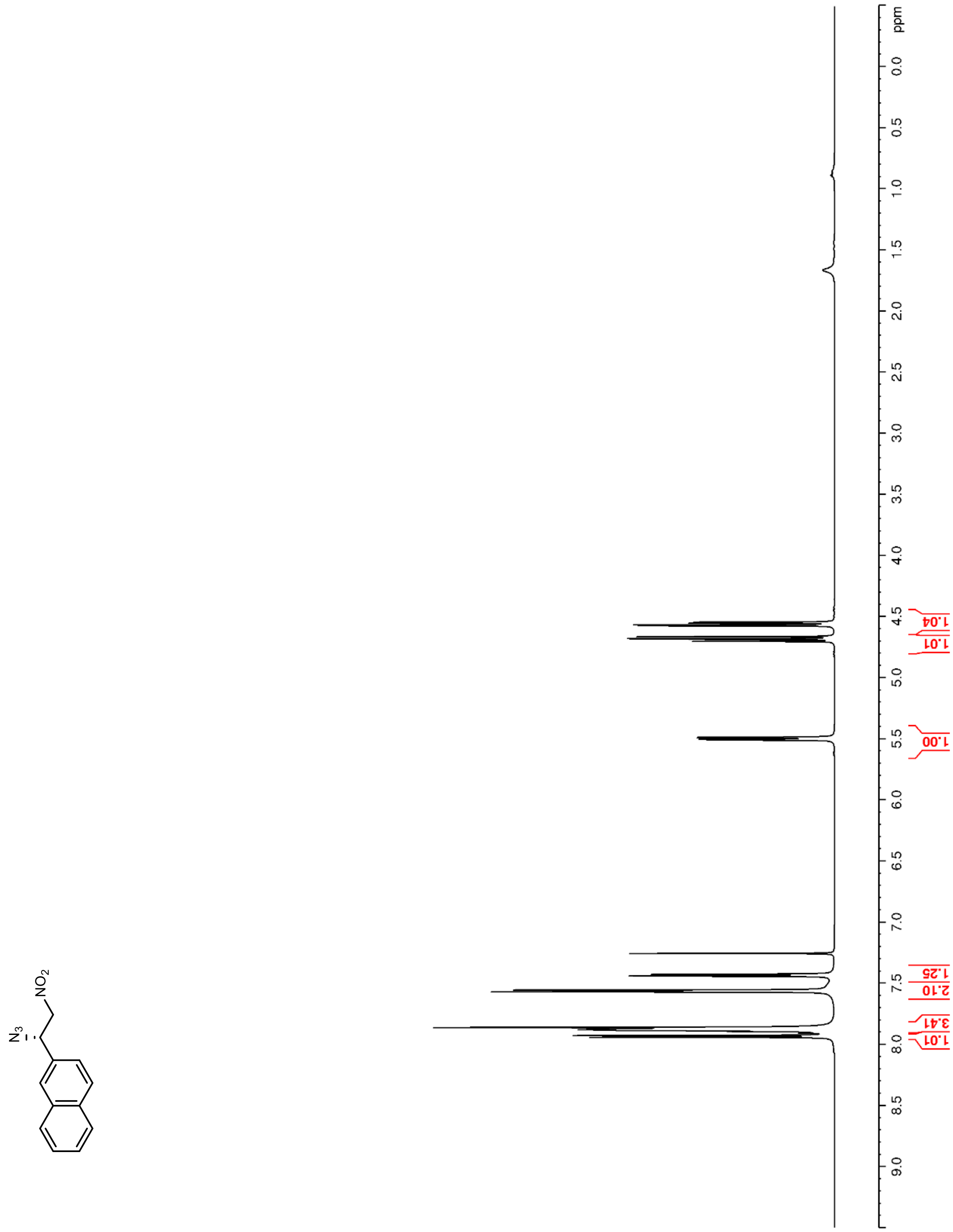

Figure 258. ^{13}C NMR (150 MHz, $CDCl_3$) of **2p**

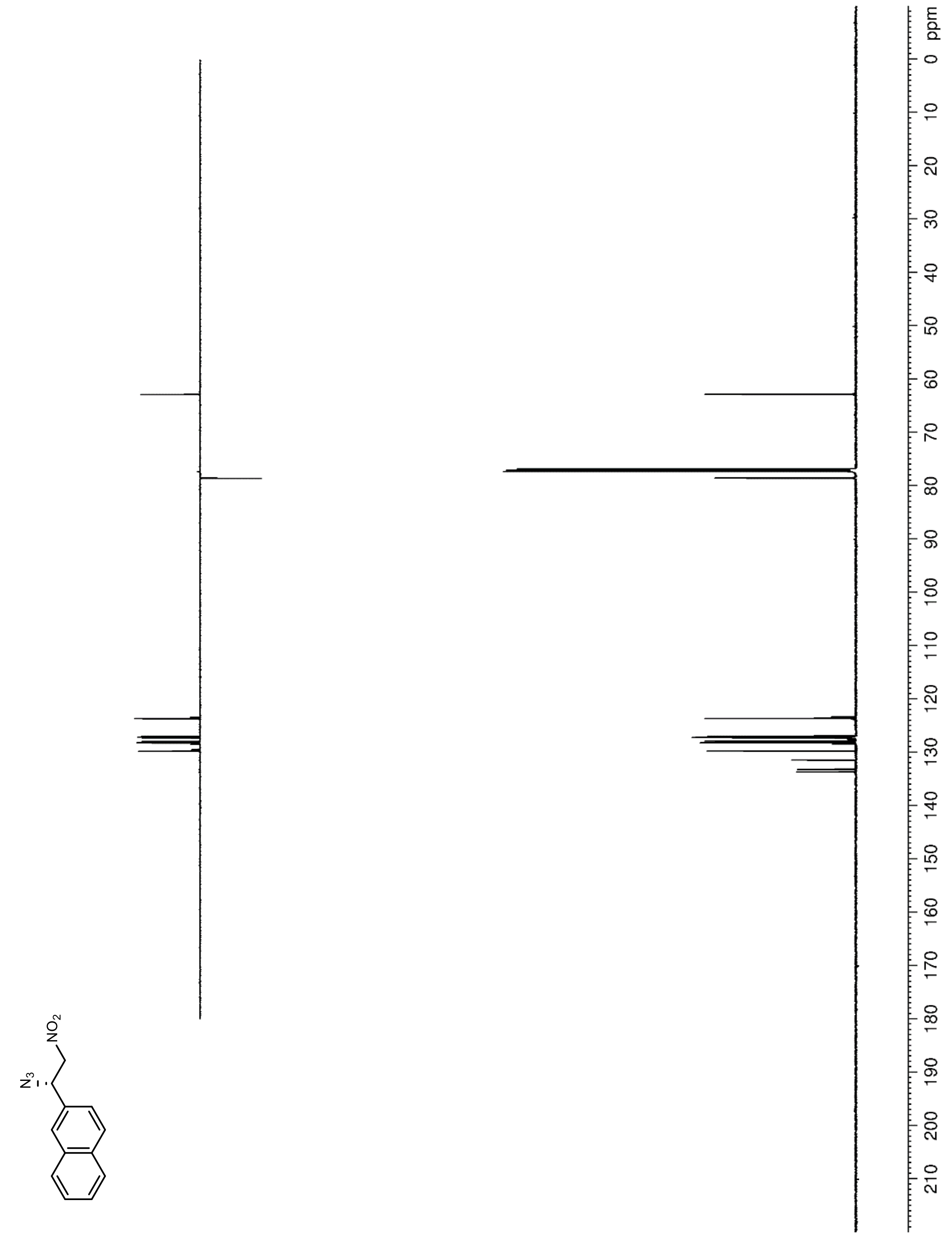

Figure 259. ^{1}H NMR (400 MHz, $CDCl_3$) of **2q**

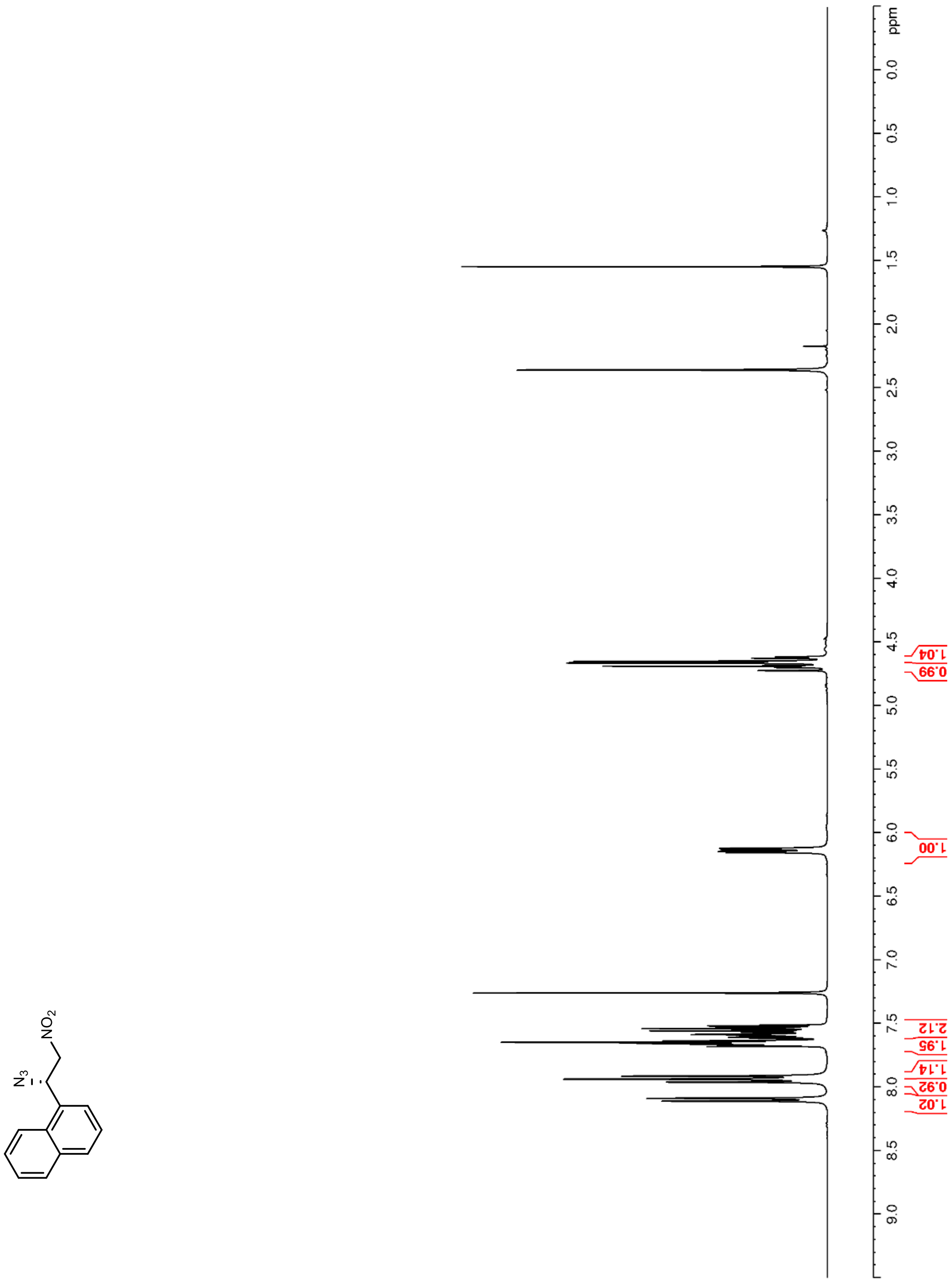

Figure 260. ^{13}C NMR (150 MHz, $CDCl_3$) of **2q**

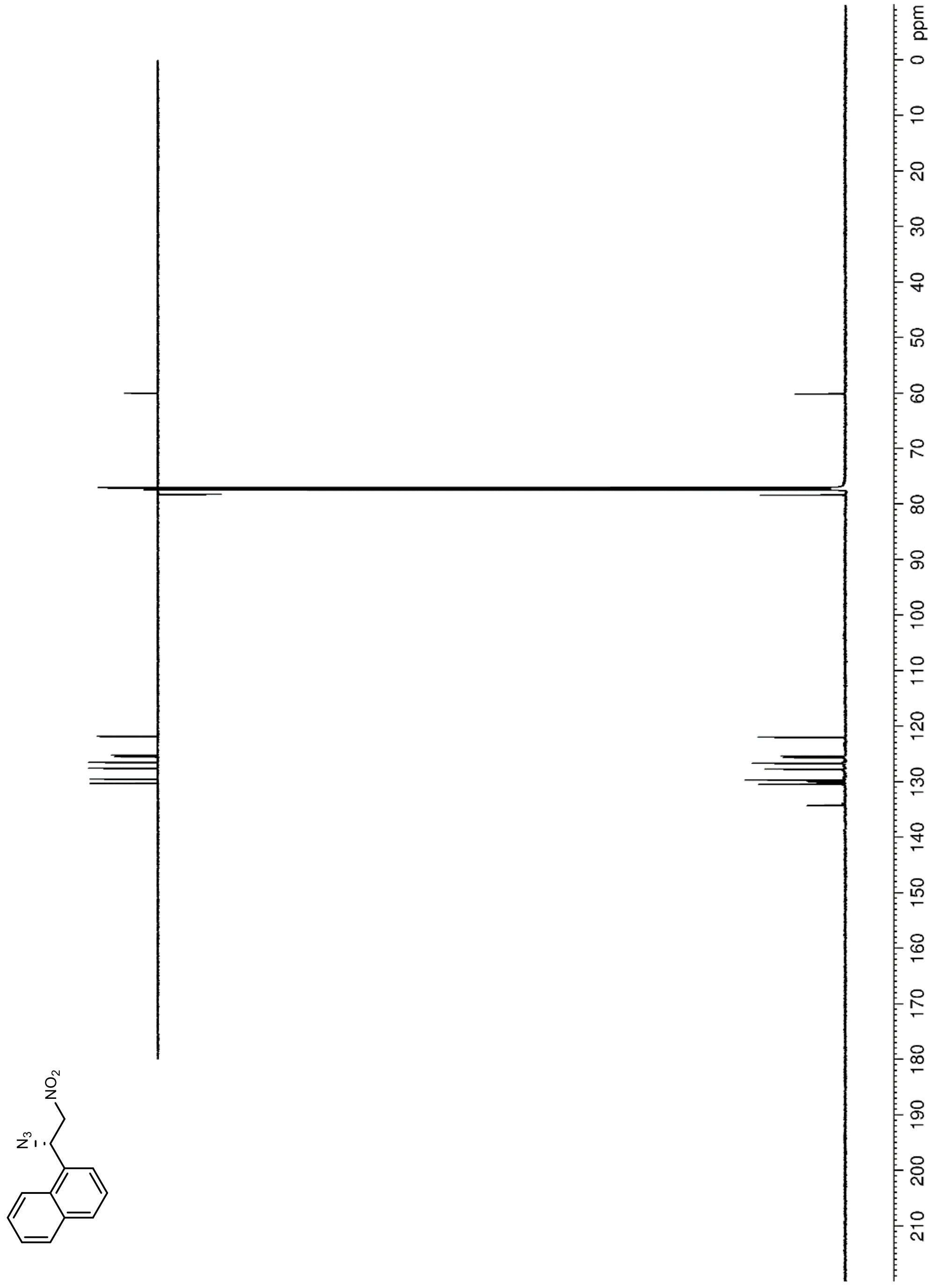

Figure 261. ^{1}H NMR (400 MHz, $CDCl_3$) of **2r**

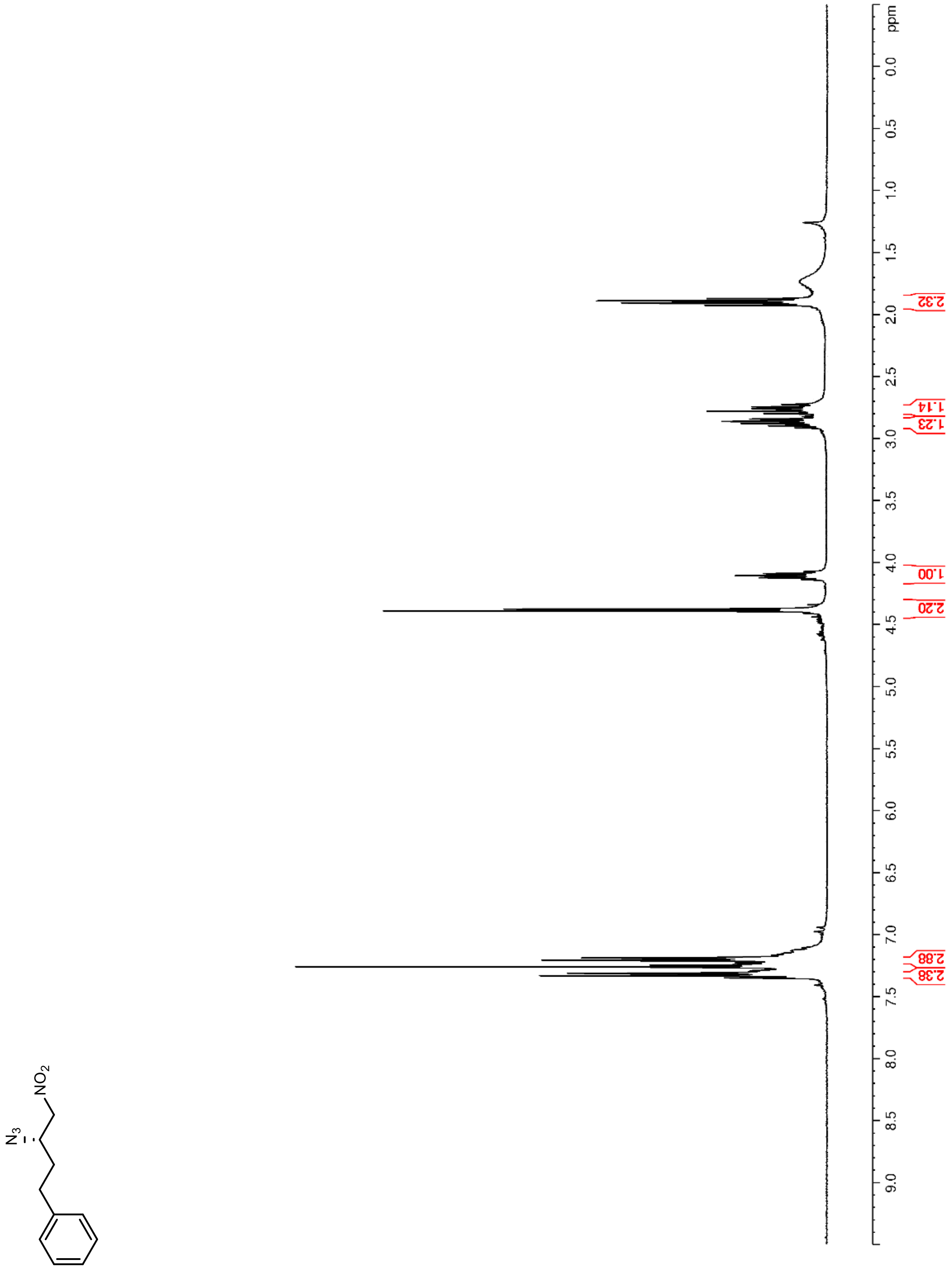

Figure 262. ^{13}C NMR (150 MHz, $CDCl_3$) of **2r**

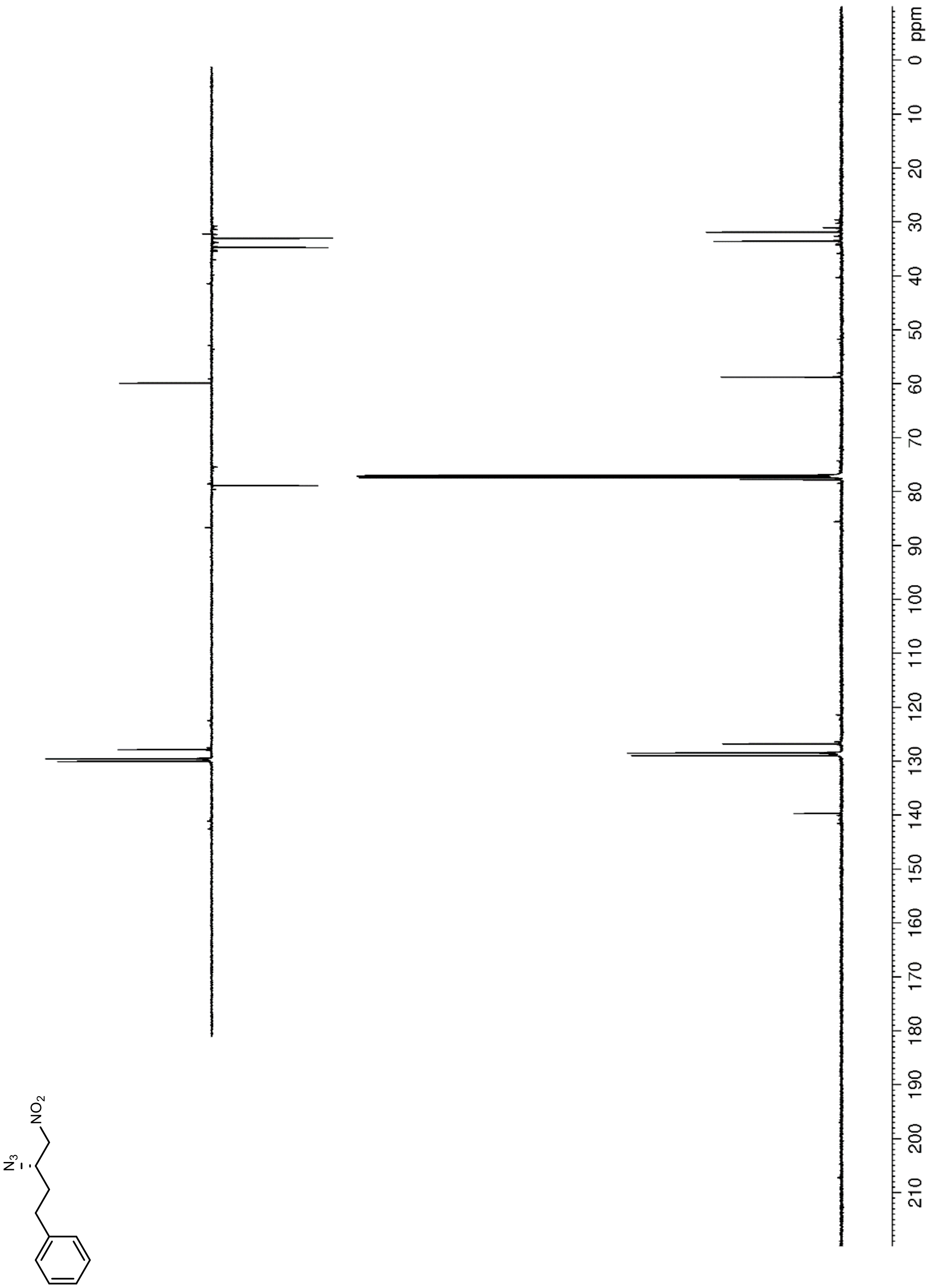

Figure 263. ^{1}H NMR (400 MHz, $CDCl_3$) of **2t**

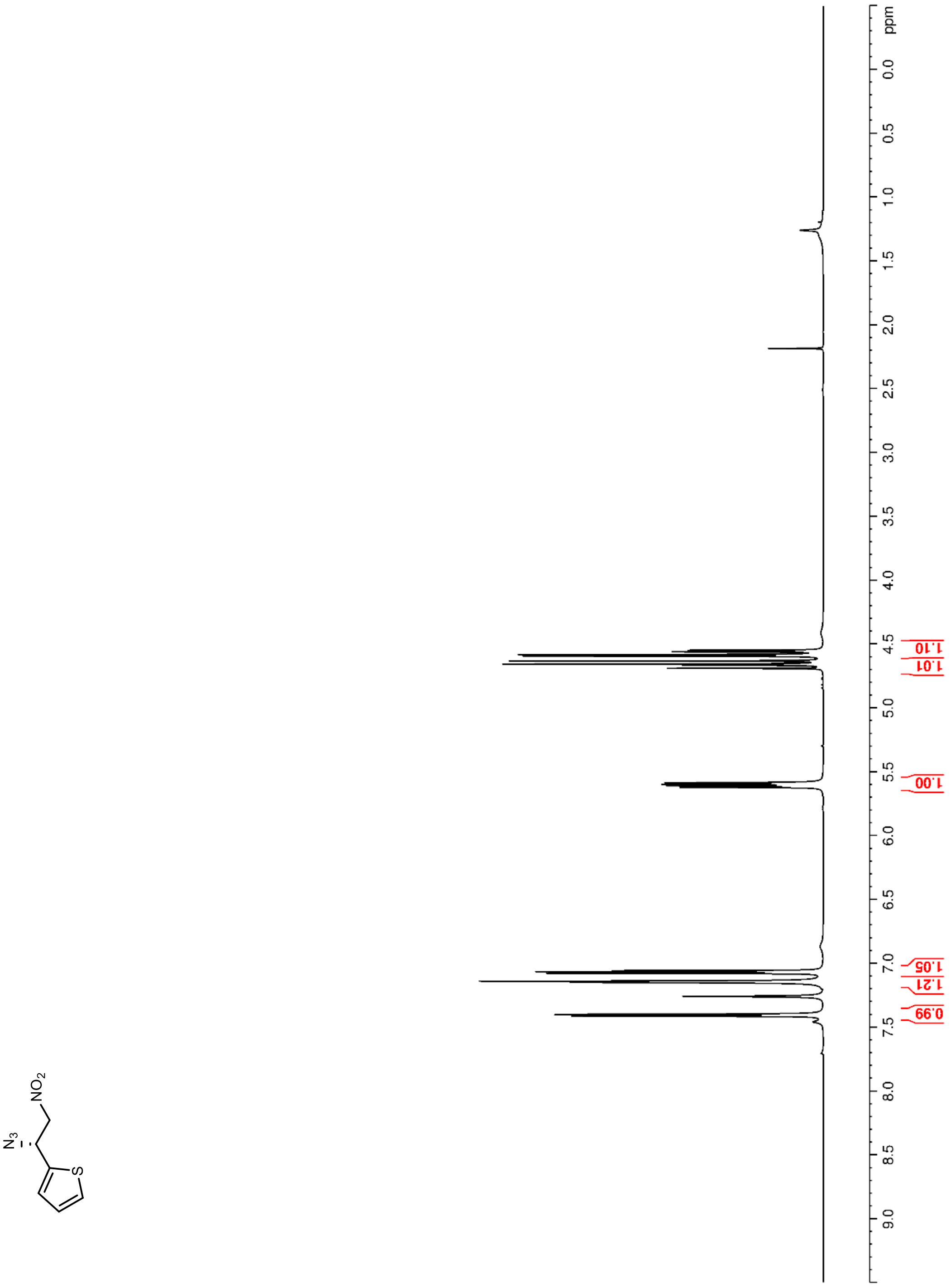

Figure 264. ^{13}C NMR (150 MHz, $CDCl_3$) of **2t**

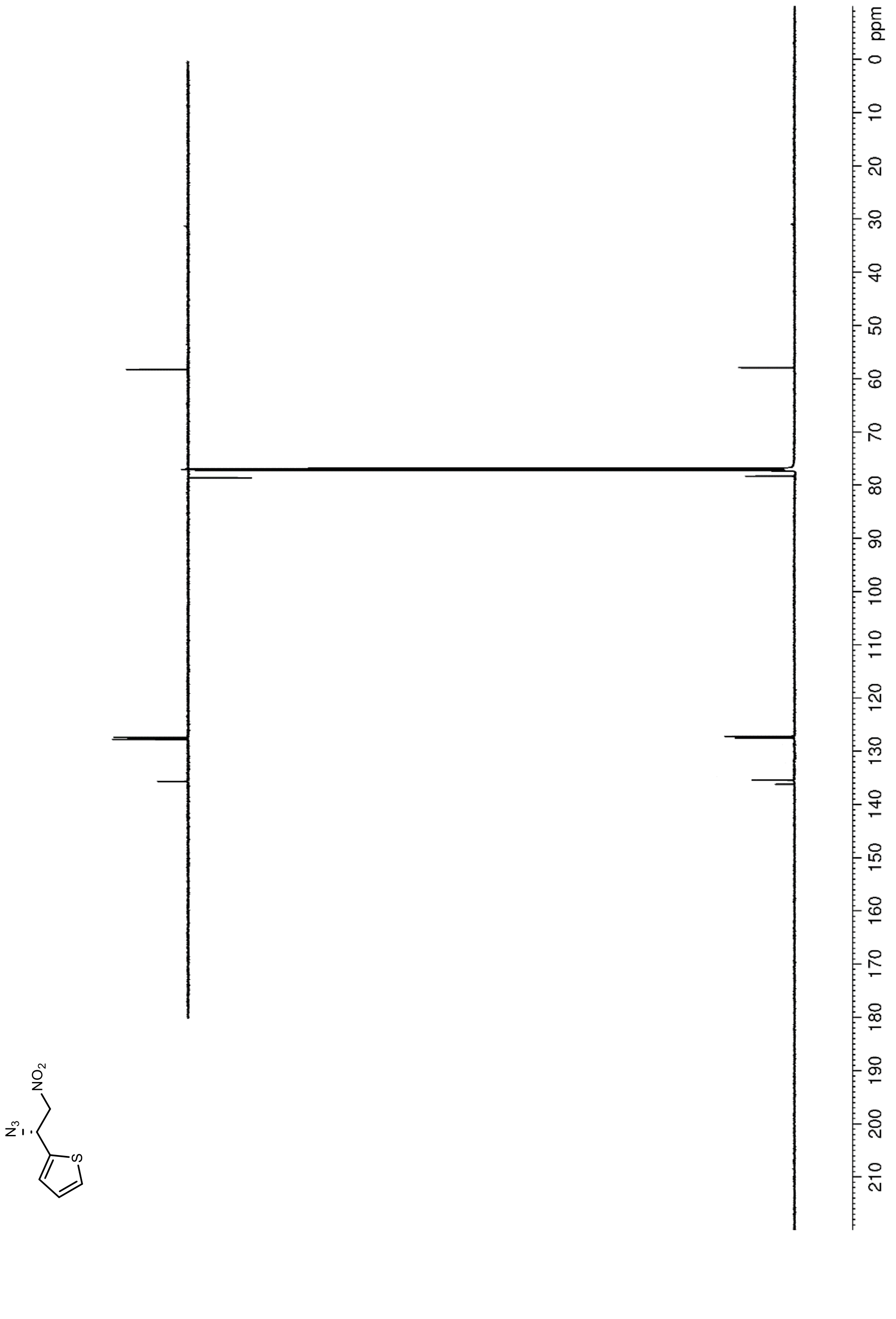

Figure 265. HPLC trace of **2a**

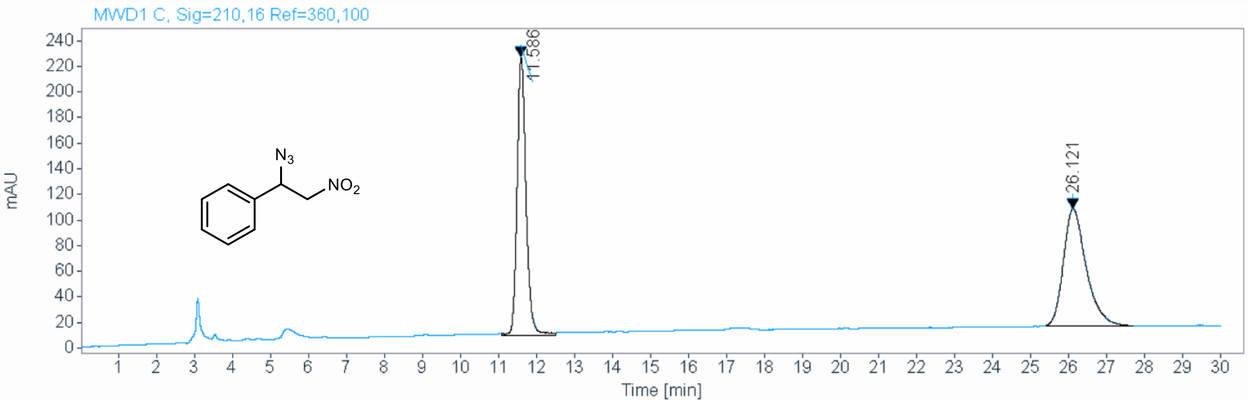

Signal: MWD1 C, Sig=210,16 Ref=360,100

RT [min]	Type	Width [min]	Area	Height	Area%	Name
11.586	MM	0.2729	3558.1868	217.3338	48.4069	
26.121	MM	0.6921	3792.3892	91.3291	51.5931	
		Sum	7350.5759			

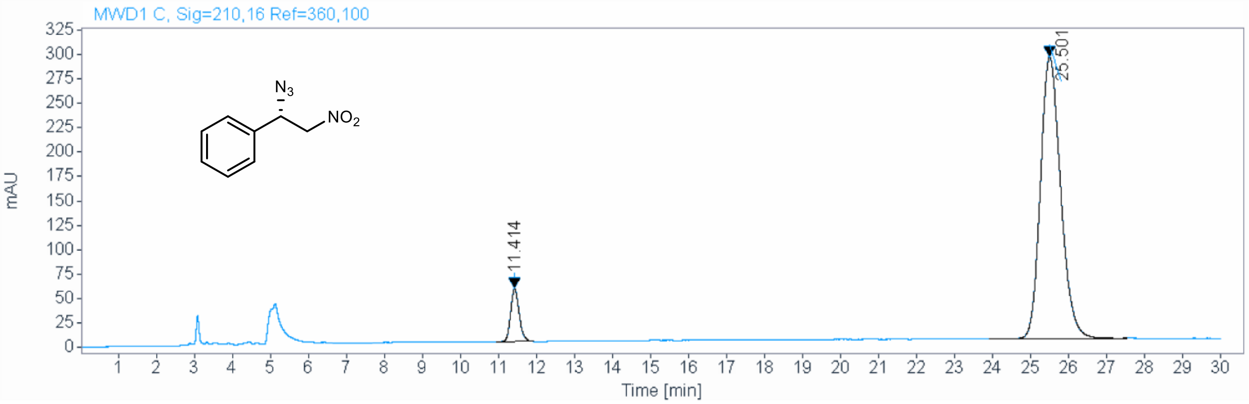

Signal: MWD1 C, Sig=210,16 Ref=360,100

RT [min]	Type	Width [min]	Area	Height	Area%	Name
11.414	MM	0.2585	842.0971	54.2841	7.3112	
25.501	MM	0.6170	10675.8691	288.3899	92.6888	
		Sum	11517.9662			

Figure 266. HPLC trace of **2b**[1]

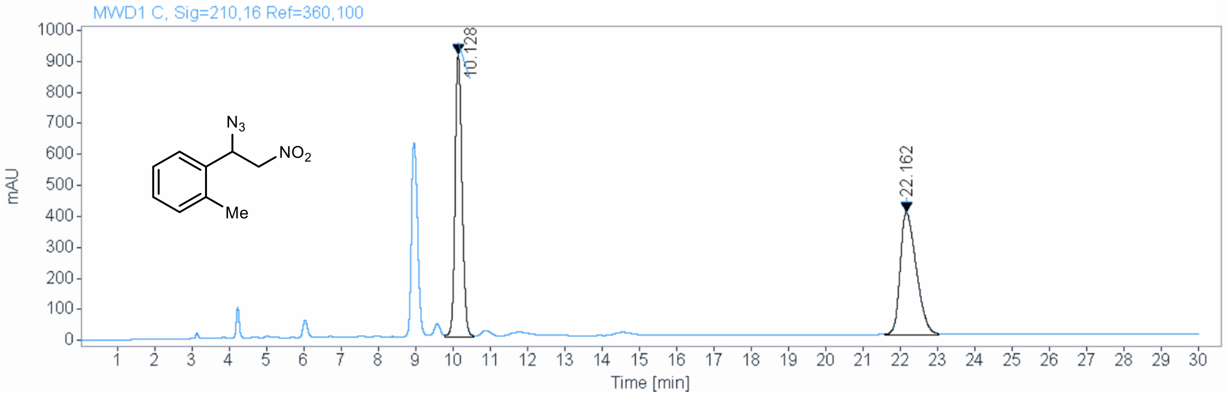

Signal: MWD1 C, Sig=210,16 Ref=360,100

RT [min]	Type	Width [min]	Area	Height	Area%	Name
10.128	MM	0.2154	11773.7676	910.9860	49.7876	
22.162	MM	0.5020	11874.2412	394.2177	50.2124	
		Sum	23648.0088			

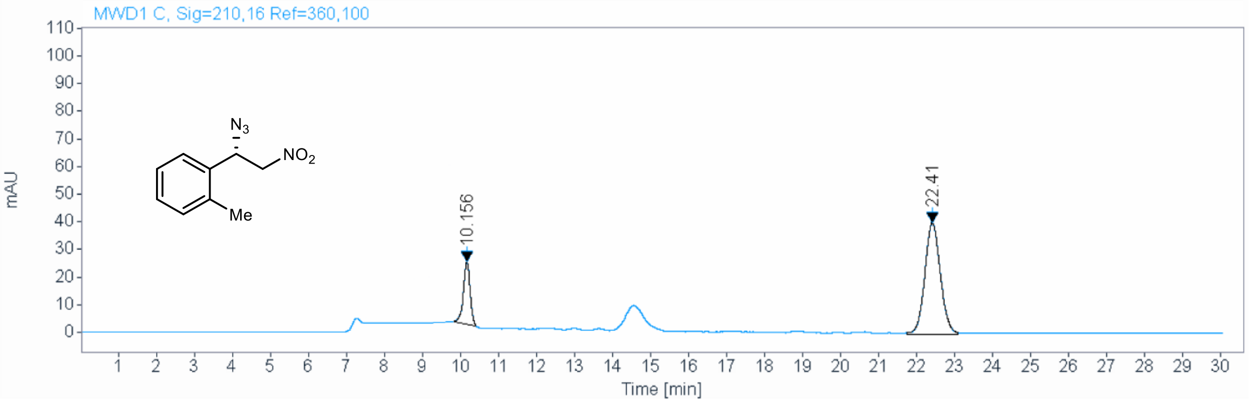

Signal: MWD1 C, Sig=210,16 Ref=360,100

RT [min]	Type	Width [min]	Area	Height	Area%	Name
10.156	MM	0.2043	275.4955	22.4711	19.3397	
22.410	VV	0.4249	1149.0151	40.2696	80.6603	
		Sum	1424.5107			

[1] In some preparations of the racemate standard, removal of unreacted nitrostyrene (or nitrostyrene geometric isomer) was challenging. It was also noted that these nitrostyrenes are intensely absorbing, enhancing their presence in the assay.

Figure 267. HPLC trace of **2c**

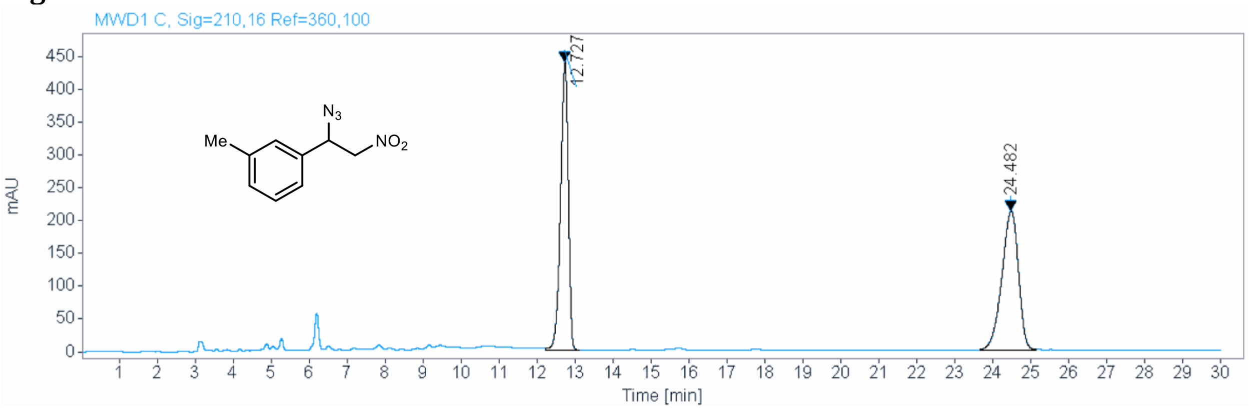

Signal:	MWD1 C, Sig=210,16 Ref=360,100					
RT [min]	Type	Width [min]	Area	Height	Area%	Name
12.727	VV	0.2208	6286.1216	439.6698	49.4539	
24.482	MM	0.5048	6424.9468	212.1309	50.5461	
		Sum	12711.0684			

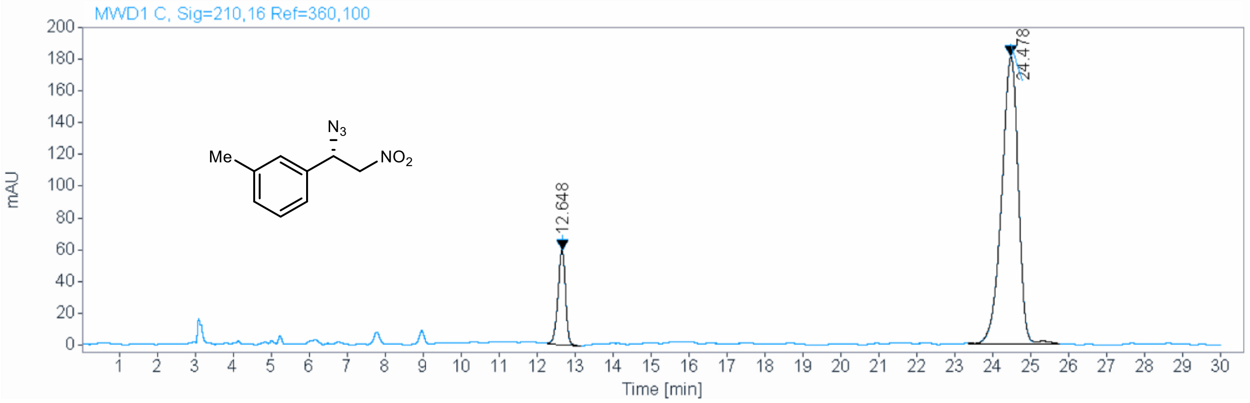

Signal:	MWD1 C, Sig=210,16 Ref=360,100					
RT [min]	Type	Width [min]	Area	Height	Area%	Name
12.648	BV	0.2138	832.4245	60.0250	13.1263	
24.478	MM	0.5063	5509.2236	181.3515	86.8737	
		Sum	6341.6481			

Figure 268. HPLC trace of **2d**

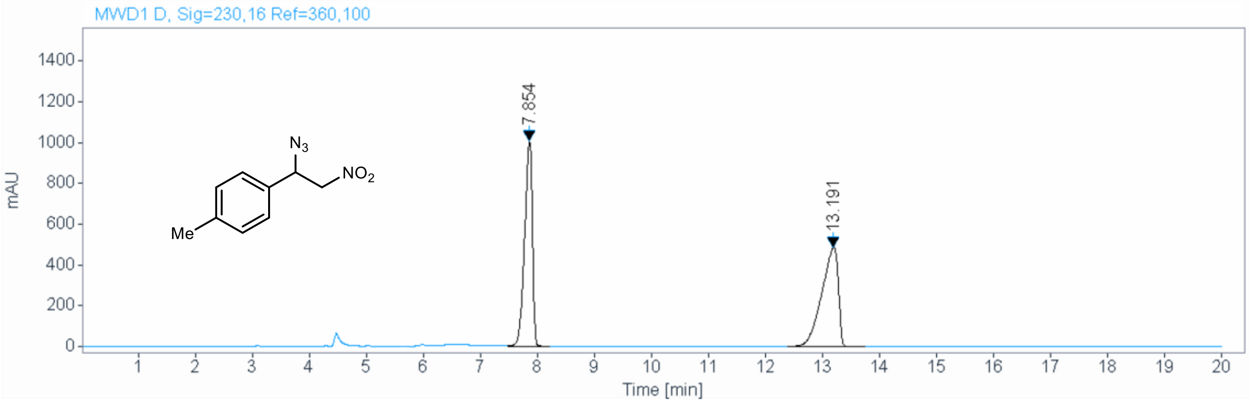

Signal:	MWD1 D, Sig=230,16 Ref=360,100					
RT [min]	Type	Width [min]	Area	Height	Area%	Name
7.854	VB	0.1485	9560.2998	1008.9910	49.4308	
13.191	BB	0.3126	9780.4639	486.3754	50.5692	
		Sum	19340.7637			

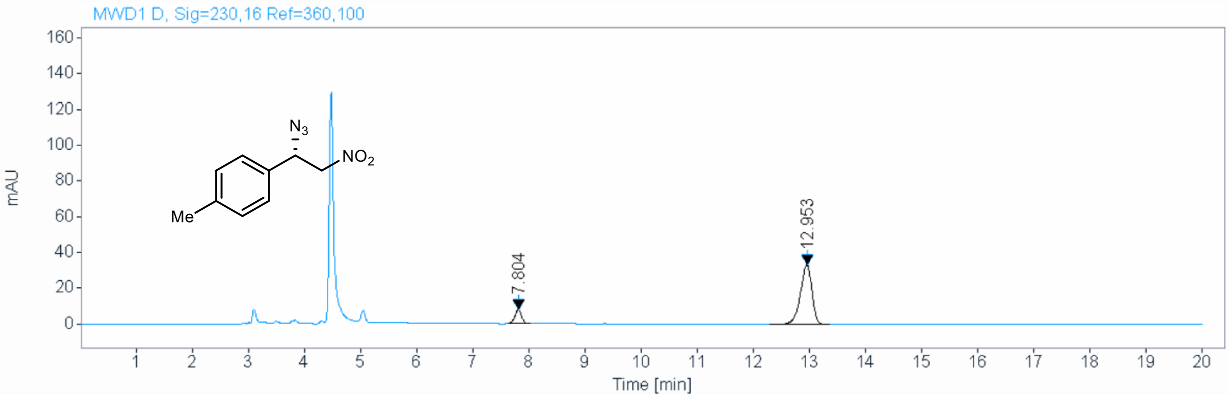

Signal:	MWD1 D, Sig=230,16 Ref=360,100					
RT [min]	Type	Width [min]	Area	Height	Area%	Name
7.804	MM	0.1371	63.3708	7.7055	11.7355	
12.953	MM	0.2387	476.6199	33.2783	88.2645	
		Sum	539.9907			

Figure 269. HPLC trace of **2e**[1]

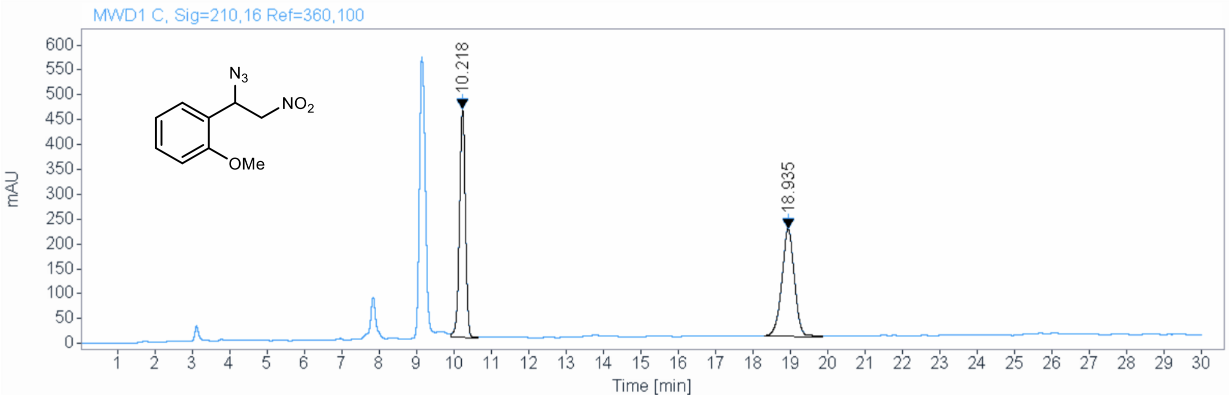

Signal:	MWD1 C, Sig=210,16 Ref=360,100					
RT [min]	Type	Width [min]	Area	Height	Area%	Name
10.218	MM	0.1834	5073.3813	460.9307	50.0713	
18.935	MM	0.3898	5058.9233	216.2976	49.9287	
		Sum	10132.3047			

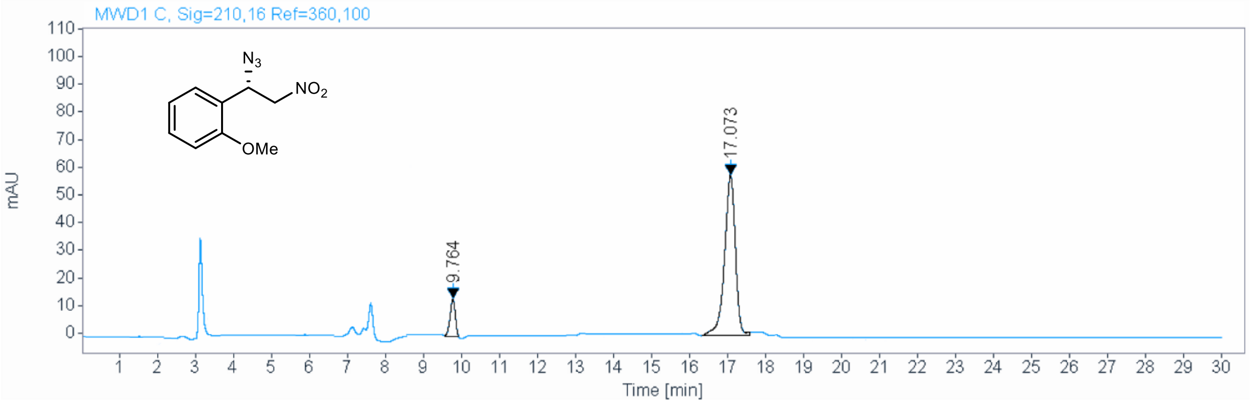

Signal:	MWD1 C, Sig=210,16 Ref=360,100					
RT [min]	Type	Width [min]	Area	Height	Area%	Name
9.764	MM	0.1584	129.8452	13.6635	10.1722	
17.073	MM	0.3291	1146.6273	58.0719	89.8278	
		Sum	1276.4725			

Figure 270. HPLC trace of **2f**[1]

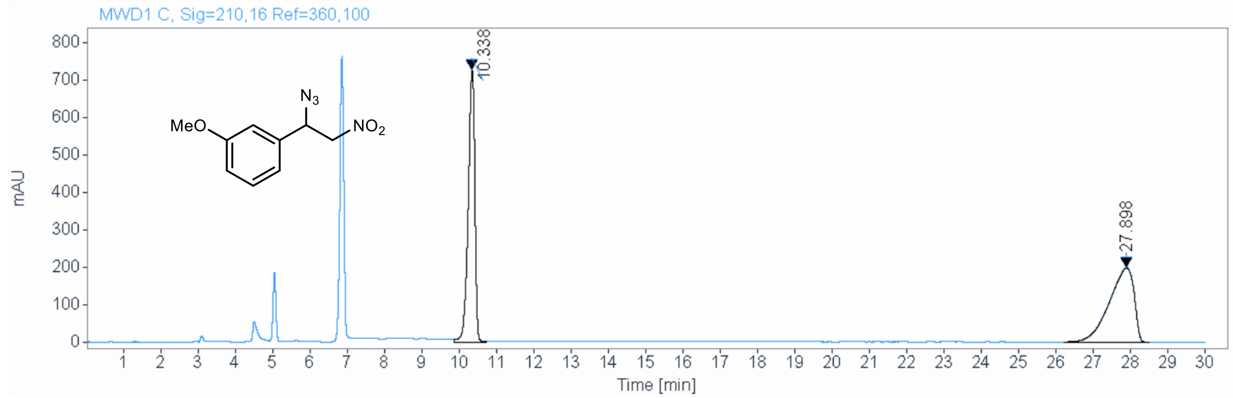

Signal:	MWD1 C, Sig=210,16 Ref=360,100					
RT [min]	Type	Width [min]	Area	Height	Area%	Name
10.338	VV	0.1935	9085.6523	728.6215	49.5487	
27.898	VV	0.6437	9251.1533	199.2513	50.4513	
		Sum	18336.8057			

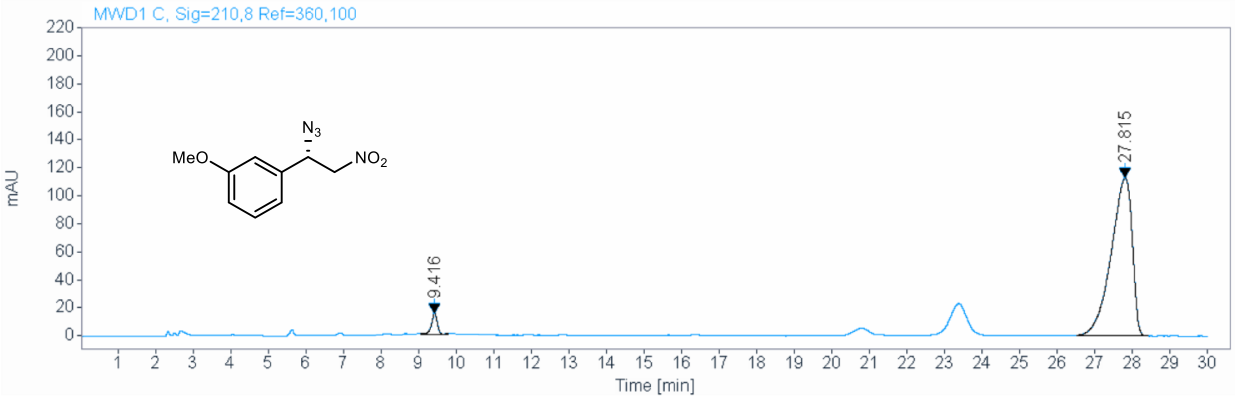

Signal:	MWD1 C, Sig=210,16 Ref=360,100					
RT [min]	Type	Width [min]	Area	Height	Area%	Name
9.416	MM	0.1872	171.0714	15.2282	3.7475	
27.815	MM	0.6488	4393.9219	112.8796	96.2525	
		Sum	4564.9933			

Figure 271. HPLC trace of **2g**

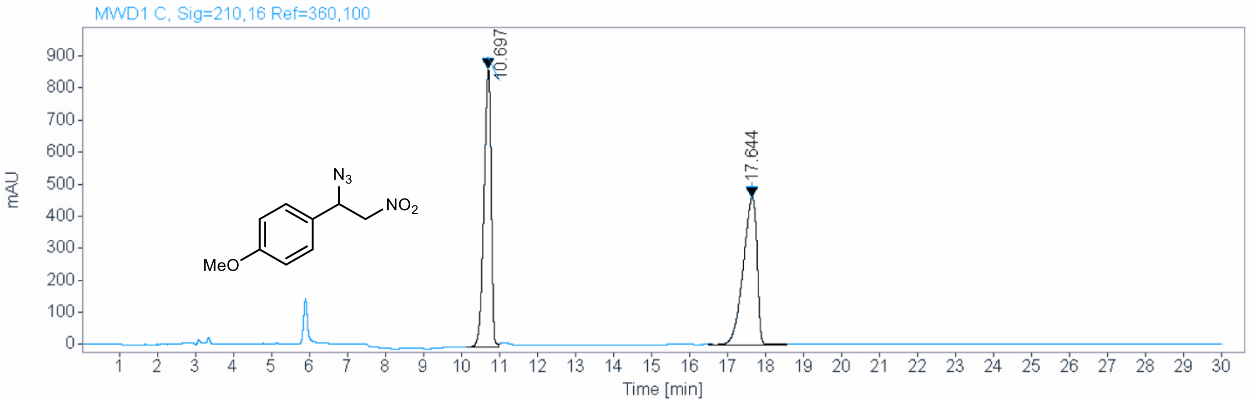

Signal: MWD1 C, Sig=210,16 Ref=360,100

RT [min]	Type	Width [min]	Area	Height	Area%	Name
10.697	MM	0.2184	11415.4580	871.2013	49.0845	
17.644	MM	0.4259	11841.2871	463.3349	50.9155	
		Sum	23256.7451			

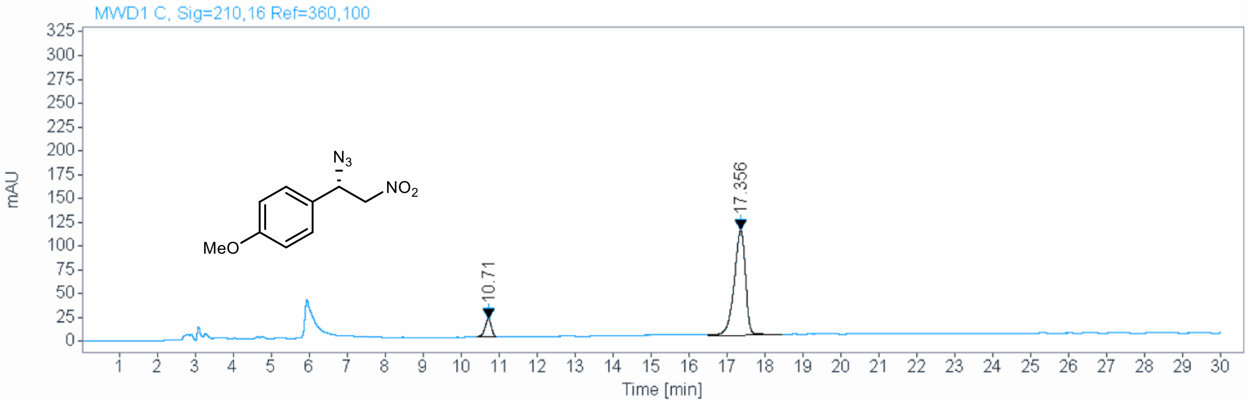

Signal: MWD1 C, Sig=210,16 Ref=360,100

RT [min]	Type	Width [min]	Area	Height	Area%	Name
10.710	MM	0.1896	212.7349	18.7017	8.6354	
17.356	MM	0.3417	2250.7739	109.7908	91.3646	
		Sum	2463.5088			

Figure 272. HPLC trace of **2h**

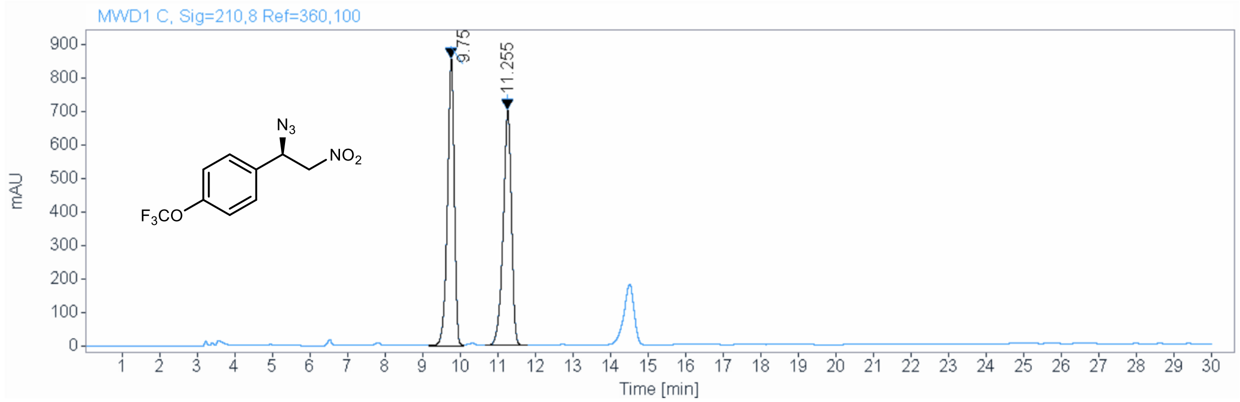

Signal: MWD1 C, Sig=210,8 Ref=360,100

RT [min]	Type	Width [min]	Area	Height	Area%	Name
9.750	VV	0.1912	10819.7500	857.3875	50.1106	
11.255	VB	0.2306	10771.9990	703.6676	49.8894	
		Sum	21591.7490			

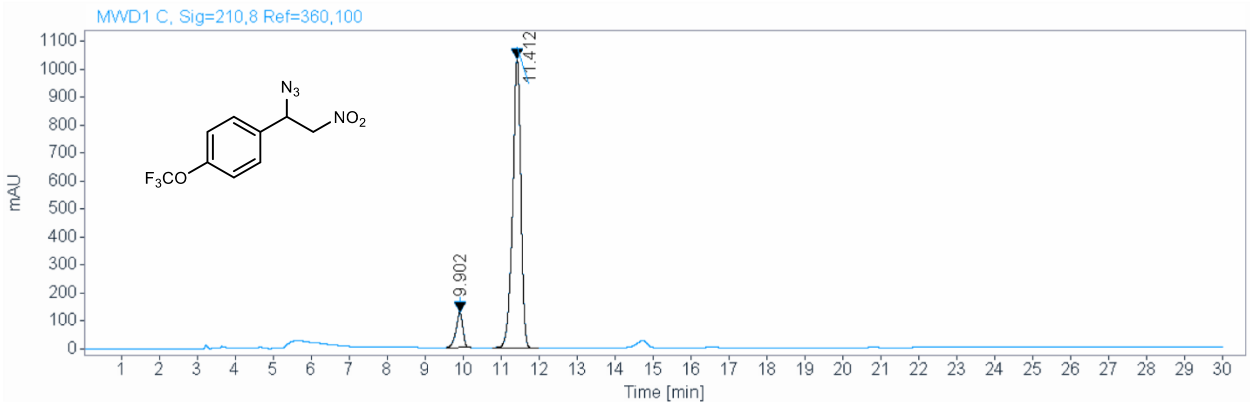

Signal: MWD1 C, Sig=210,8 Ref=360,100

RT [min]	Type	Width [min]	Area	Height	Area%	Name
9.902	MM	0.2046	1549.4381	126.2099	9.0232	
11.412	VB	0.2282	15622.3594	1034.4730	90.9768	
		Sum	17171.7975			

Figure 273. HPLC trace of **2i**[1]

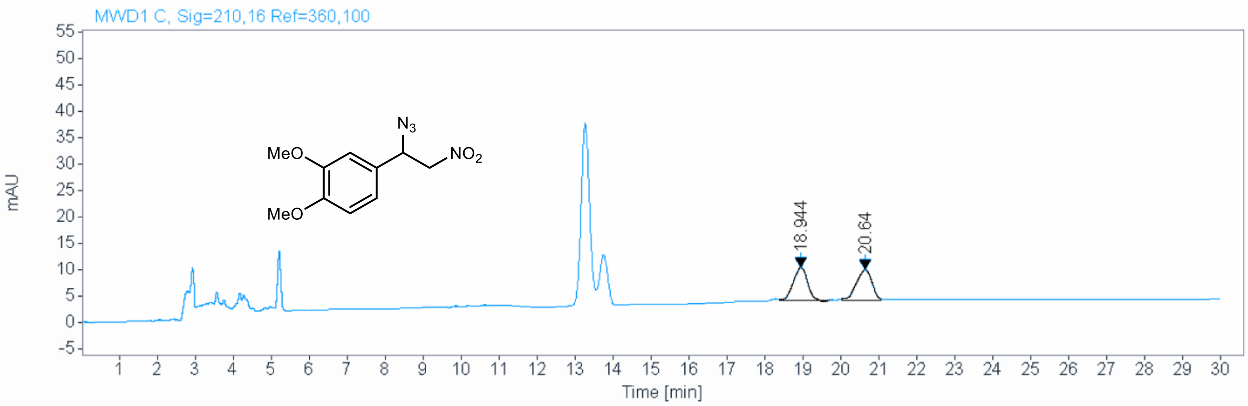

Signal: MWD1 C, Sig=210,16 Ref=360,100

RT [min]	Type	Width [min]	Area	Height	Area%	Name
18.944	MM	0.4452	165.2222	6.1849	49.3007	
20.640	MM	0.4771	169.9093	5.9359	50.6993	
		Sum	335.1315			

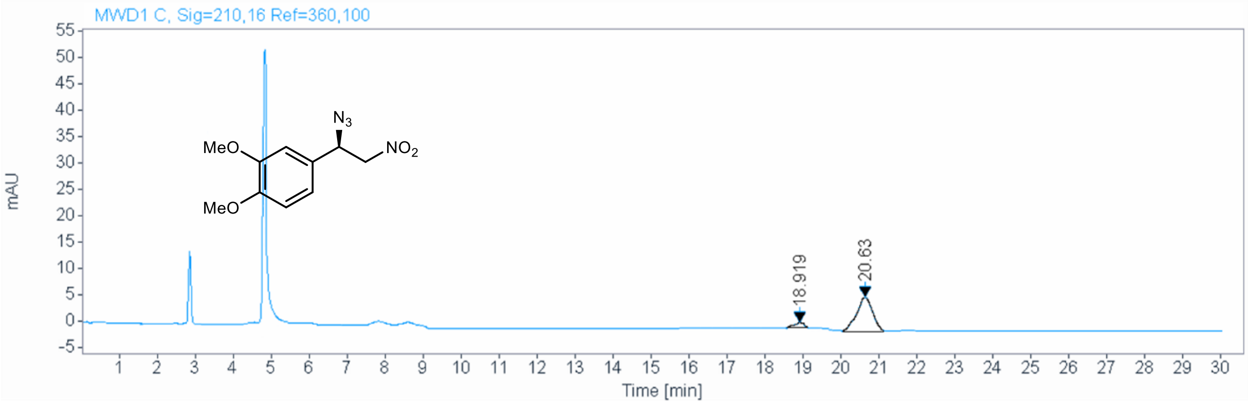

Signal: MWD1 C, Sig=210,16 Ref=360,100

RT [min]	Type	Width [min]	Area	Height	Area%	Name
18.919	MM	0.3057	18.3892	1.0025	8.9001	
20.630	MM	0.4881	188.2282	6.4273	91.0999	
		Sum	206.6174			

Figure 274. HPLC trace of **2j**

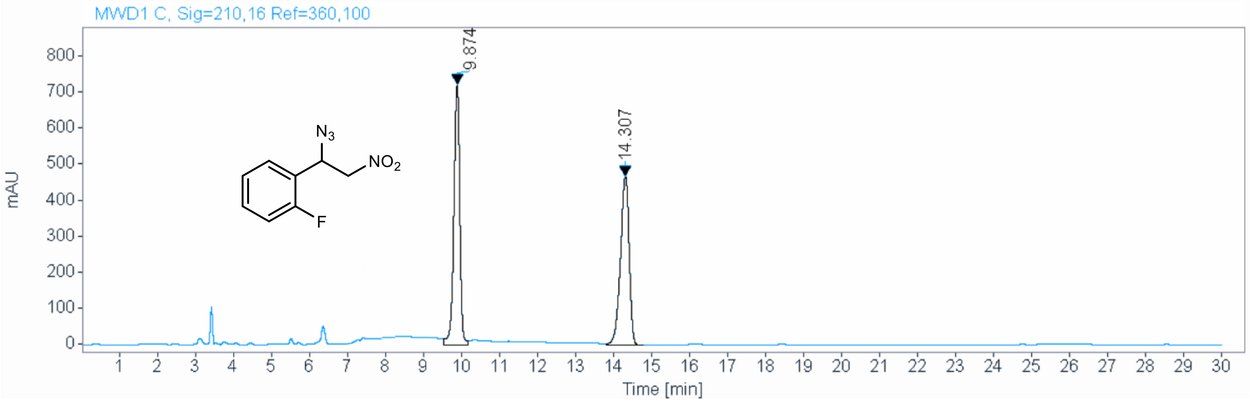

Signal:	MWD1 C, Sig=210,16 Ref=360,100					
RT [min]	Type	Width [min]	Area	Height	Area%	Name
9.874	VV	0.1659	7803.0767	723.3345	51.0644	
14.307	VV	0.2479	7477.7881	469.2250	48.9356	
		Sum	15280.8647			

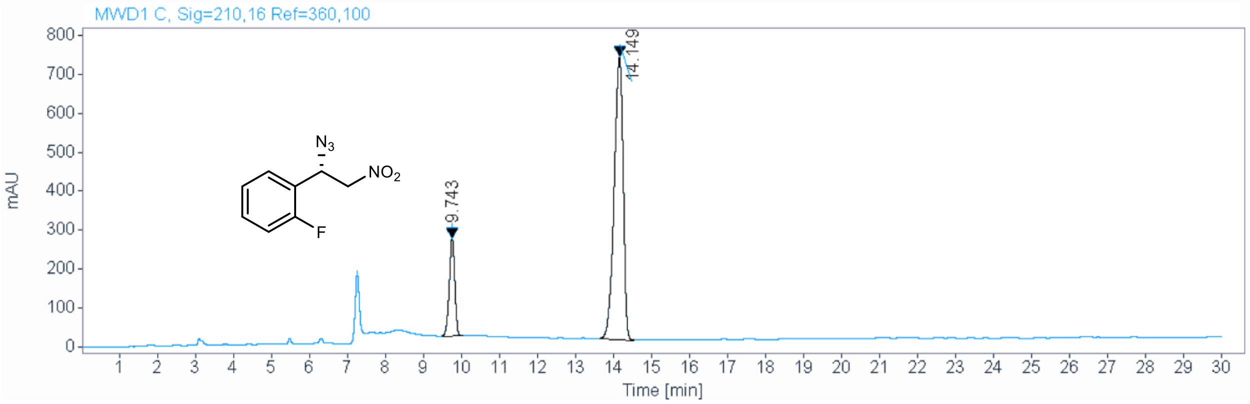

Signal:	MWD1 C, Sig=210,16 Ref=360,100					
RT [min]	Type	Width [min]	Area	Height	Area%	Name
9.743	MM	0.1667	2516.0999	251.4954	17.3383	
14.149	MM	0.2755	11995.7080	725.7960	82.6617	
		Sum	14511.8079			

Figure 275. HPLC trace of **2k**

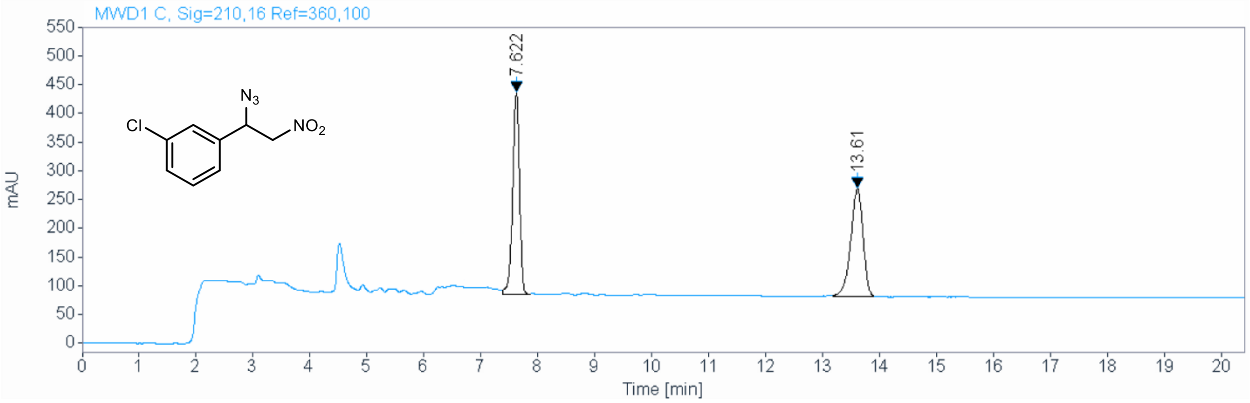

Signal: MWD1 C, Sig=210,16 Ref=360,100

RT [min]	Type	Width [min]	Area	Height	Area%	Name
7.622	MM	0.1386	2932.1545	352.6850	50.7178	
13.610	MM	0.2516	2849.1624	188.7206	49.2822	
		Sum	5781.3169			

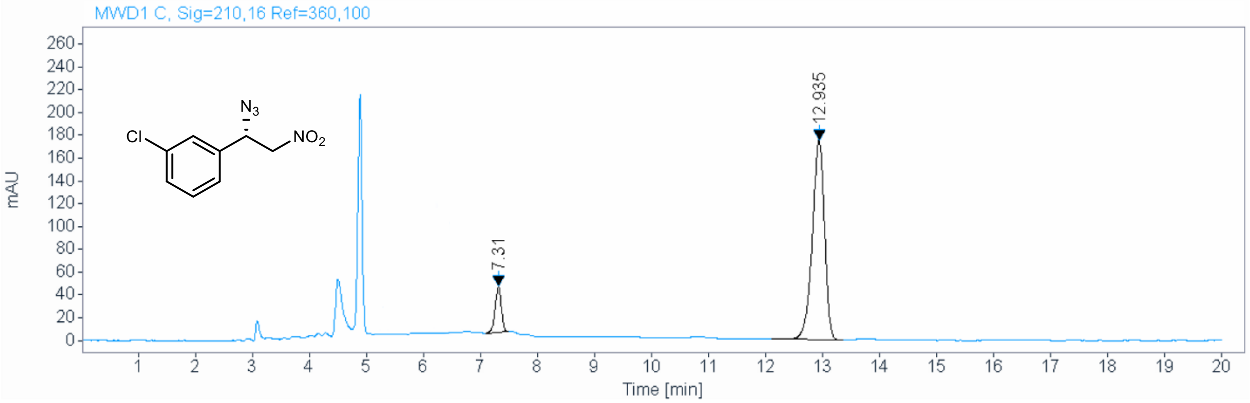

Signal: MWD1 C, Sig=210,16 Ref=360,100

RT [min]	Type	Width [min]	Area	Height	Area%	Name
7.310	MM	0.1260	303.8000	40.1930	10.4016	
12.935	MM	0.2493	2616.8999	174.9819	89.5984	
		Sum	2920.6999			

Figure 276. HPLC trace of **2l**

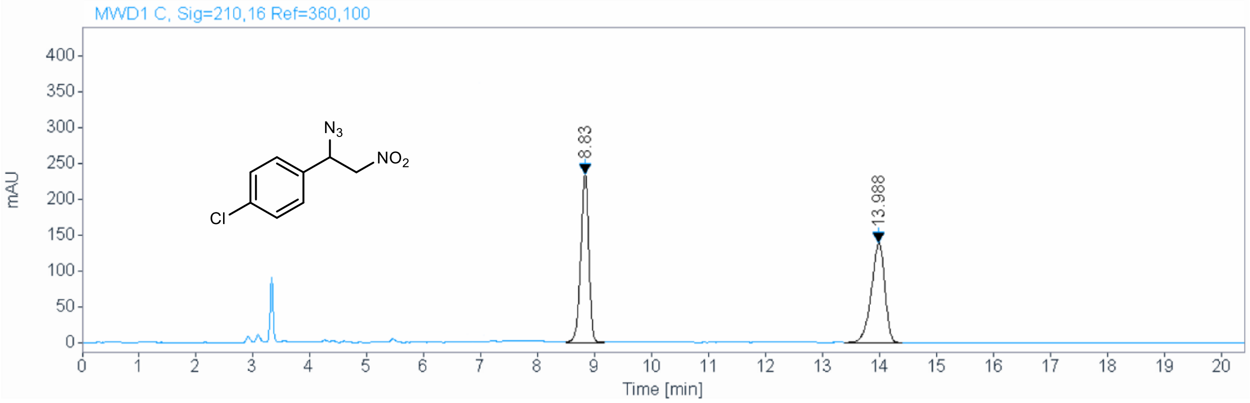

Signal: MWD1 C, Sig=210,16 Ref=360,100

RT [min]	Type	Width [min]	Area	Height	Area%	Name
8.830	VV	0.1522	2346.3052	235.6307	50.0043	
13.988	VV	0.2531	2345.8982	140.3141	49.9957	
		Sum	4692.2034			

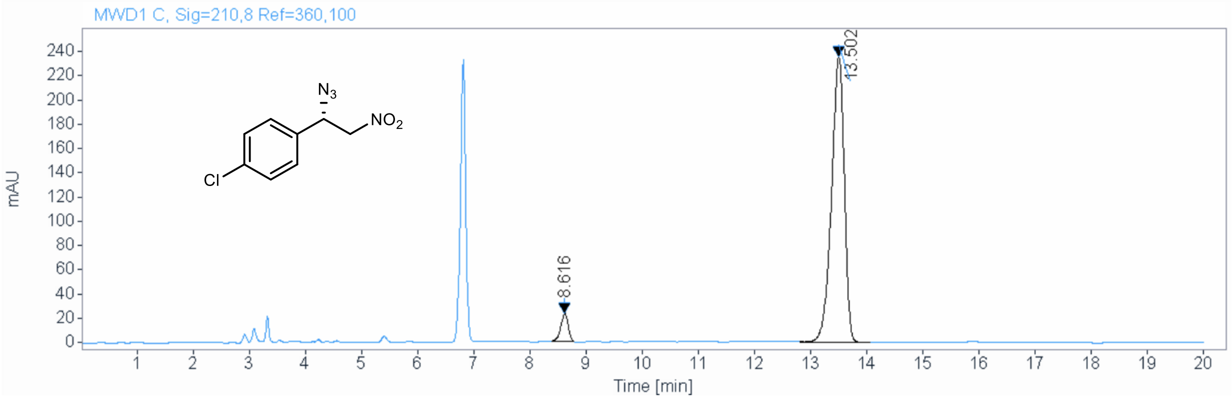

Signal: MWD1 C, Sig=210,8 Ref=360,100

RT [min]	Type	Width [min]	Area	Height	Area%	Name
8.616	MM	0.1501	204.7368	22.7385	5.1783	
13.502	MM	0.2651	3748.9824	235.6889	94.8217	
		Sum	3953.7192			

Figure 277. HPLC trace of **2m**

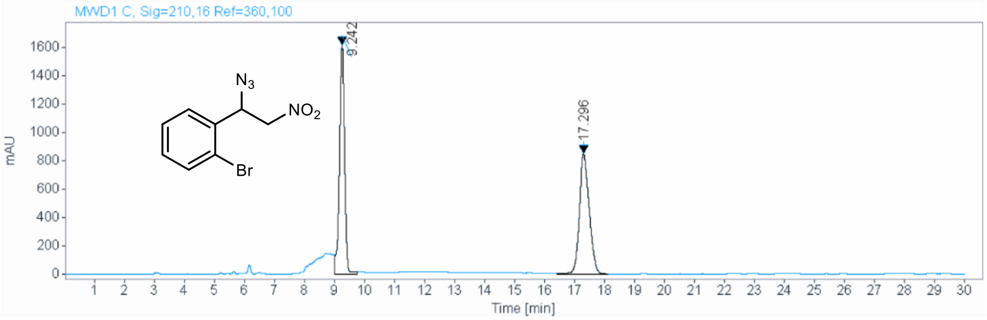

RT [min]	Type	Width [min]	Area	Height	Area%	Name
9.242	VV	0.1816	19088.4980	1618.8105	49.5804	
17.296	VV	0.3458	19411.5723	852.1639	50.4196	
		Sum	38500.0703			

Signal: MWD1 C, Sig=210,16 Ref=360,100

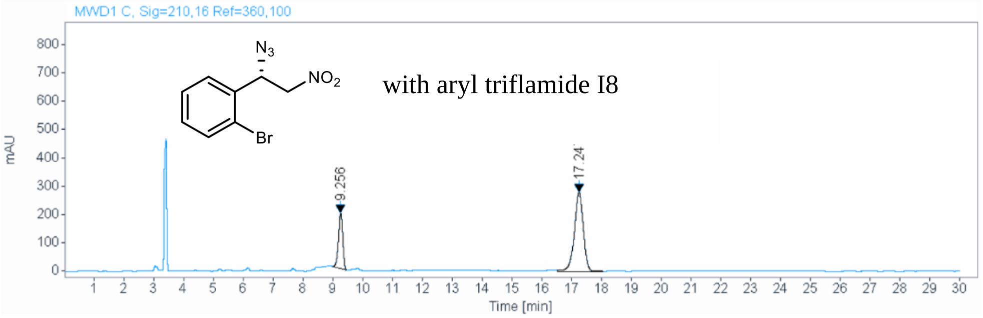

Signal: MWD1 C, Sig=210,16 Ref=360,100

RT [min]	Type	Width [min]	Area	Height	Area%	Name
9.256	MM	0.1644	1932.3685	195.8752	24.9687	
17.247	MM	0.3475	5806.7837	278.5425	75.0313	
		Sum	7739.1522			

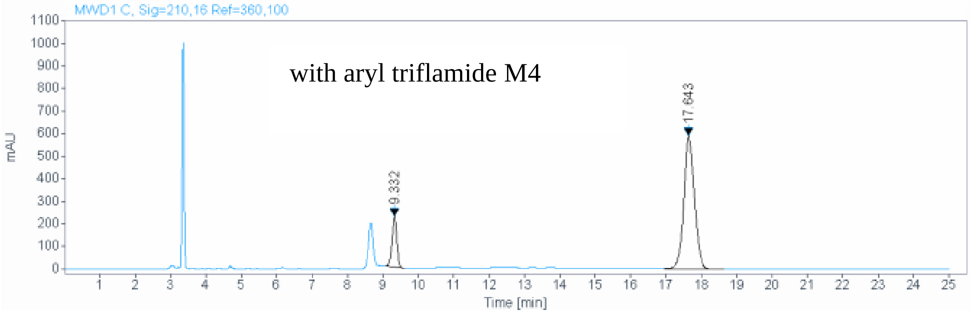

Signal: MWD1 C, Sig=210,16 Ref=360,100

RT [min]	Type	Width [min]	Area	Height	Area%	Name
9.332	MM	0.1638	2216.8025	225.5995	15.1131	
17.643	VV	0.3177	12451.2988	591.1878	84.8869	
		Sum	14668.1013			

Figure 278. HPLC trace of **2n**[1]

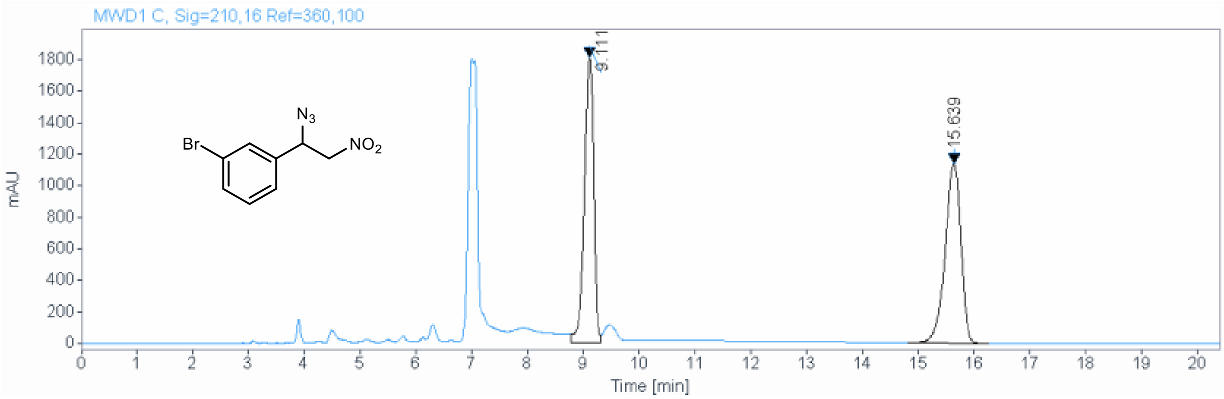

**Signal:**	MWD1 C, Sig=210,16 Ref=360,100				
**RT [min]**	**Type**	**Width [min]**	**Area**	**Height**	**Area% Name**
9.111	VV	0.1814	21009.9941	1810.5463	48.4185
15.639	VV	0.3070	22382.4922	1140.0751	51.5815
		Sum	43392.4863		

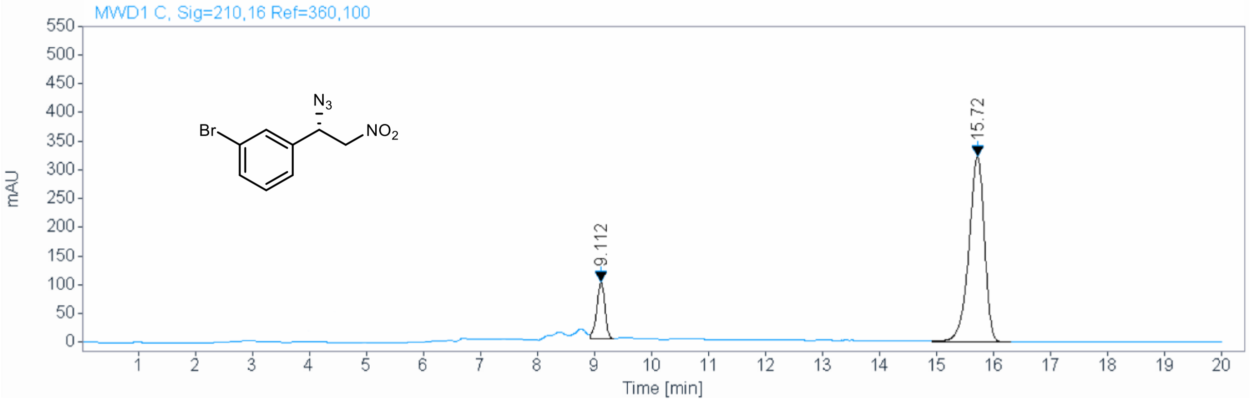

**Signal:**	MWD1 C, Sig=210,16 Ref=360,100				
**RT [min]**	**Type**	**Width [min]**	**Area**	**Height**	**Area% Name**
9.112	MM	0.1639	968.7303	98.4999	13.5124
15.720	VV	0.2933	6200.4478	323.9643	86.4876
		Sum	7169.1780		

Figure 279. HPLC trace of **2o**

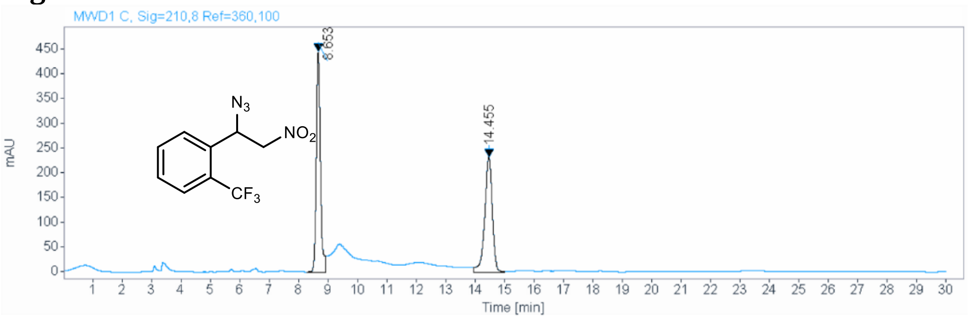

Signal: MWD1 C, Sig=210,8 Ref=360,100

RT [min]	Type	Width [min]	Area	Height	Area%	Name
8.653	VV	0.1495	4356.0996	447.5947	51.0819	
14.455	VB	0.2705	4171.5796	233.3972	48.9181	
		Sum	8527.6792			

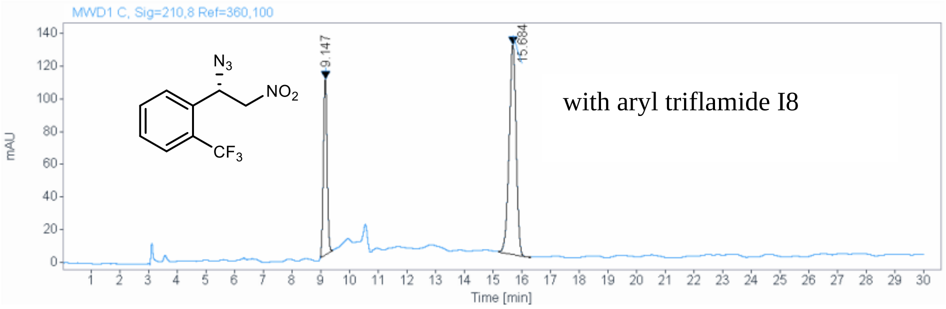

Signal: MWD1 C, Sig=210,8 Ref=360,100

RT [min]	Type	Width [min]	Area	Height	Area%	Name
9.147	MM	0.1502	972.3218	107.9214	30.9172	
15.684	MM	0.2819	2172.6045	128.4643	69.0828	
		Sum	3144.9263			

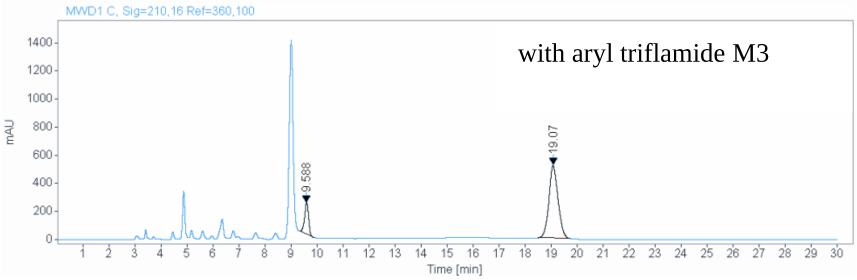

Signal: MWD1 C, Sig=210,16 Ref=360,100

RT [min]	Type	Width [min]	Area	Height	Area%	Name
9.588	MM	0.1791	2446.0681	227.5774	16.3349	
19.070	MM	0.4031	12528.4385	518.0085	83.6651	
		Sum	14974.5066			

Figure 280. HPLC trace of **2p**

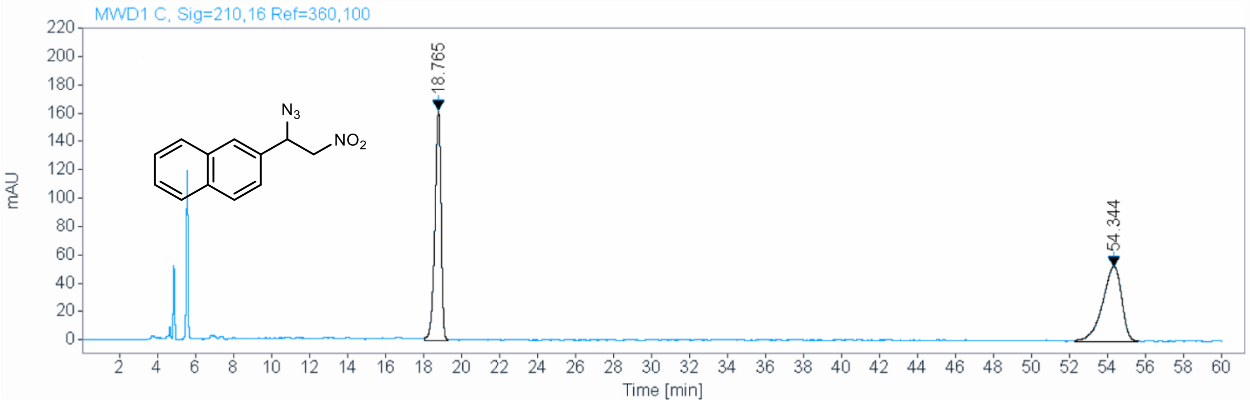

Signal: MWD1 C, Sig=210,16 Ref=360,100

RT [min]	Type	Width [min]	Area	Height	Area%	Name
18.765	BV	0.3466	3689.1072	162.6895	49.9039	
54.344	VV	0.8235	3703.3098	52.8487	50.0961	
		Sum	7392.4170			

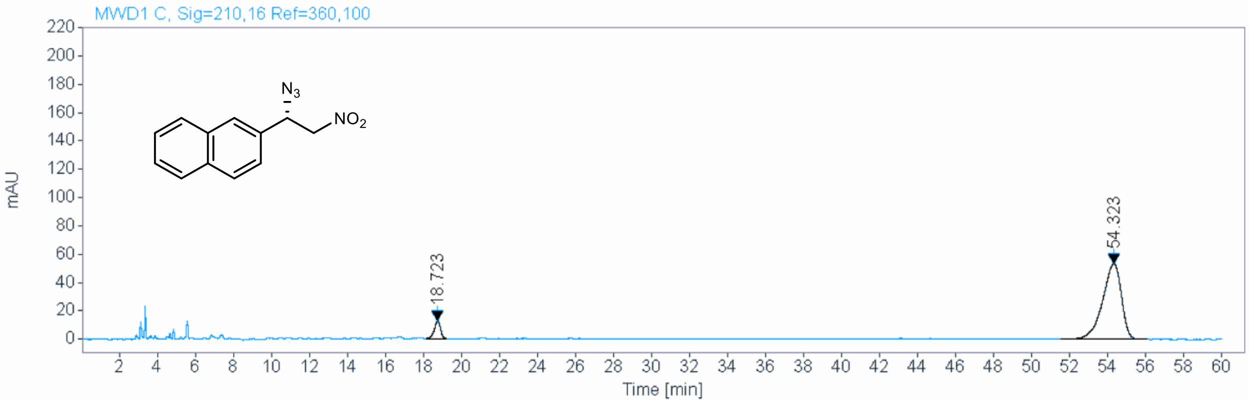

Signal: MWD1 C, Sig=210,16 Ref=360,100

RT [min]	Type	Width [min]	Area	Height	Area%	Name
18.723	MM	0.3655	278.6481	12.7058	7.0158	
54.323	MM	1.1546	3693.0708	53.3105	92.9842	
		Sum	3971.7189			

Figure 281. HPLC trace of **2q**[1]

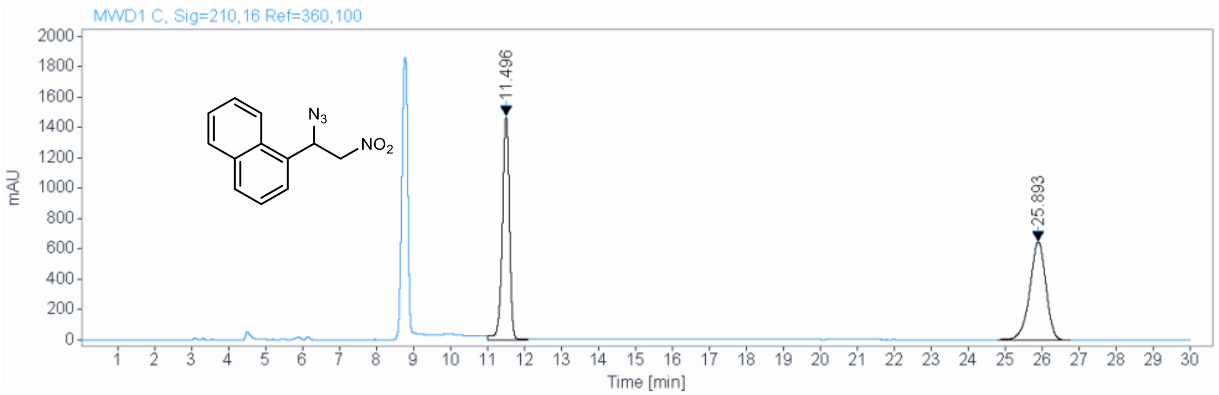

Signal: MWD1 C, Sig=210,16 Ref=360,100

RT [min]	Type	Width [min]	Area	Height	Area%	Name
11.496	VV	0.2063	19850.9512	1481.9366	50.1866	
25.893	VV	0.4669	19703.3047	647.6182	49.8134	
		Sum	39554.2559			

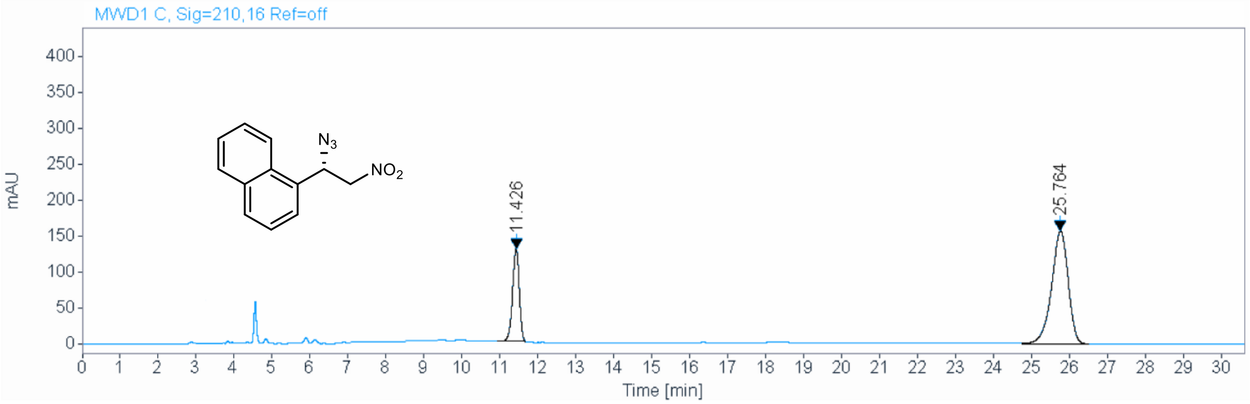

Signal: MWD1 C, Sig=210,16 Ref=off

RT [min]	Type	Width [min]	Area	Height	Area%	Name
11.426	MM	0.2041	1582.0746	129.1632	24.4839	
25.764	VV	0.4618	4879.6270	158.2458	75.5161	
		Sum	6461.7015			

Figure 282. HPLC trace of **2r**

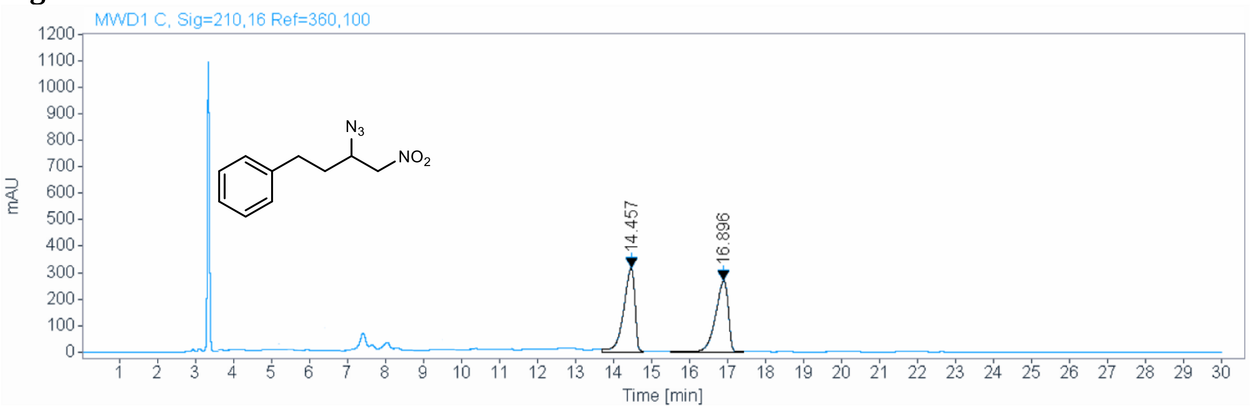

Signal: MWD1 C, Sig=210,16 Ref=360,100

RT [min]	Type	Width [min]	Area	Height	Area%	Name
14.457	VV	0.3238	6639.4492	317.7753	50.7104	
16.896	VV	0.3676	6453.4336	269.2358	49.2896	
		Sum	13092.8828			

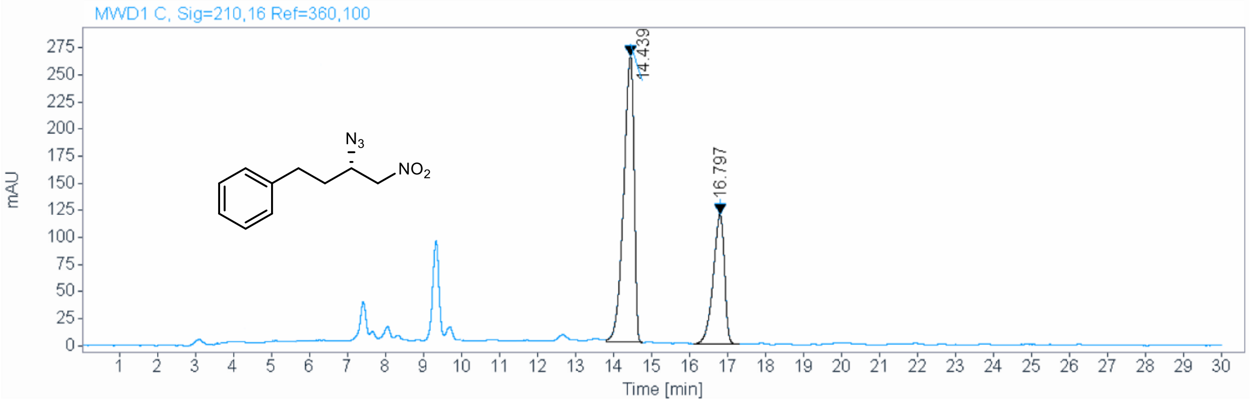

Signal: MWD1 C, Sig=210,16 Ref=360,100

RT [min]	Type	Width [min]	Area	Height	Area%	Name
14.439	MM	0.3168	5008.8887	263.5440	67.2375	
16.797	MM	0.3401	2440.6582	119.5900	32.7625	
		Sum	7449.5469			

Figure 283. HPLC trace of **2s**

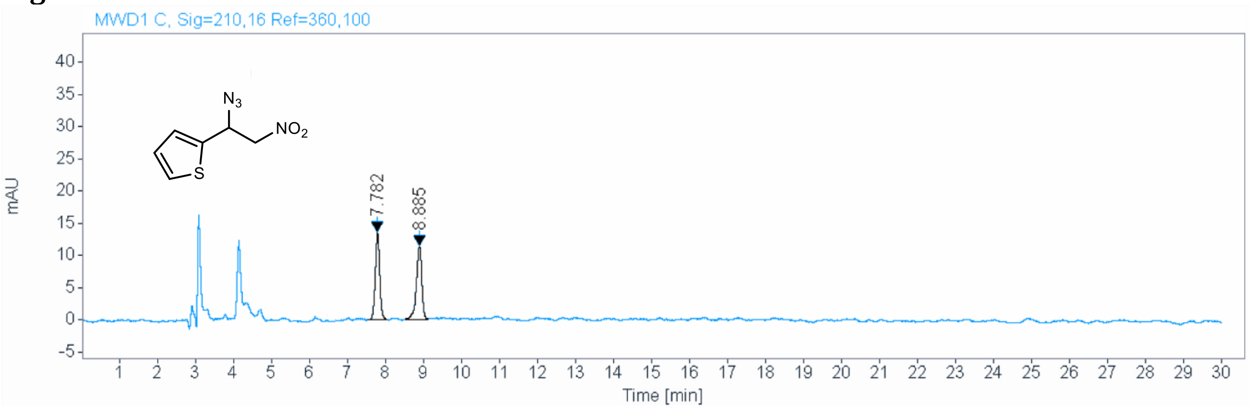

Signal: MWD1 C, Sig=210,16 Ref=360,100

RT [min]	Type	Width [min]	Area	Height	Area%	Name
7.782	MM	0.1334	108.0185	13.4949	49.2026	
8.885	MM	0.1624	111.5197	11.4474	50.7974	
		Sum	219.5382			

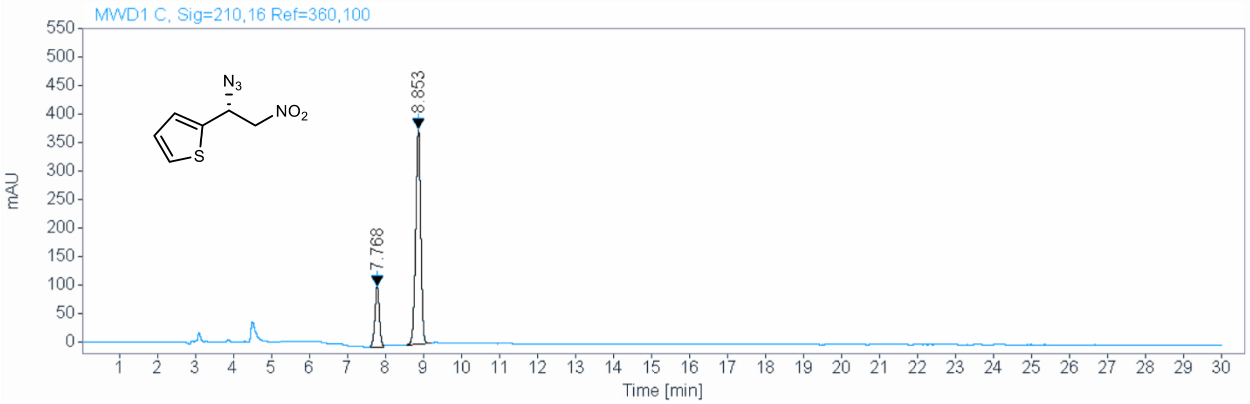

Signal: MWD1 C, Sig=210,16 Ref=360,100

RT [min]	Type	Width [min]	Area	Height	Area%	Name
7.768	MM	0.1356	882.3692	108.4876	20.0599	
8.853	MM	0.1546	3516.2947	379.1107	79.9401	
		Sum	4398.6639			

Figure 284. HPLC trace of **2t**[1]

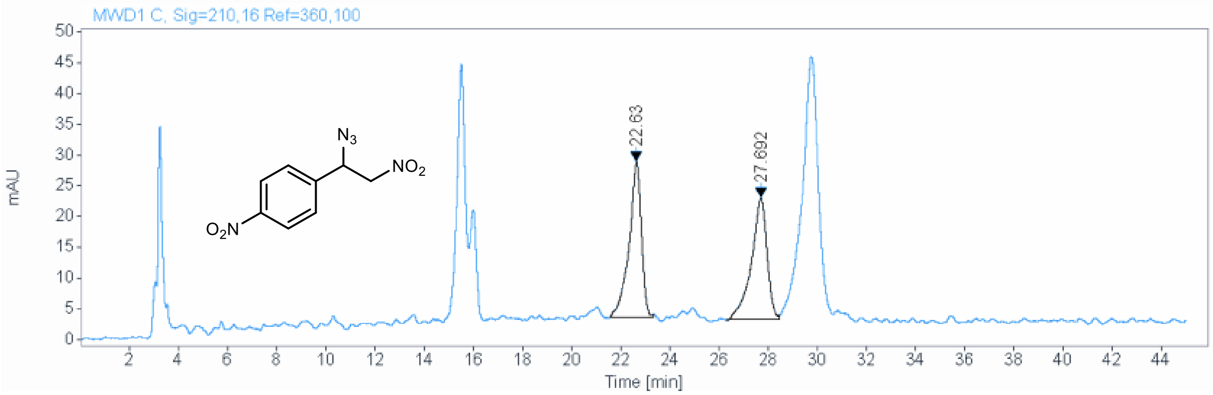

Signal: MWD1 C, Sig=210,16 Ref=360,100

RT [min]	Type	Width [min]	Area	Height	Area%	Name
22.630	MM	0.5808	878.6578	25.2155	50.2702	
27.692	MM	0.7362	869.2138	19.6772	49.7298	
		Sum	1747.8716			

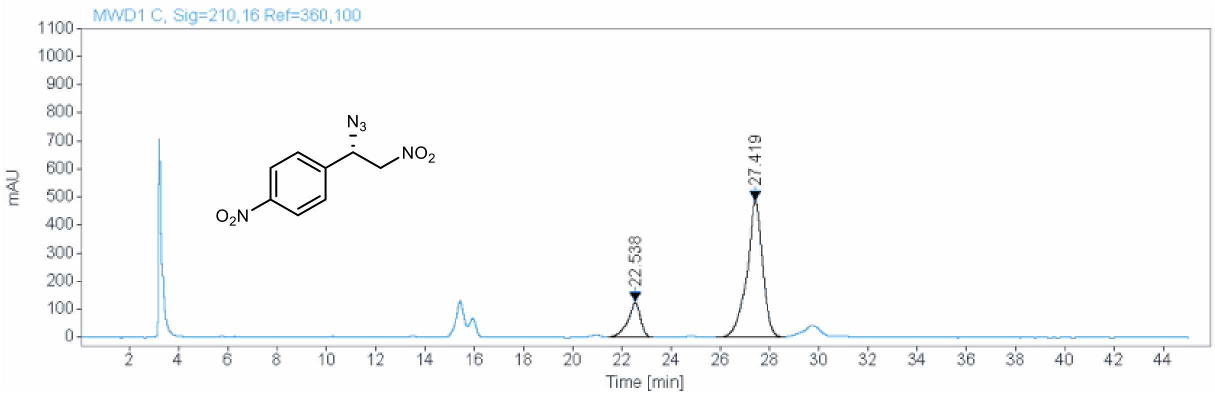

Signal: MWD1 C, Sig=210,16 Ref=360,100

RT [min]	Type	Width [min]	Area	Height	Area%	Name
22.538	MM	0.5958	4441.0728	124.2317	16.9833	
27.419	MM	0.7416	21708.5781	487.8741	83.0167	
		Sum	26149.6509			
